# Supplementary material for: Latent Association Between Diets and Glioma Risk: A Mendelian Randomization Analysis
Source: Nutrients. 2025 Feb 5;17(3):582. doi: 10.3390/nu17030582 (PMC11819737; doi:10.3390/nu17030582)
Supplement: Supplementary file 1 [file nutrients-17-00582-s001.zip › nutrients-3462880-supplementary/Sup_30.pdf]

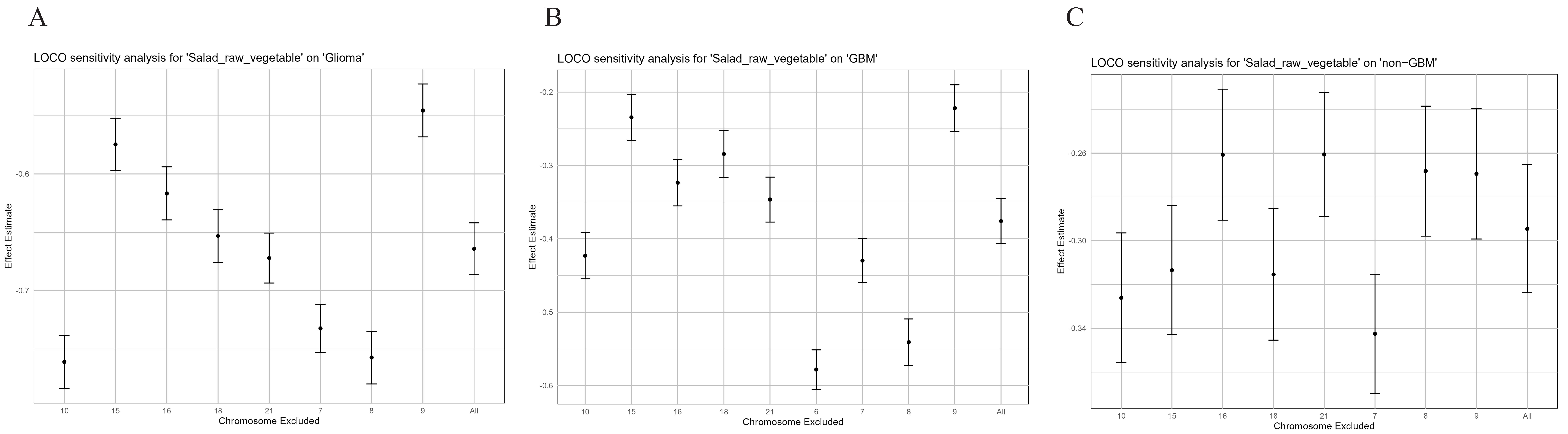
A
LOCO sensitivity analysis for 'Salad_raw_vegetable' on 'Glioma'
Effect Estimate
-0.6
-0.7
Chromosome Excluded
10
15
16
18
21
7
8
9
All
B
LOCO sensitivity analysis for 'Salad_raw_vegetable' on 'GBM'
Effect Estimate
-0.2
-0.3
-0.4
-0.5
-0.6
Chromosome Excluded
10
15
16
18
21
6
7
8
9
All
C
LOCO sensitivity analysis for 'Salad_raw_vegetable' on 'non−GBM'
Effect Estimate
-0.26
-0.30
-0.34
Chromosome Excluded
10
15
16
18
21
7
8
9
All
