# Supplementary figures and images for: Latent Association Between Diets and Glioma Risk: A Mendelian Randomization Analysis
Source: Nutrients. 2025 Feb 5;17(3):582. doi: 10.3390/nu17030582 (PMC11819737; doi:10.3390/nu17030582)

A

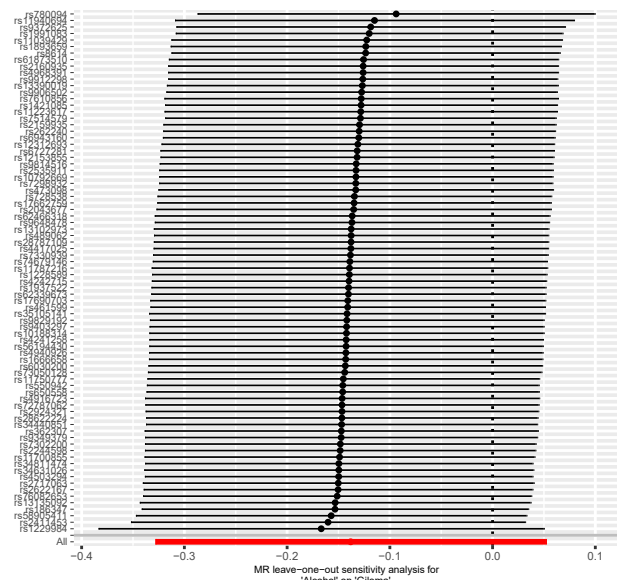

B

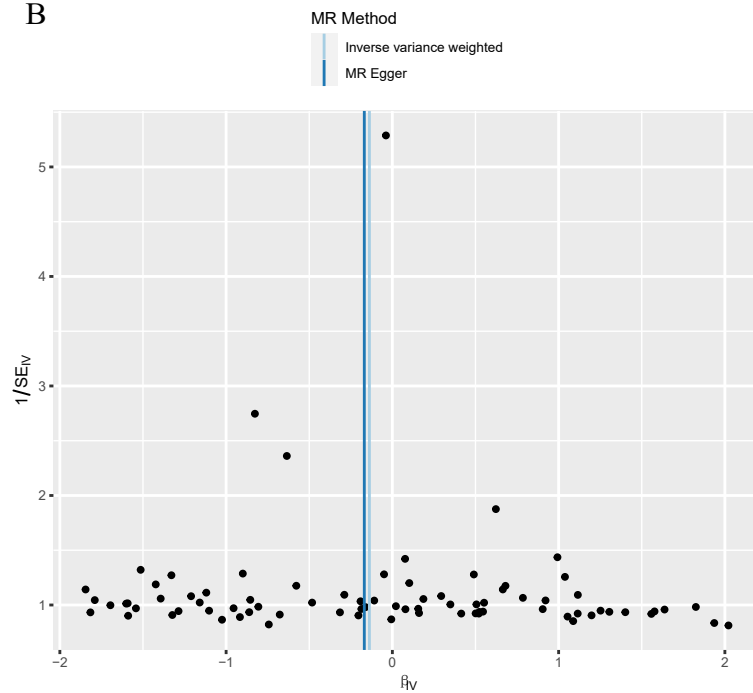

C

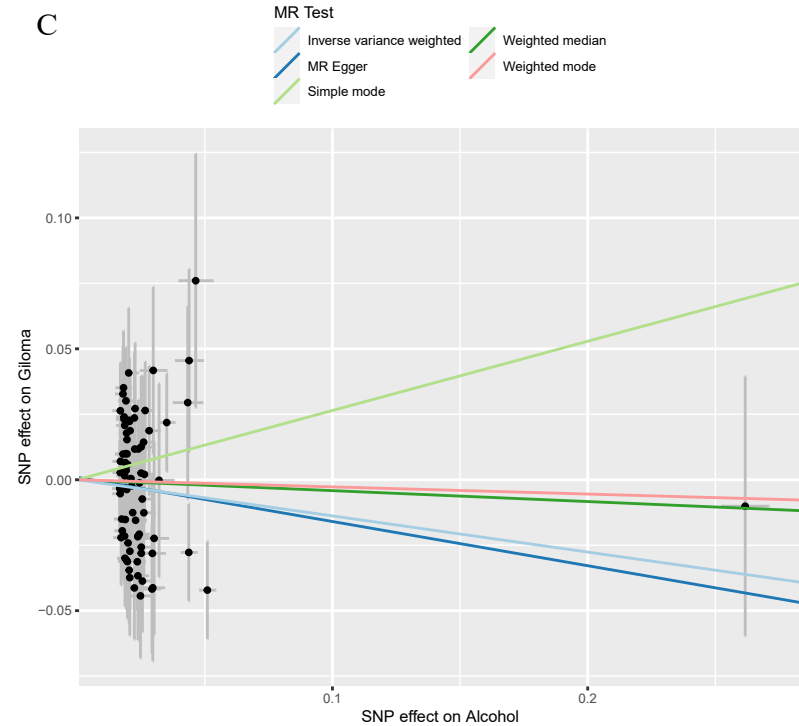

D

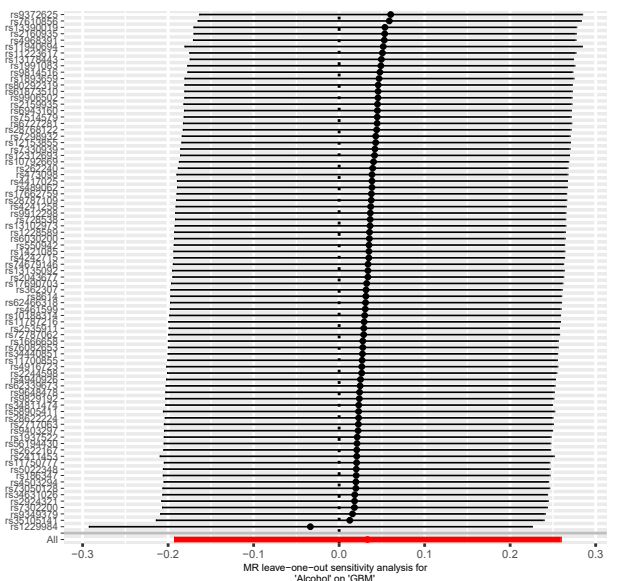

E

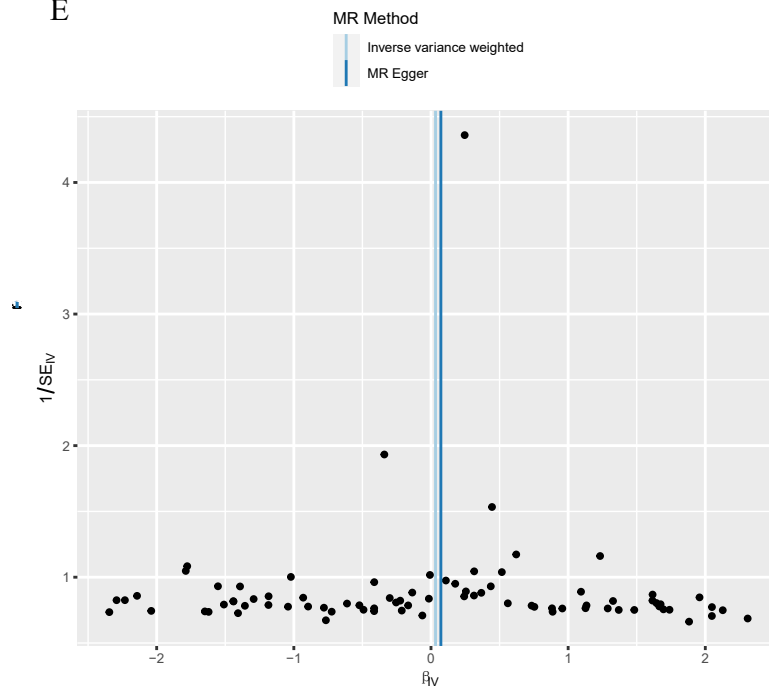

F

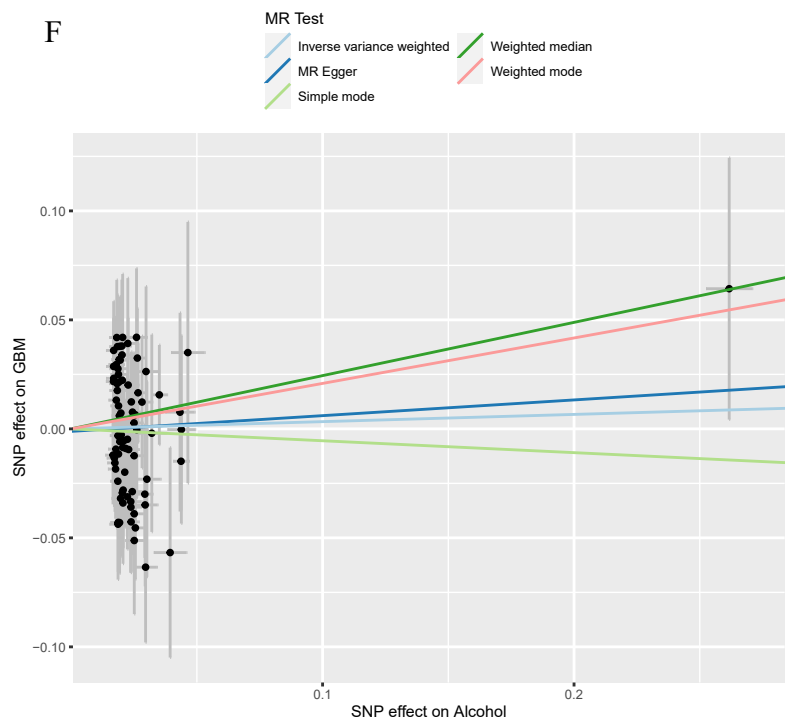

G

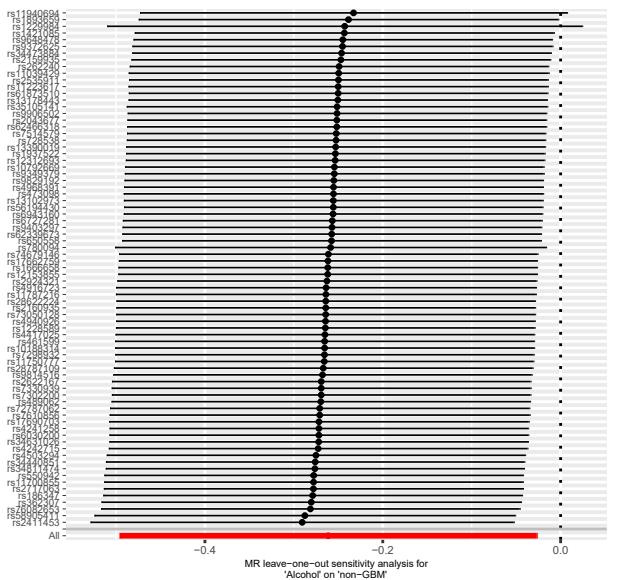

H

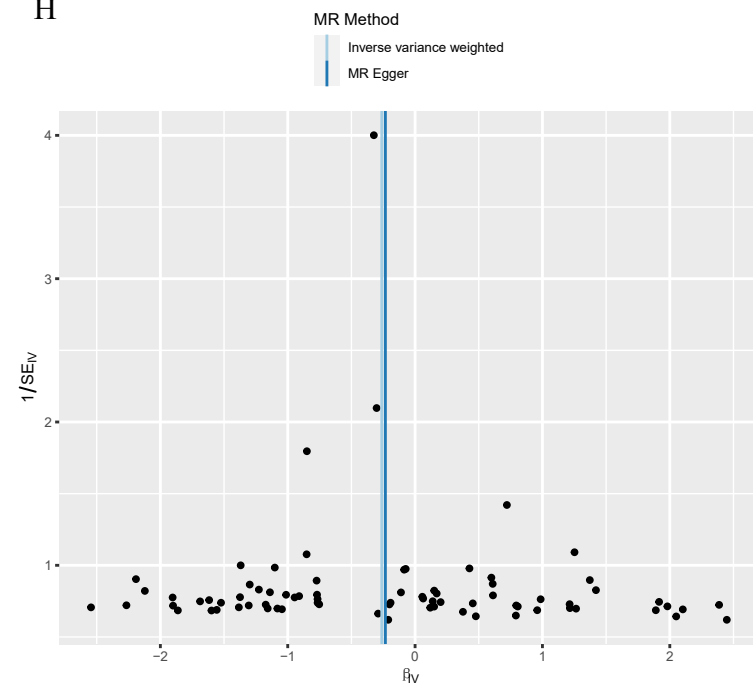

I

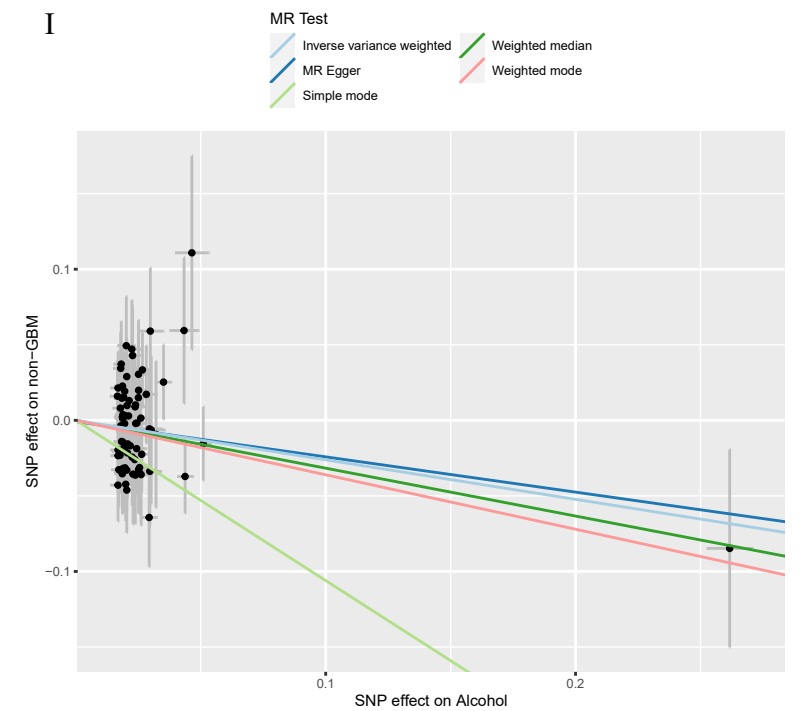

Supplement: Supplementary file 1 [file nutrients-17-00582-s001.zip › nutrients-3462880-supplementary/Sup_1.pdf]

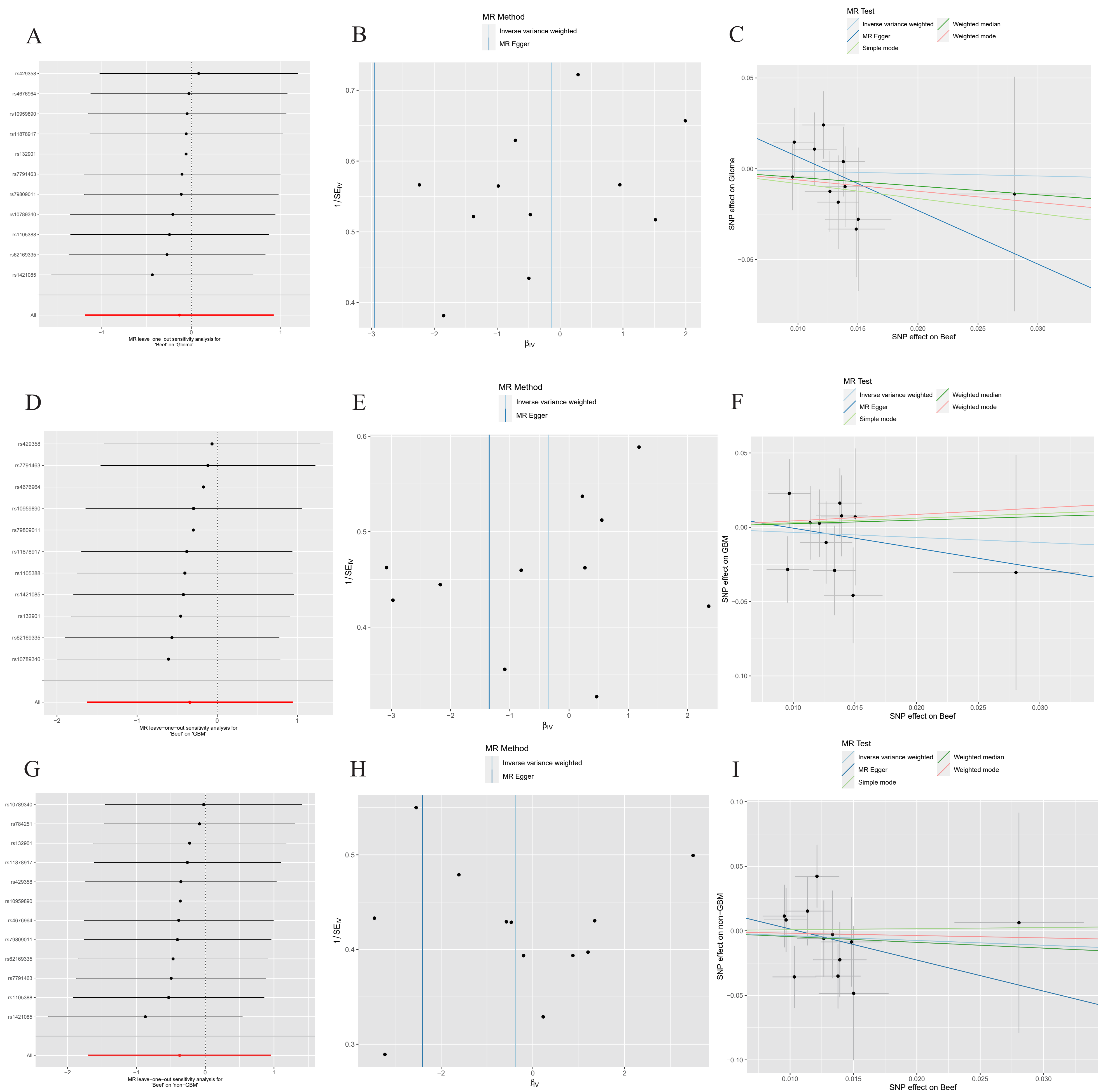

Supplement: Supplementary file 1 [file nutrients-17-00582-s001.zip › nutrients-3462880-supplementary/Sup_10.pdf]

A

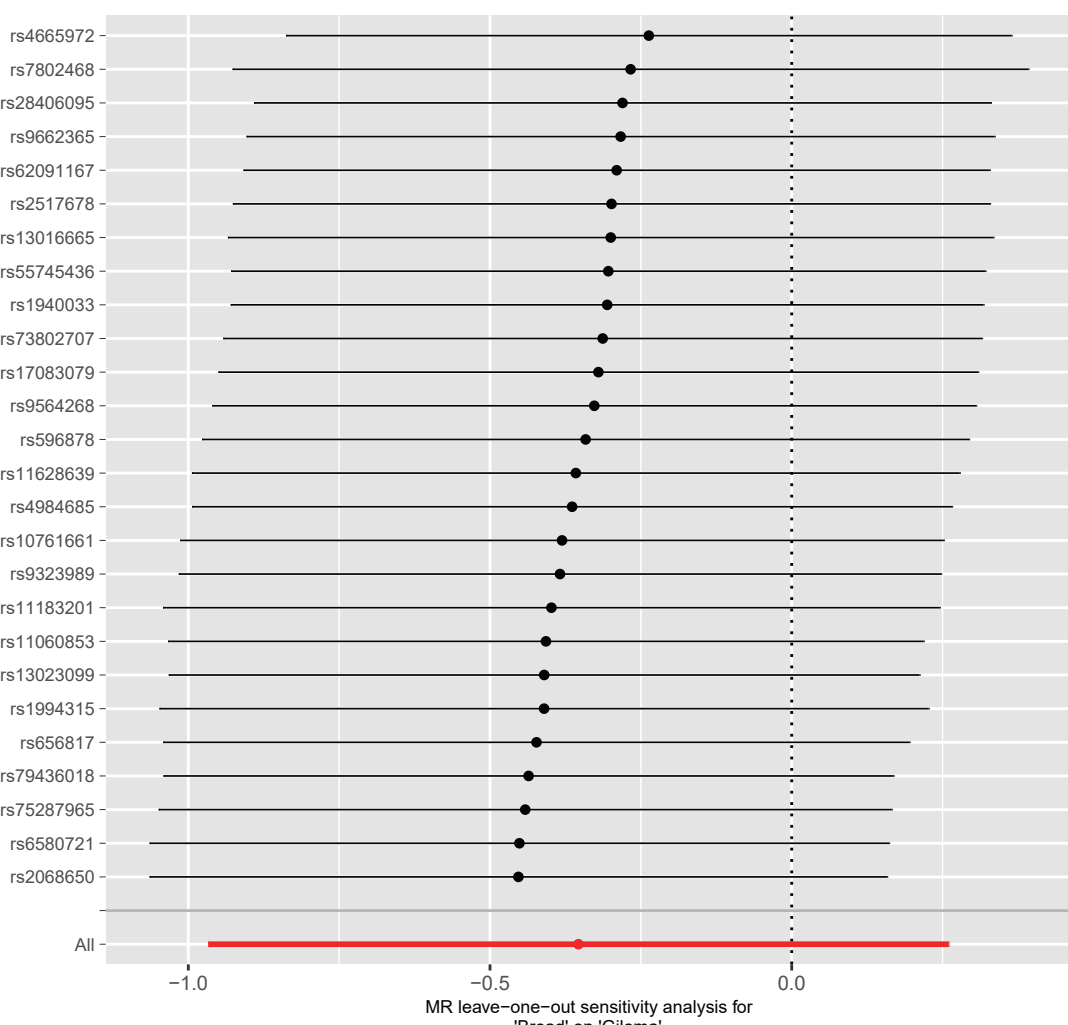

B

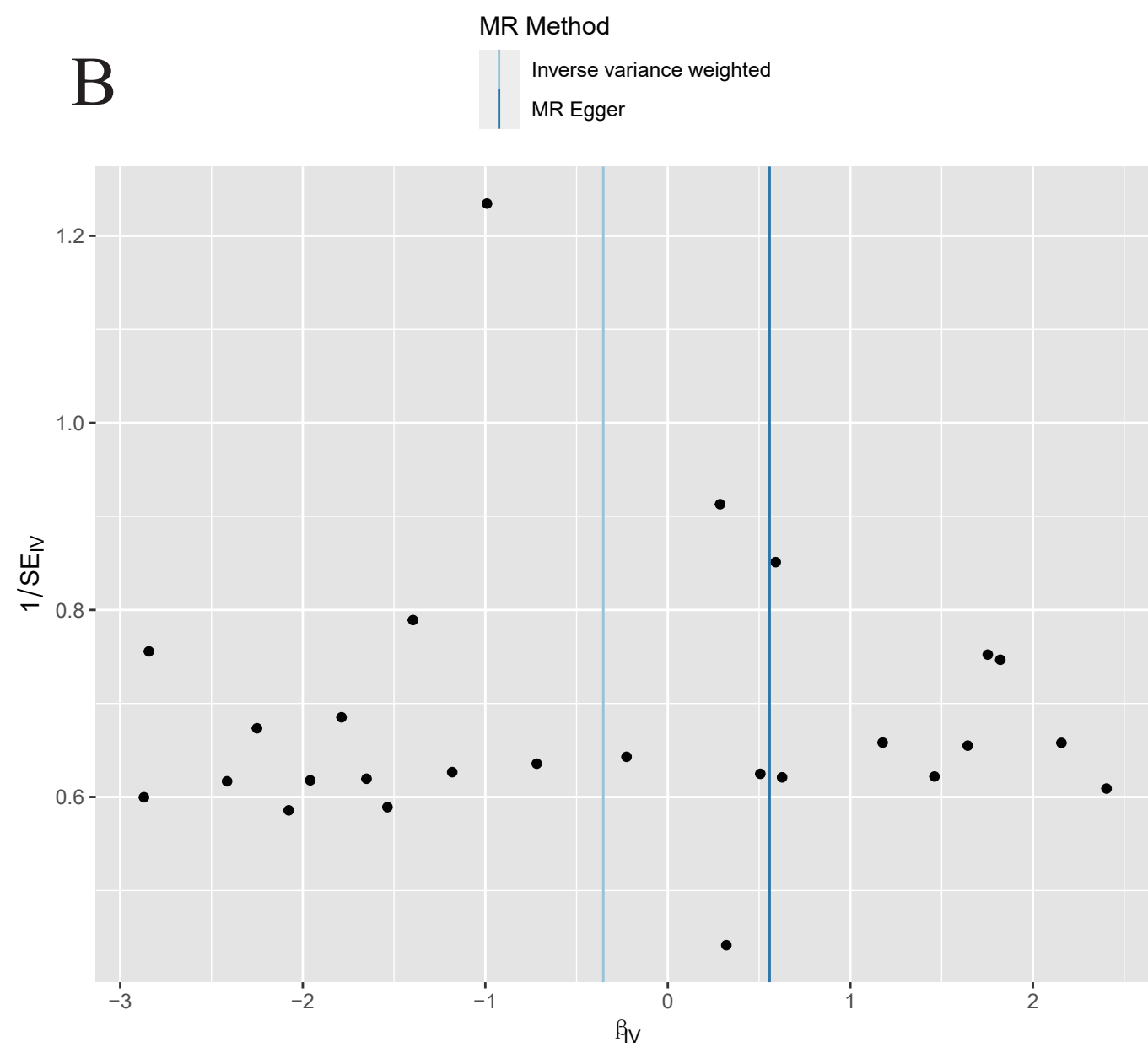

C

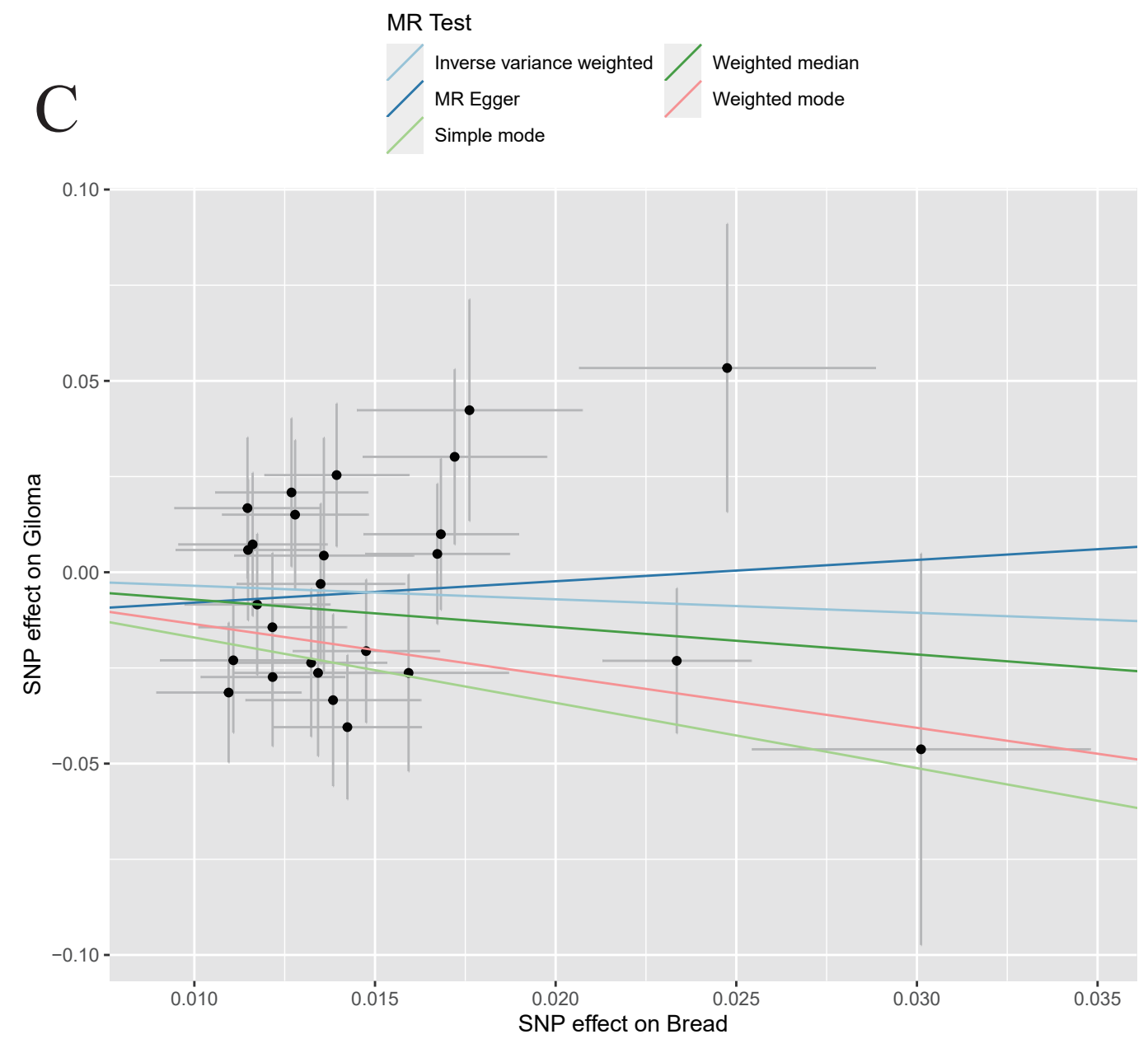

D

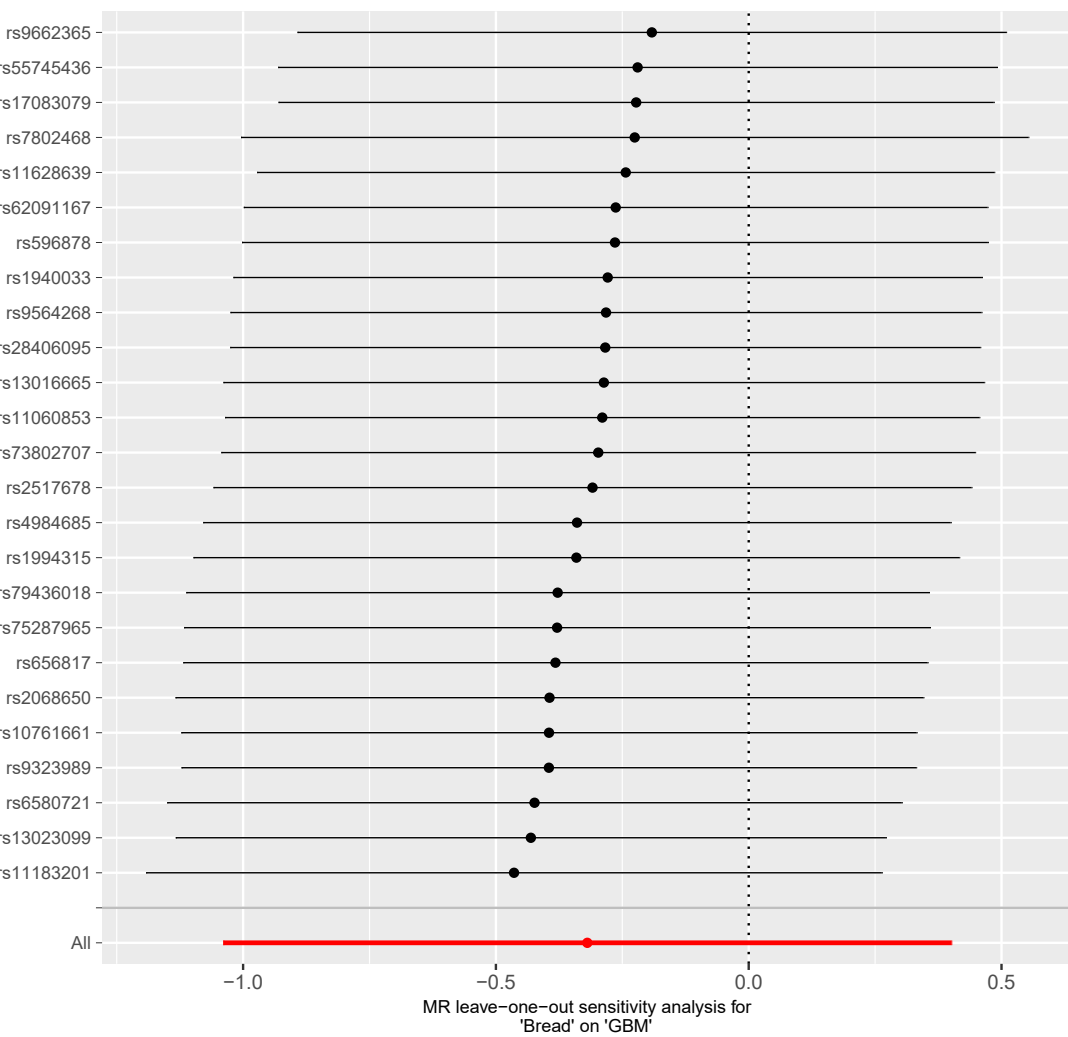

E

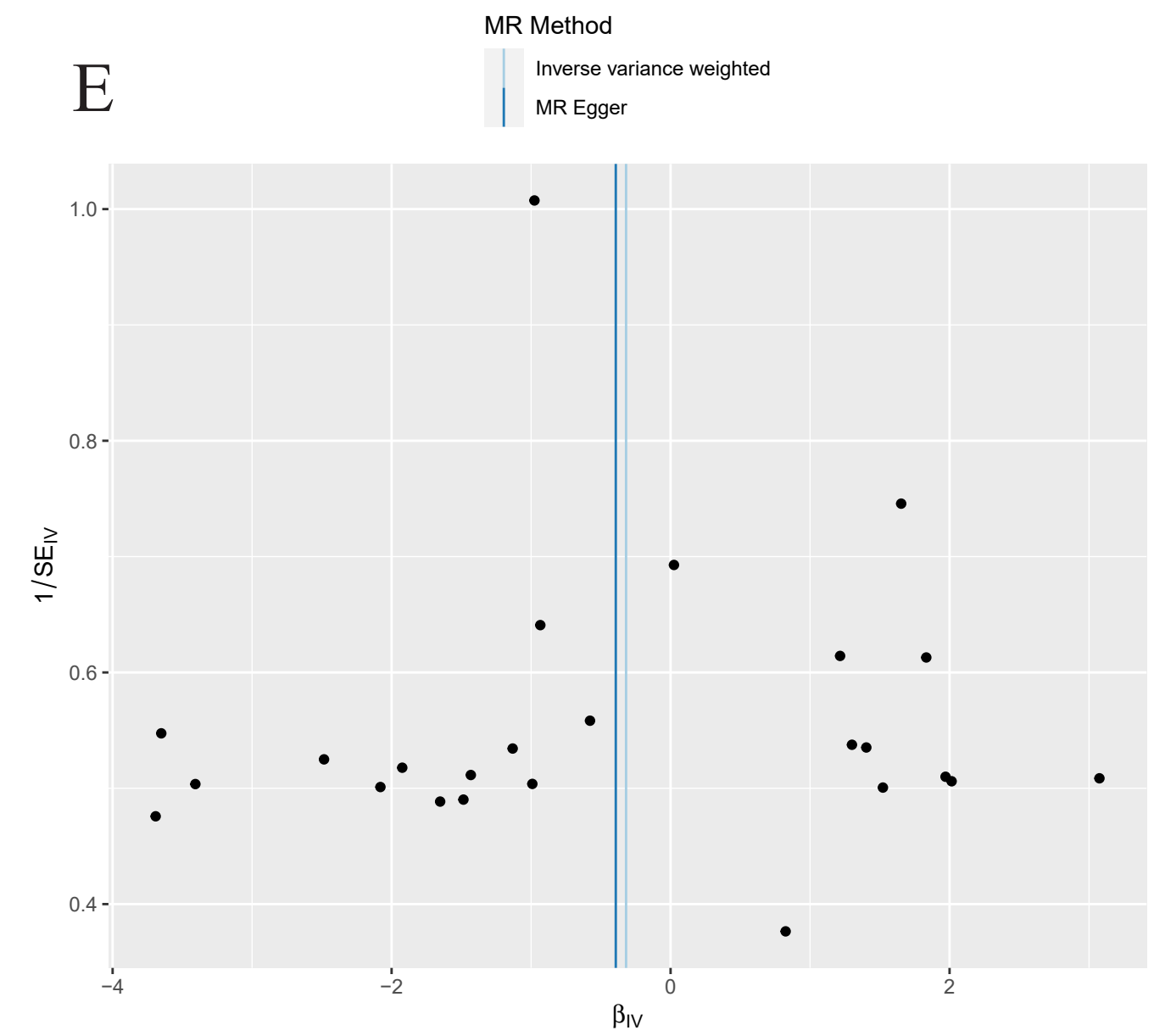

F

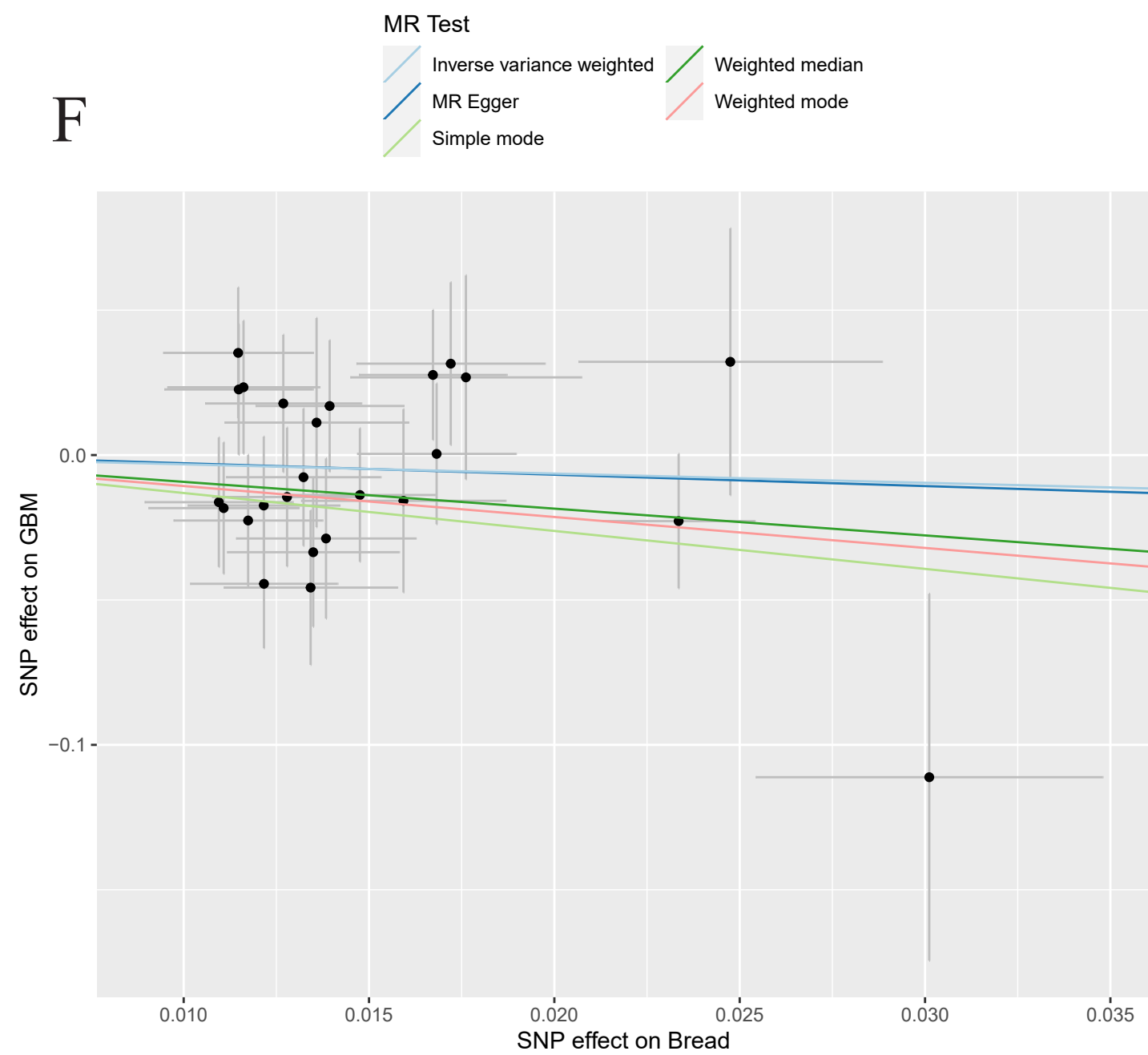

G

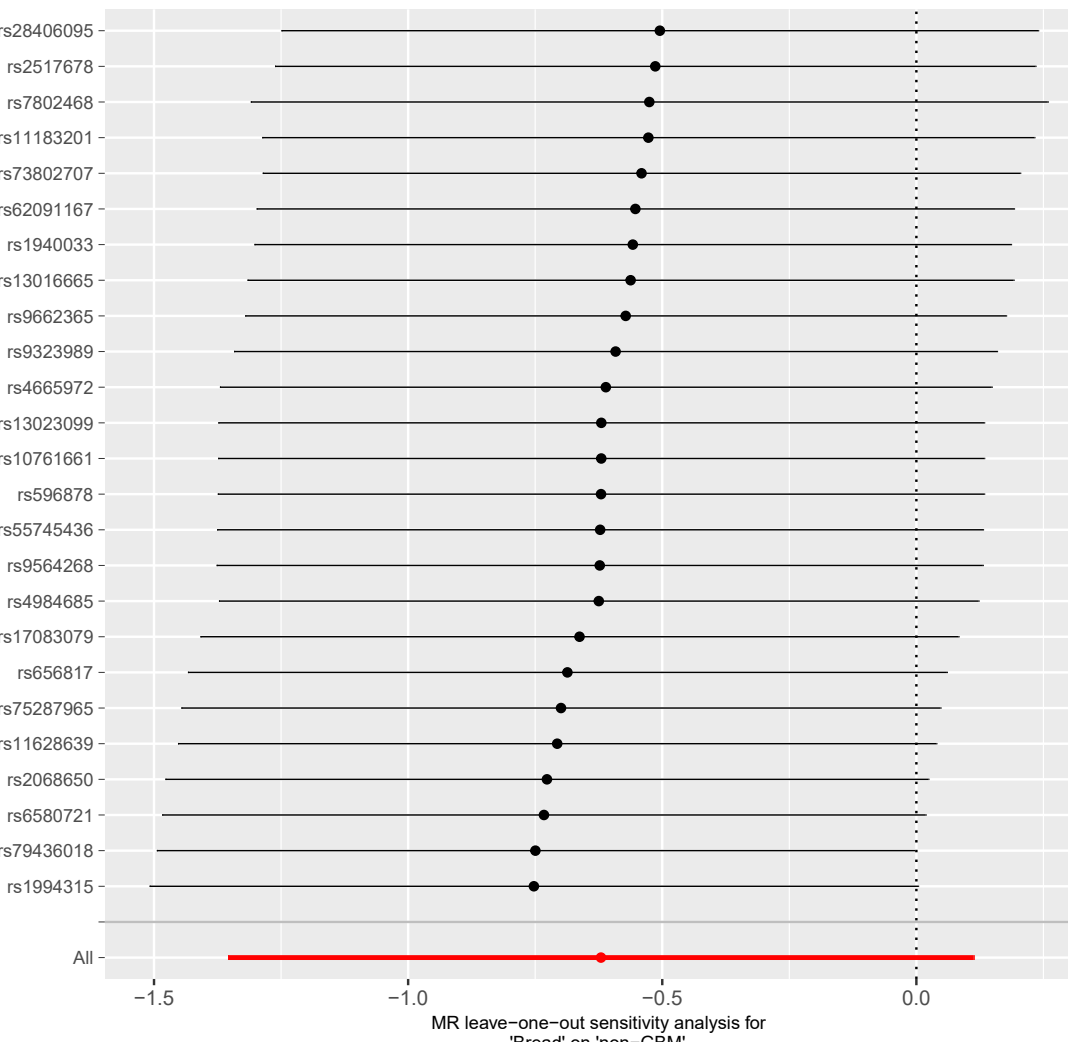

H

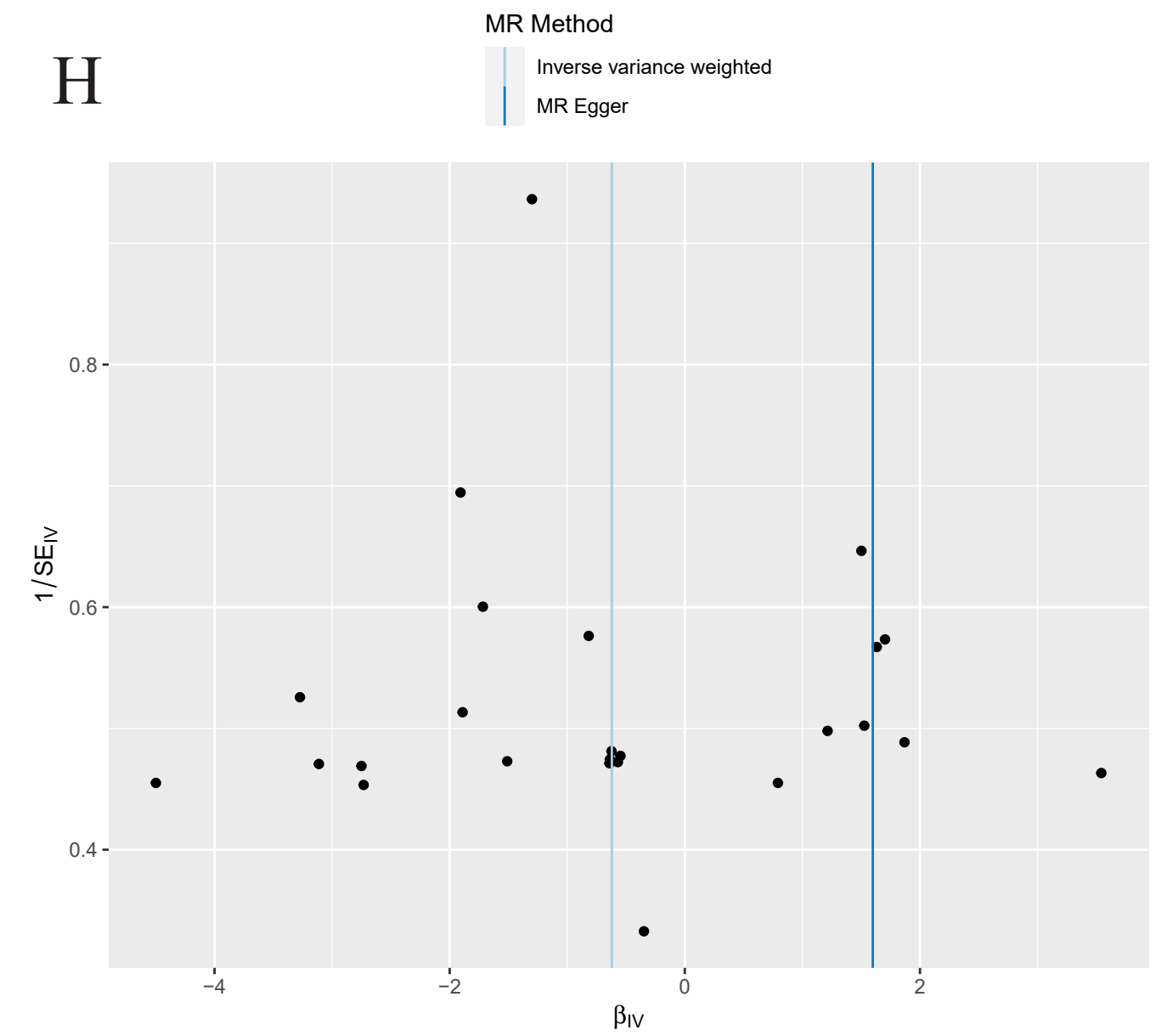

I

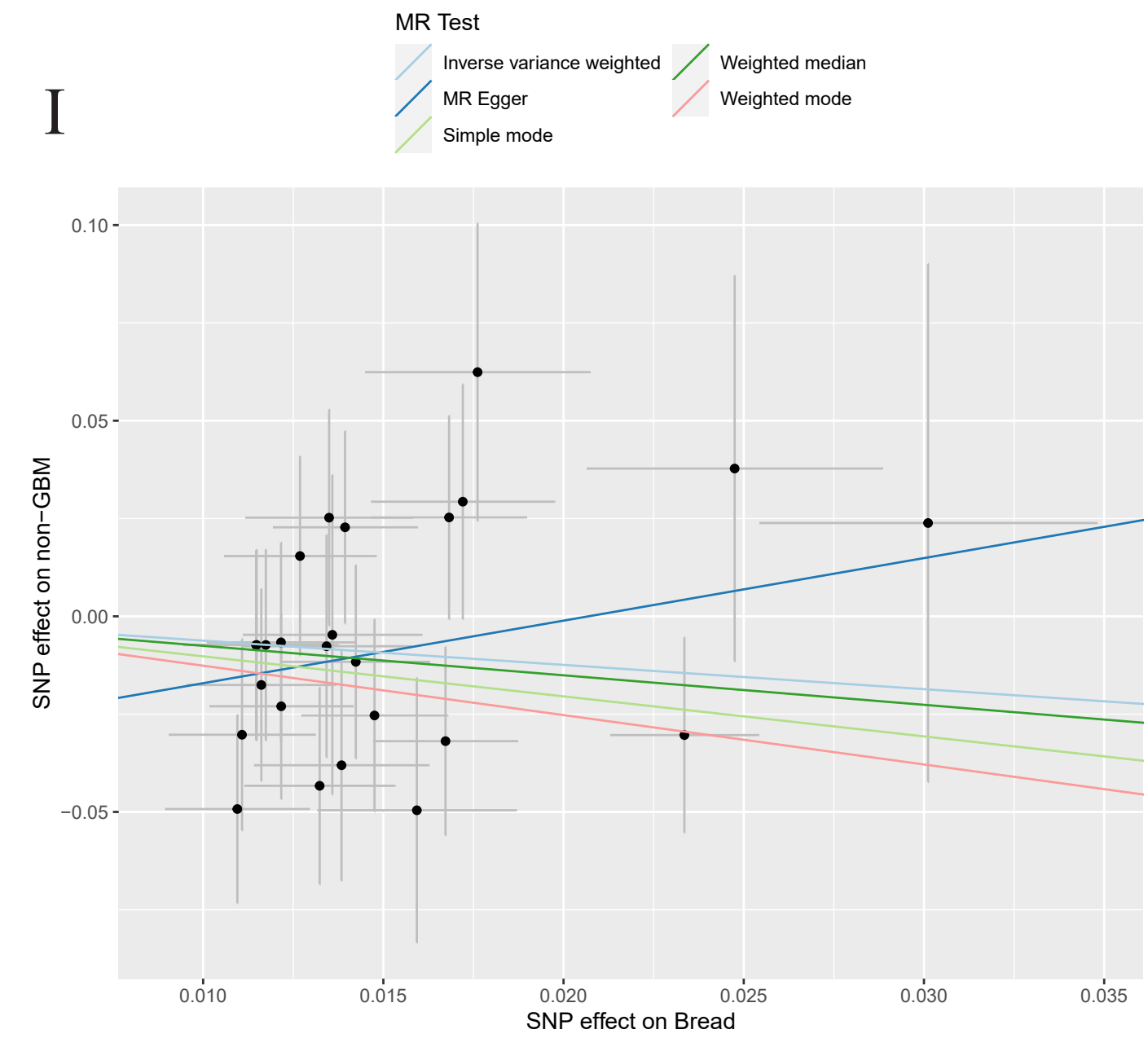

Supplement: Supplementary file 1 [file nutrients-17-00582-s001.zip › nutrients-3462880-supplementary/Sup_11.pdf]

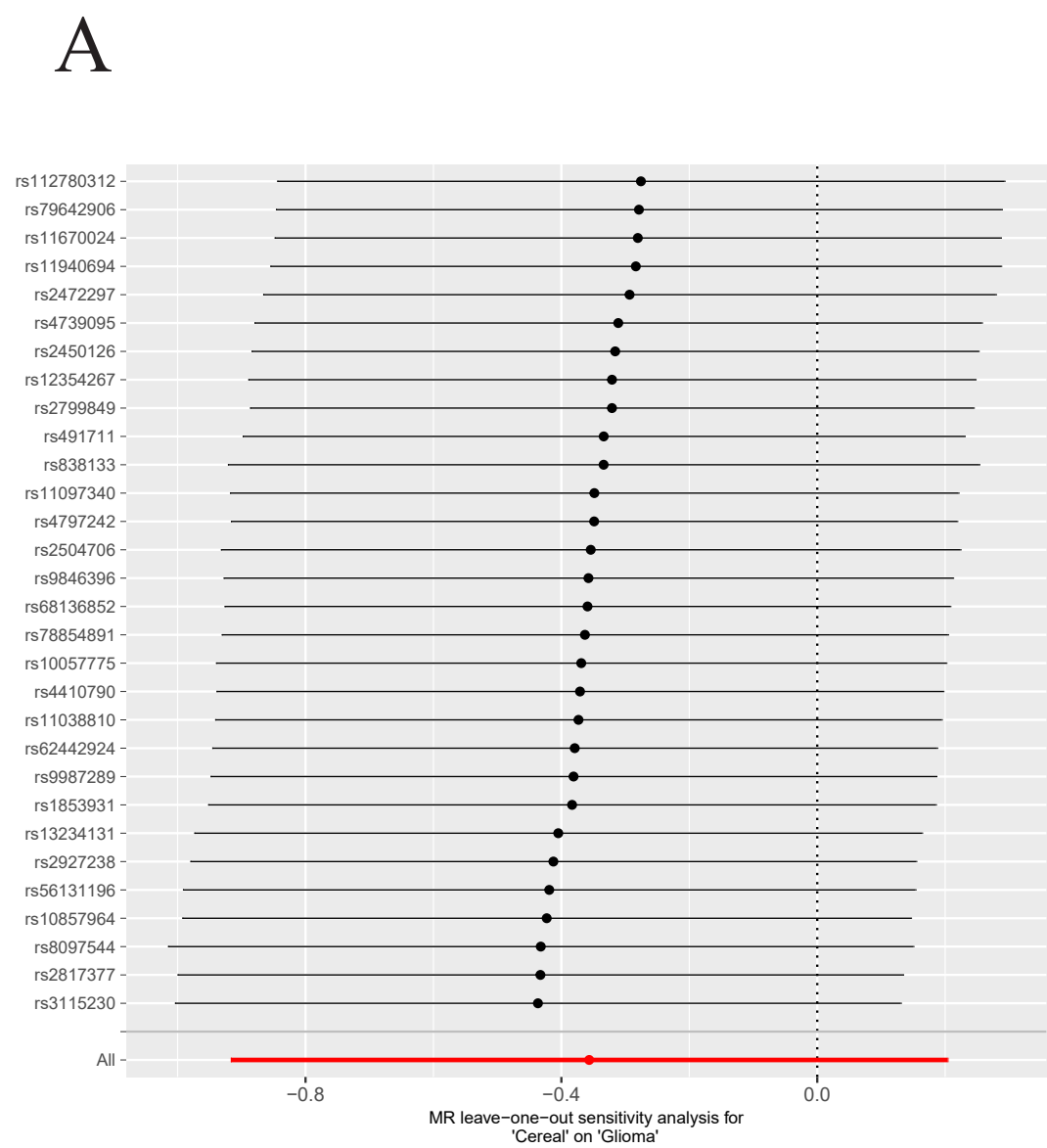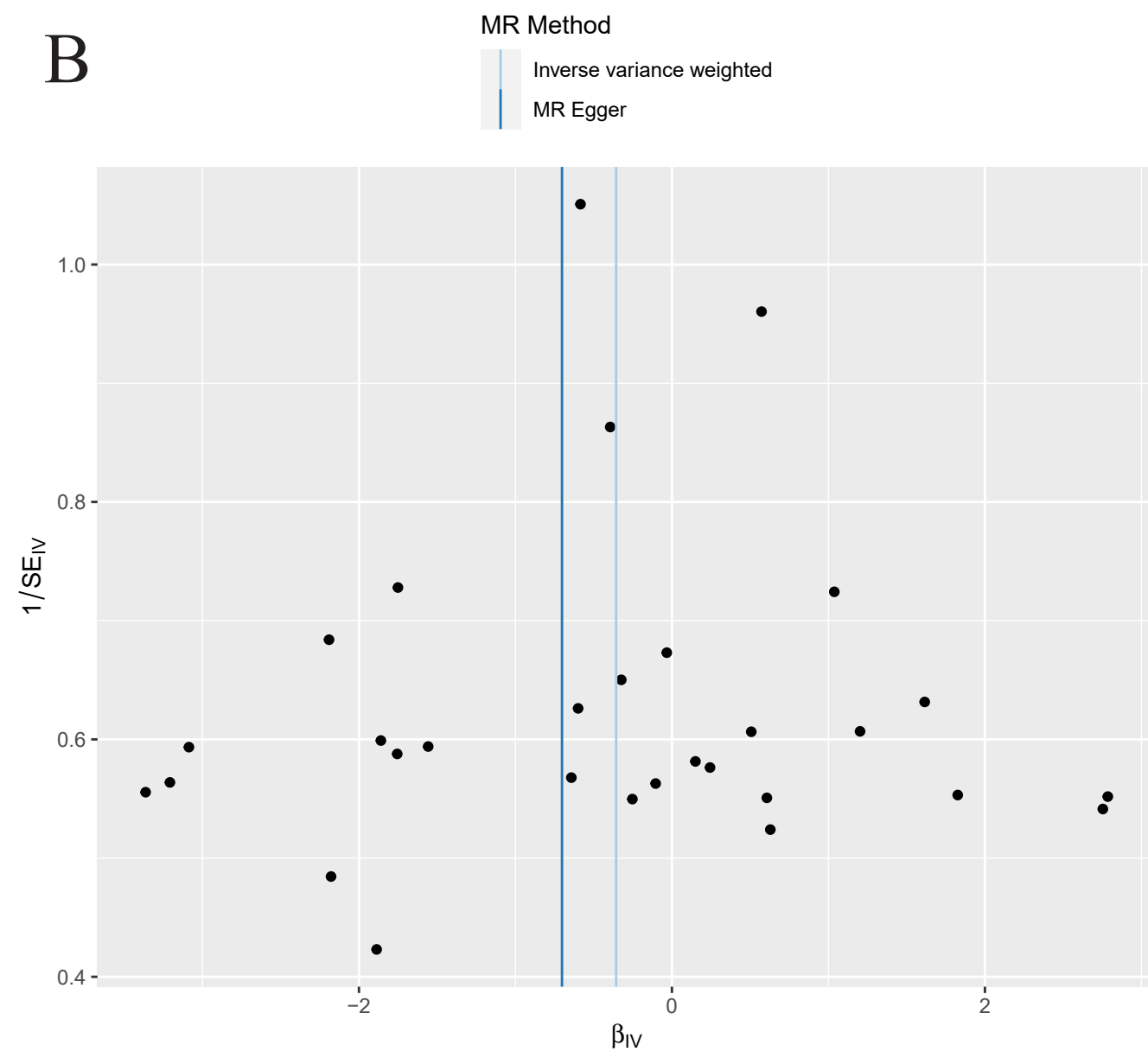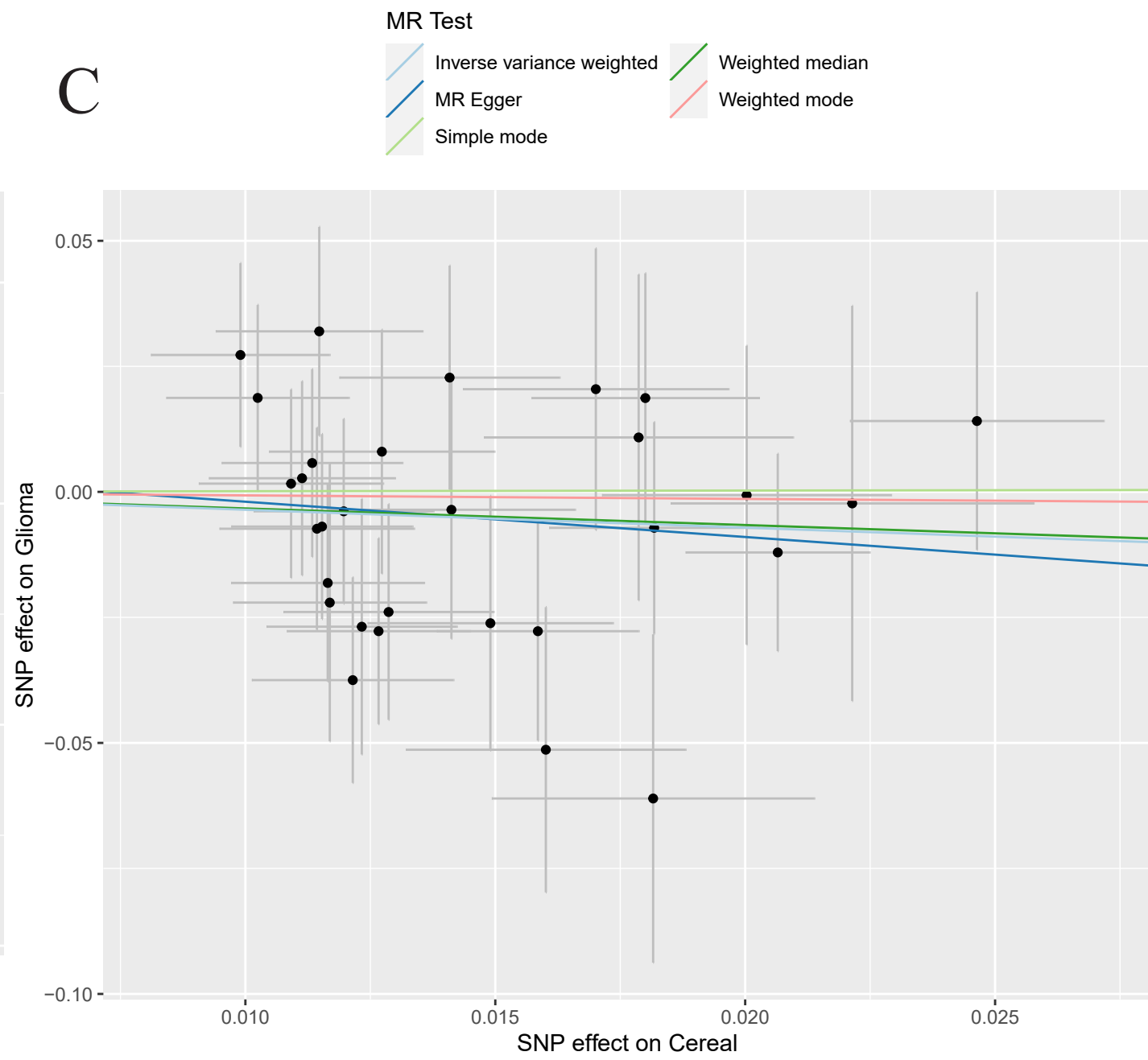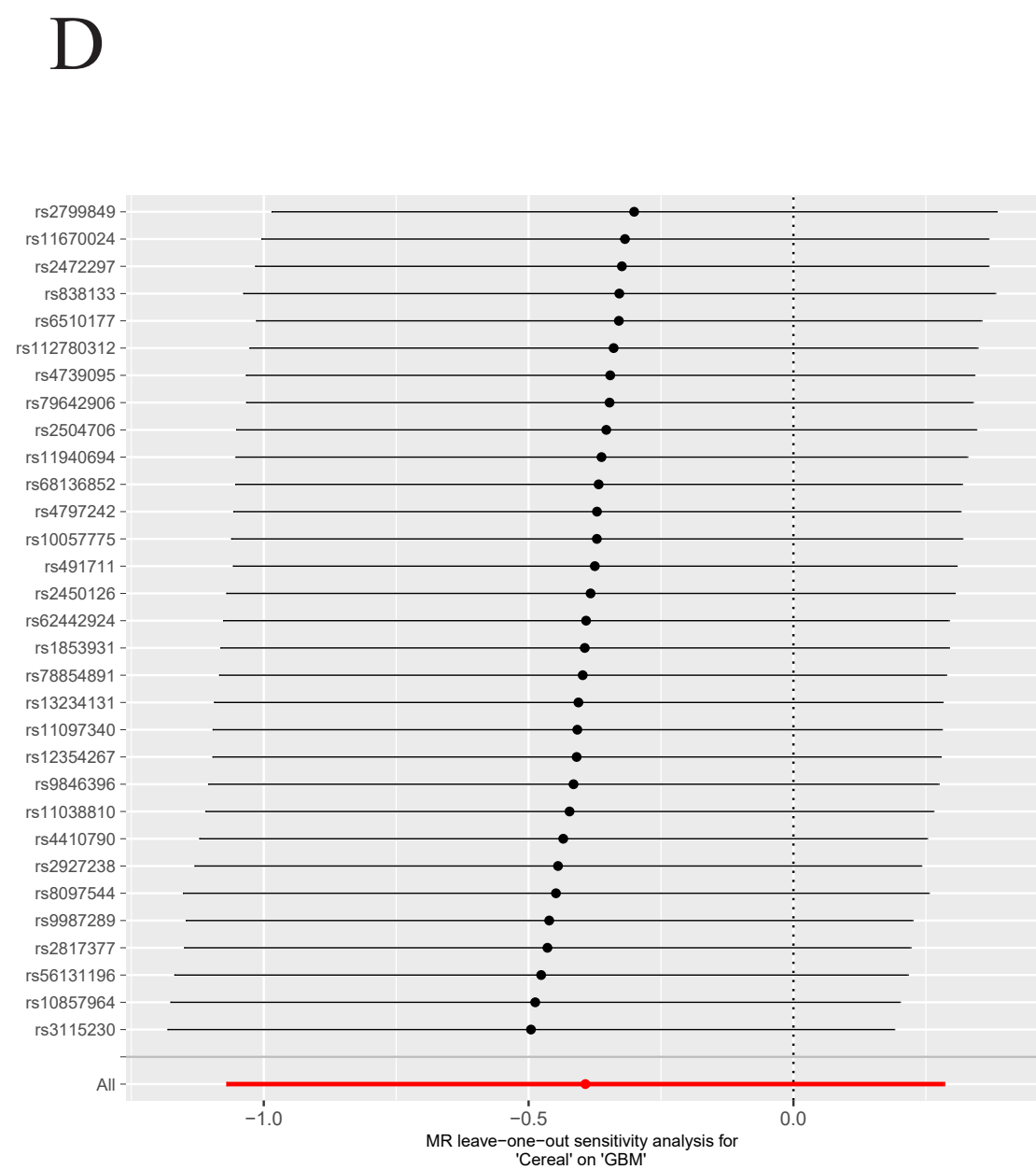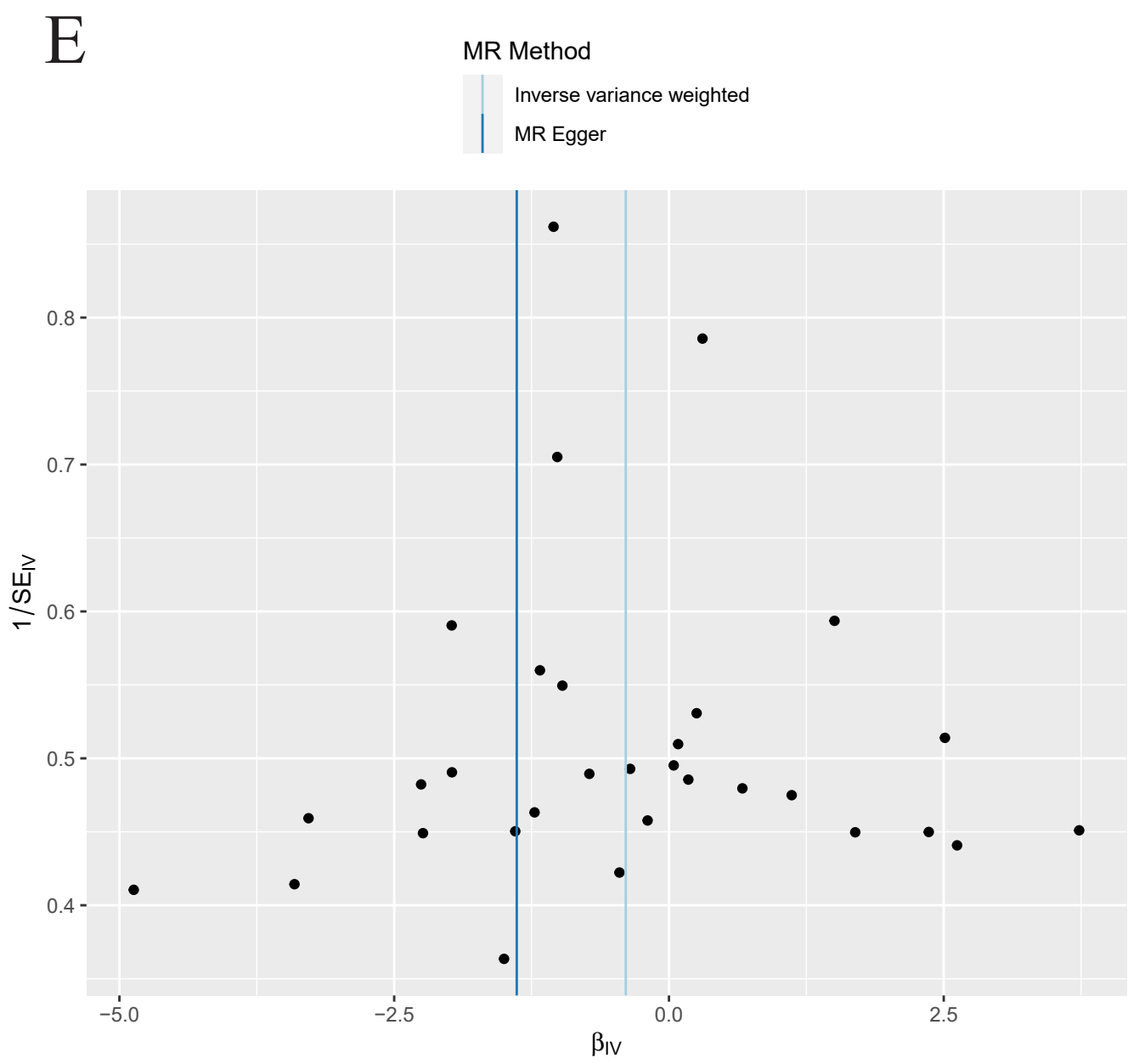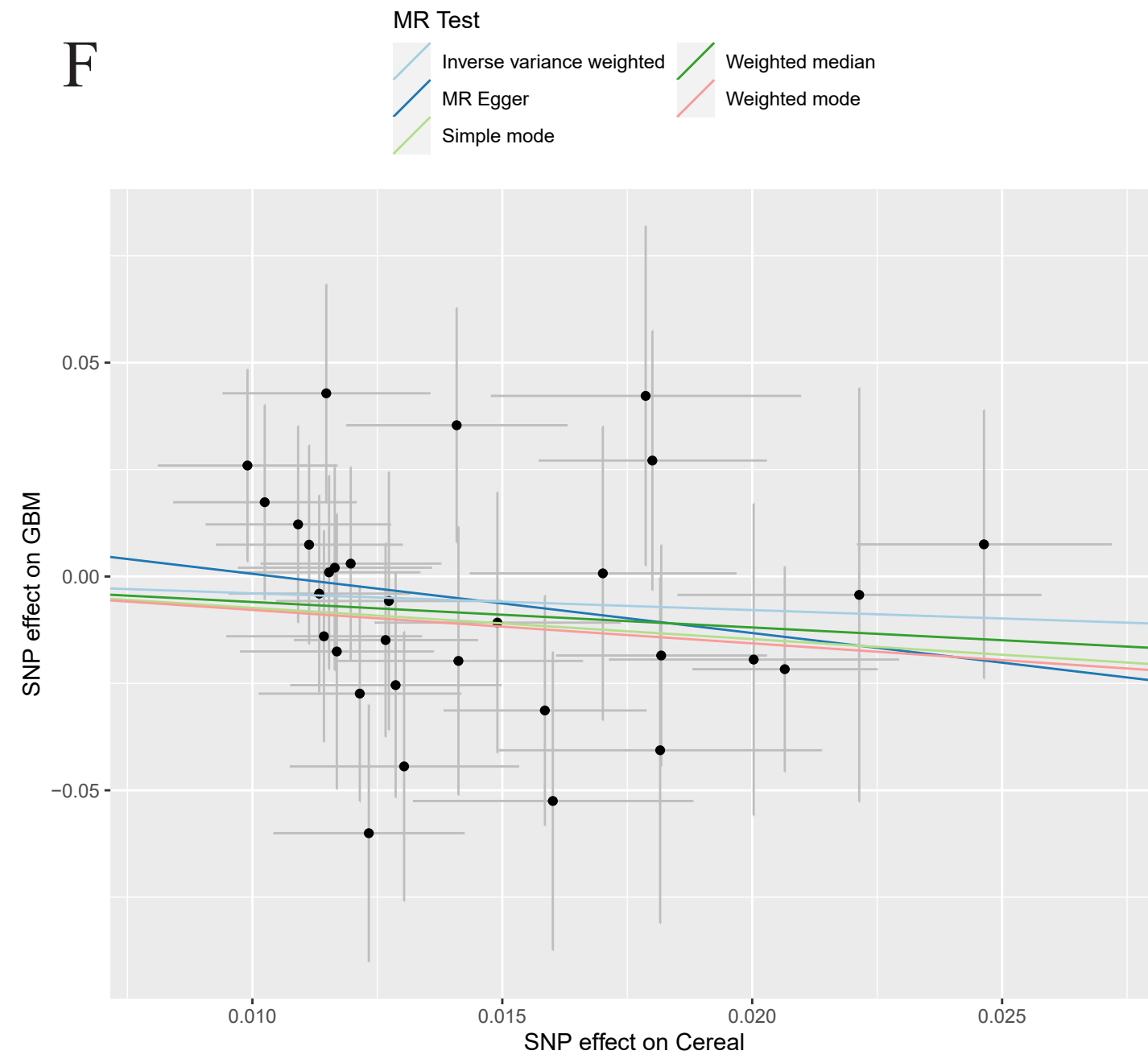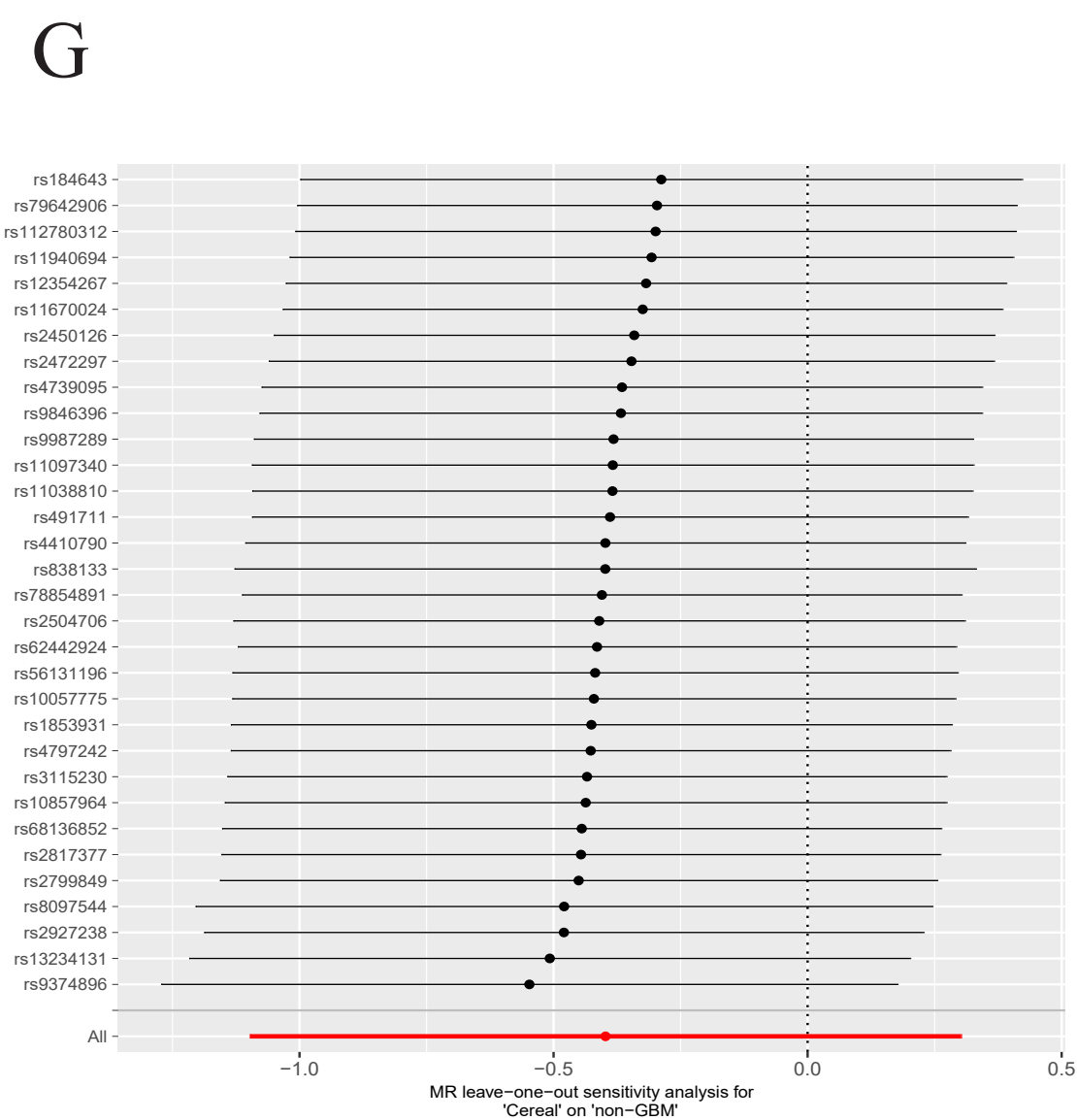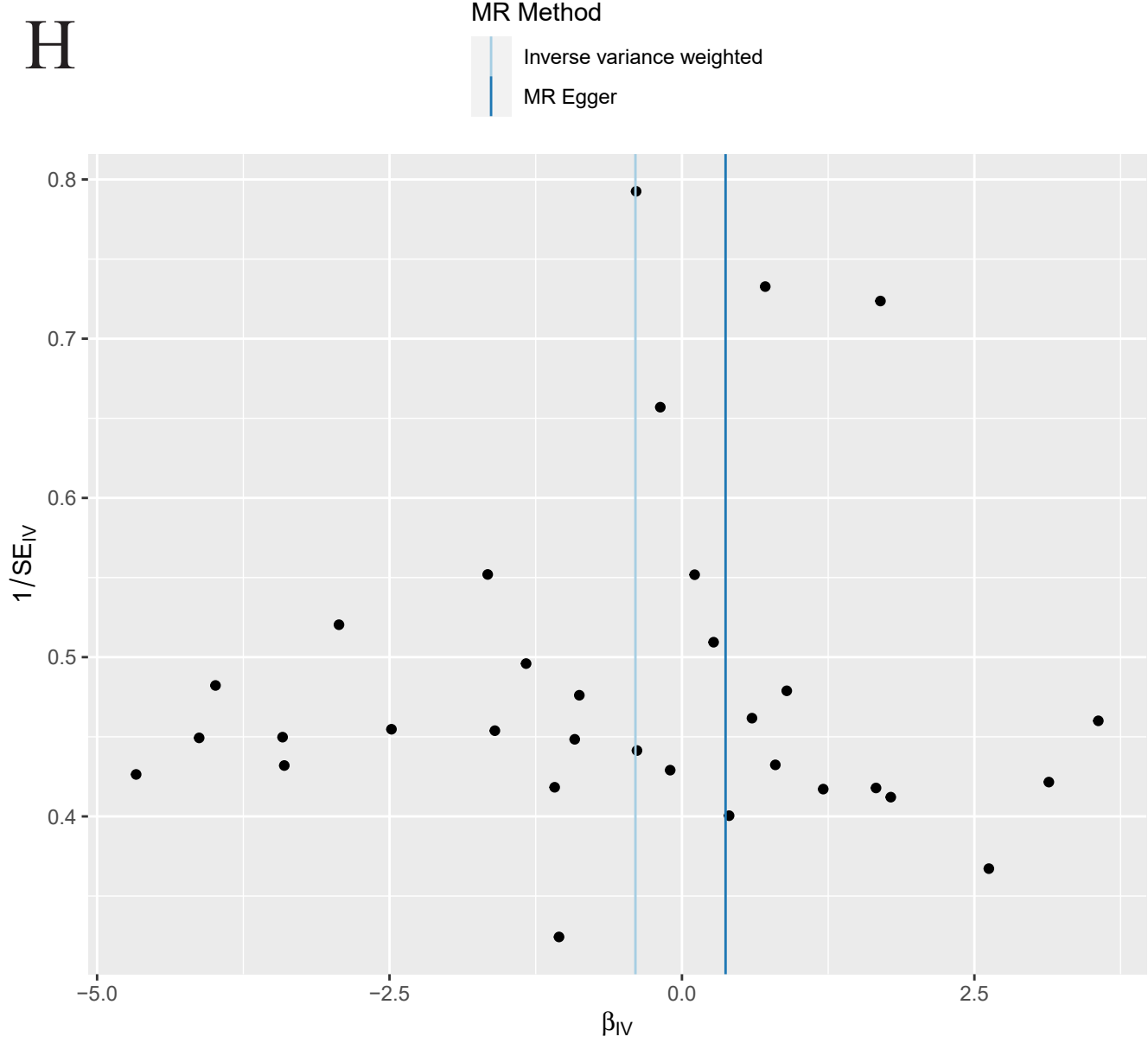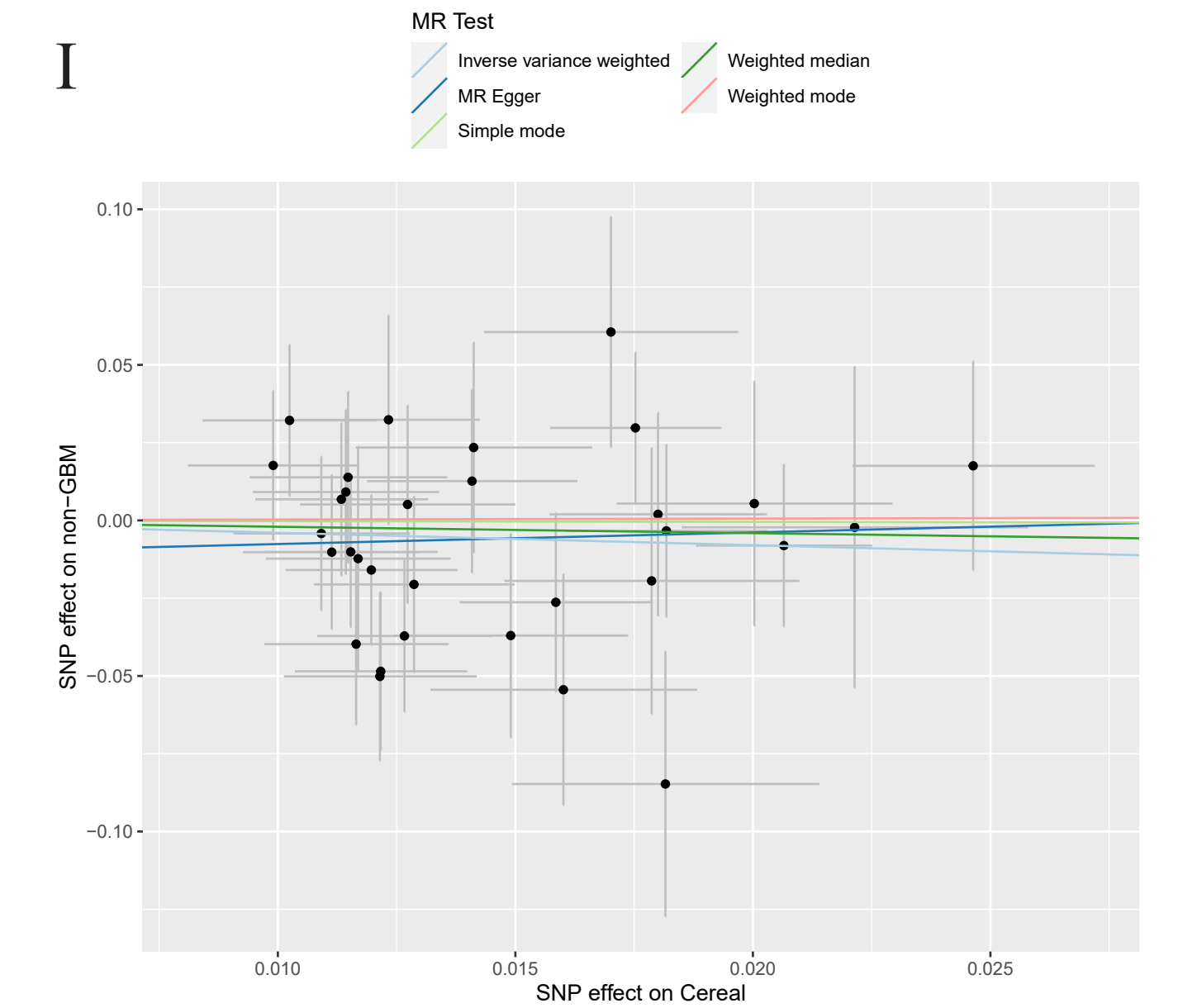

Supplement: Supplementary file 1 [file nutrients-17-00582-s001.zip › nutrients-3462880-supplementary/Sup_12.pdf]

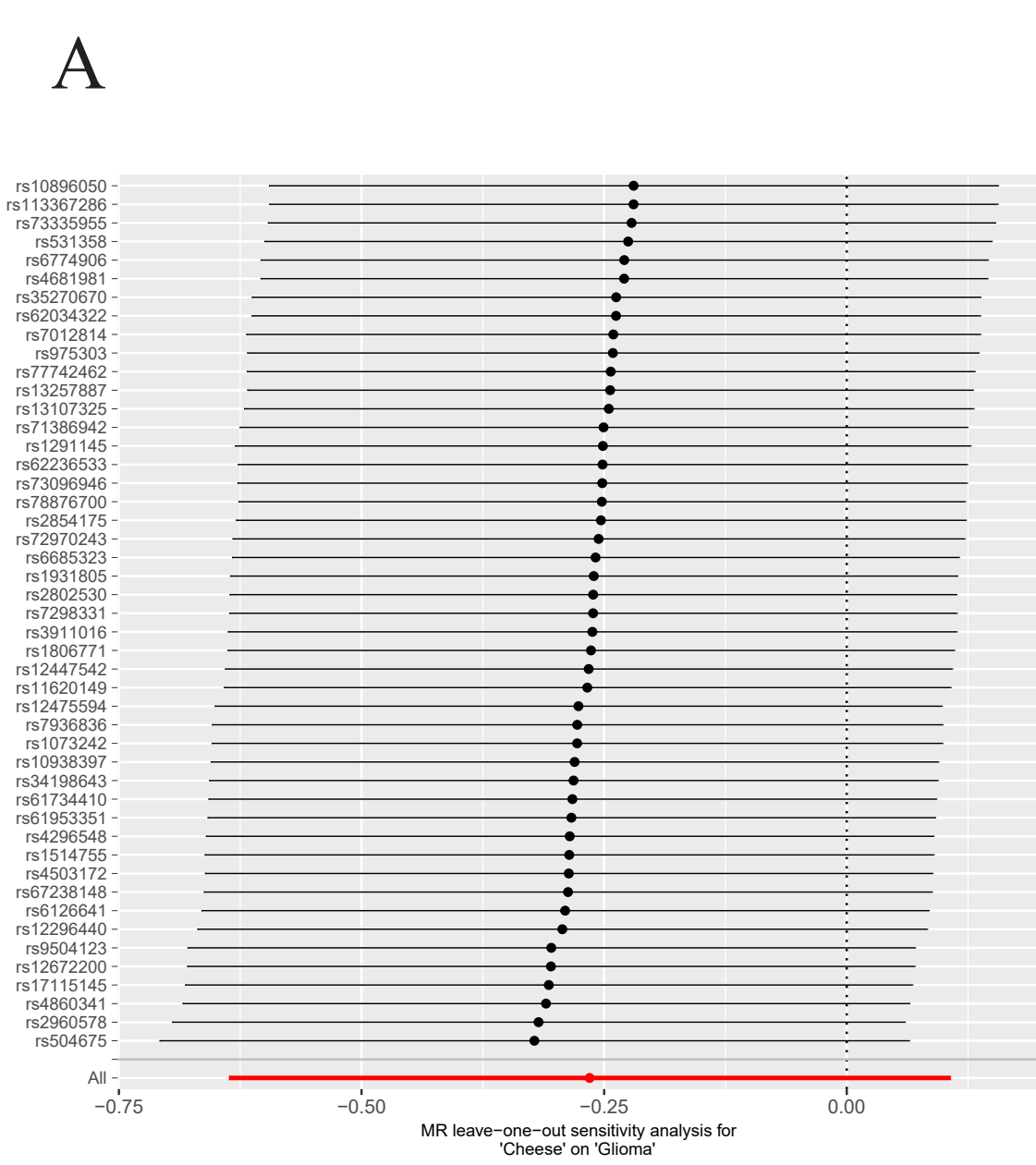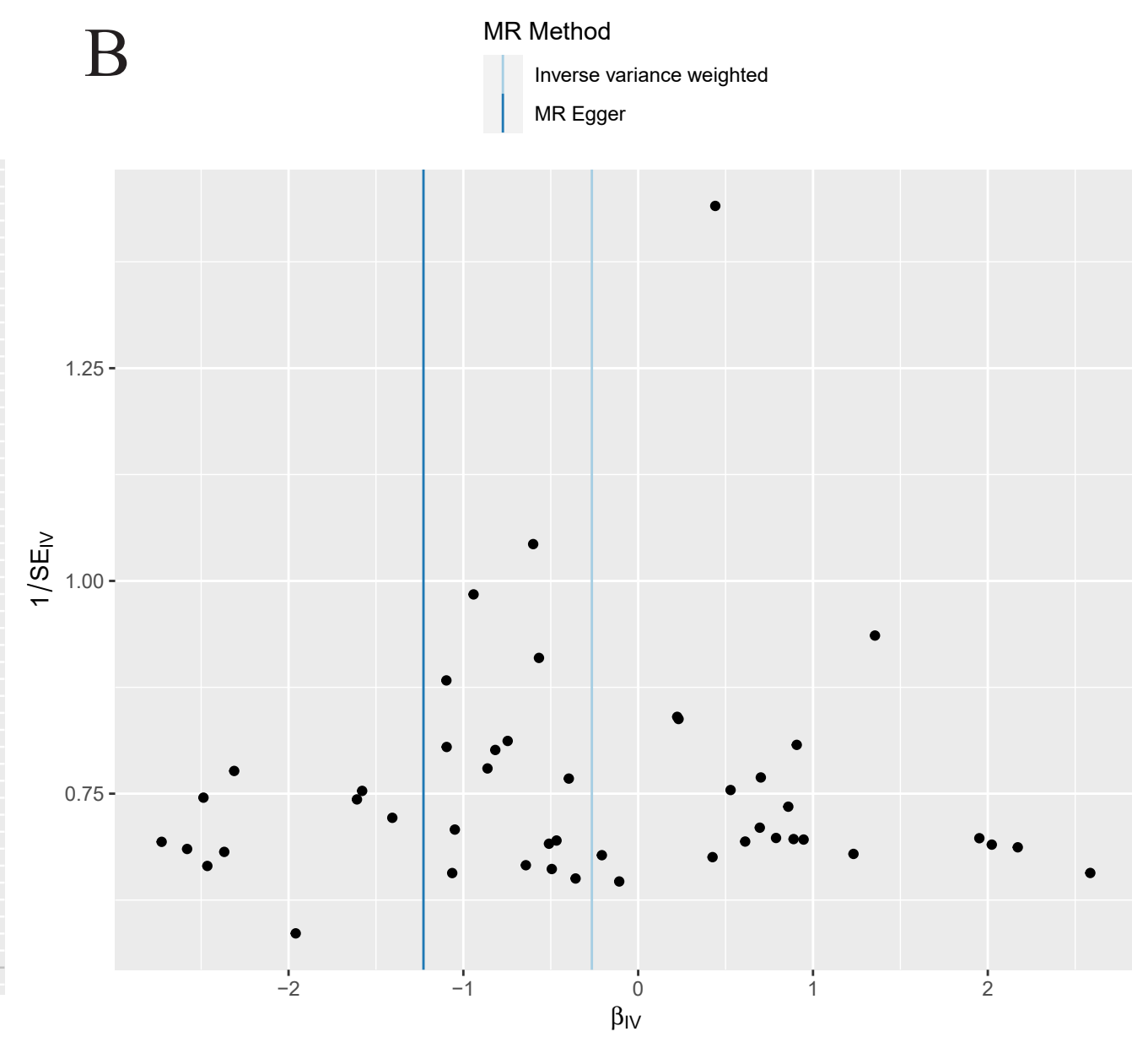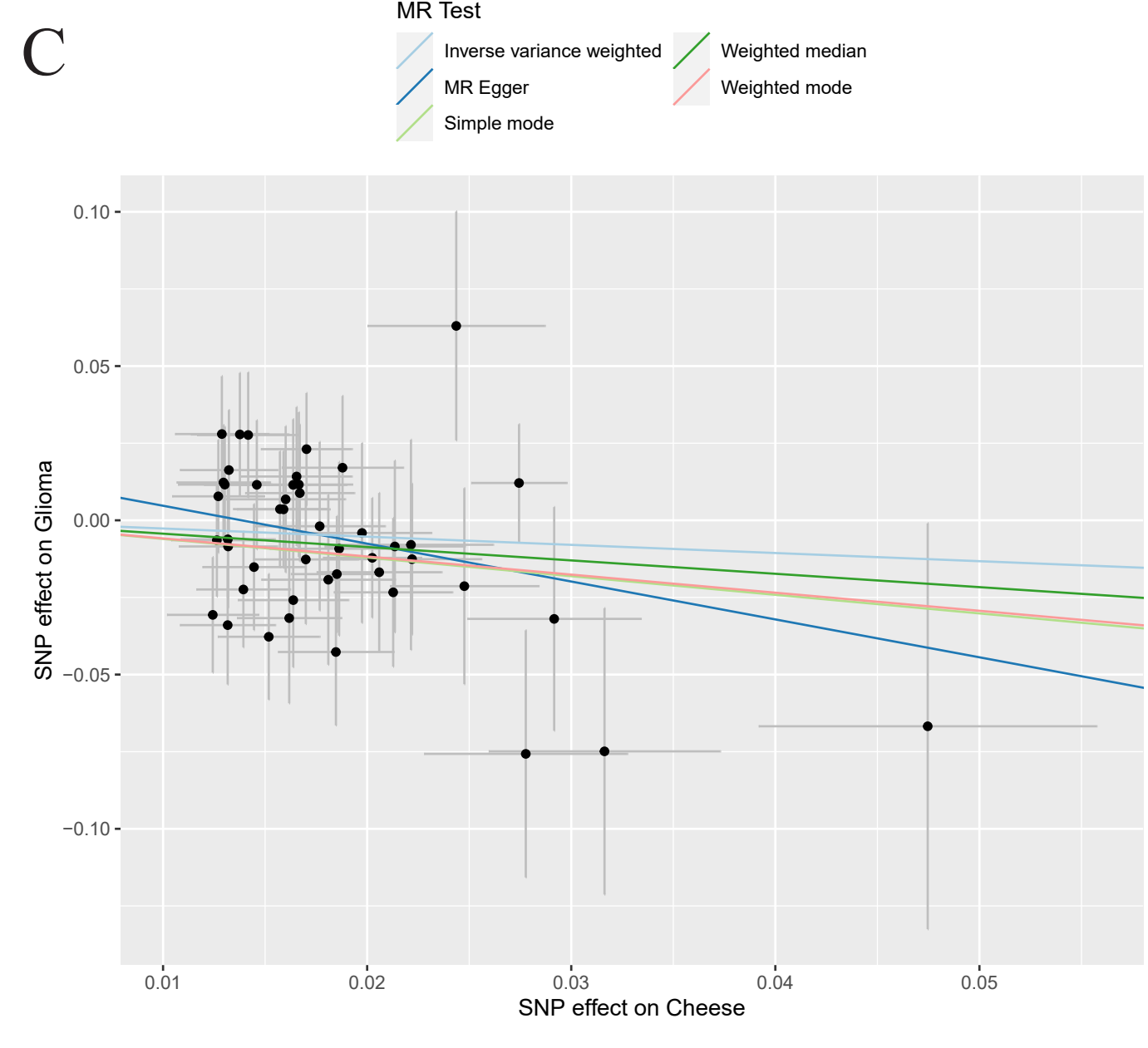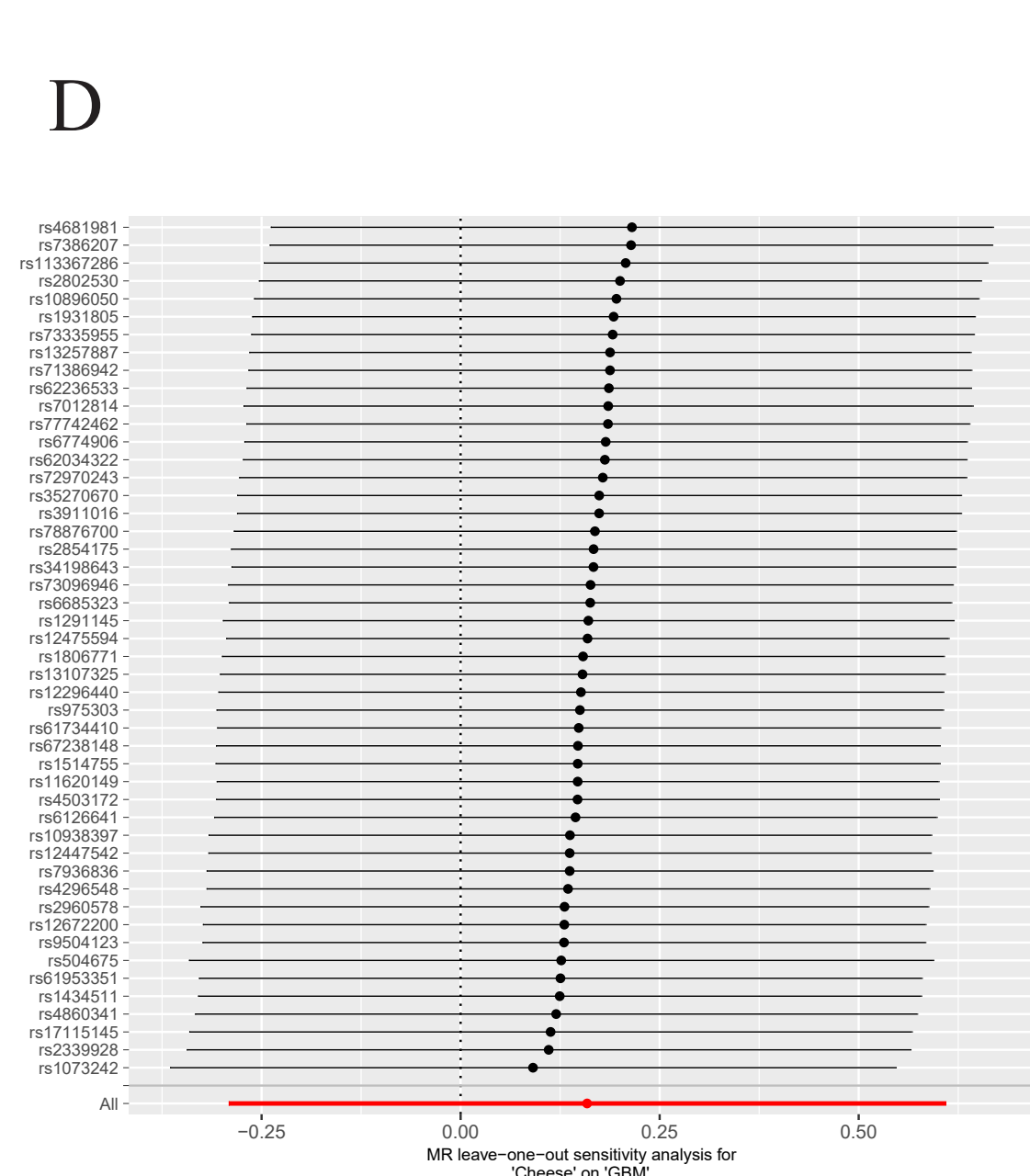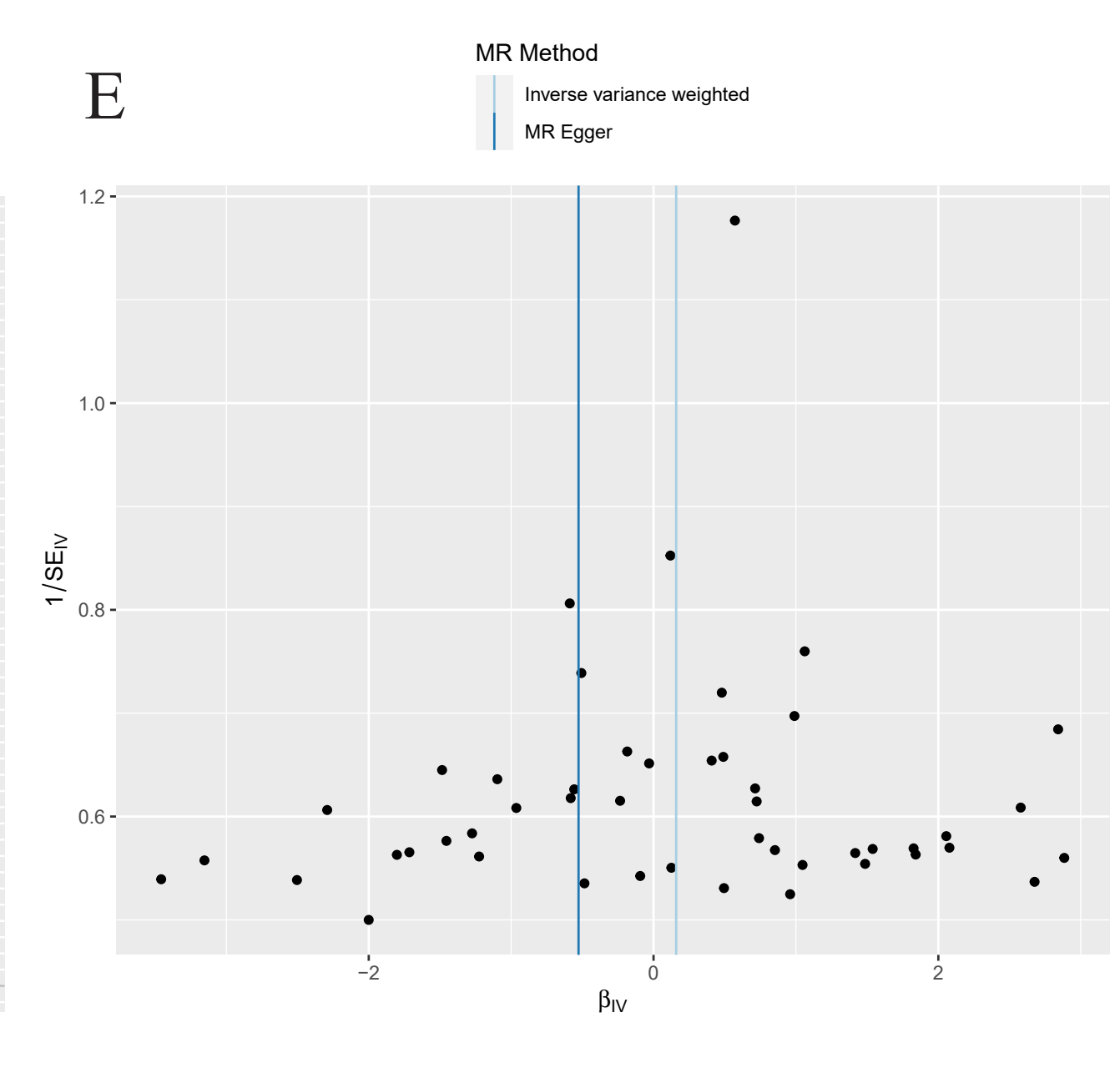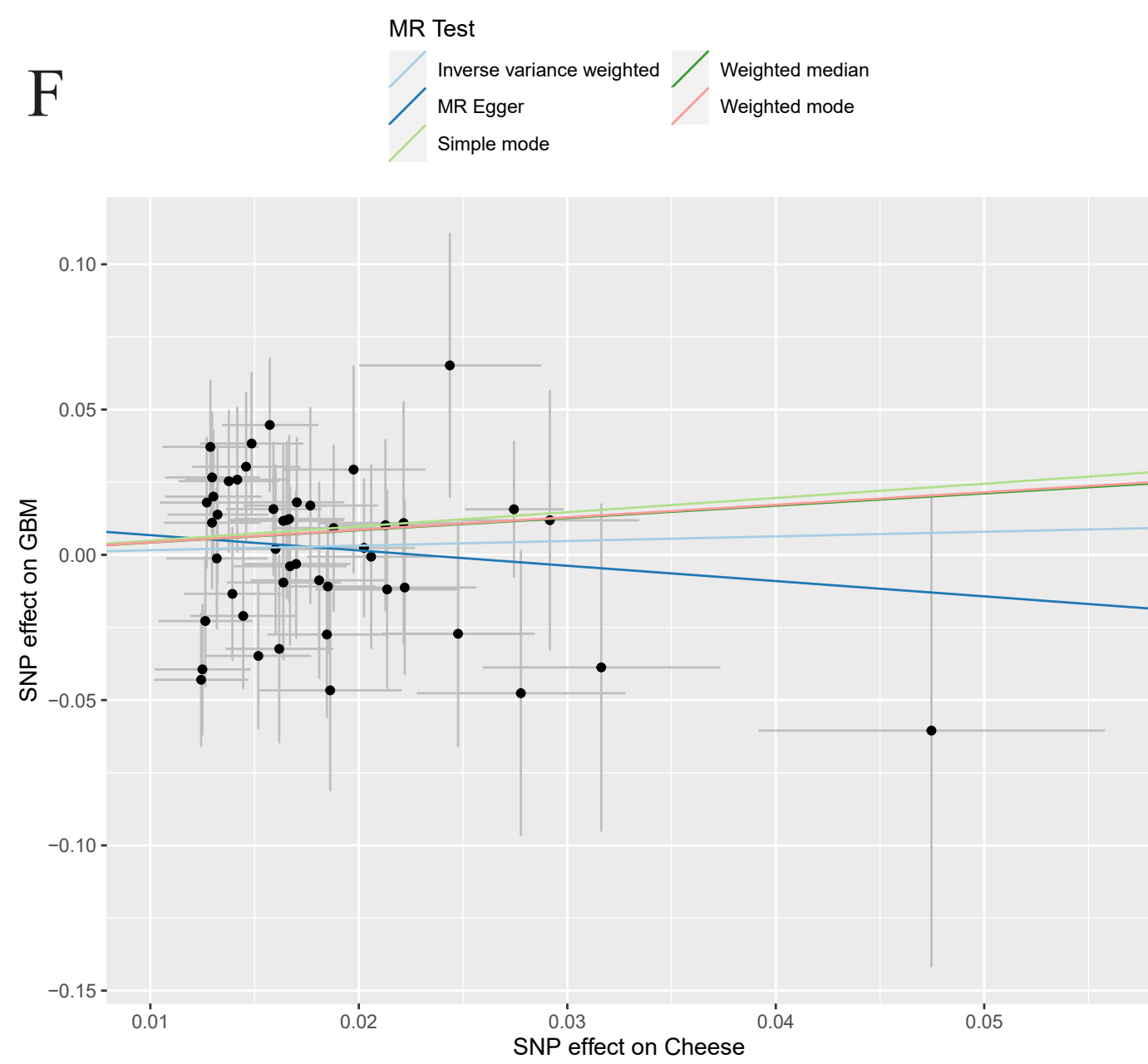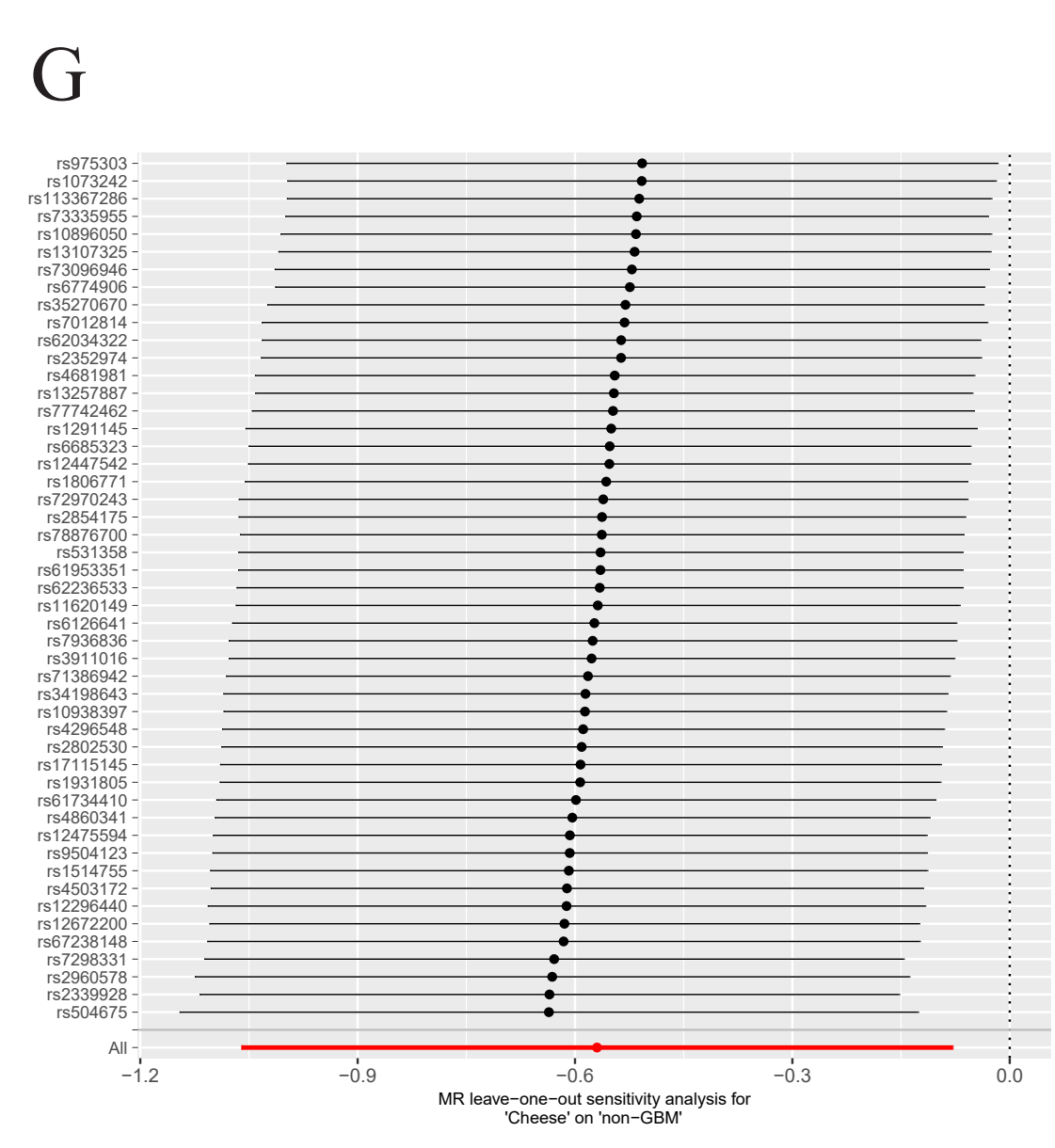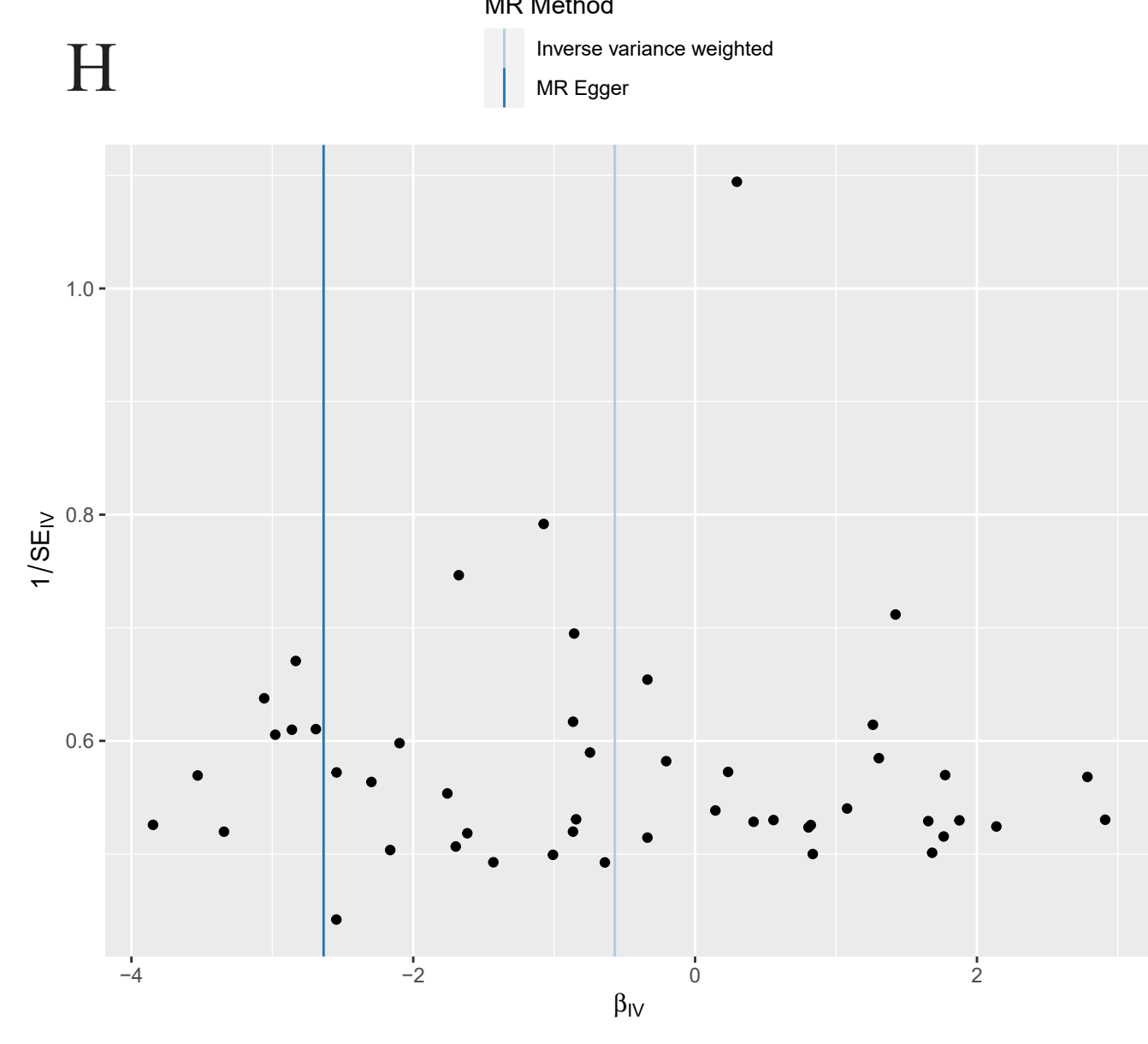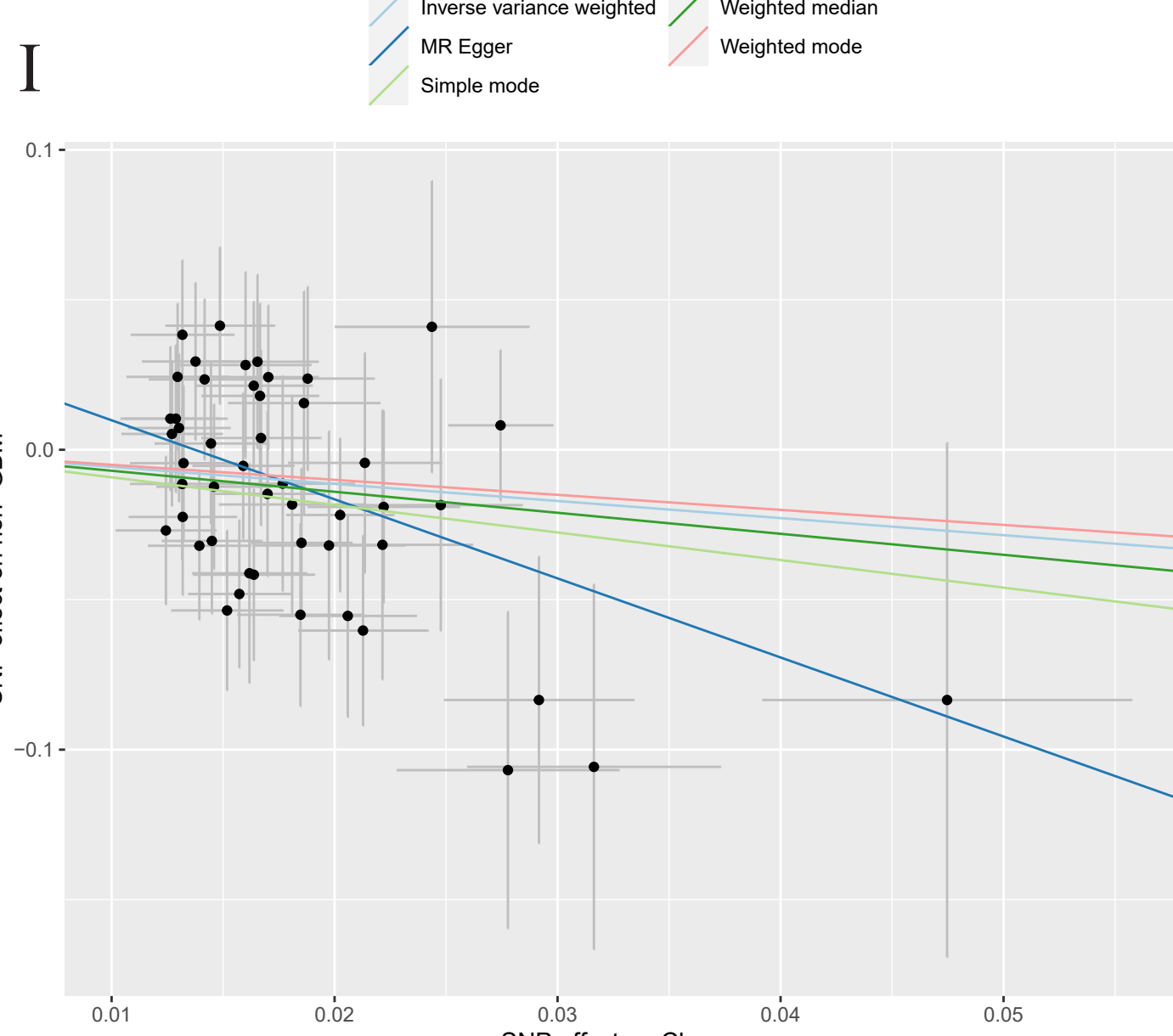

Supplement: Supplementary file 1 [file nutrients-17-00582-s001.zip › nutrients-3462880-supplementary/Sup_13.pdf]

A

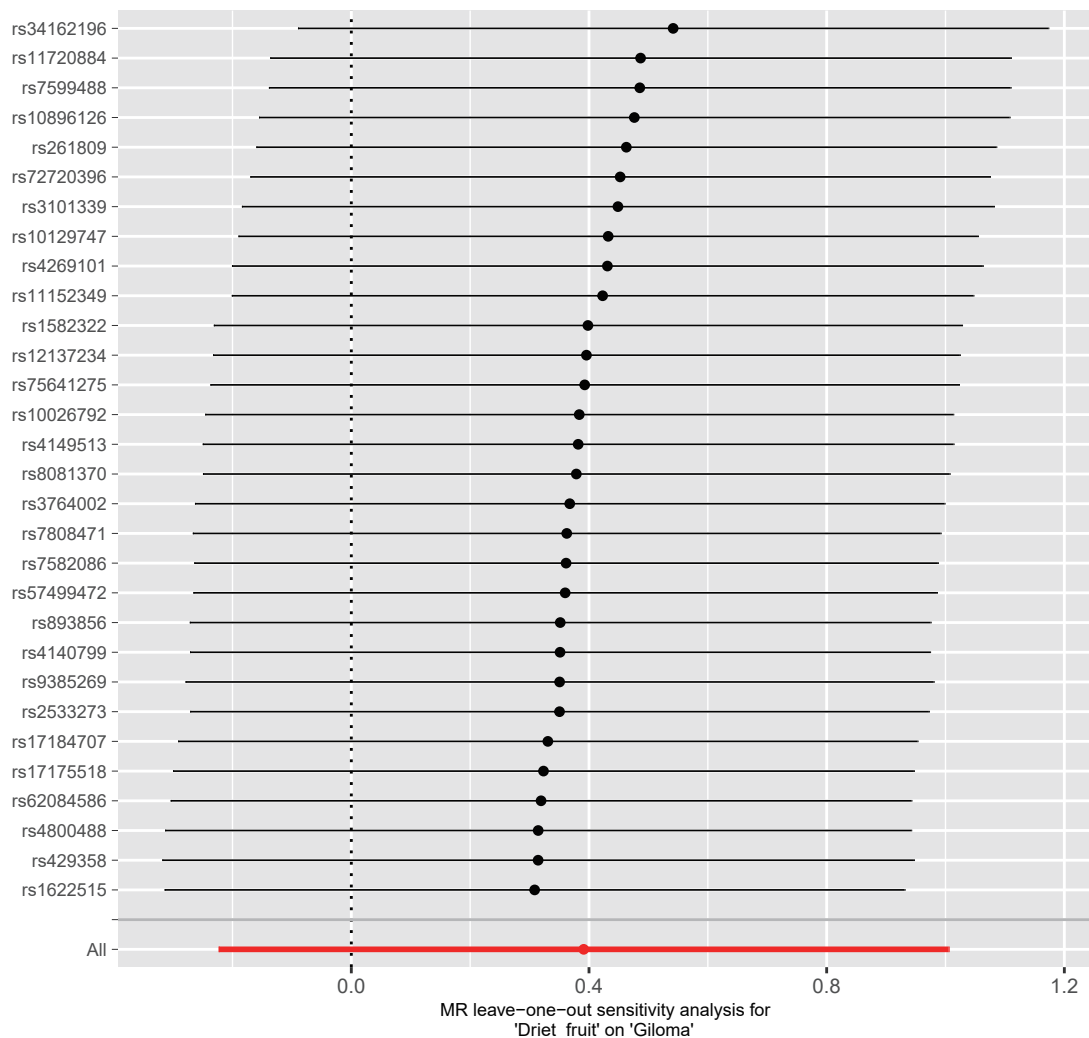

B

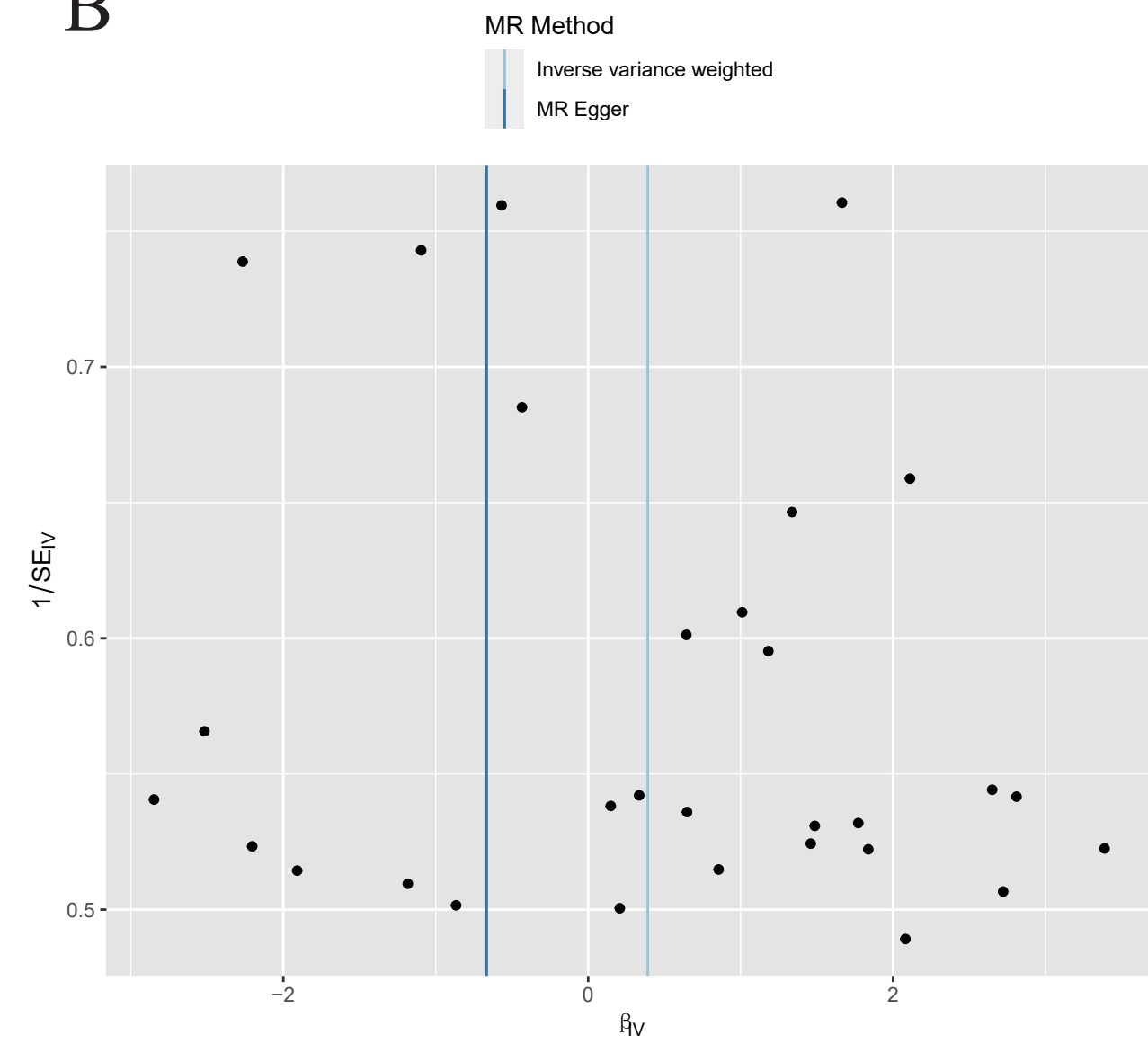

C

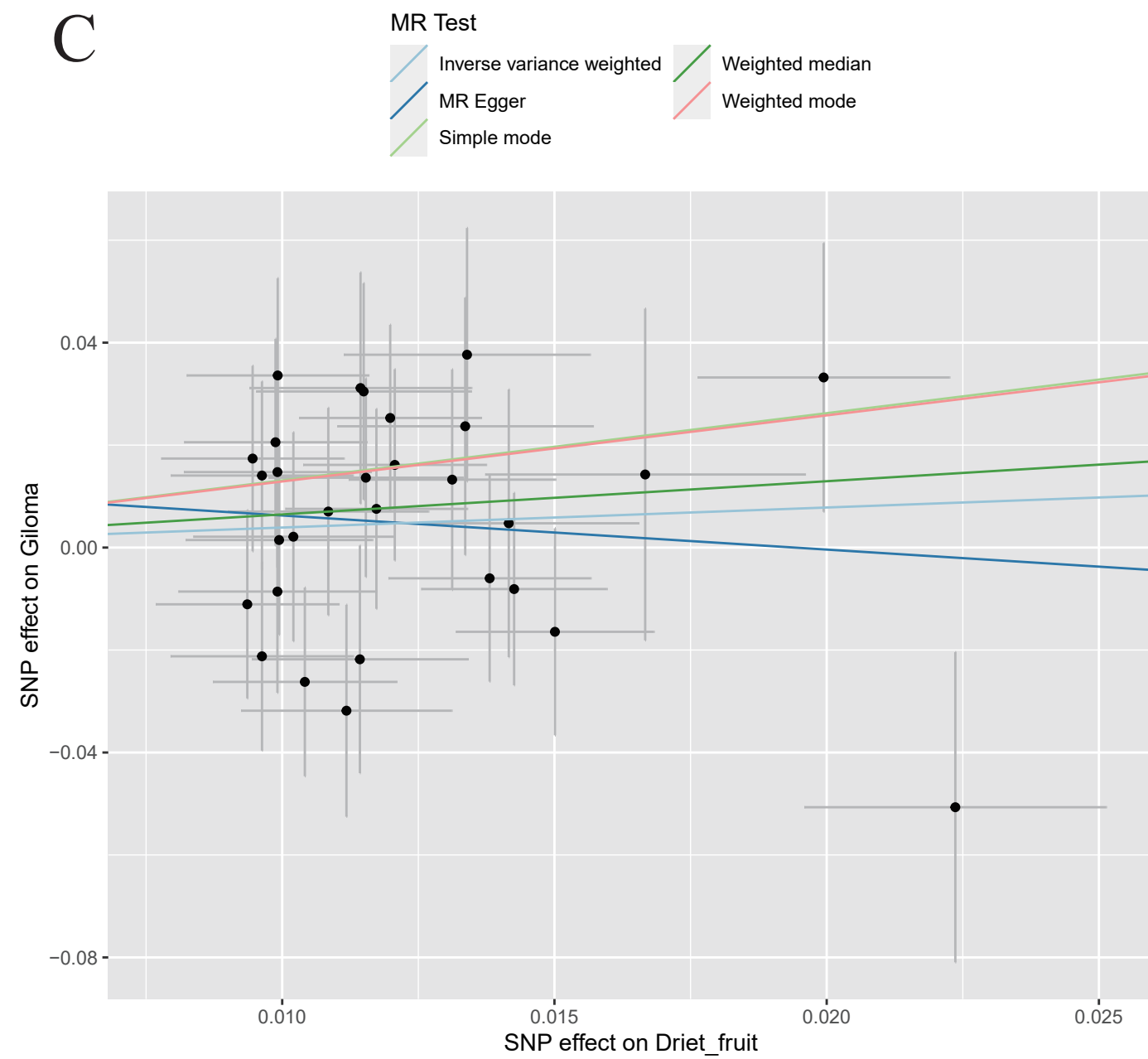

D

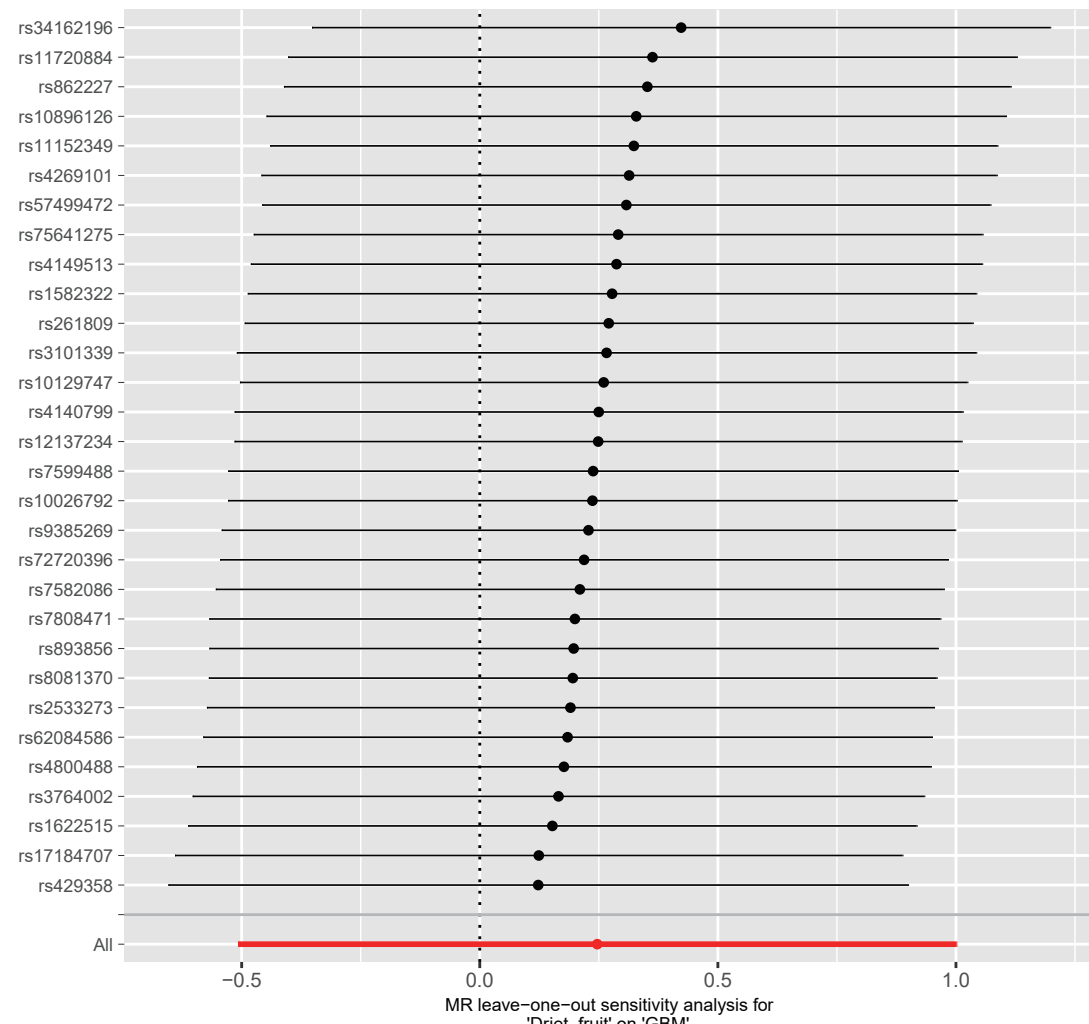

E

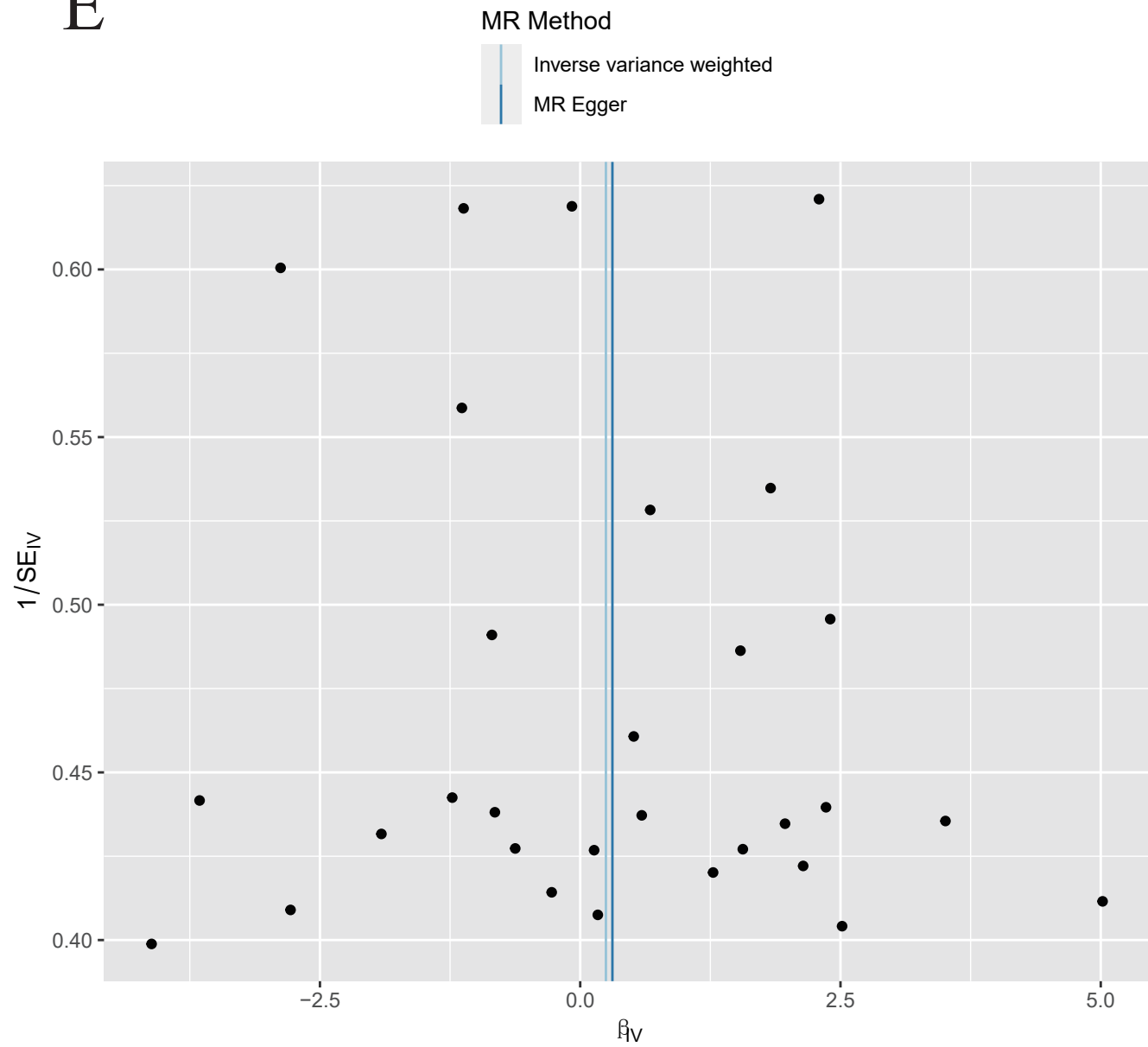

F

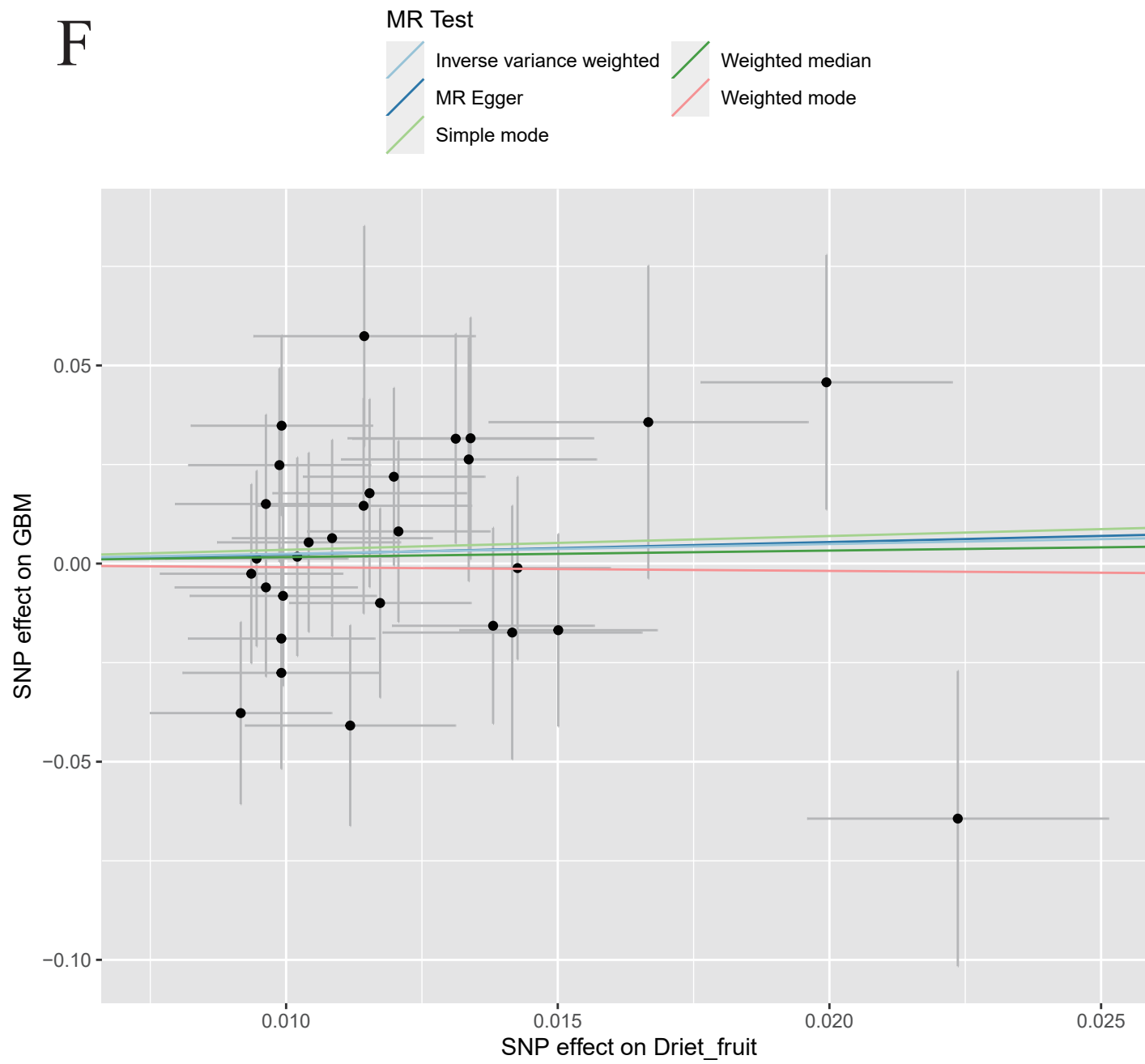

G

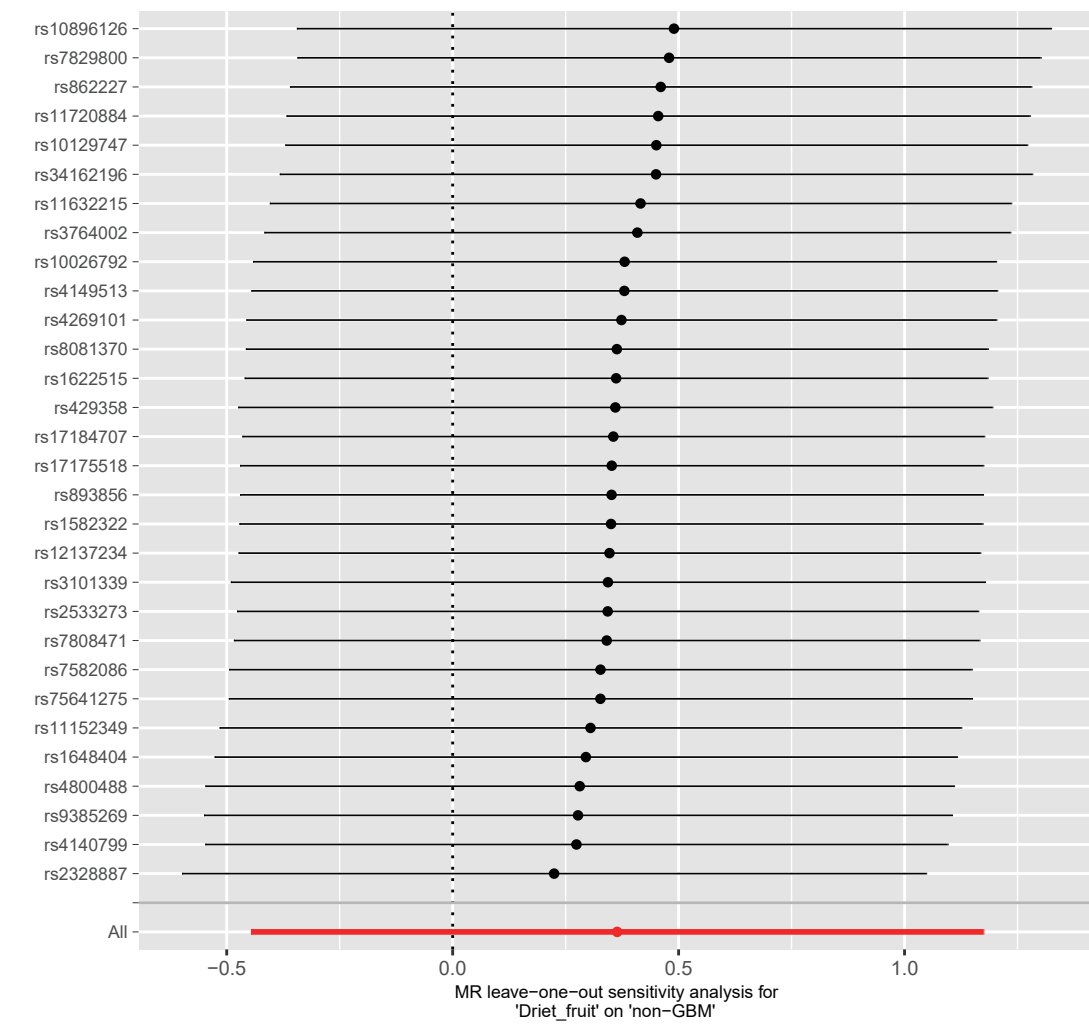

H

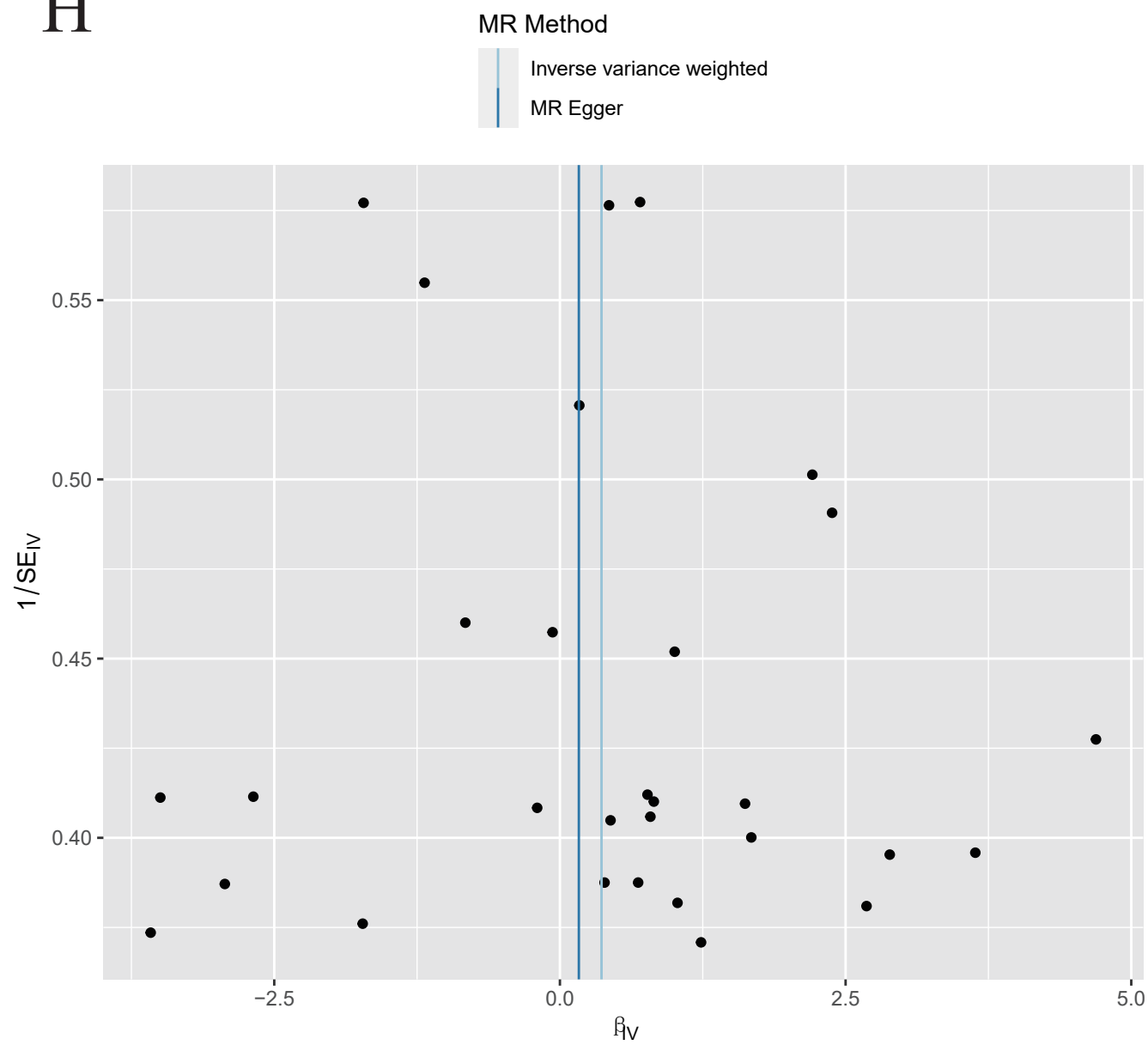

I

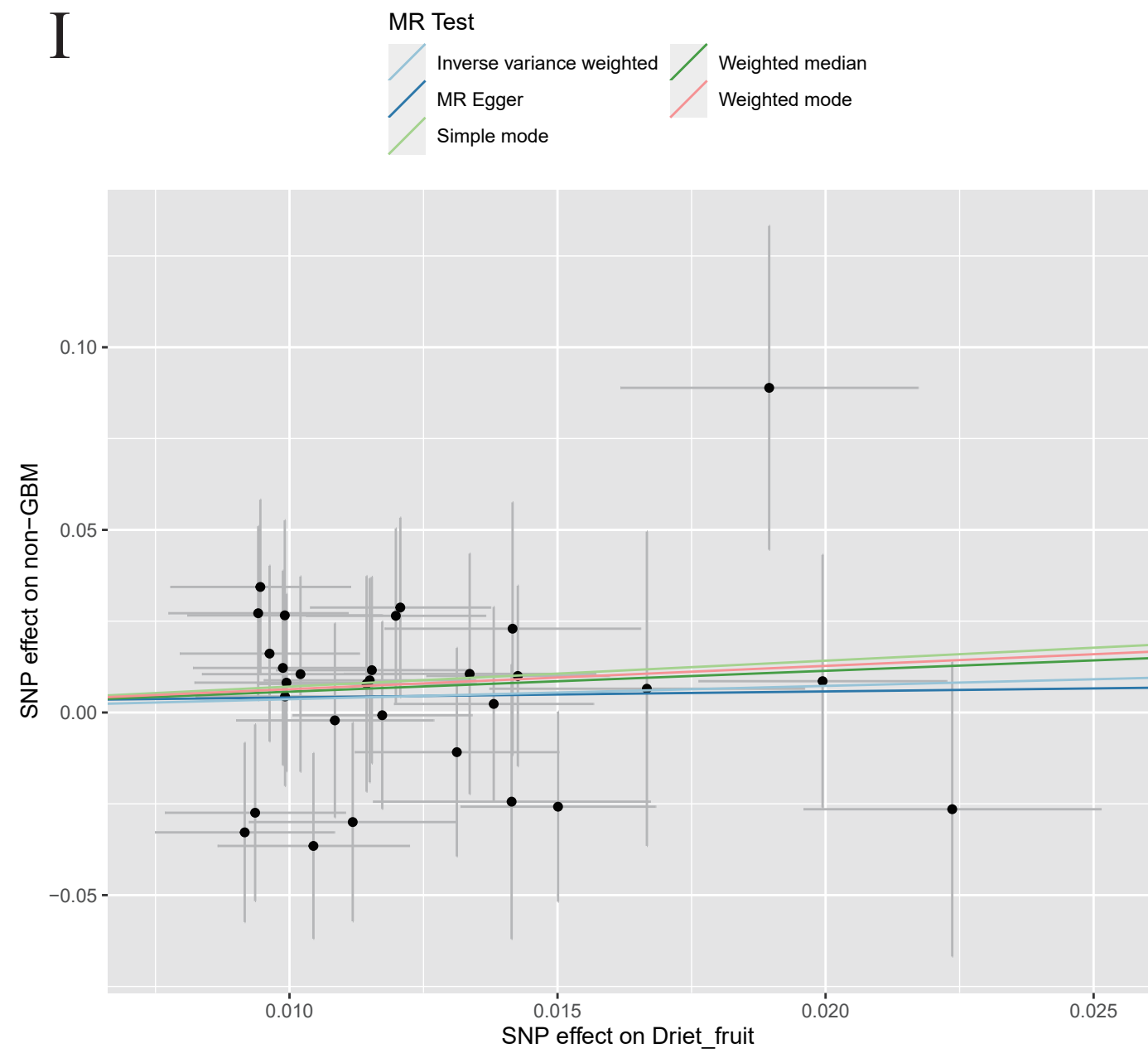

Supplement: Supplementary file 1 [file nutrients-17-00582-s001.zip › nutrients-3462880-supplementary/Sup_14.pdf]

A

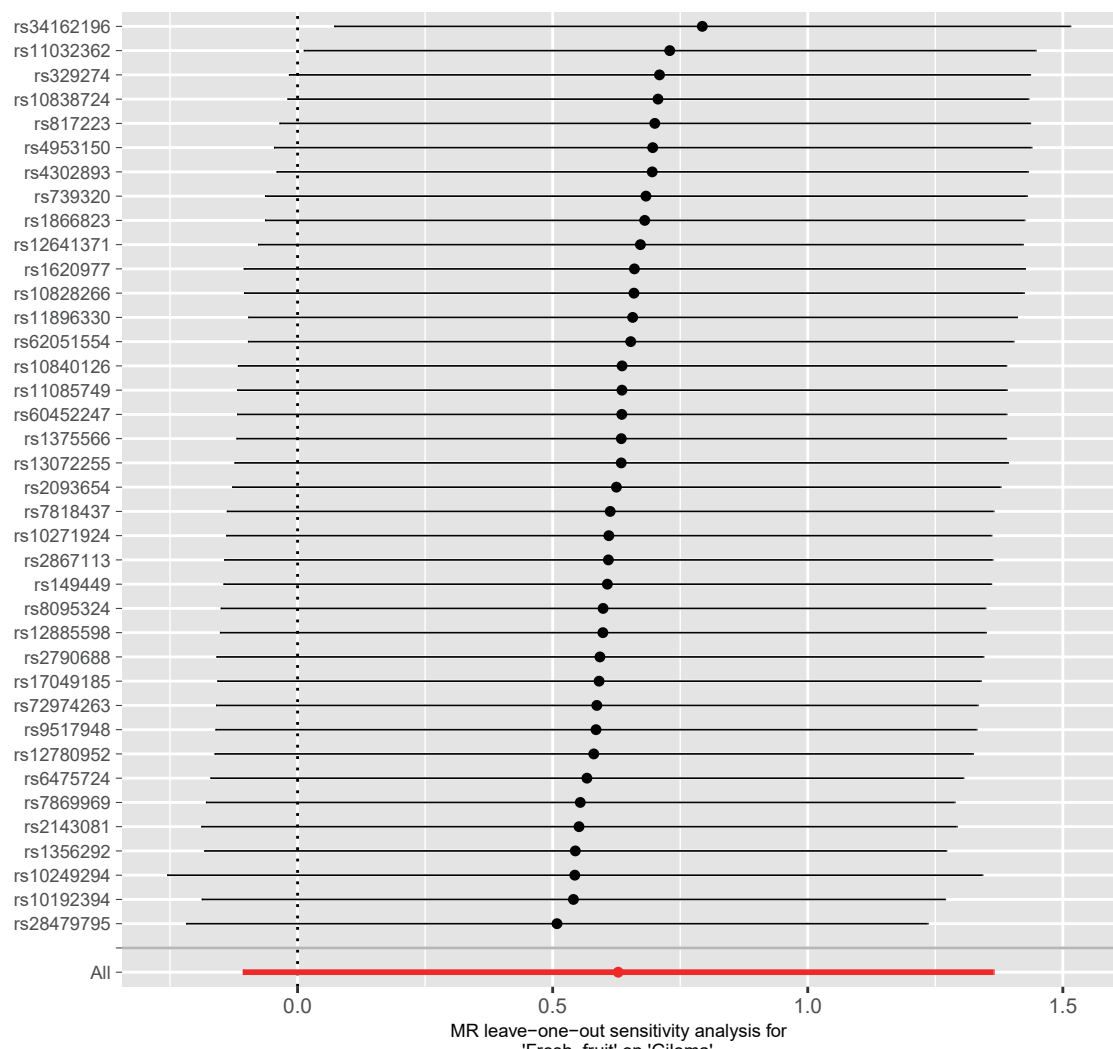

B

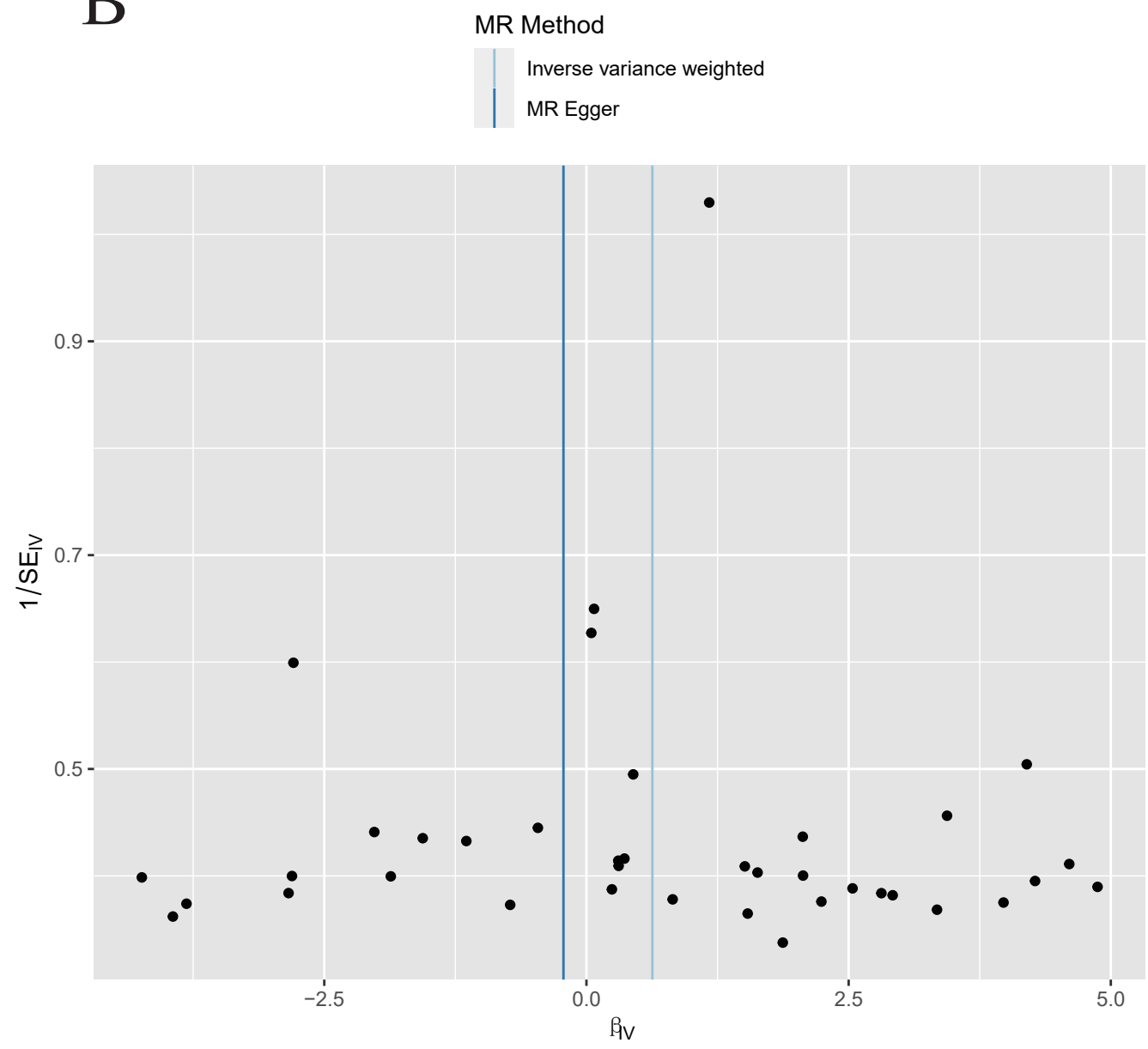

C

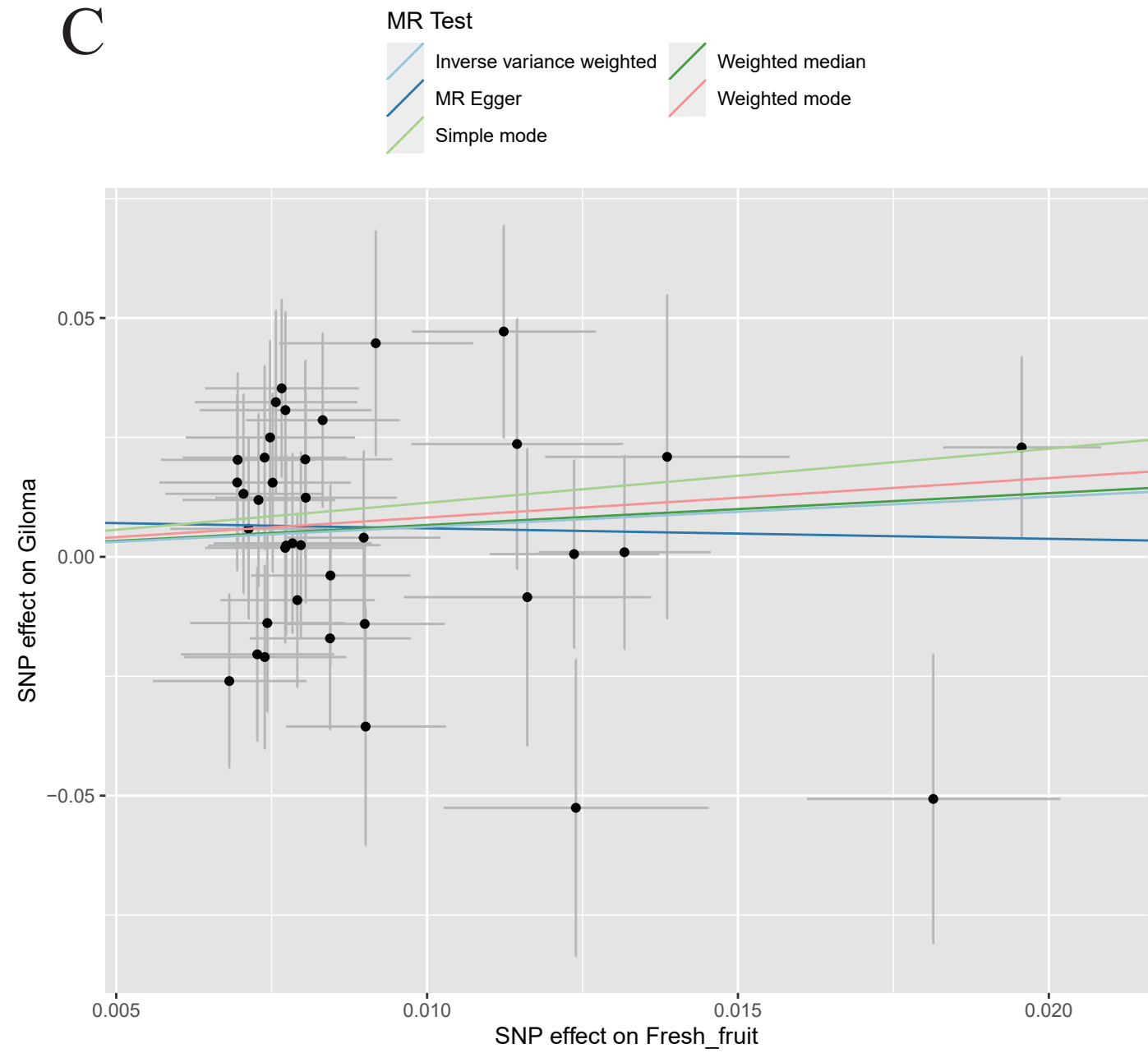

D

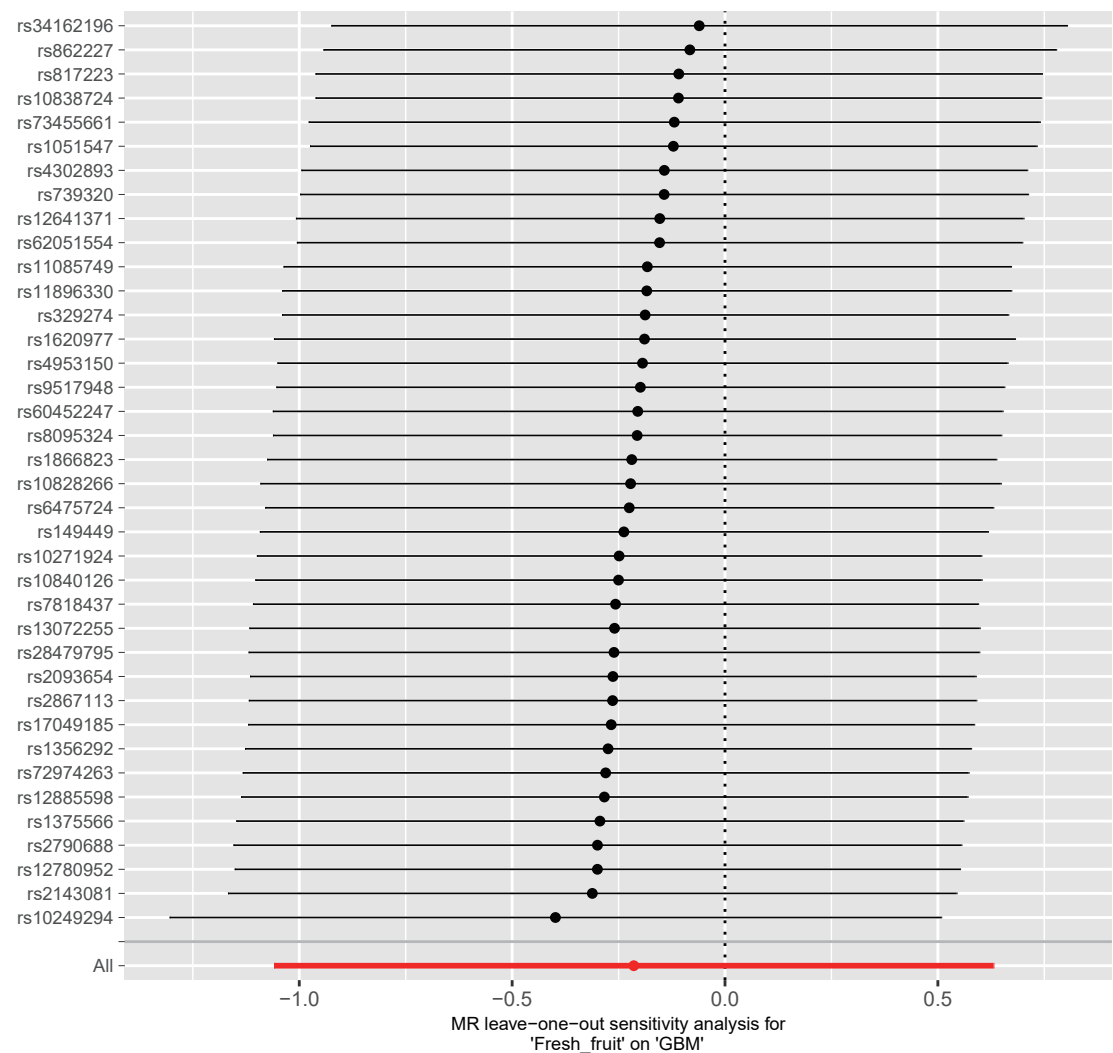

E

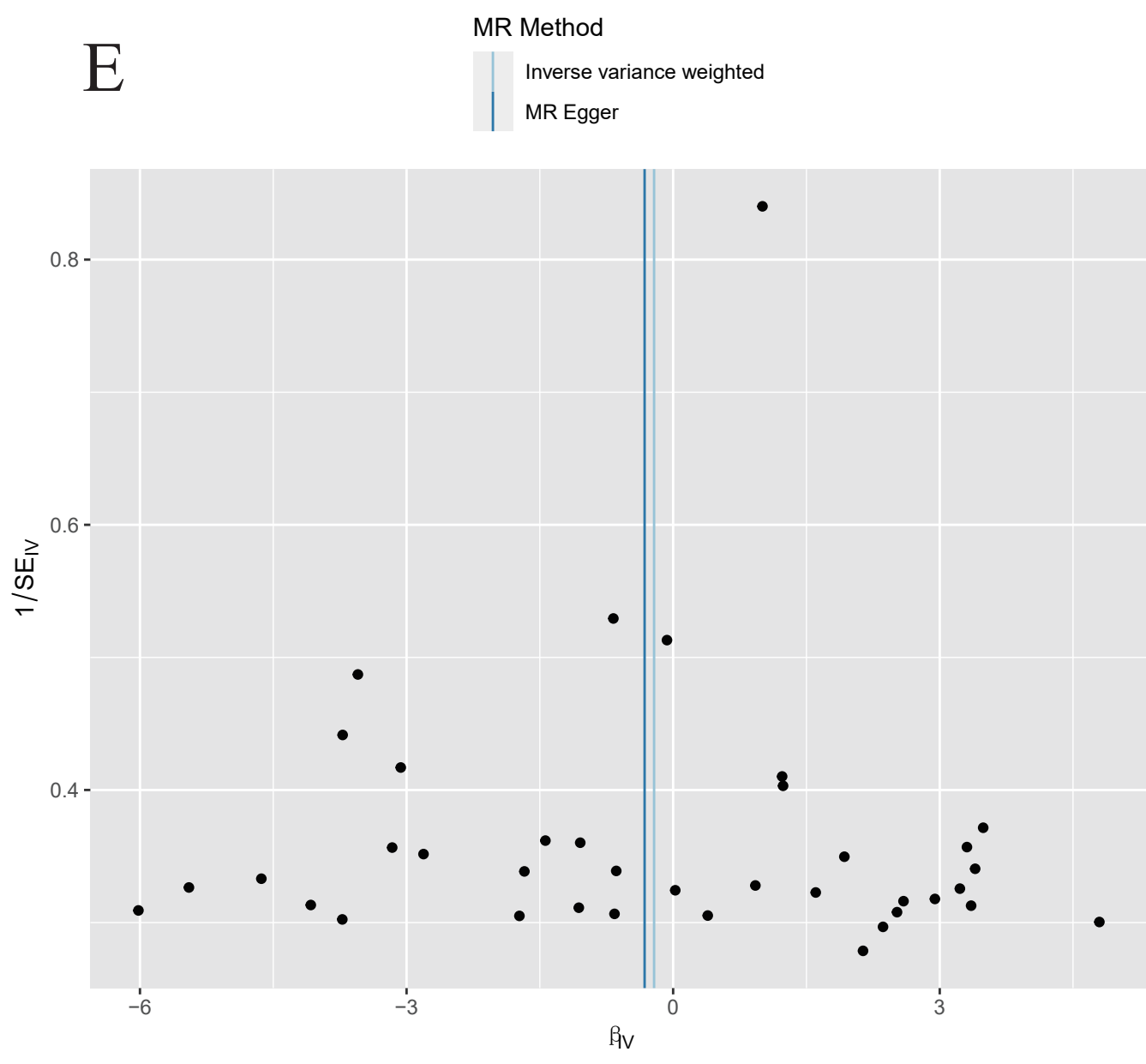

F

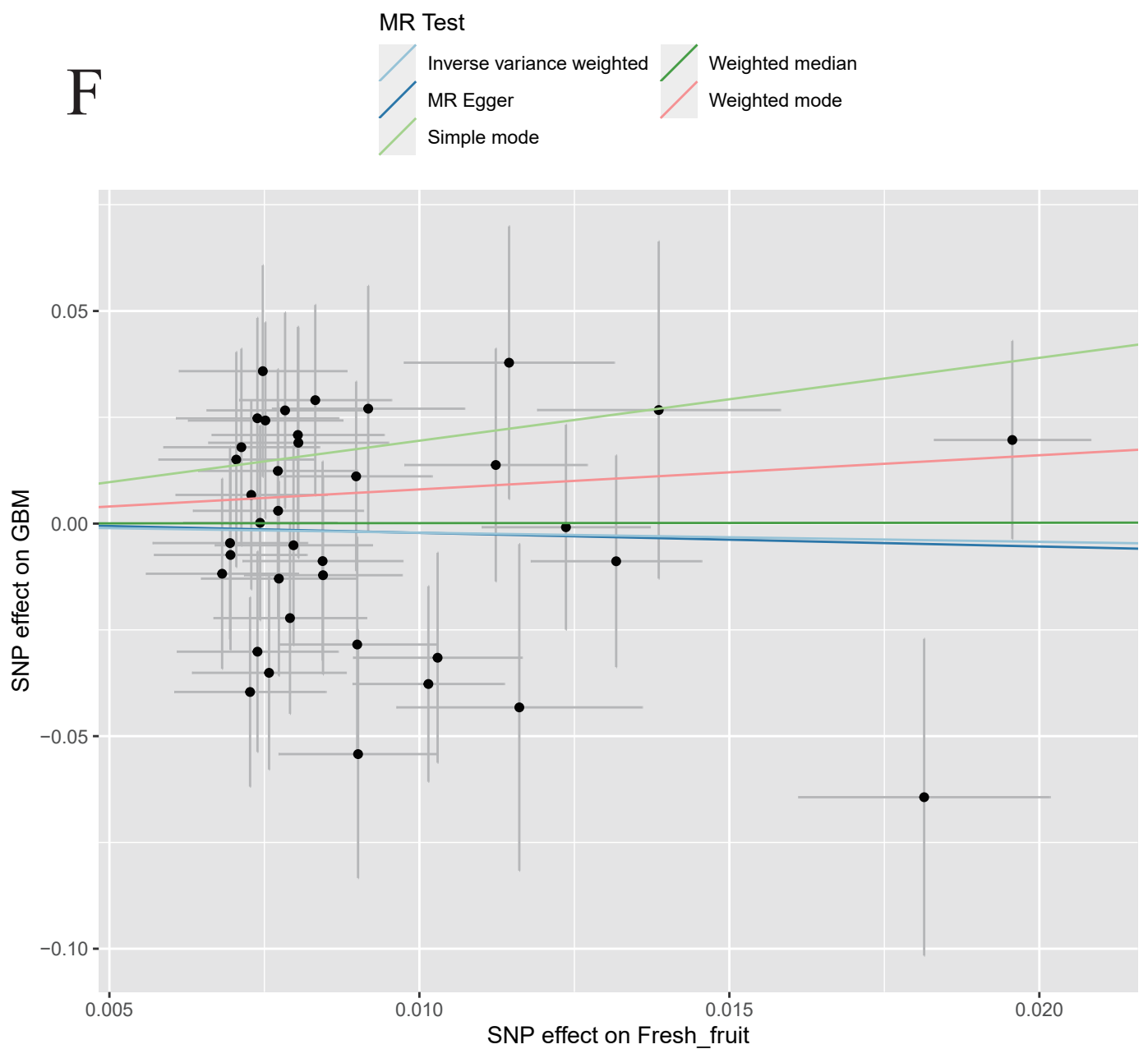

G

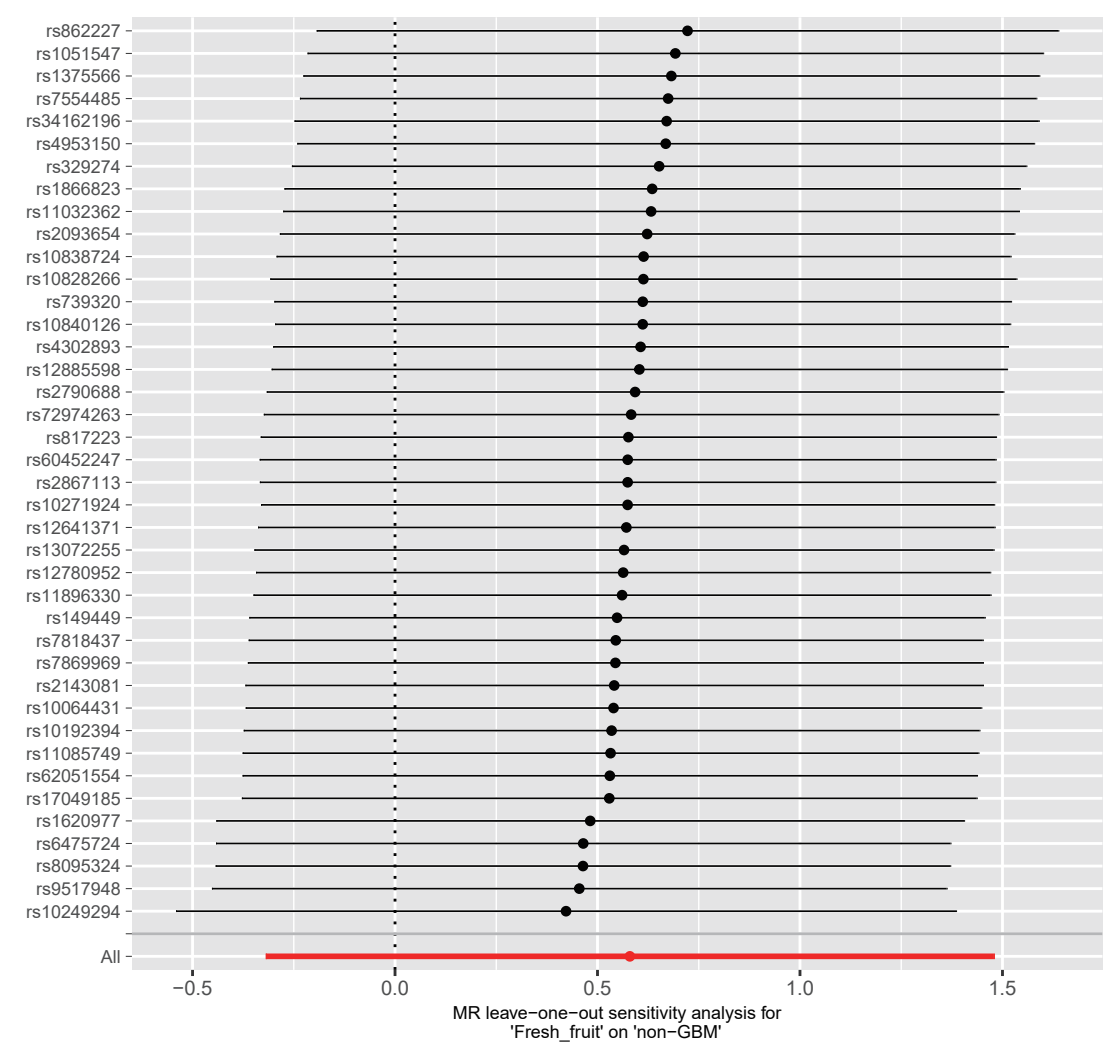

H

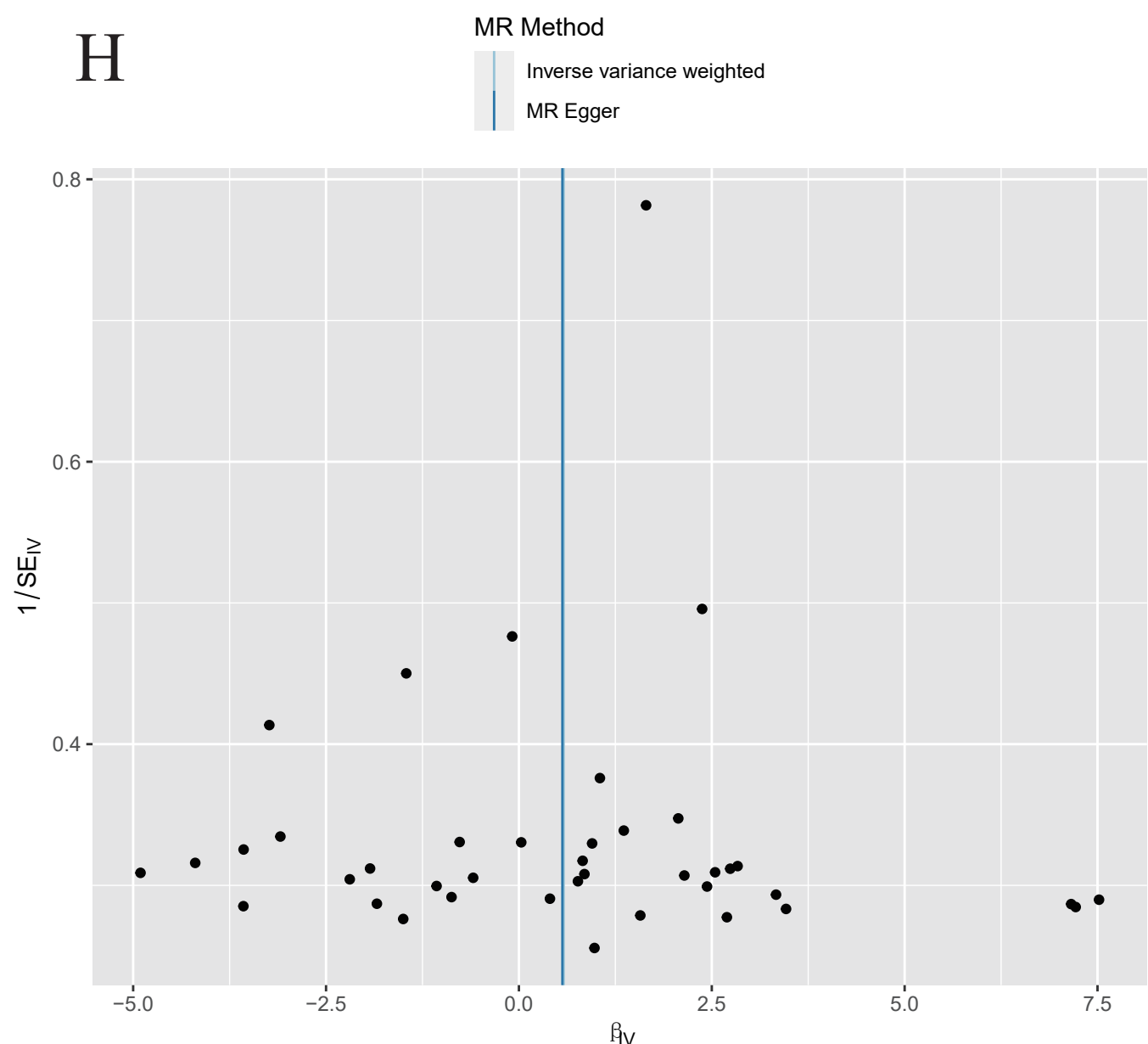

I

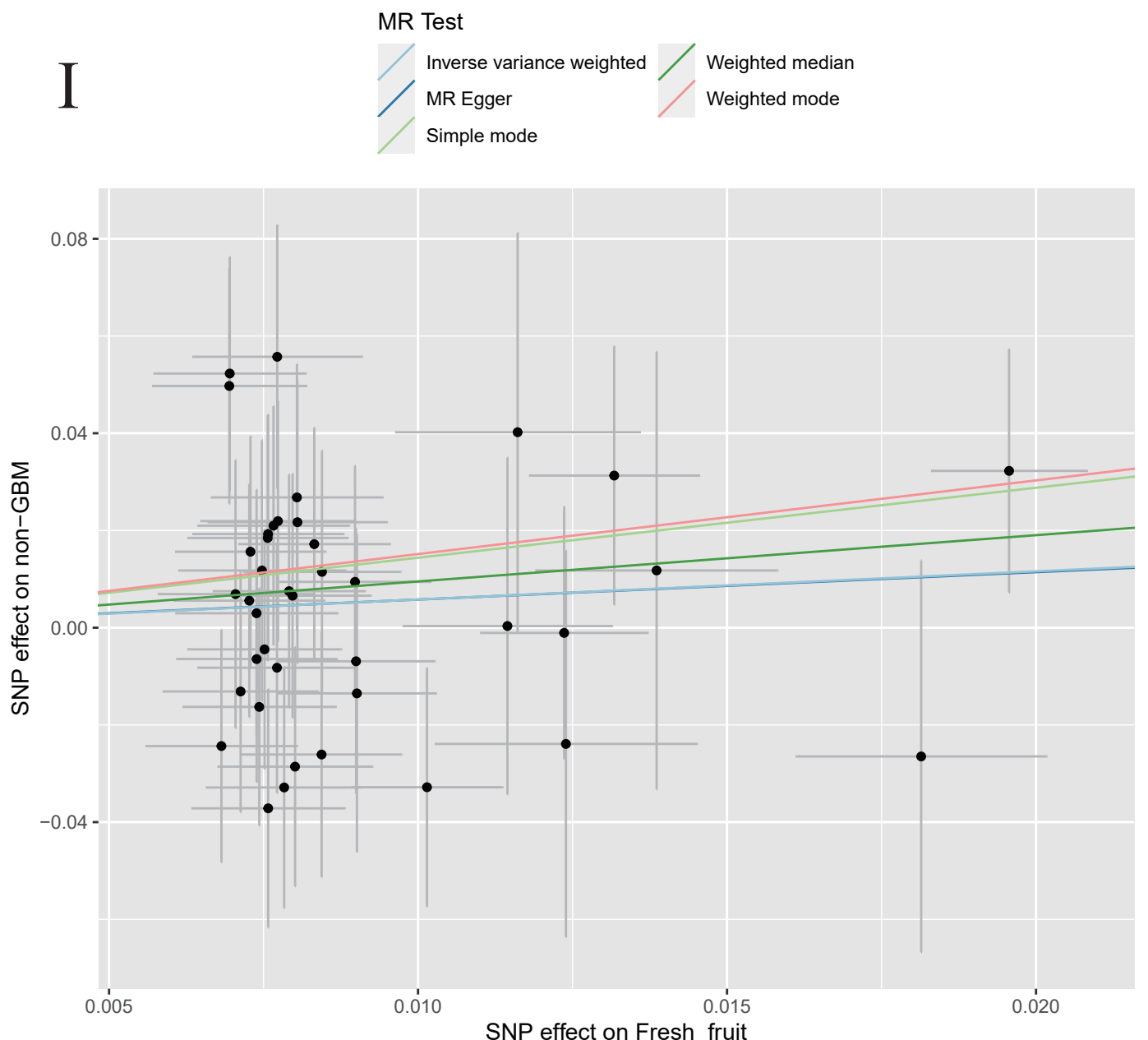

Supplement: Supplementary file 1 [file nutrients-17-00582-s001.zip › nutrients-3462880-supplementary/Sup_15.pdf]

A

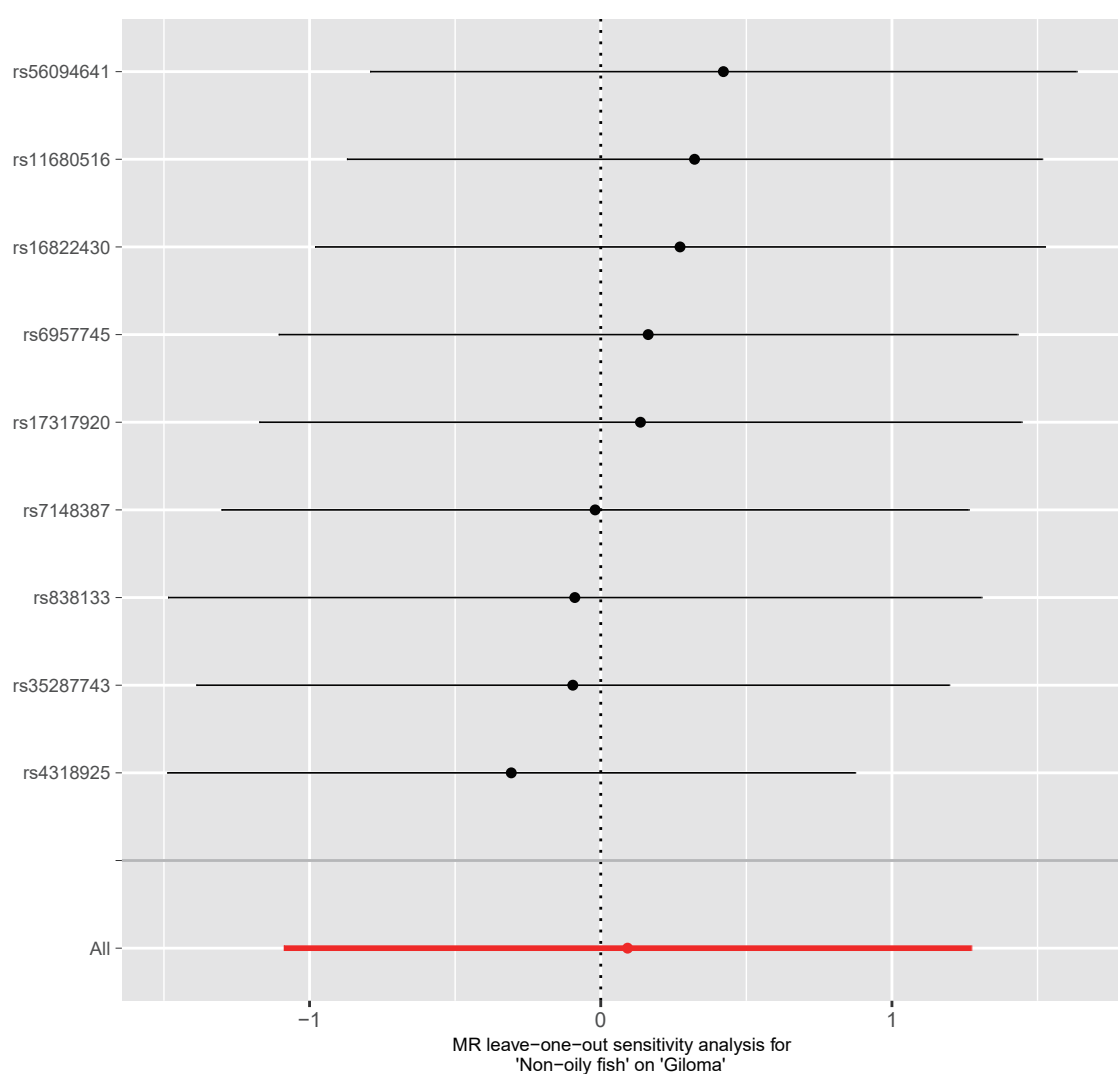

B

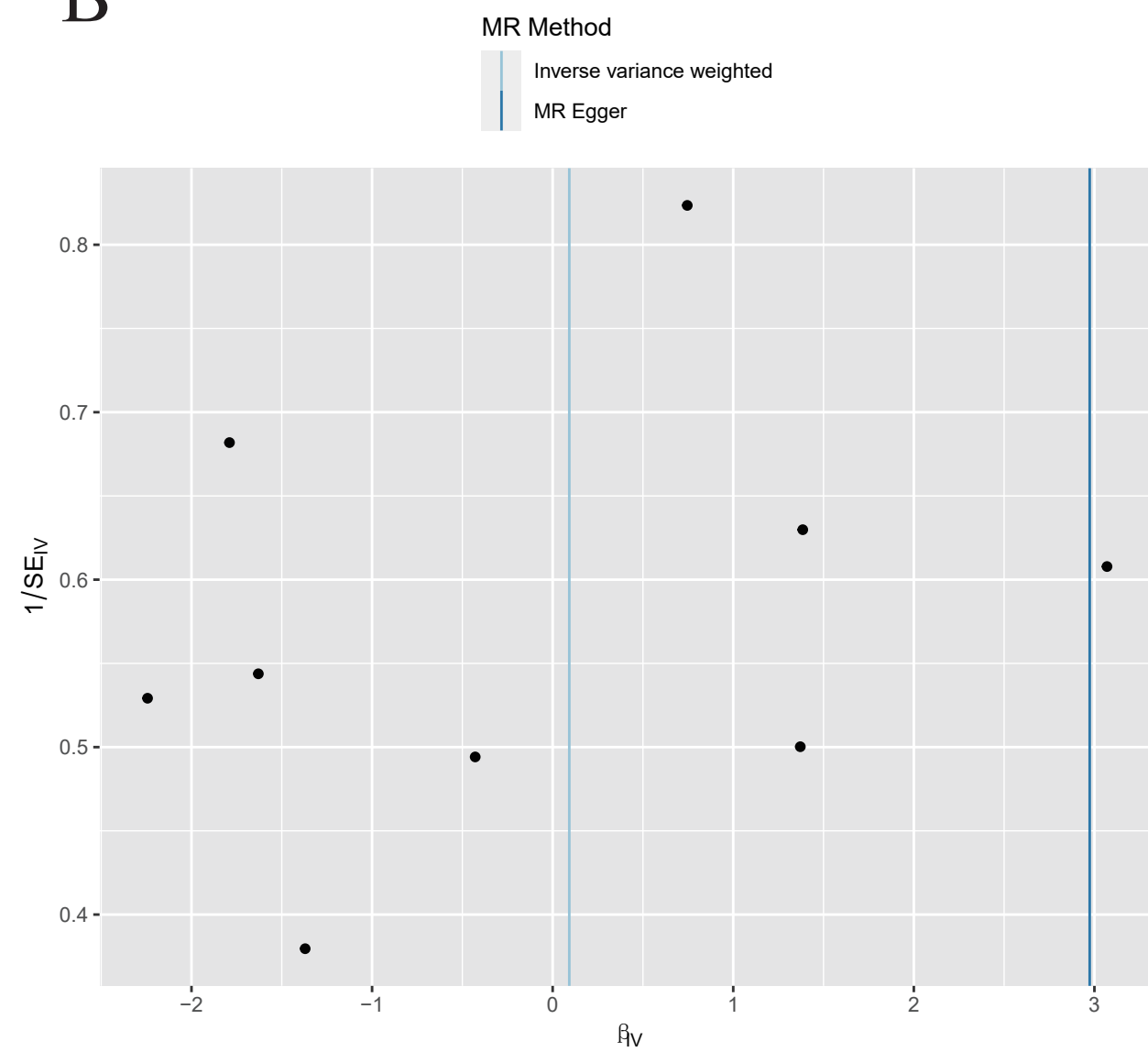

C

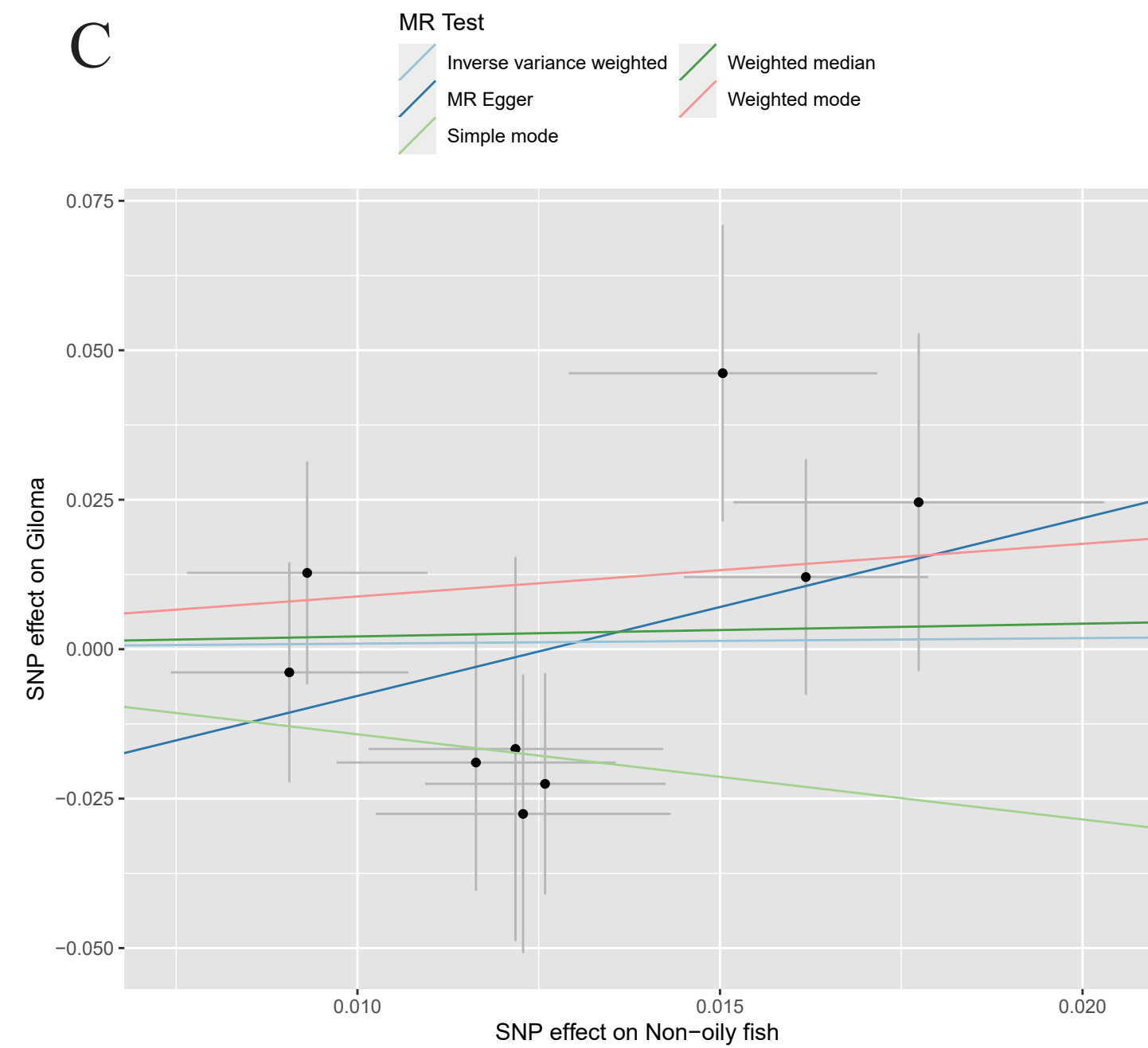

D

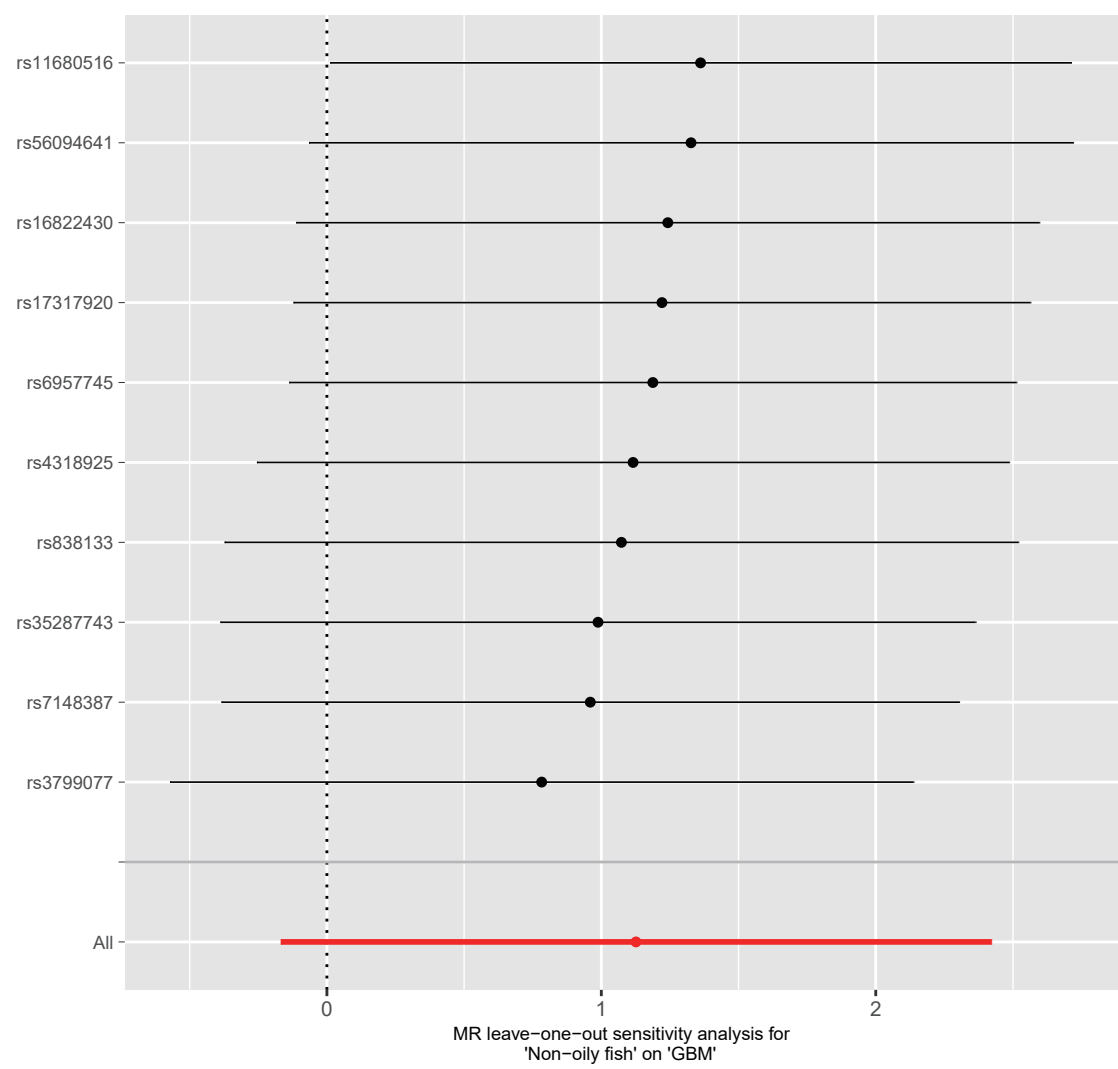

E

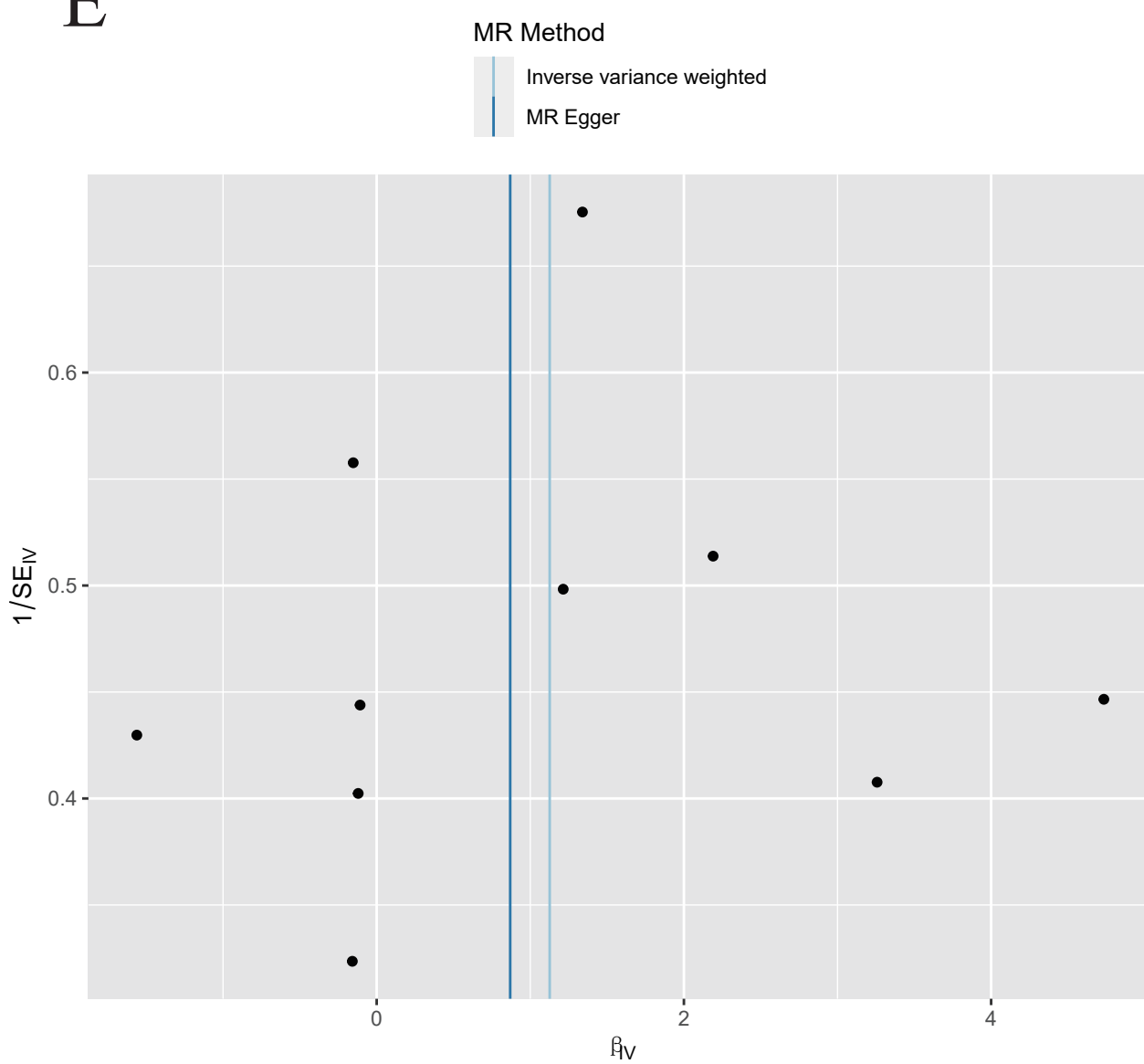

F

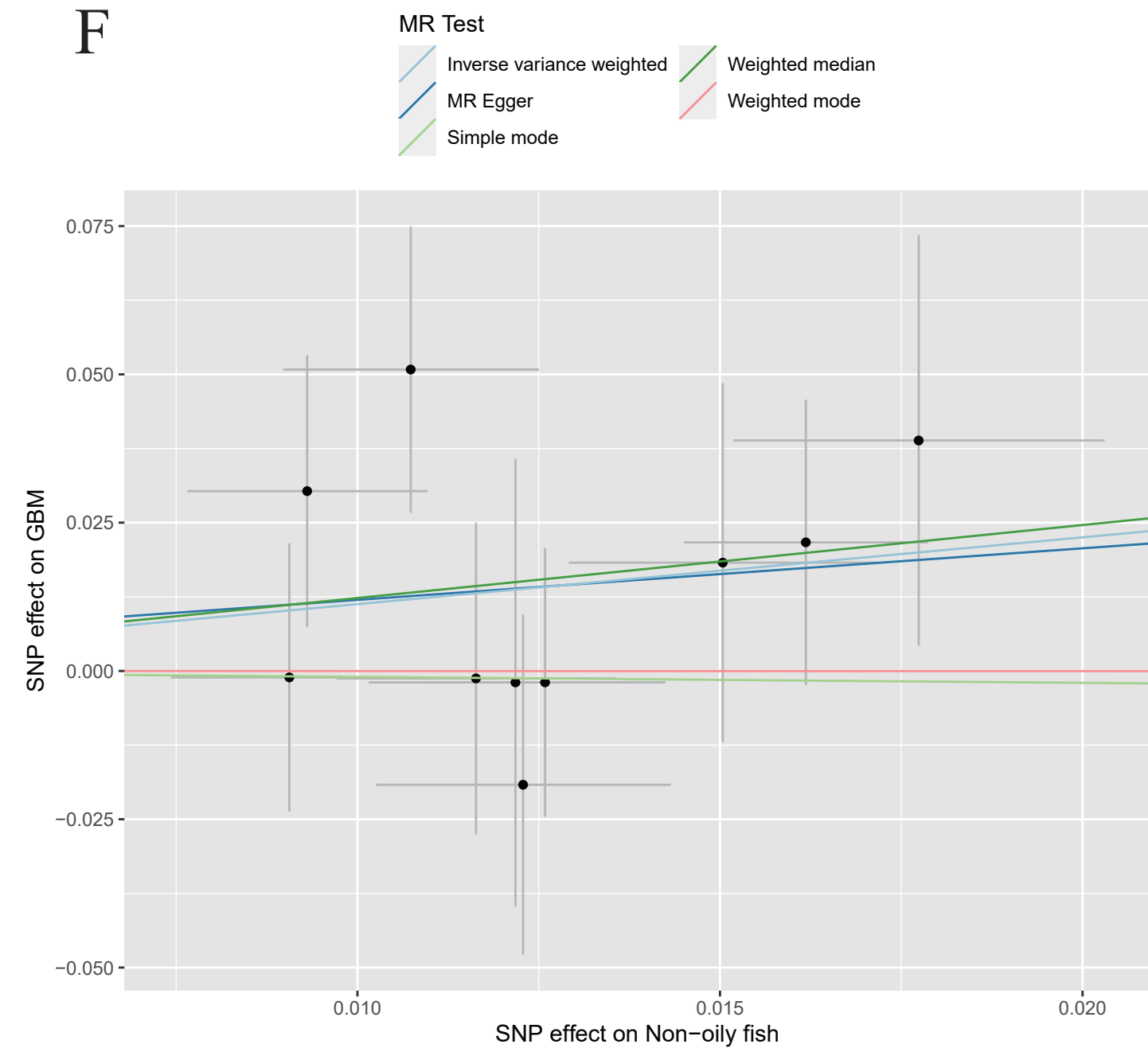

G

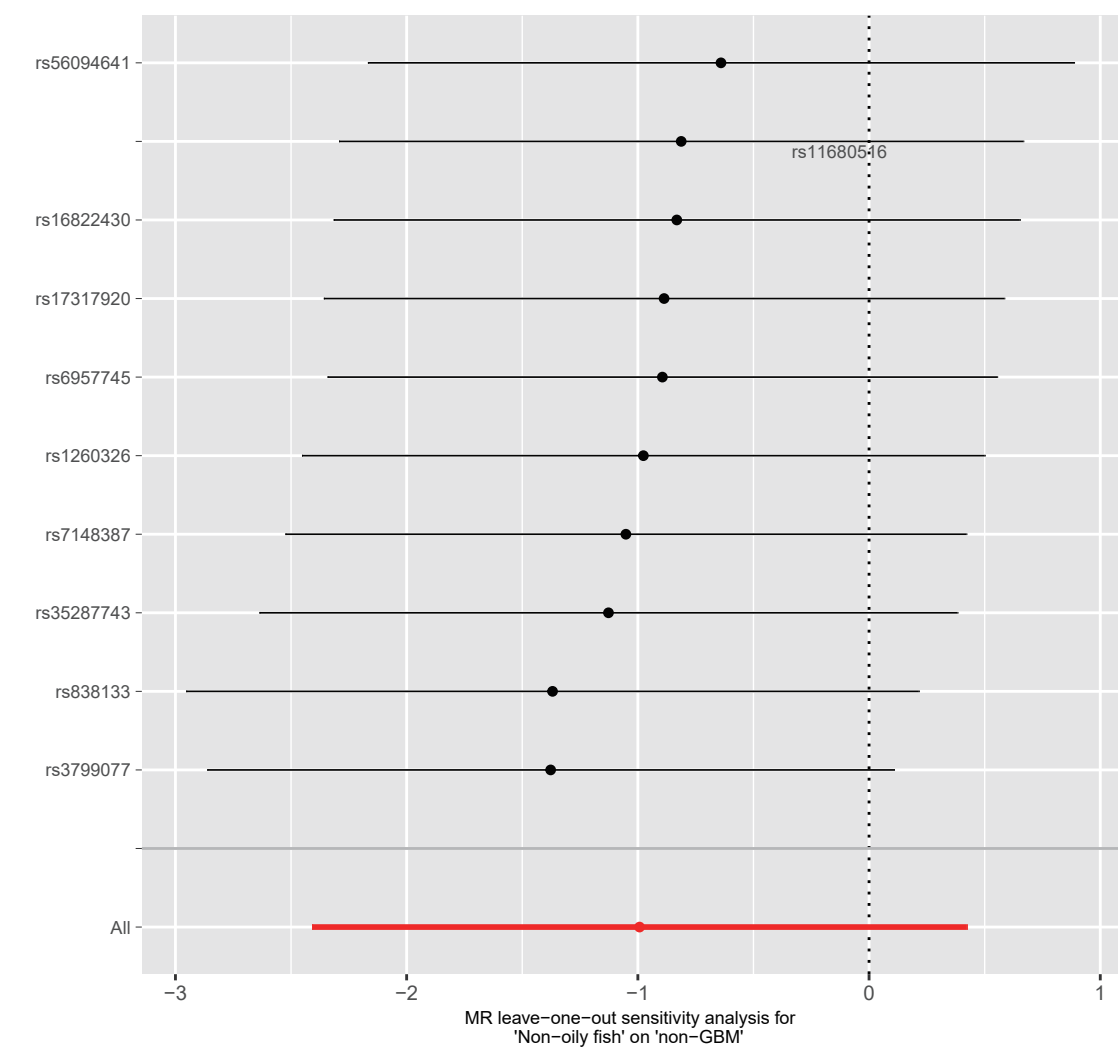

H

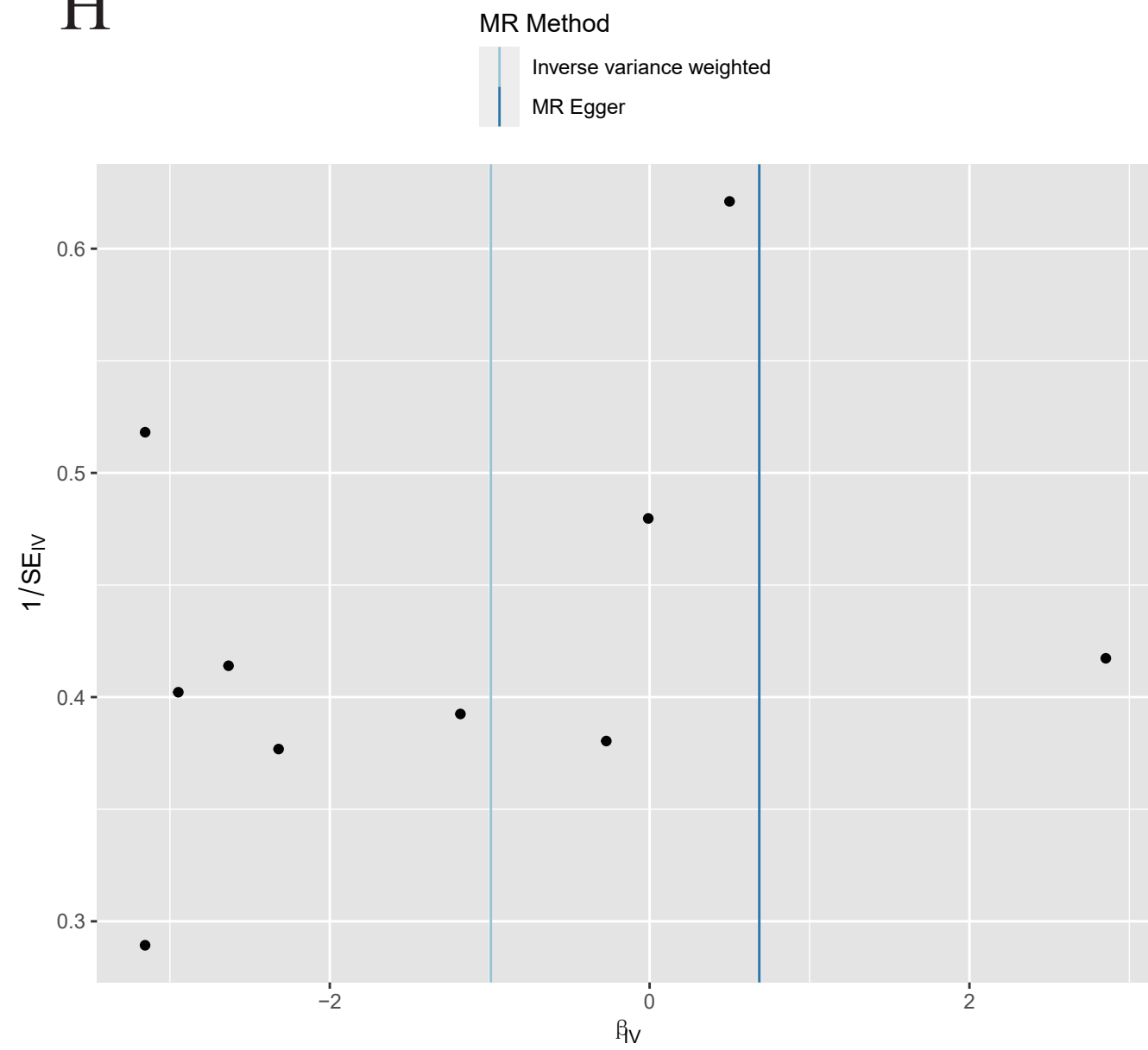

I

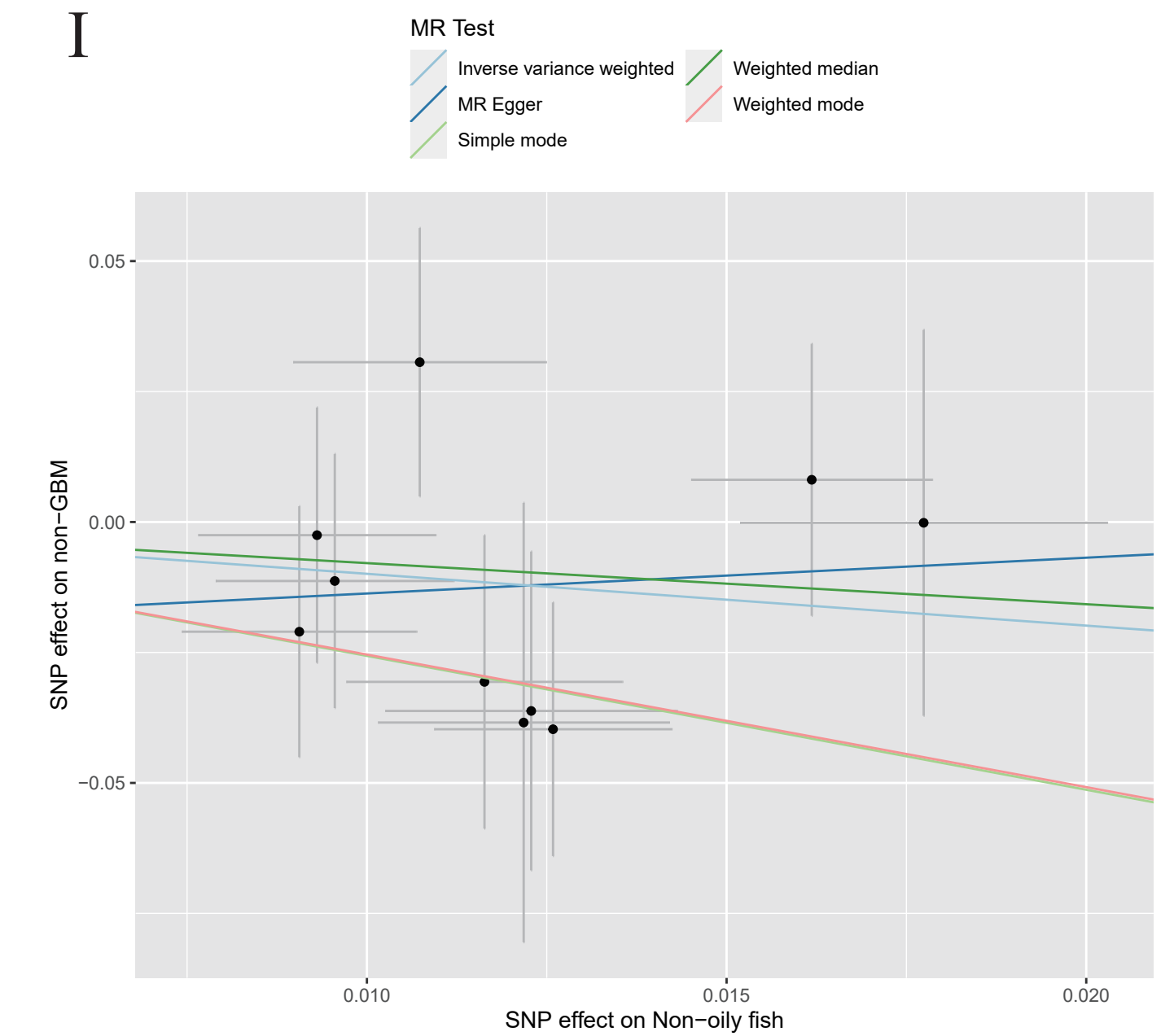

Supplement: Supplementary file 1 [file nutrients-17-00582-s001.zip › nutrients-3462880-supplementary/Sup_16.pdf]

A

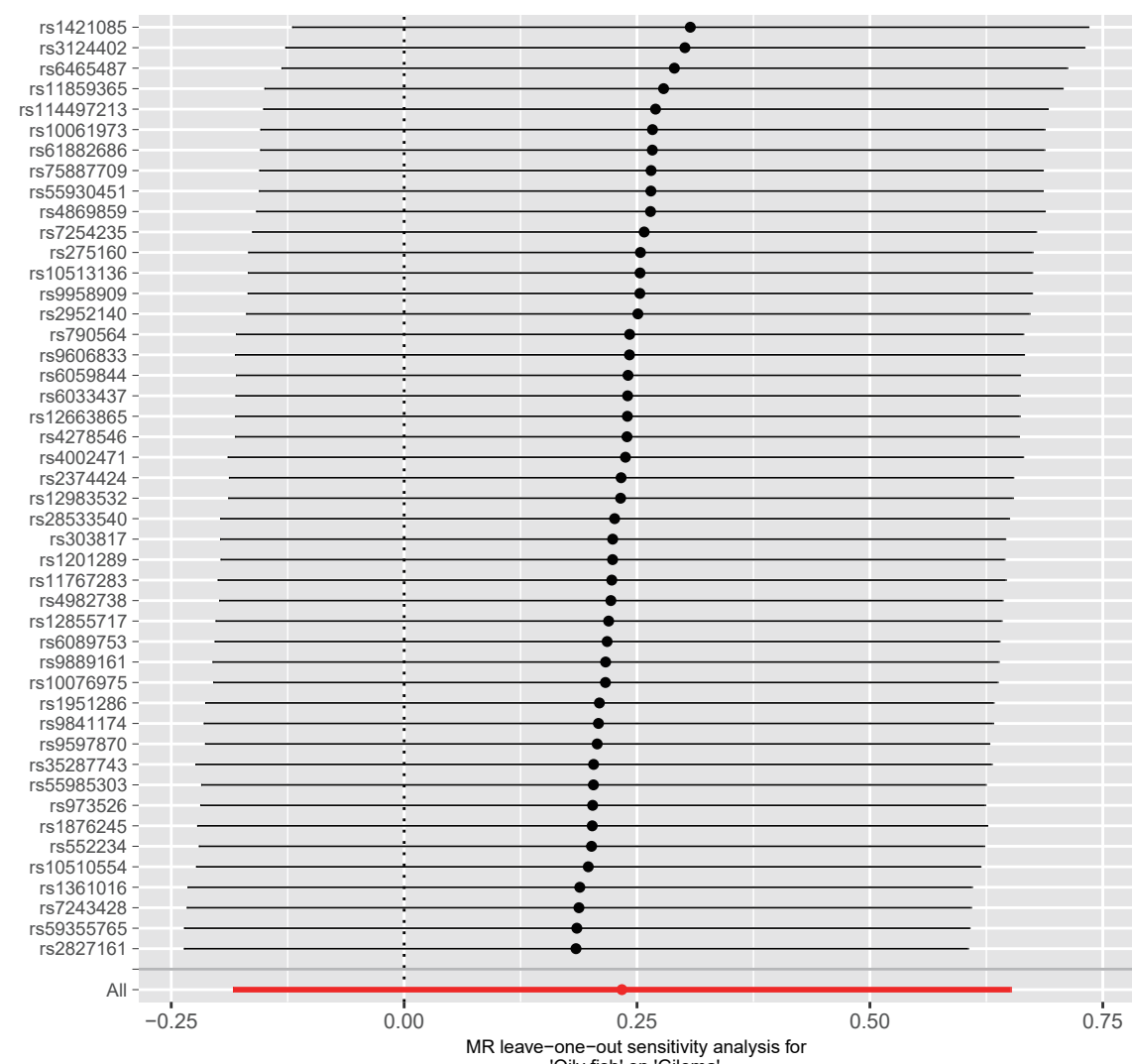

B

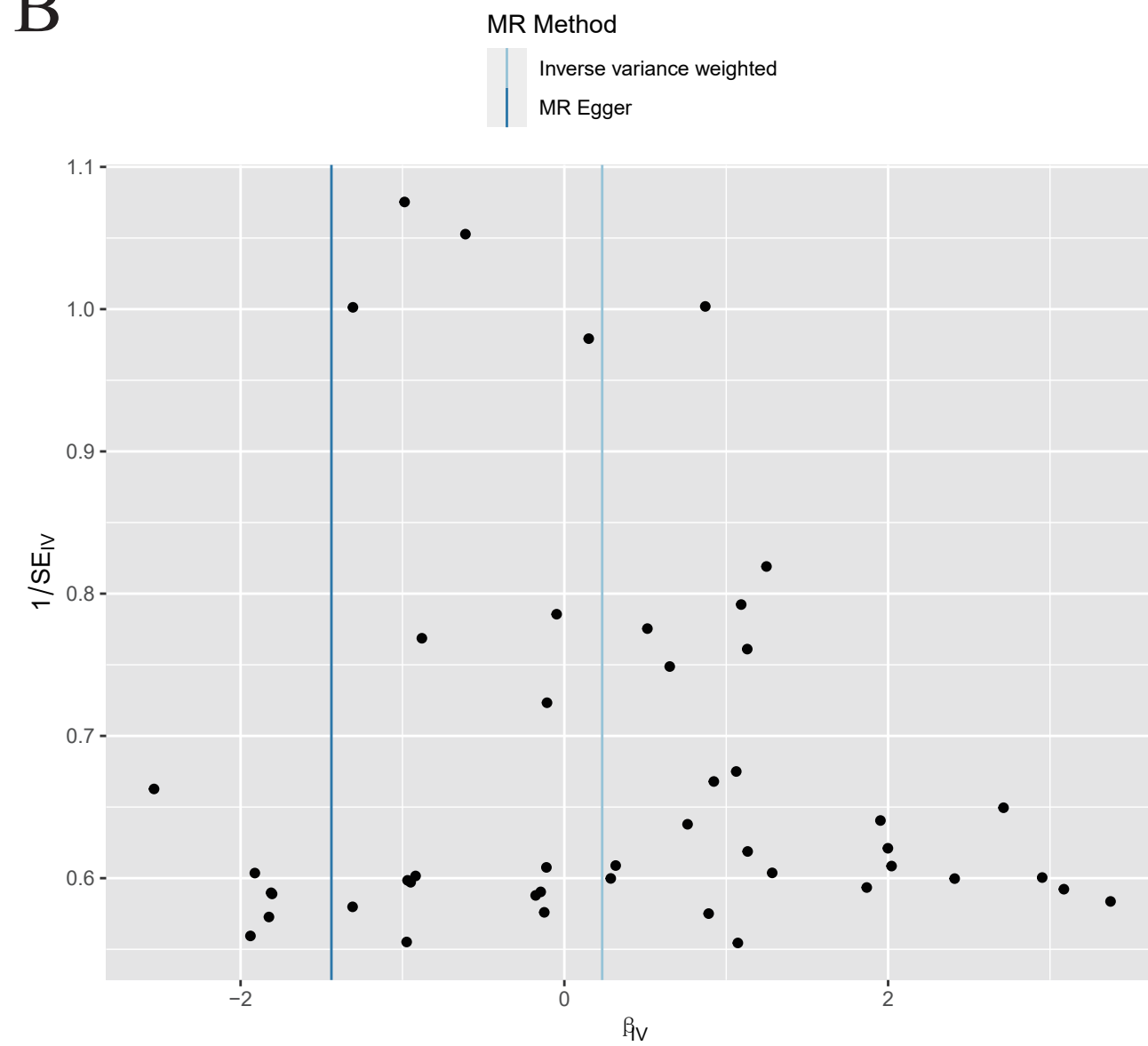

C

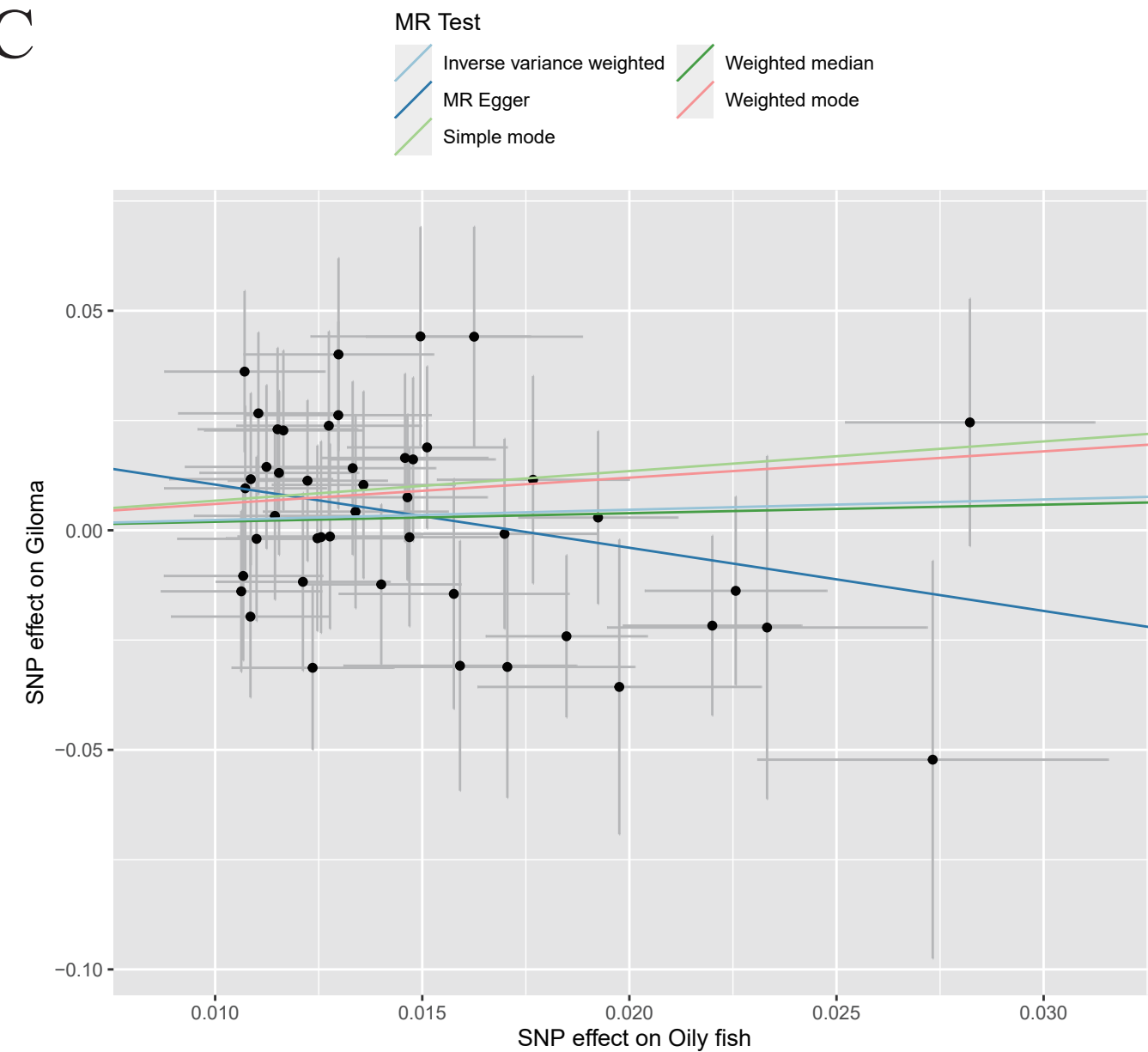

D

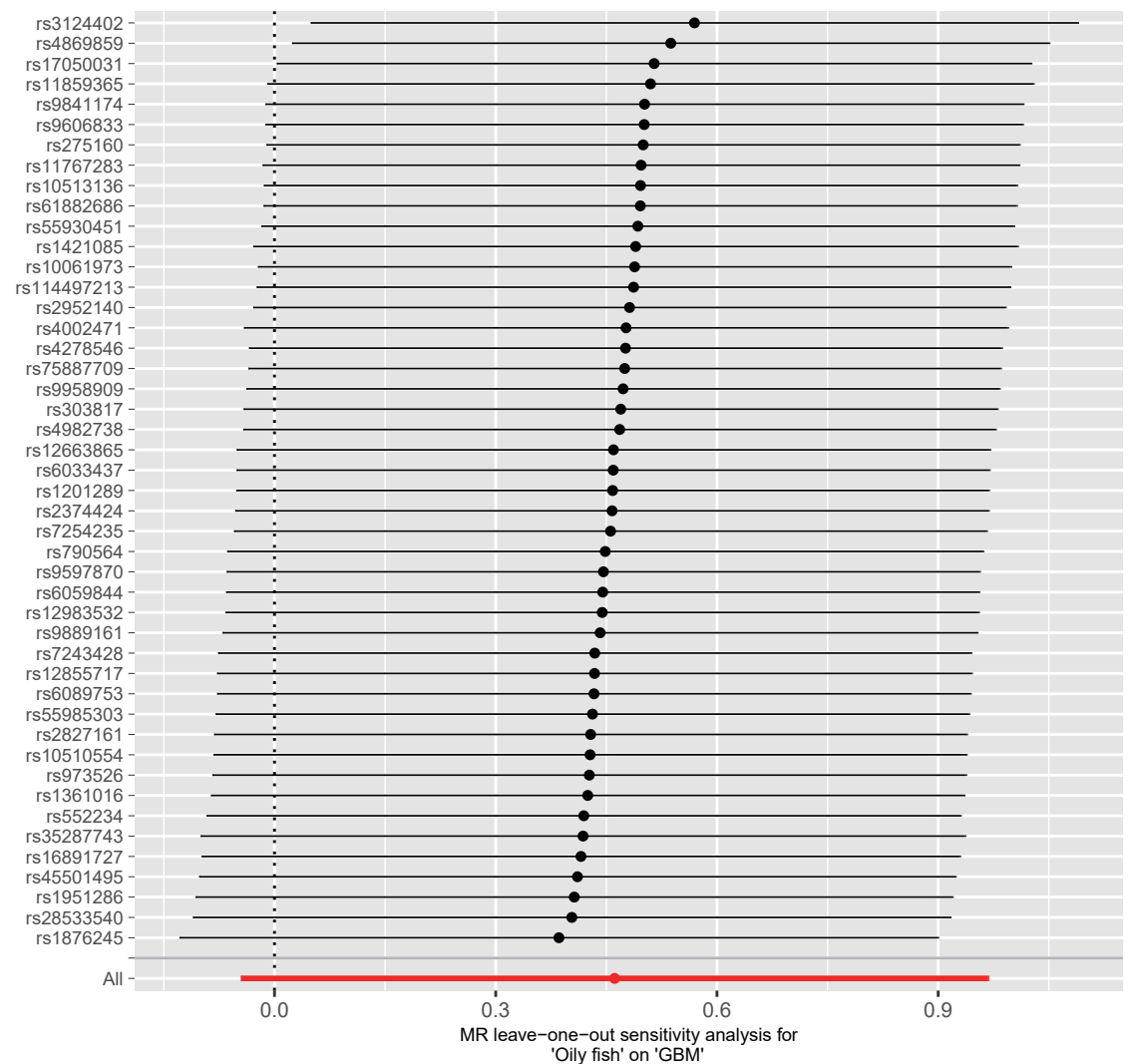

E

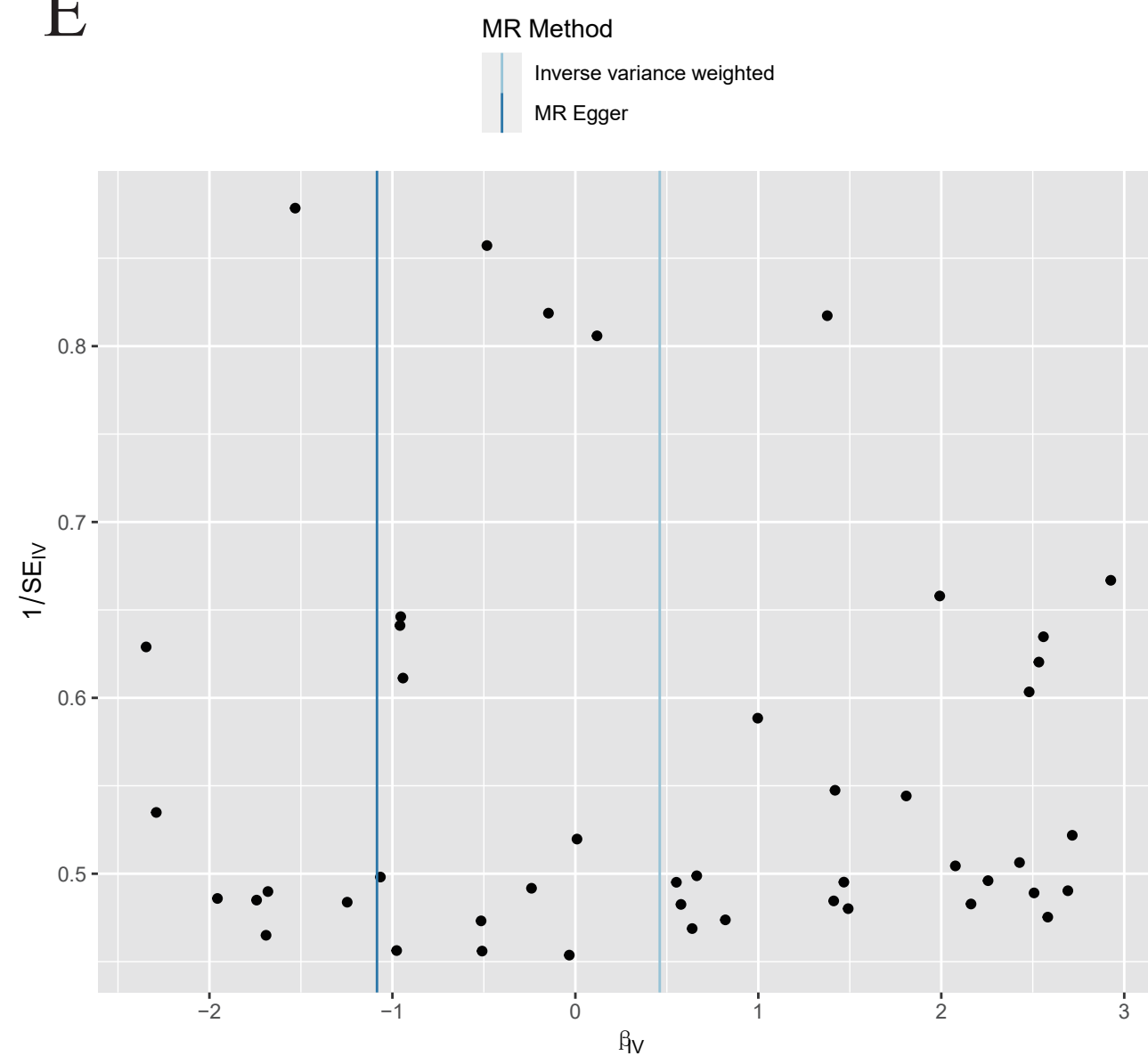

F

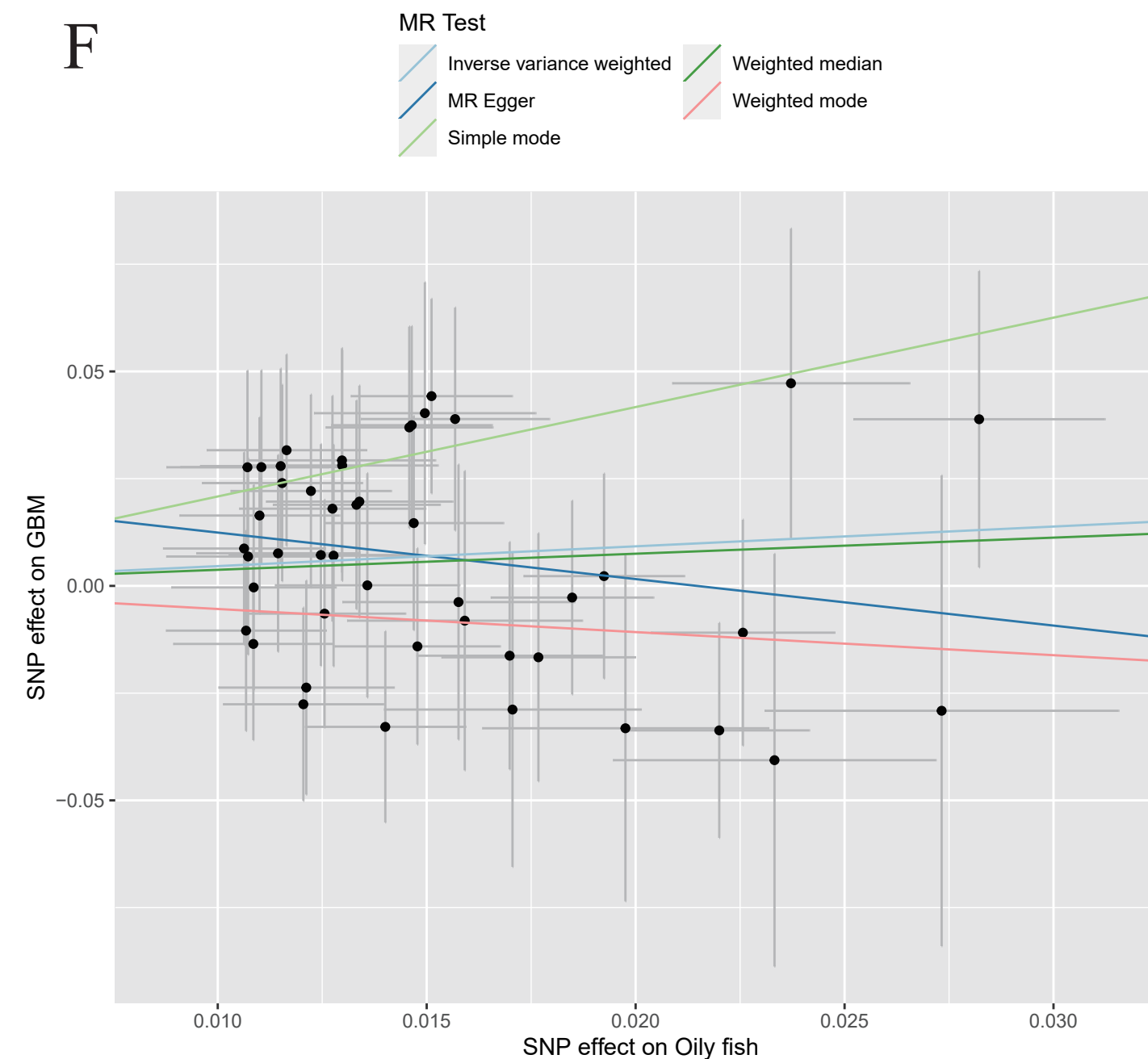

G

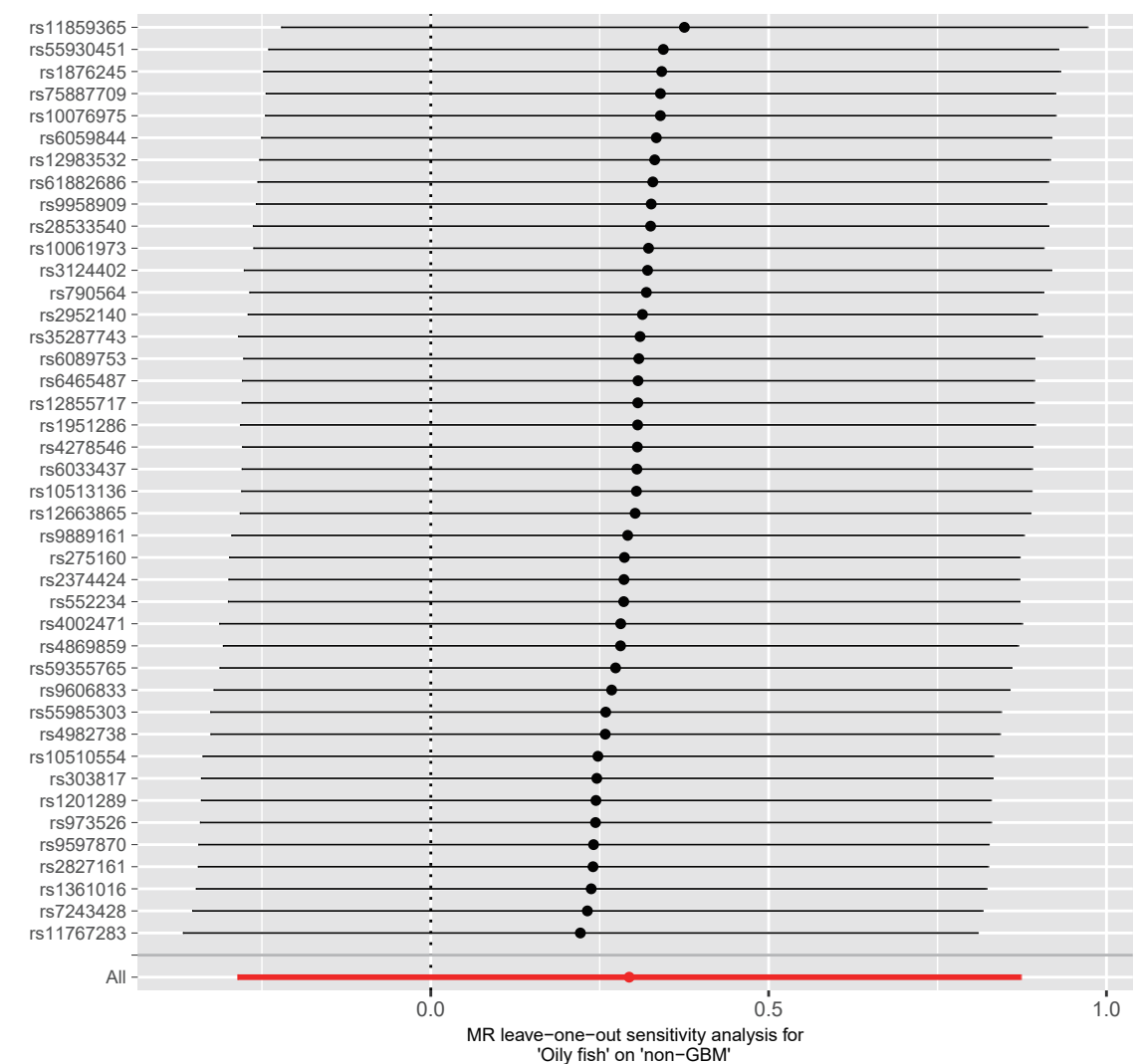

H

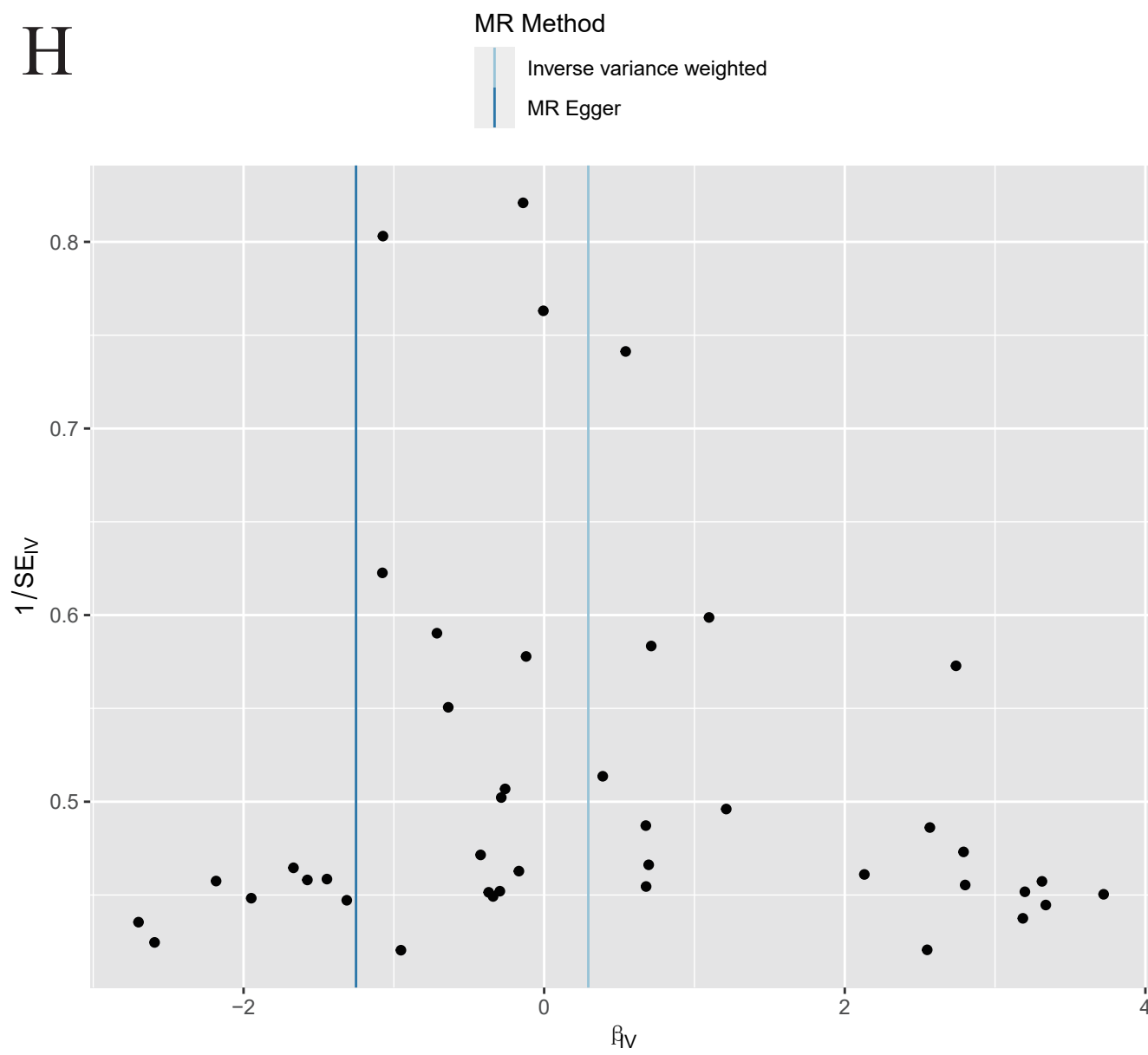

I

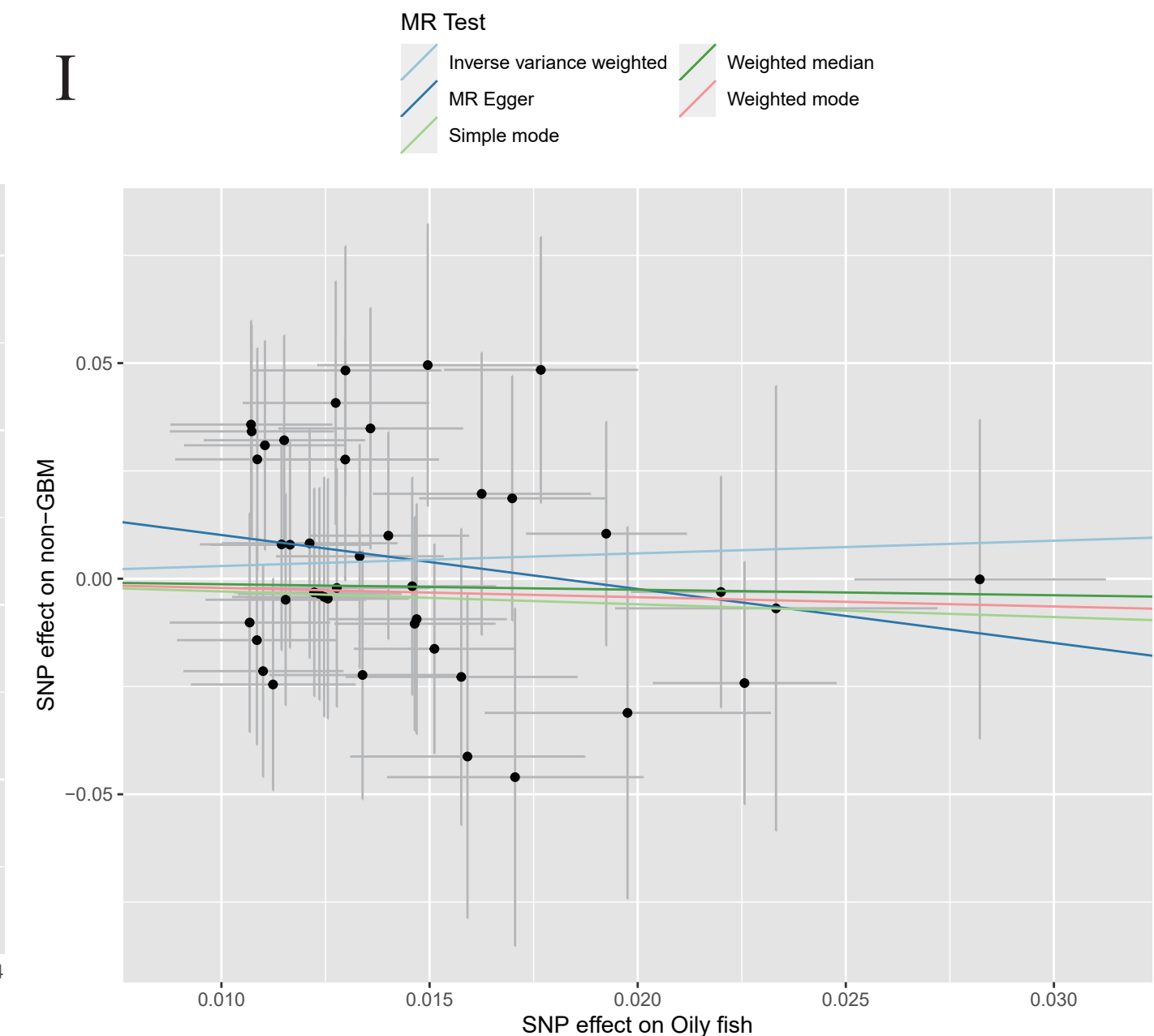

Supplement: Supplementary file 1 [file nutrients-17-00582-s001.zip › nutrients-3462880-supplementary/Sup_17.pdf]

A

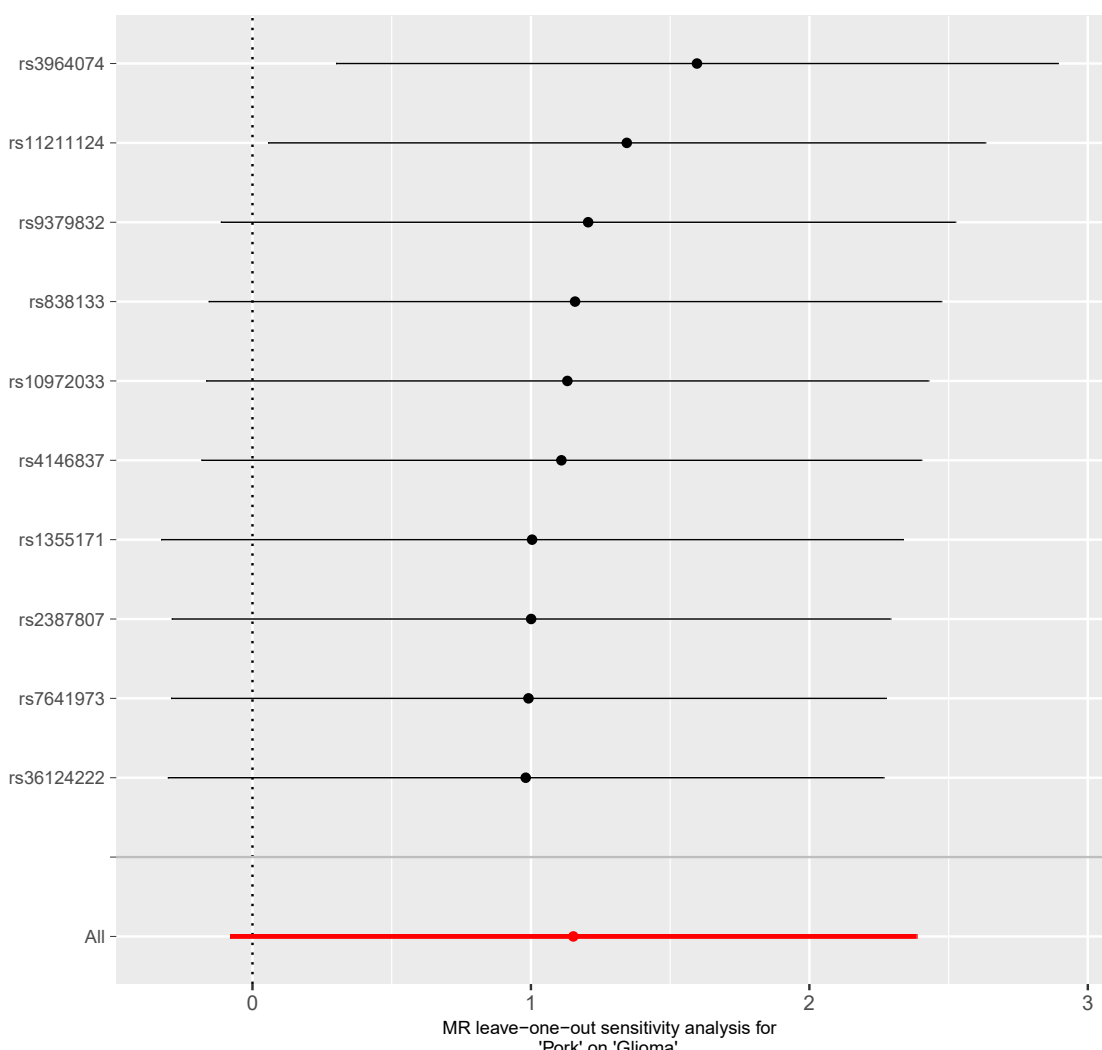

B

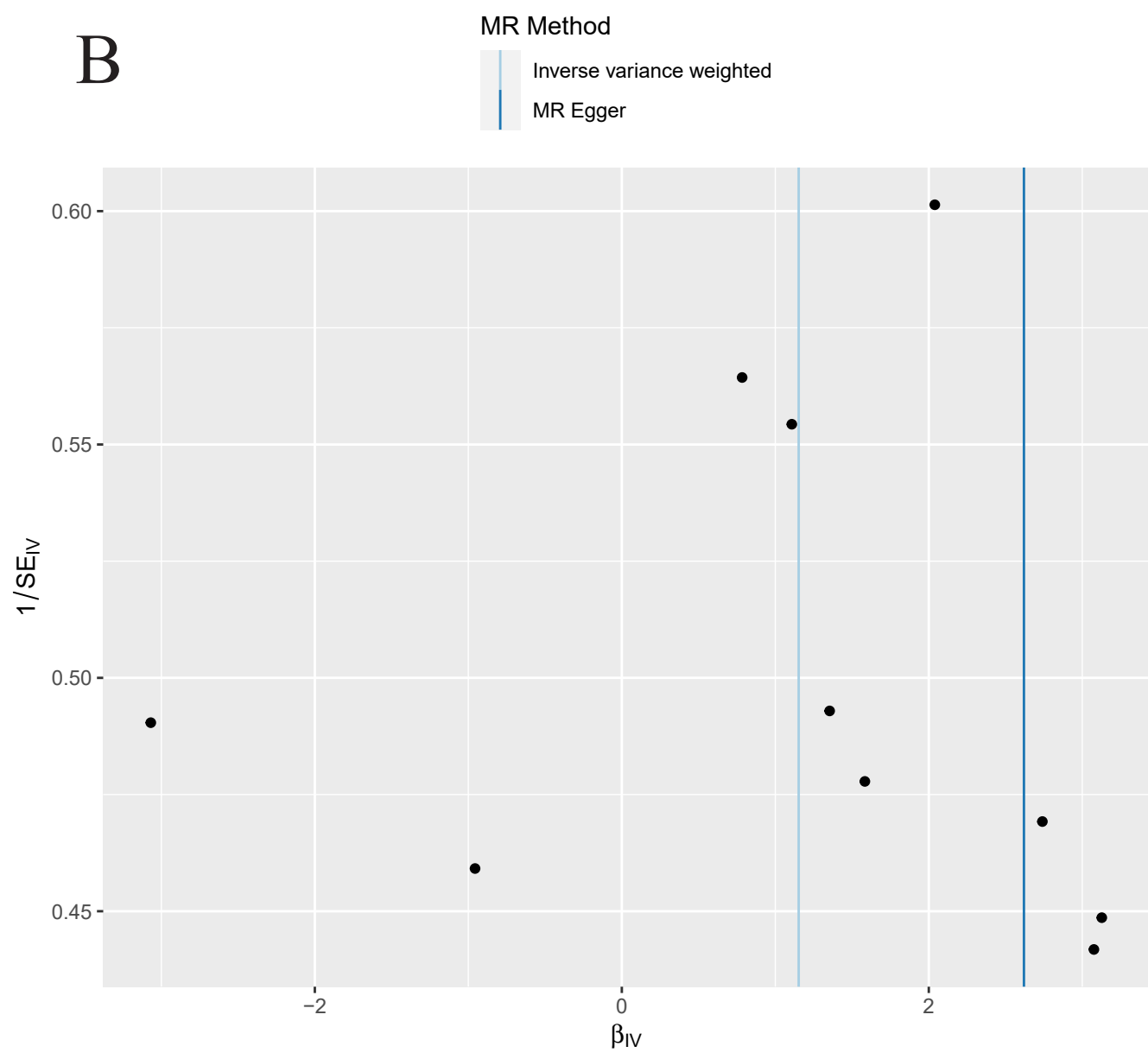

C

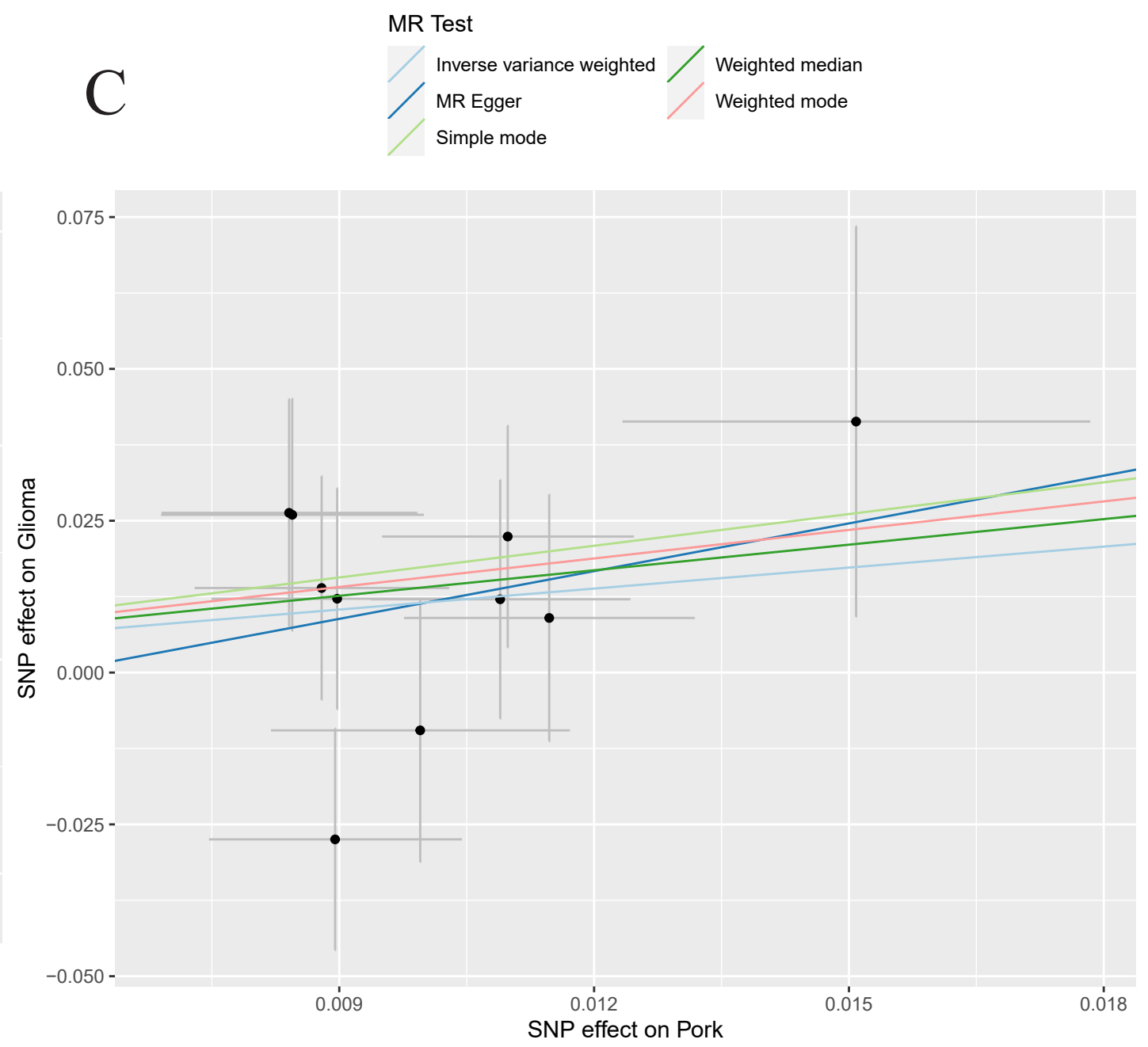

D

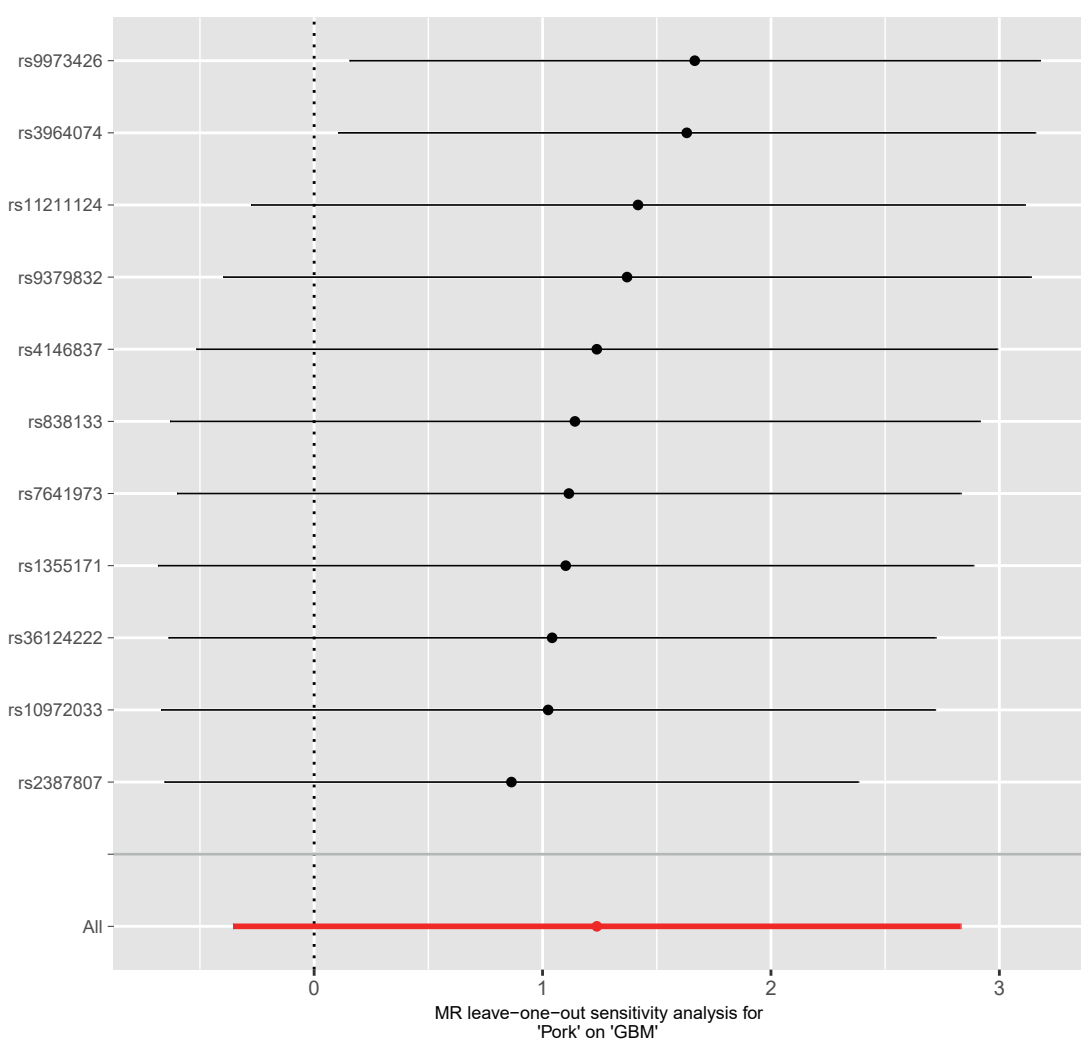

E

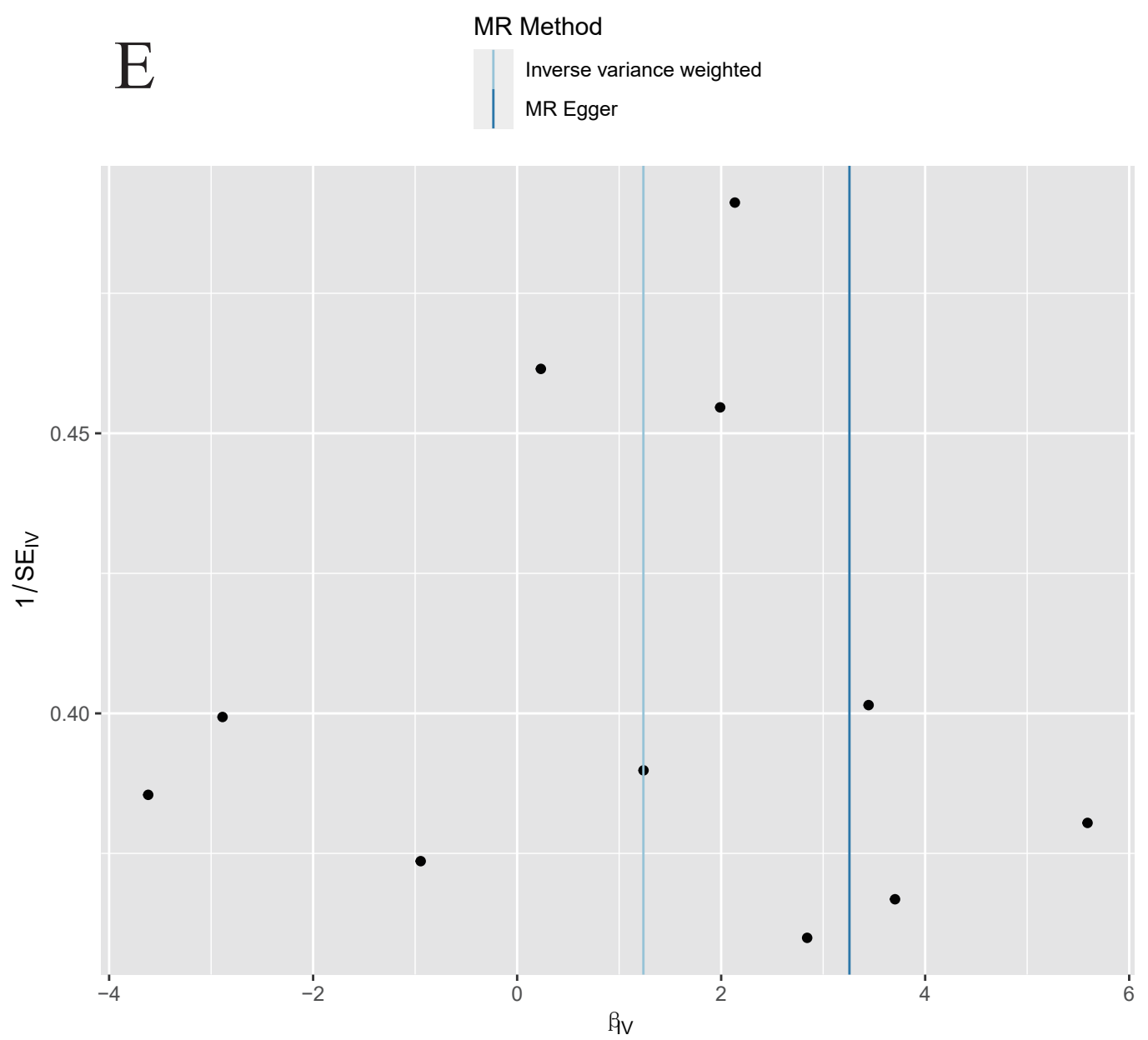

F

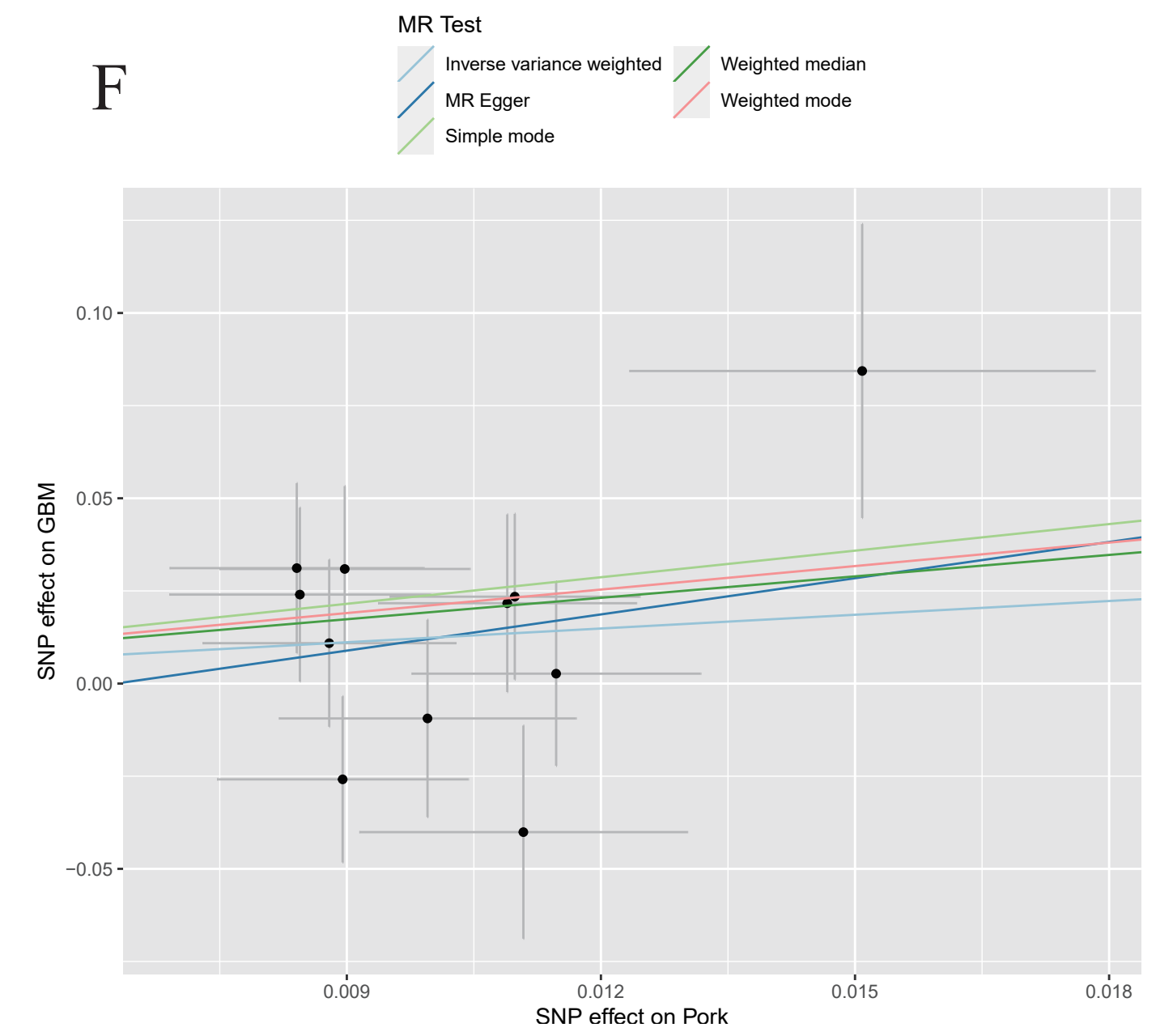

G

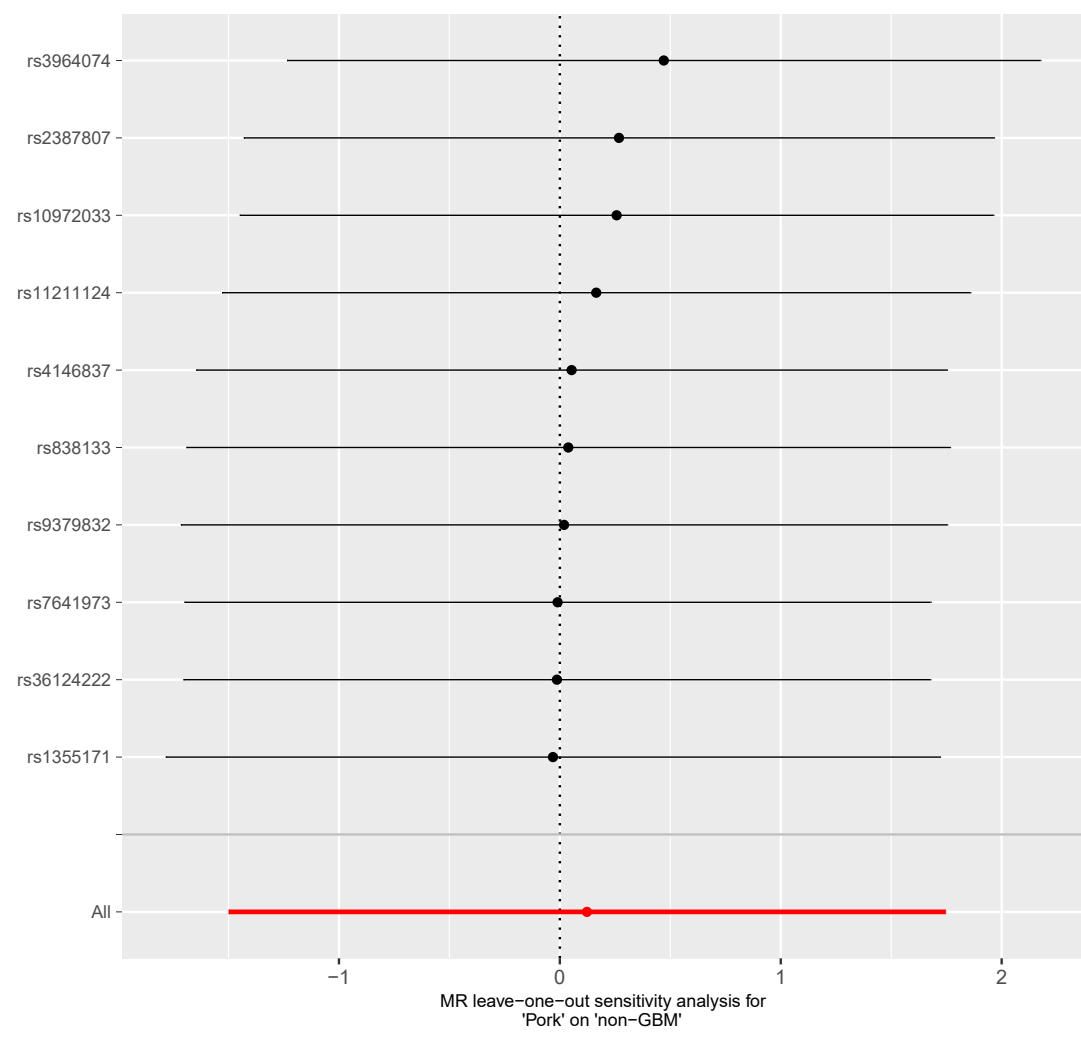

H

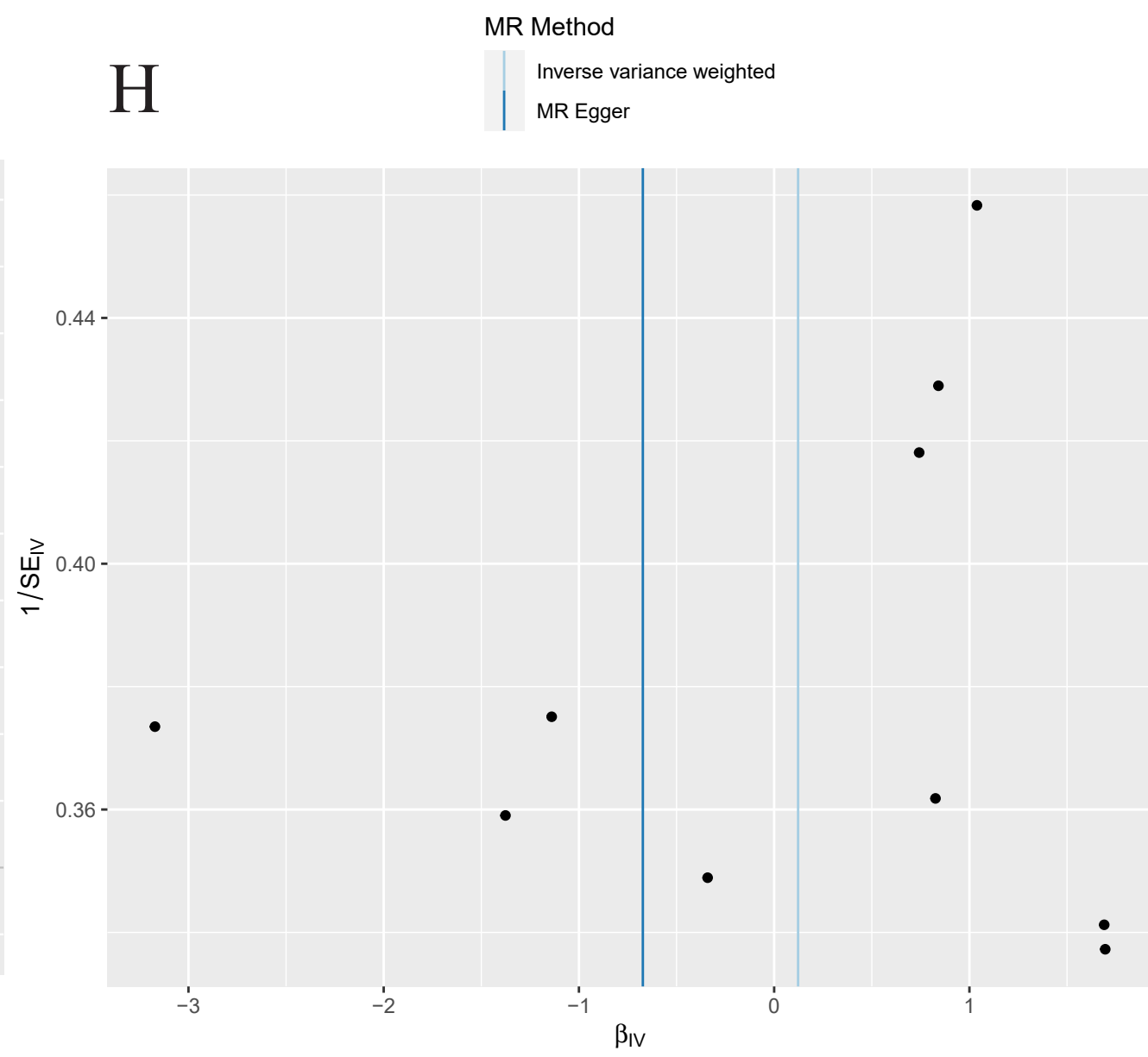

I

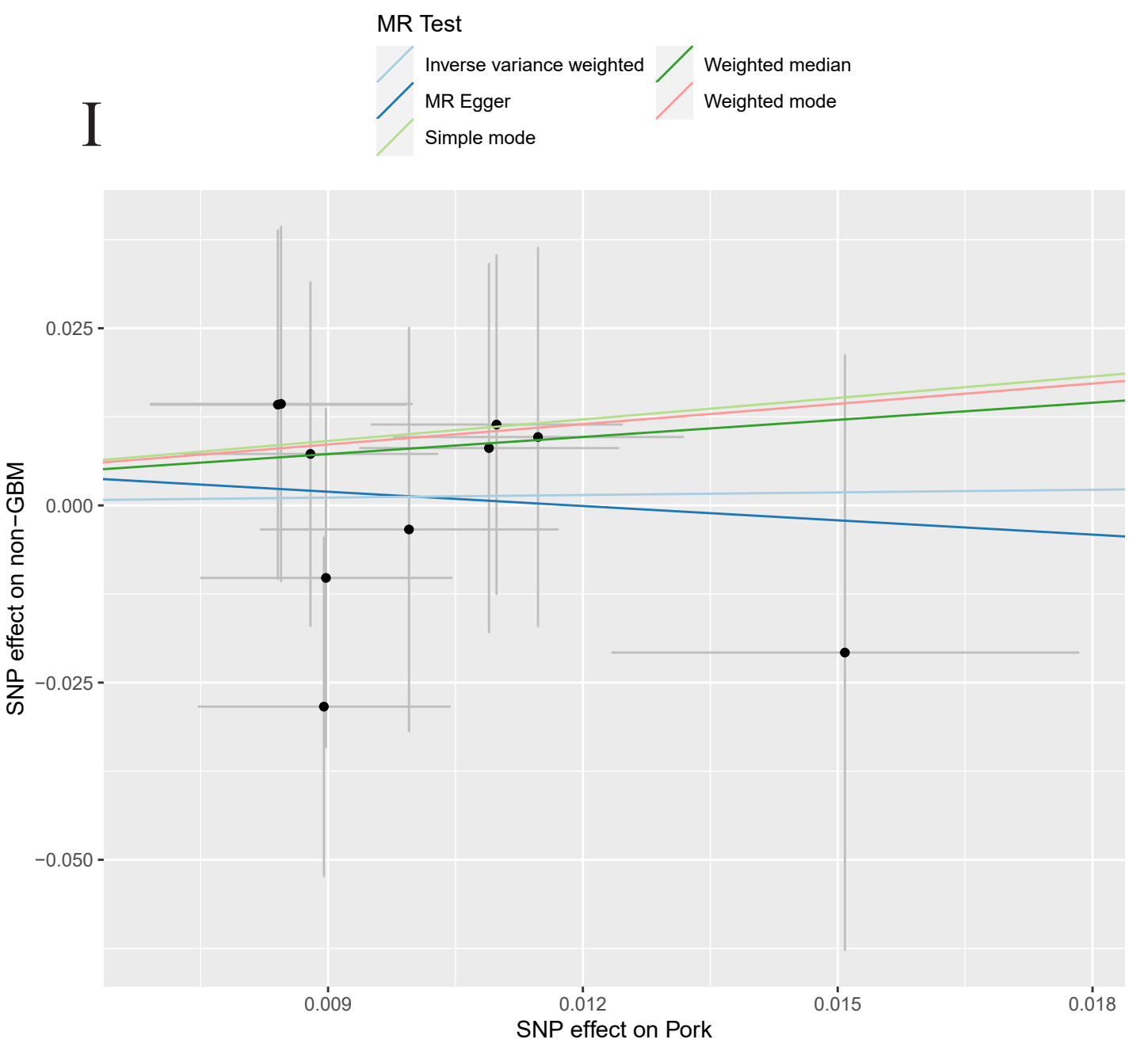

Supplement: Supplementary file 1 [file nutrients-17-00582-s001.zip › nutrients-3462880-supplementary/Sup_18.pdf]

A

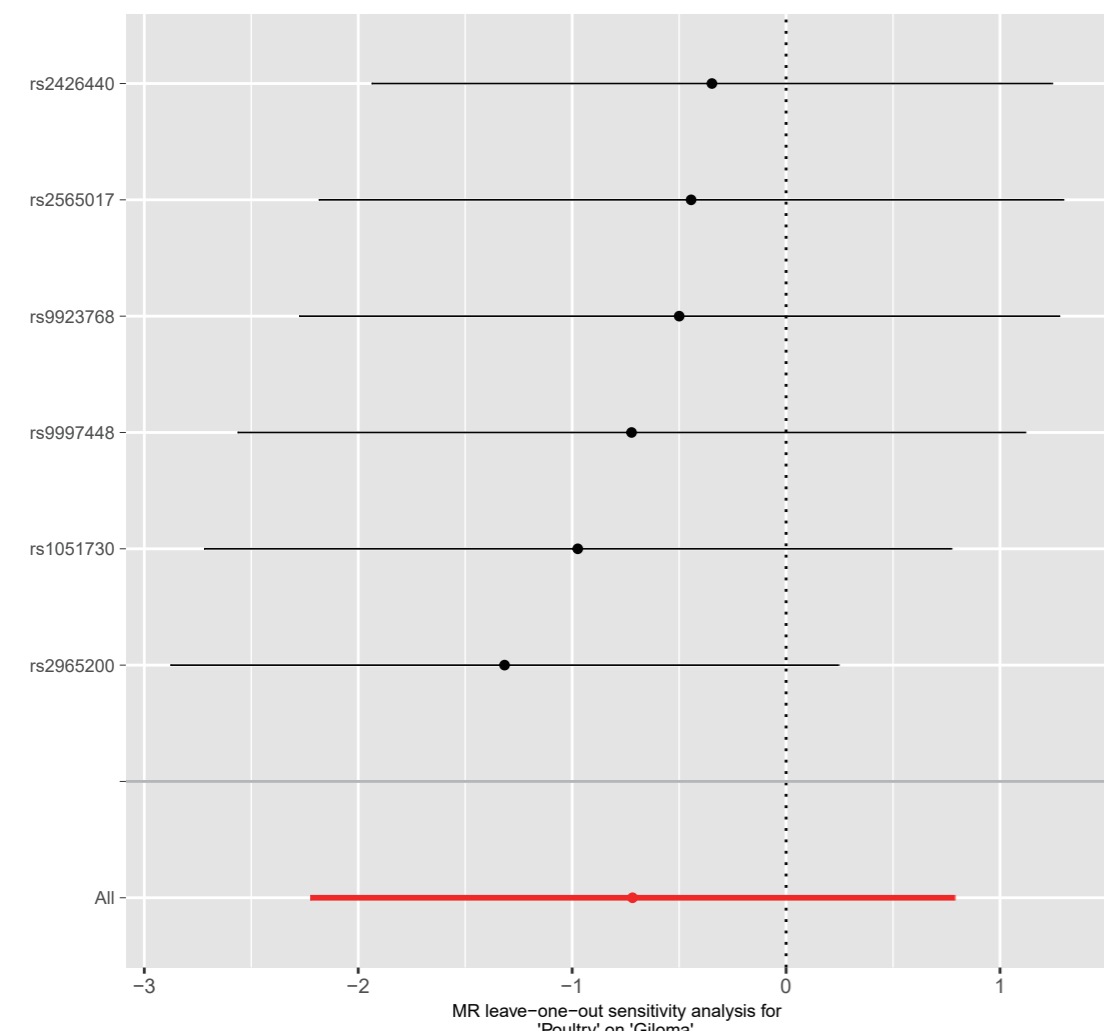

B

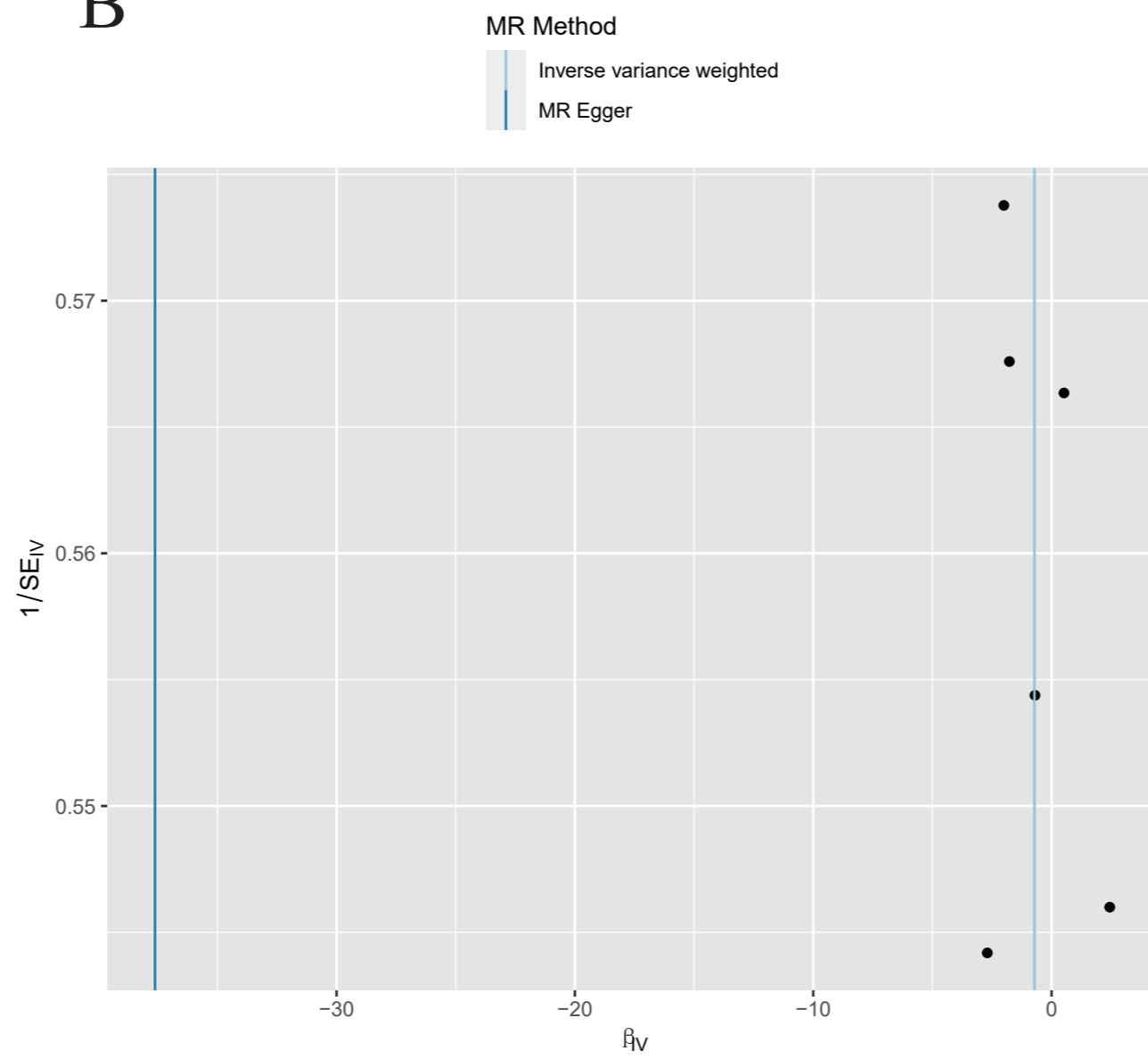

C

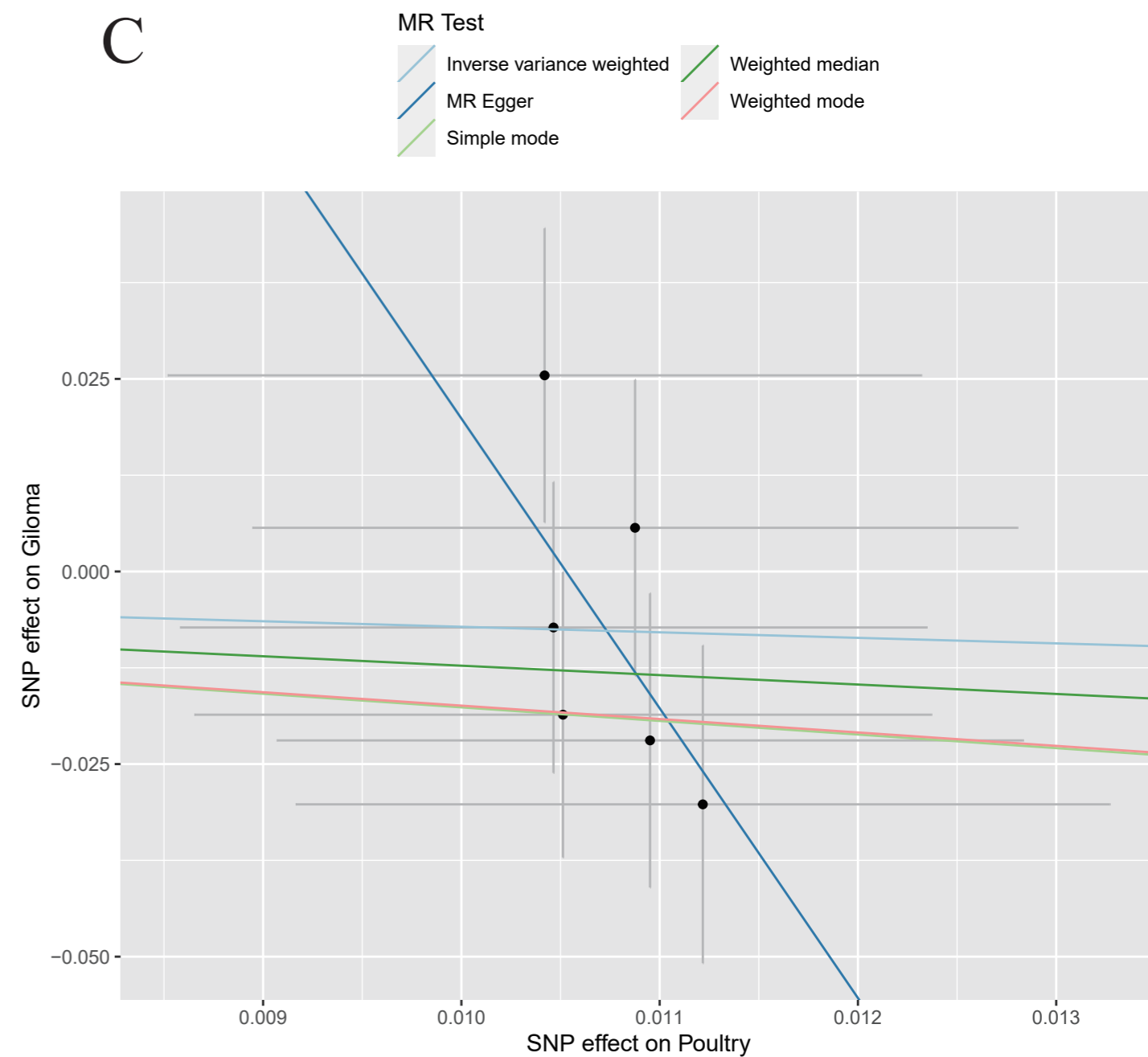

D

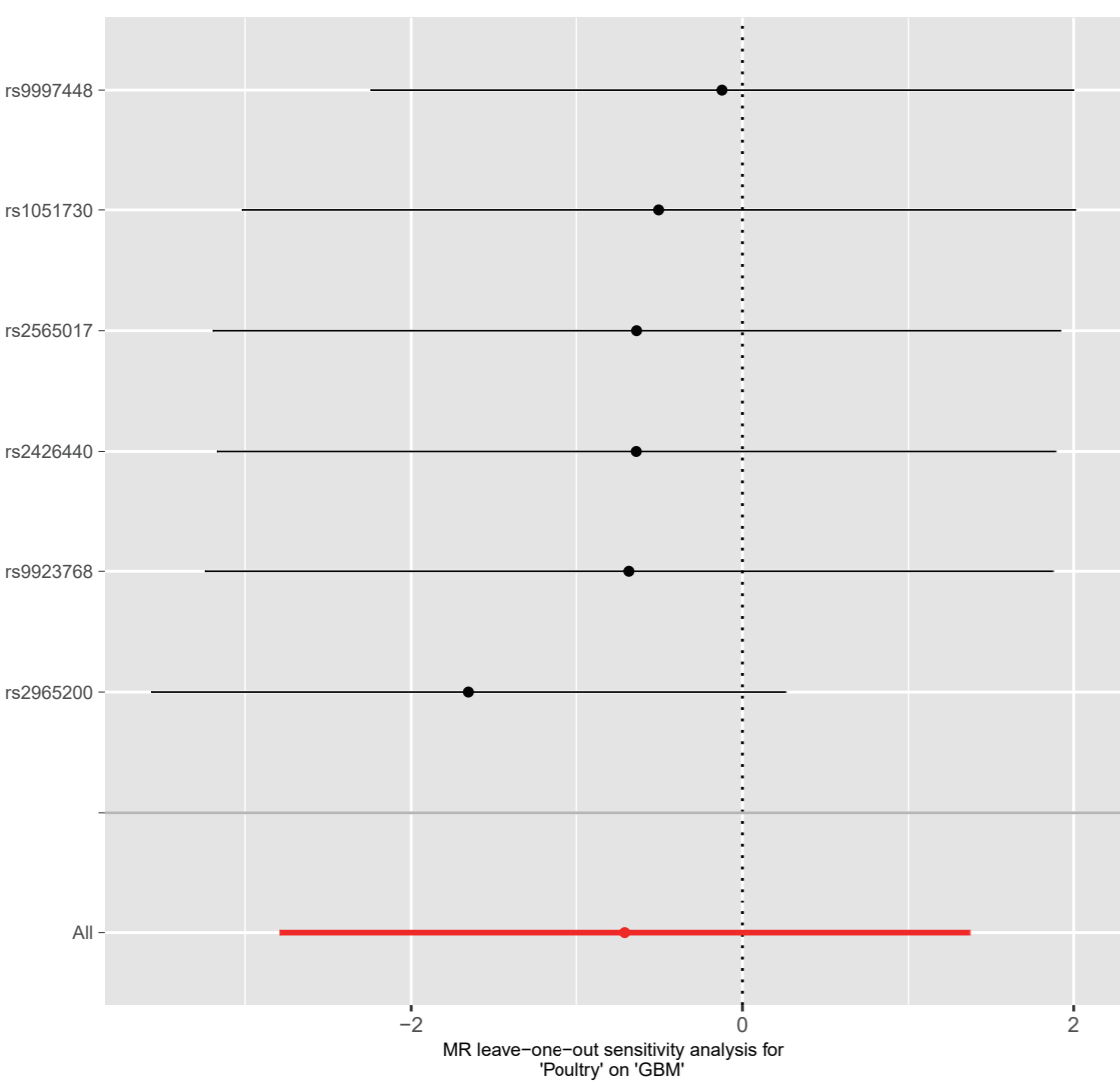

E

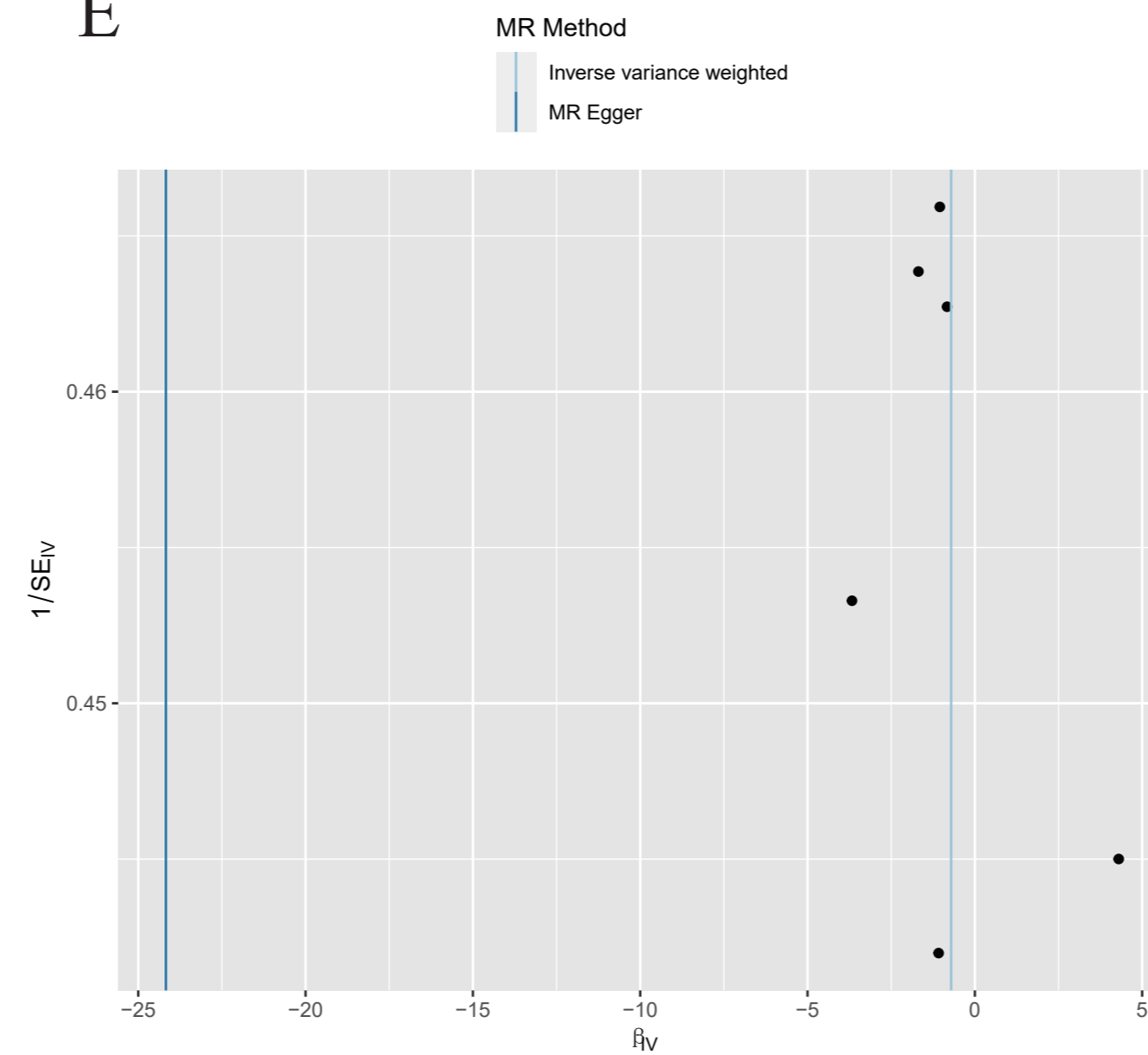

F

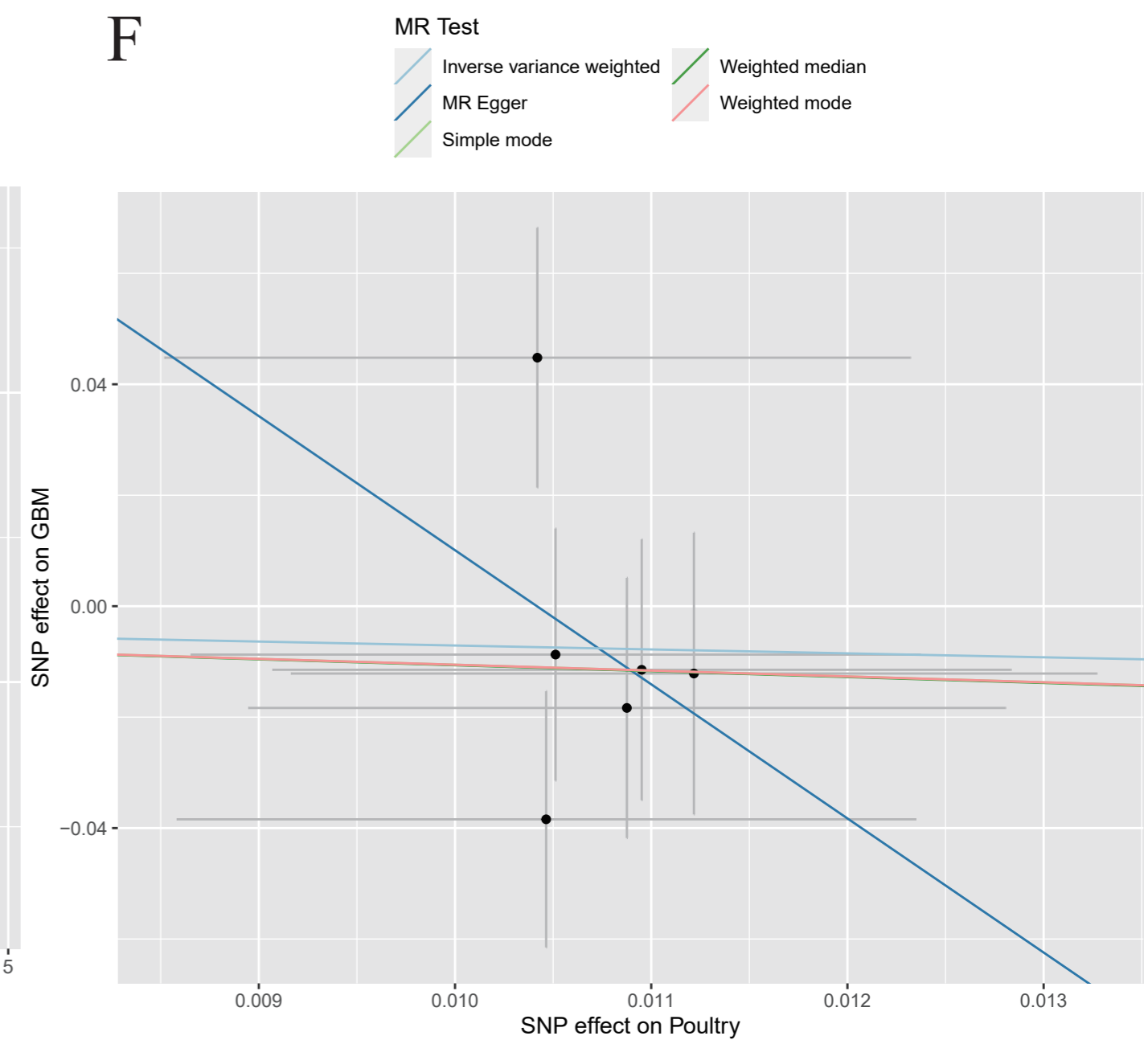

G

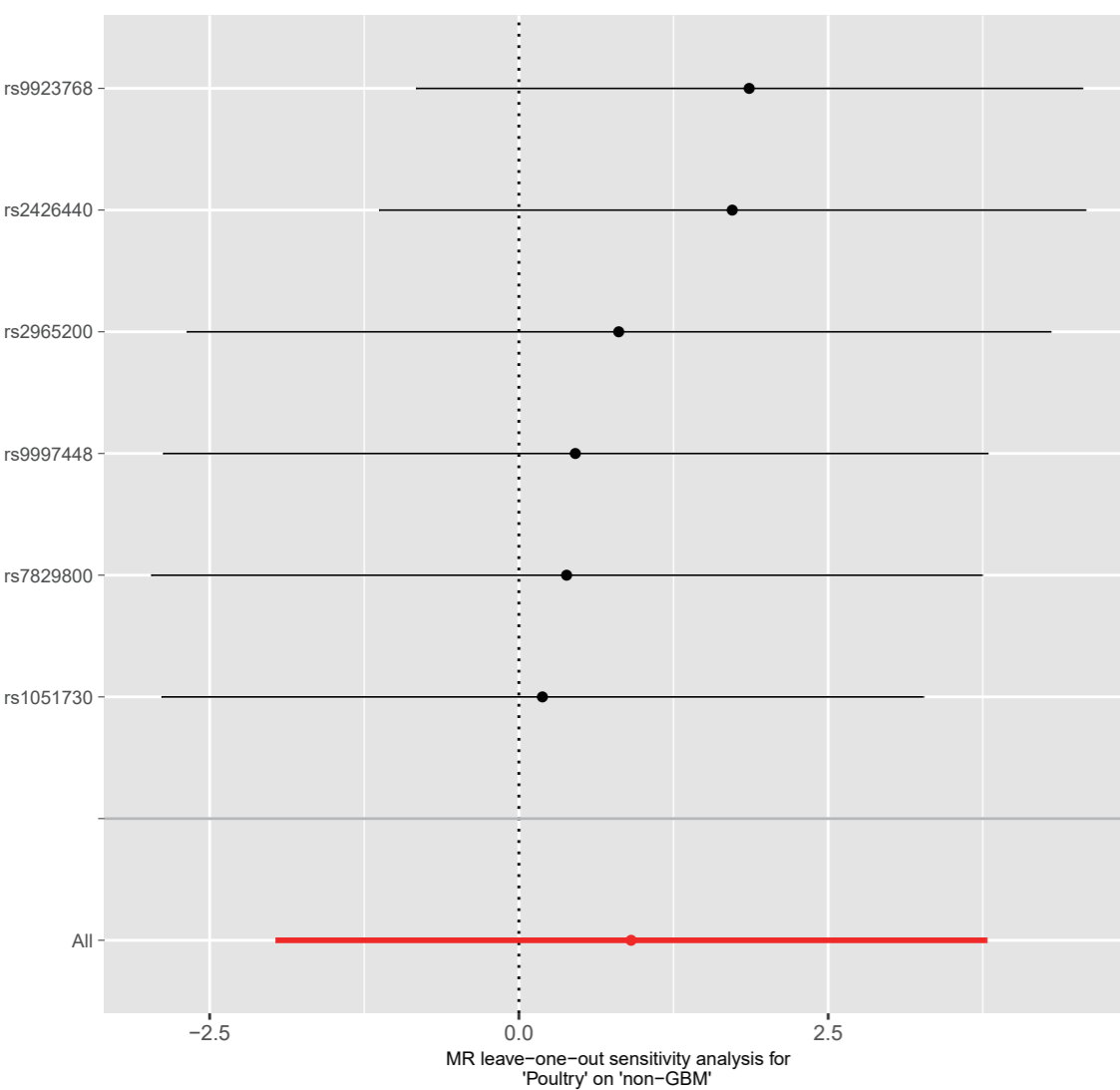

H

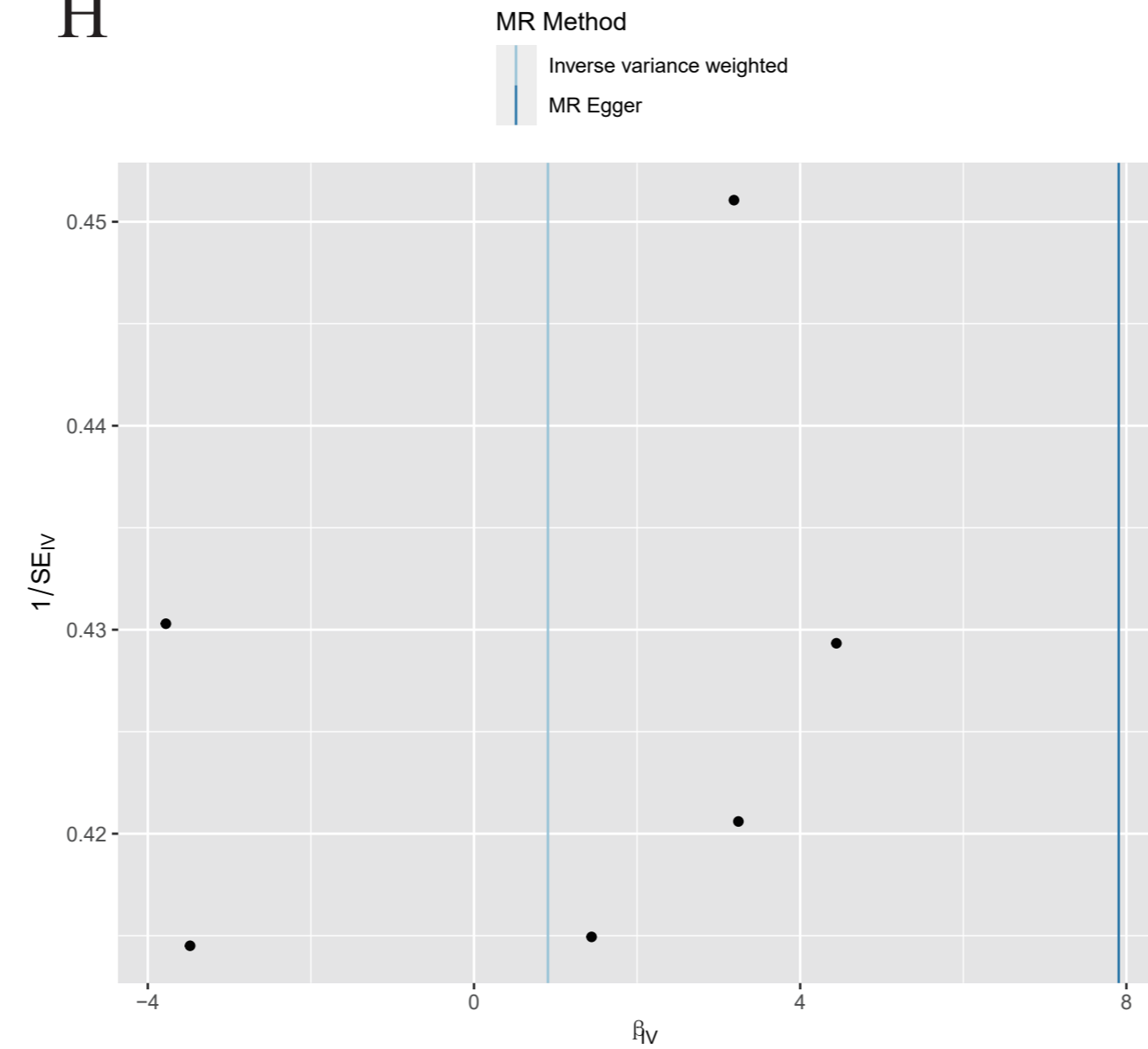

I

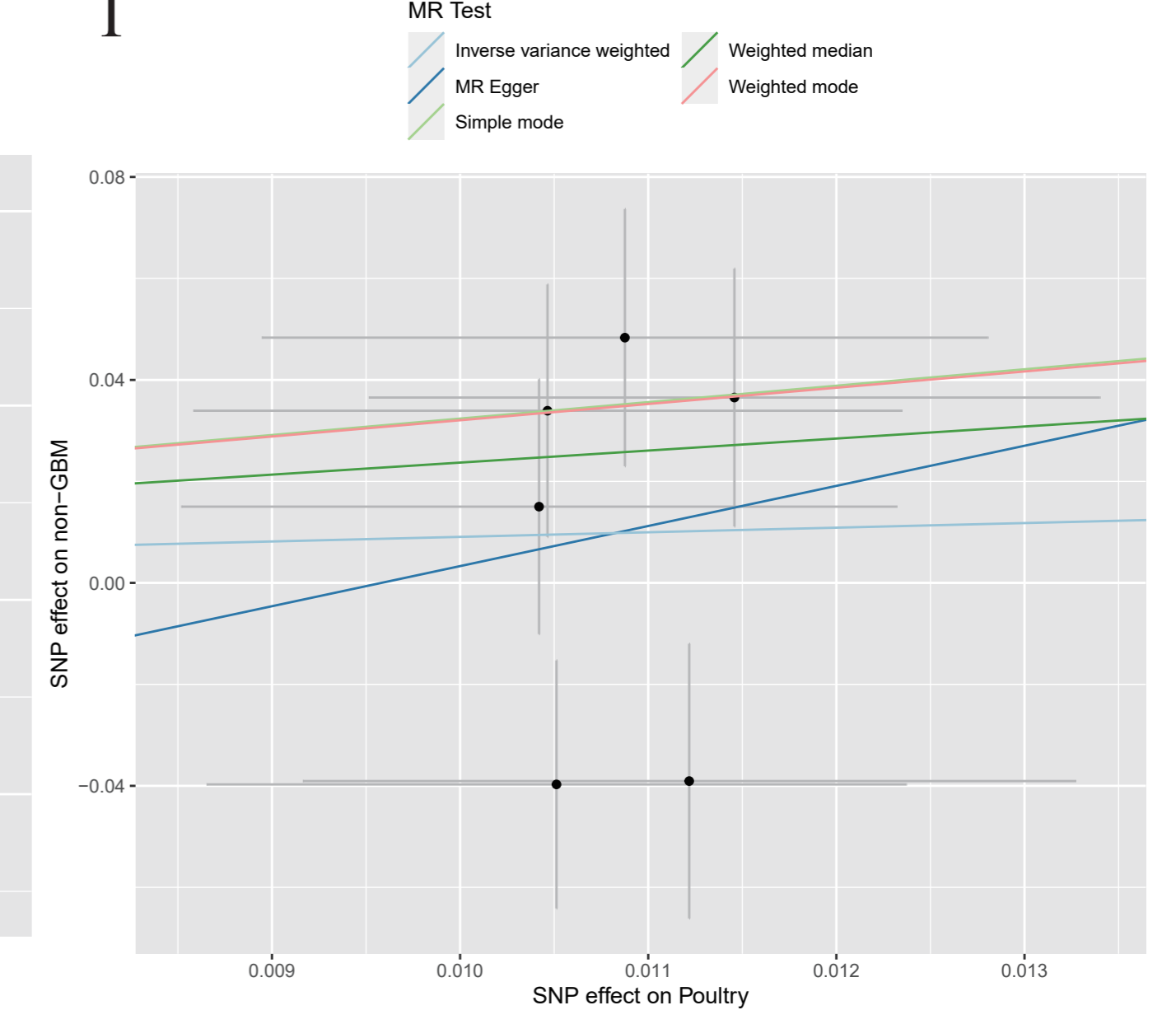

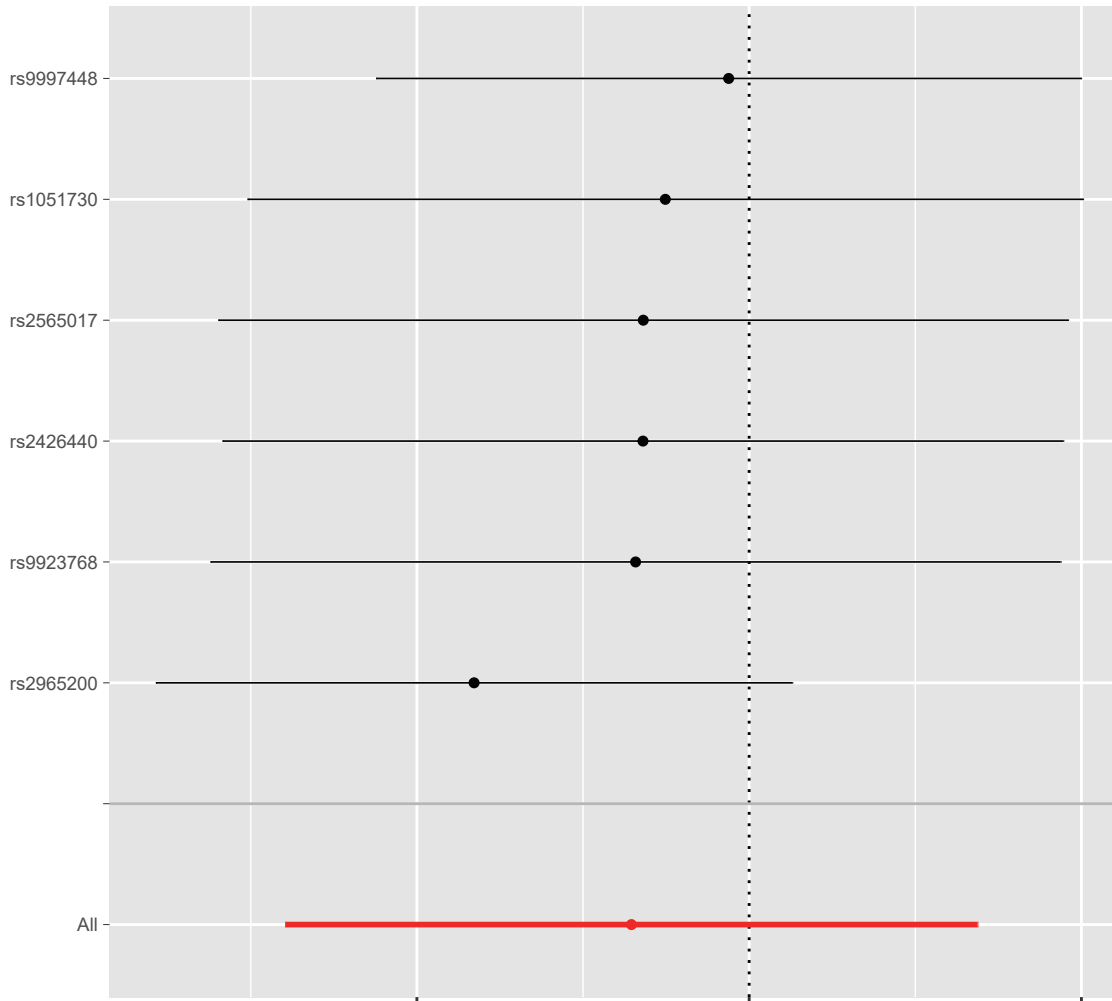

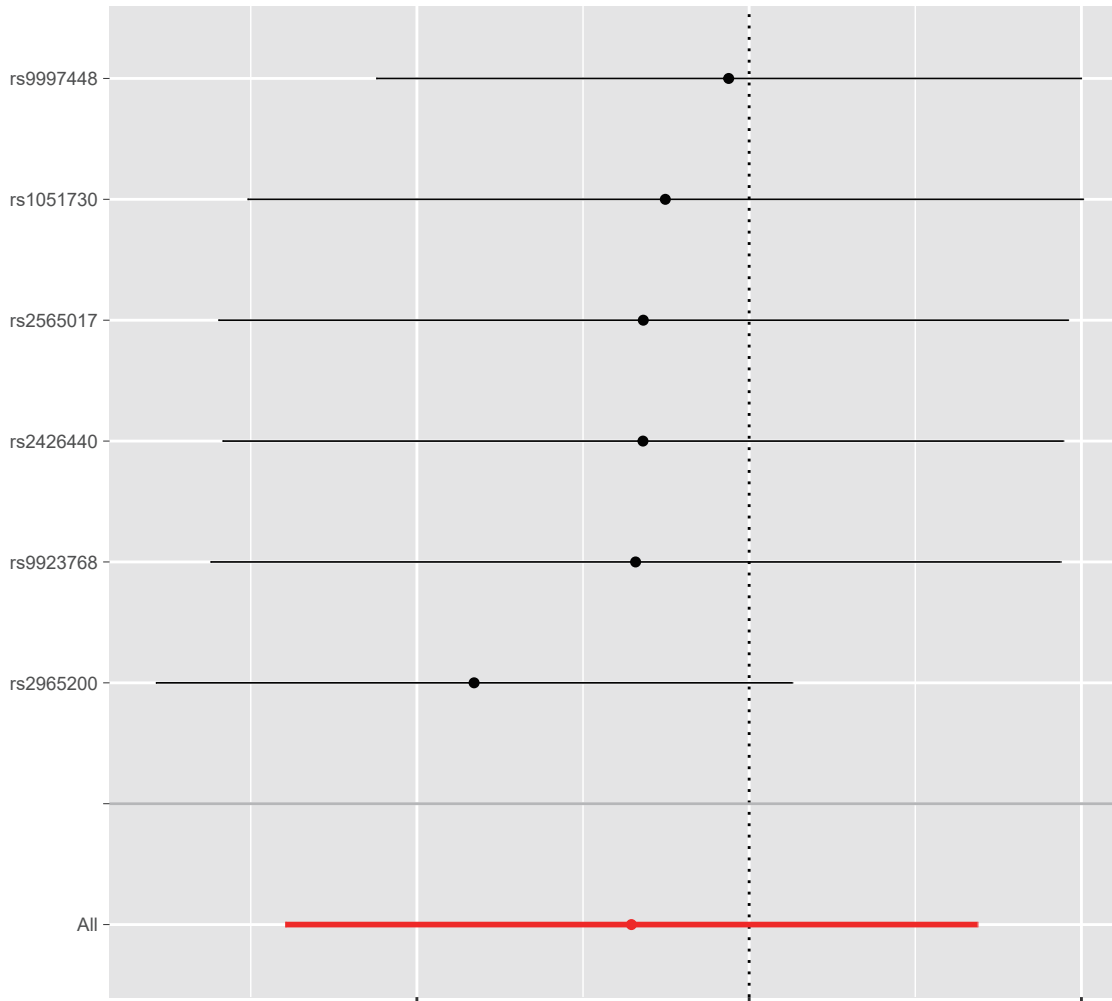

A

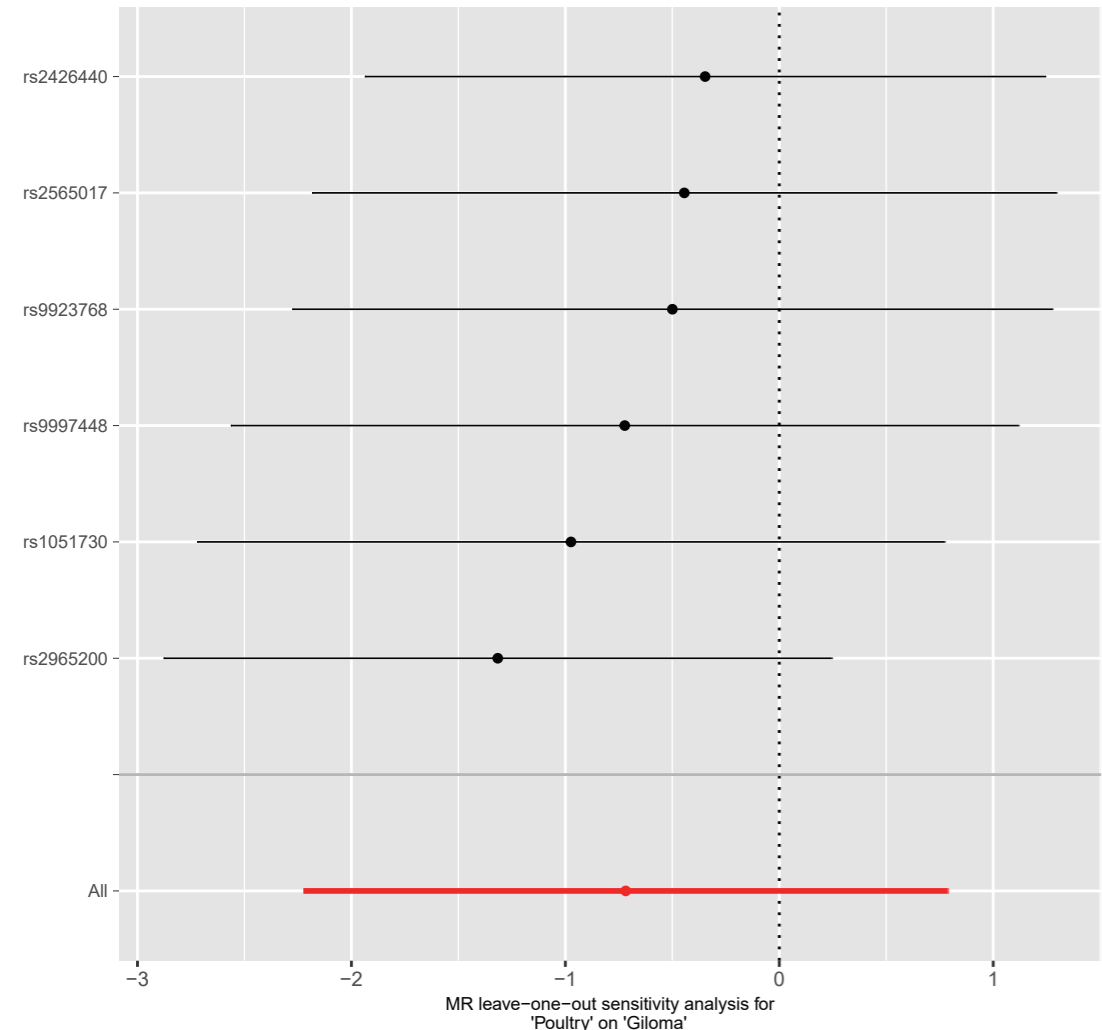

B

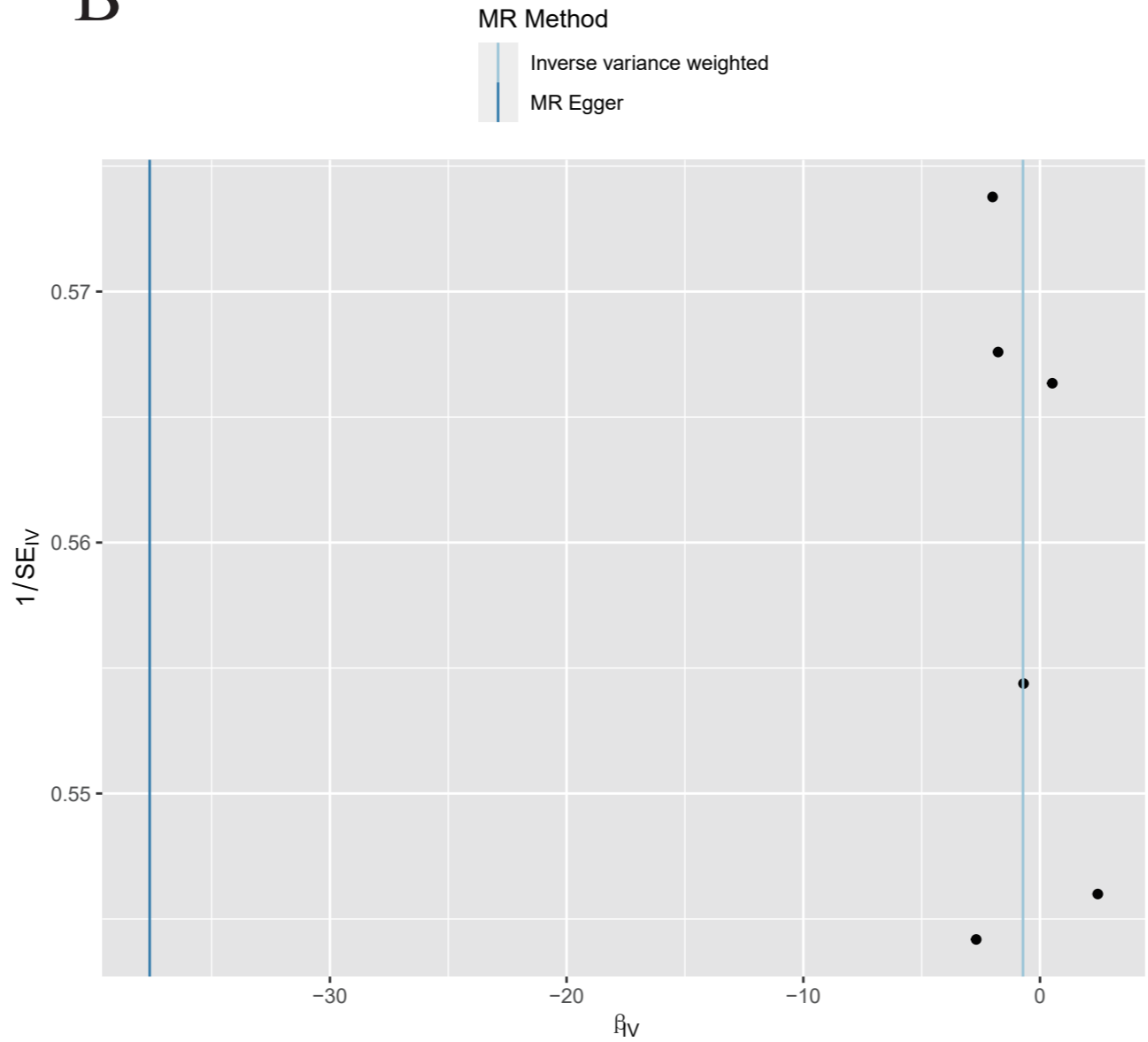

C

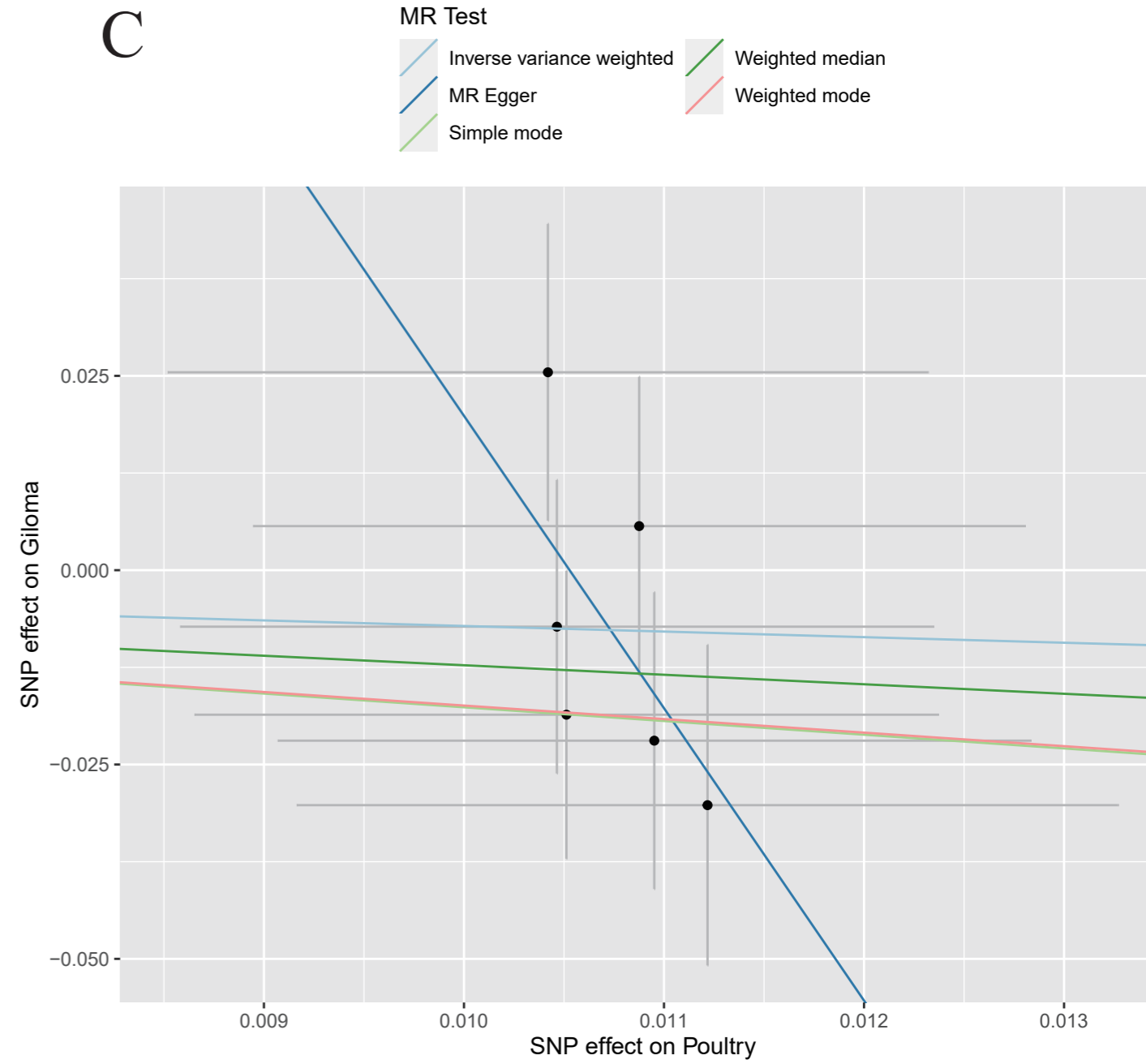

D

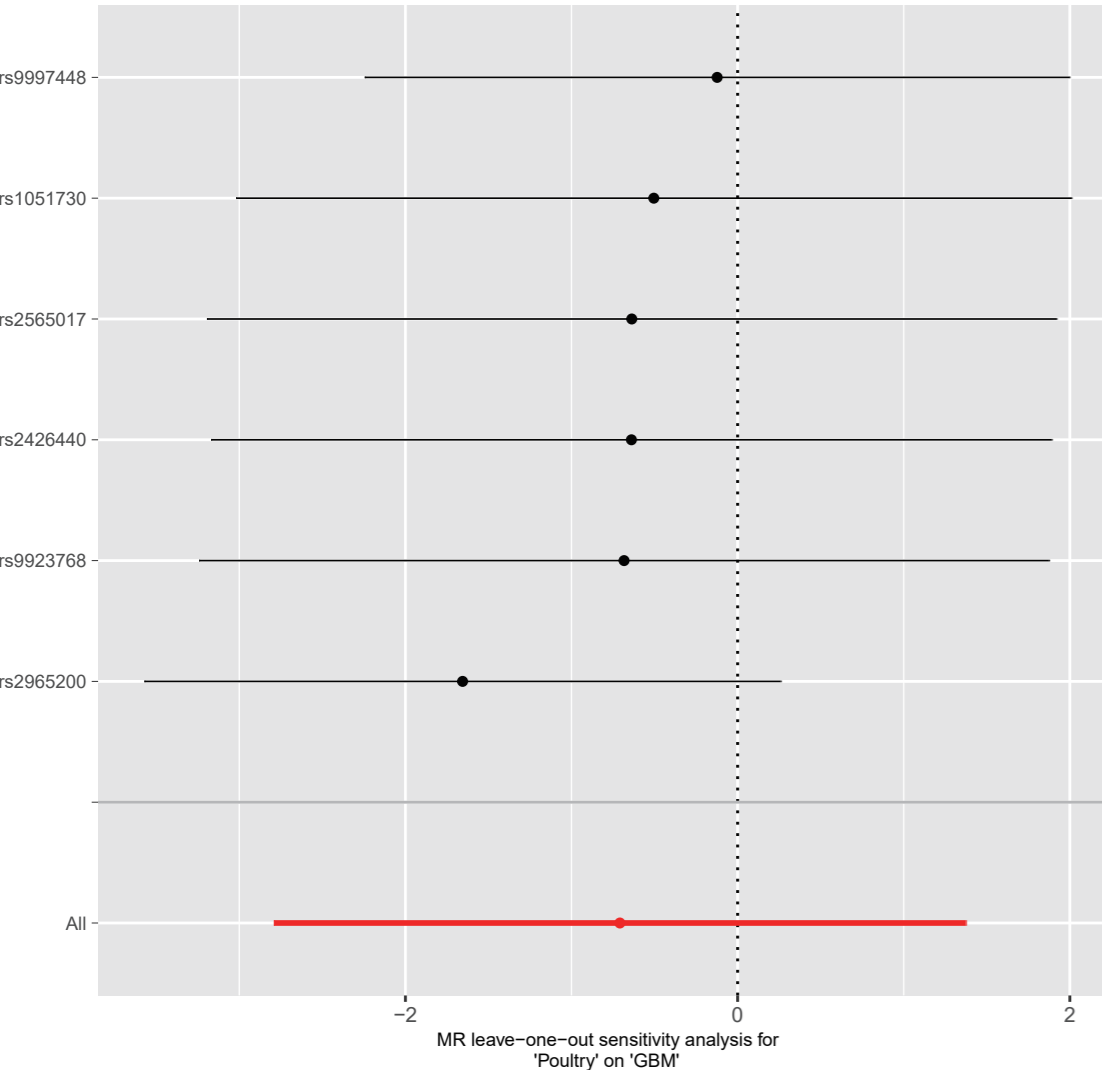

E

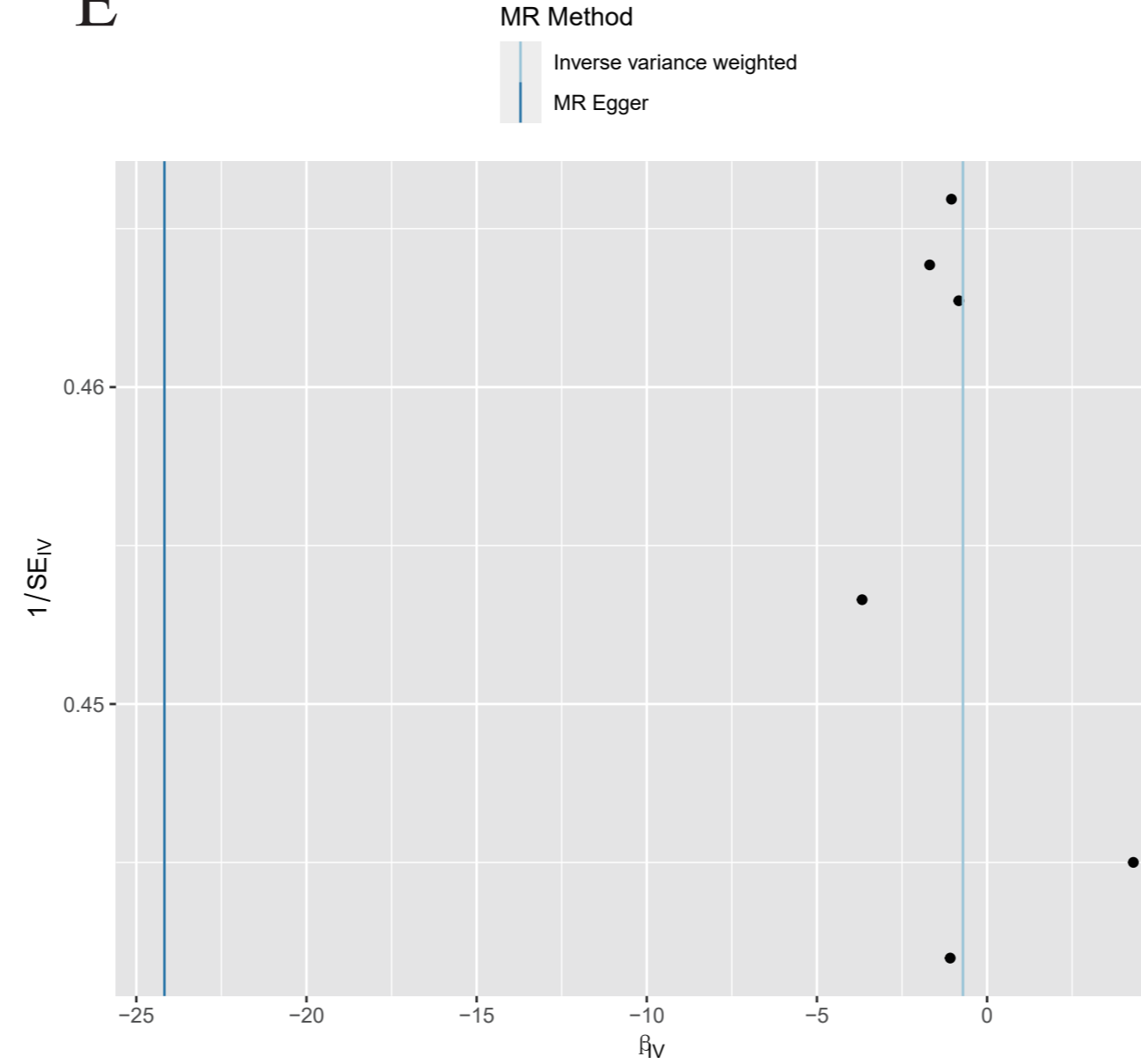

F

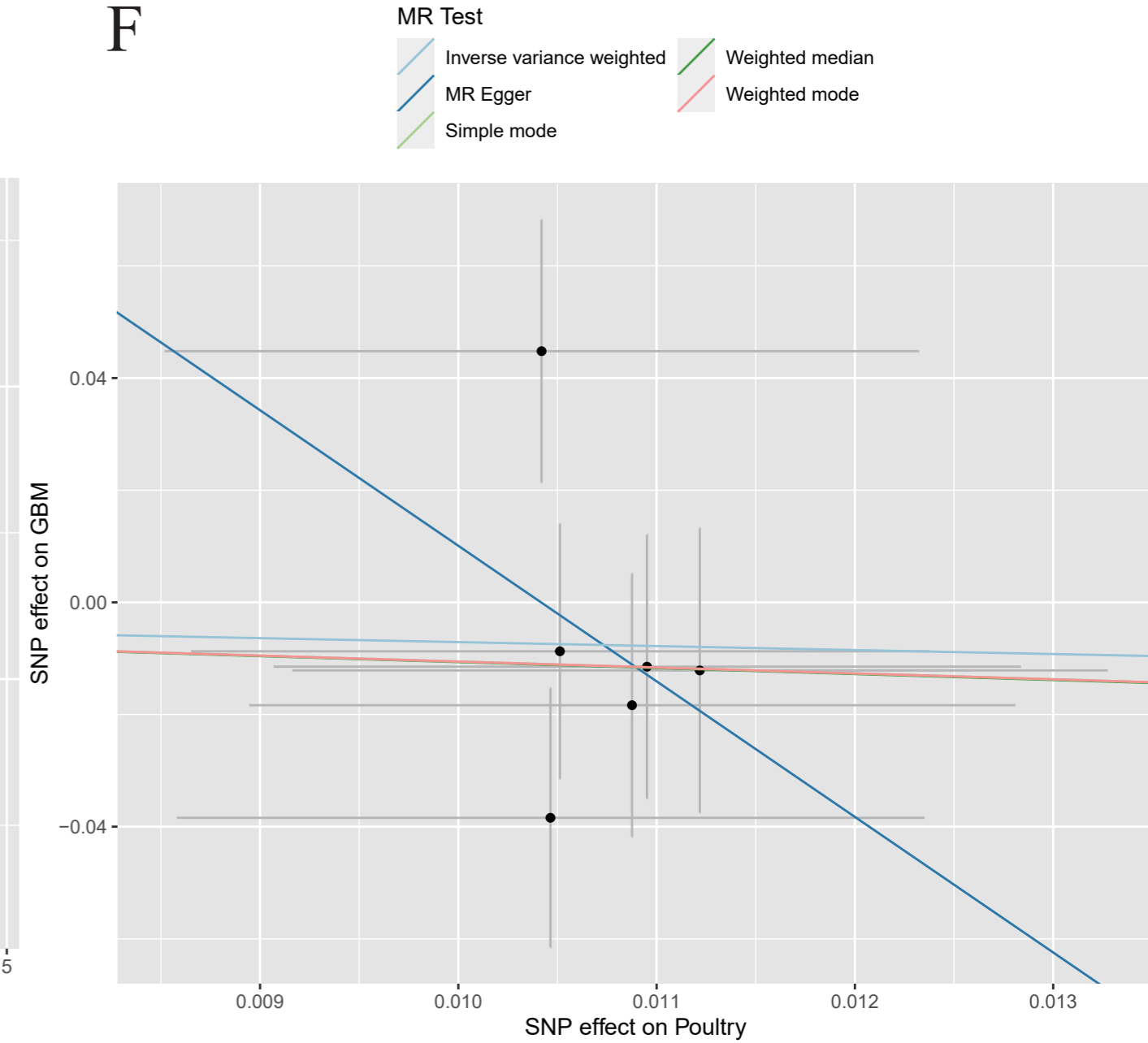

G

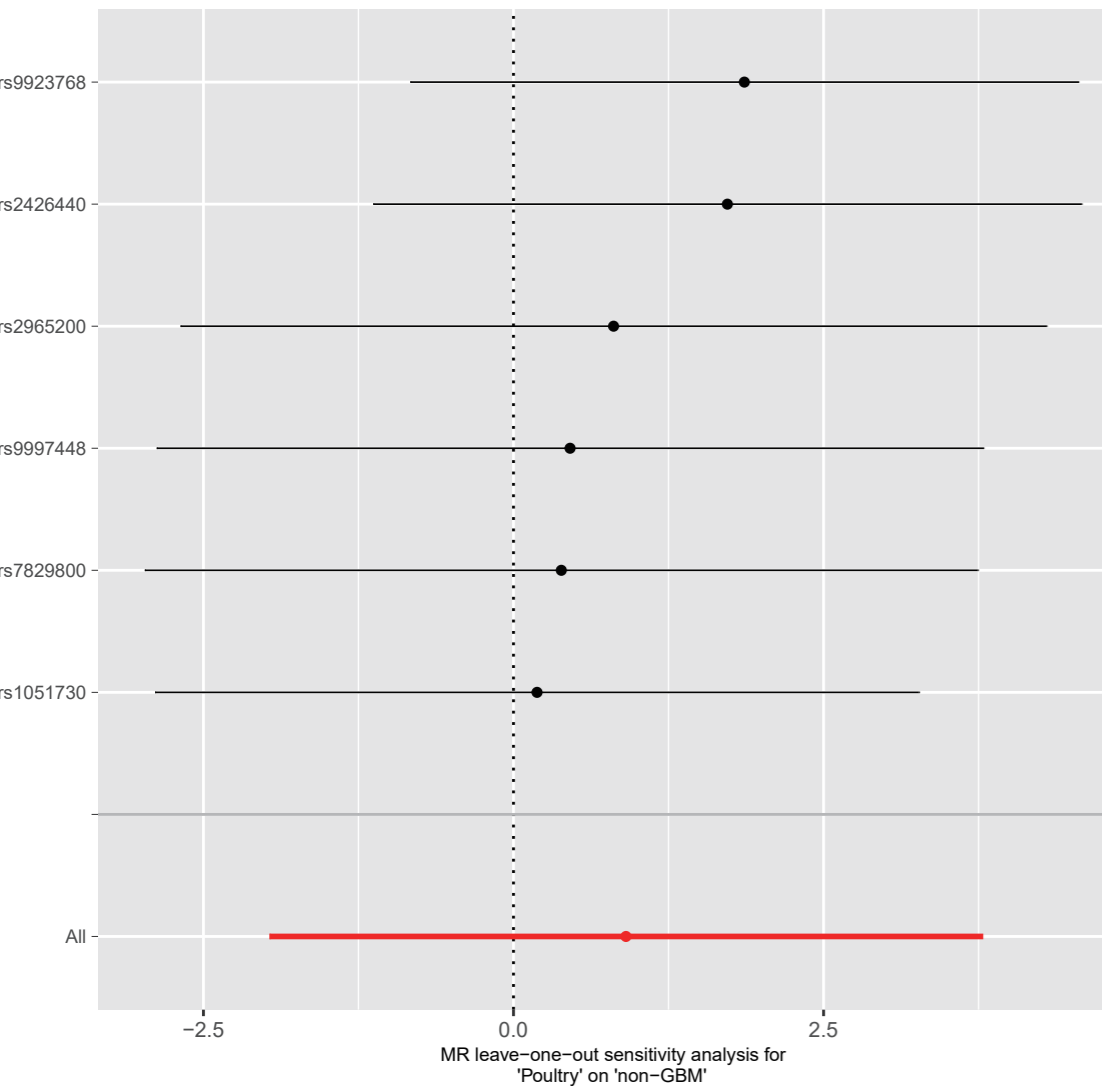

H

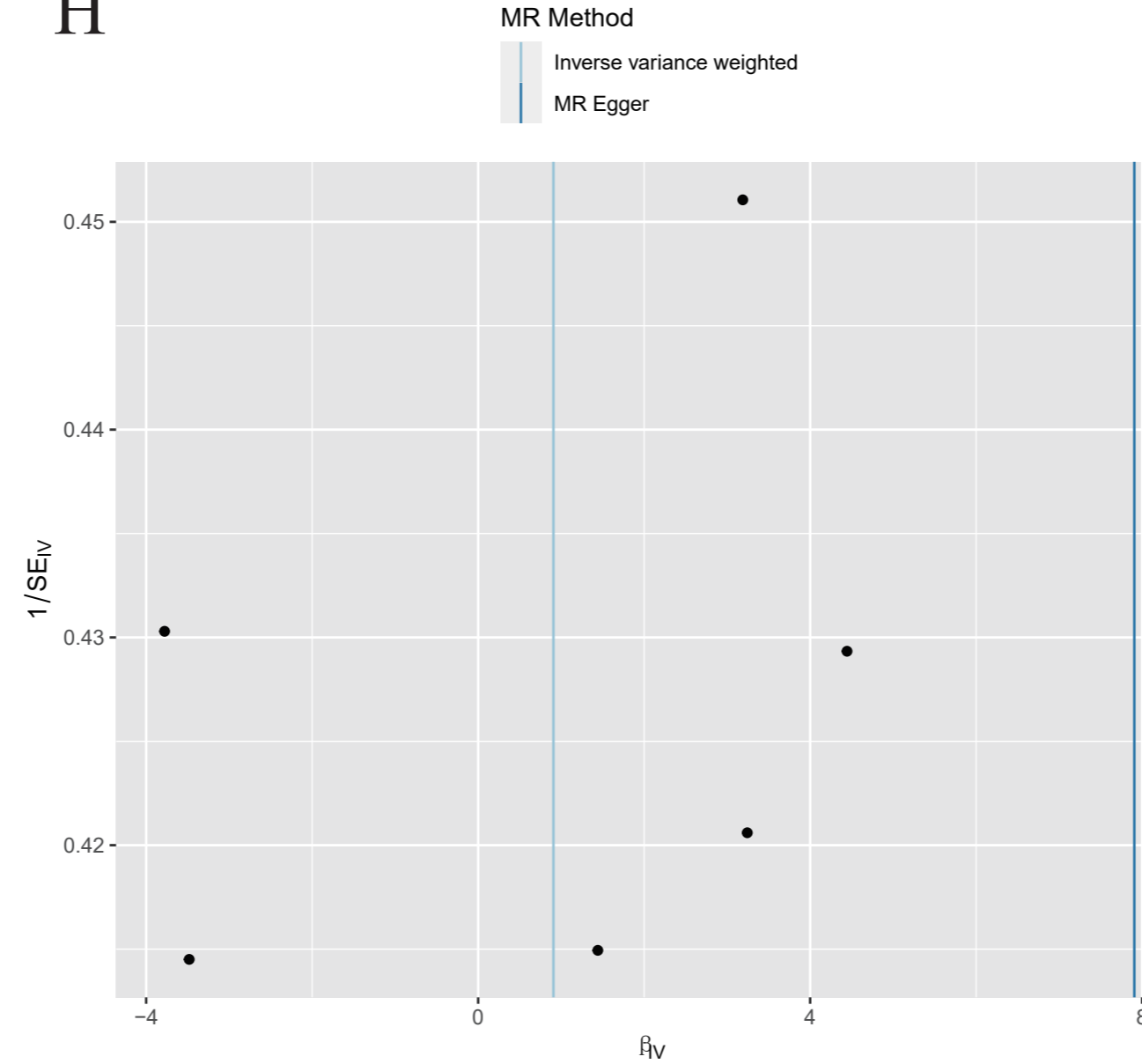

I

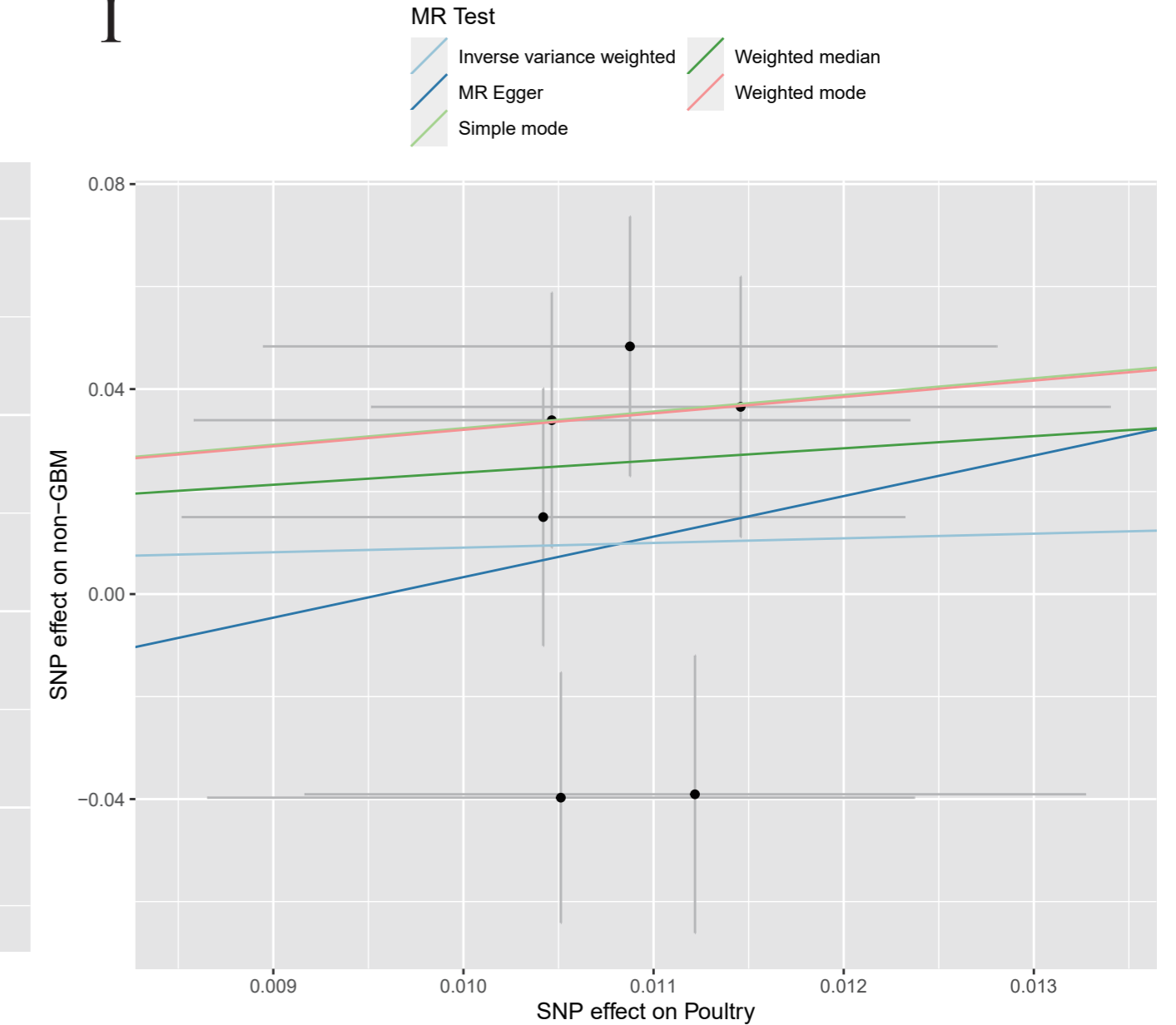

Supplement: Supplementary file 1 [file nutrients-17-00582-s001.zip › nutrients-3462880-supplementary/Sup_19.pdf]

A

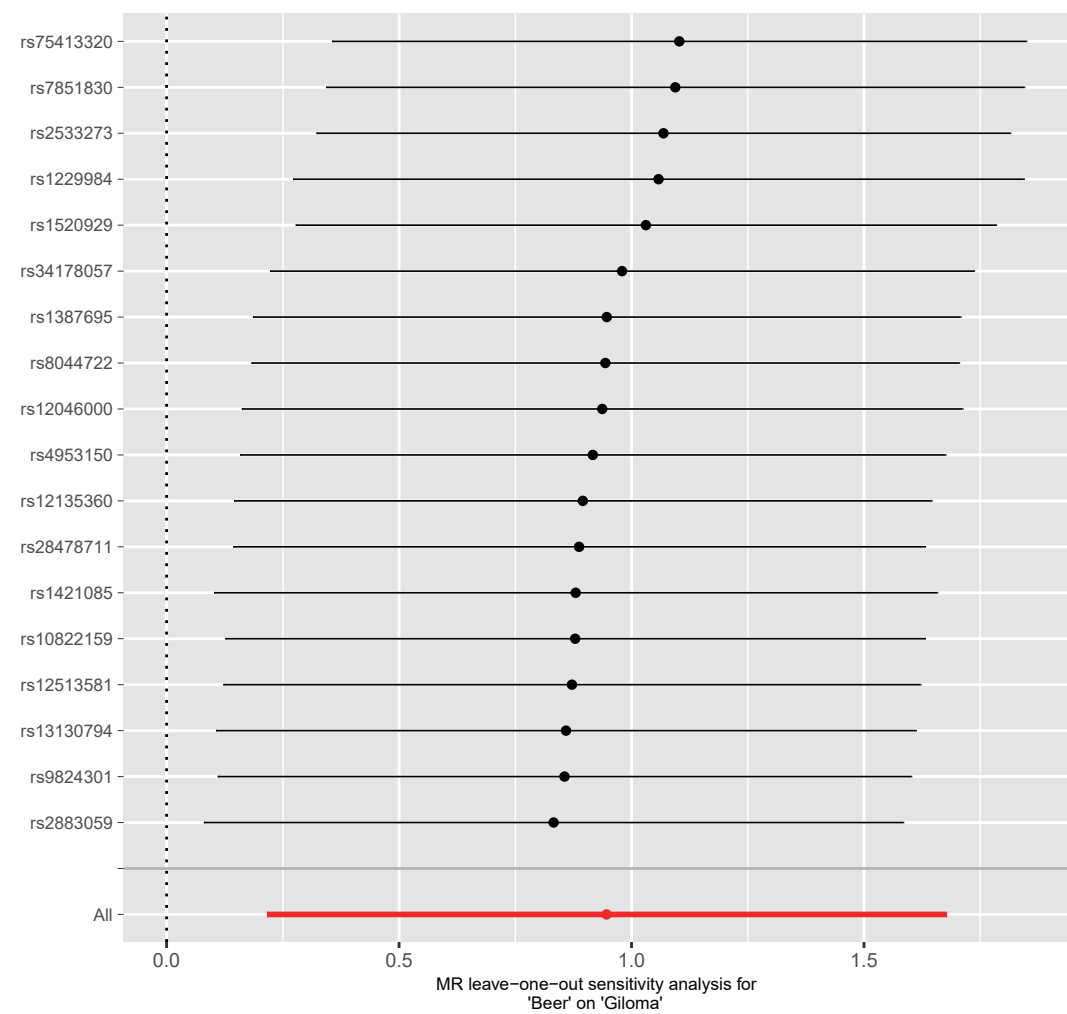

B

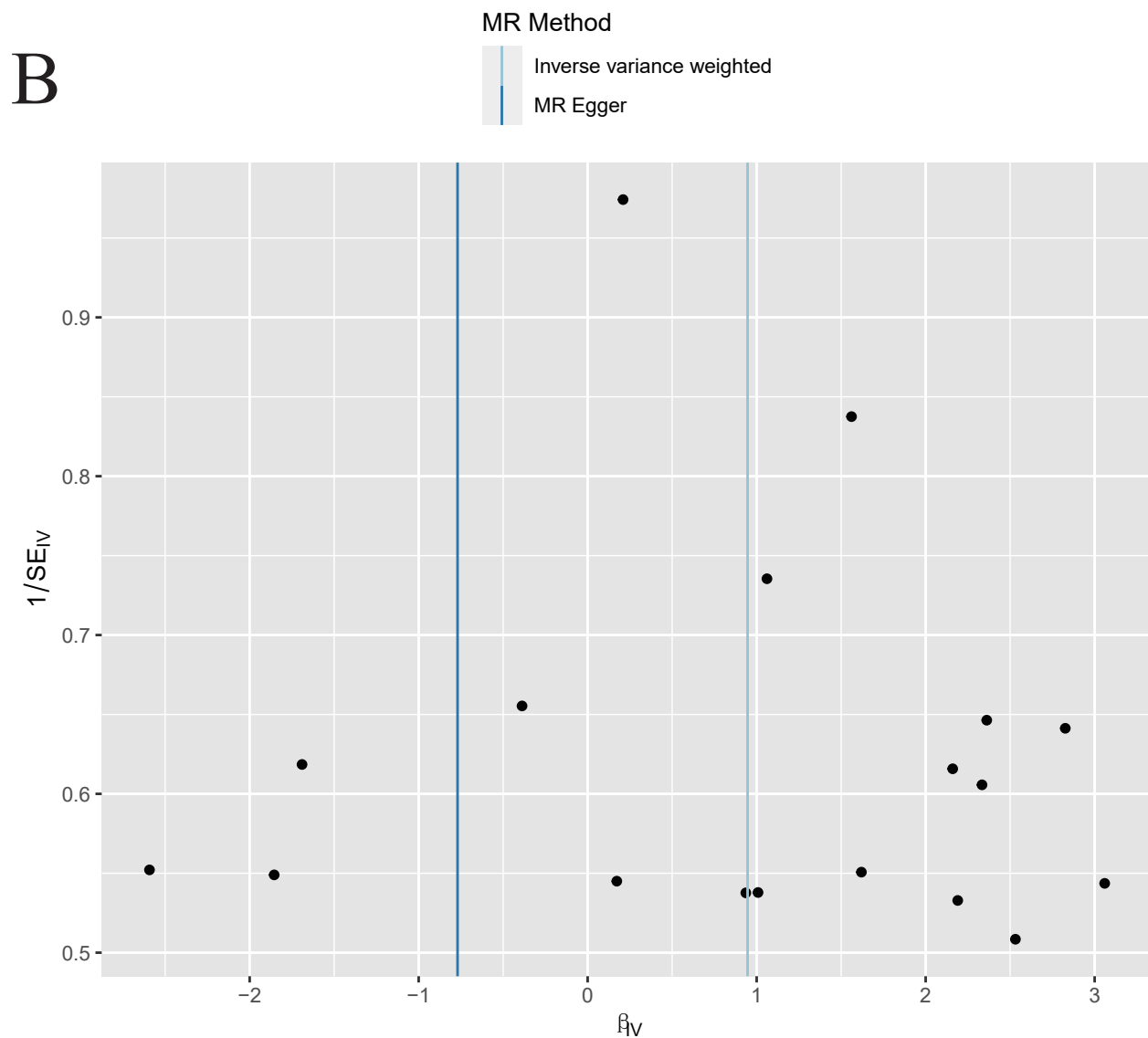

C

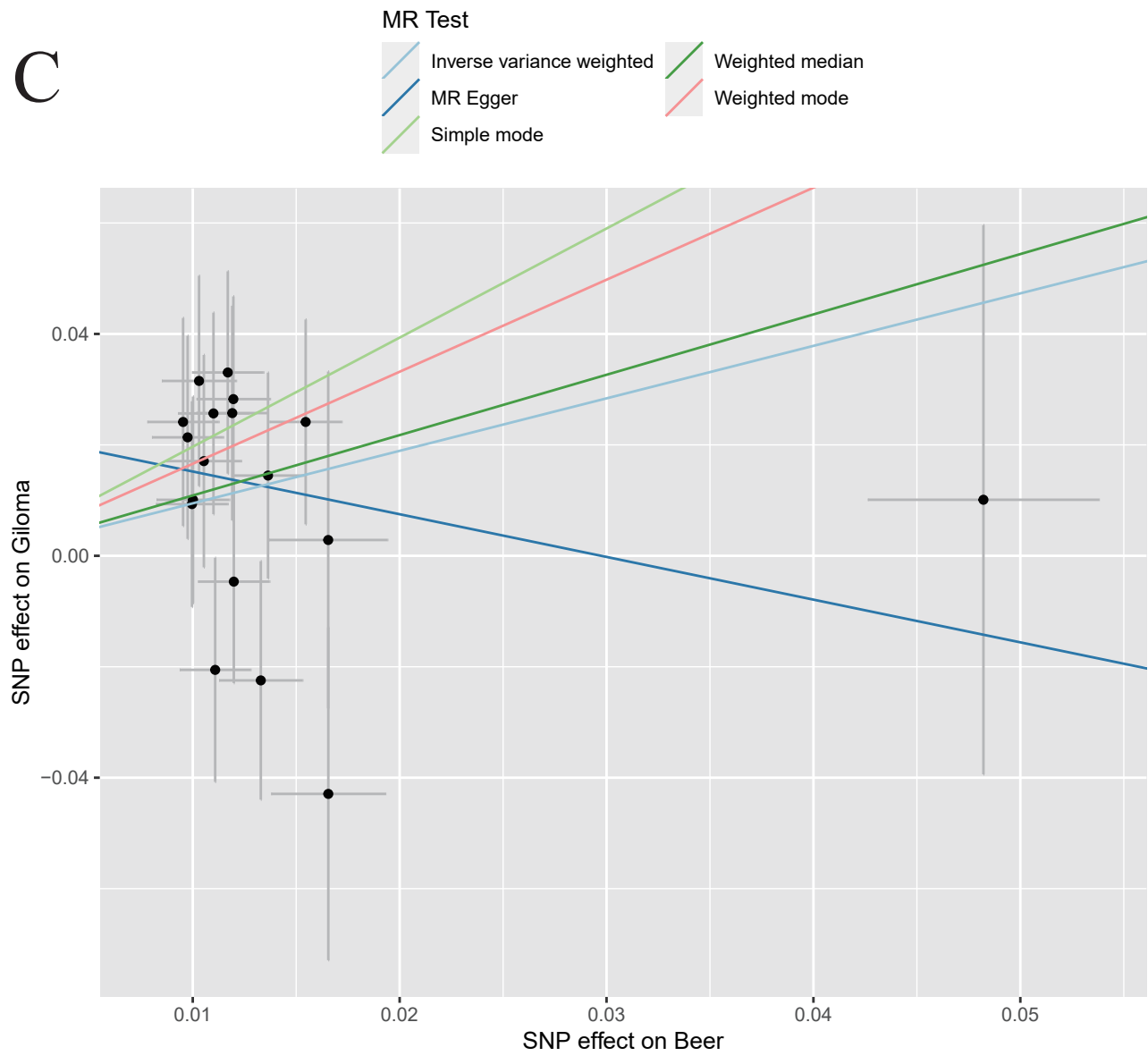

D

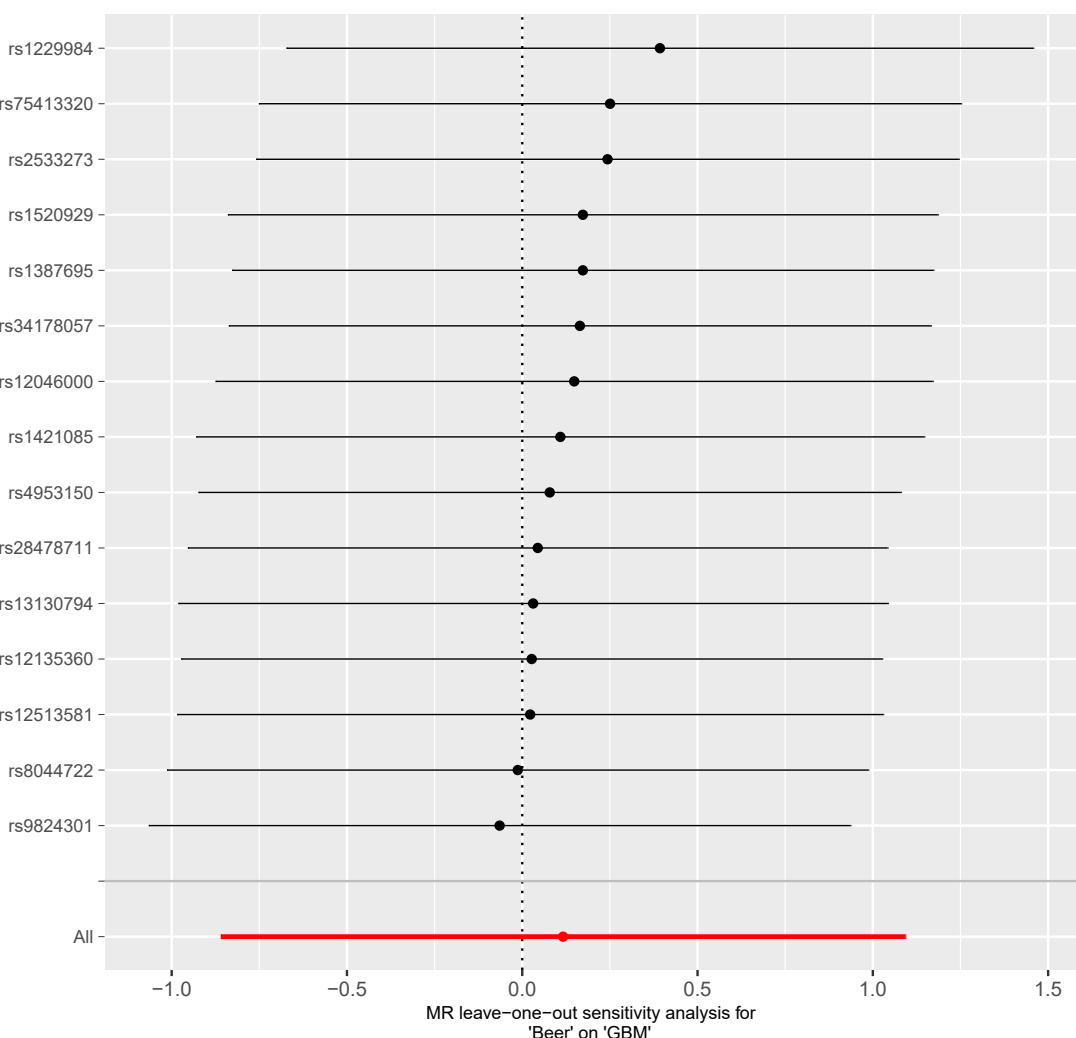

E

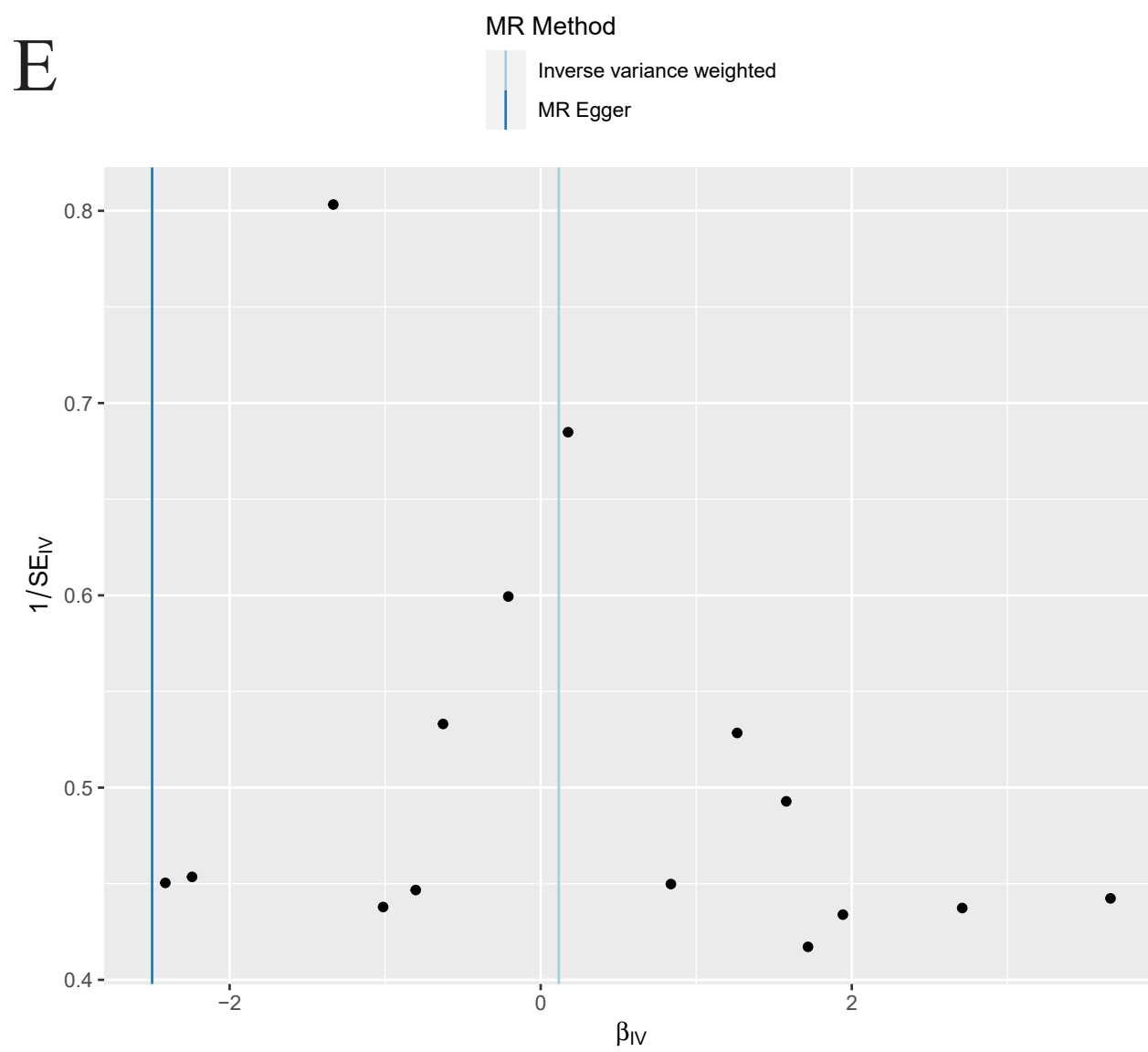

F

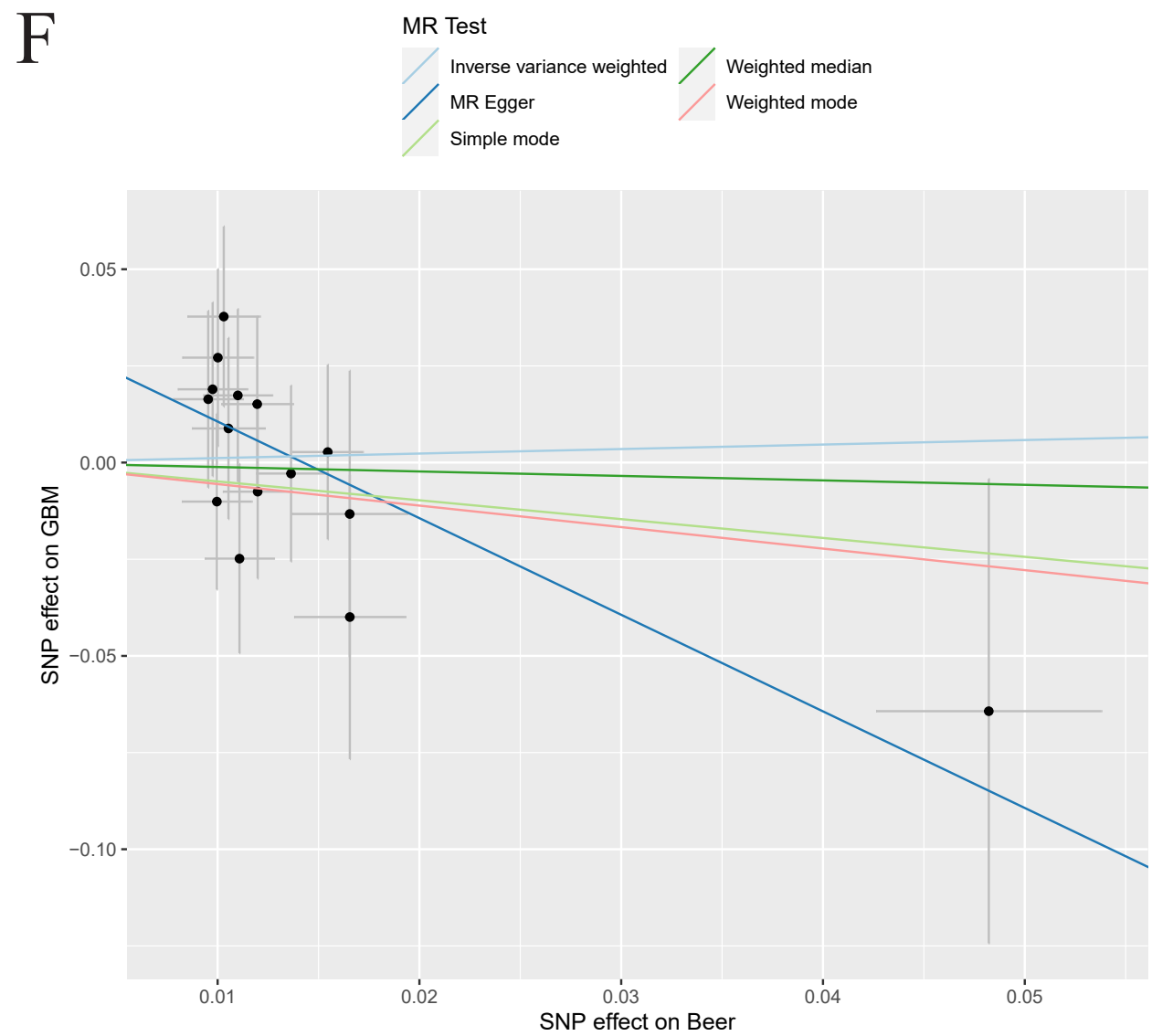

G

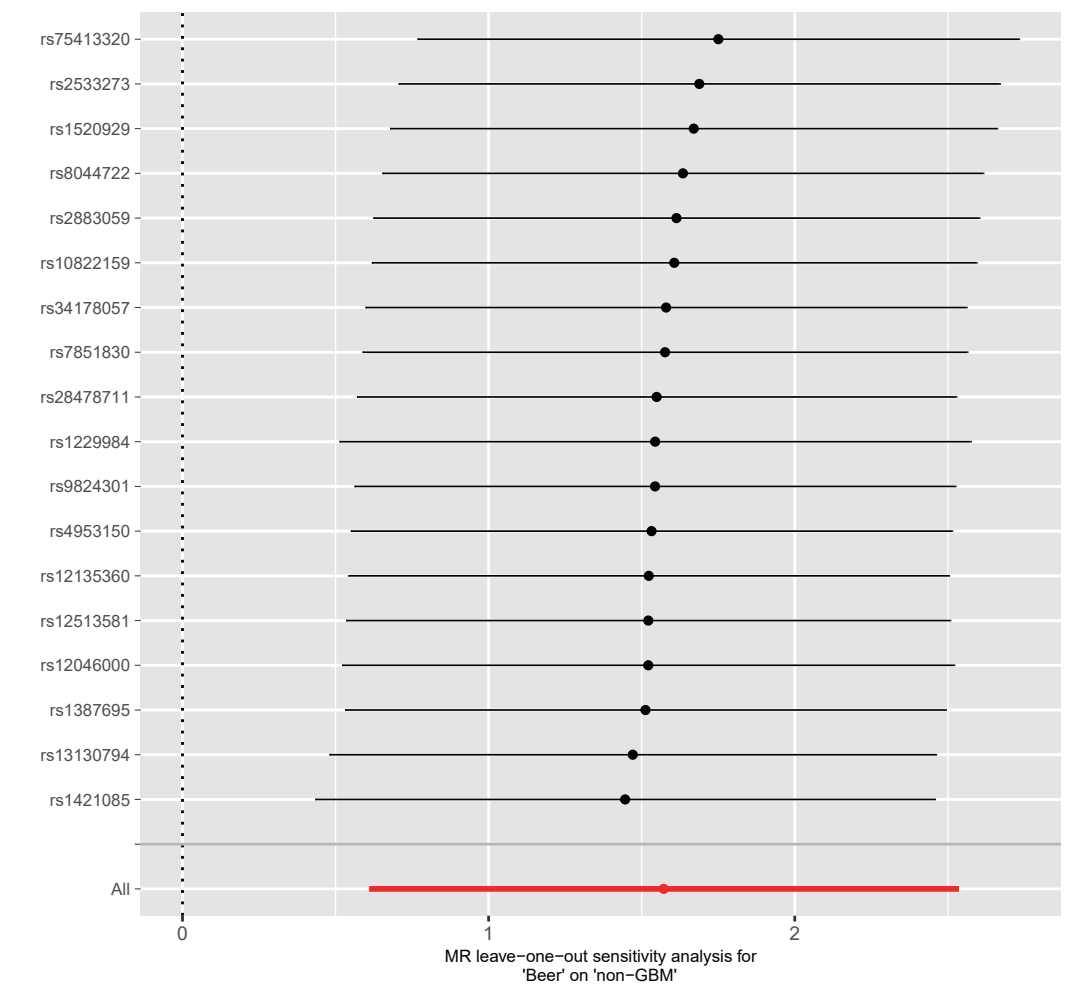

H

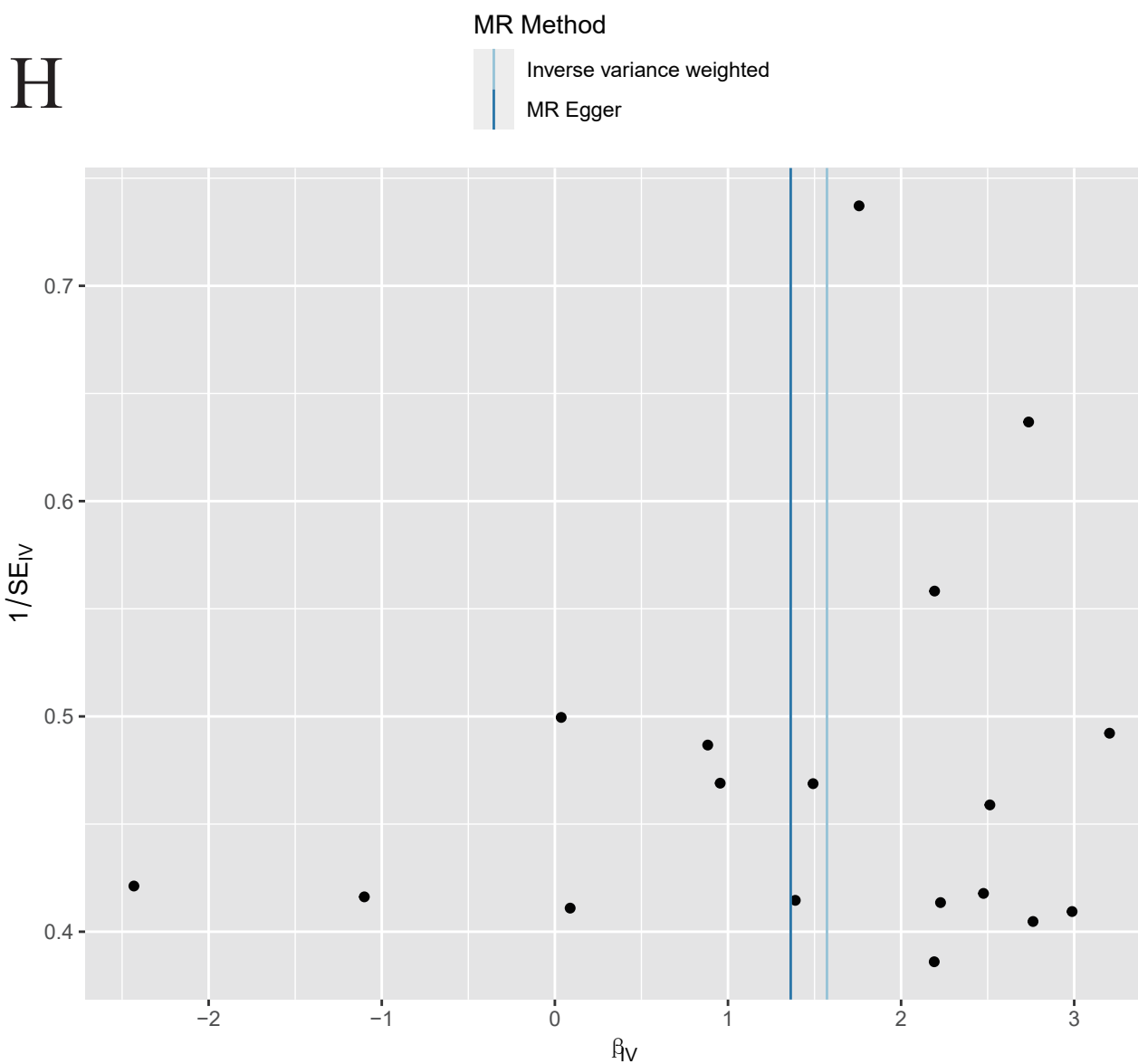

I

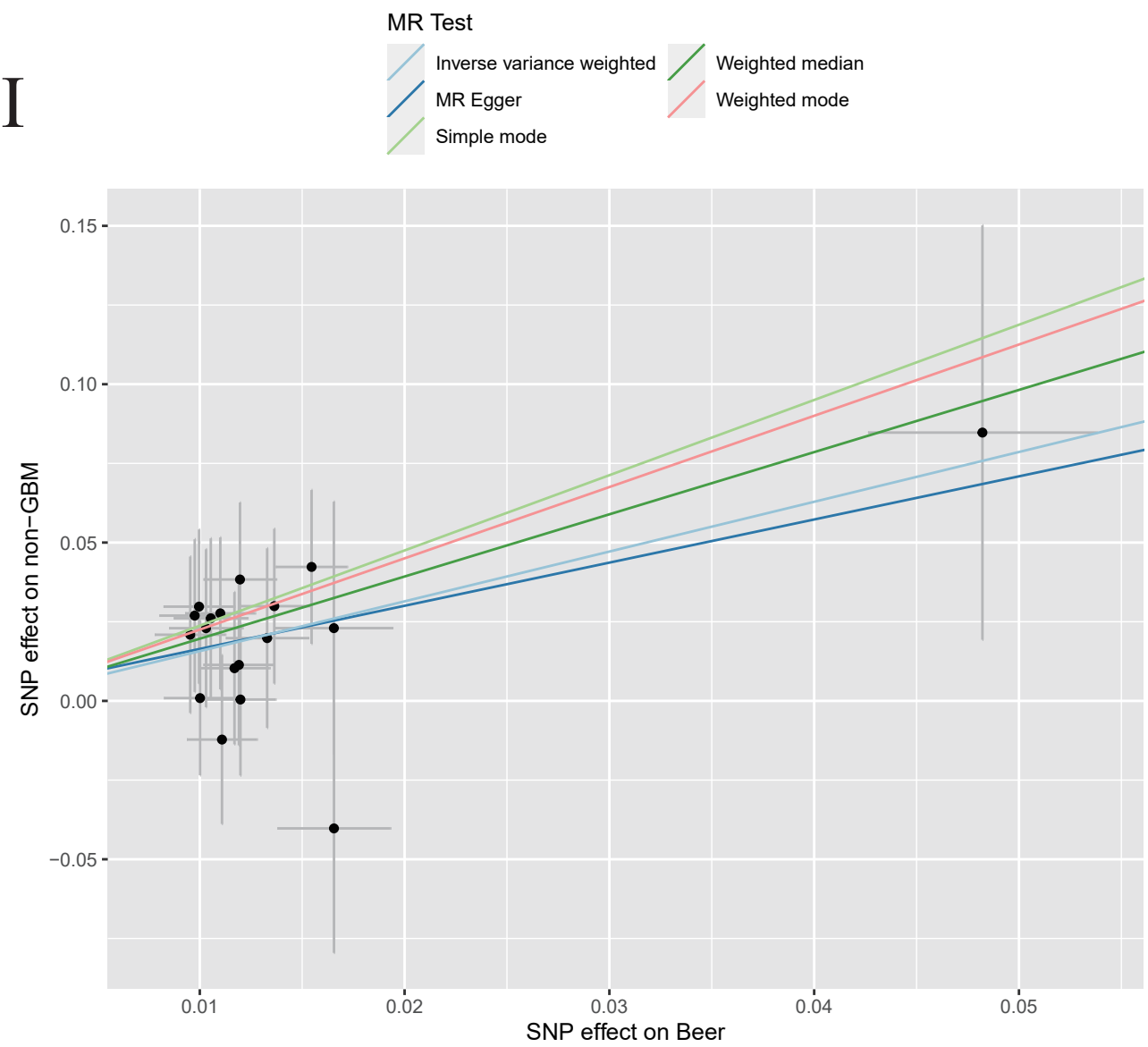

Supplement: Supplementary file 1 [file nutrients-17-00582-s001.zip › nutrients-3462880-supplementary/Sup_2.pdf]

A

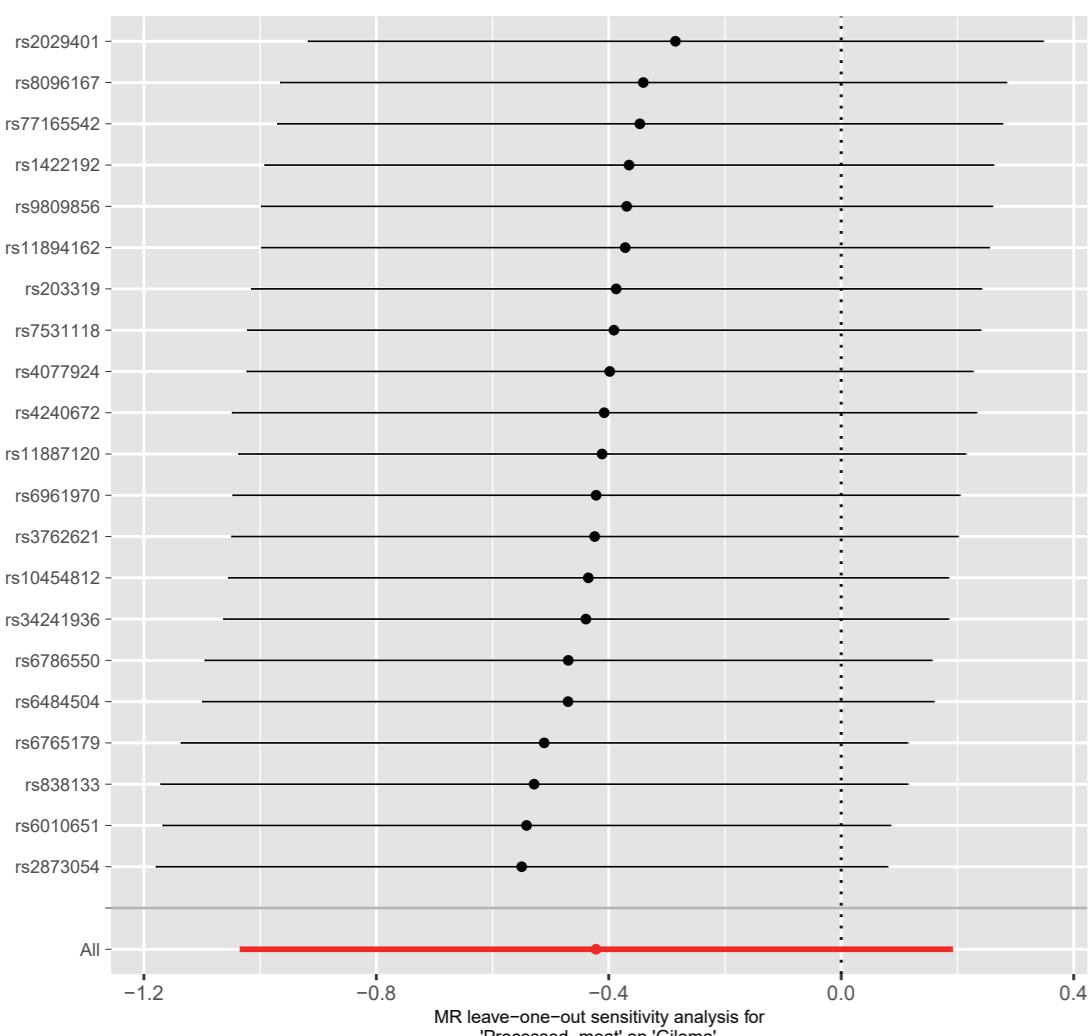

B

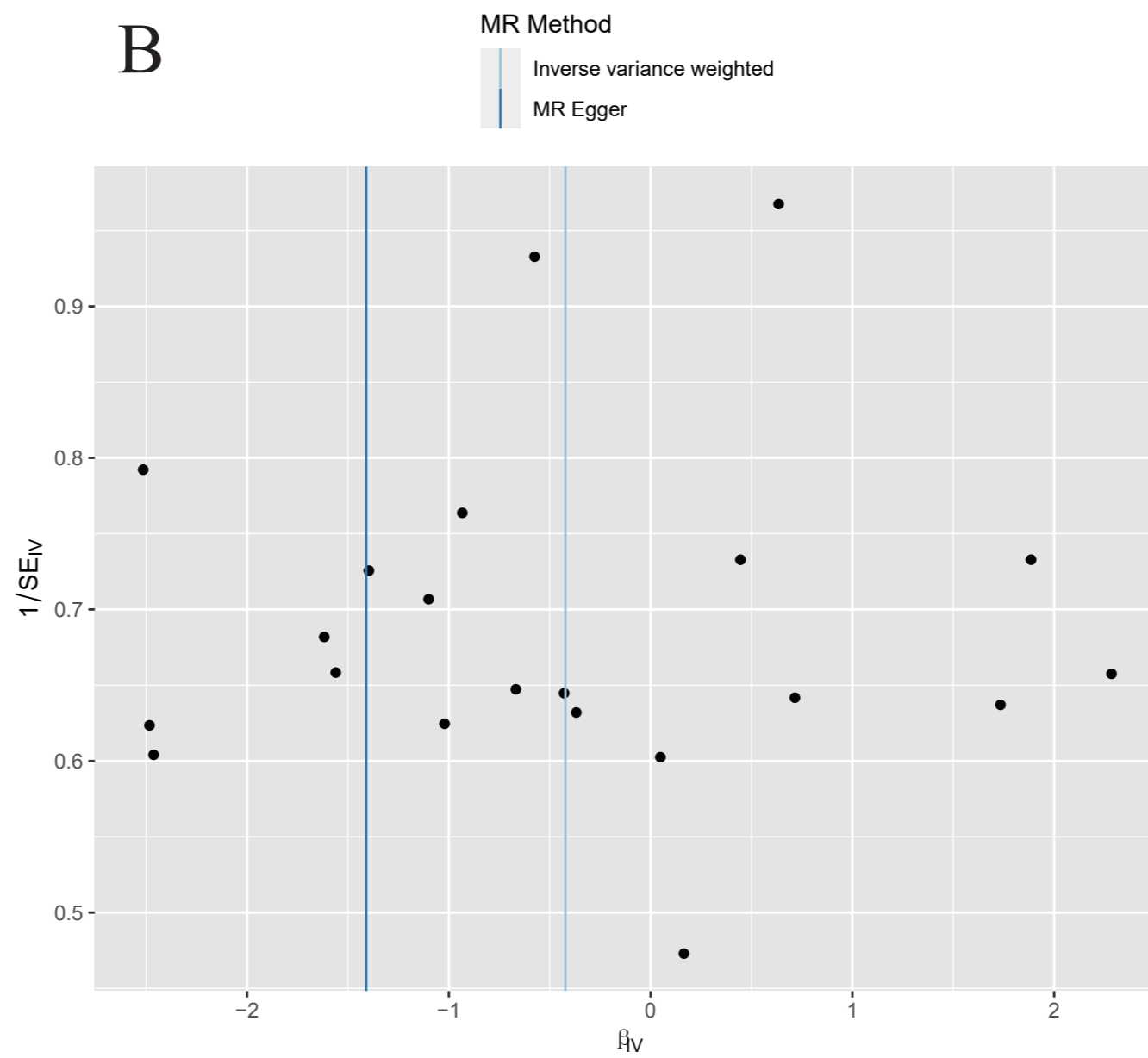

C

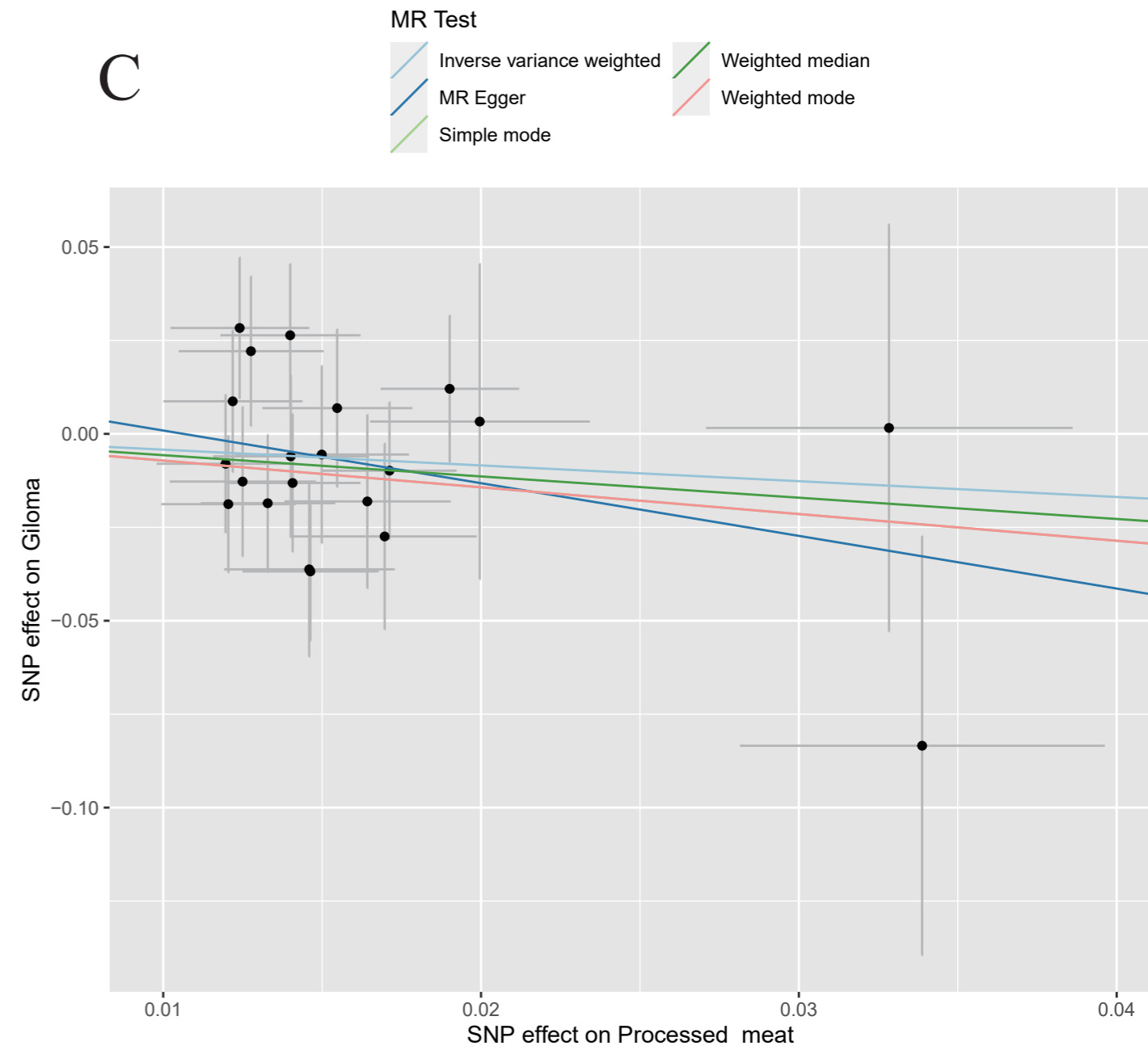

D

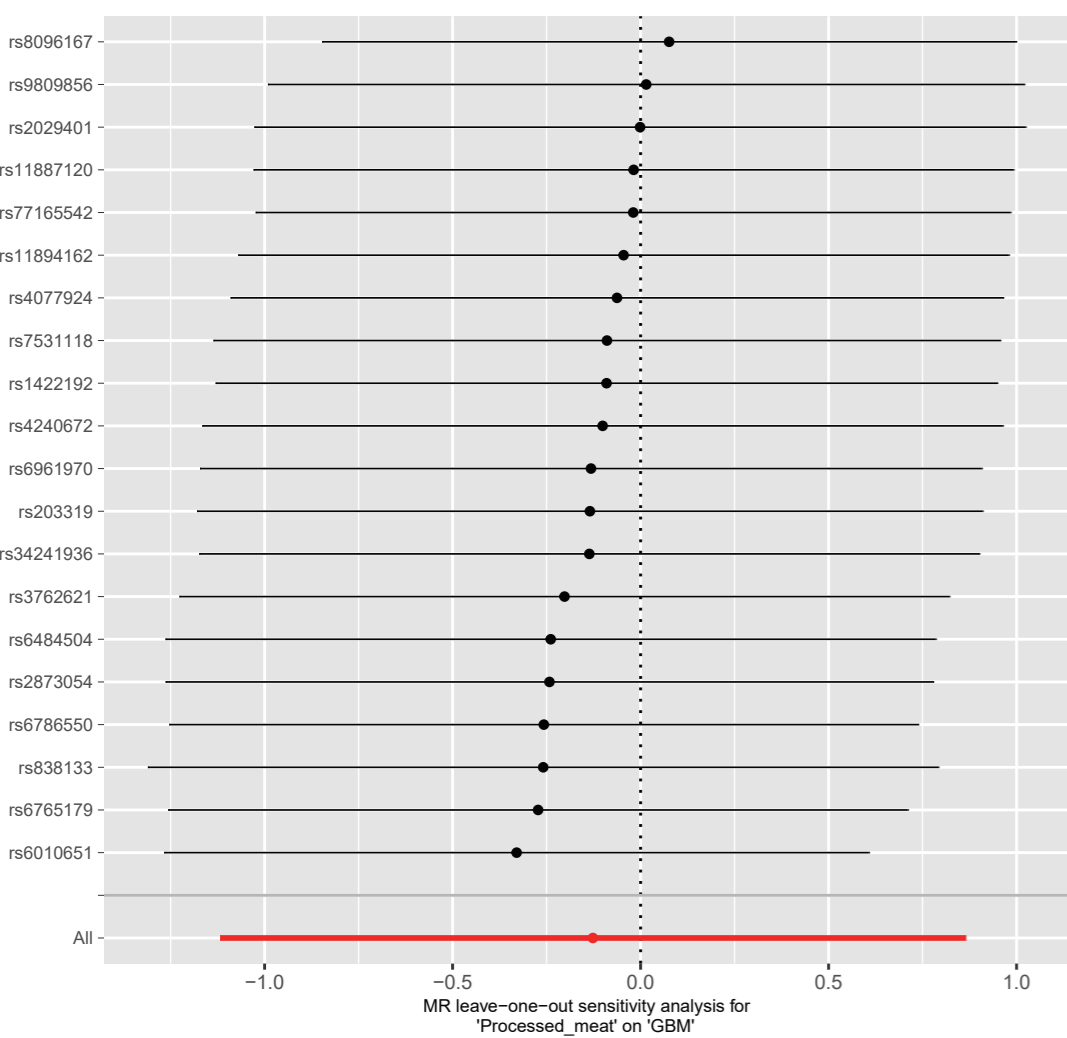

E

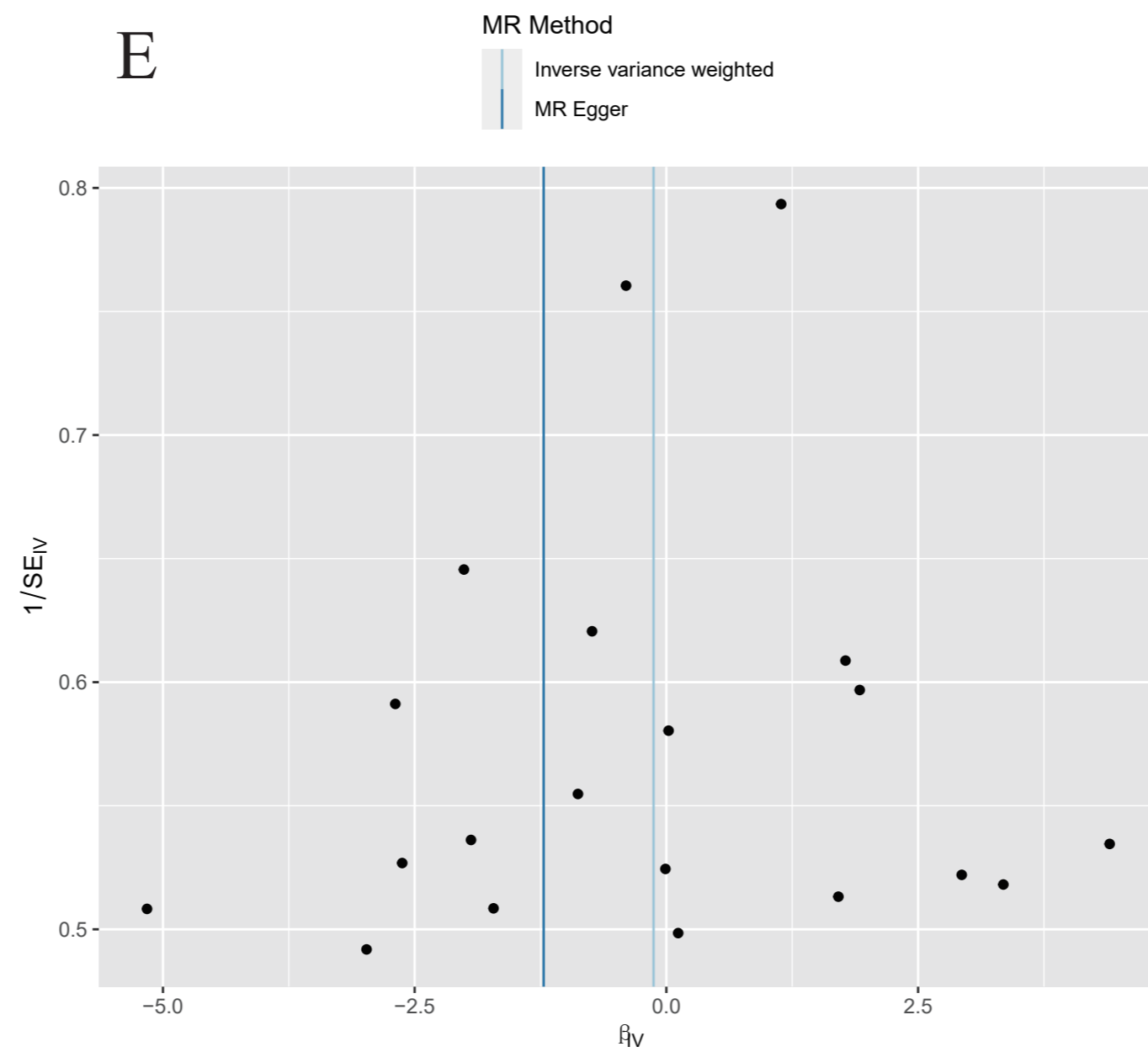

F

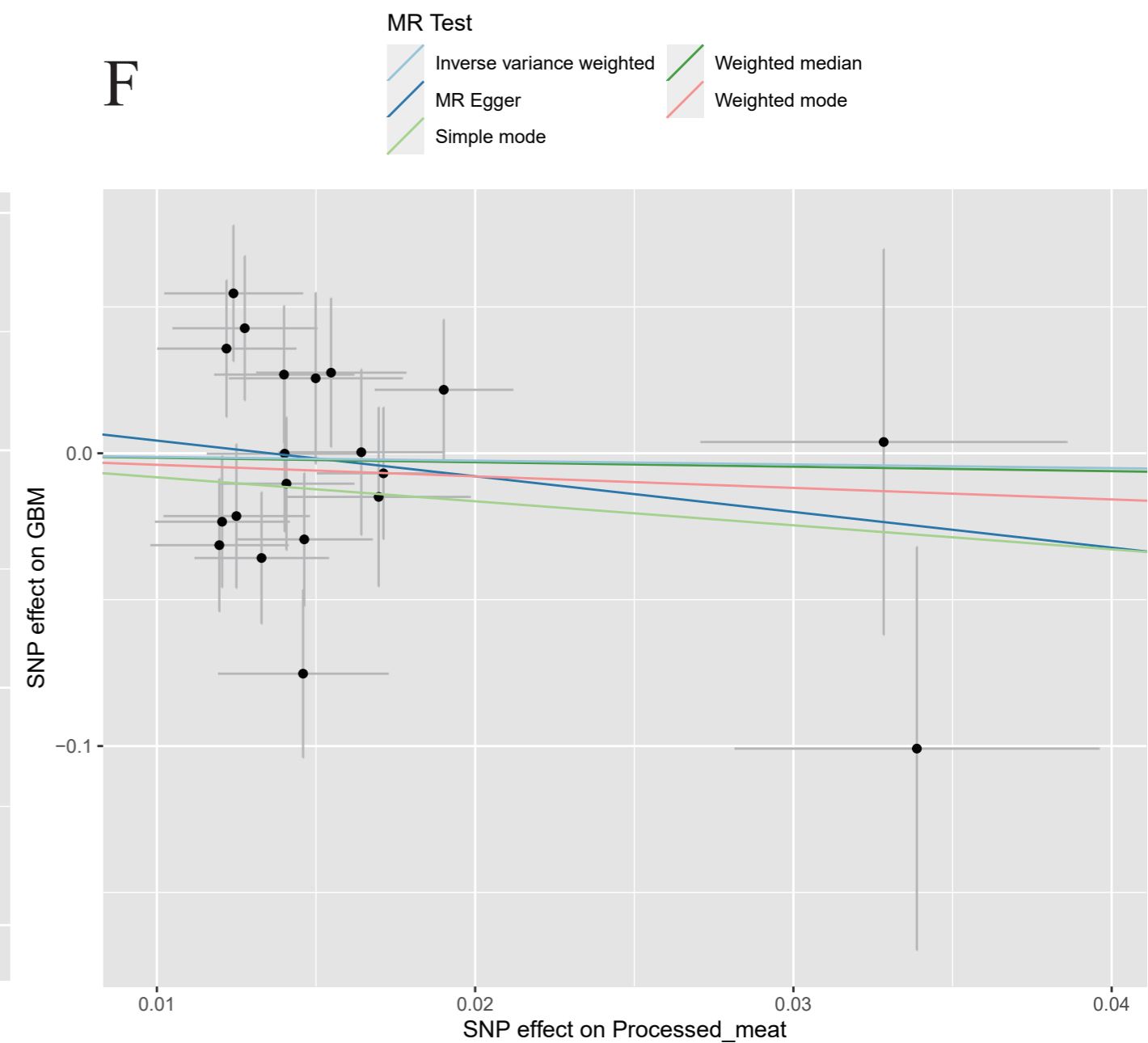

G

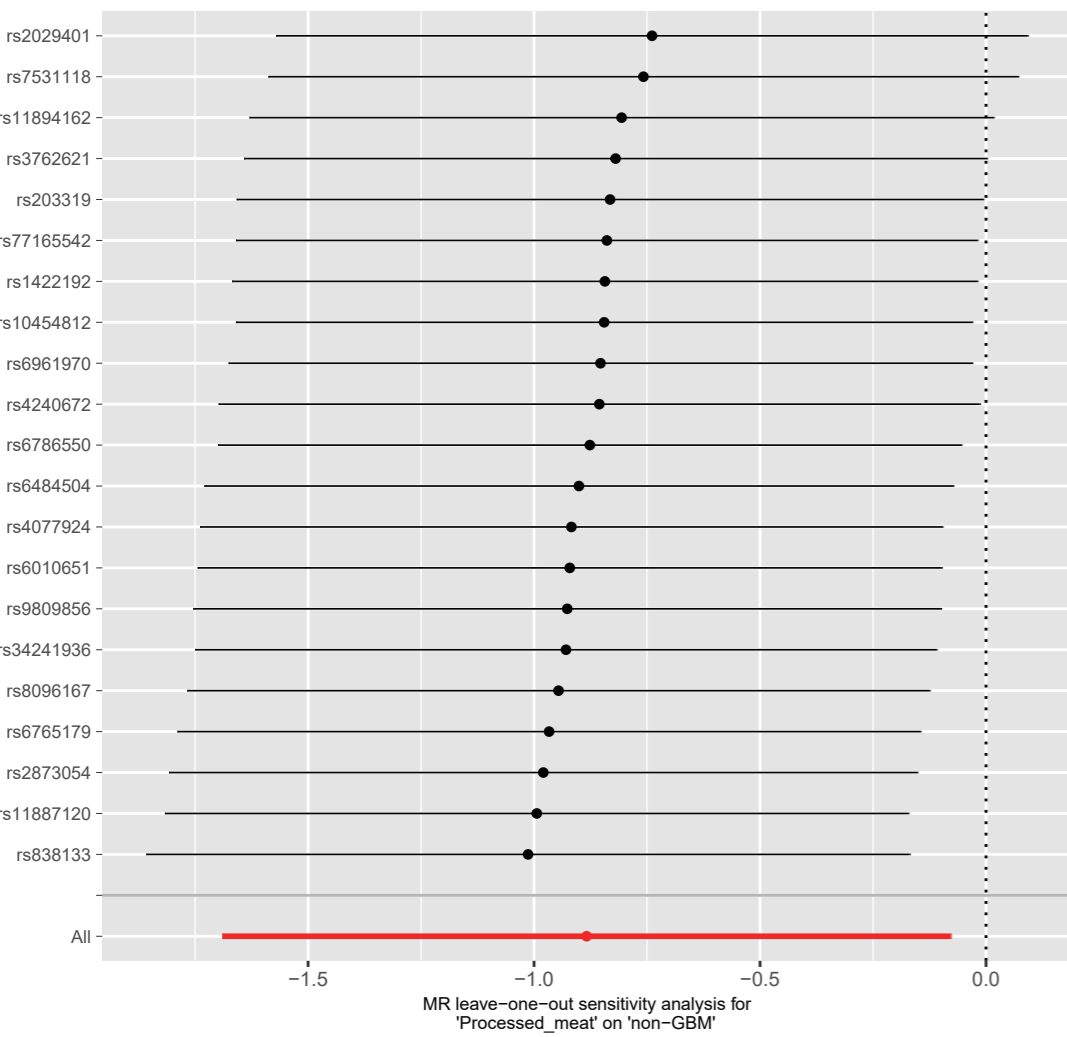

H

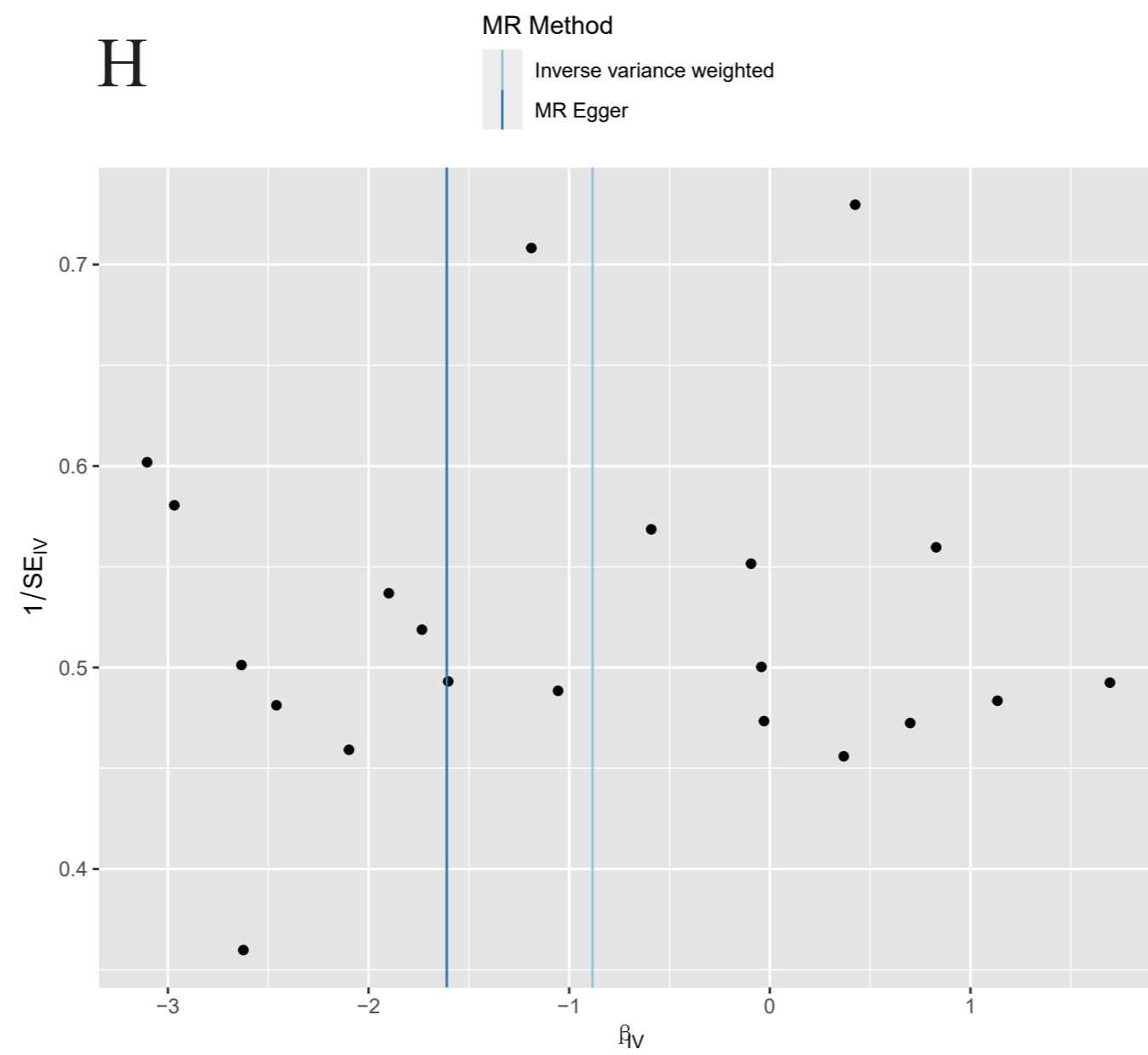

I

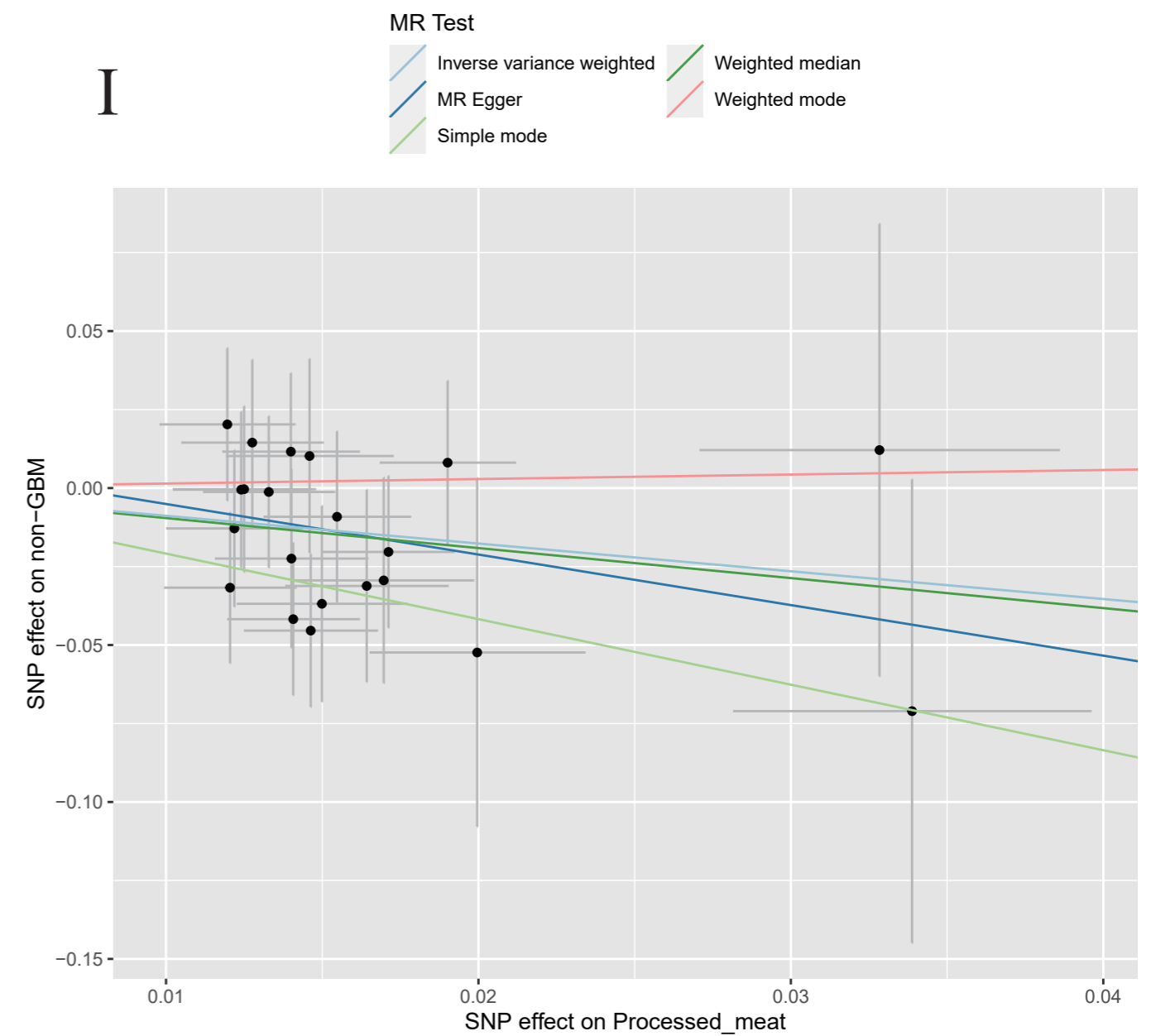

A

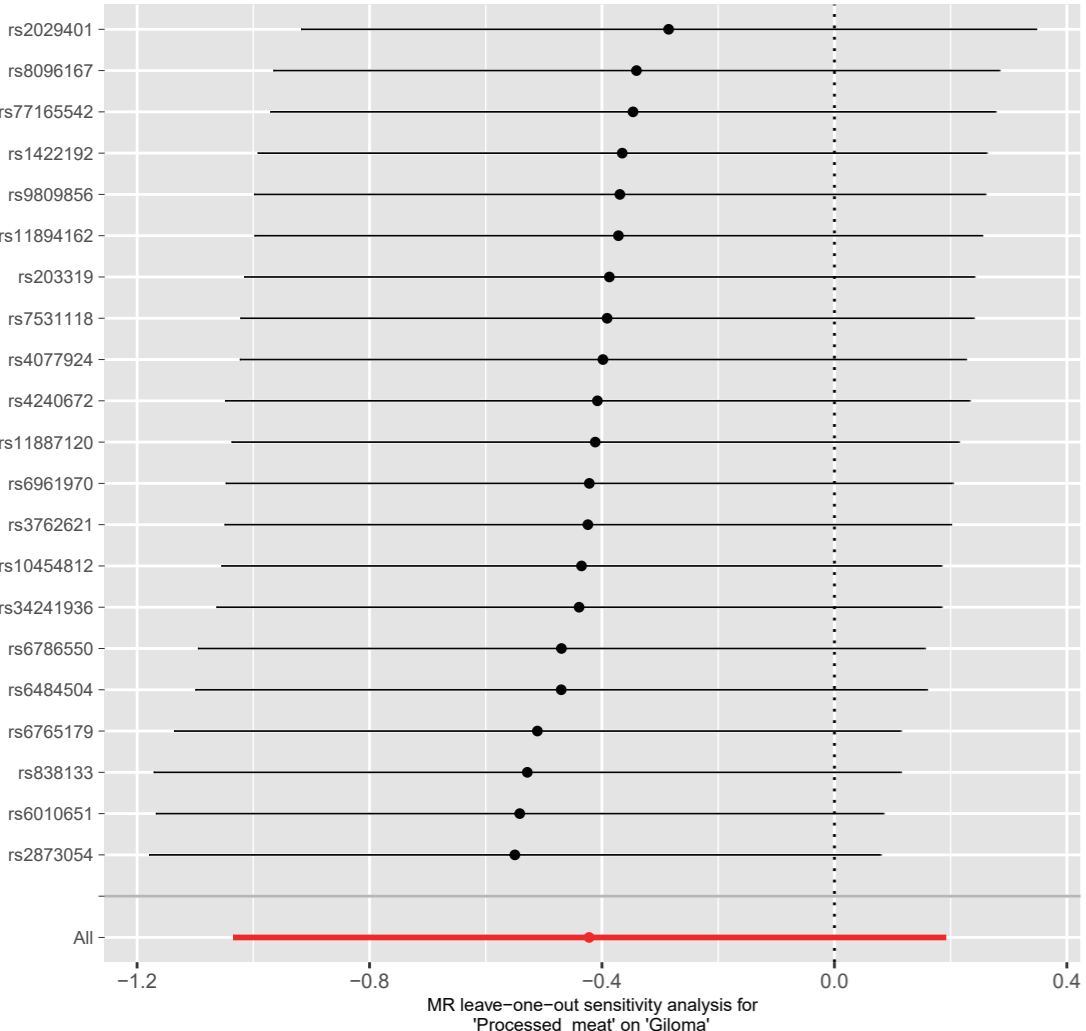

B

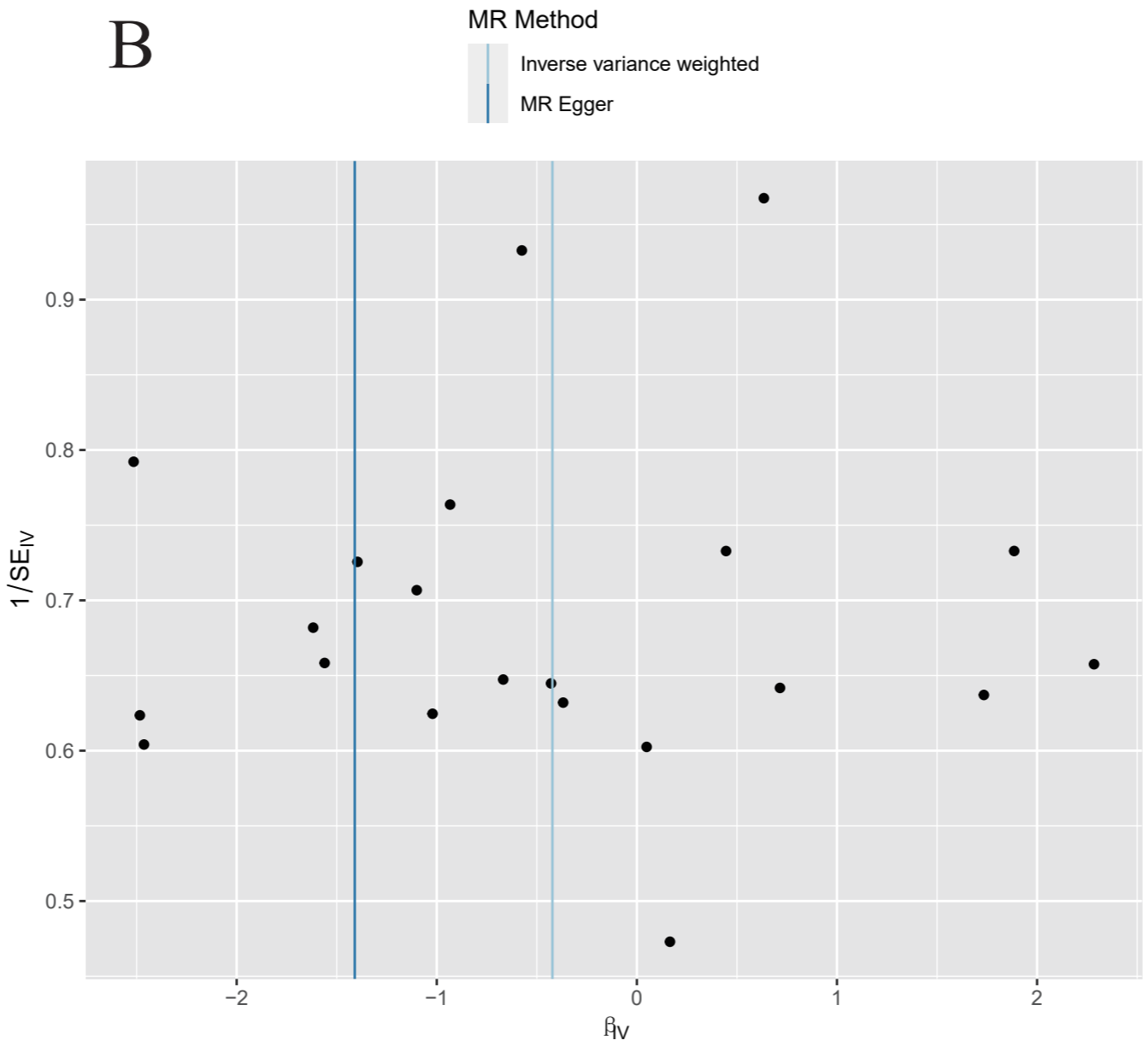

C

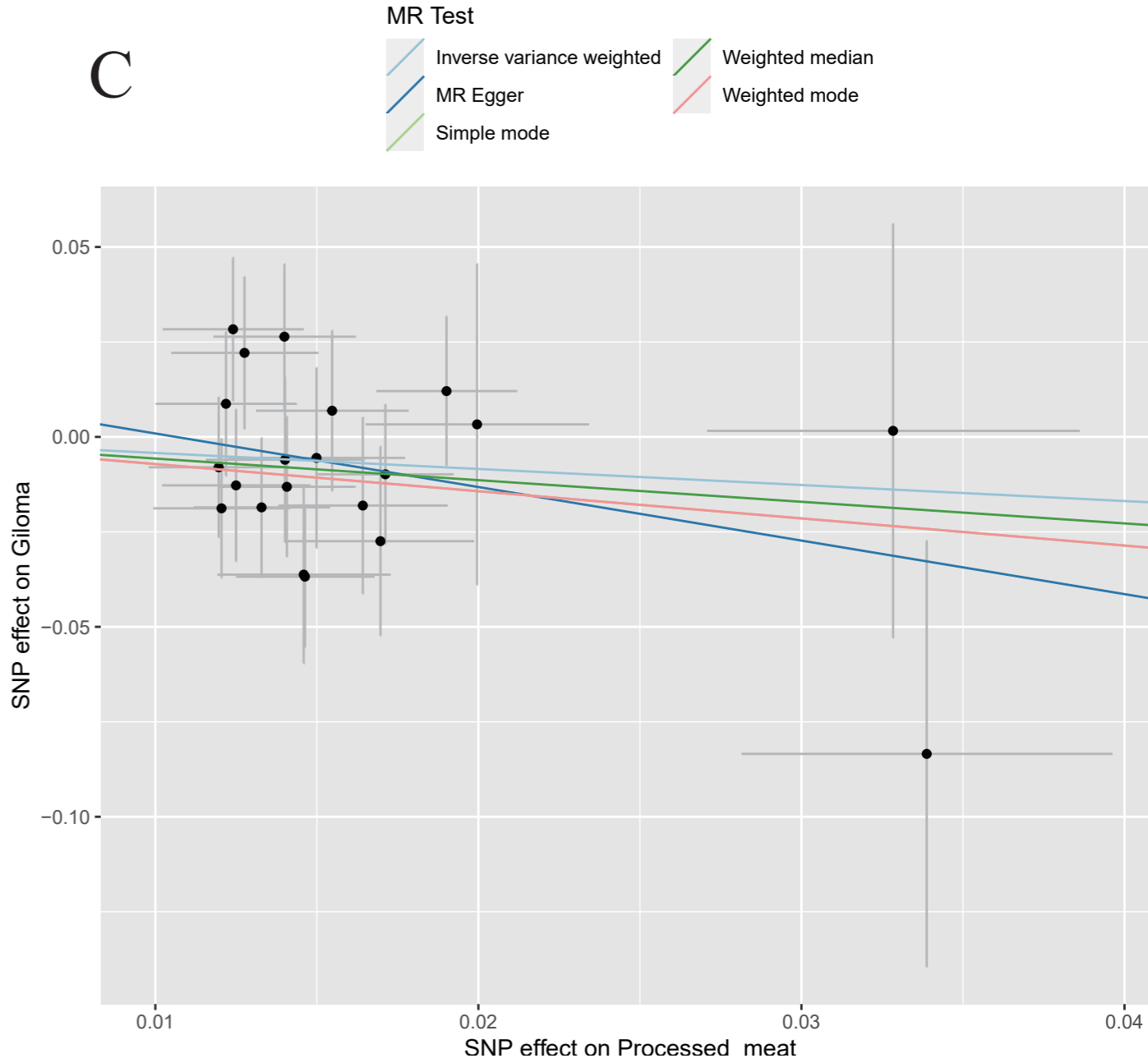

D

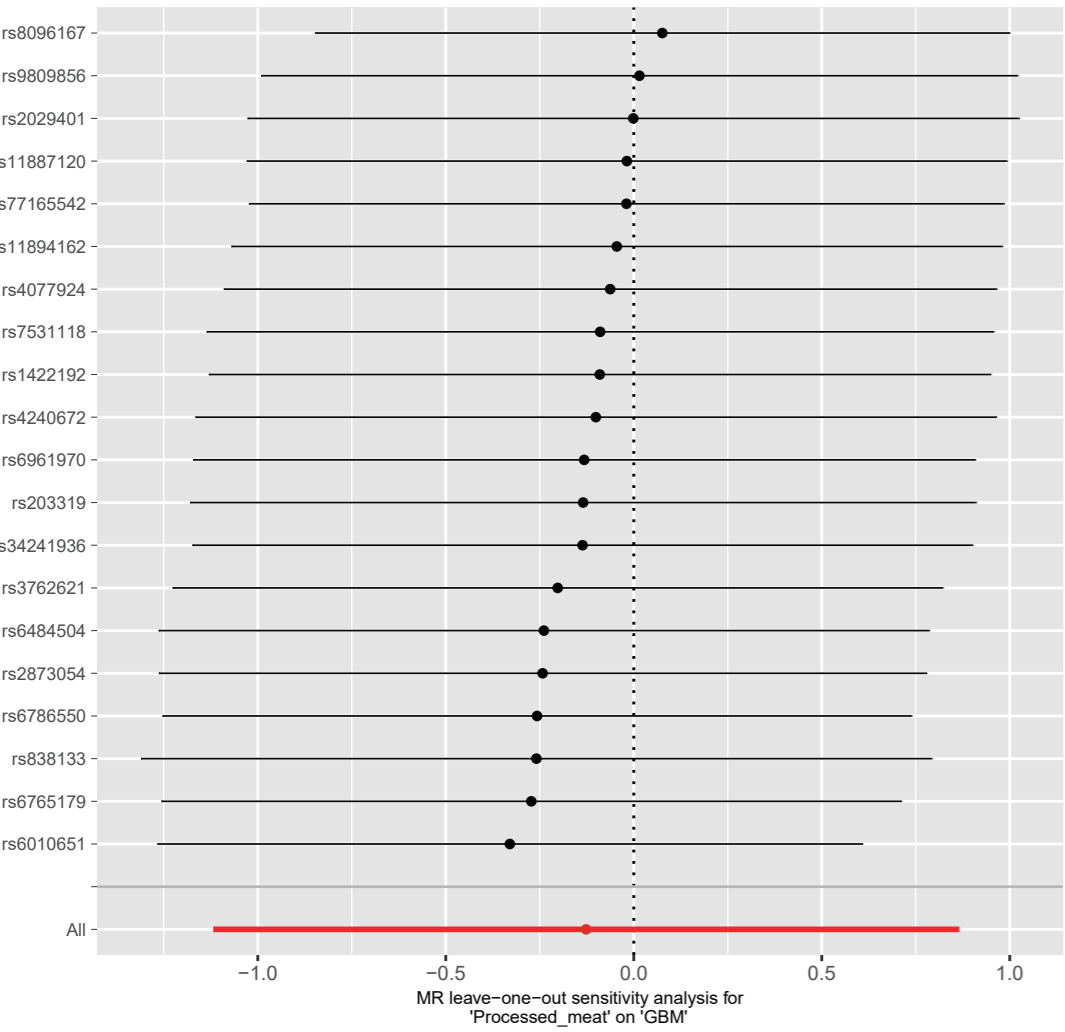

E

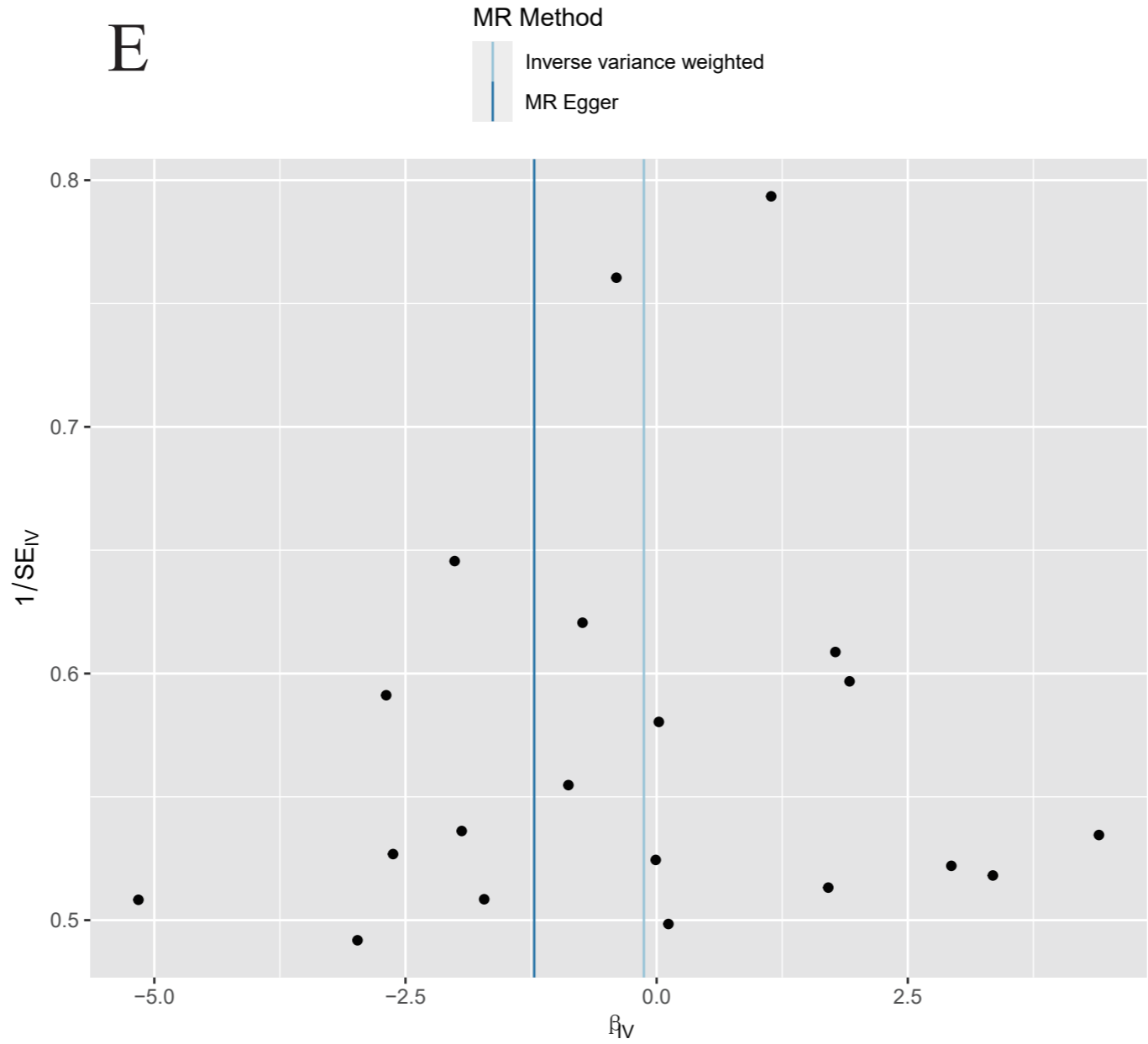

F

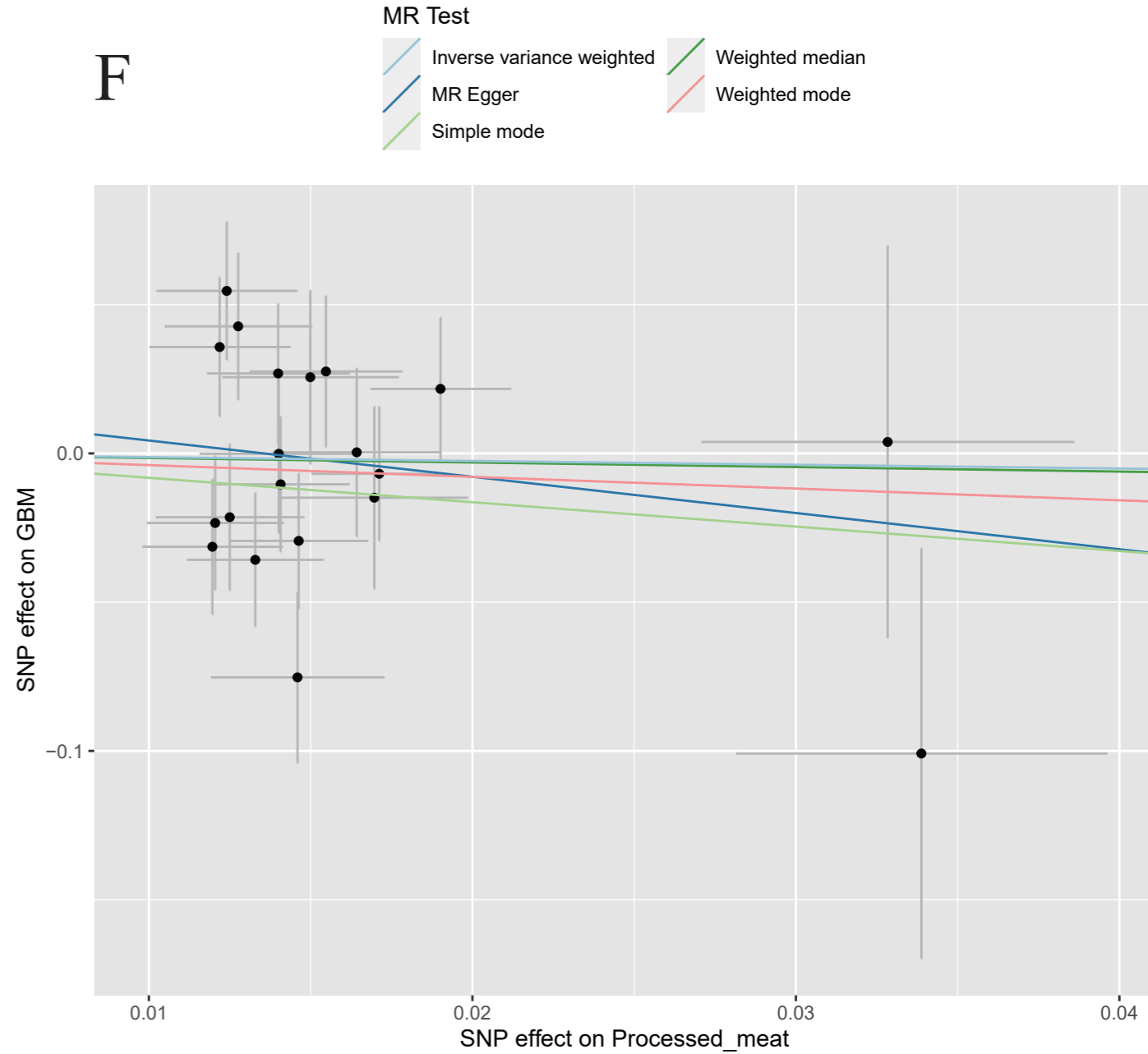

G

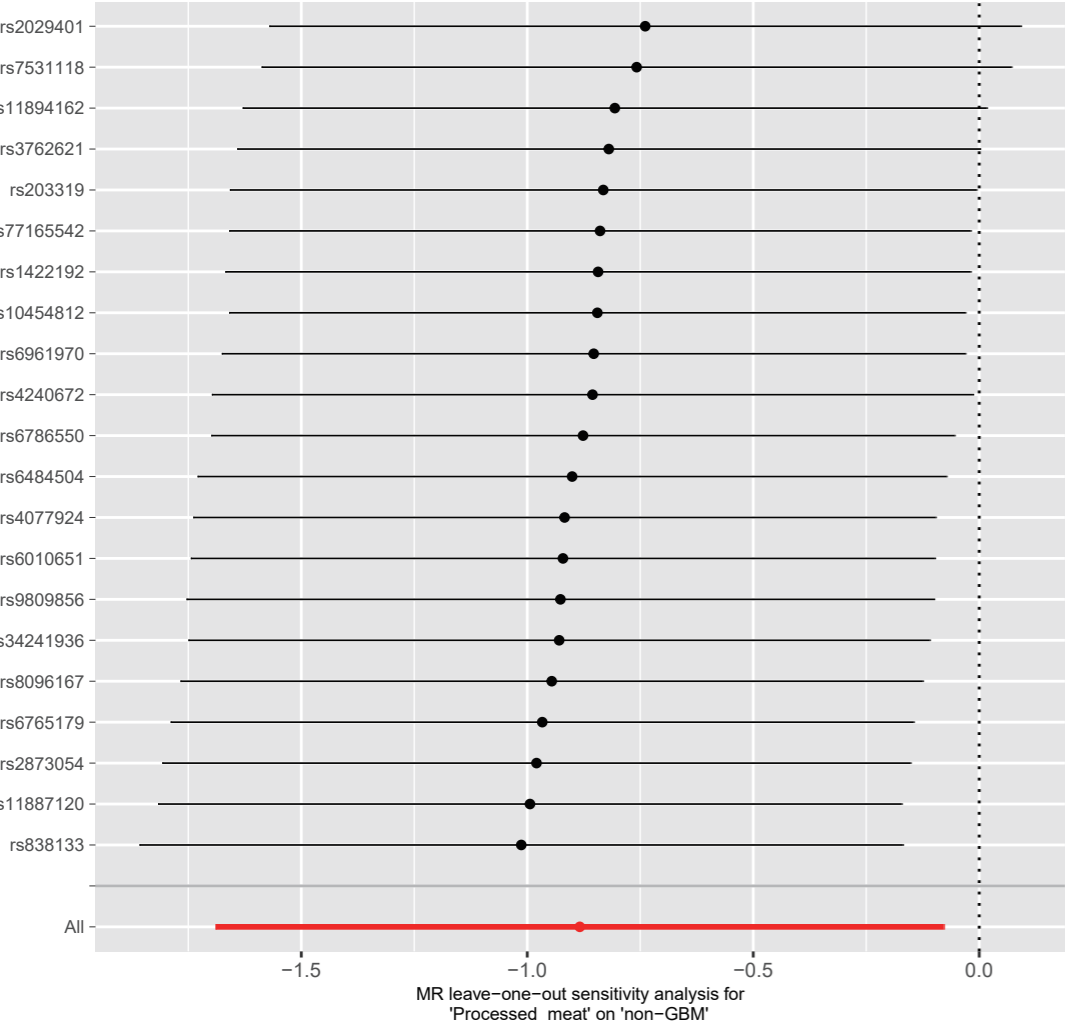

H

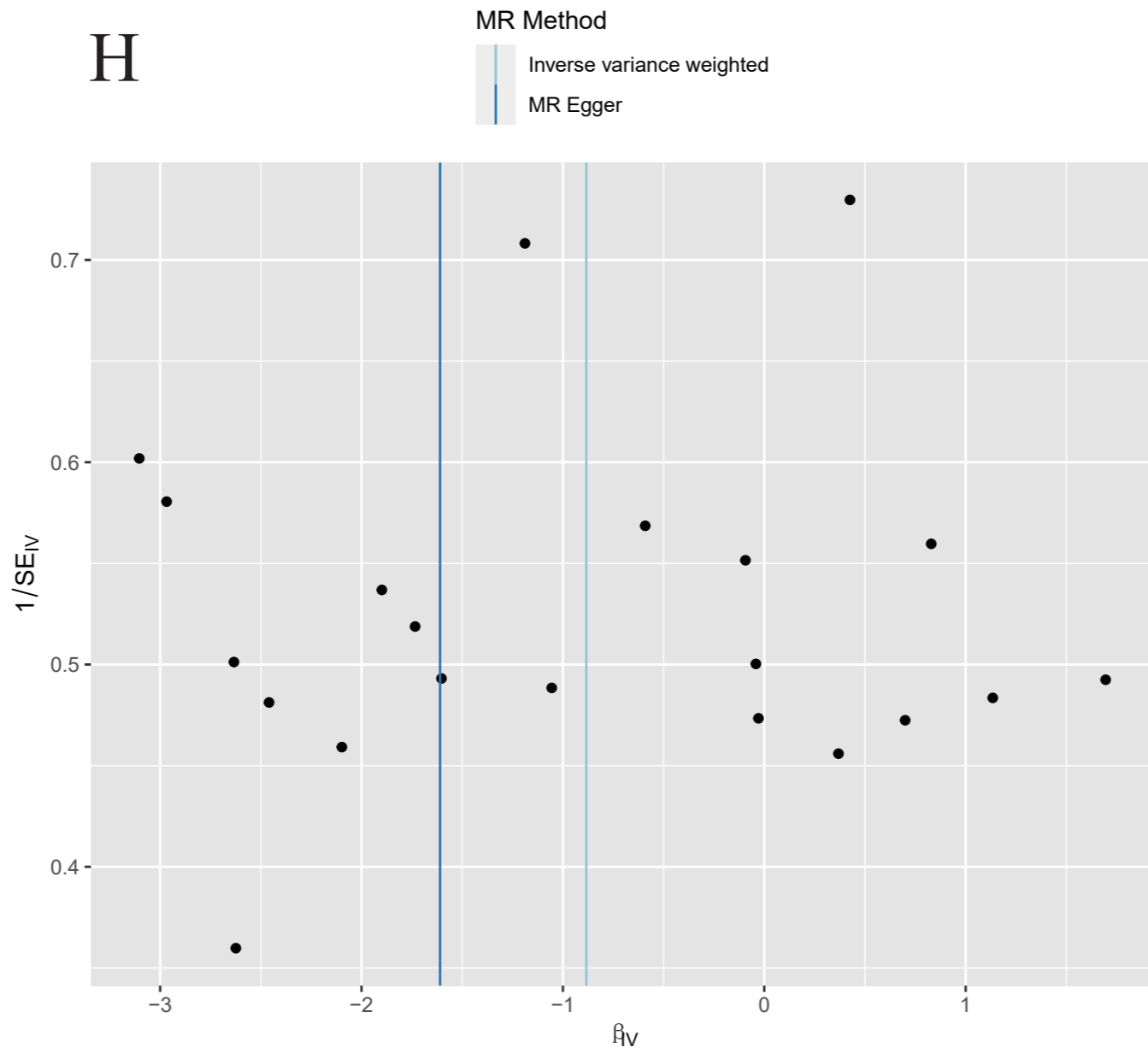

I

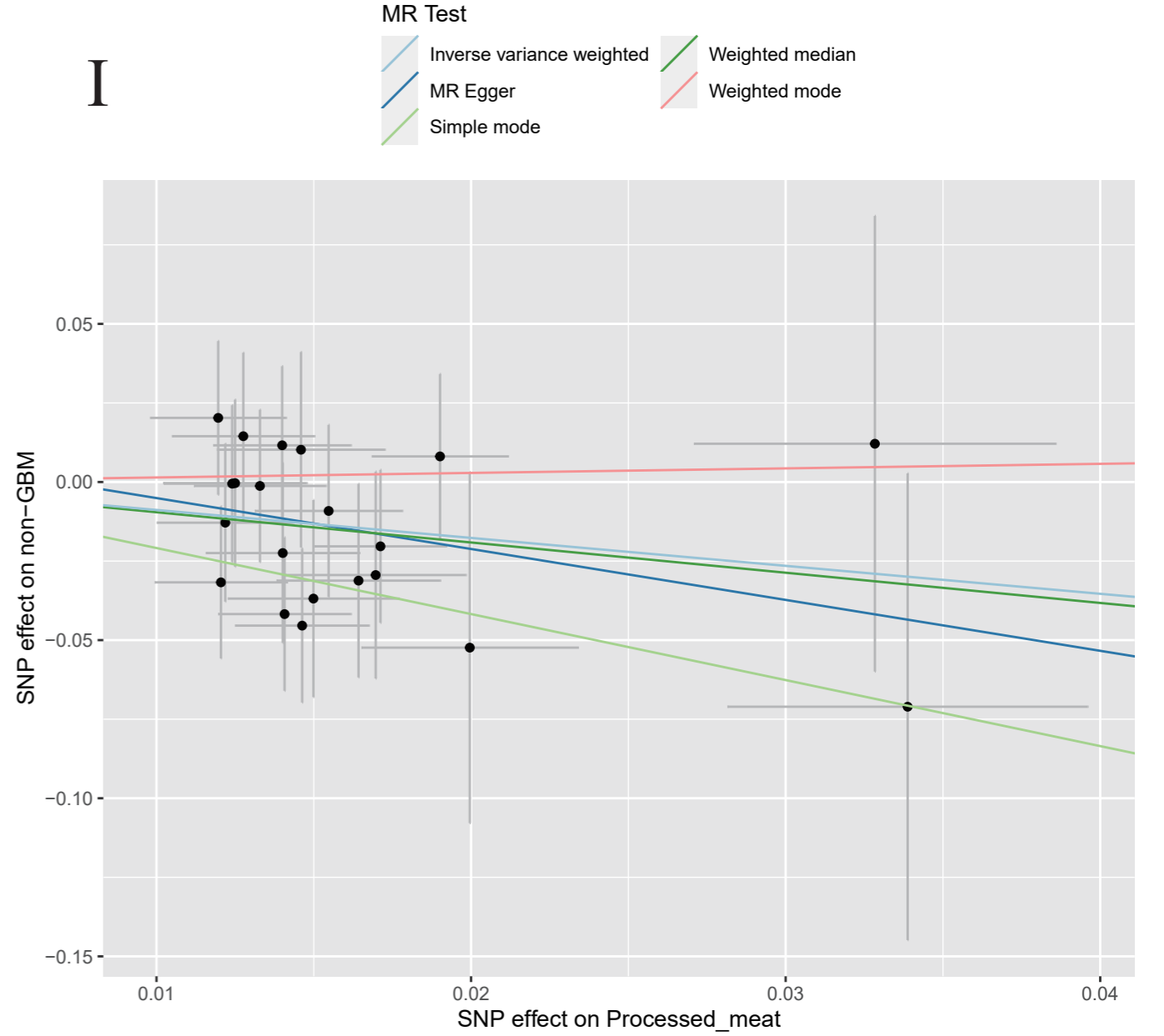

Supplement: Supplementary file 1 [file nutrients-17-00582-s001.zip › nutrients-3462880-supplementary/Sup_20.pdf]

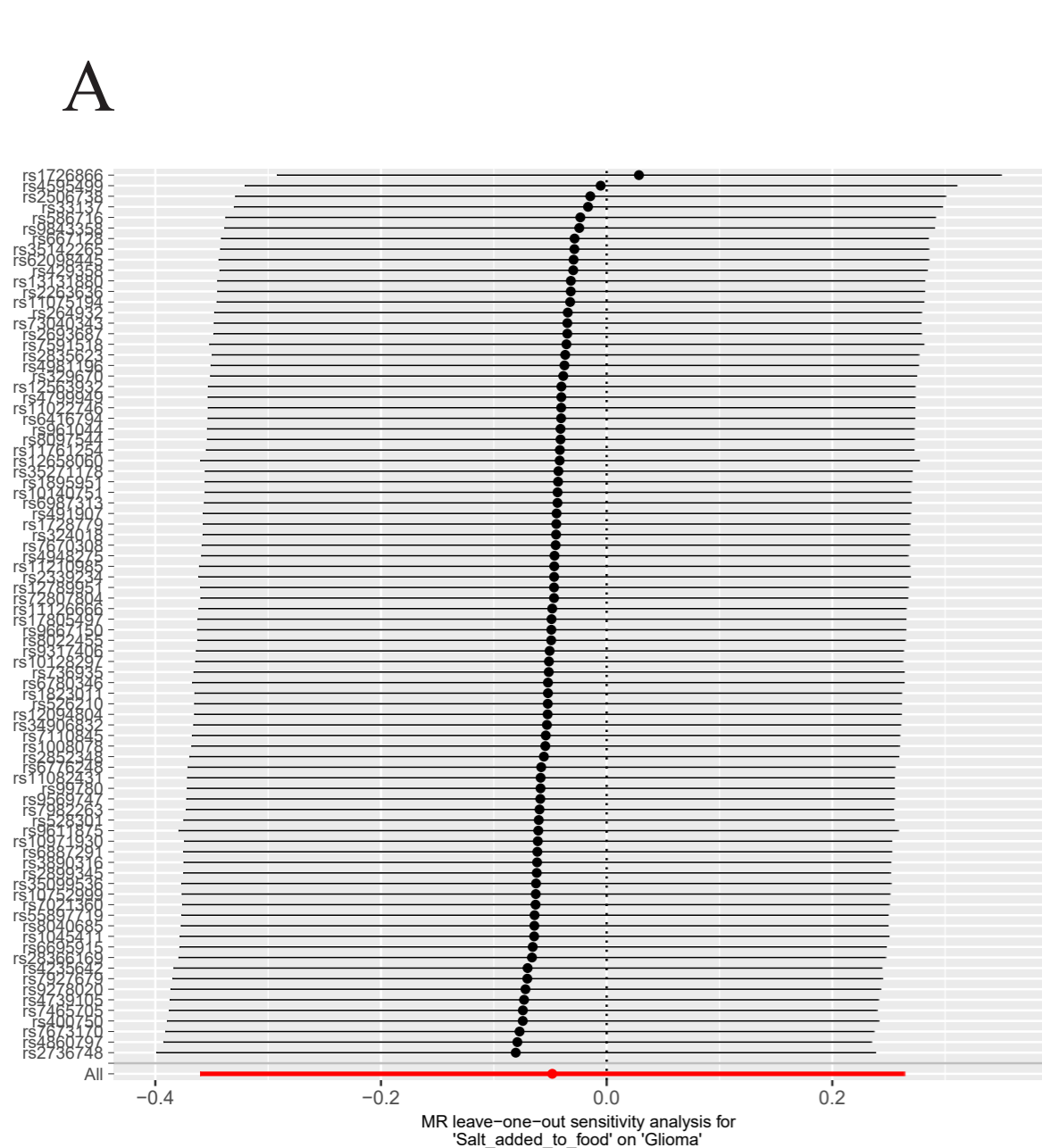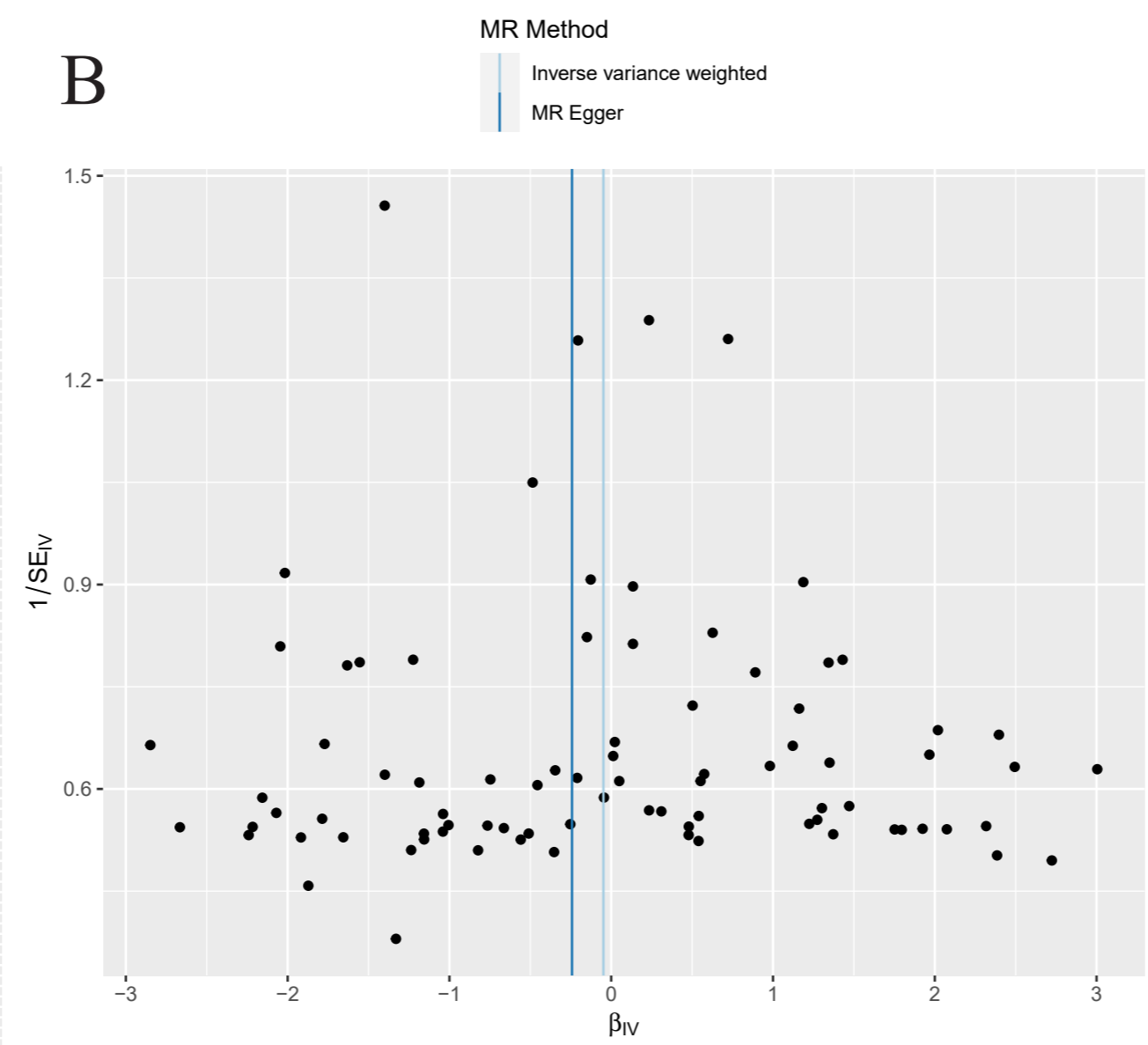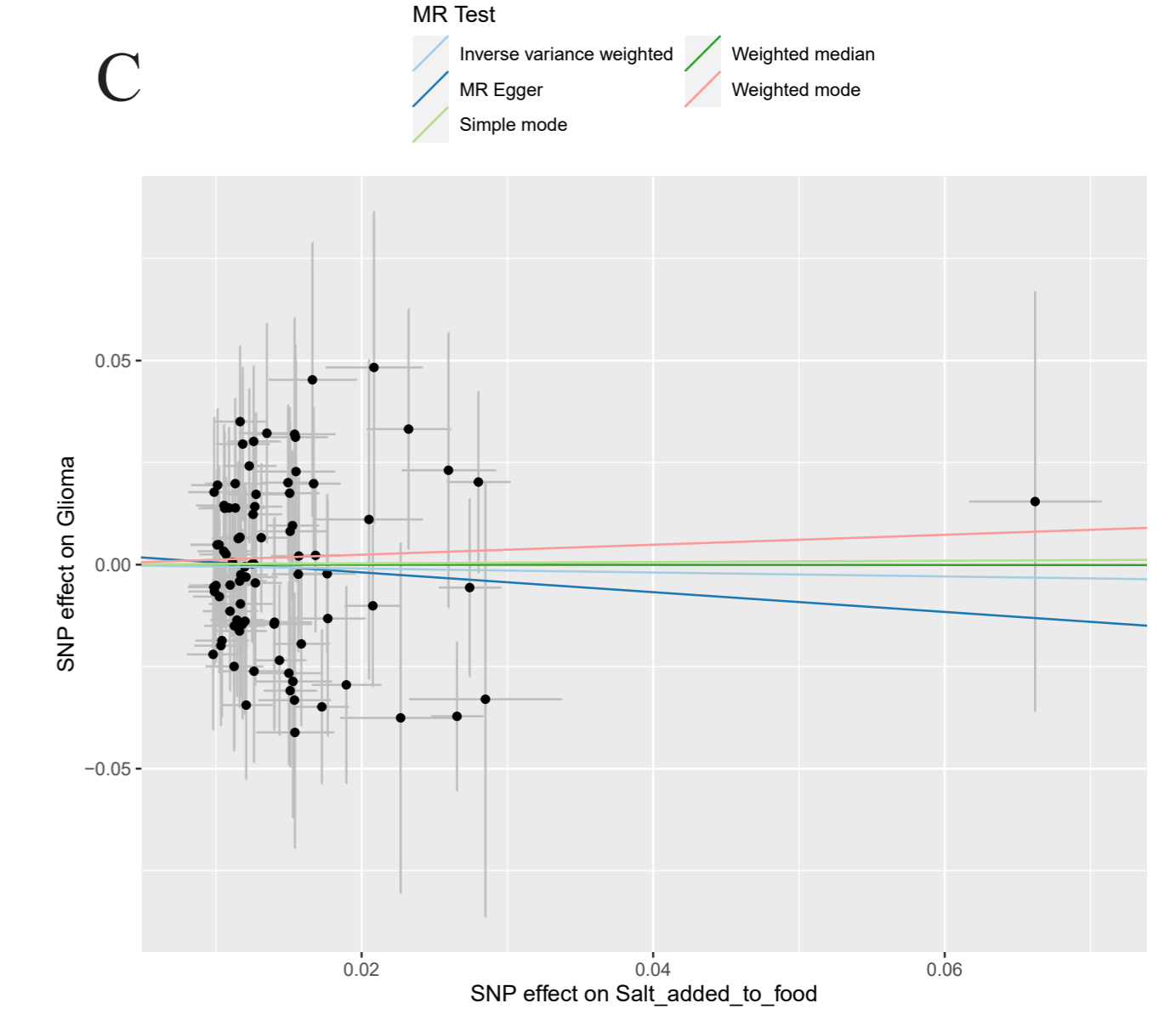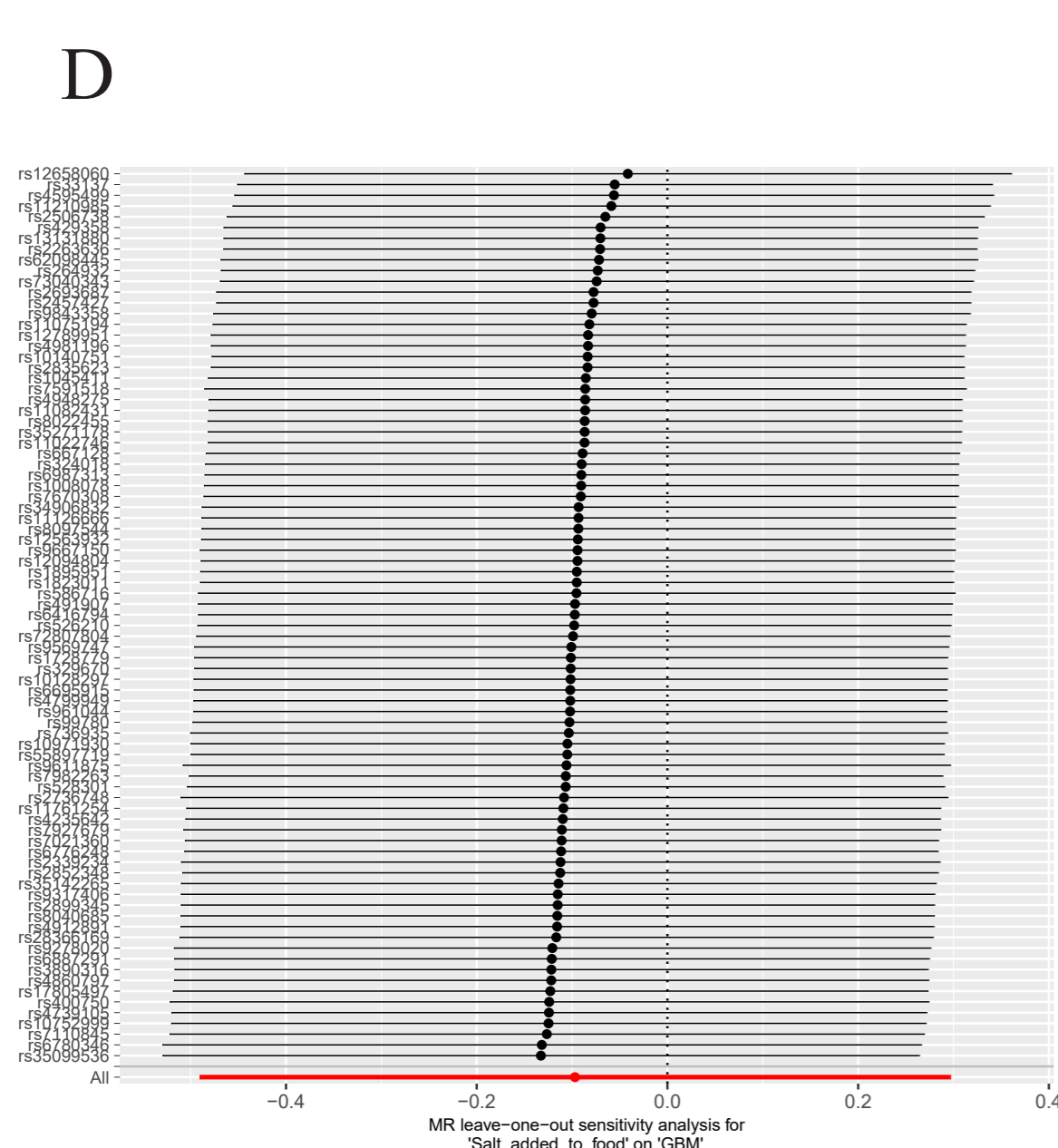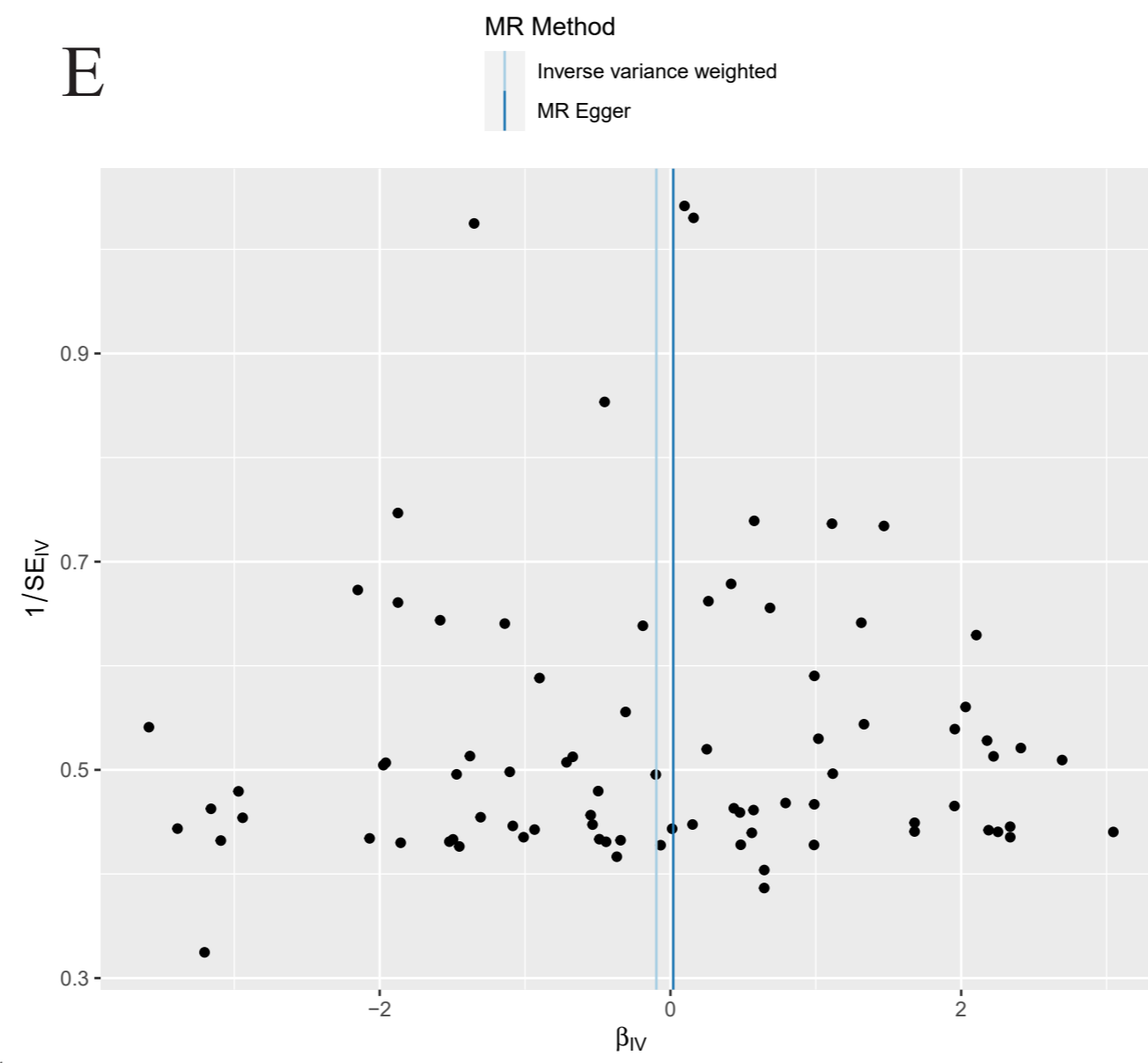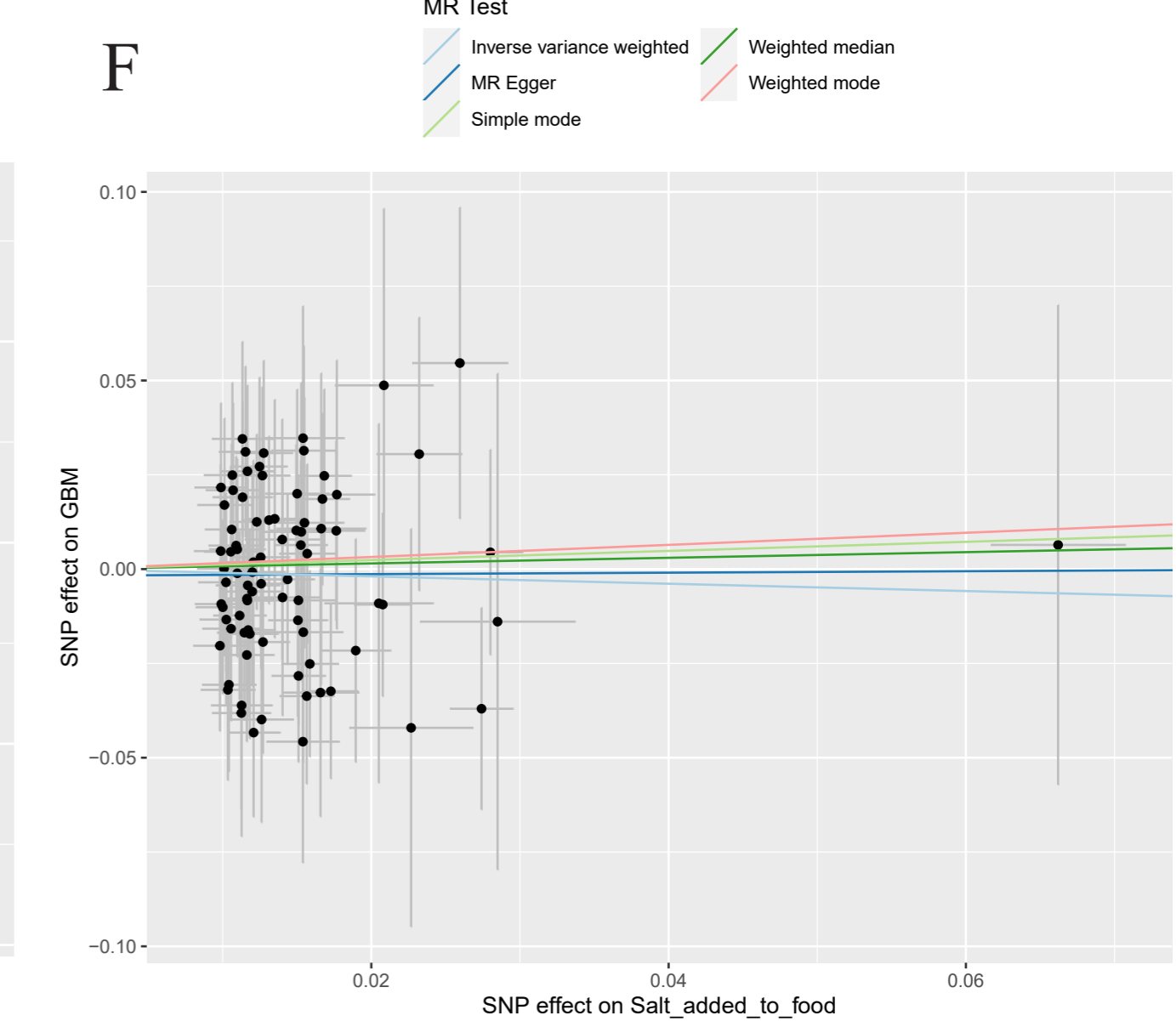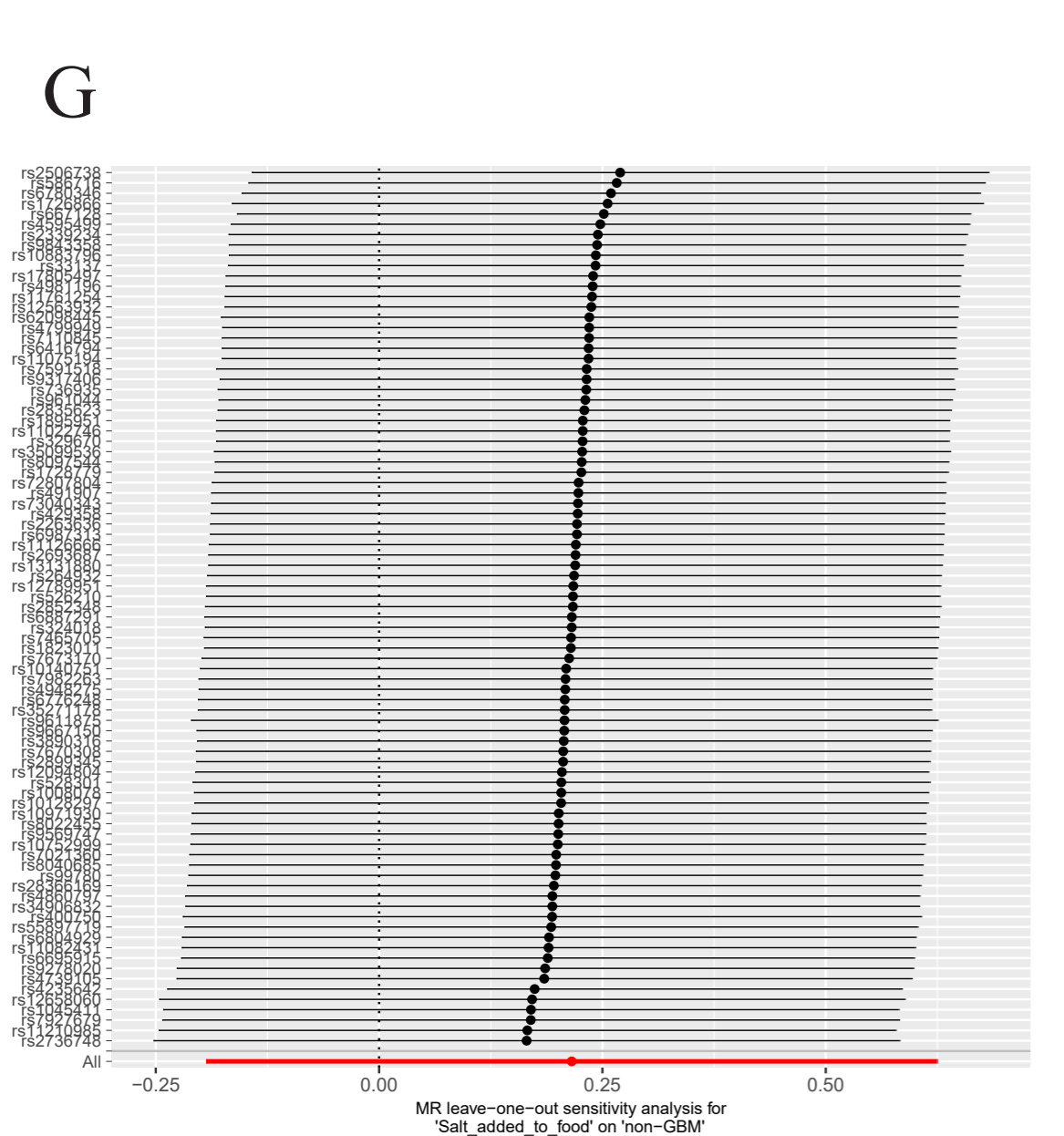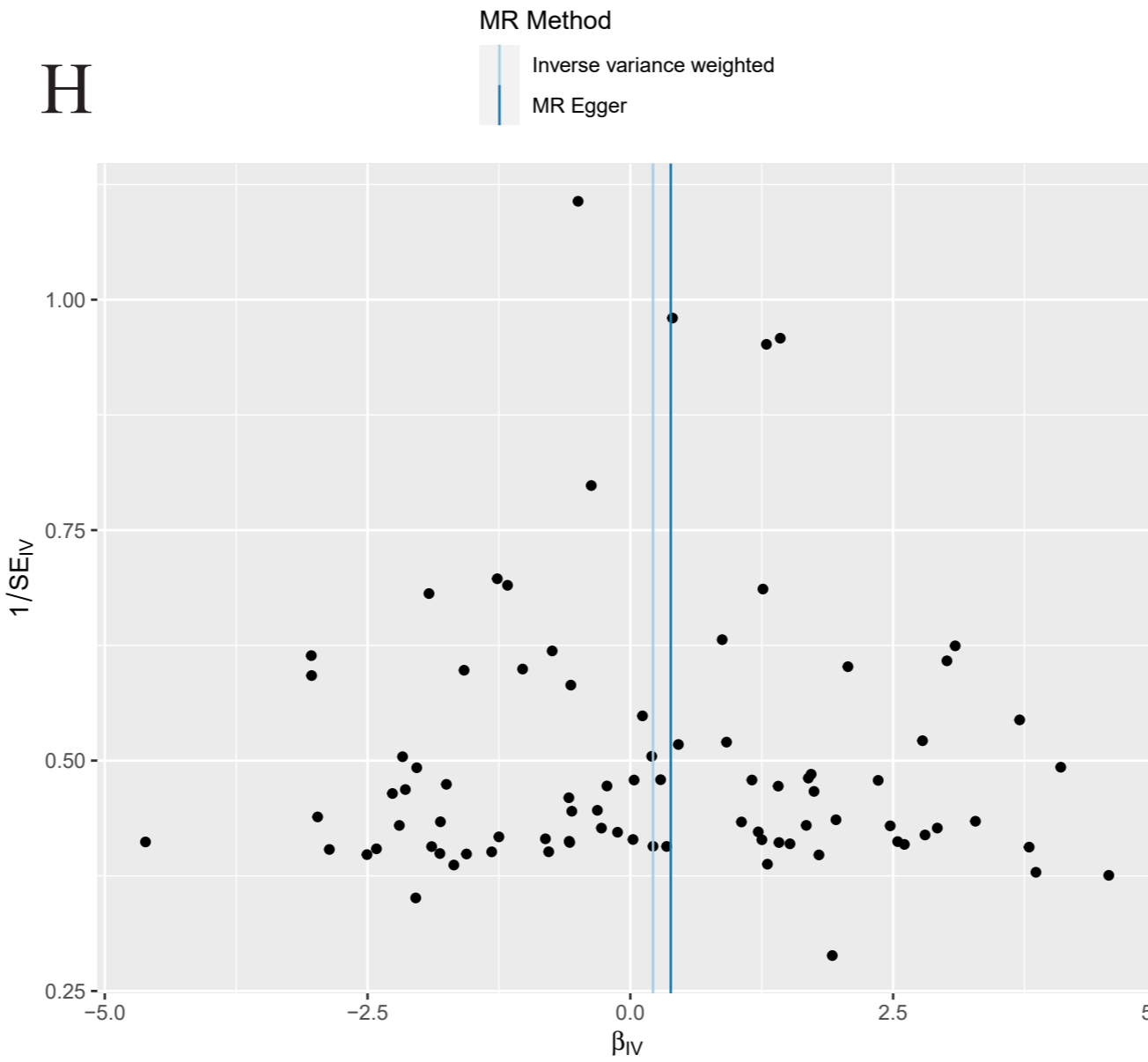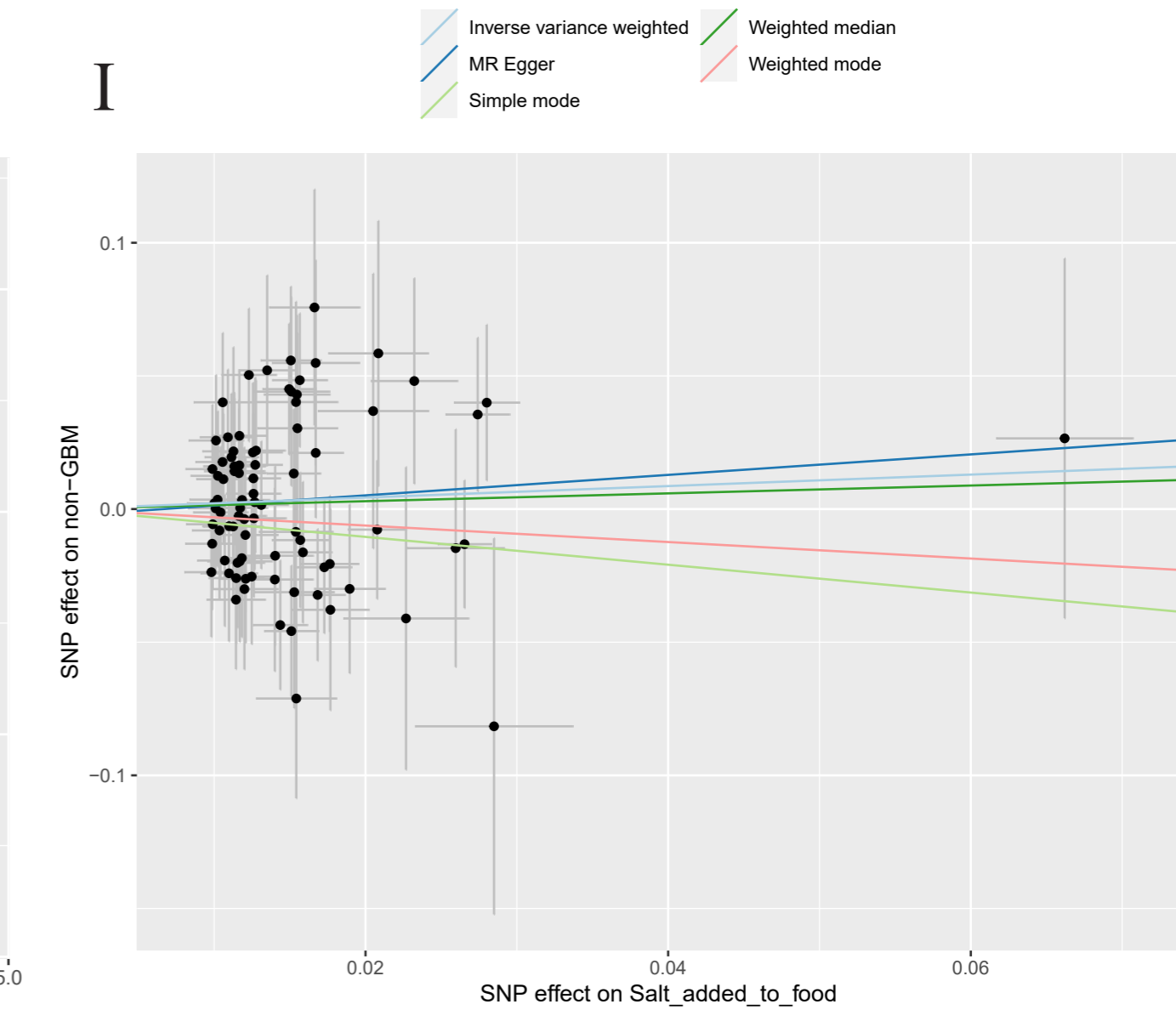

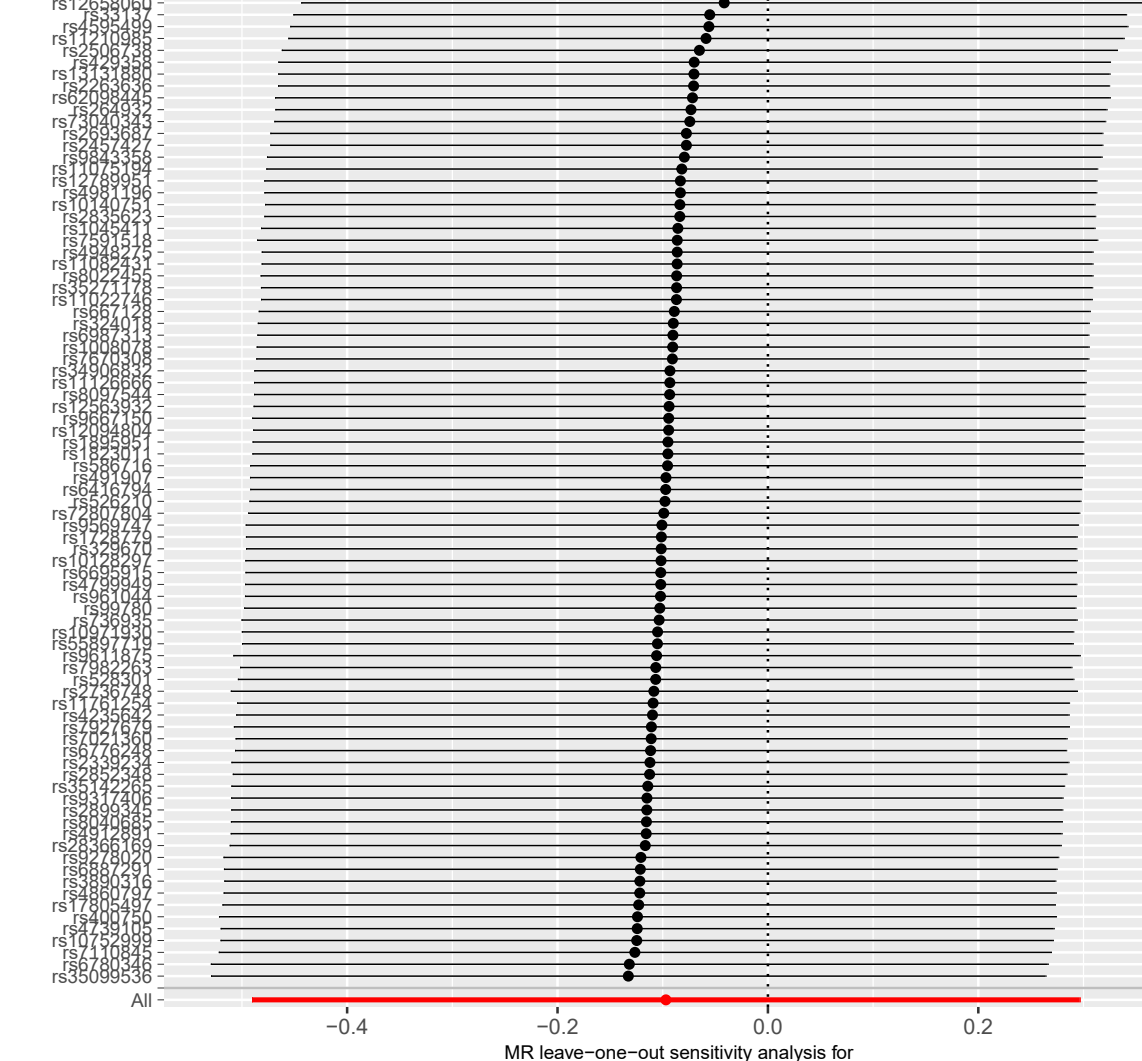

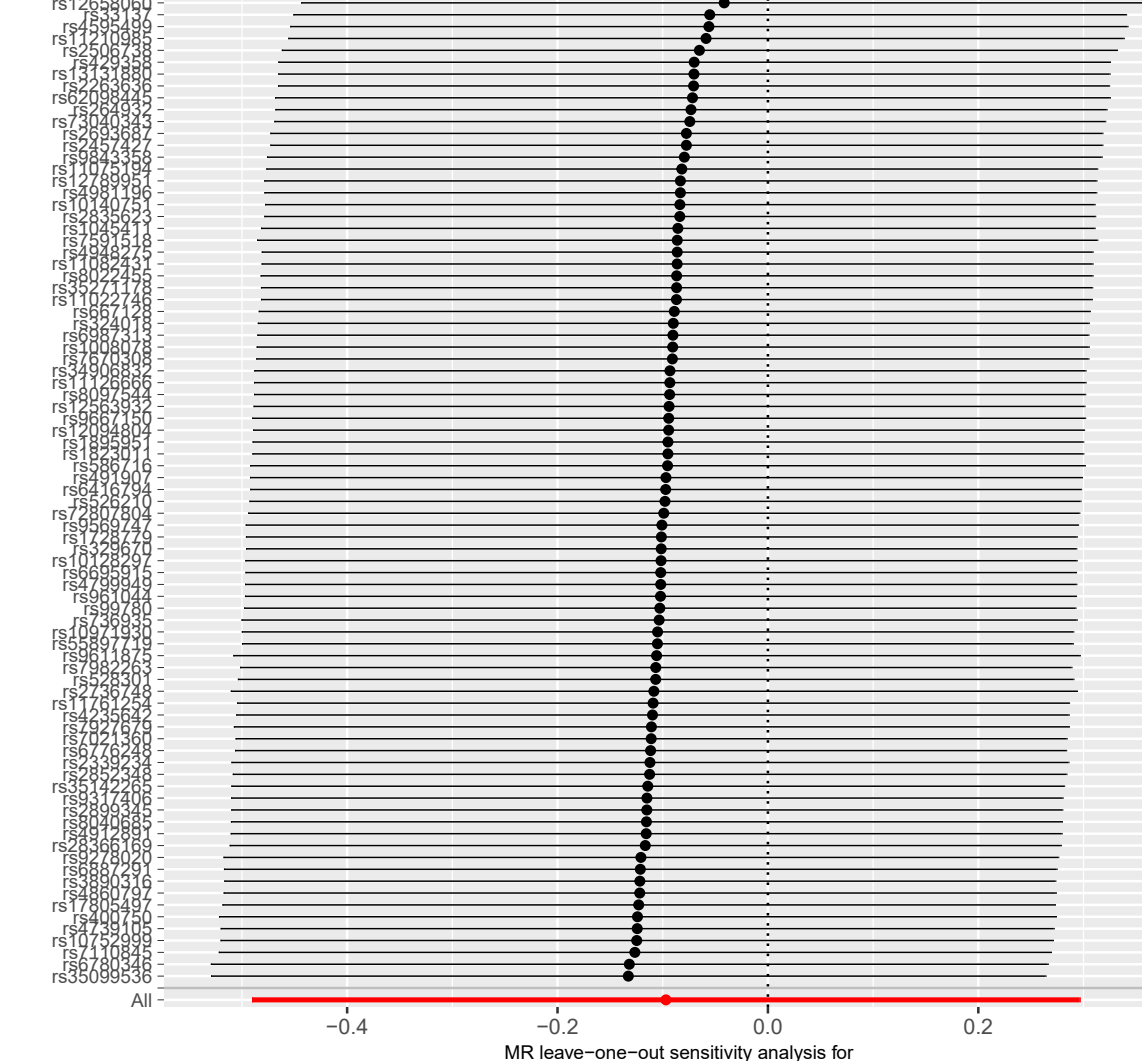

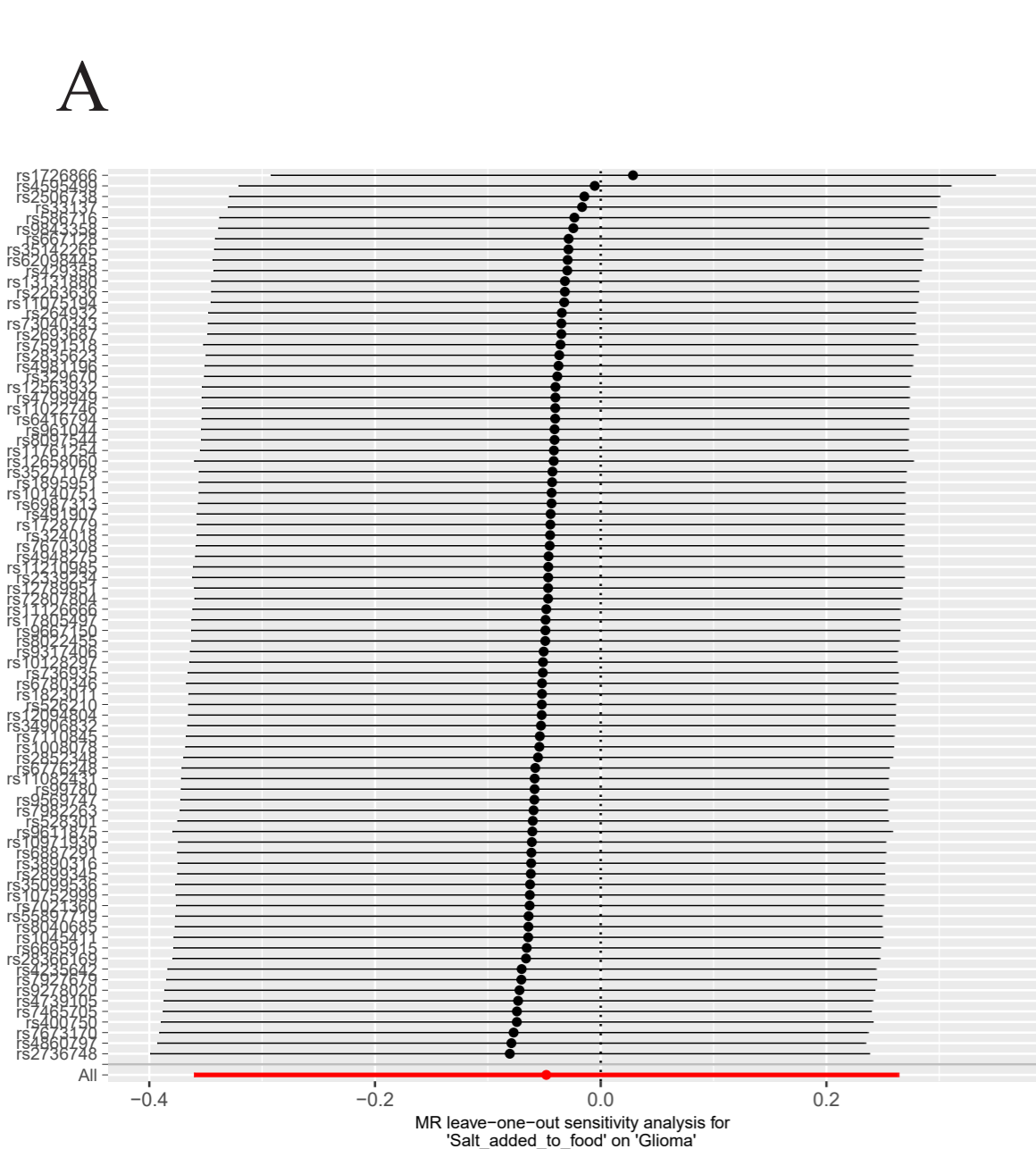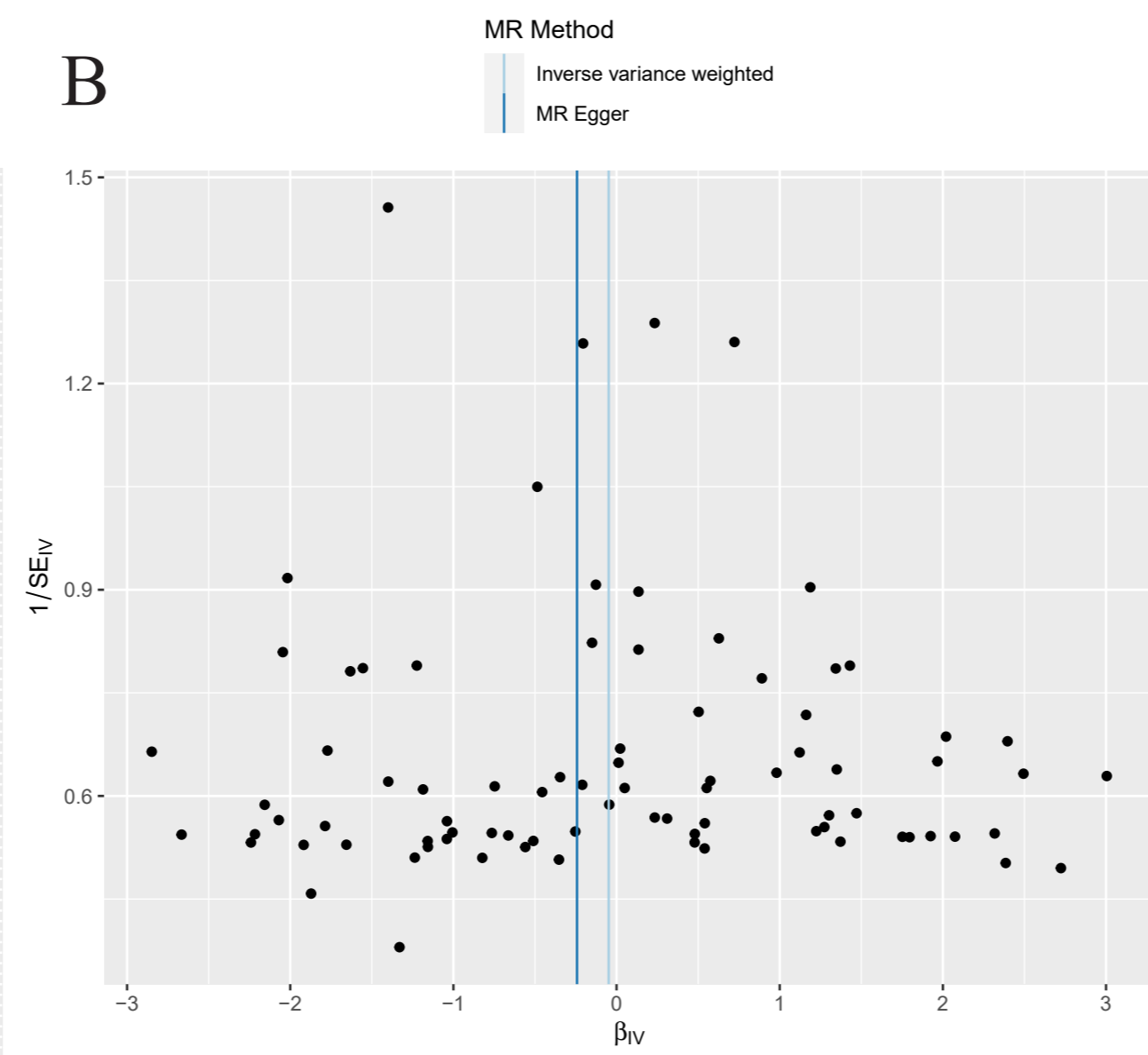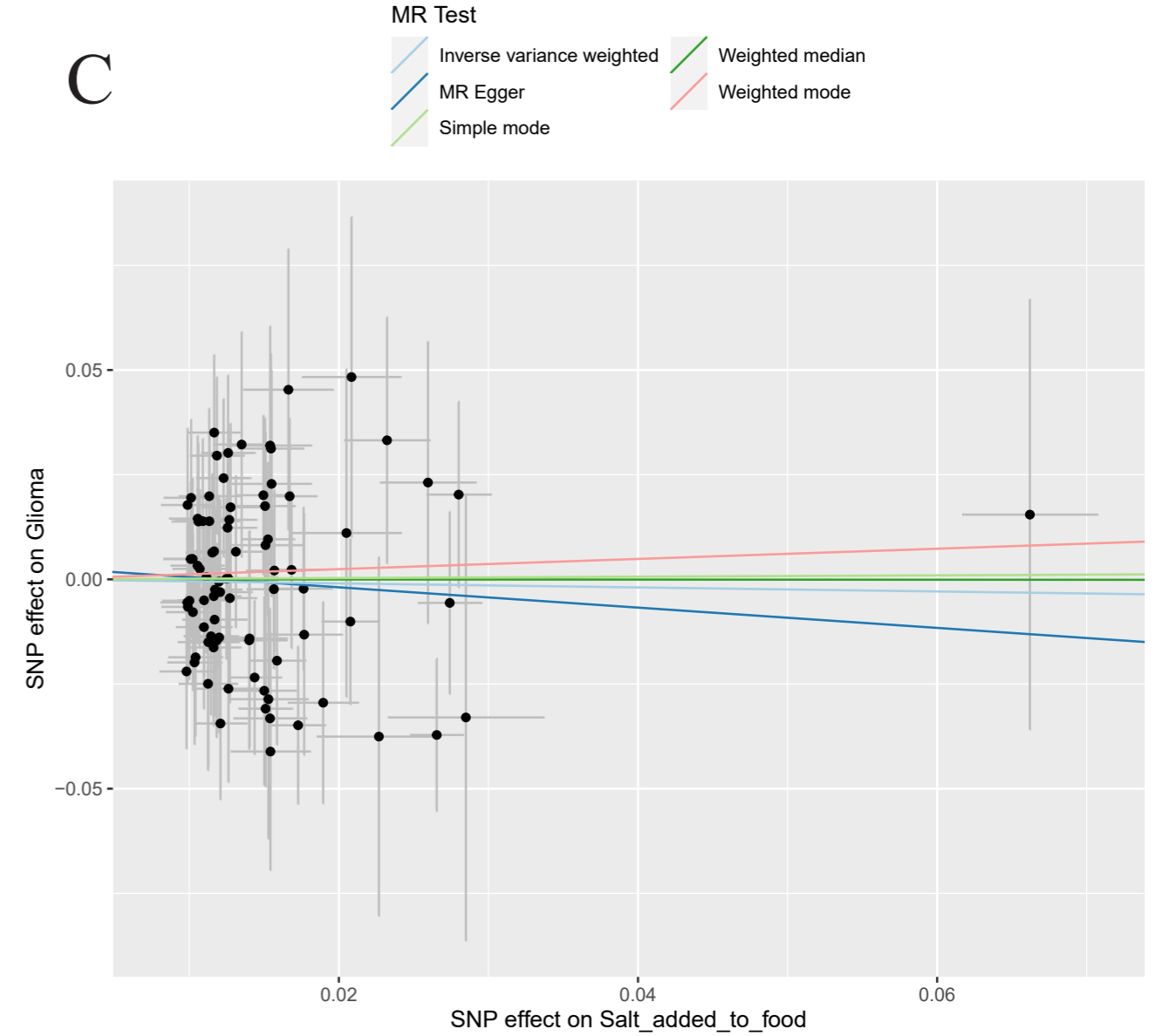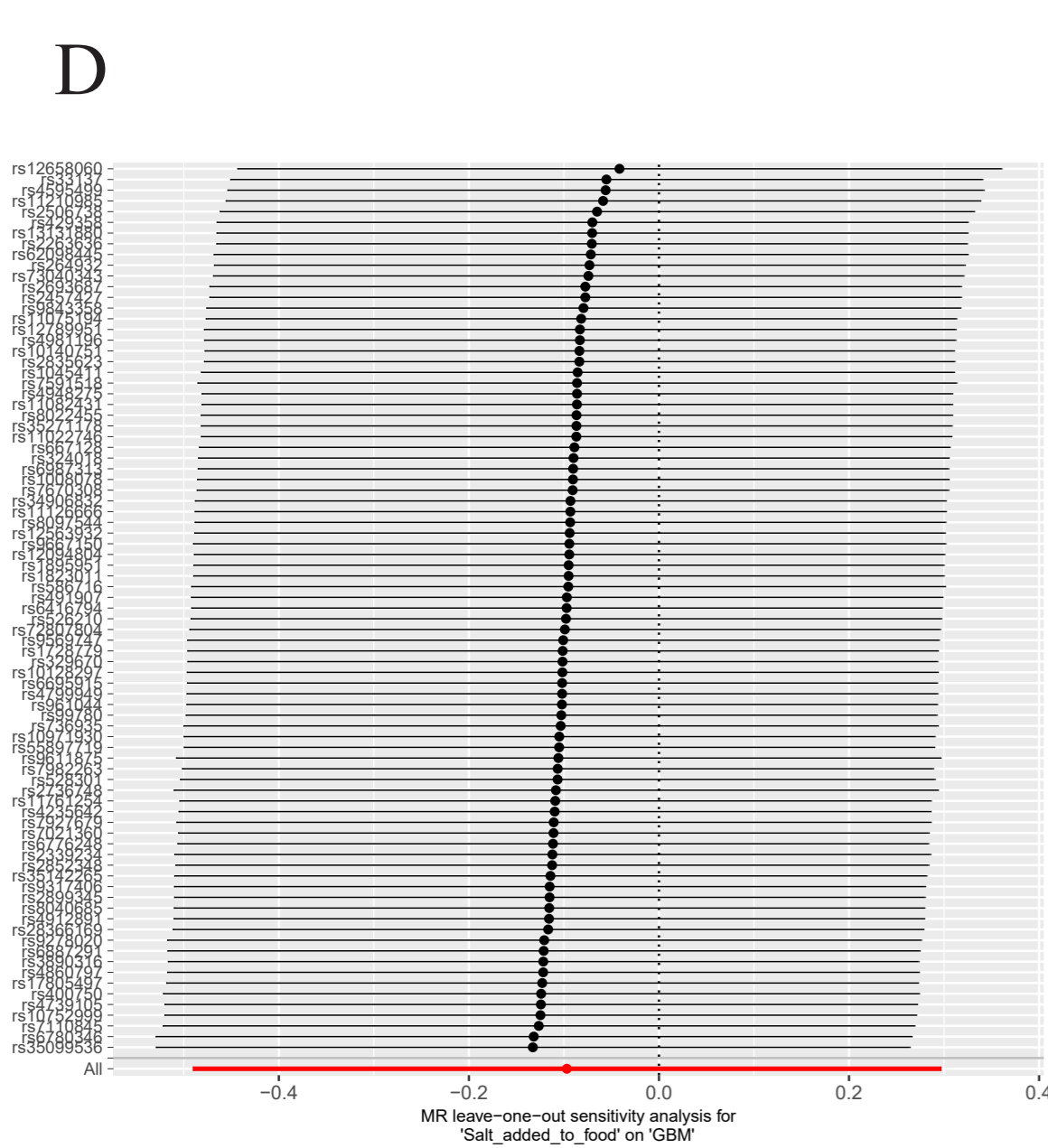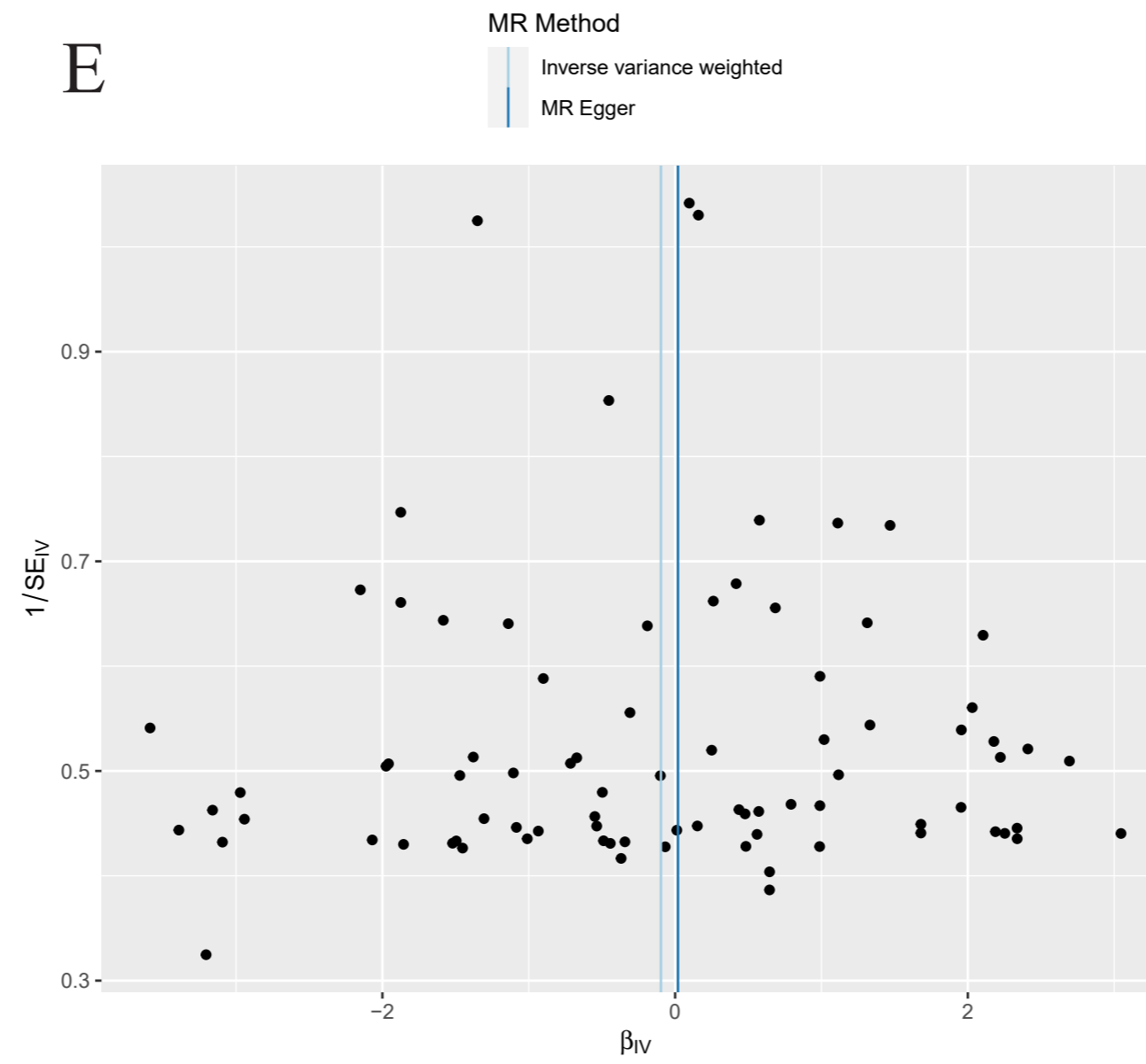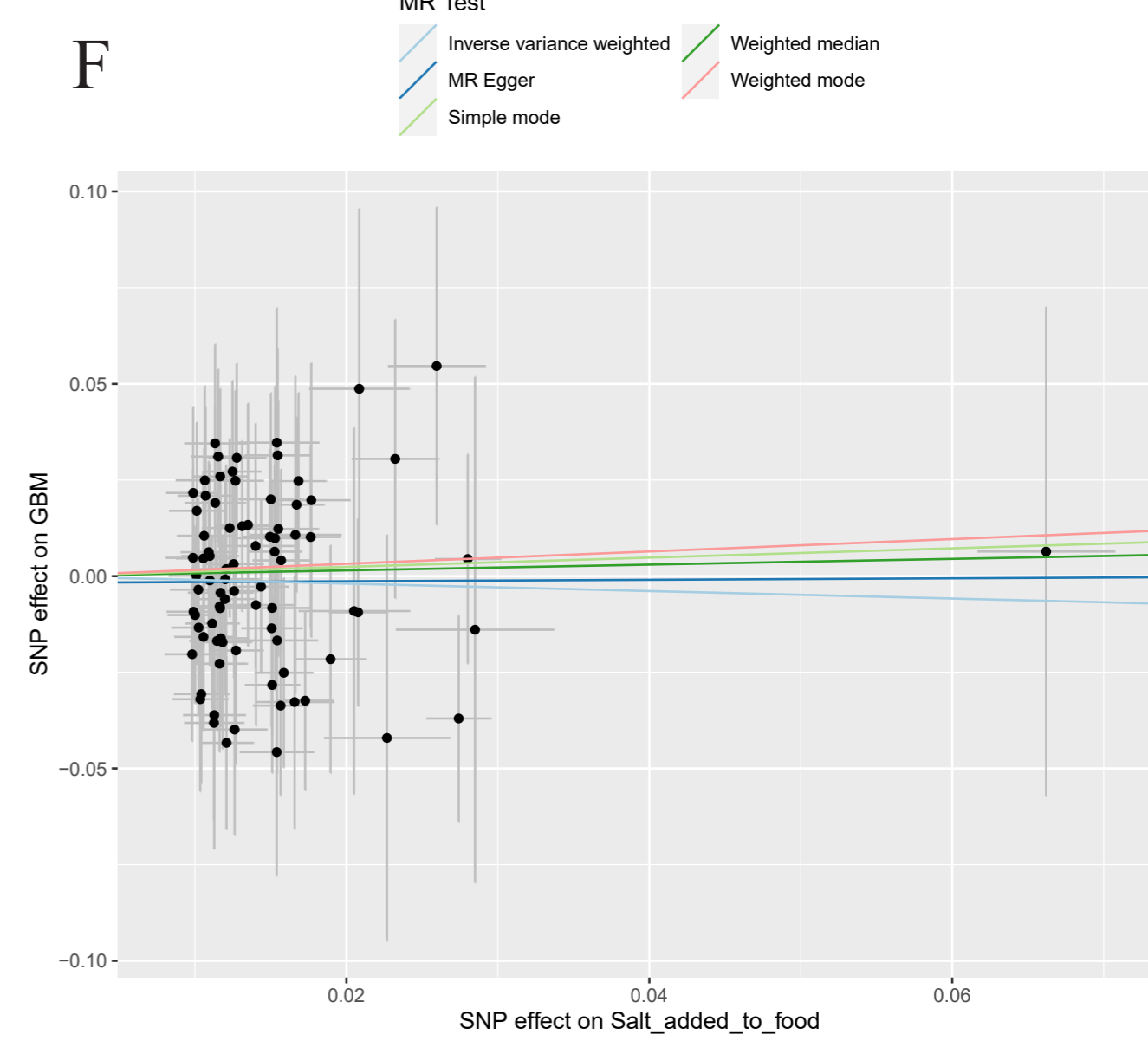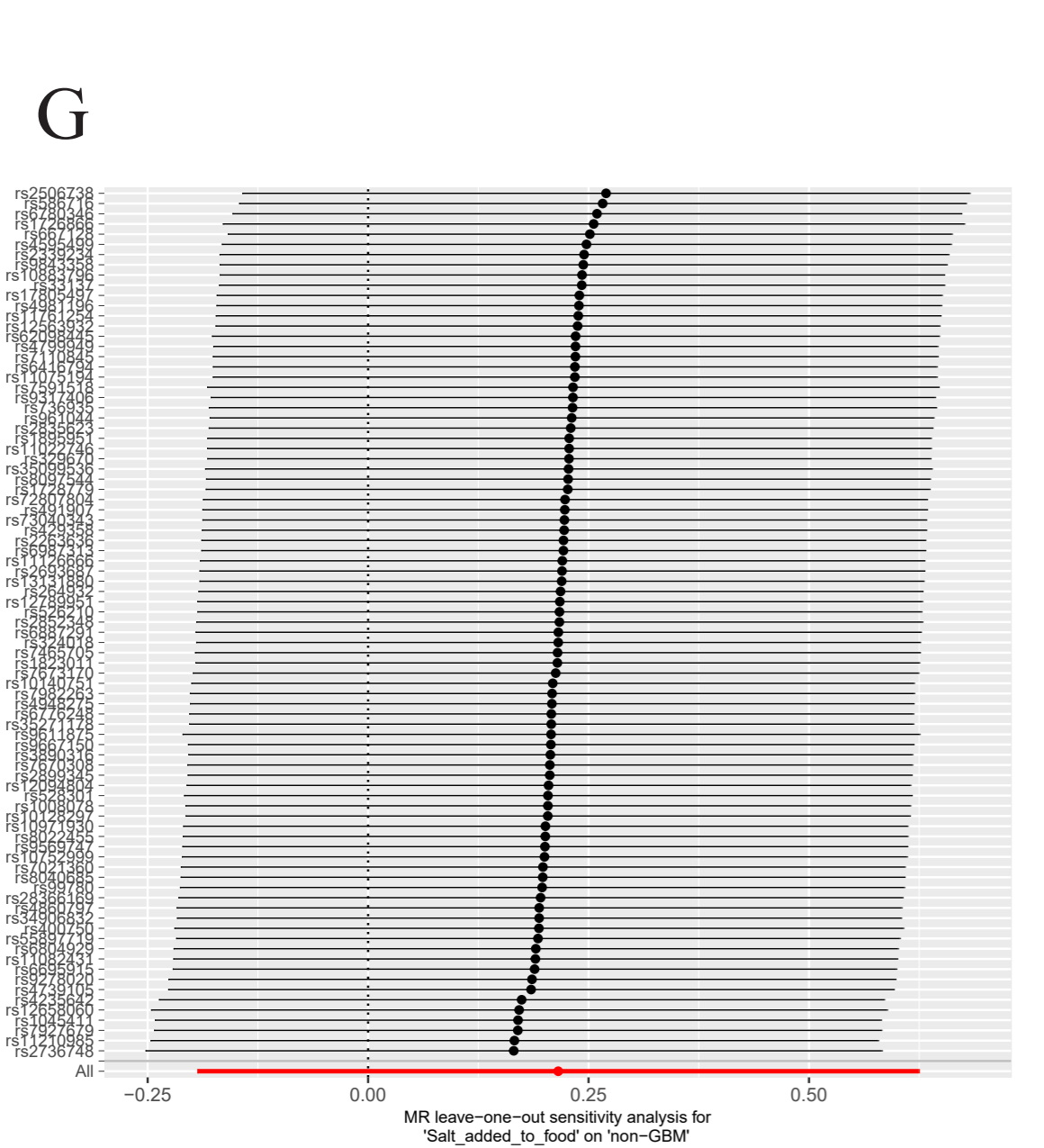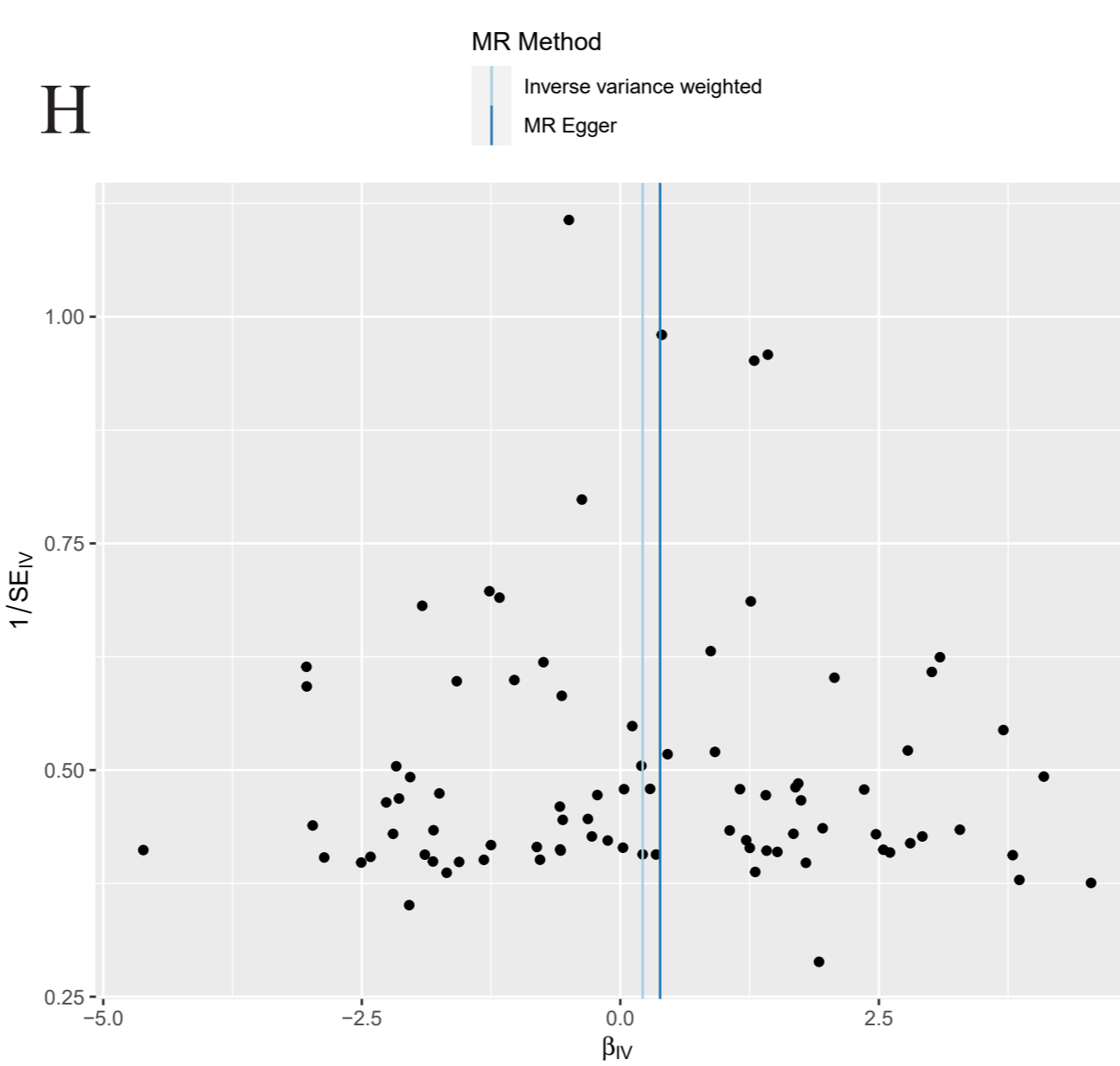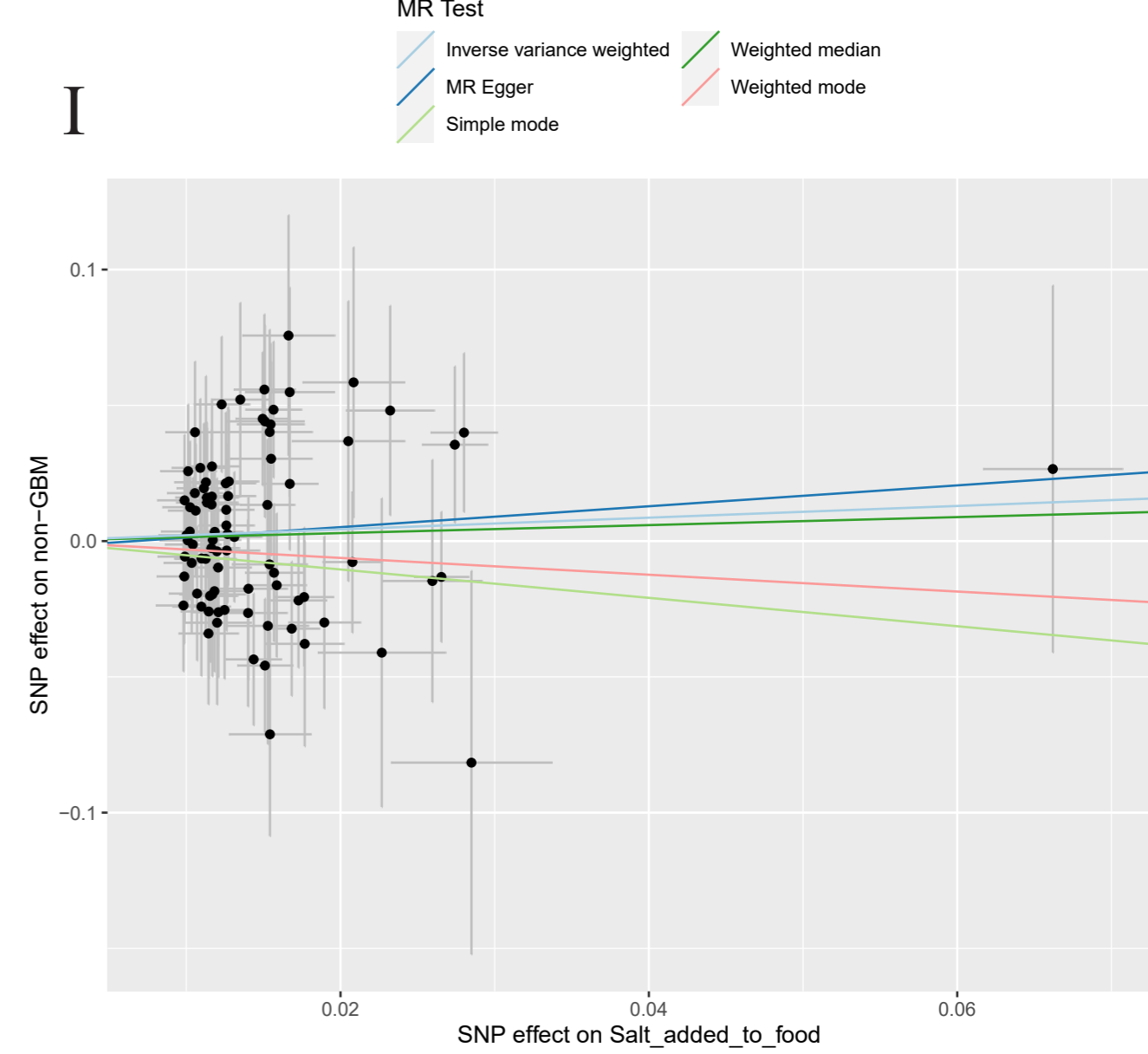

Supplement: Supplementary file 1 [file nutrients-17-00582-s001.zip › nutrients-3462880-supplementary/Sup_21.pdf]

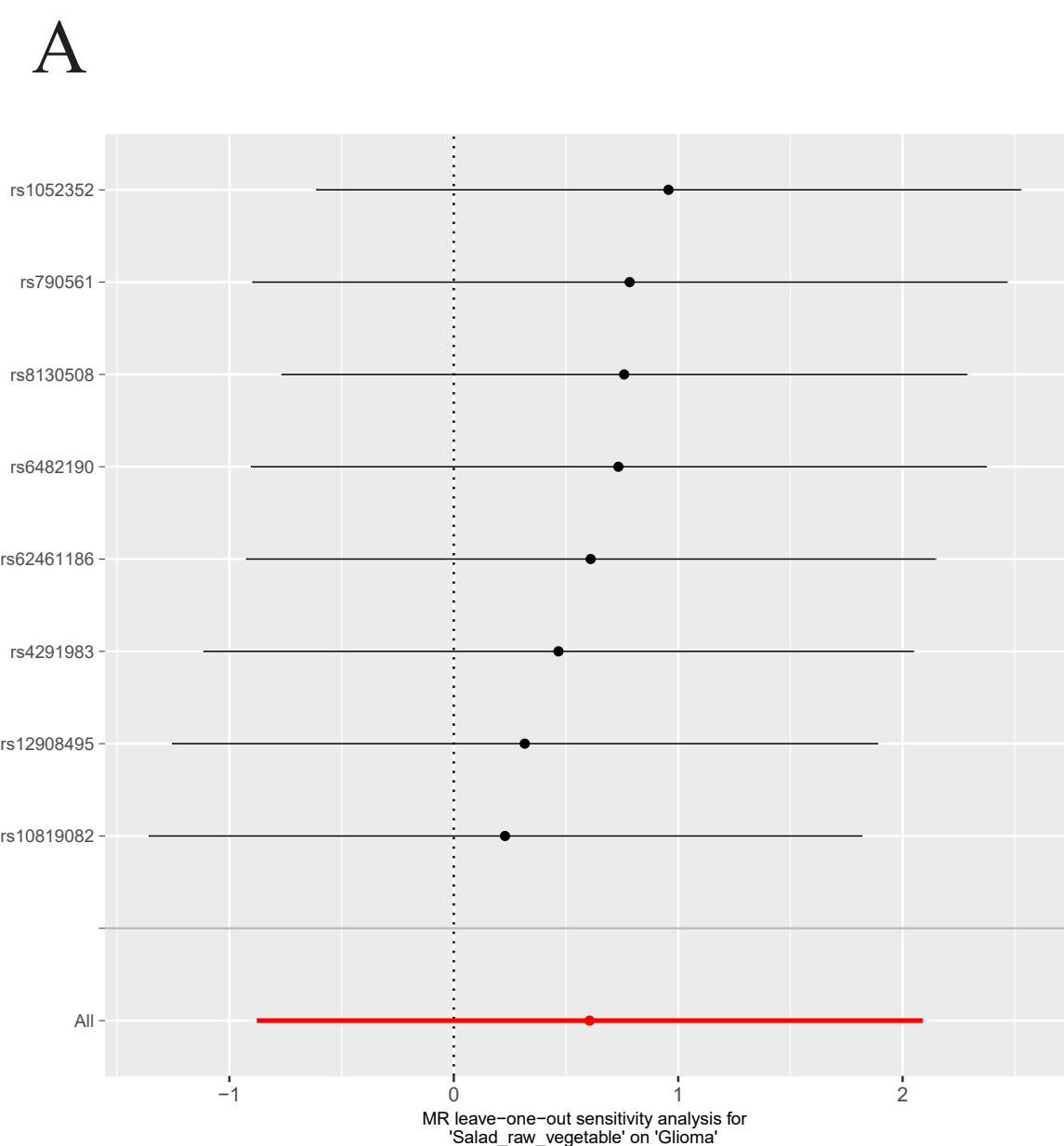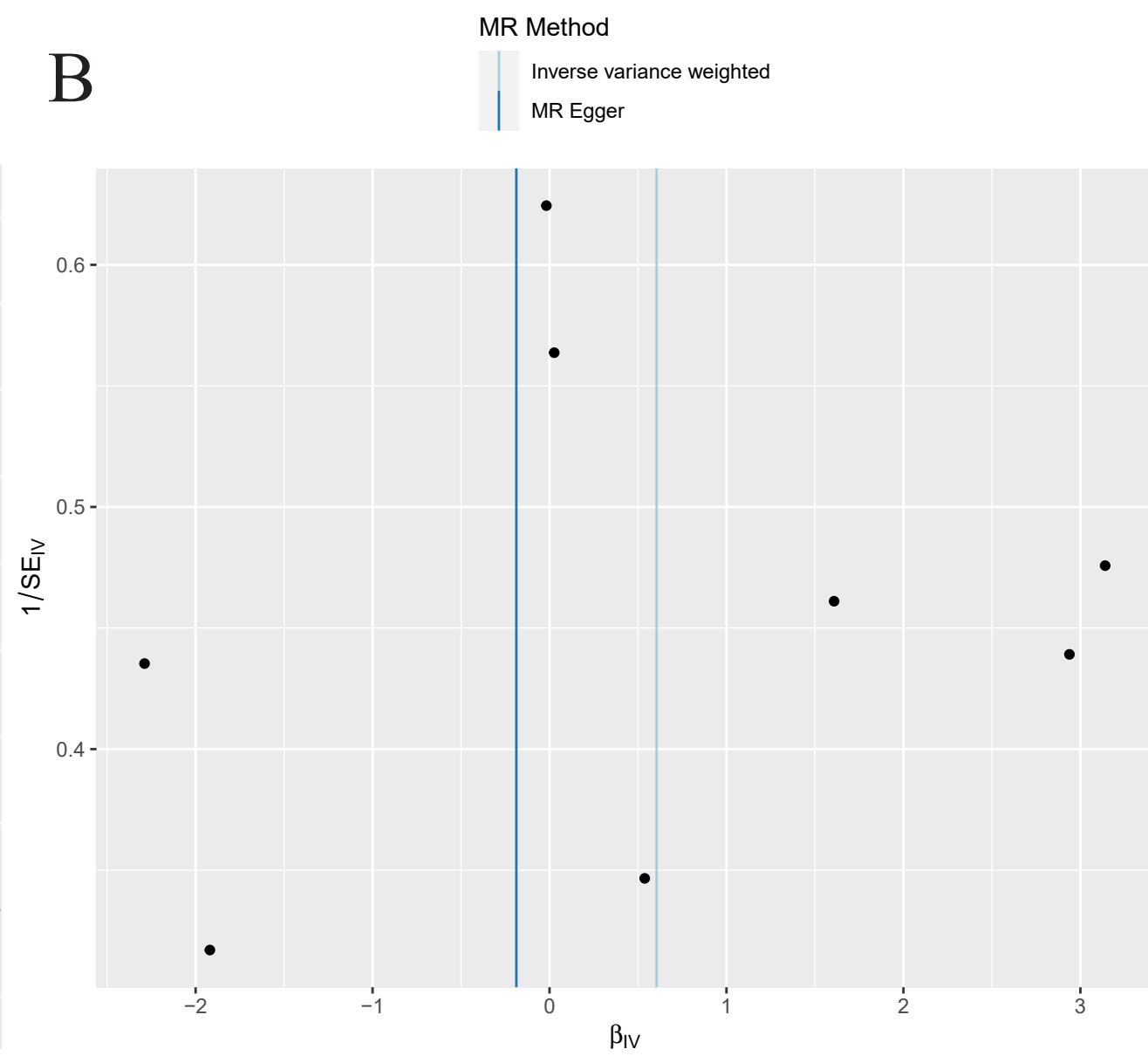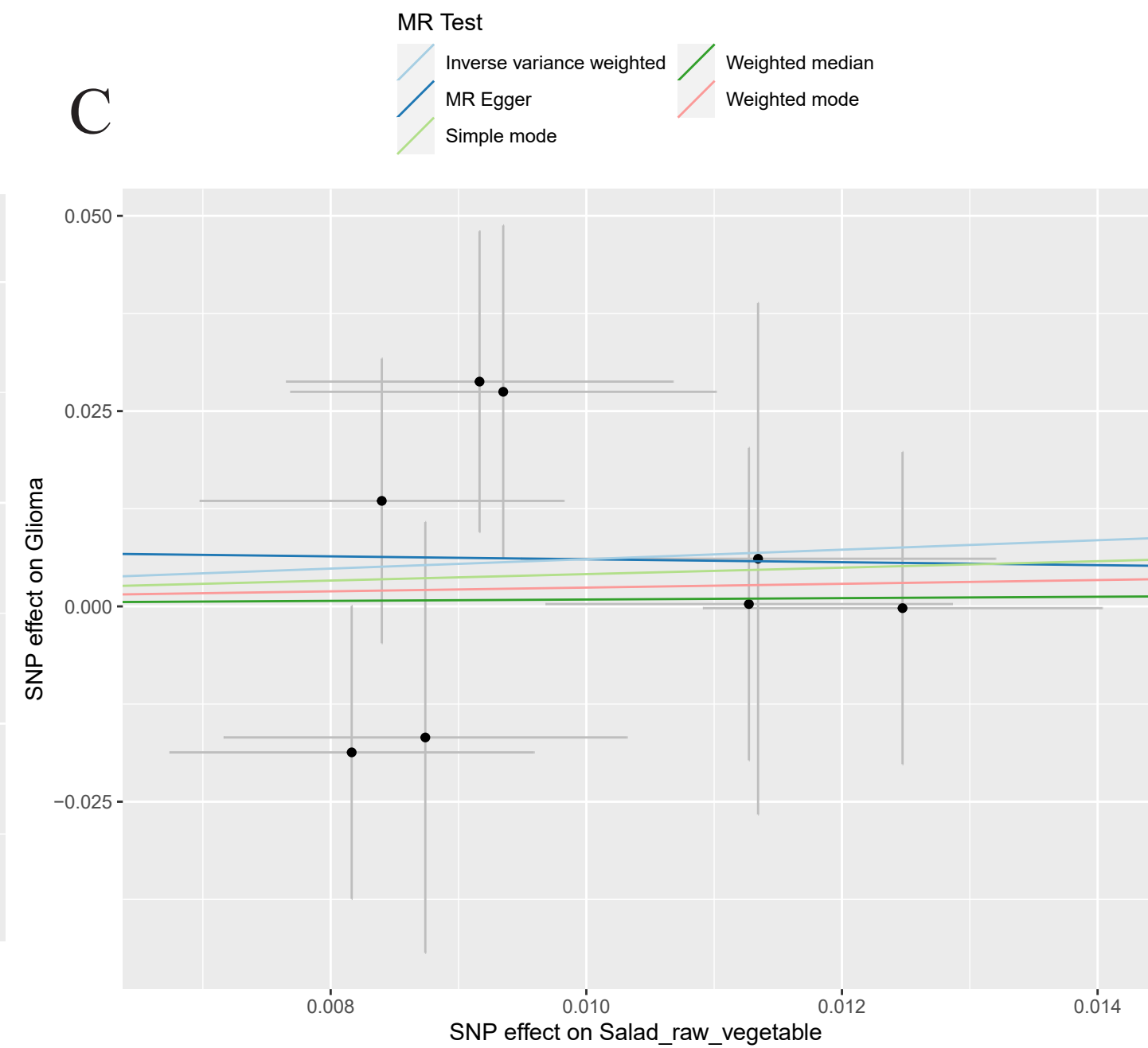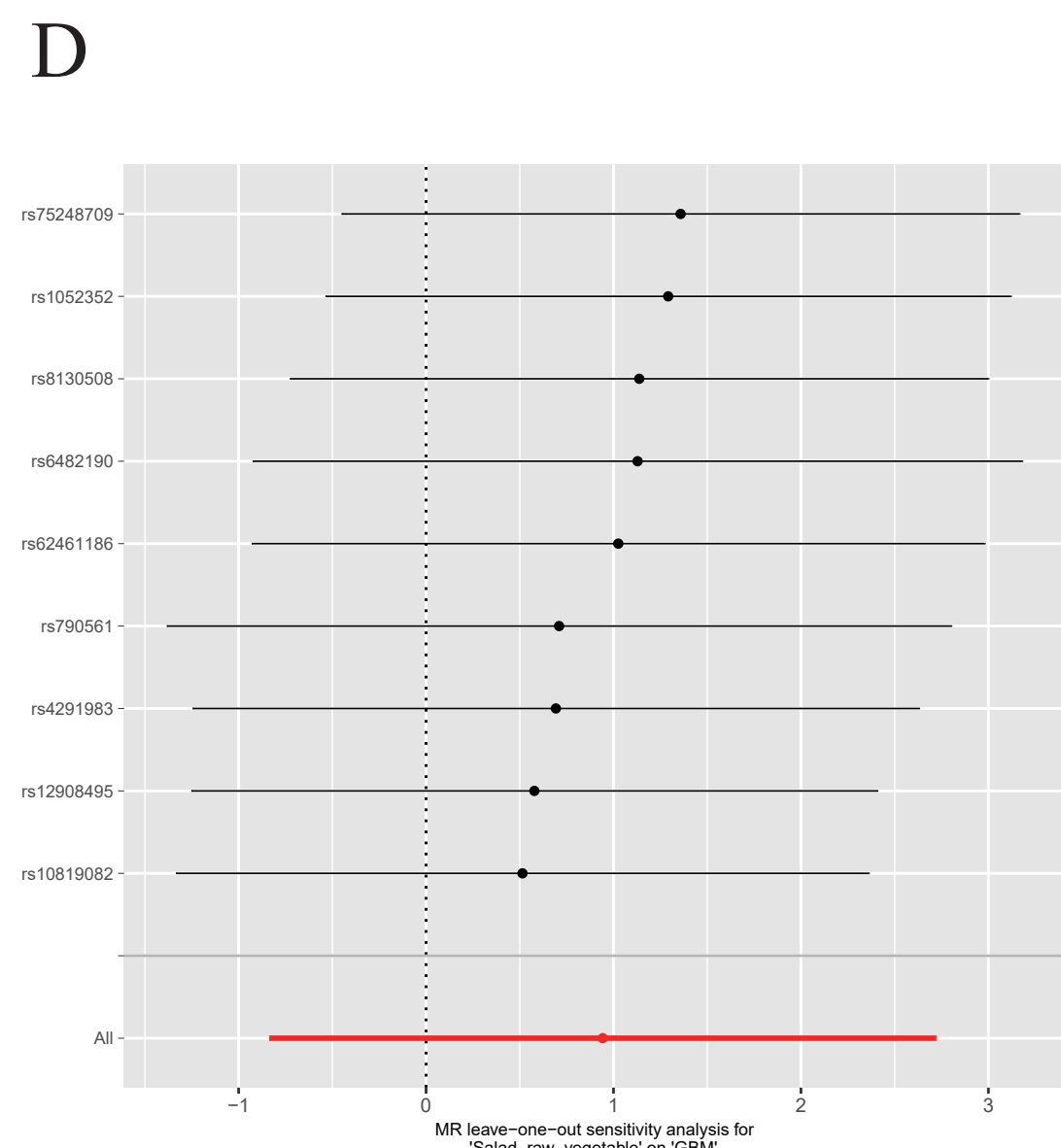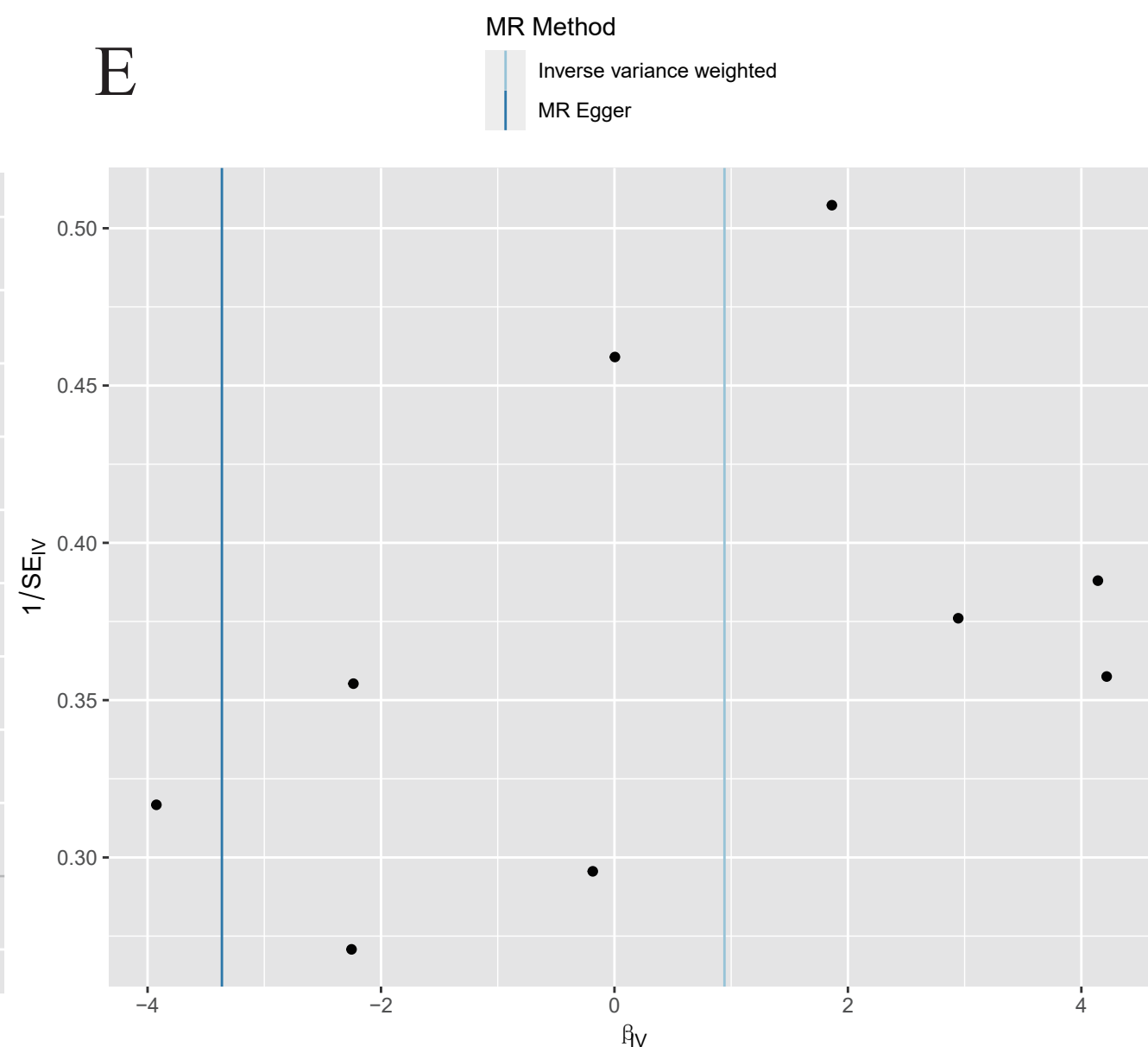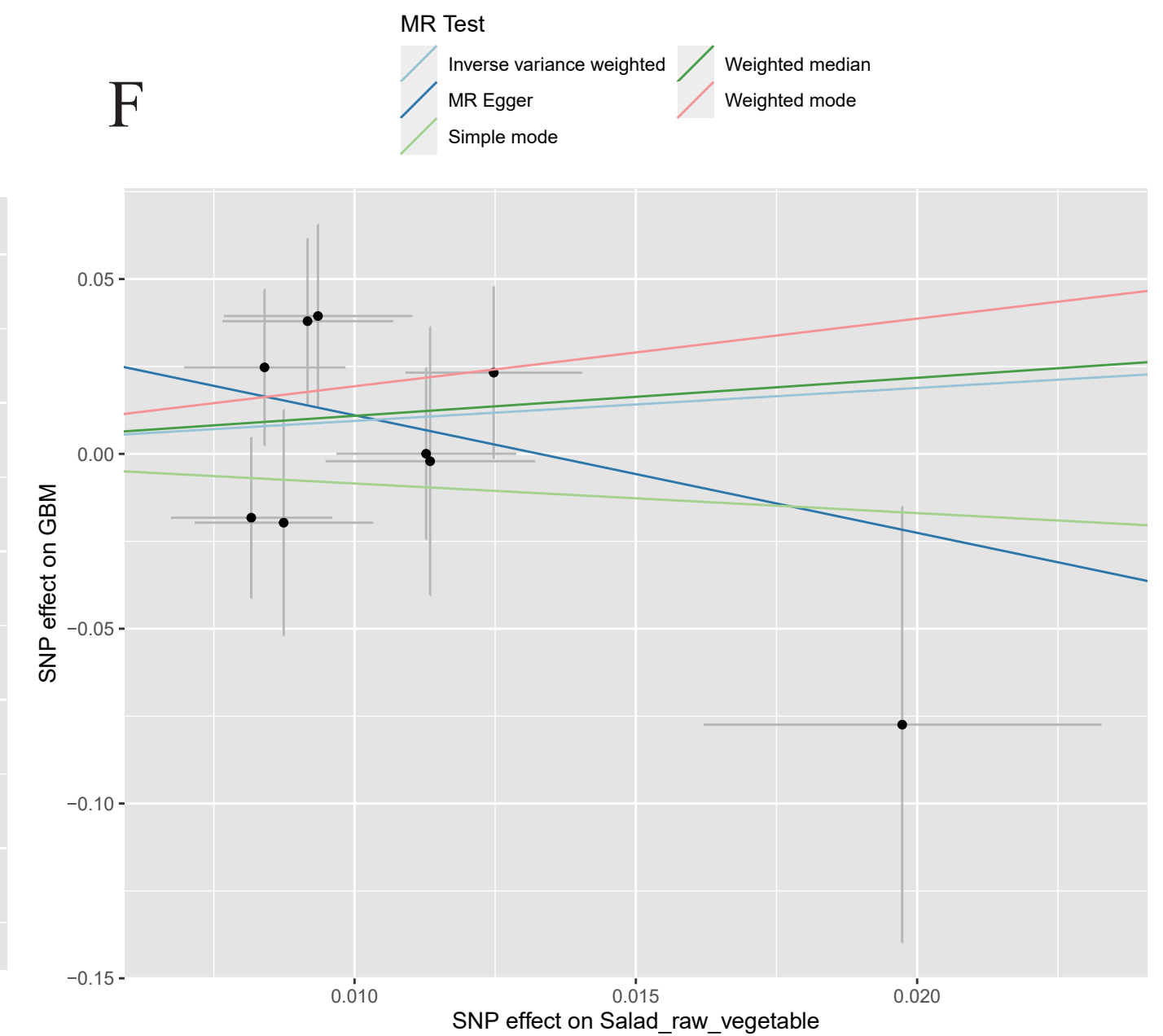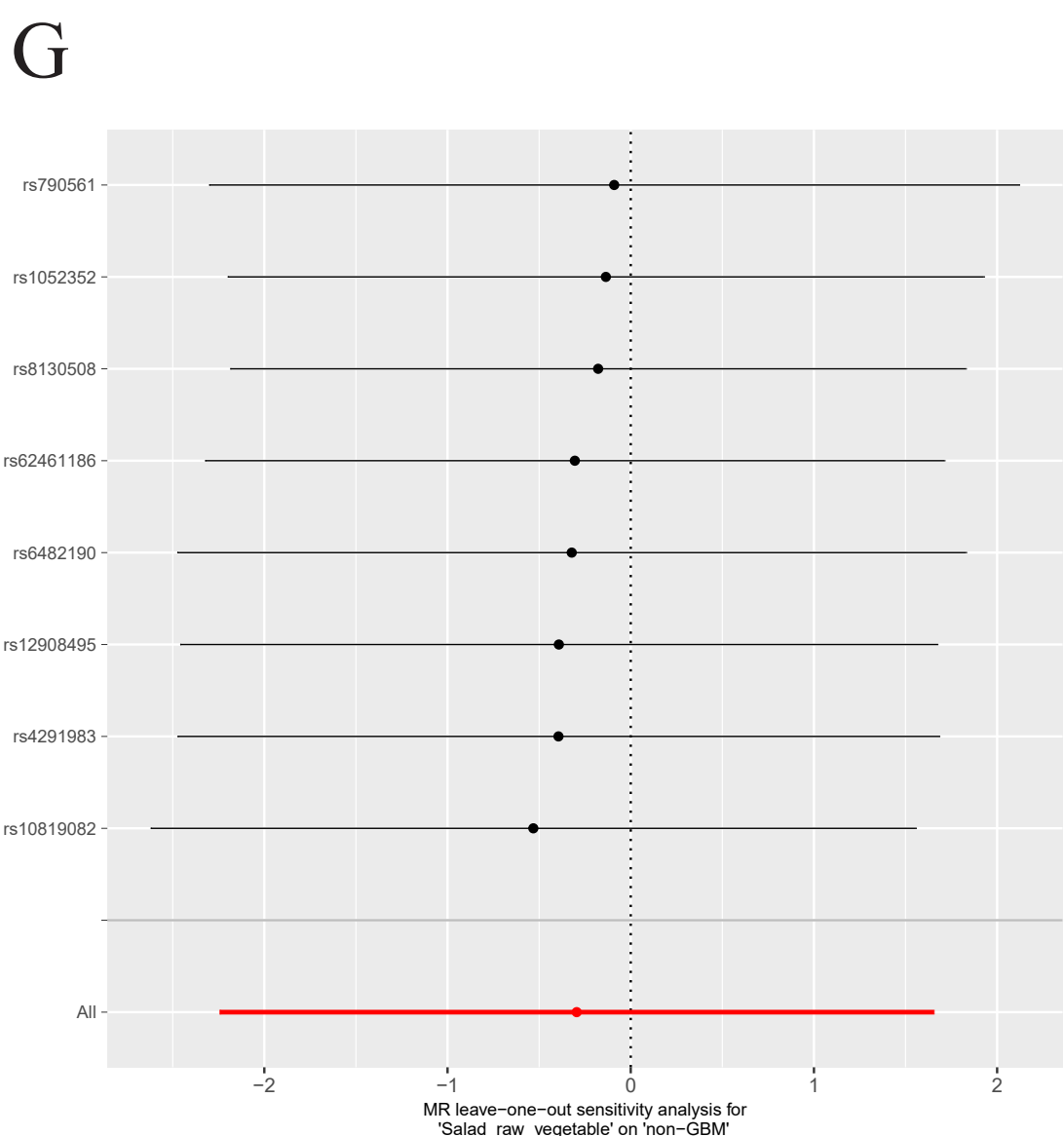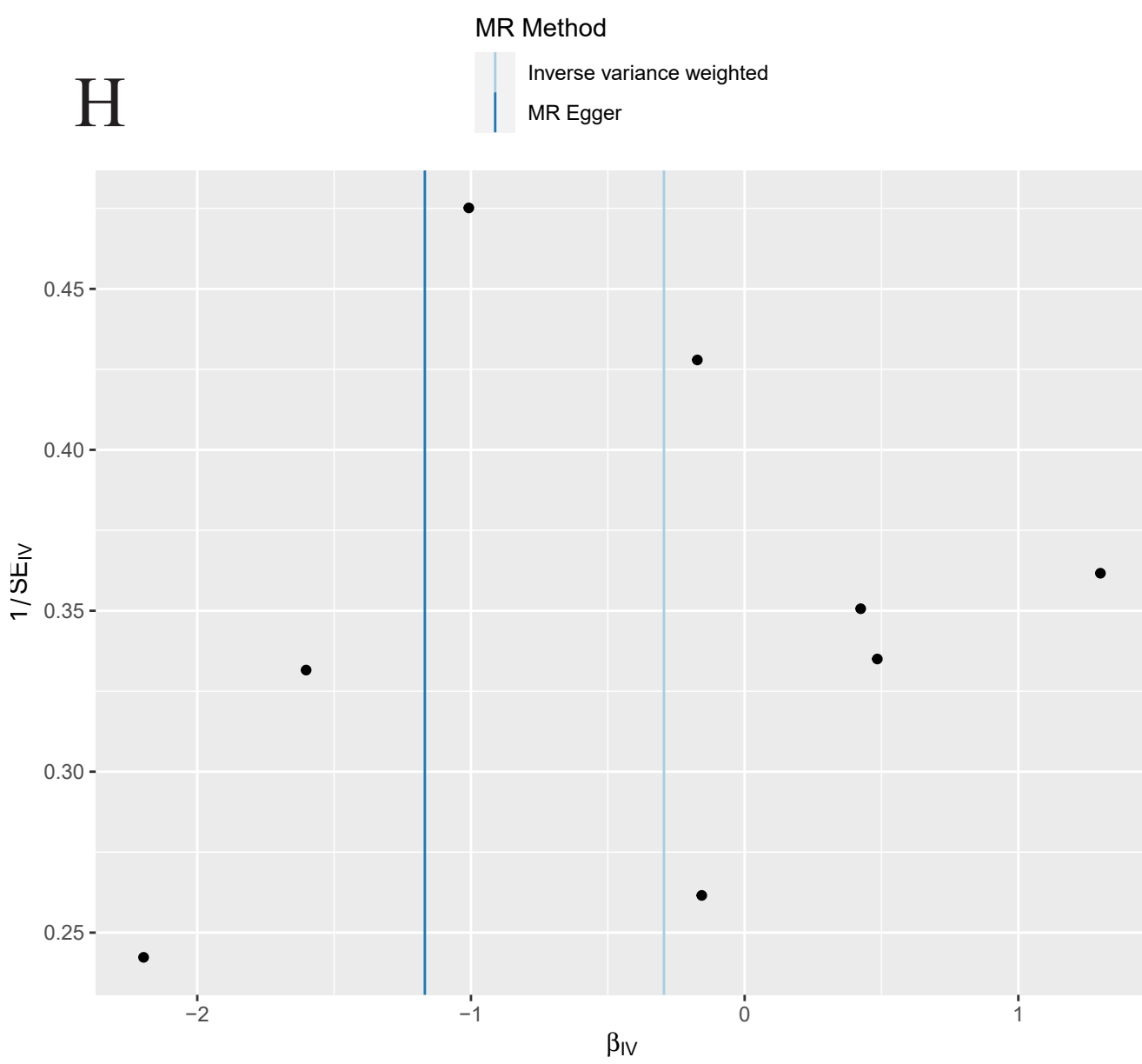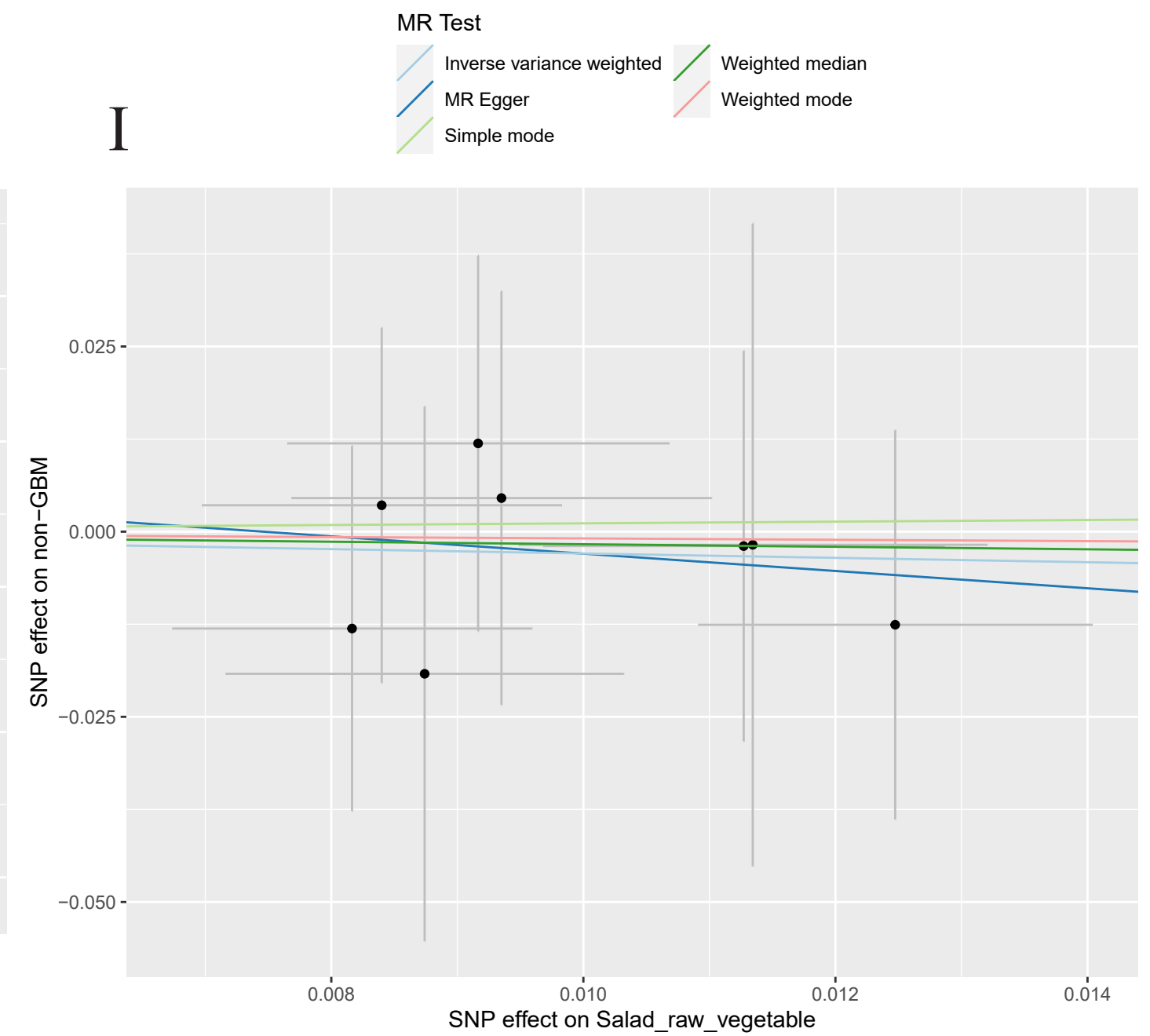

Supplement: Supplementary file 1 [file nutrients-17-00582-s001.zip › nutrients-3462880-supplementary/Sup_22.pdf]

A

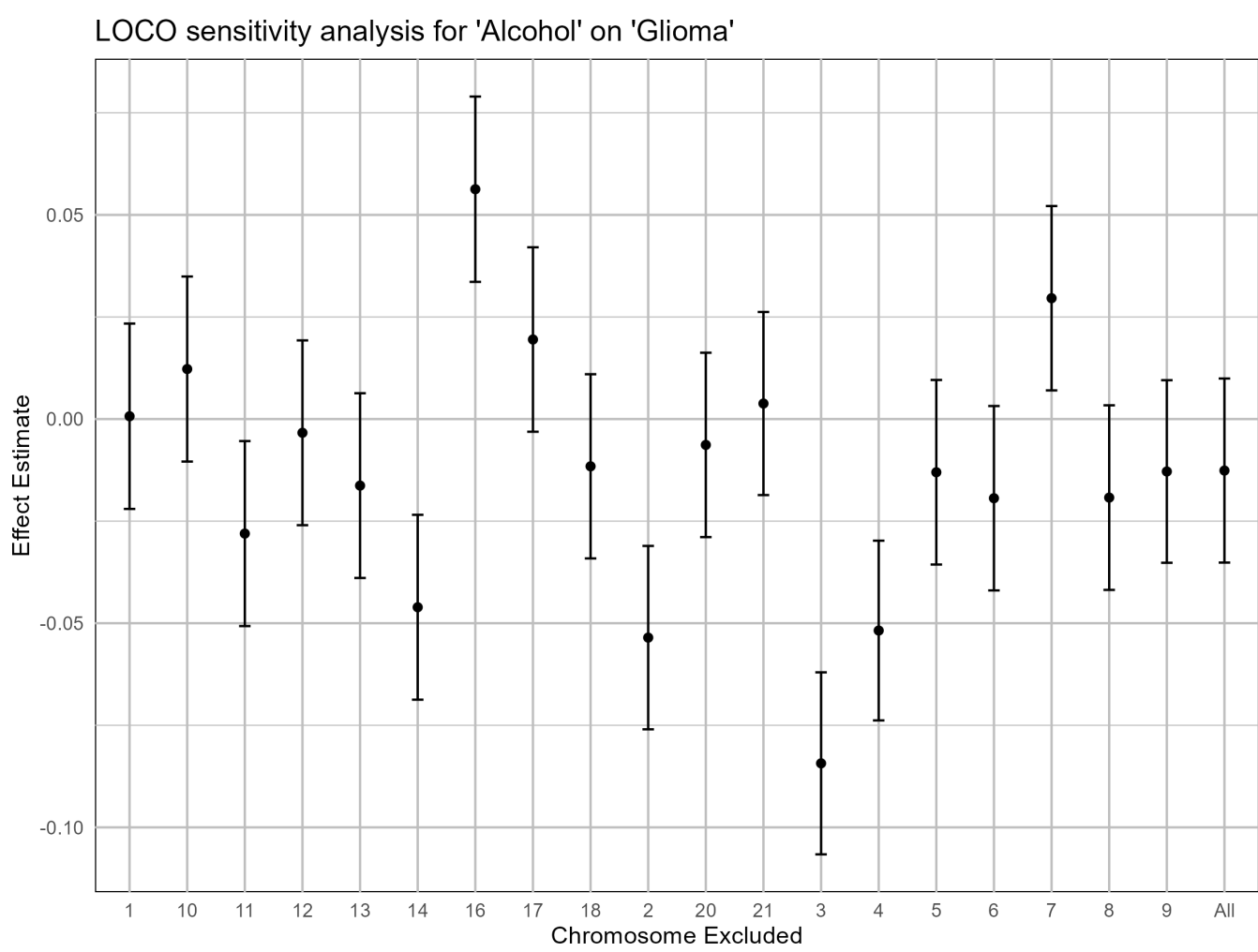

B

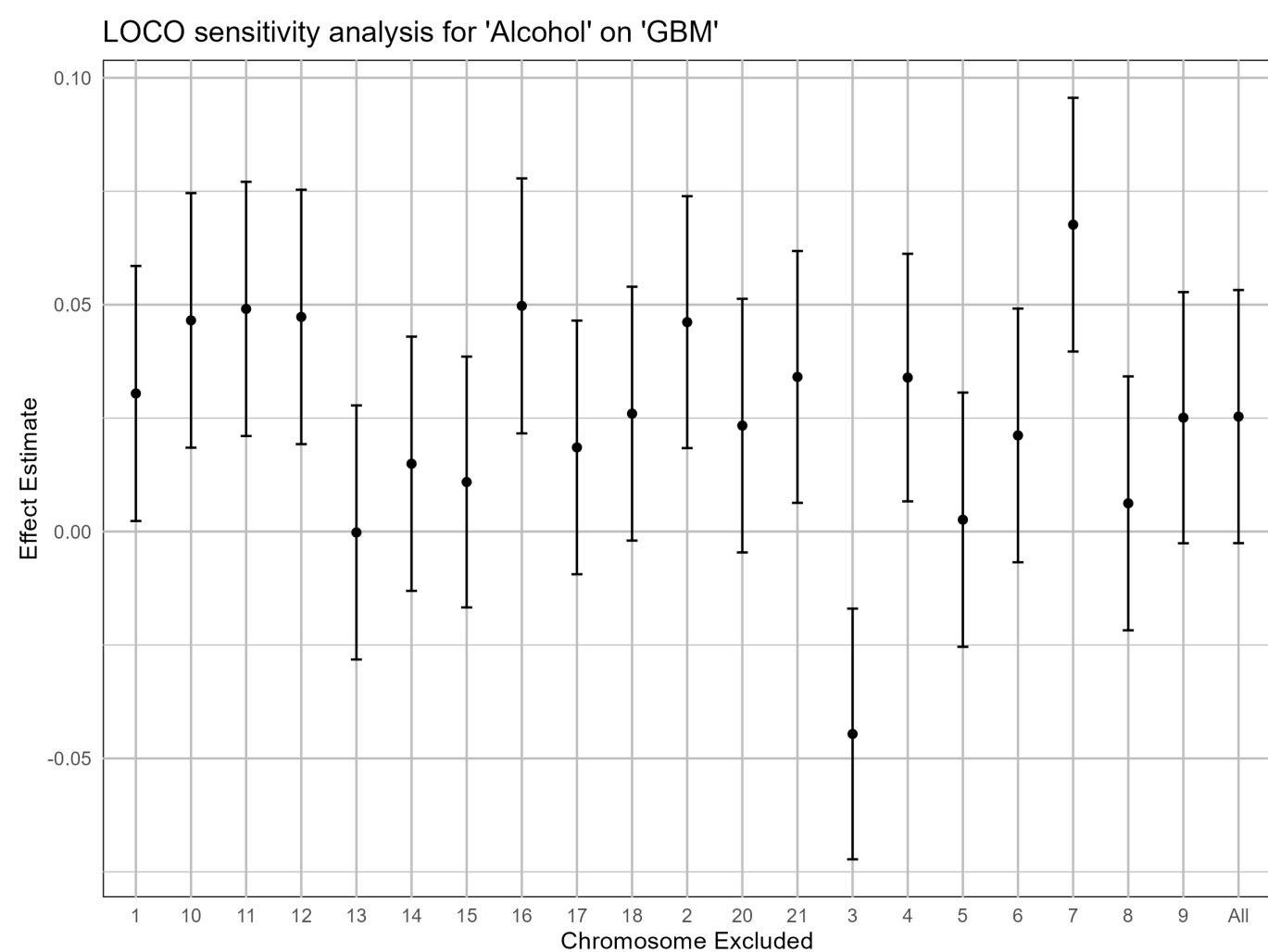

C

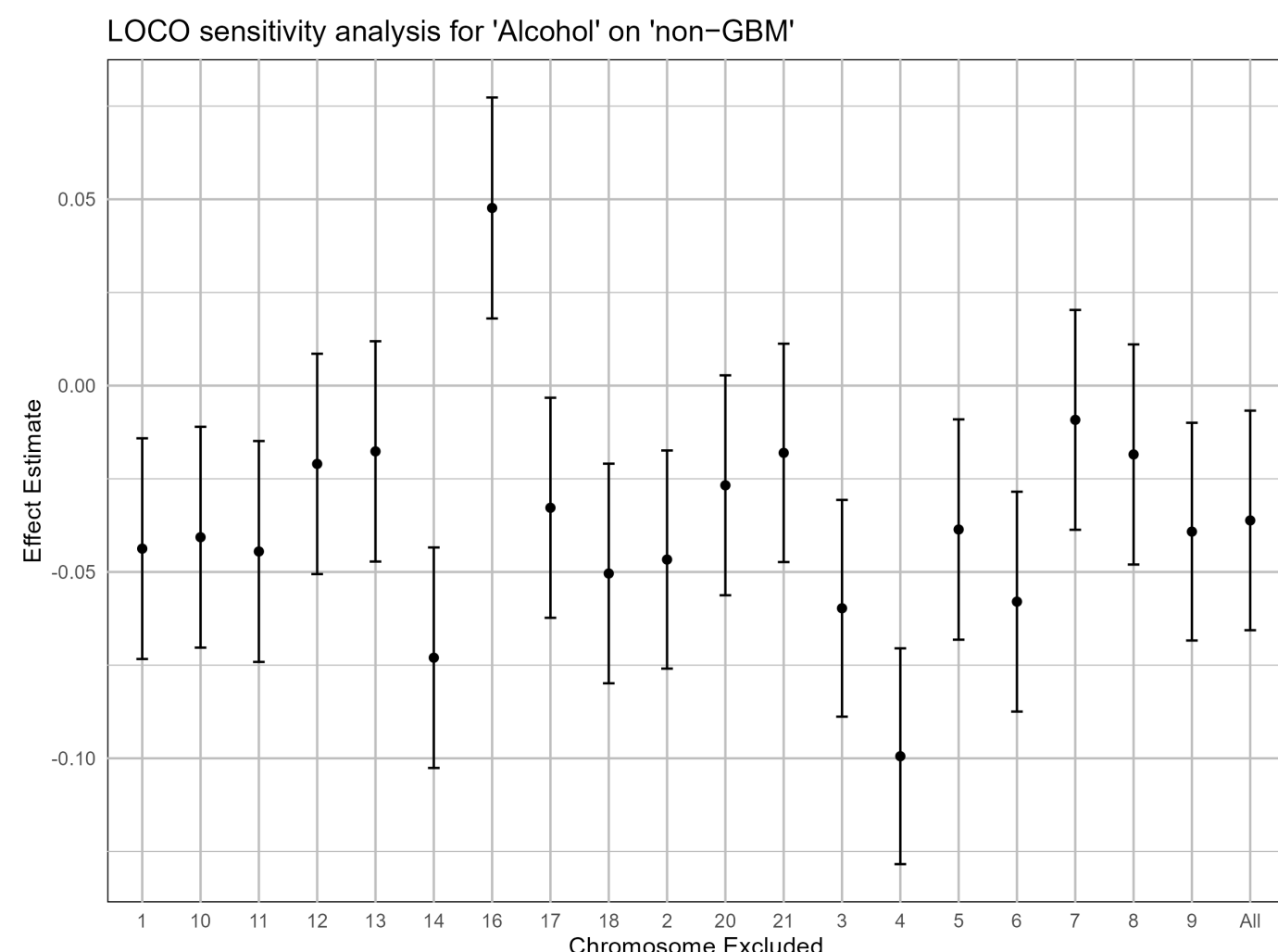

D

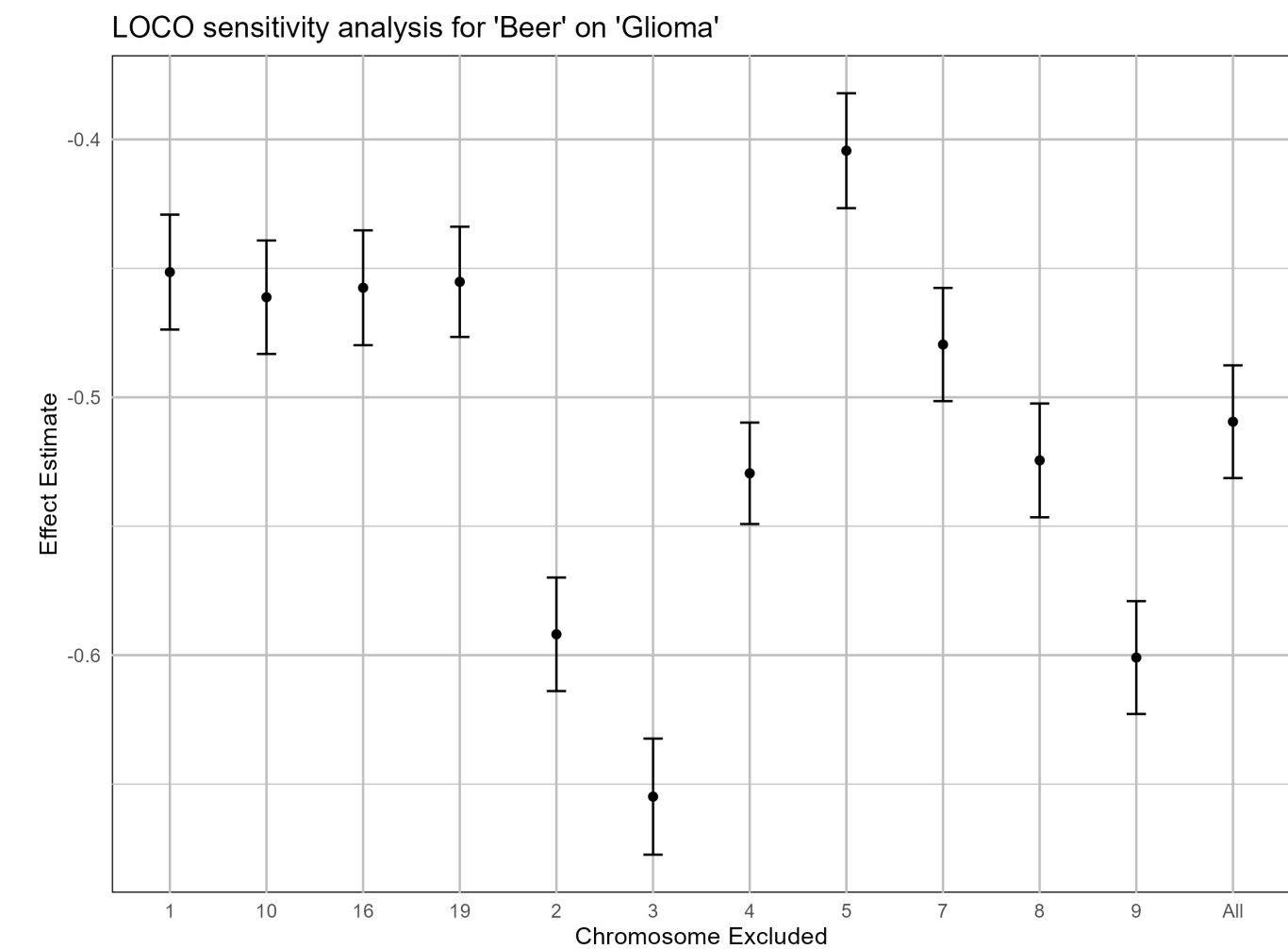

E

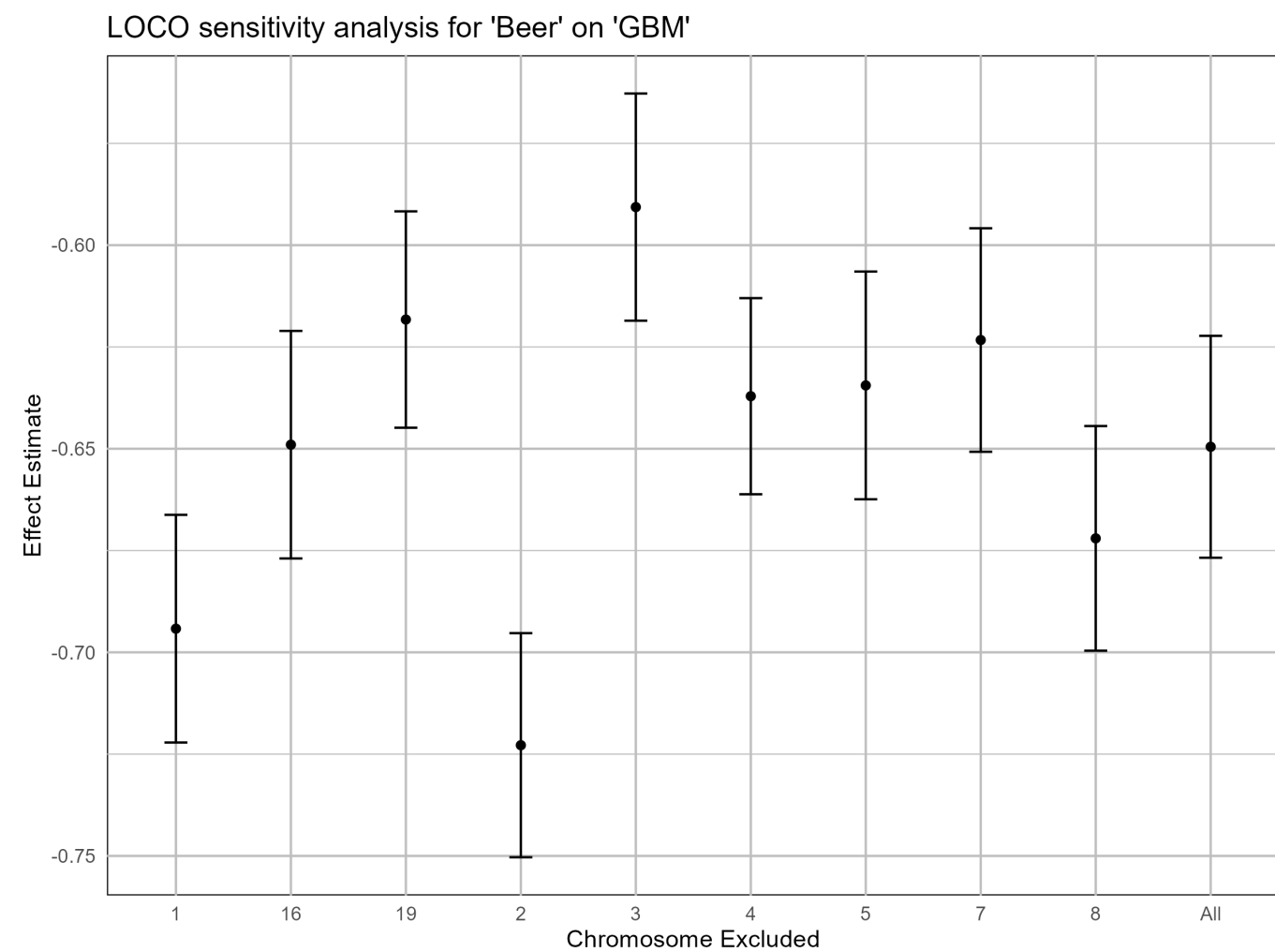

F

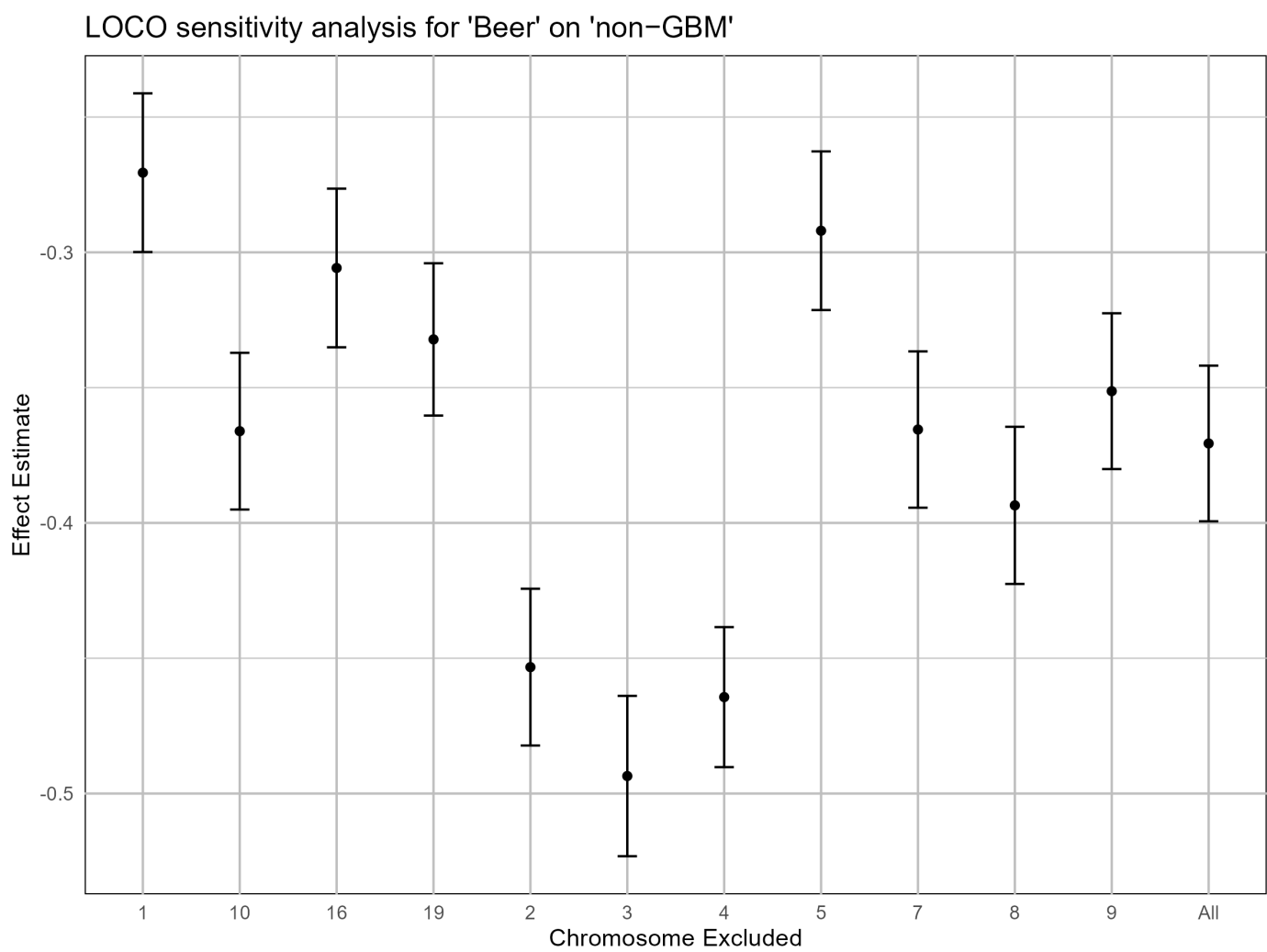

G

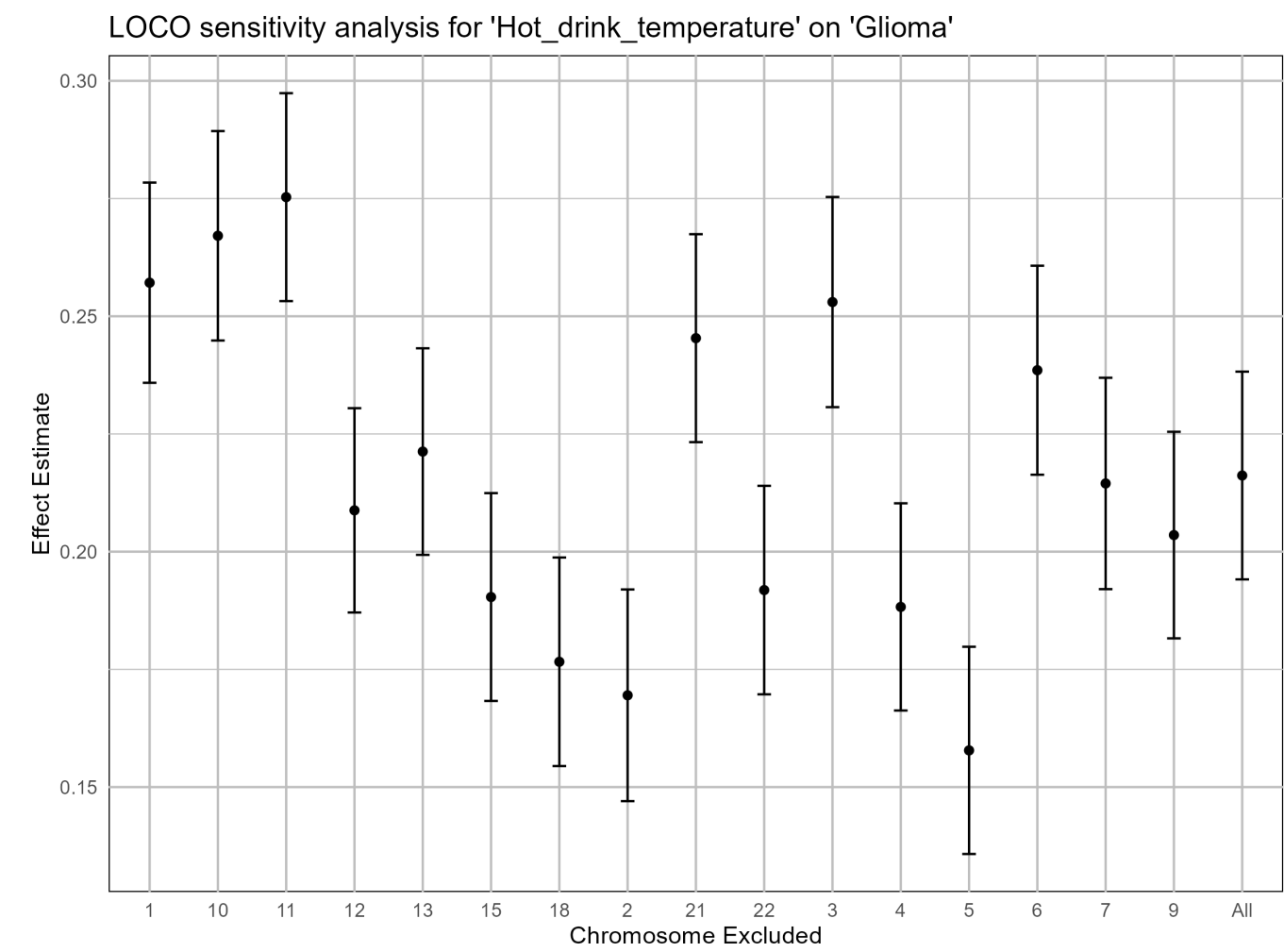

H

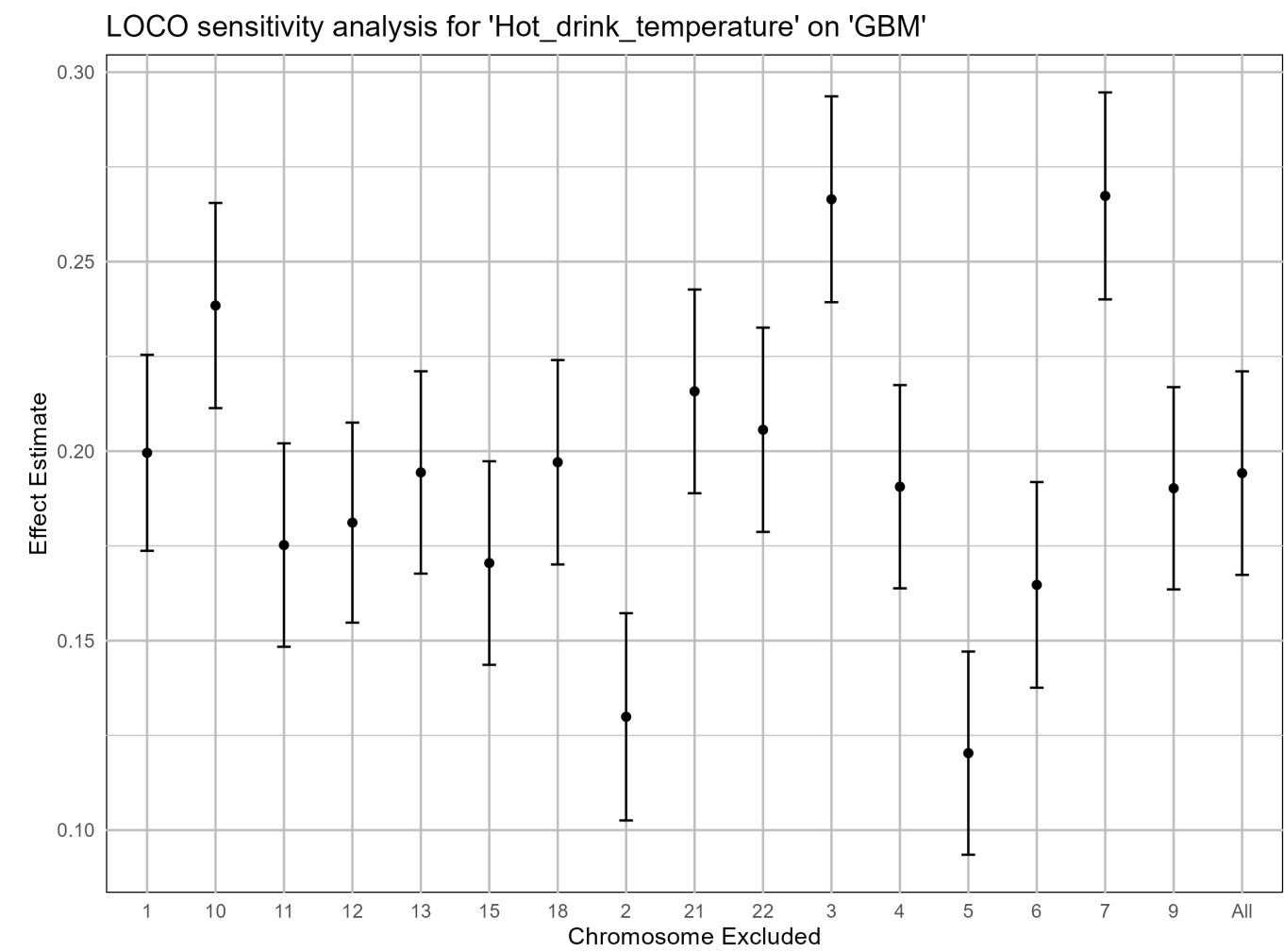

I

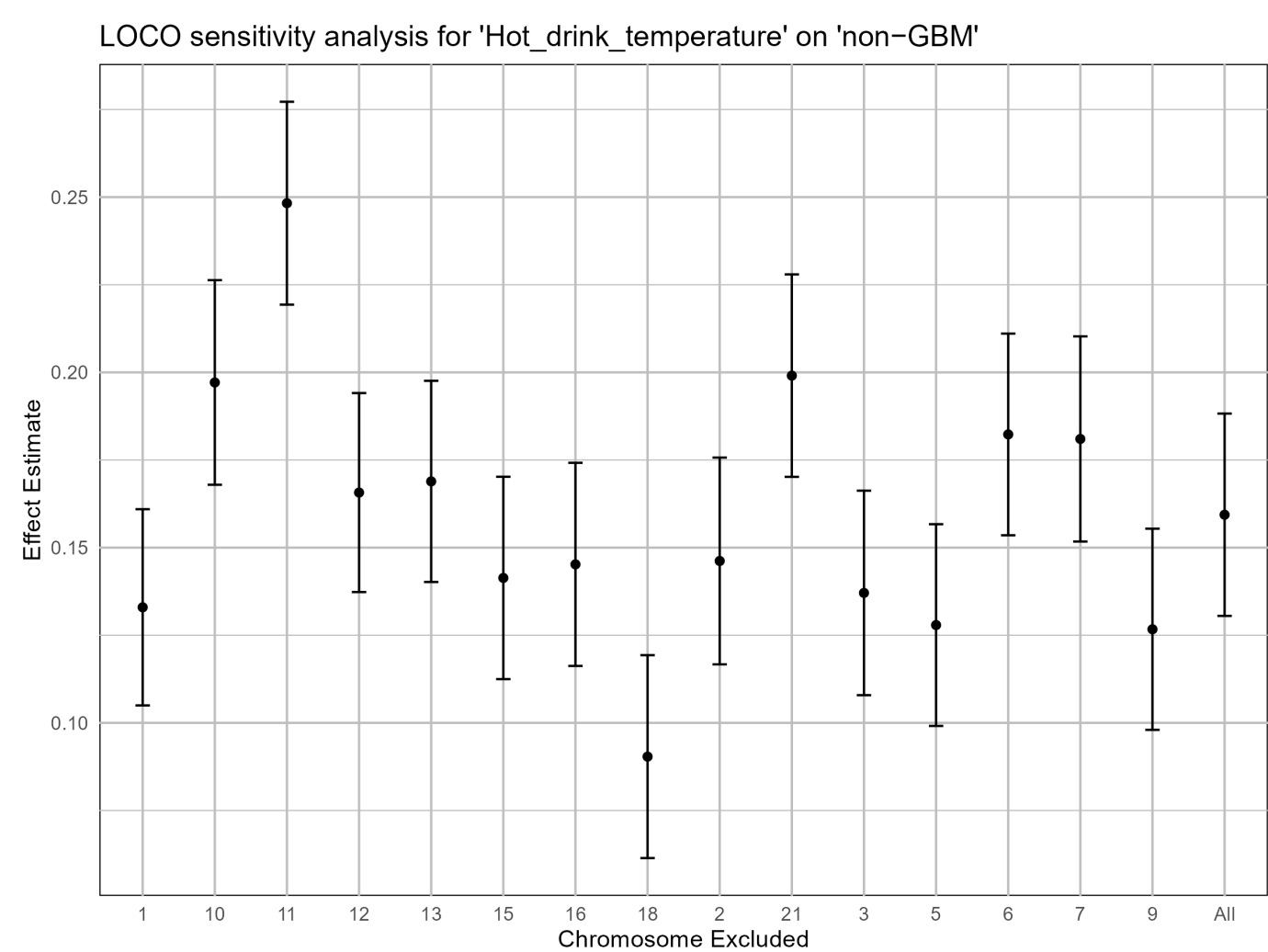

Supplement: Supplementary file 1 [file nutrients-17-00582-s001.zip › nutrients-3462880-supplementary/Sup_23.pdf]

A

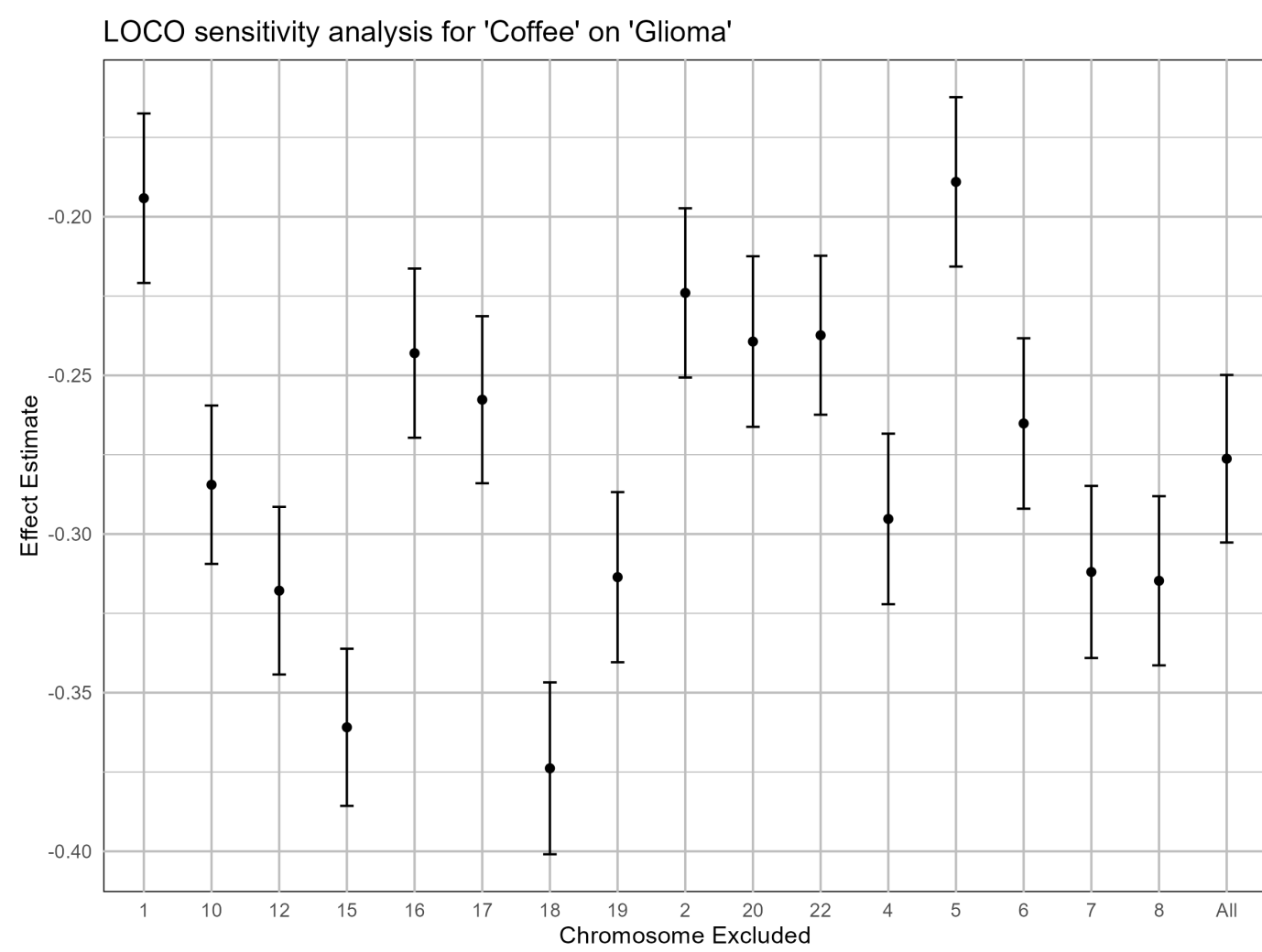

B

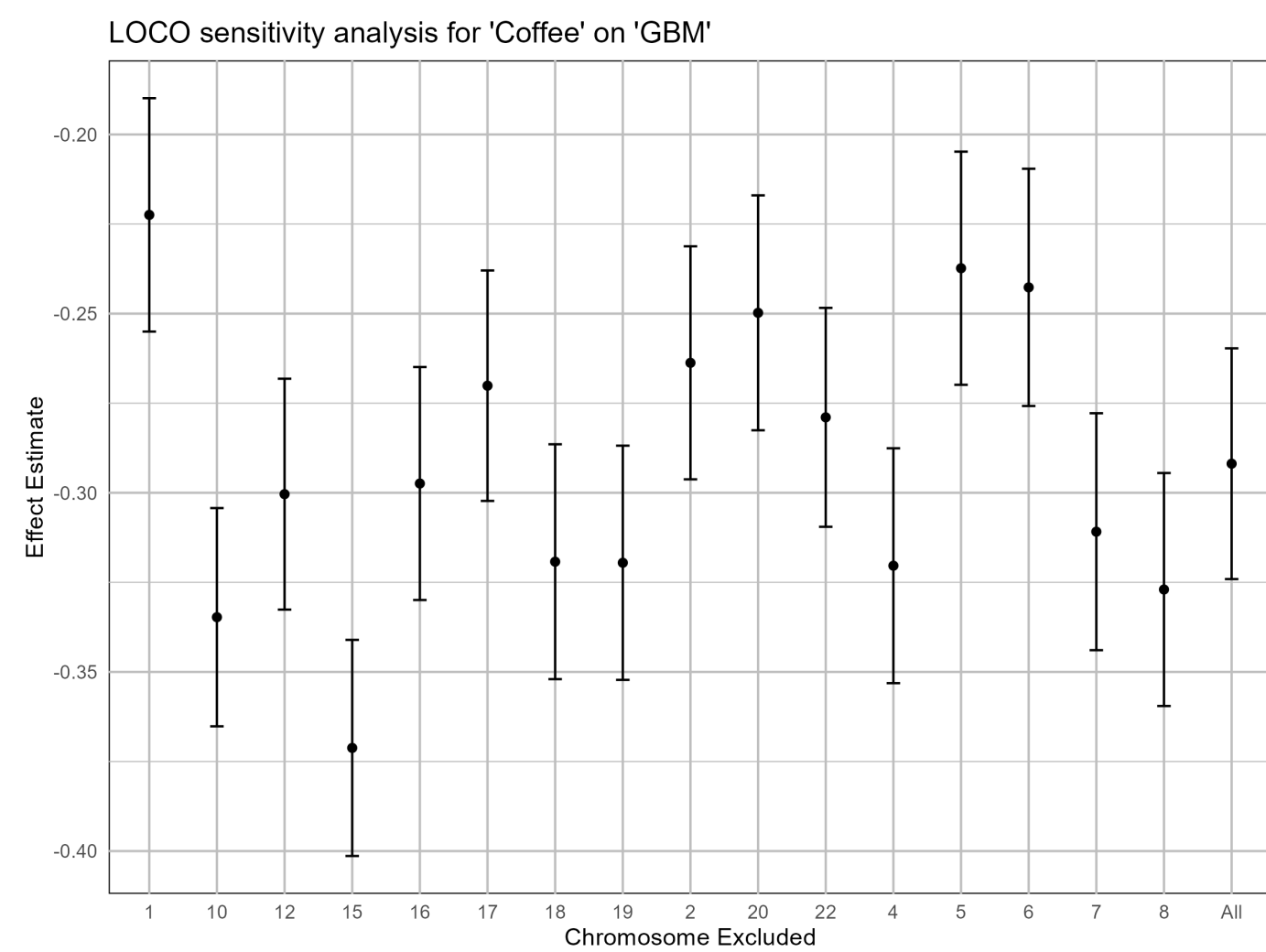

C

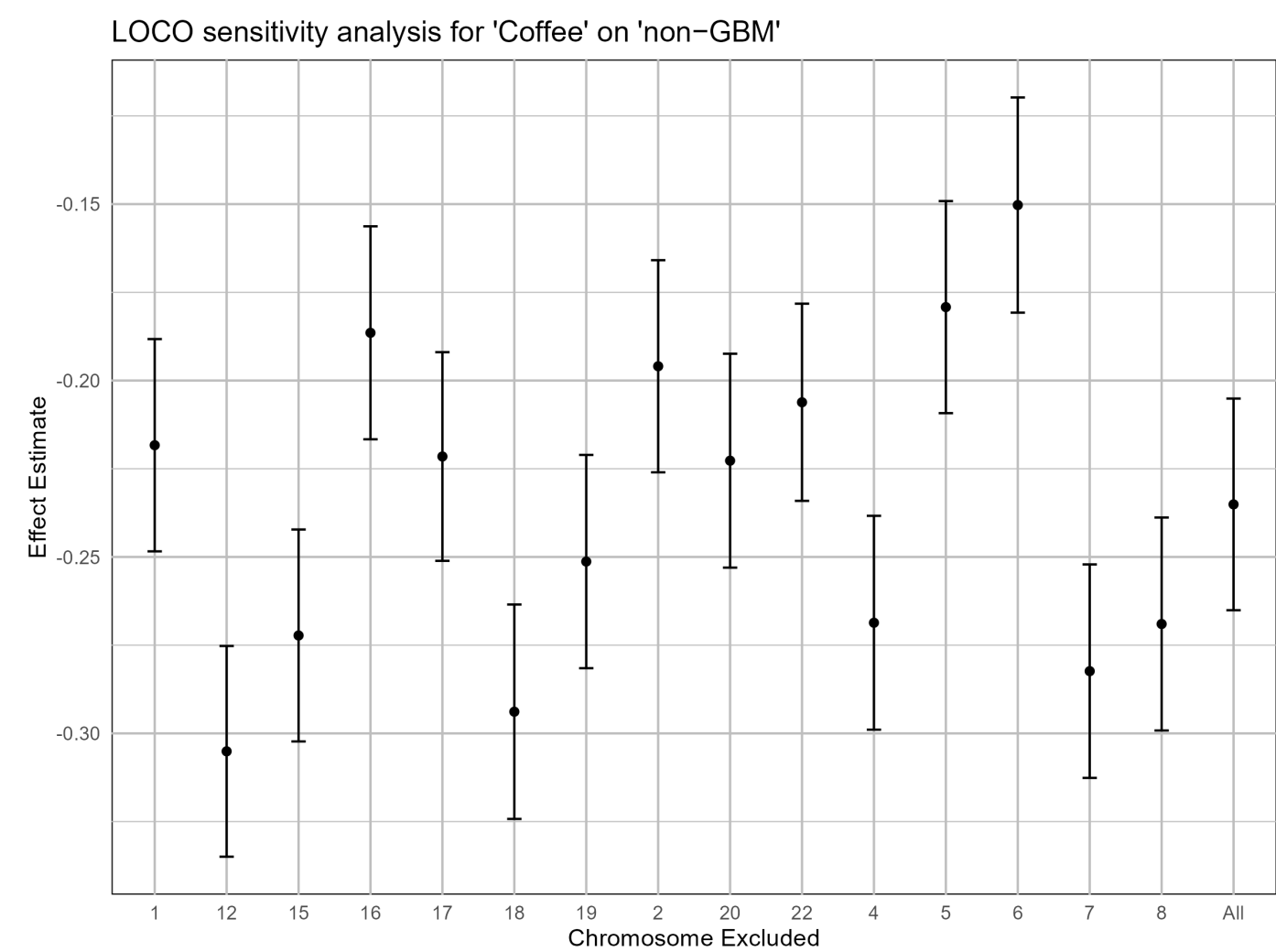

D

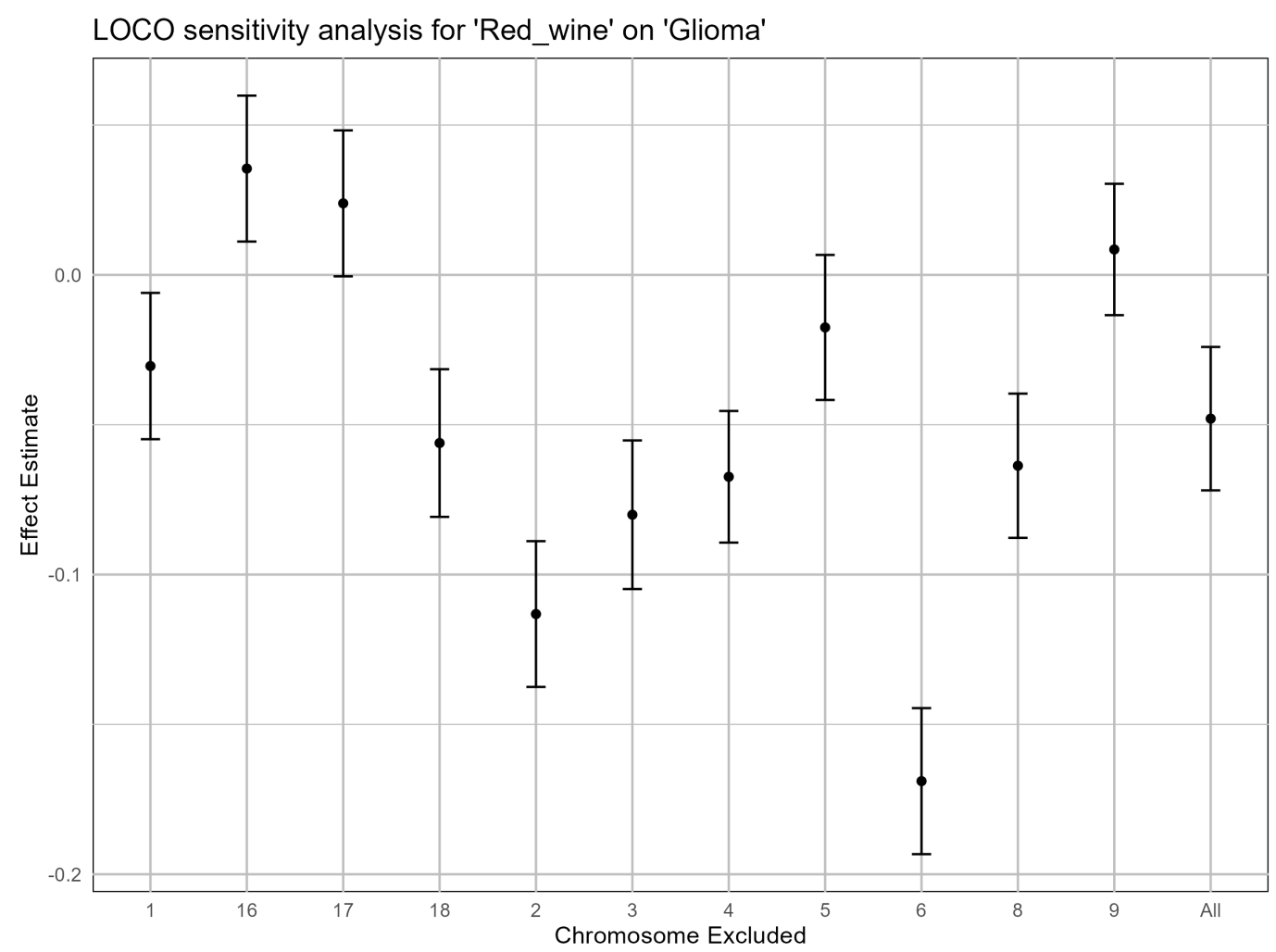

E

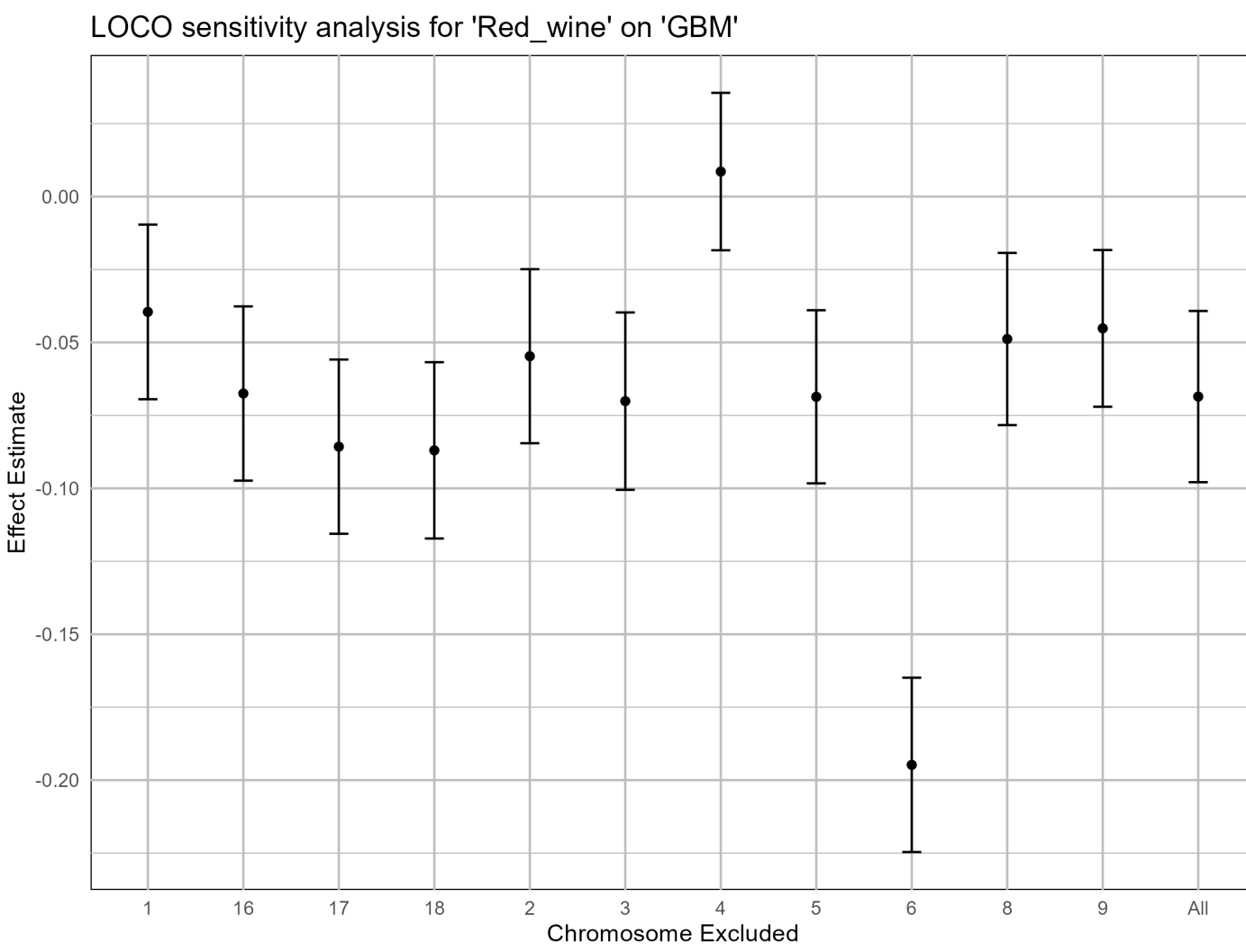

F

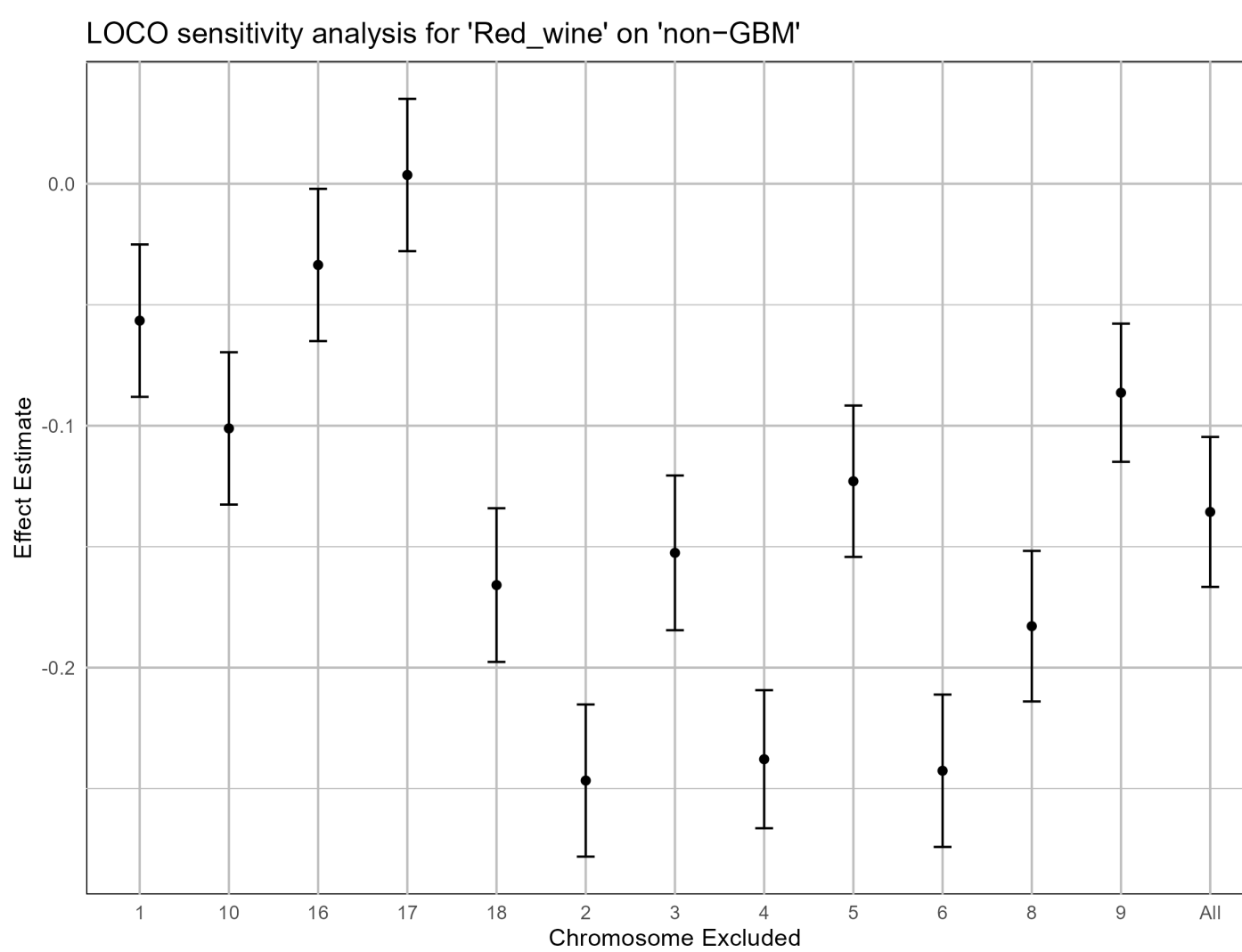

G

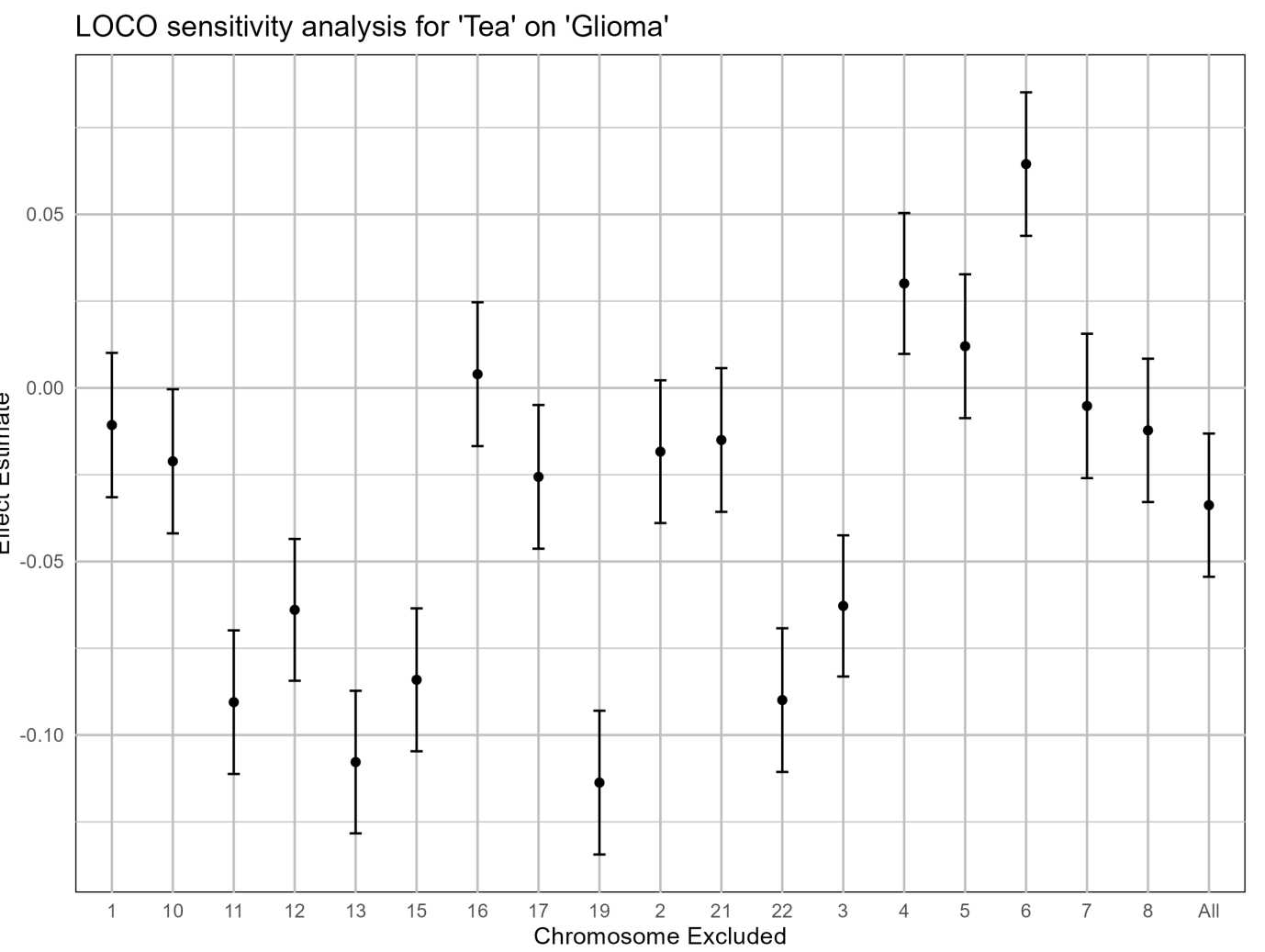

H

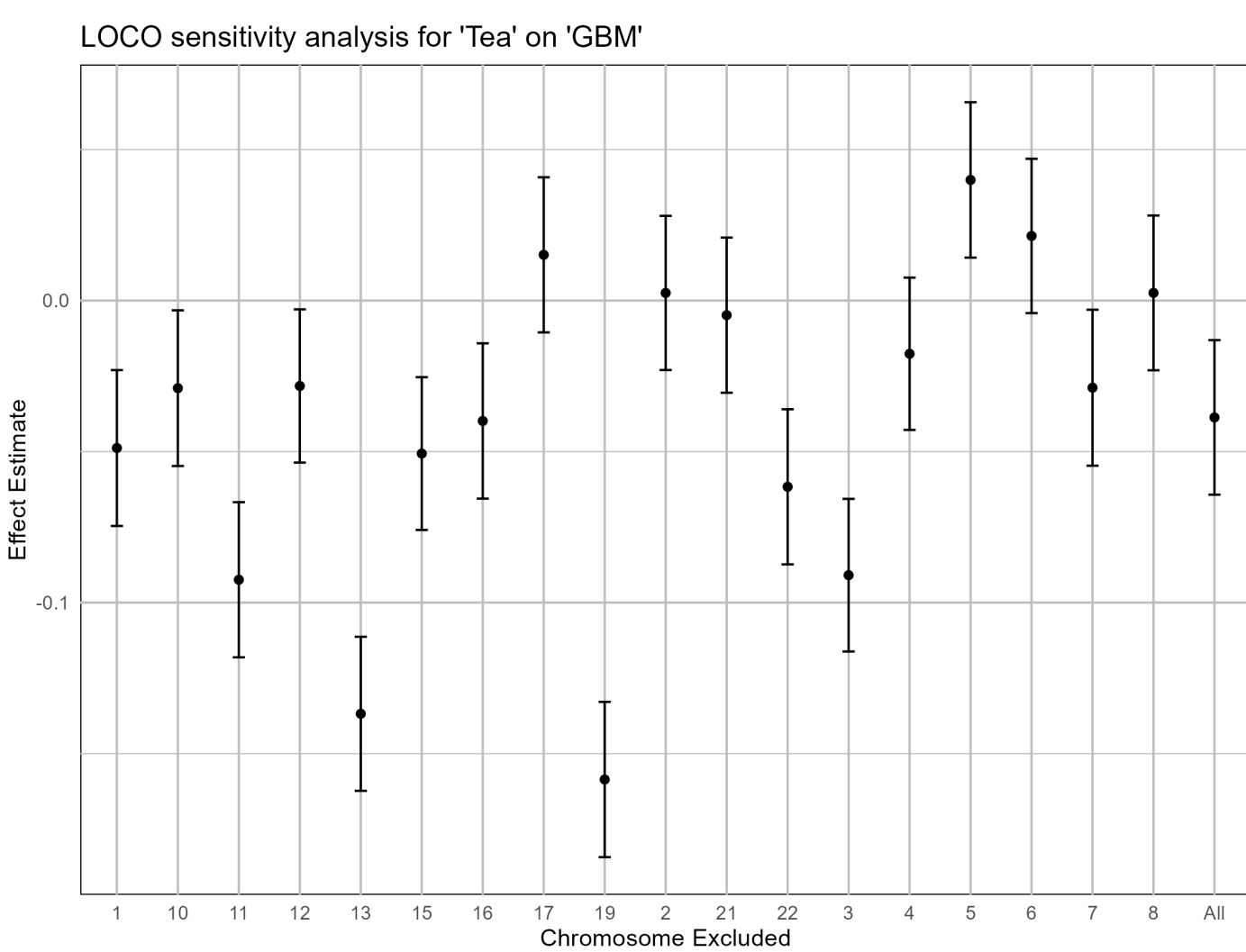

I

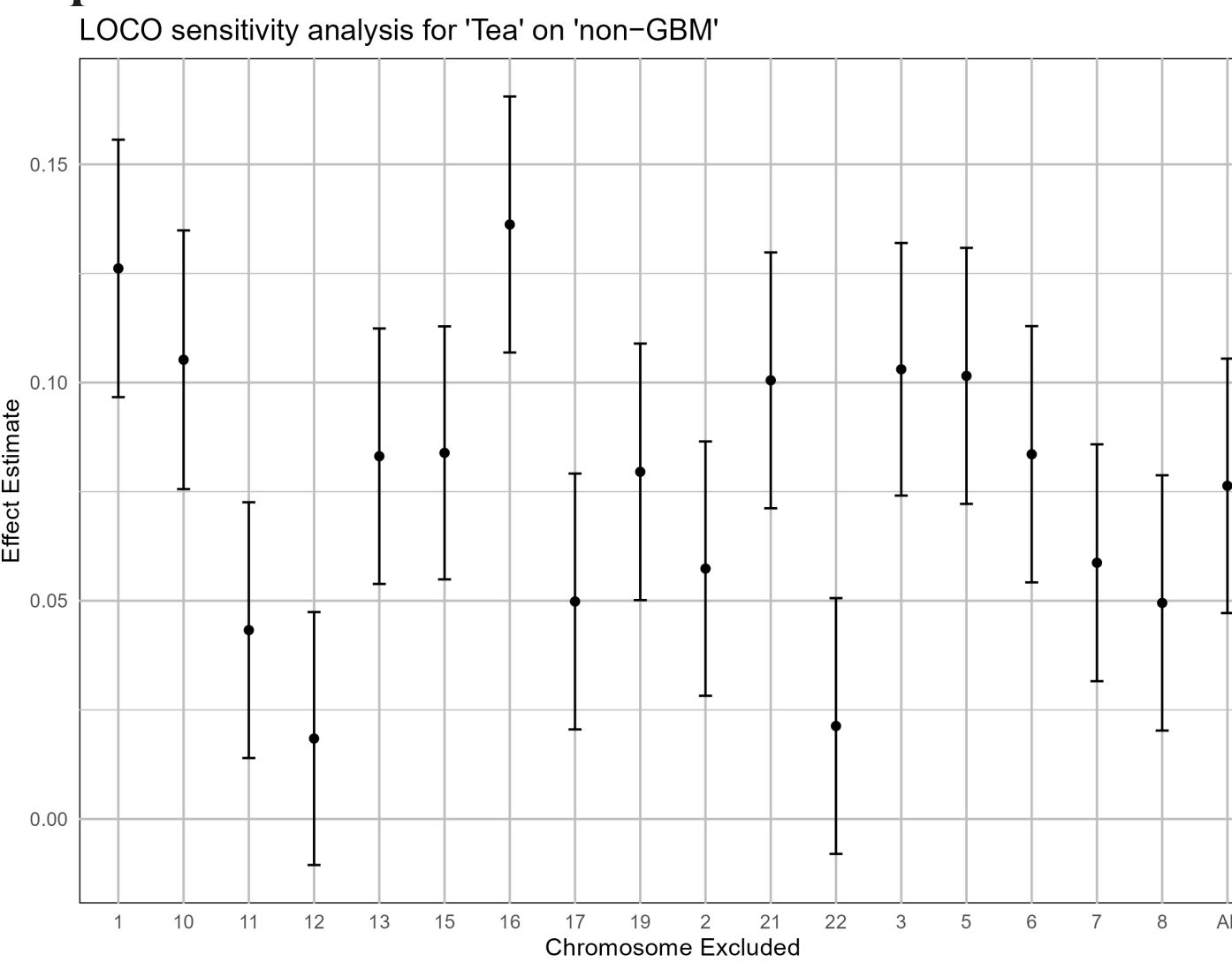

Supplement: Supplementary file 1 [file nutrients-17-00582-s001.zip › nutrients-3462880-supplementary/Sup_24.pdf]

A

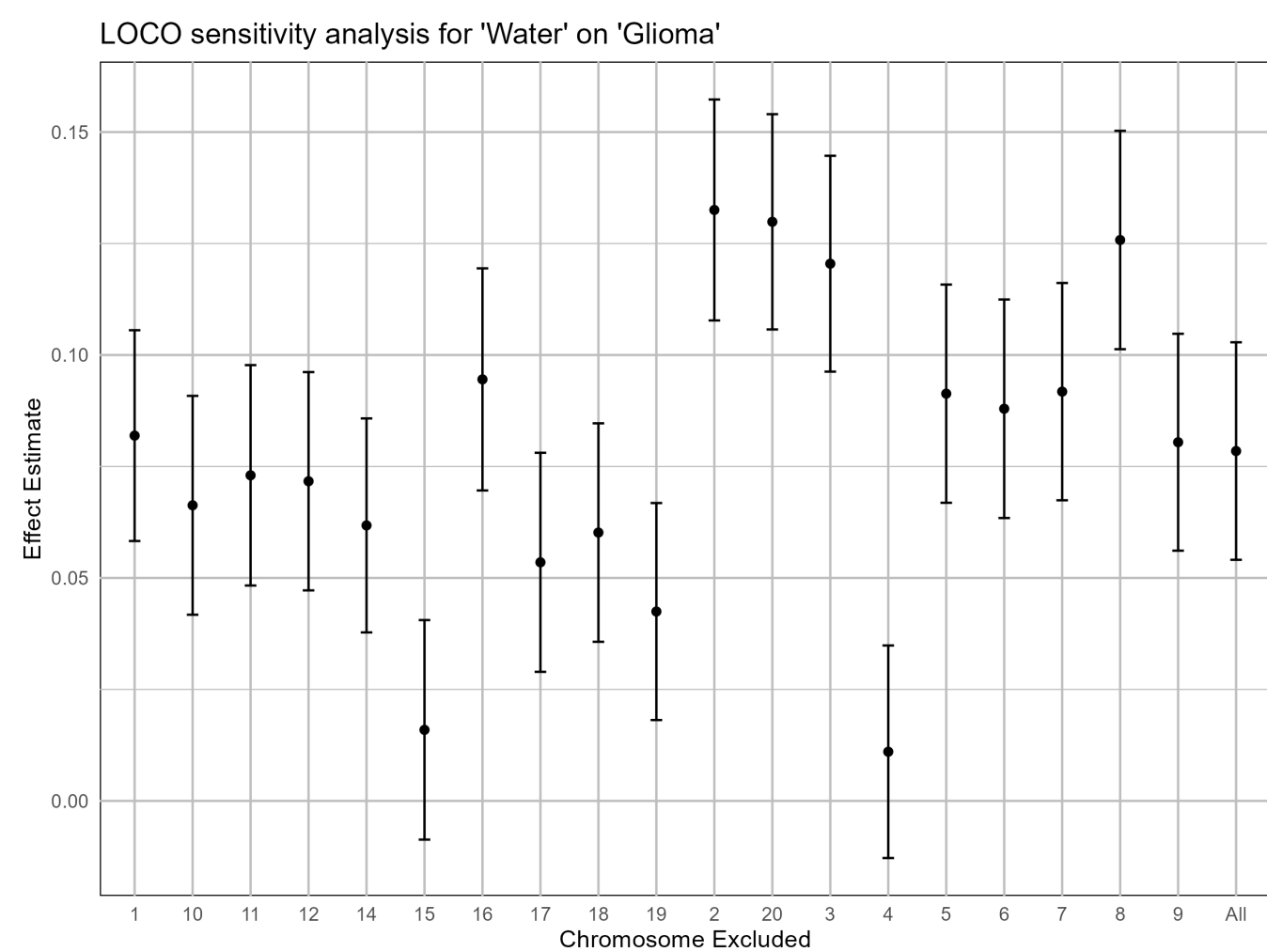

B

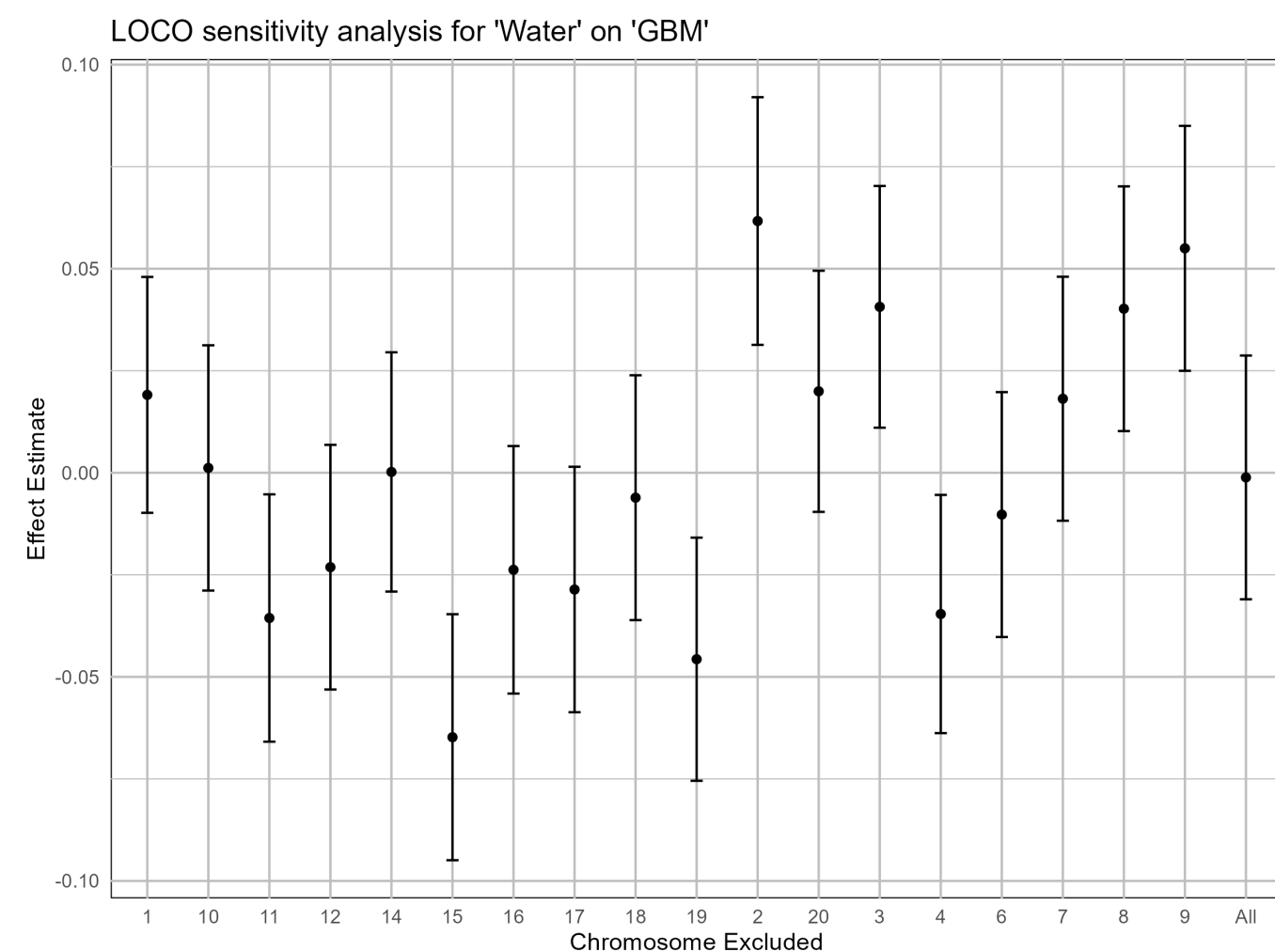

C

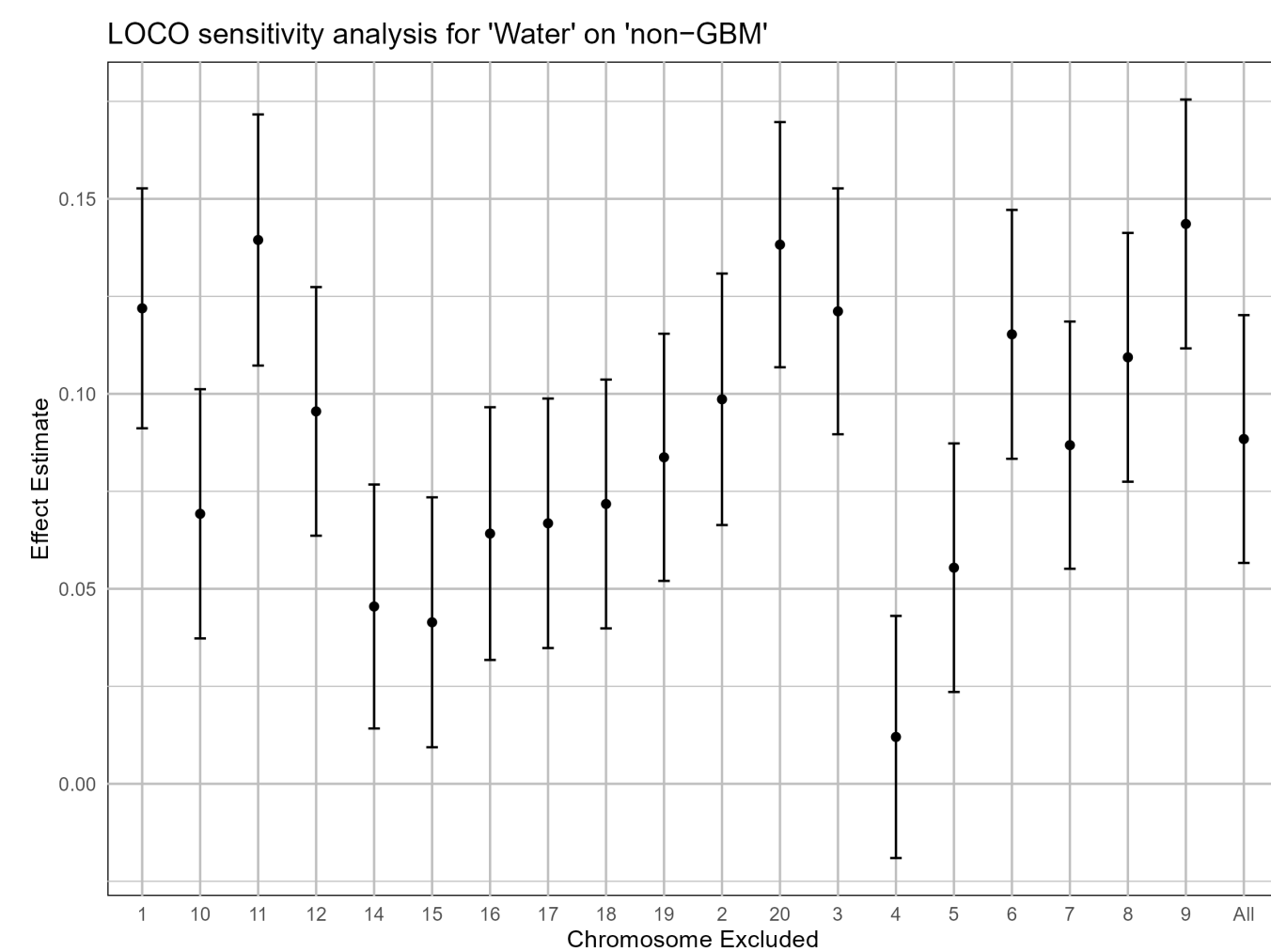

D

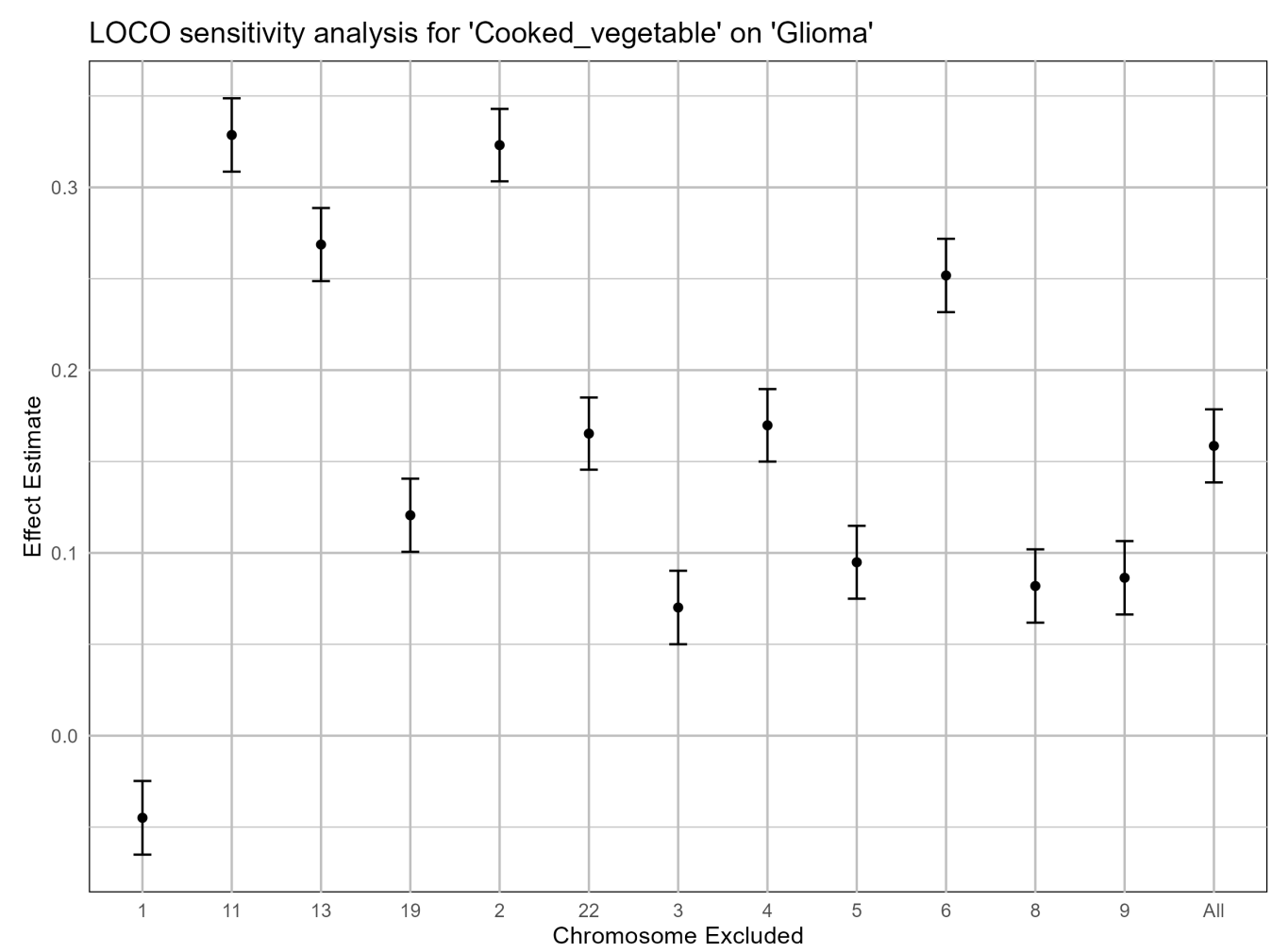

E

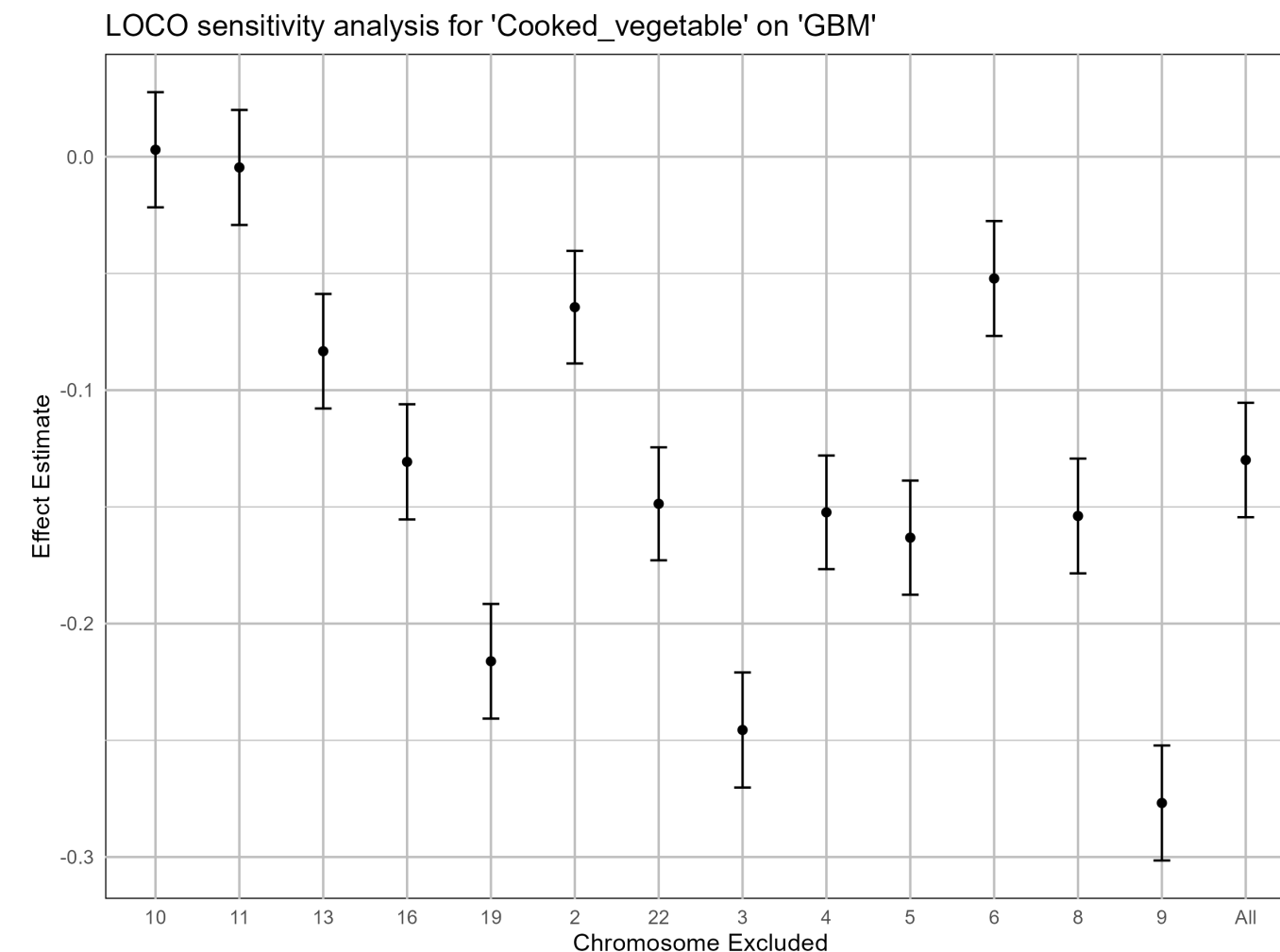

F

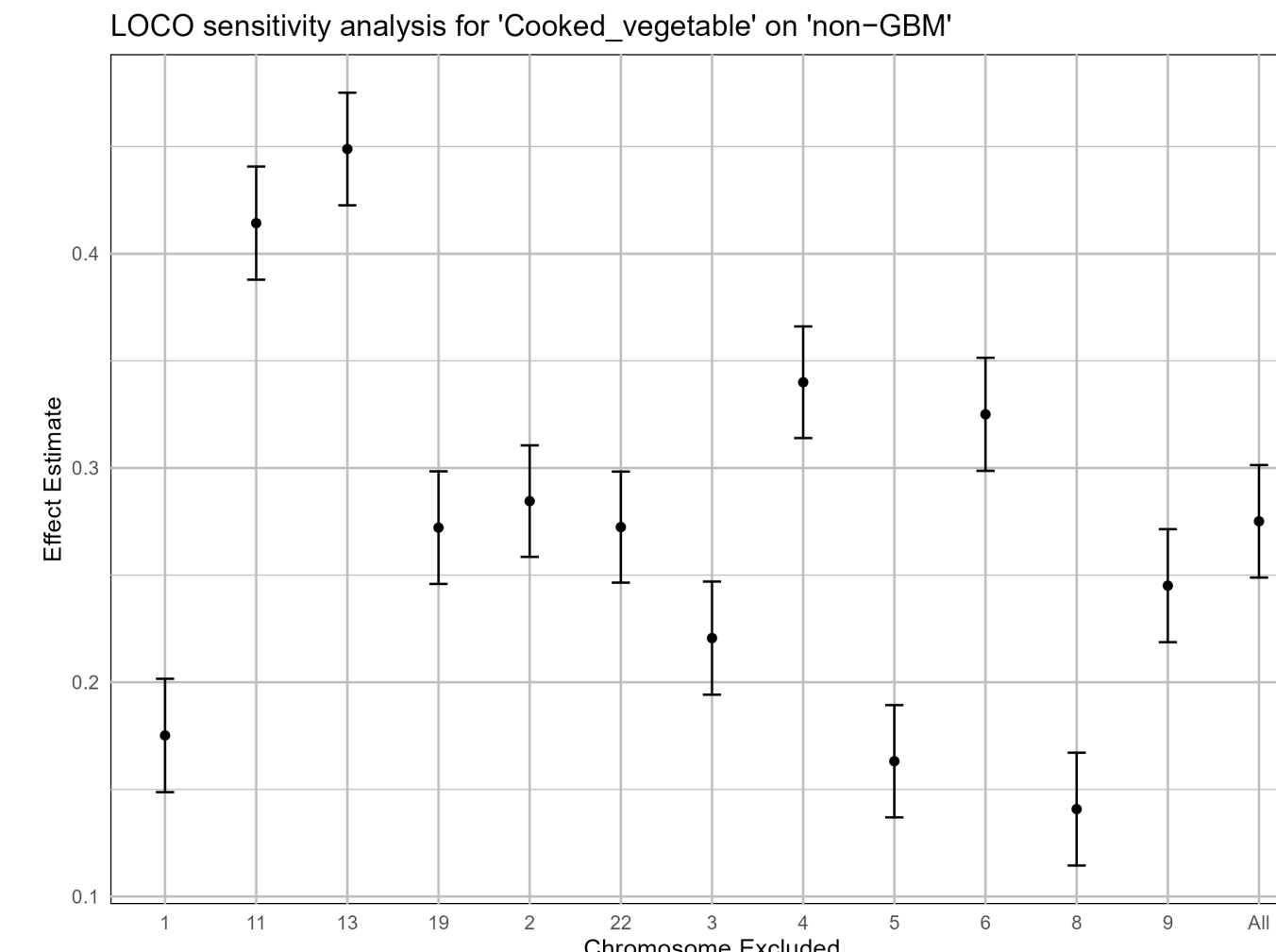

G

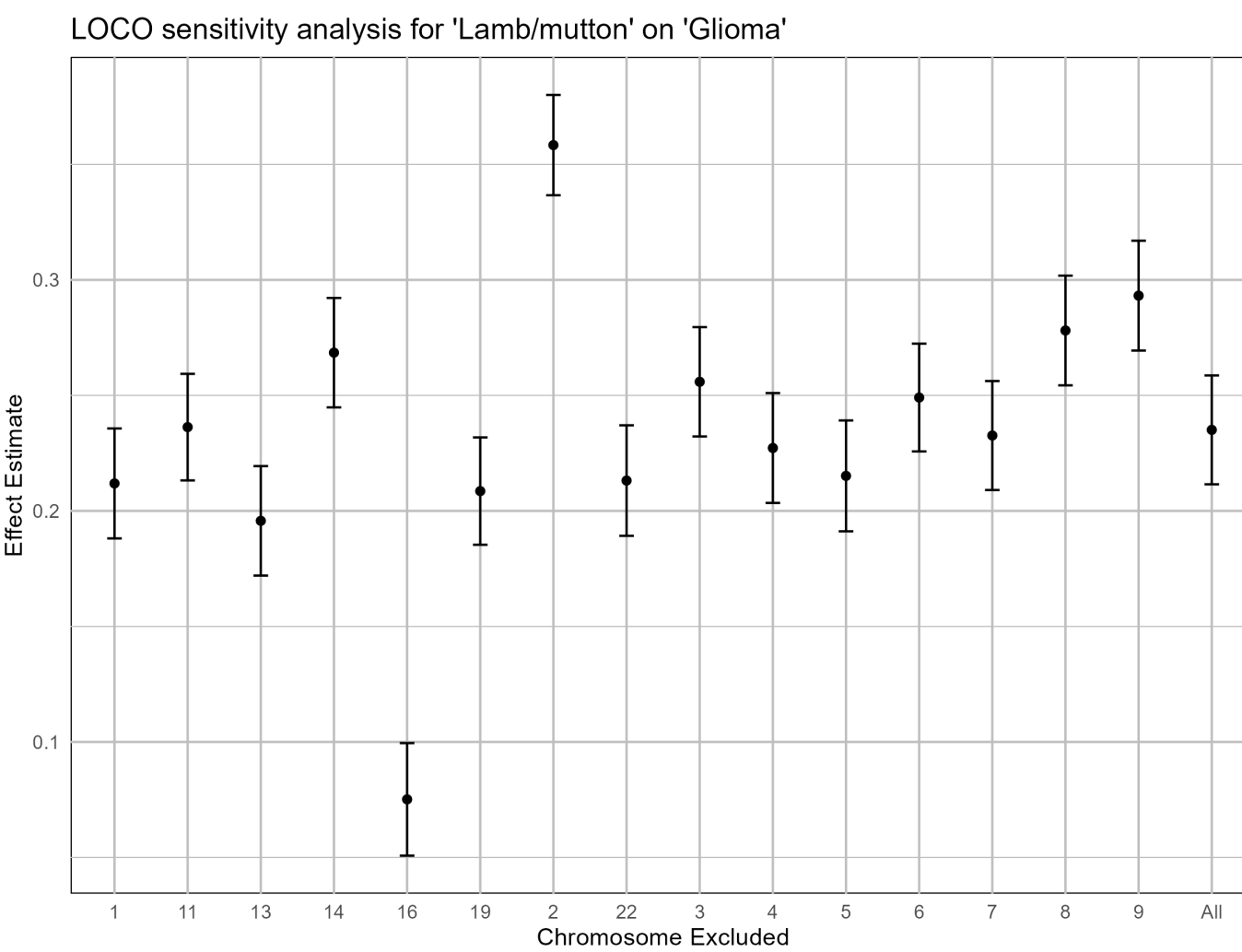

H

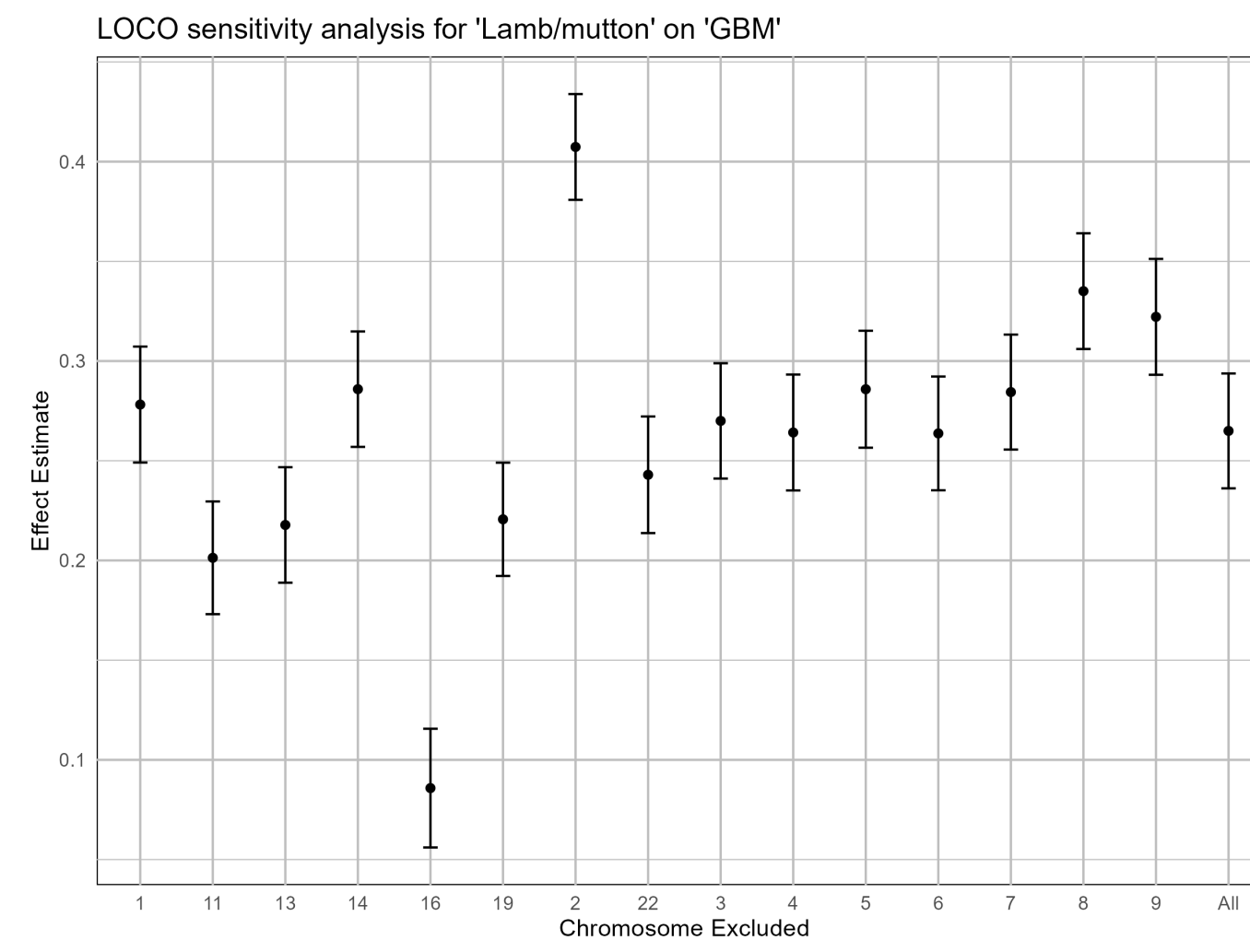

I

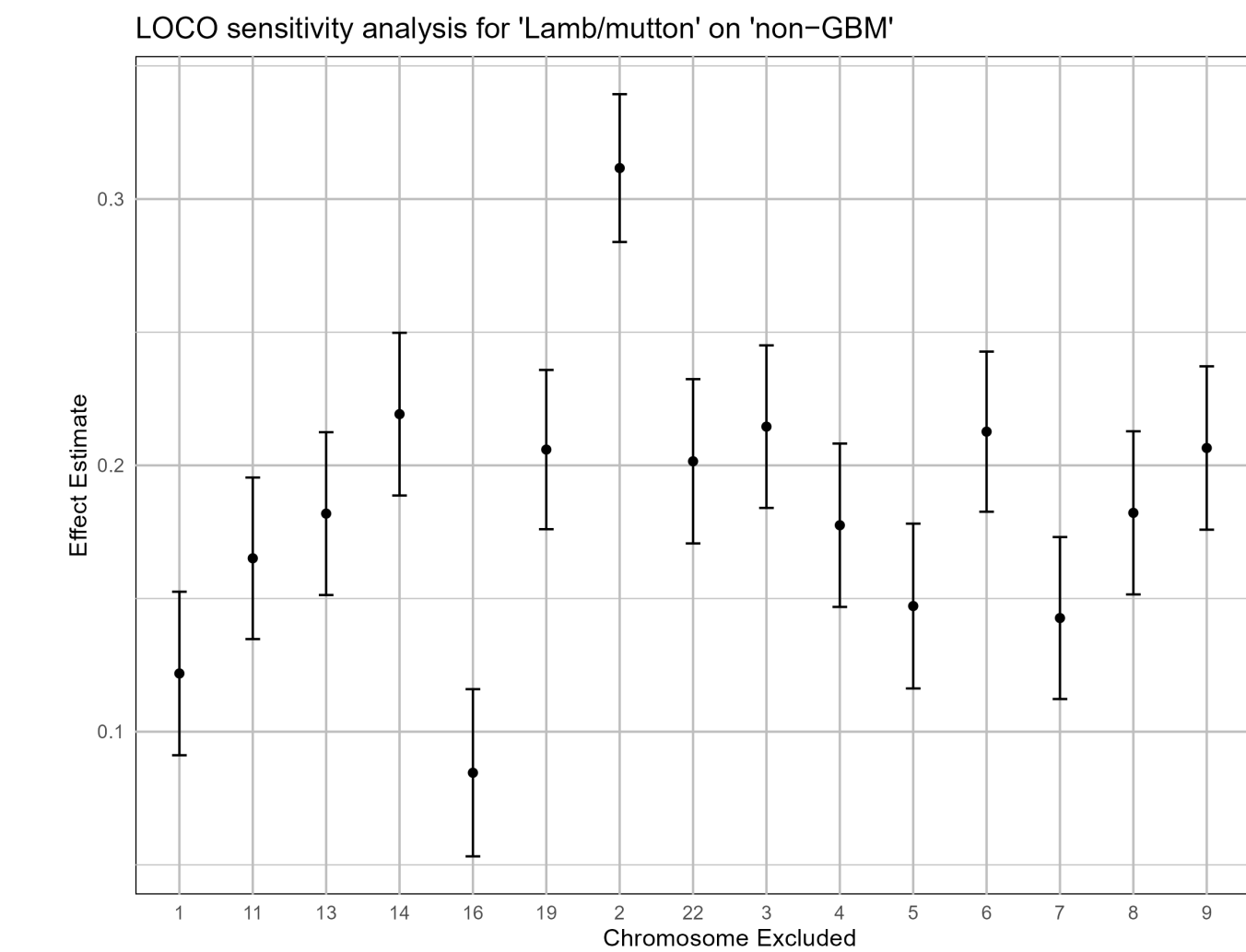

Supplement: Supplementary file 1 [file nutrients-17-00582-s001.zip › nutrients-3462880-supplementary/Sup_25.pdf]

A

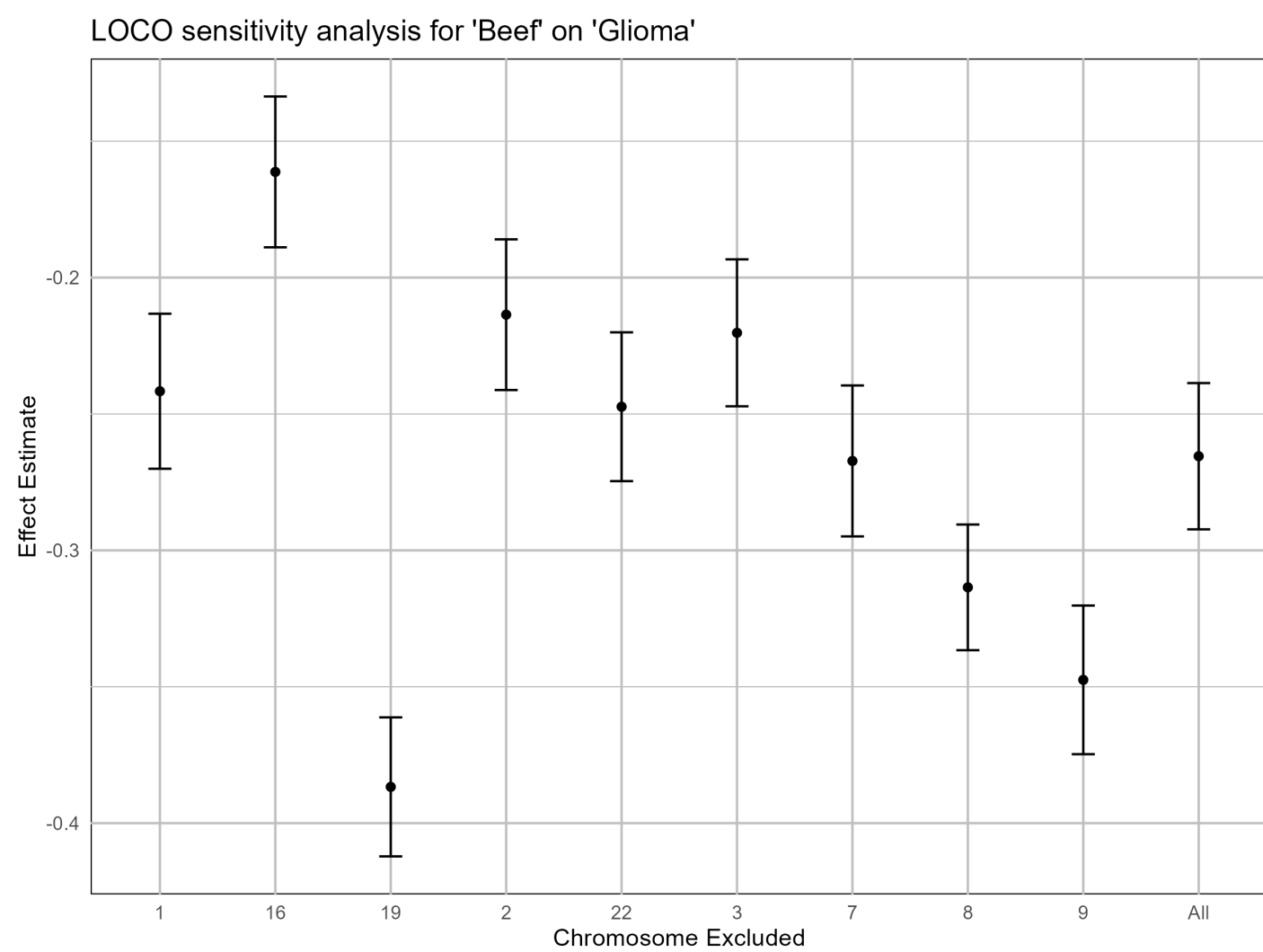

B

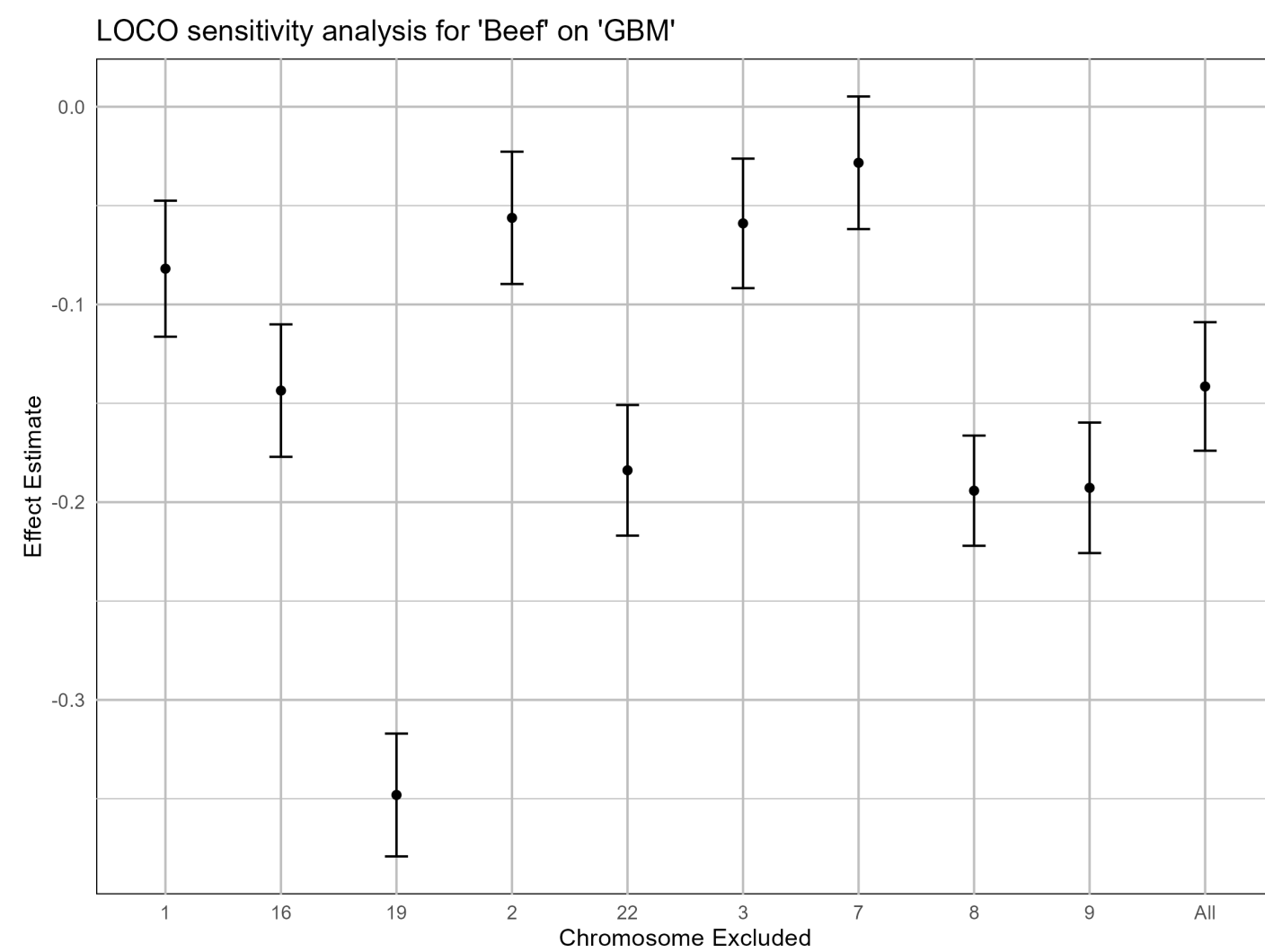

C

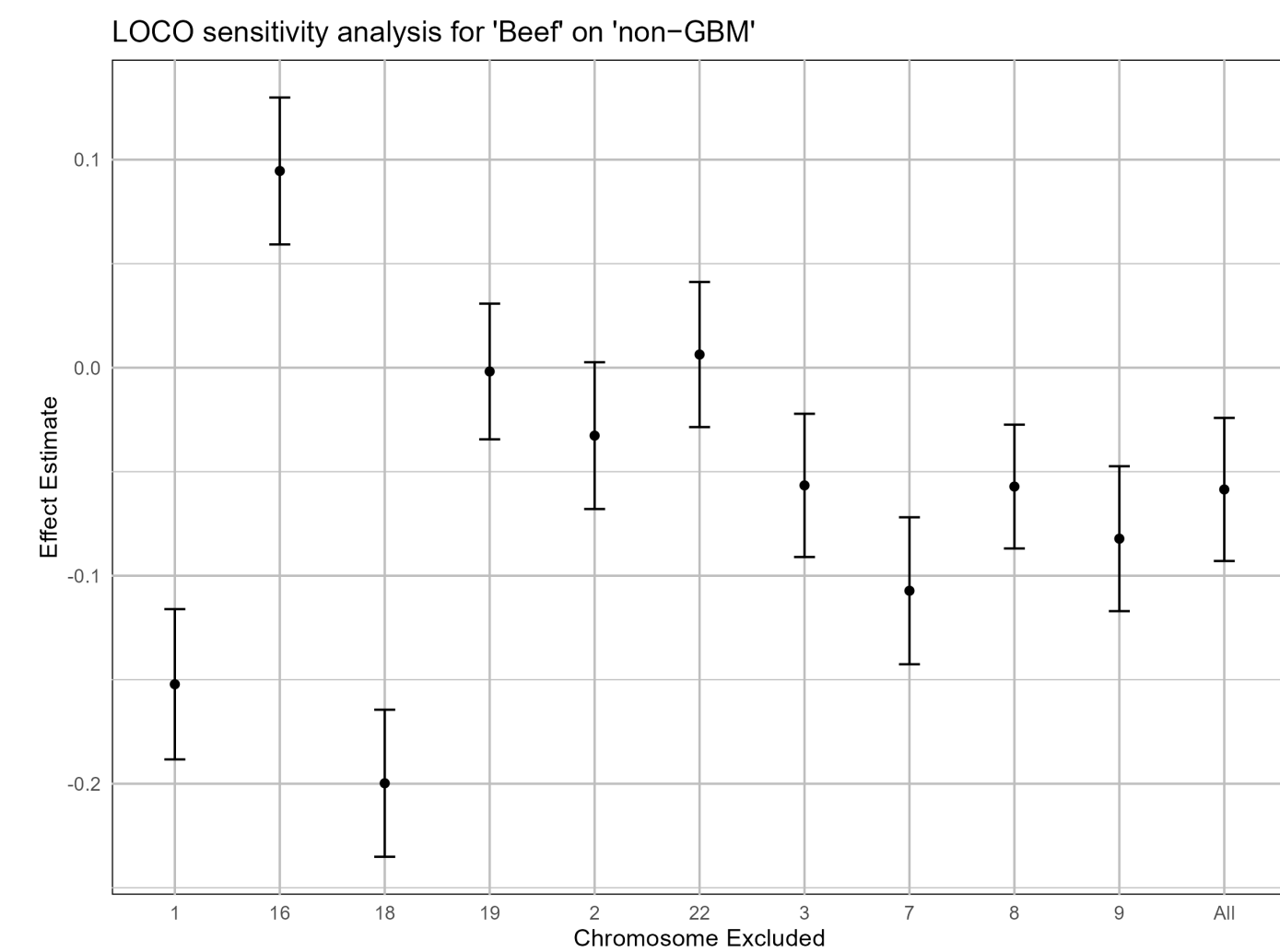

D

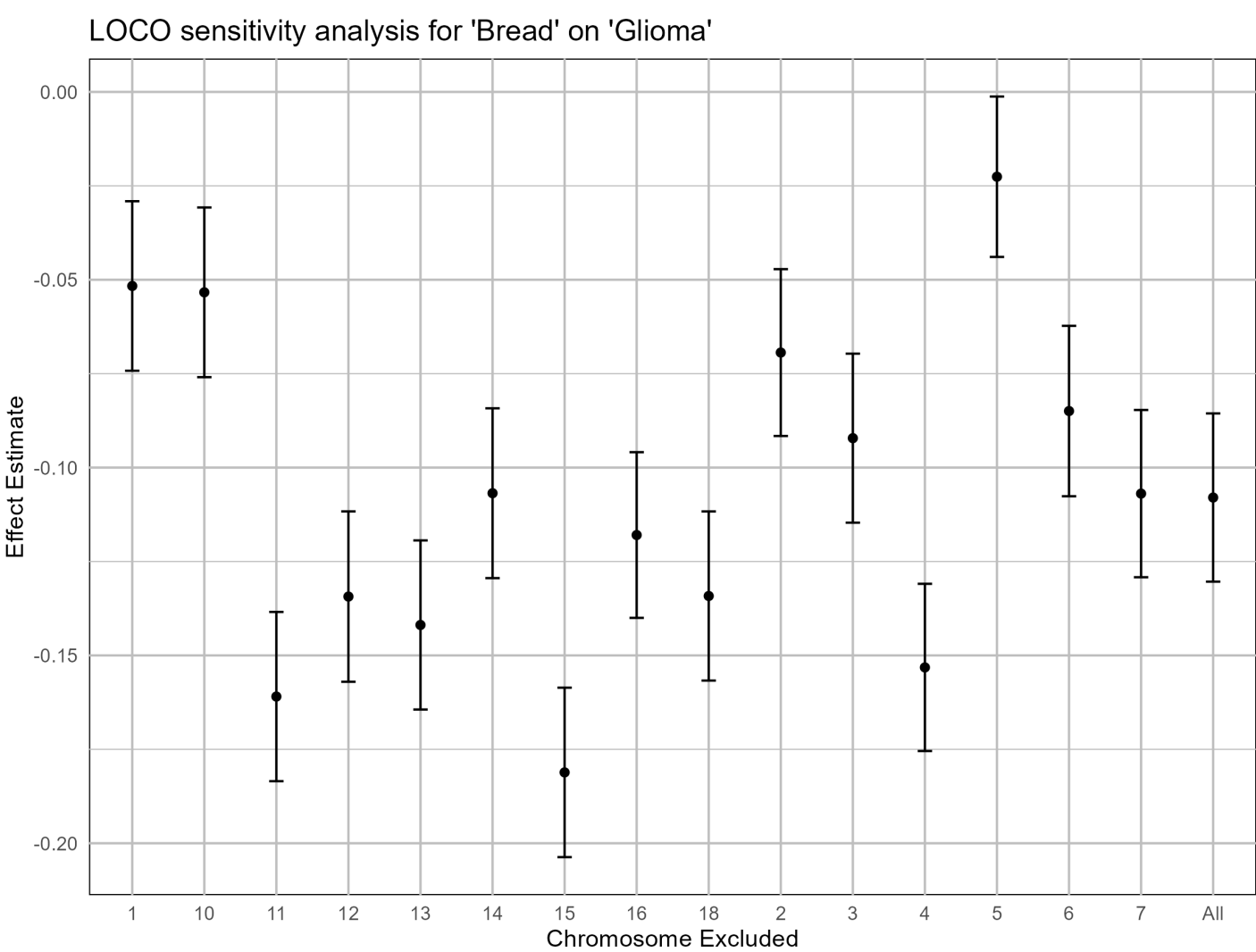

E

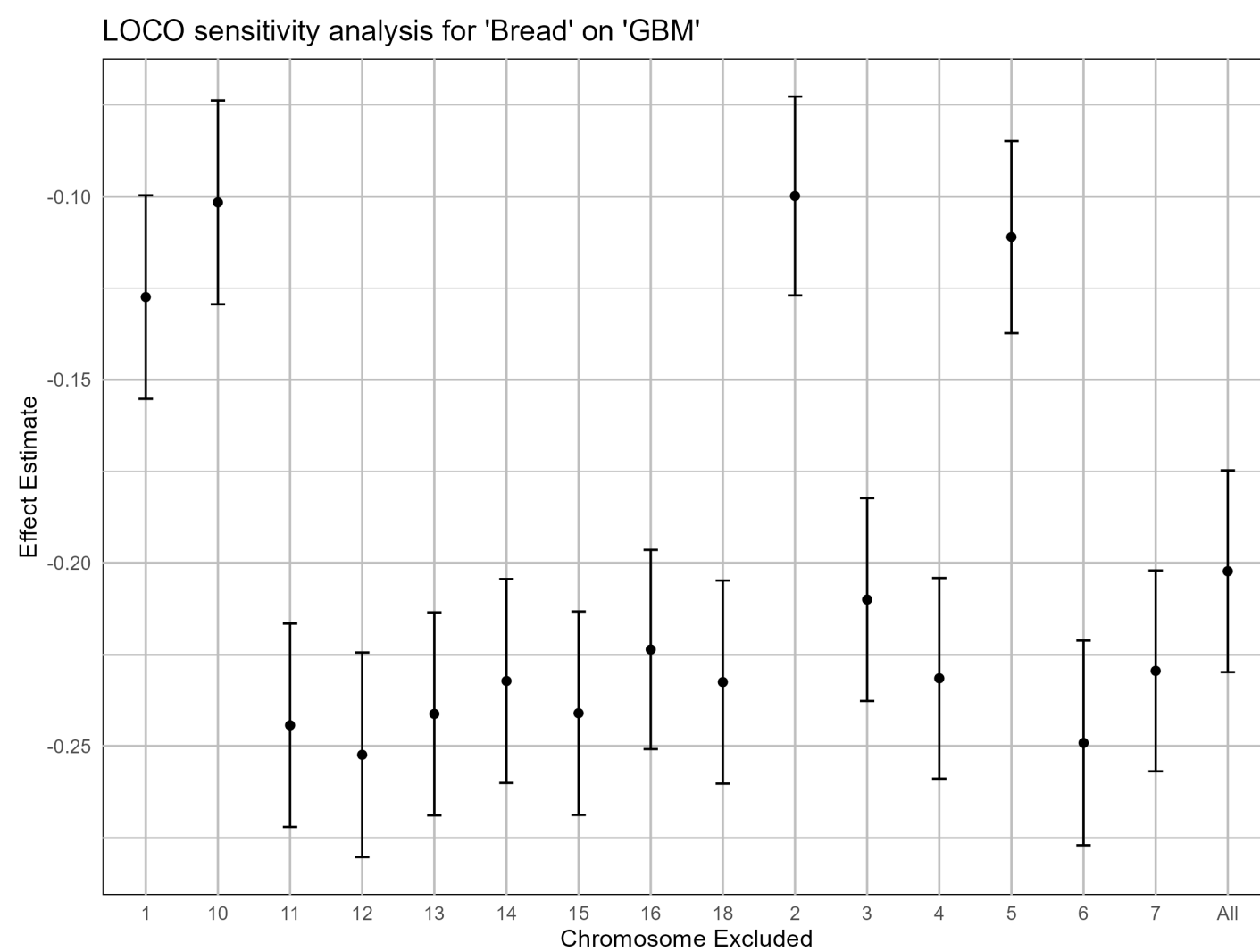

F

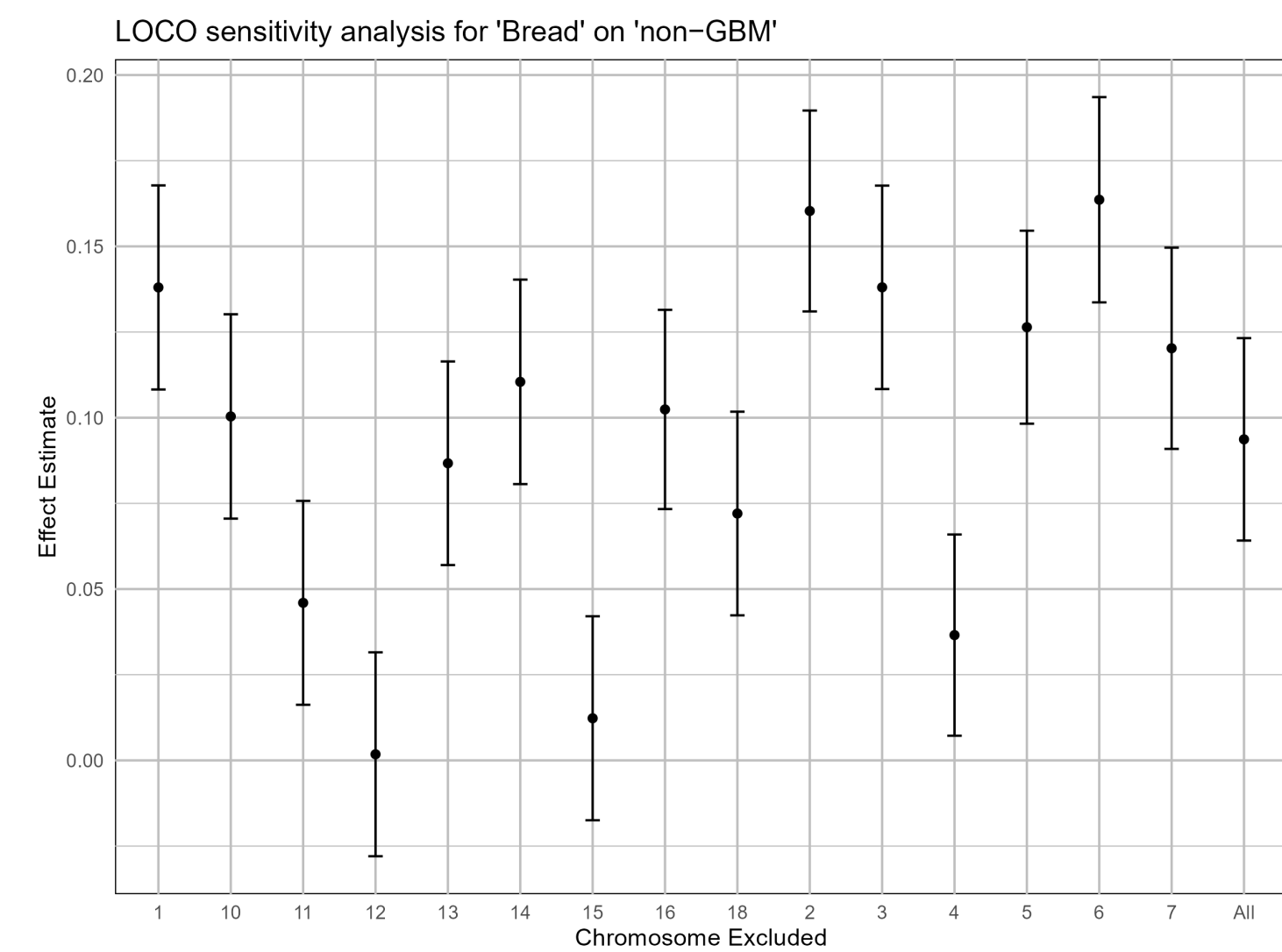

G

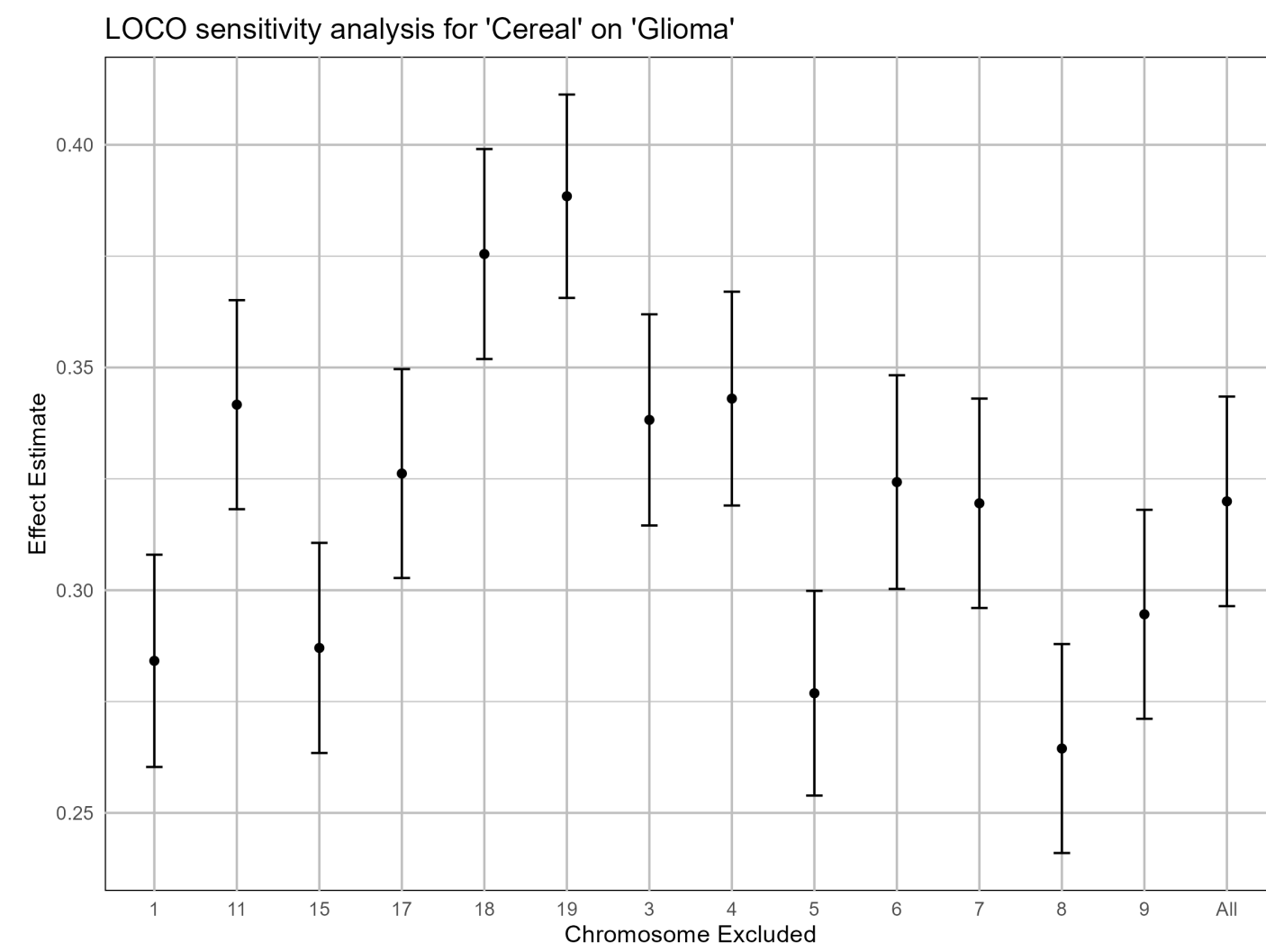

H

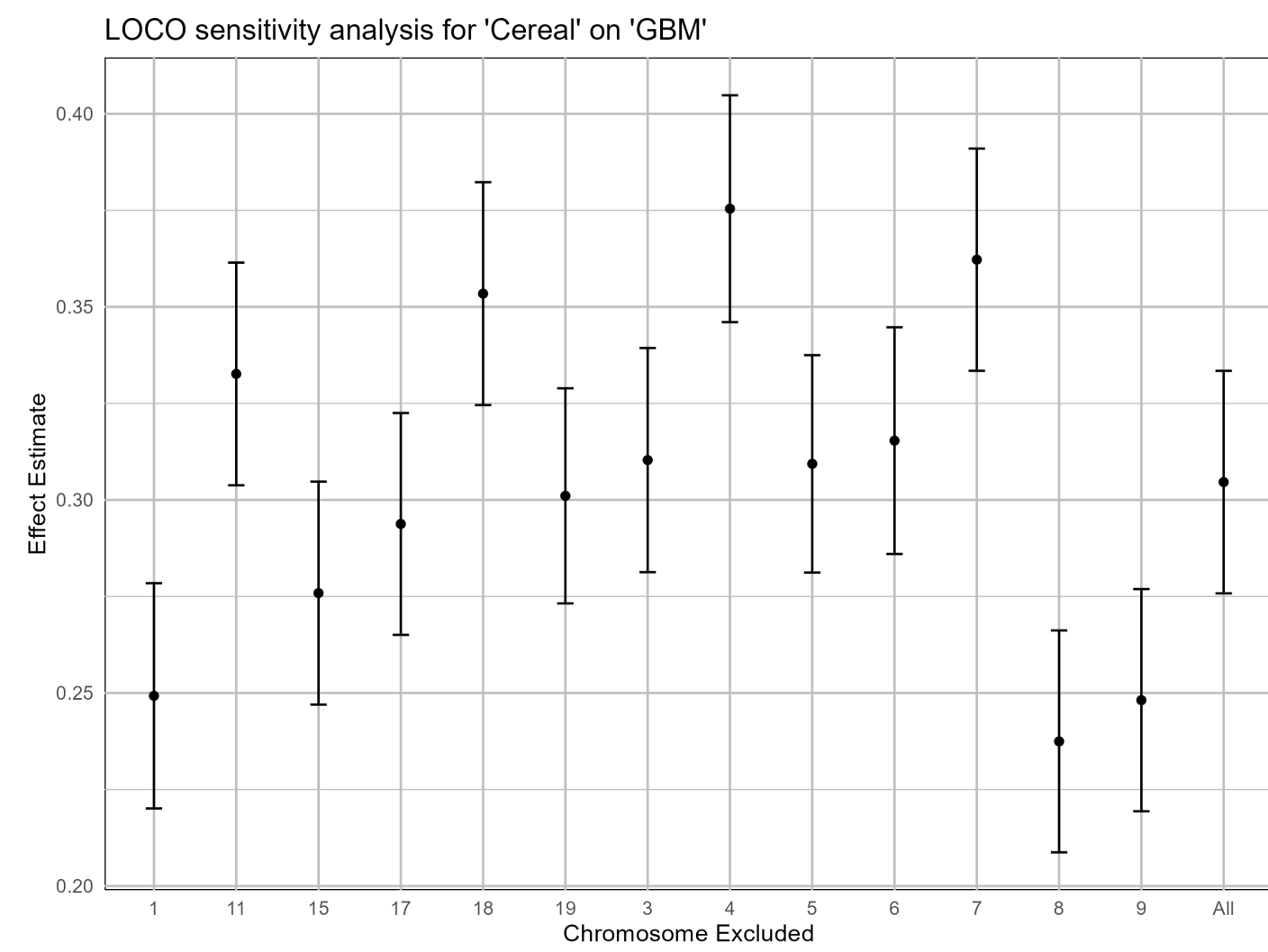

I

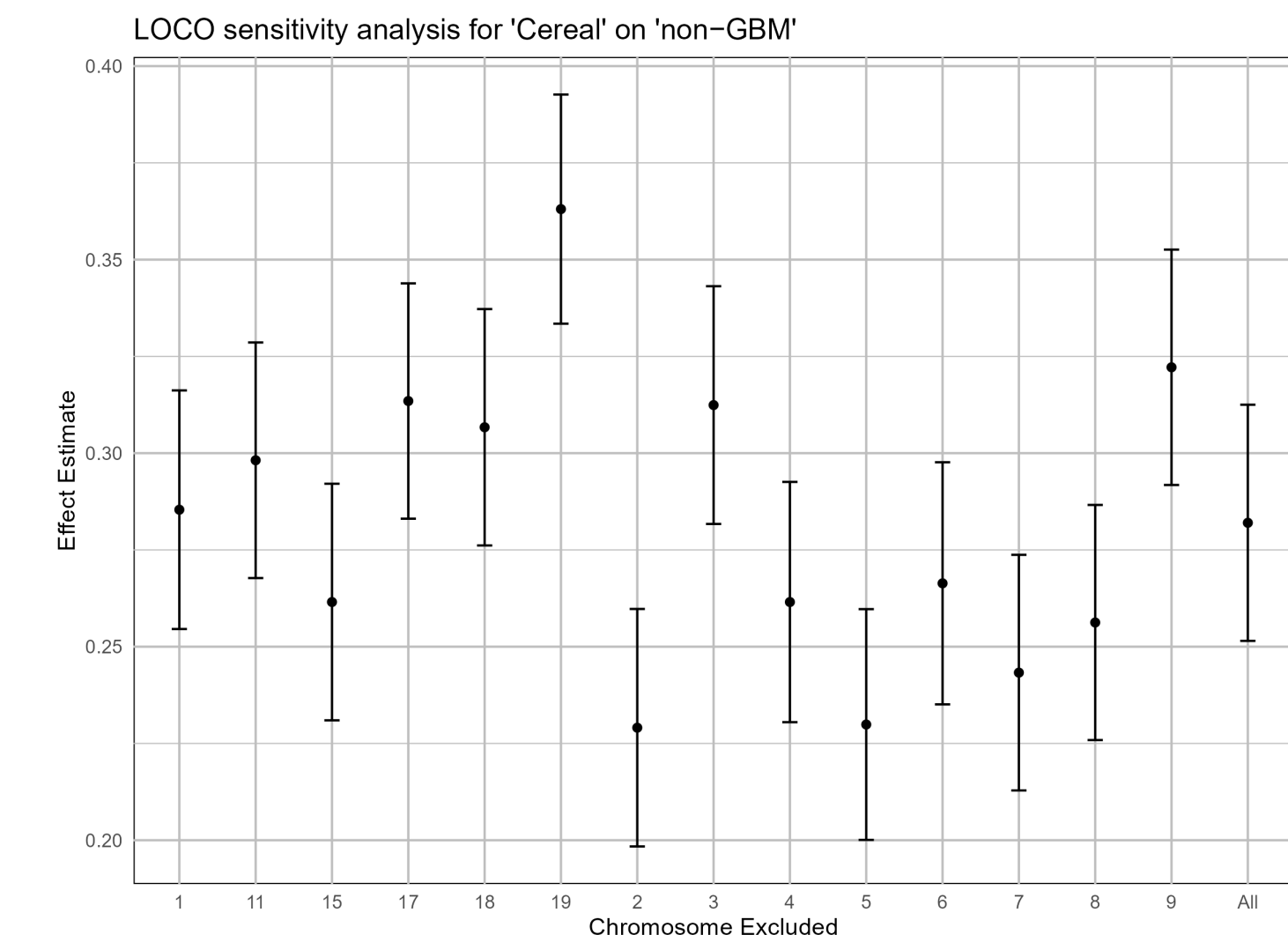

Supplement: Supplementary file 1 [file nutrients-17-00582-s001.zip › nutrients-3462880-supplementary/Sup_26.pdf]

A

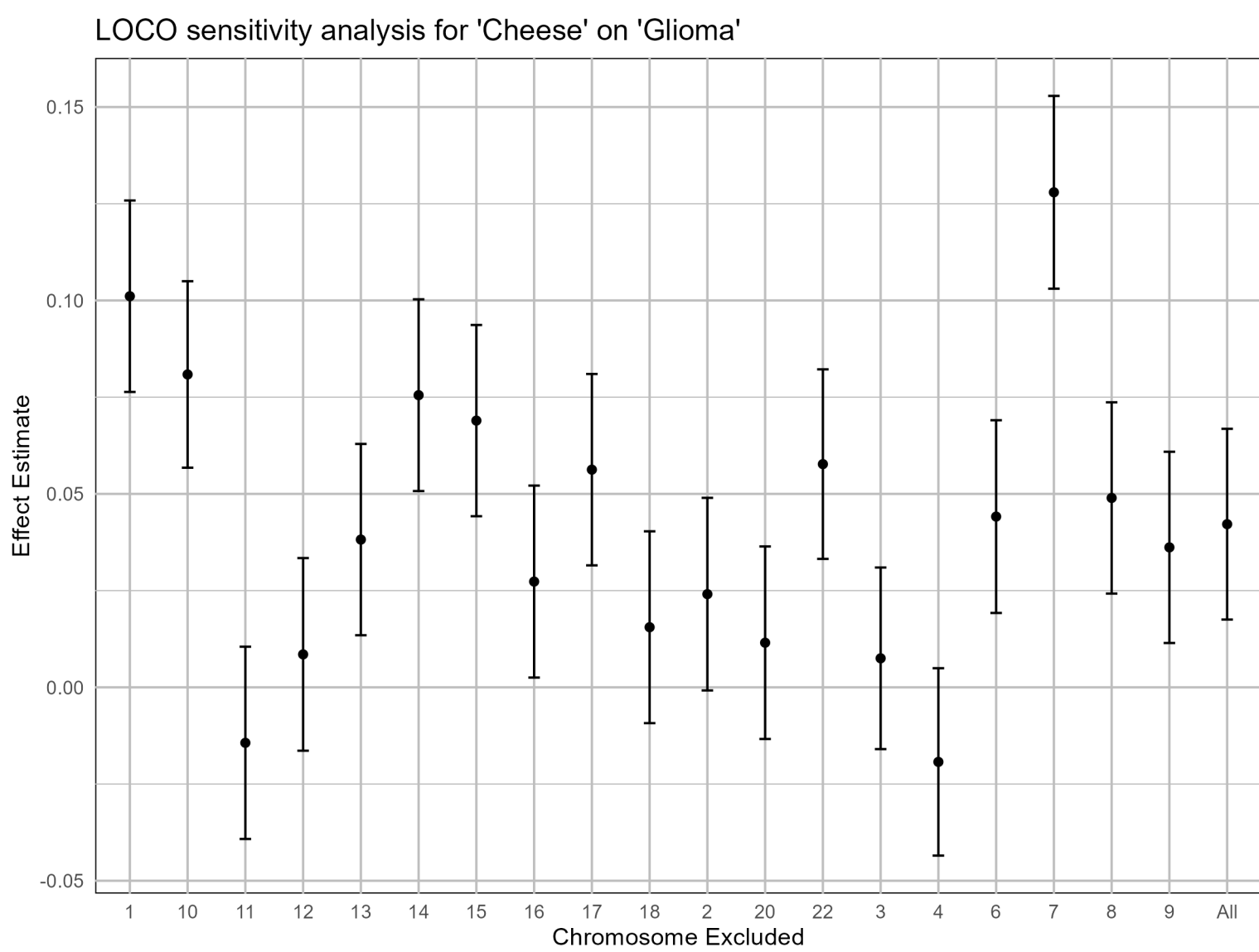

B

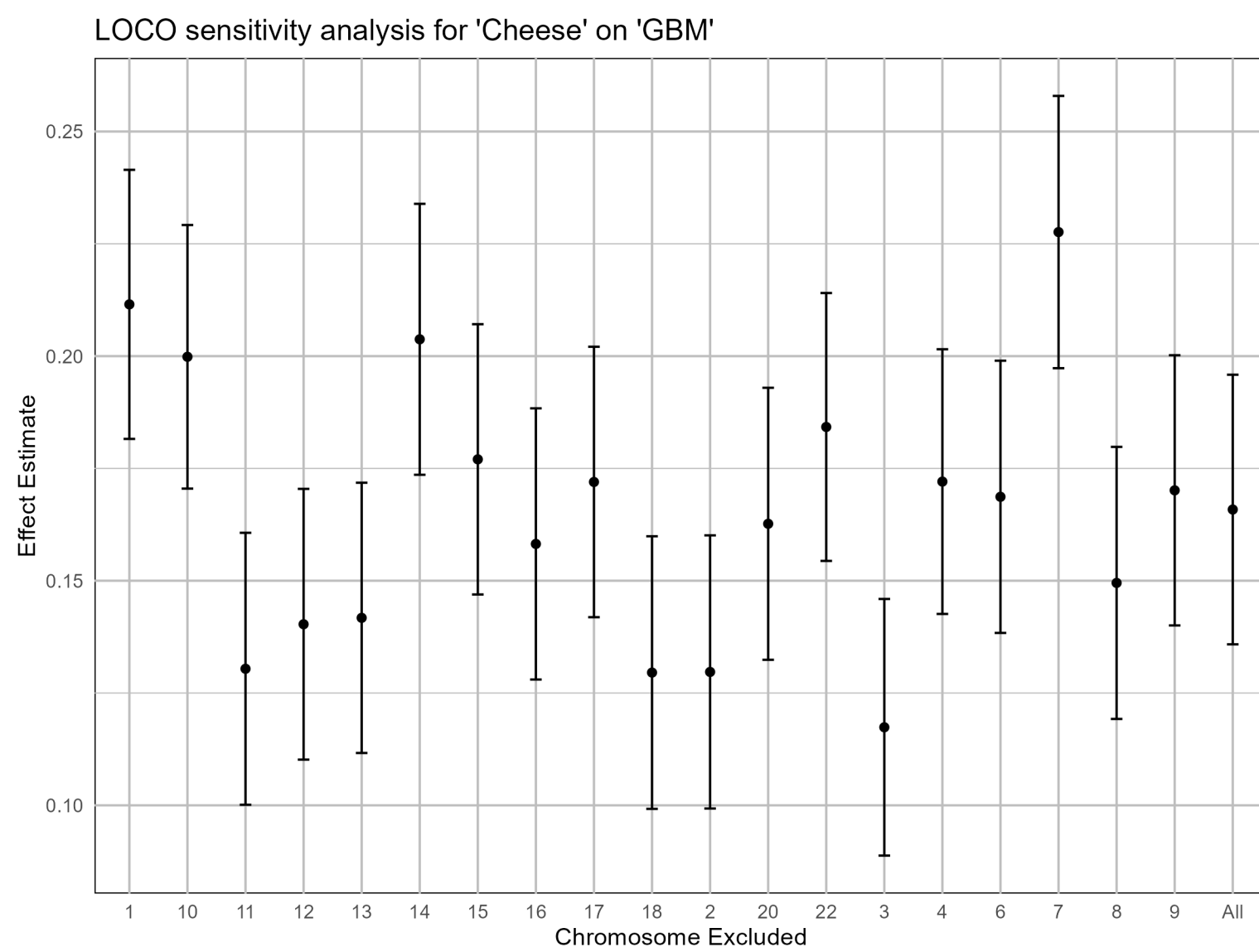

C

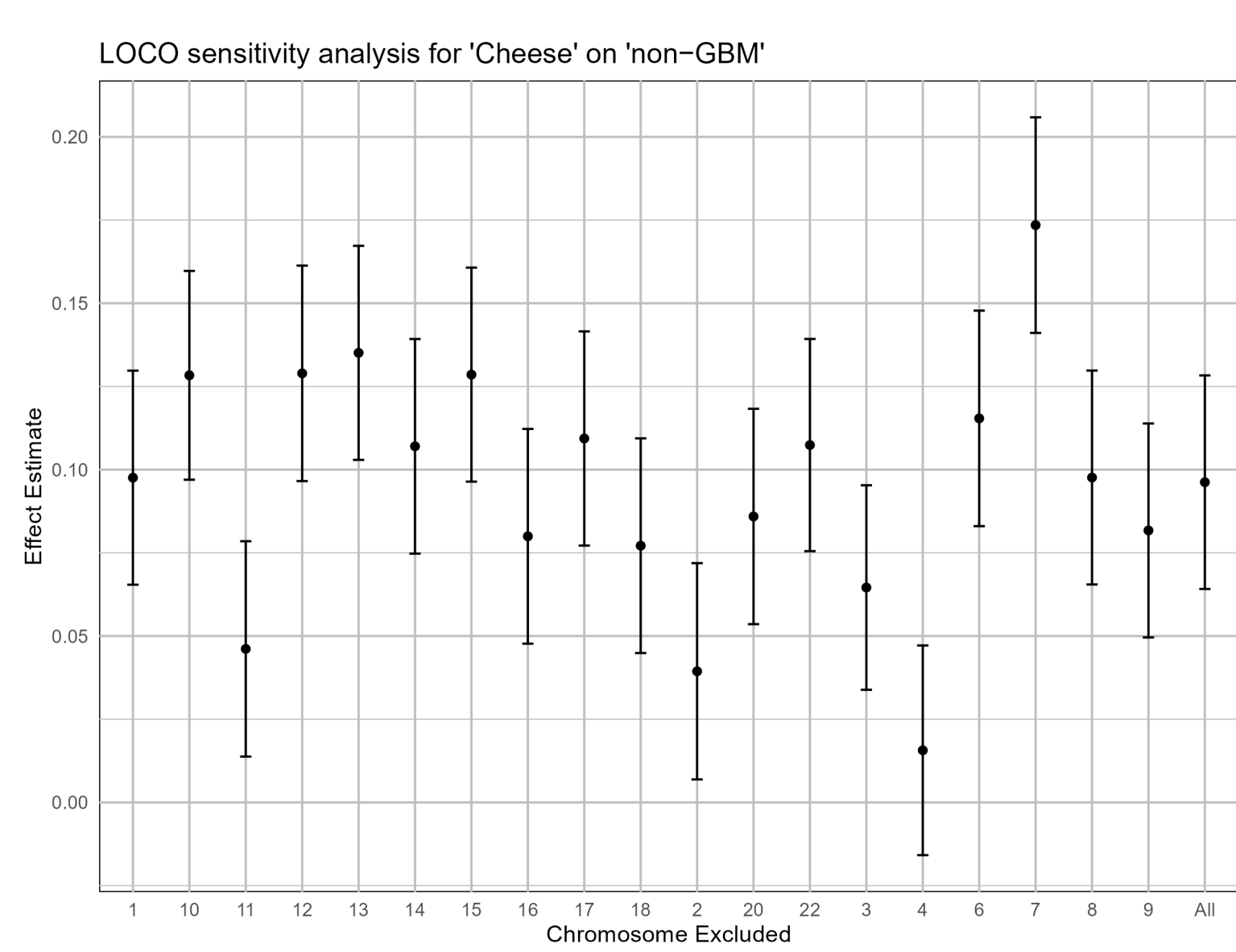

D

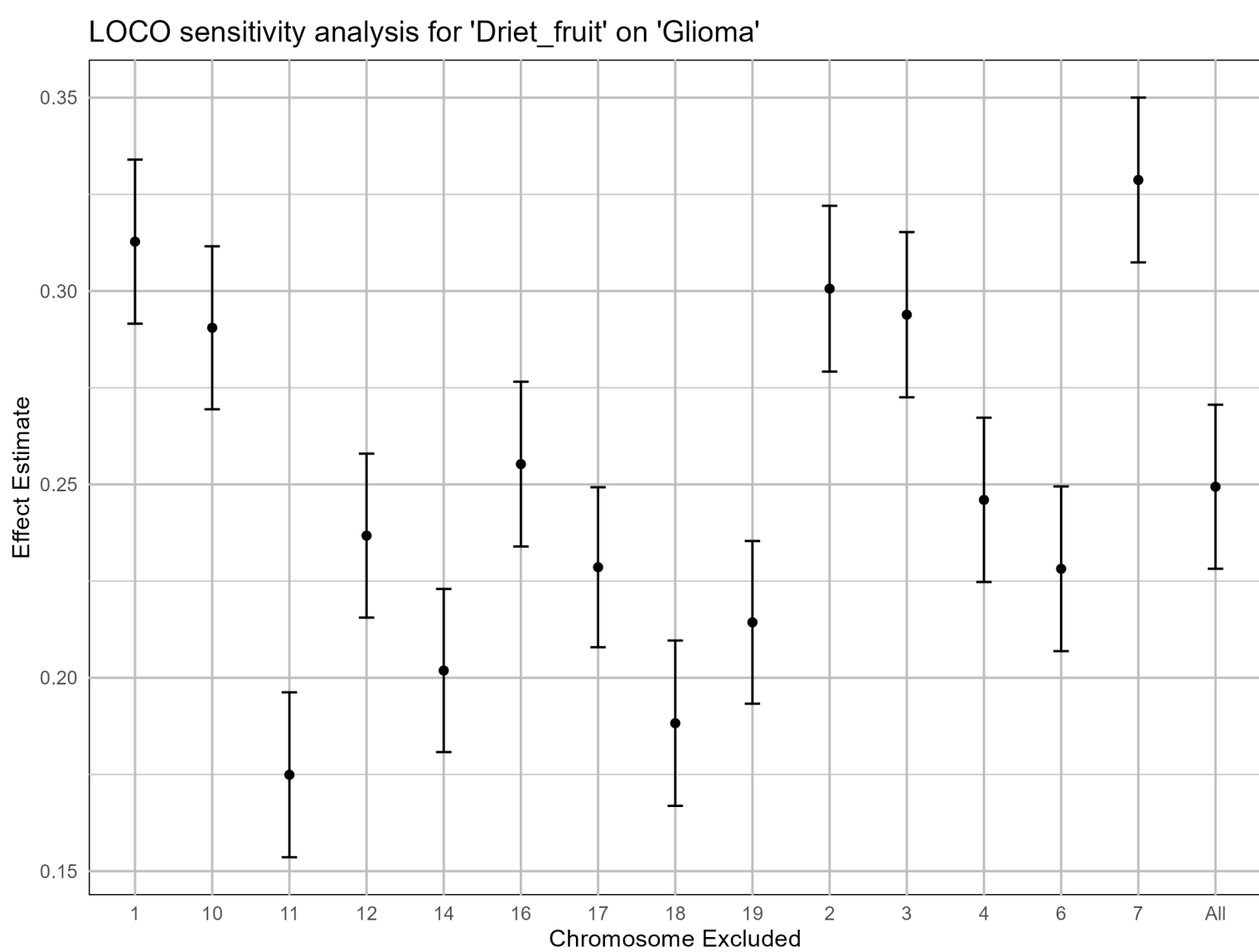

E

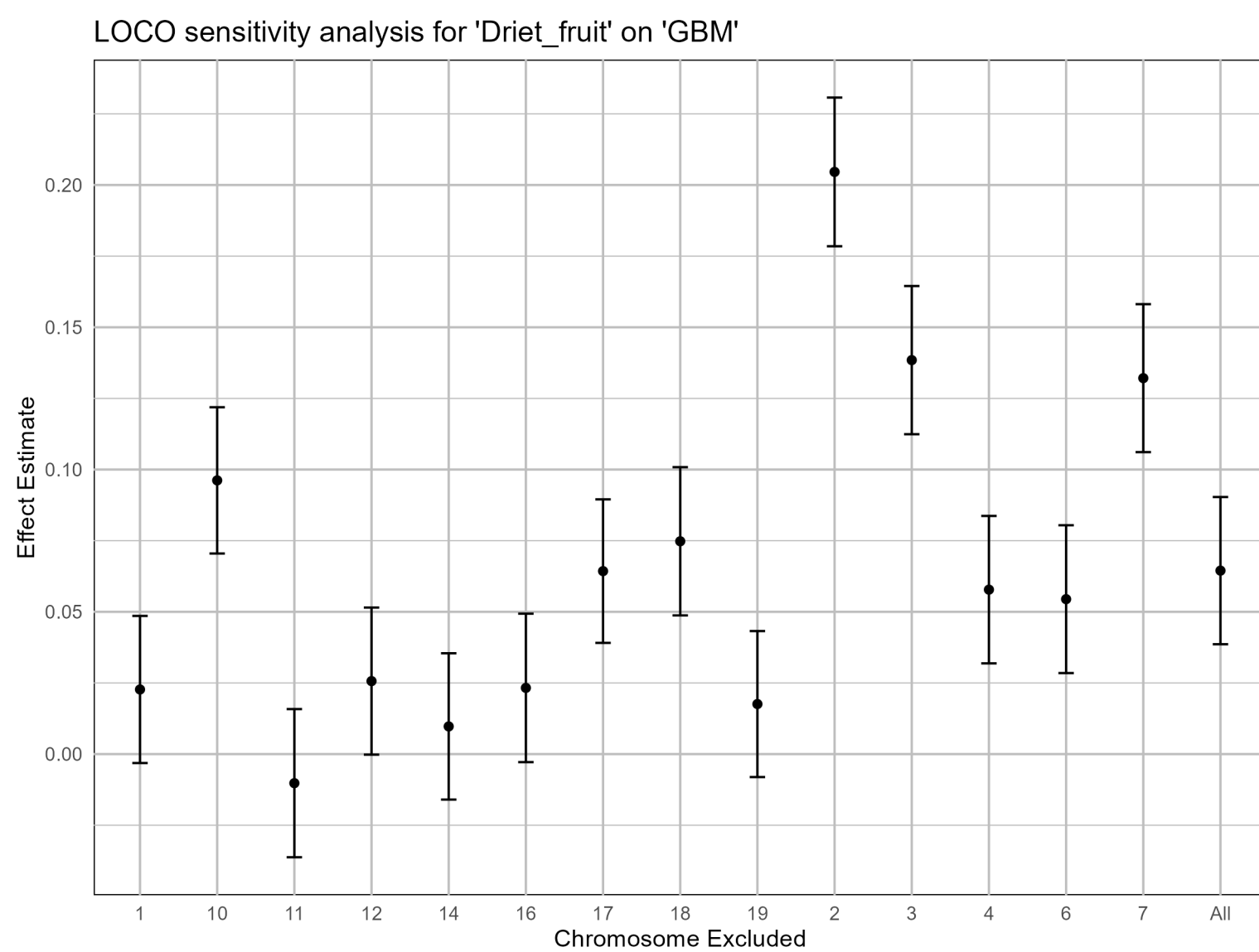

F

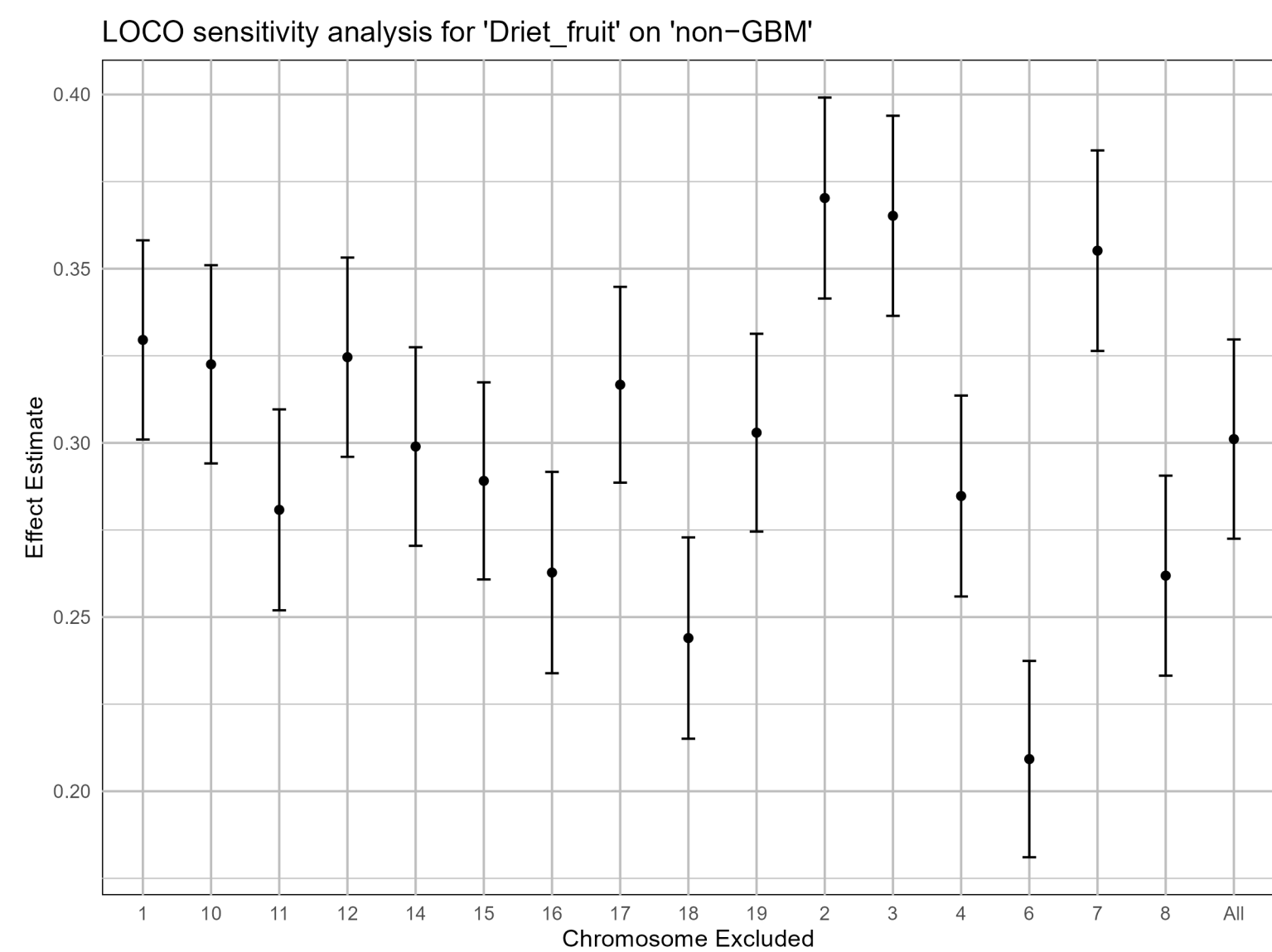

G

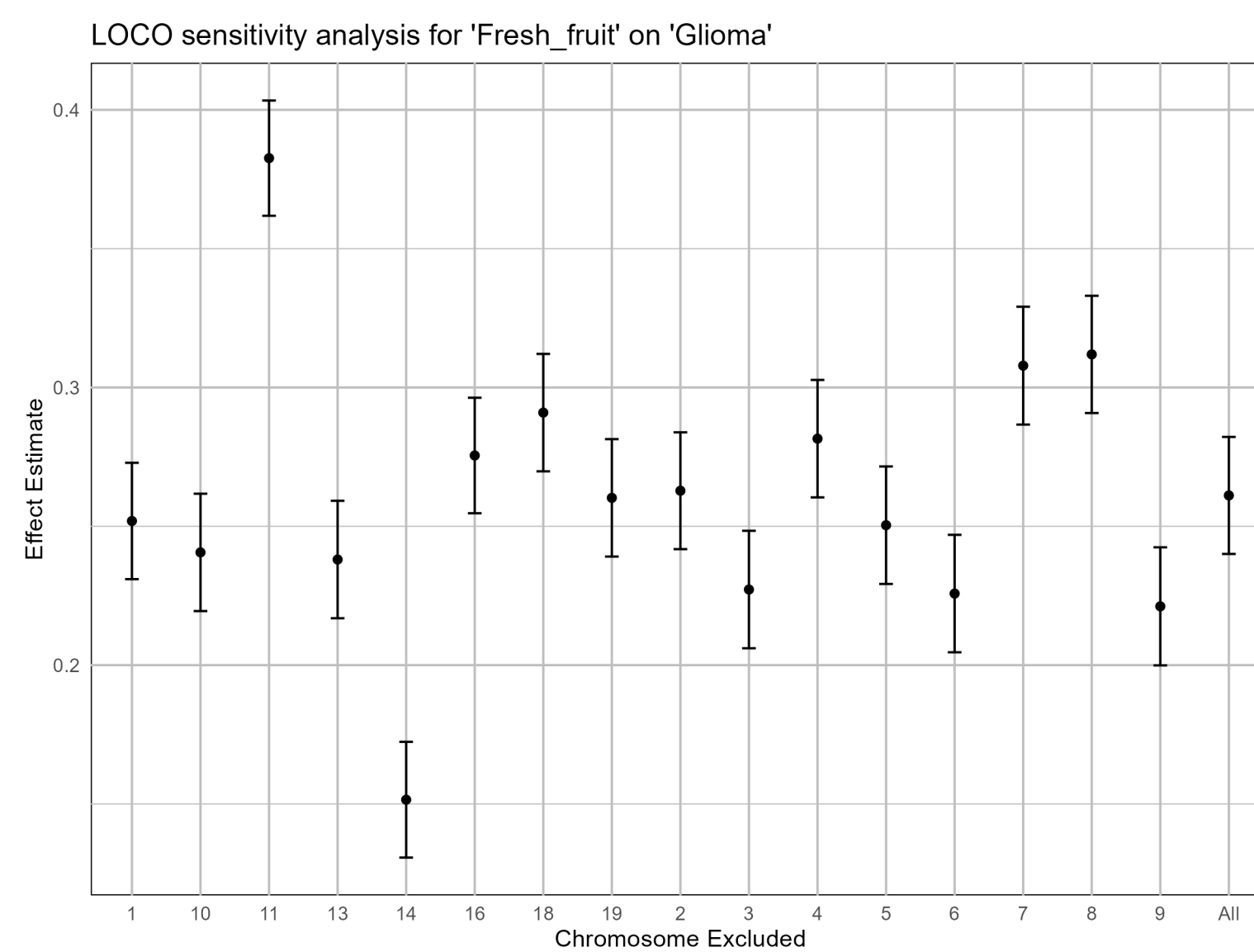

H

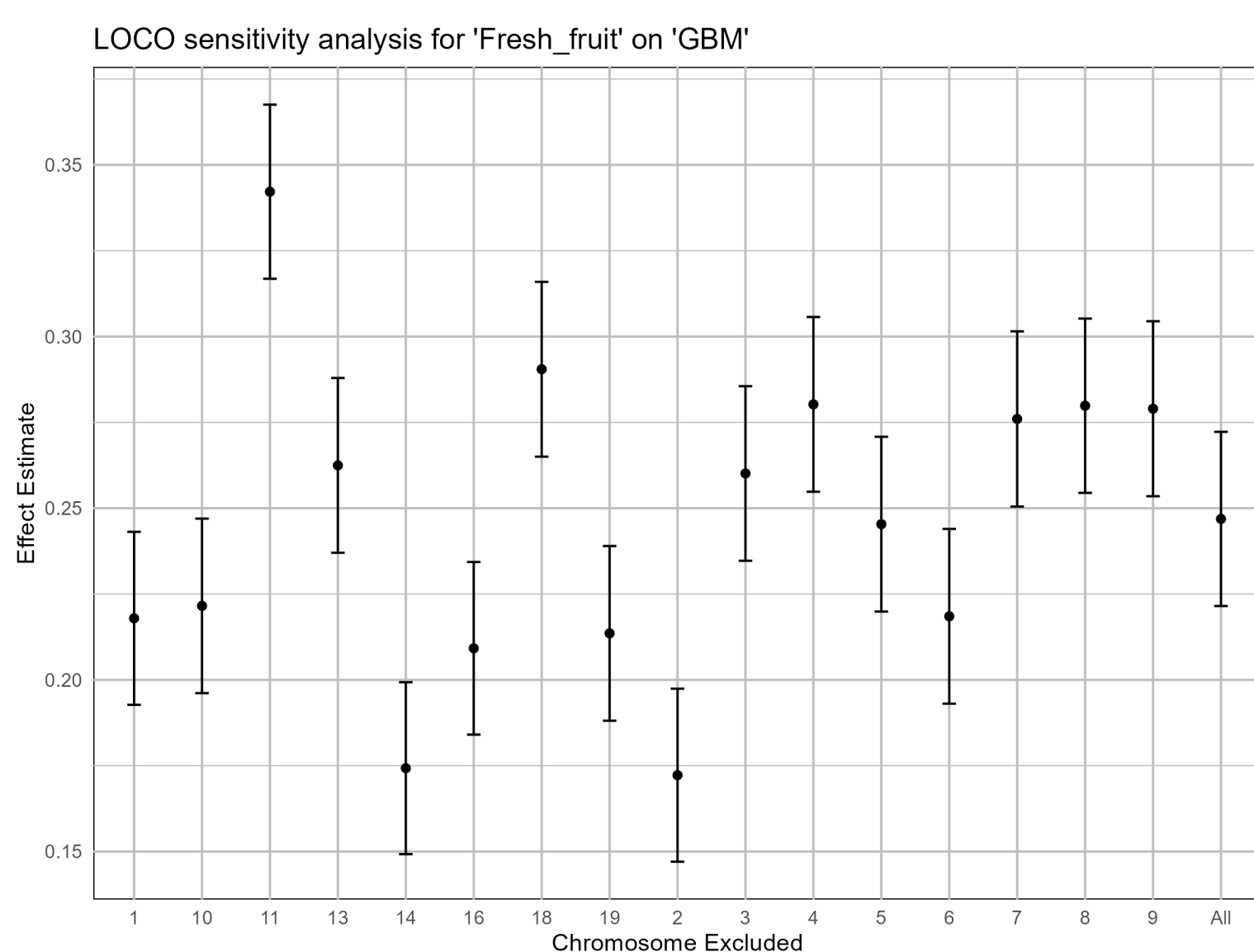

I

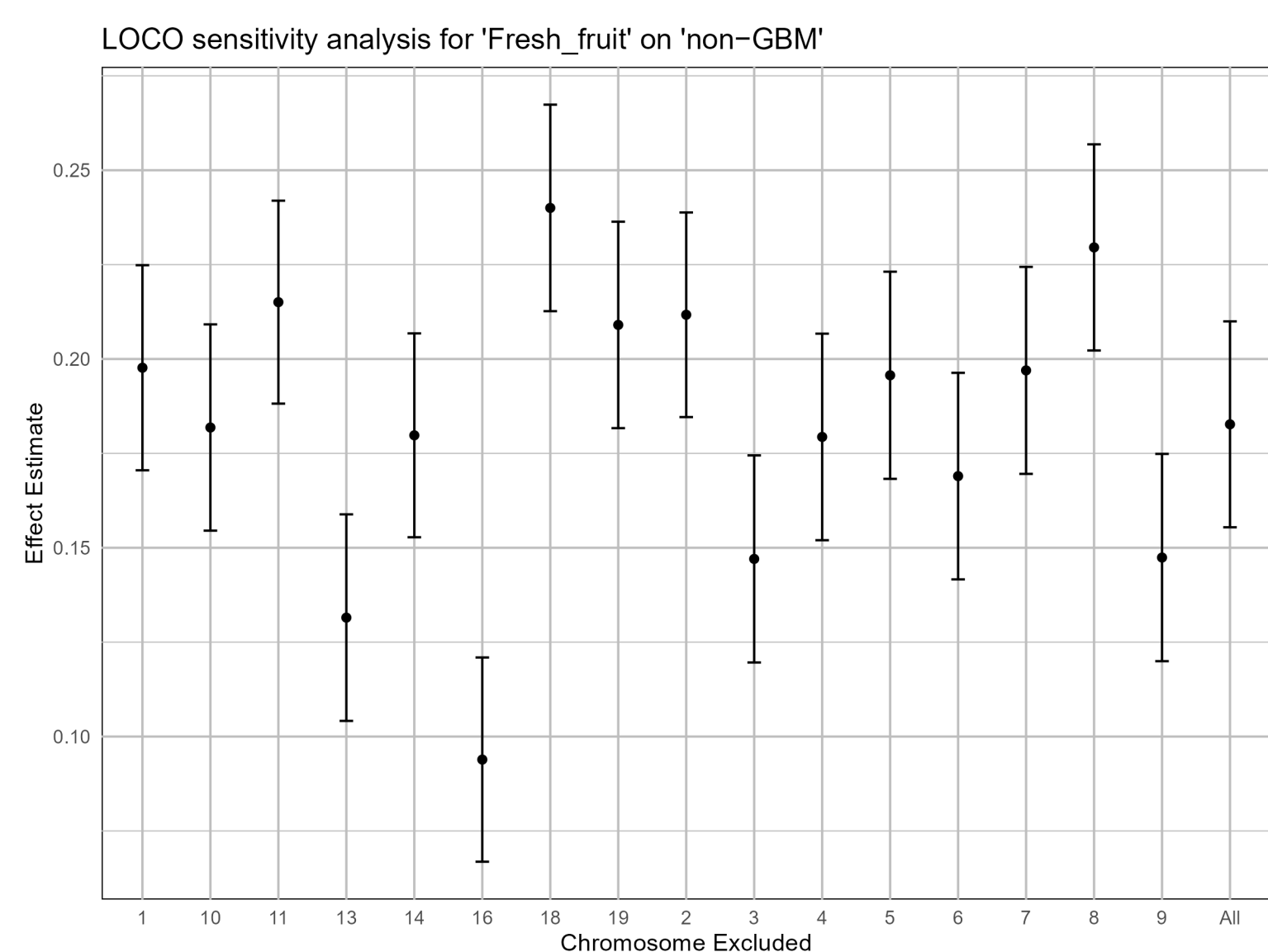

Supplement: Supplementary file 1 [file nutrients-17-00582-s001.zip › nutrients-3462880-supplementary/Sup_27.pdf]

A

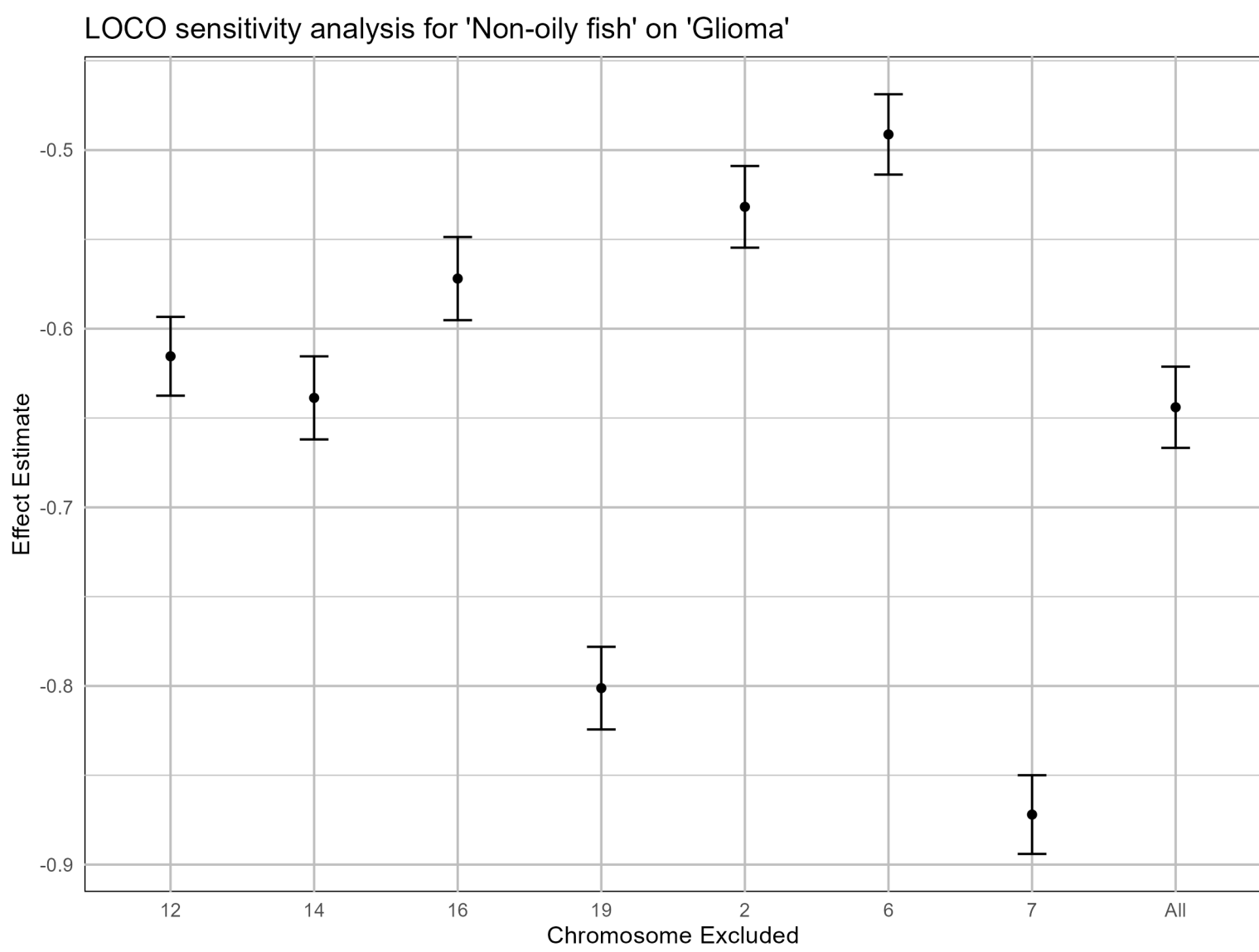

B

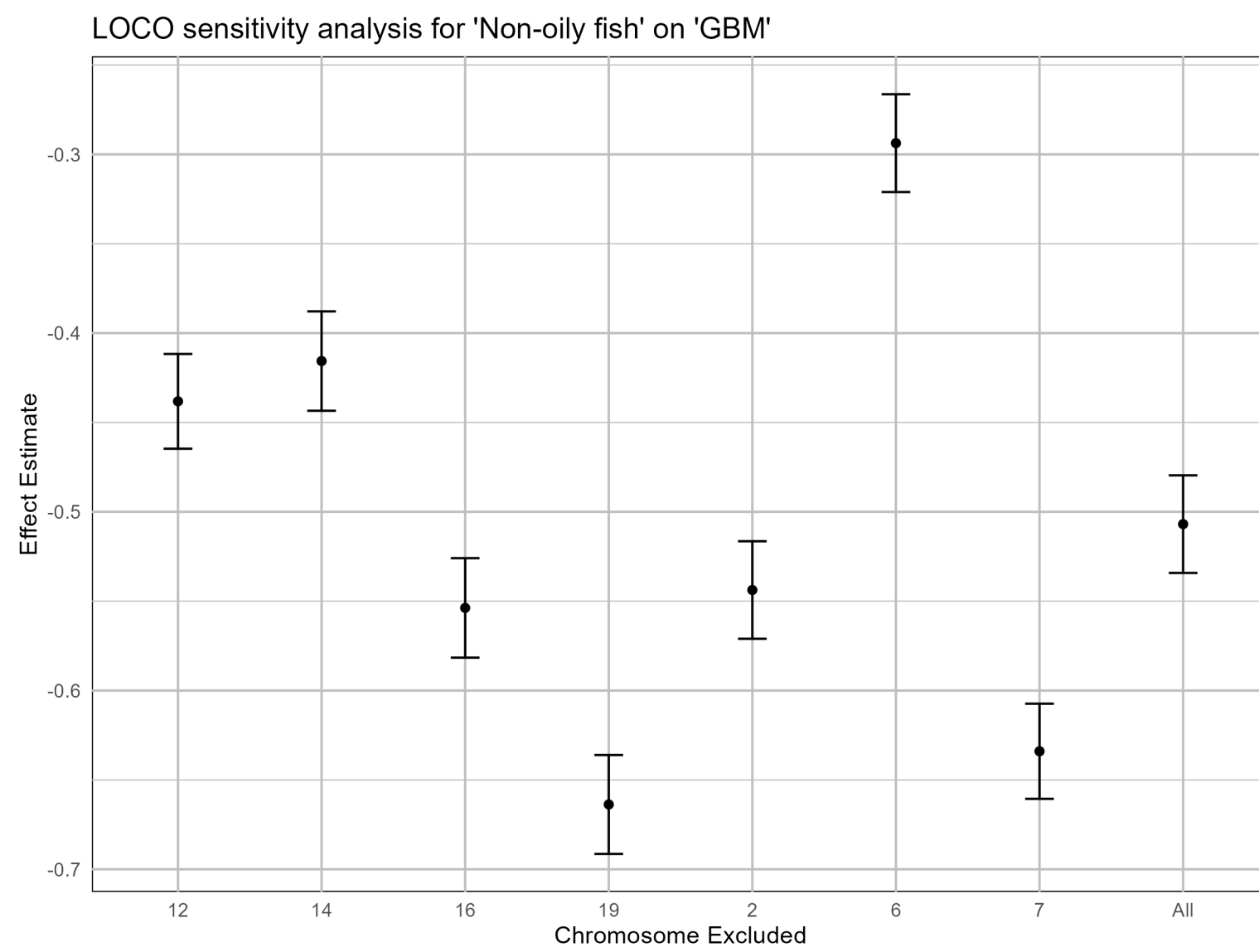

C

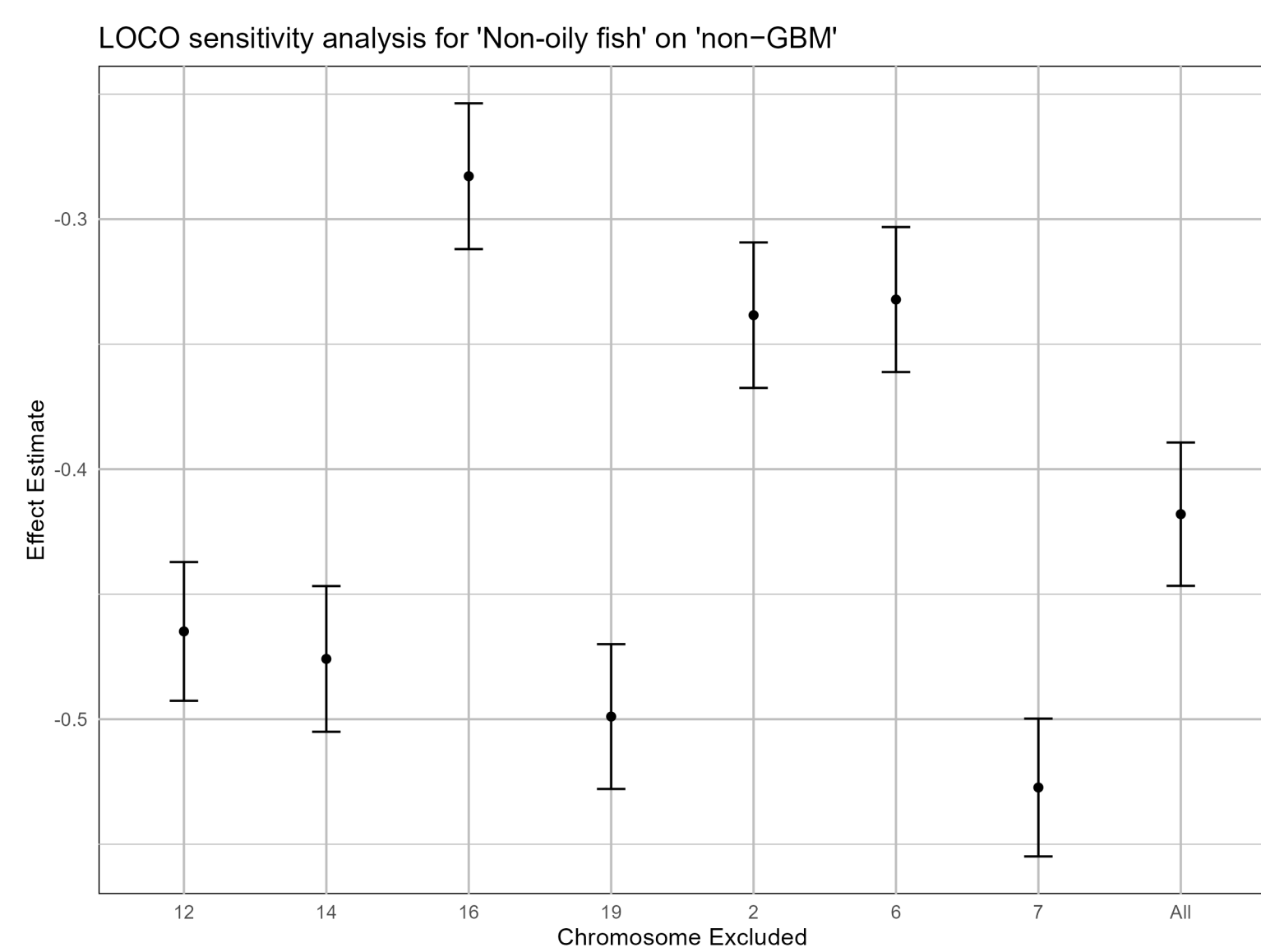

D

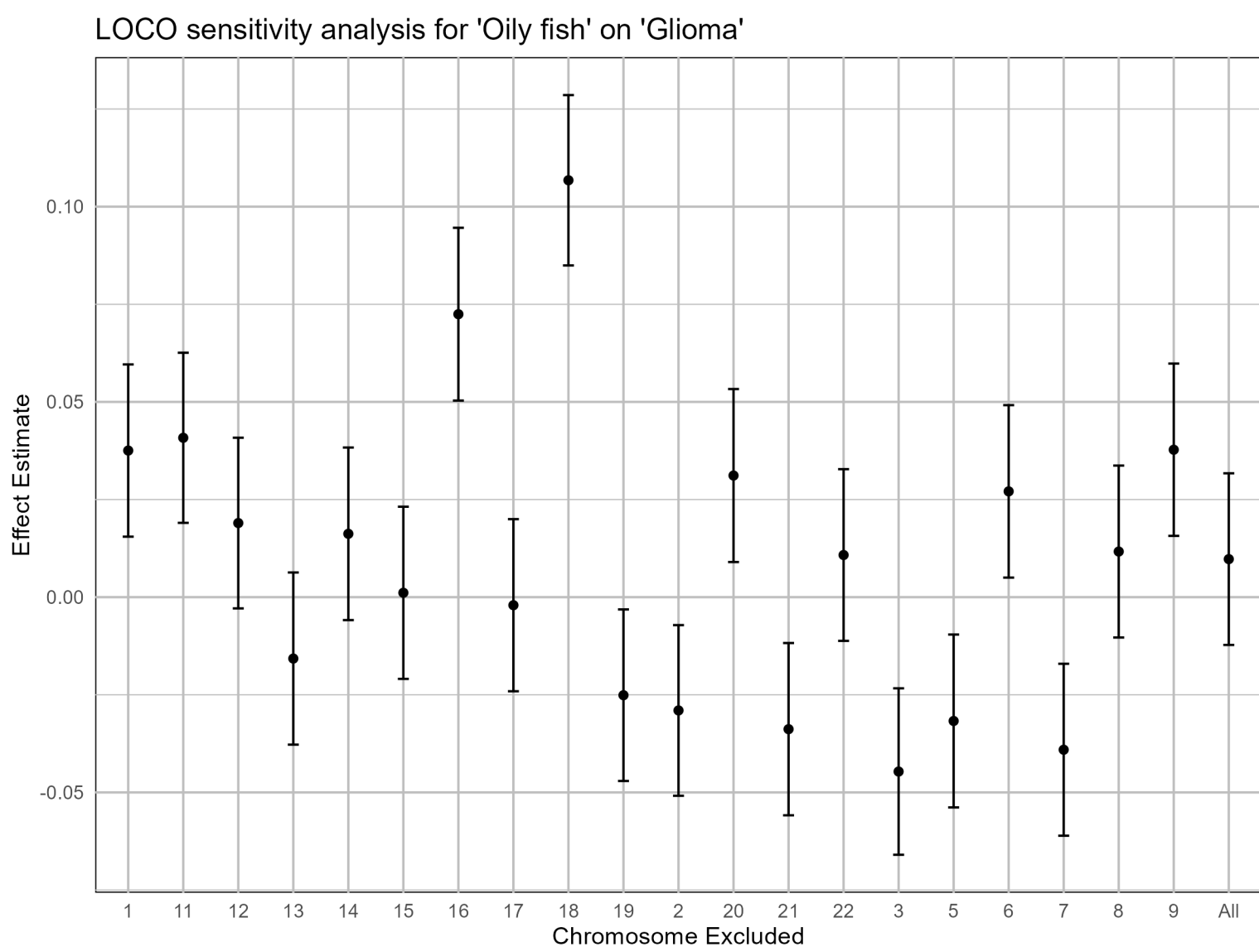

E

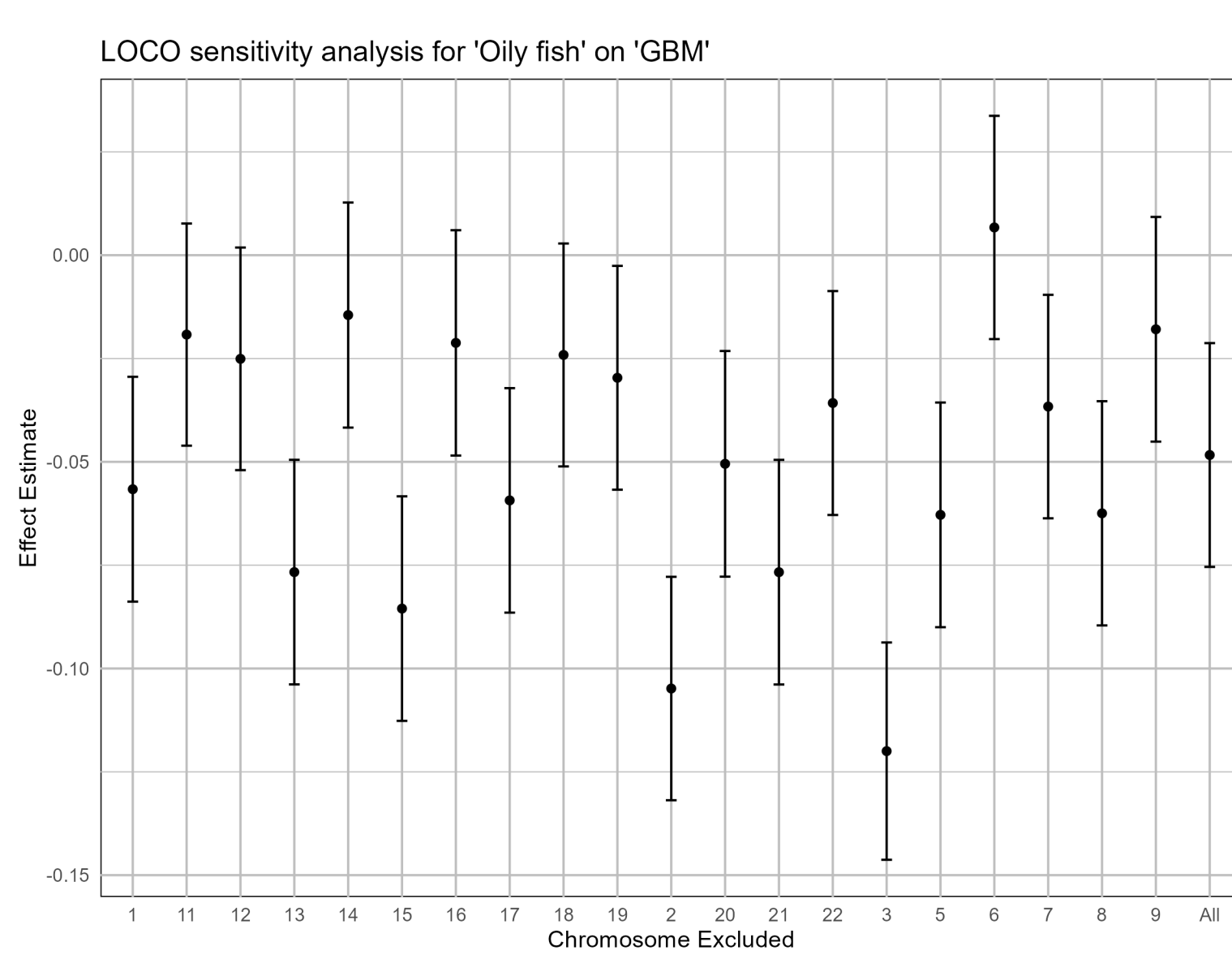

F

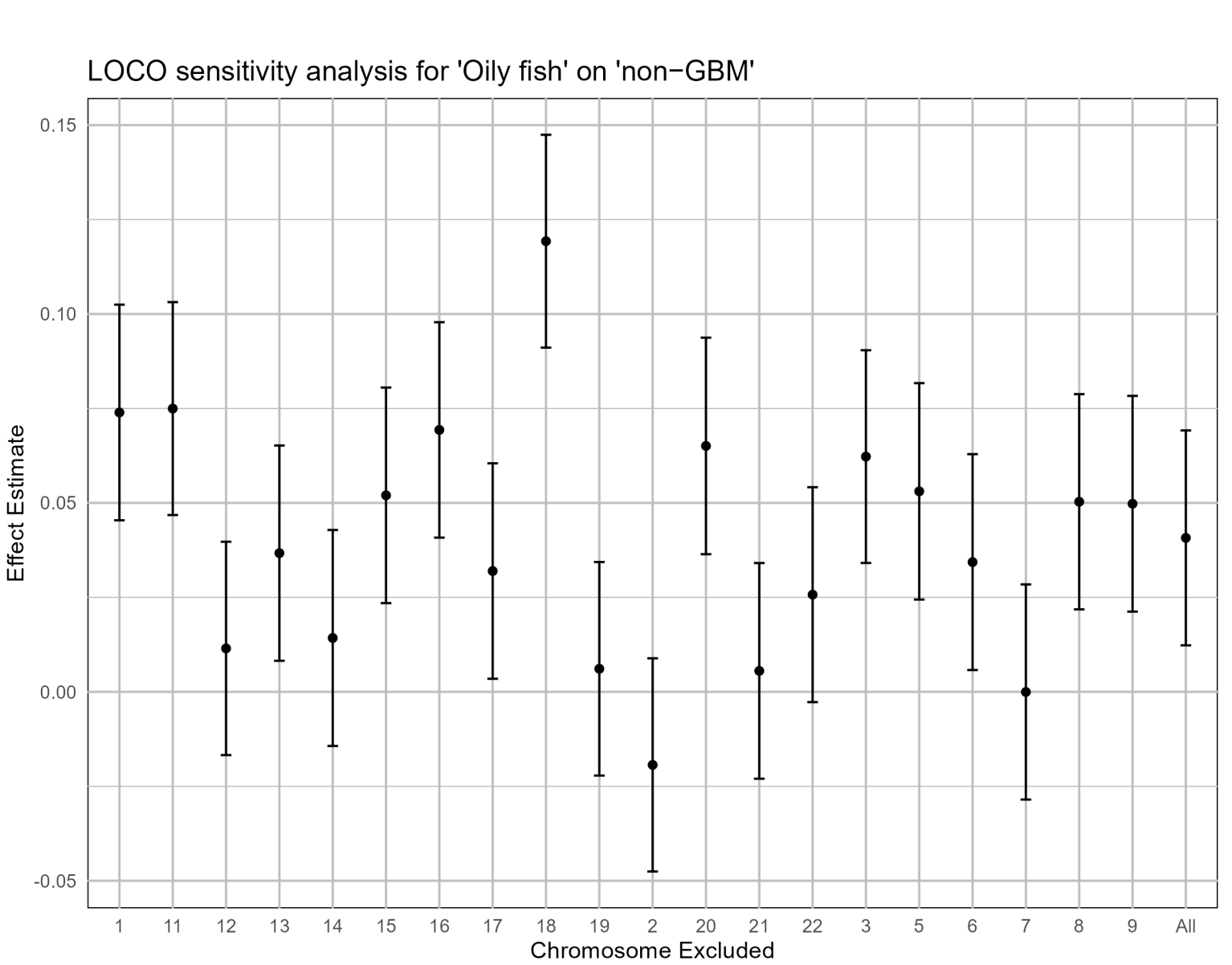

G

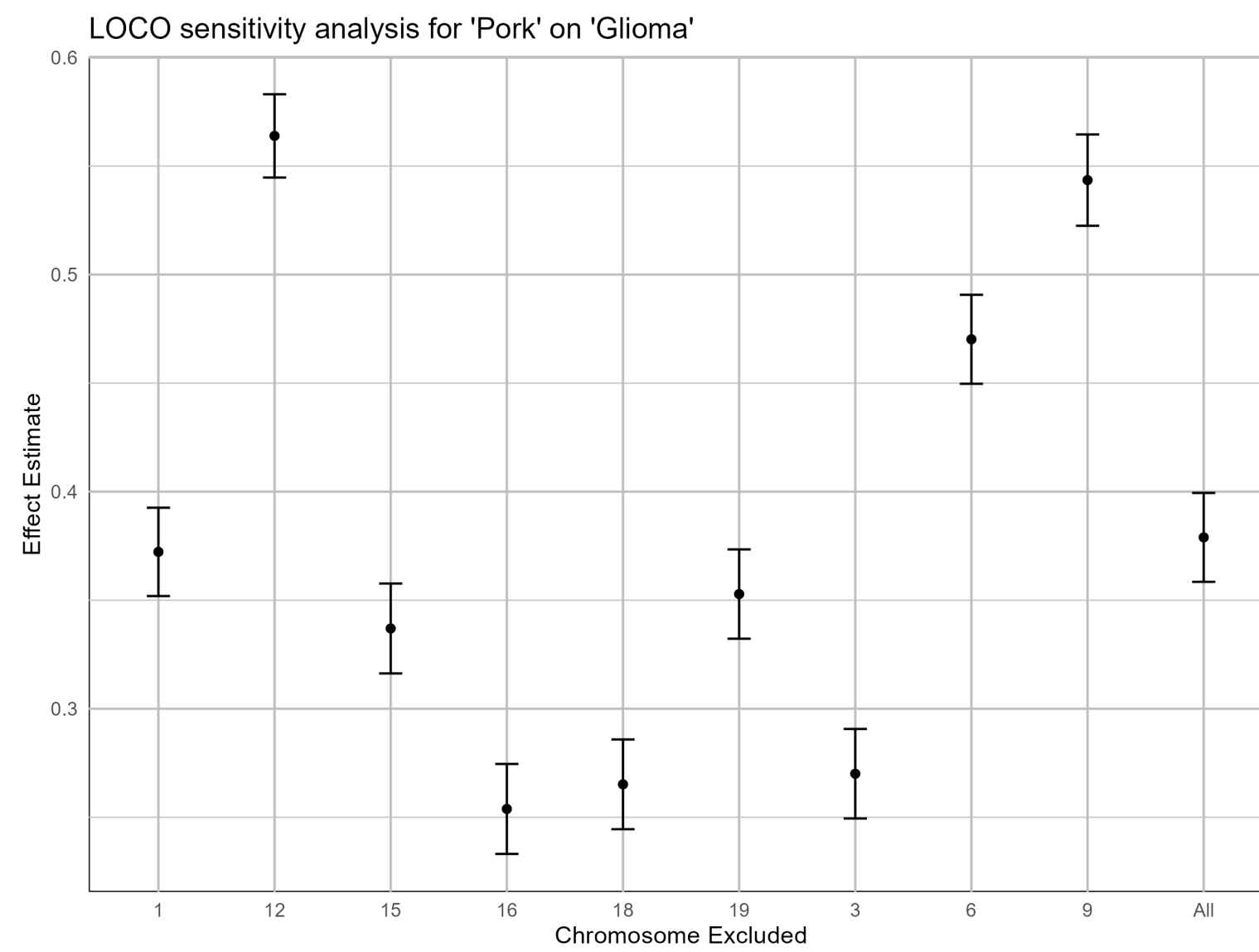

H

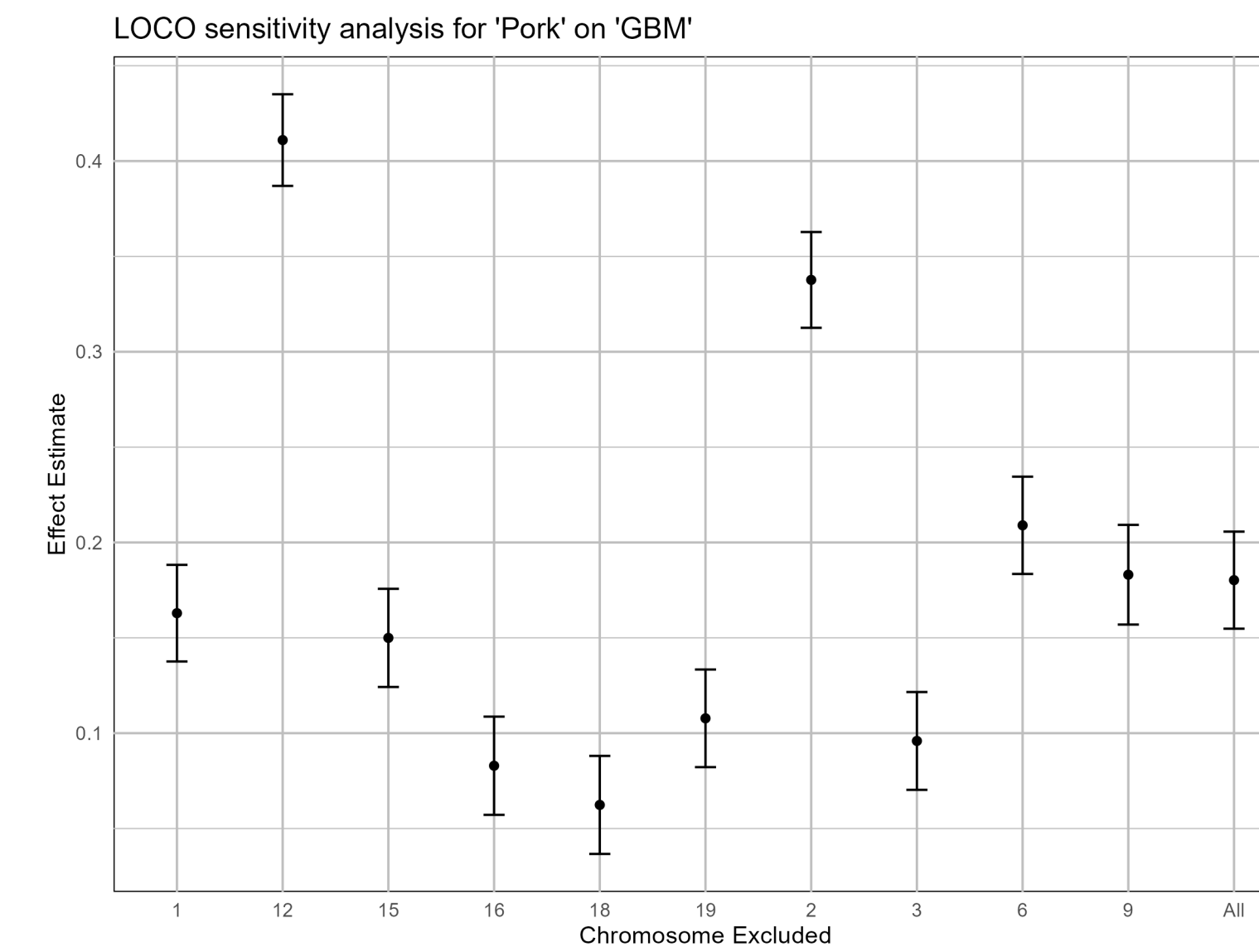

I

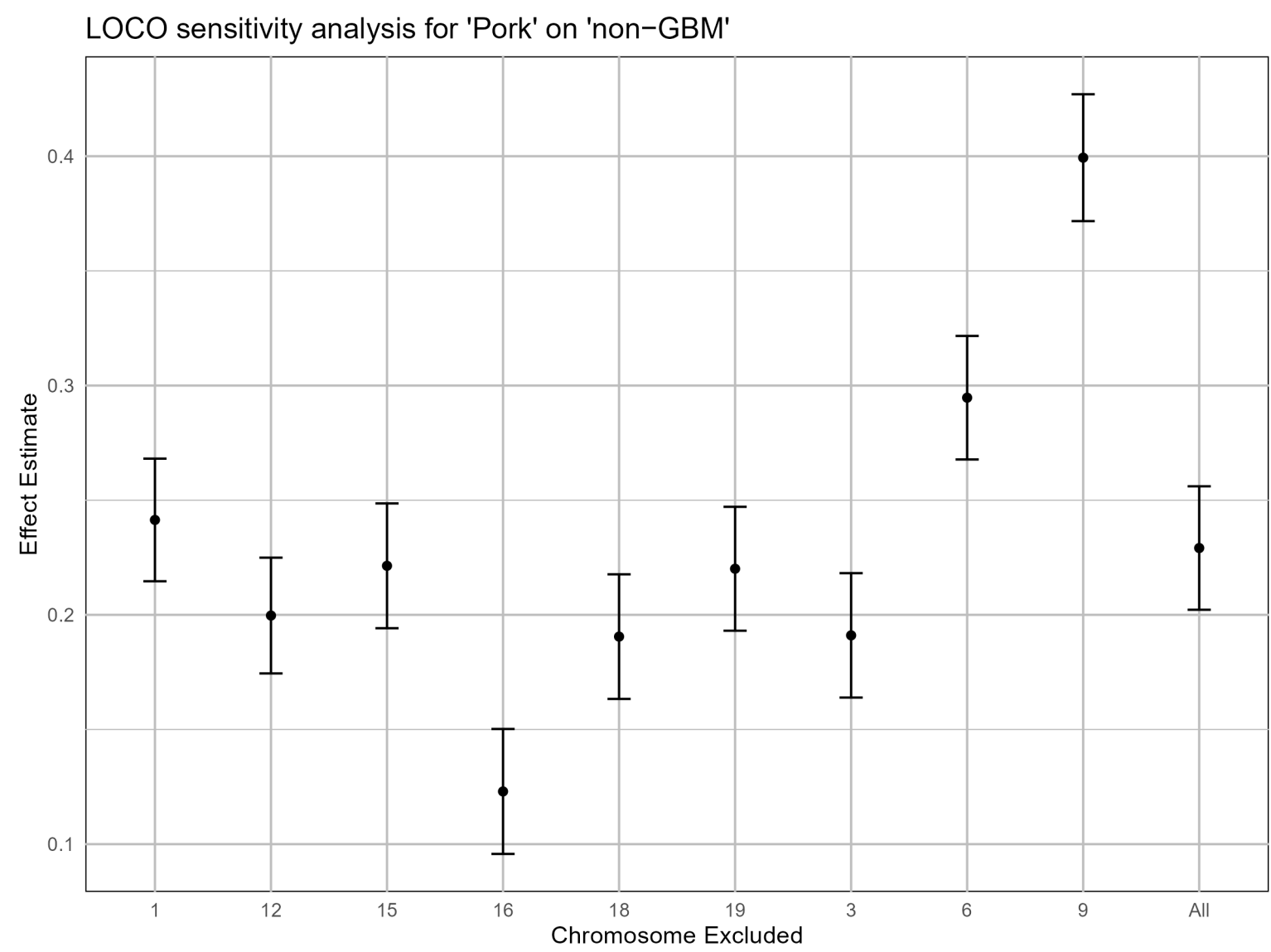

Supplement: Supplementary file 1 [file nutrients-17-00582-s001.zip › nutrients-3462880-supplementary/Sup_28.pdf]

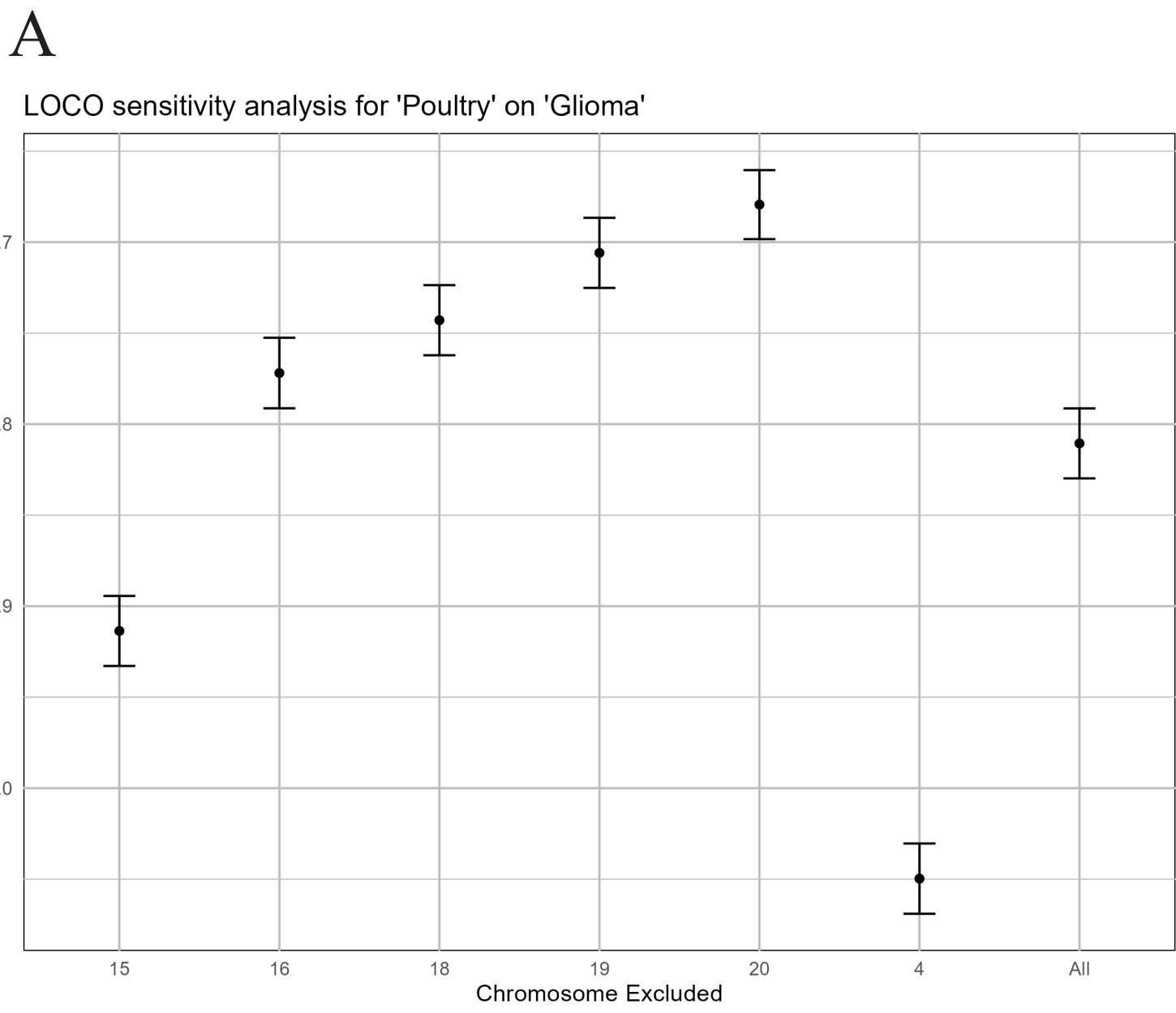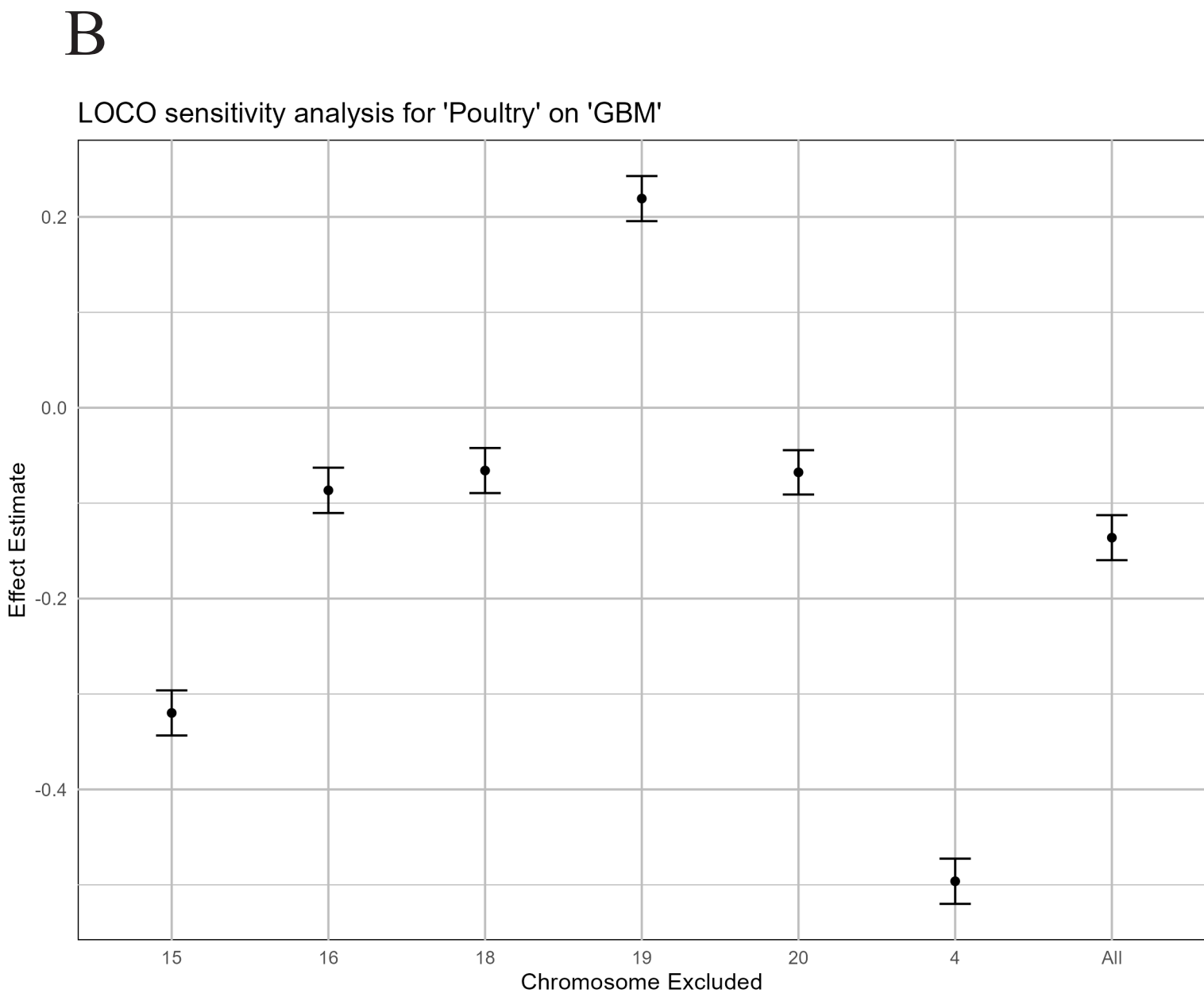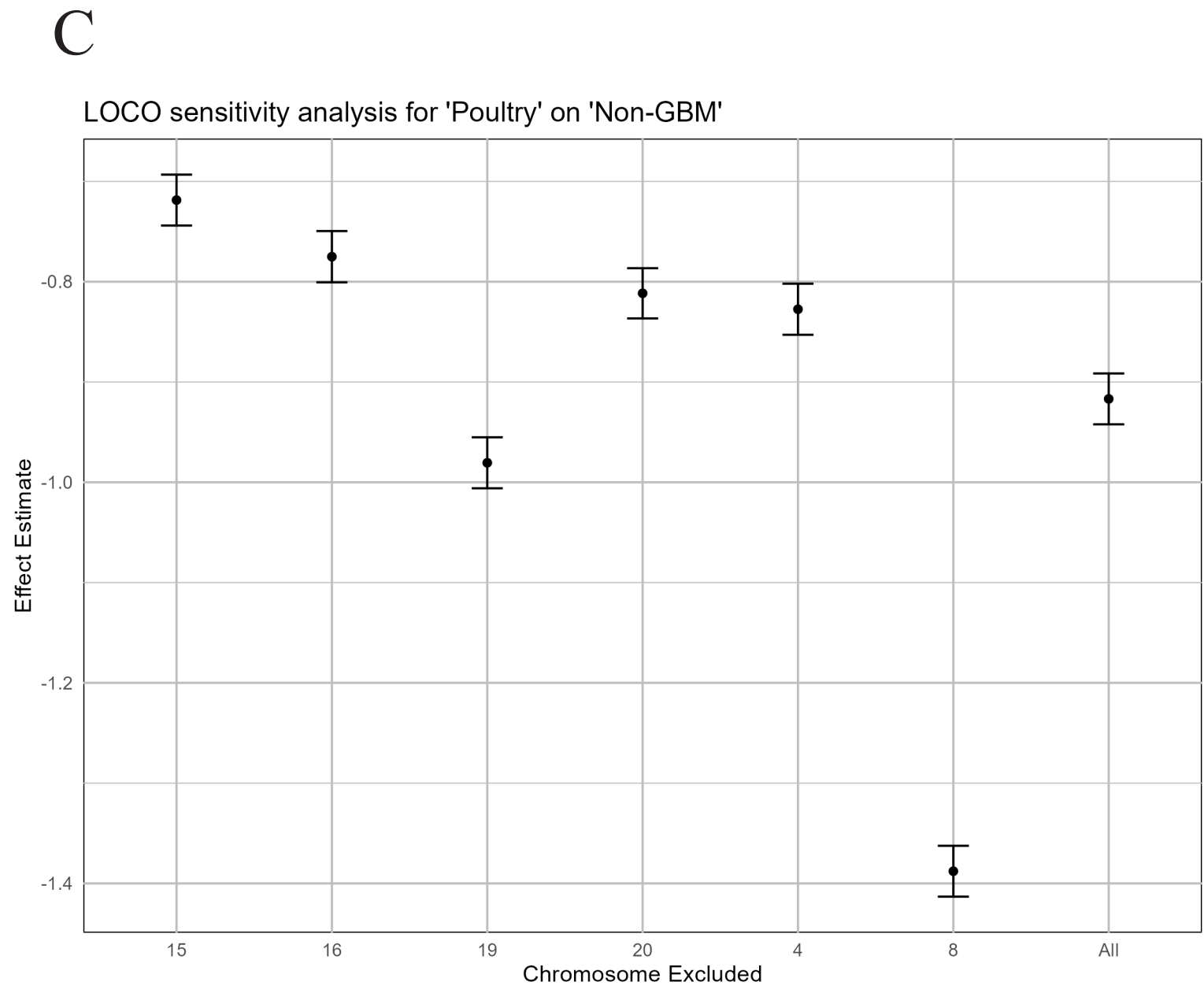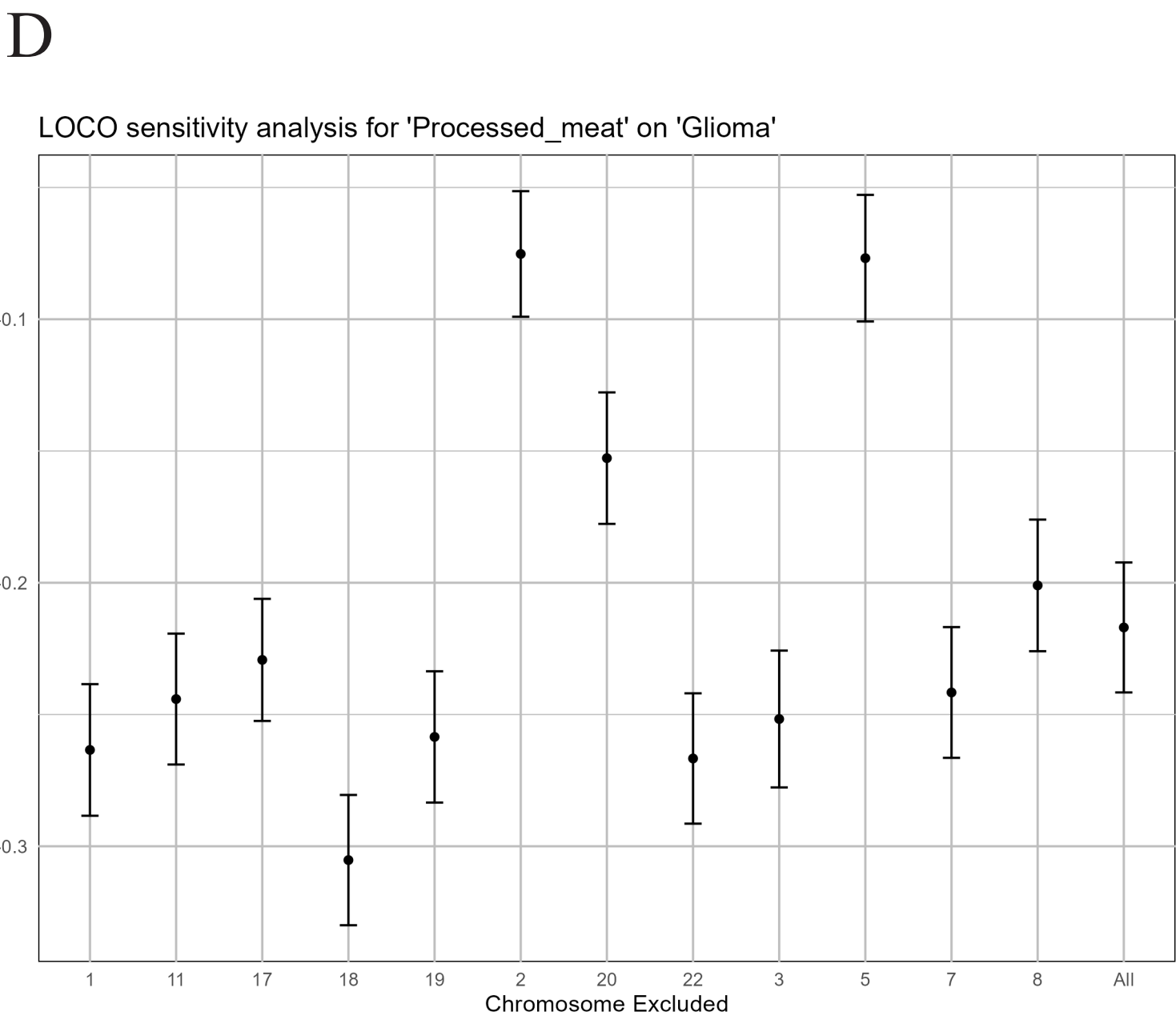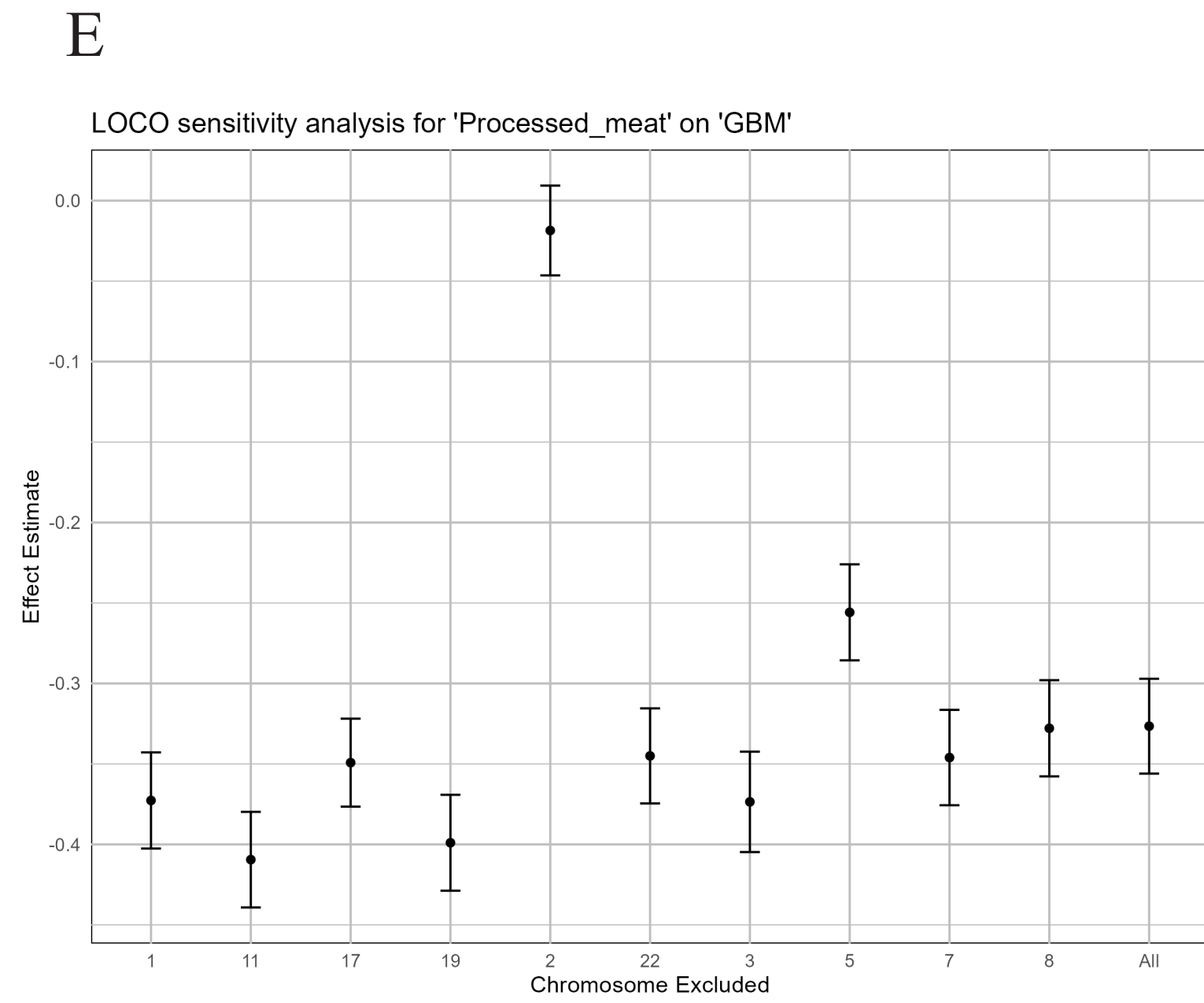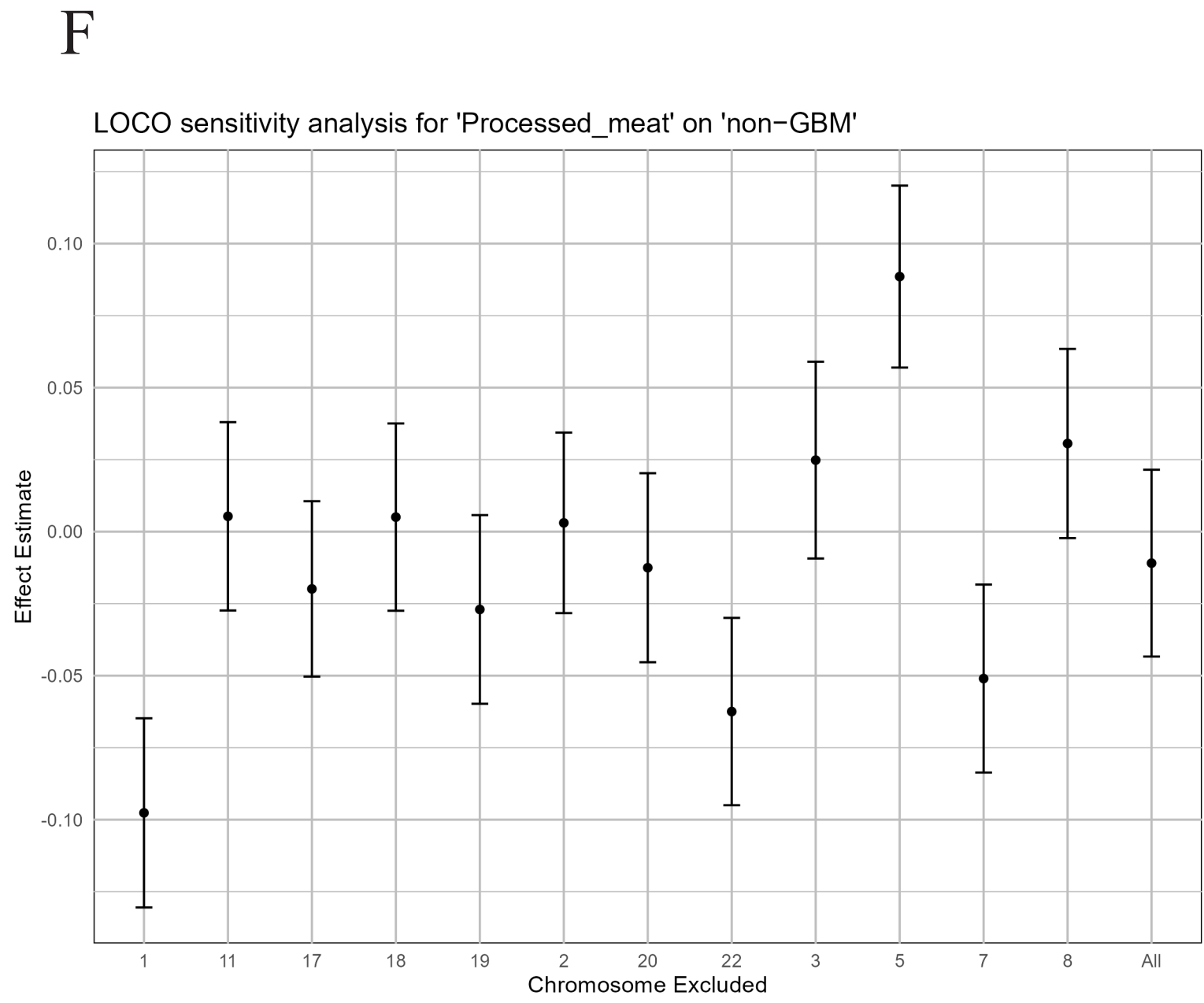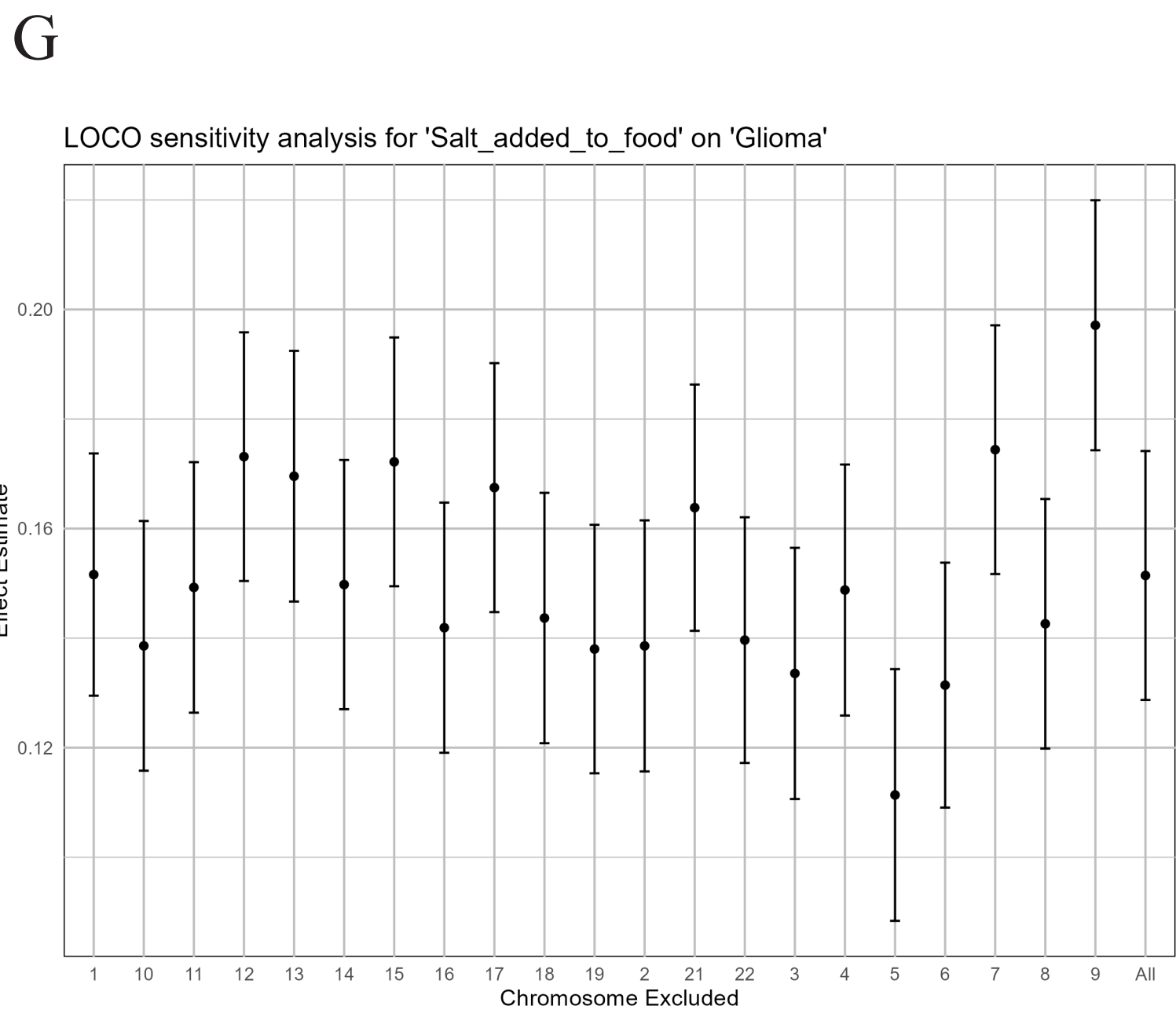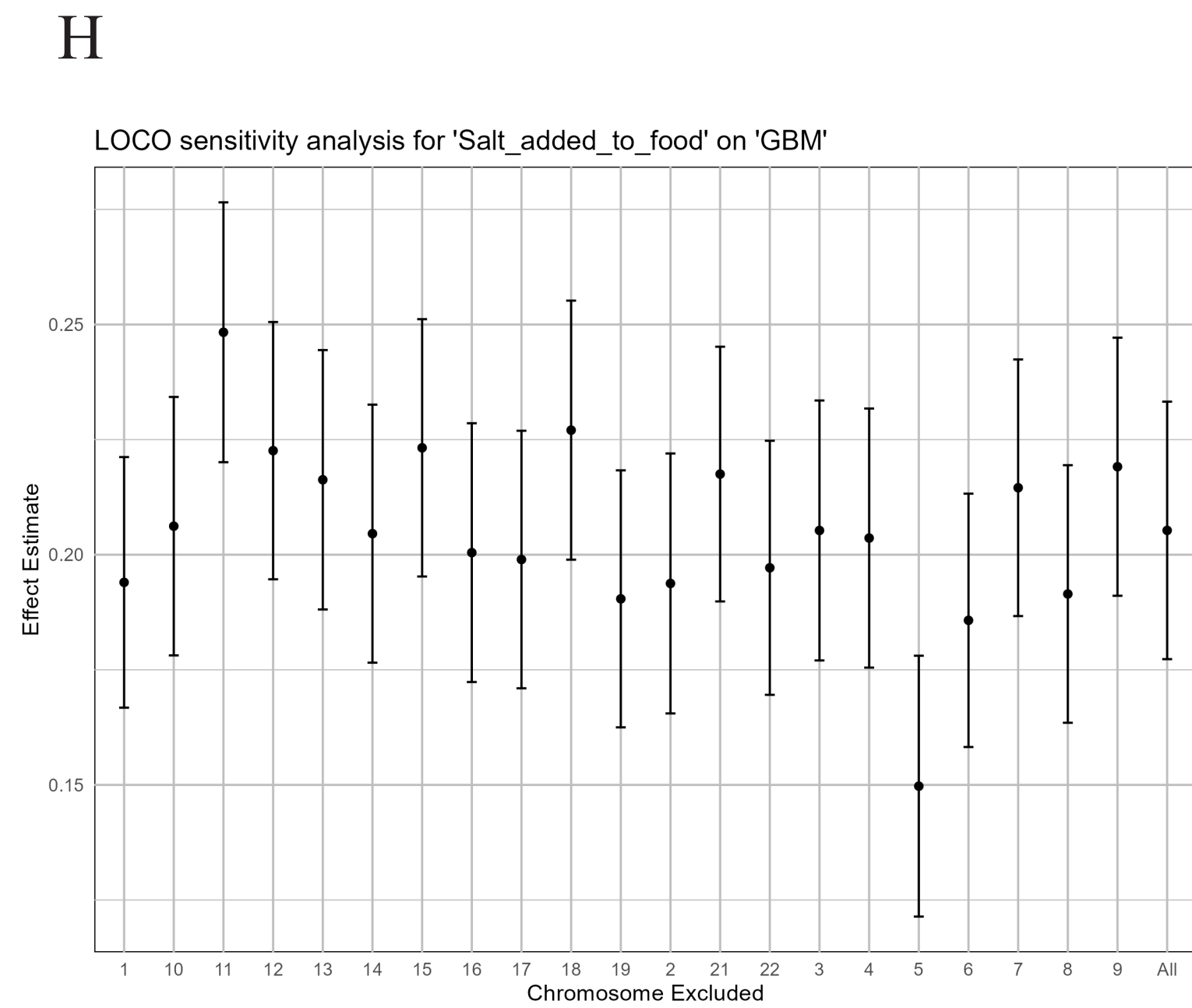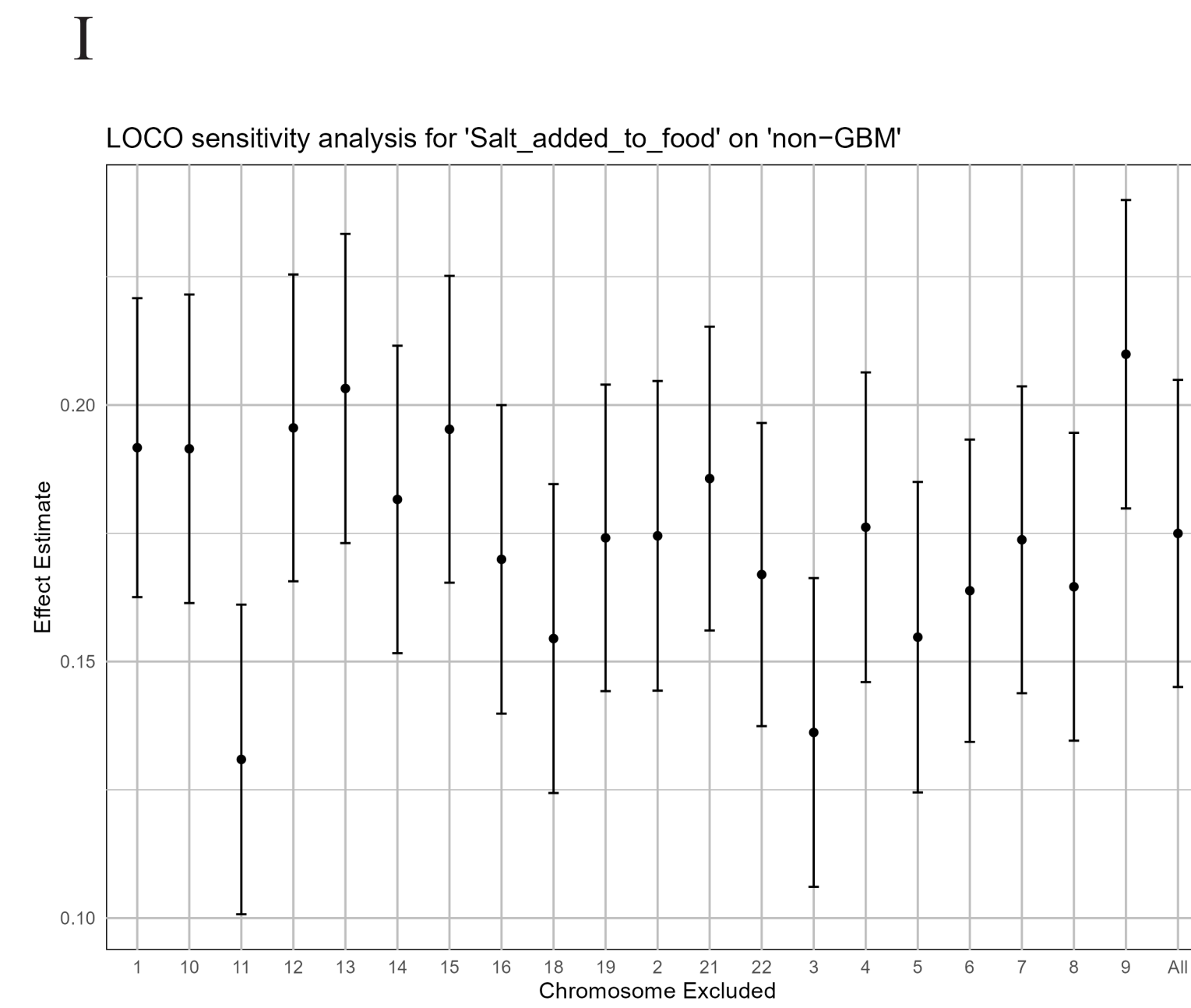

Supplement: Supplementary file 1 [file nutrients-17-00582-s001.zip › nutrients-3462880-supplementary/Sup_29.pdf]

A

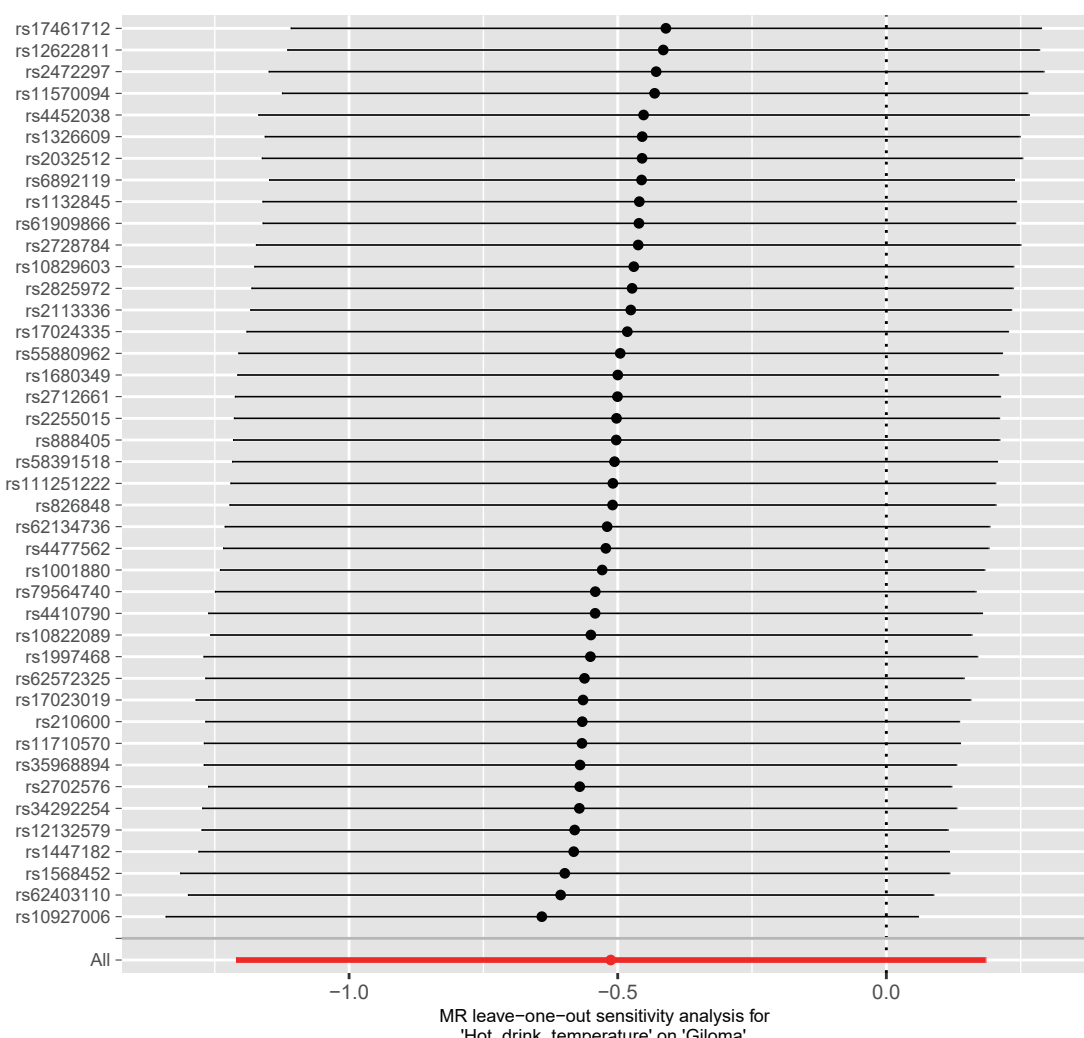

B

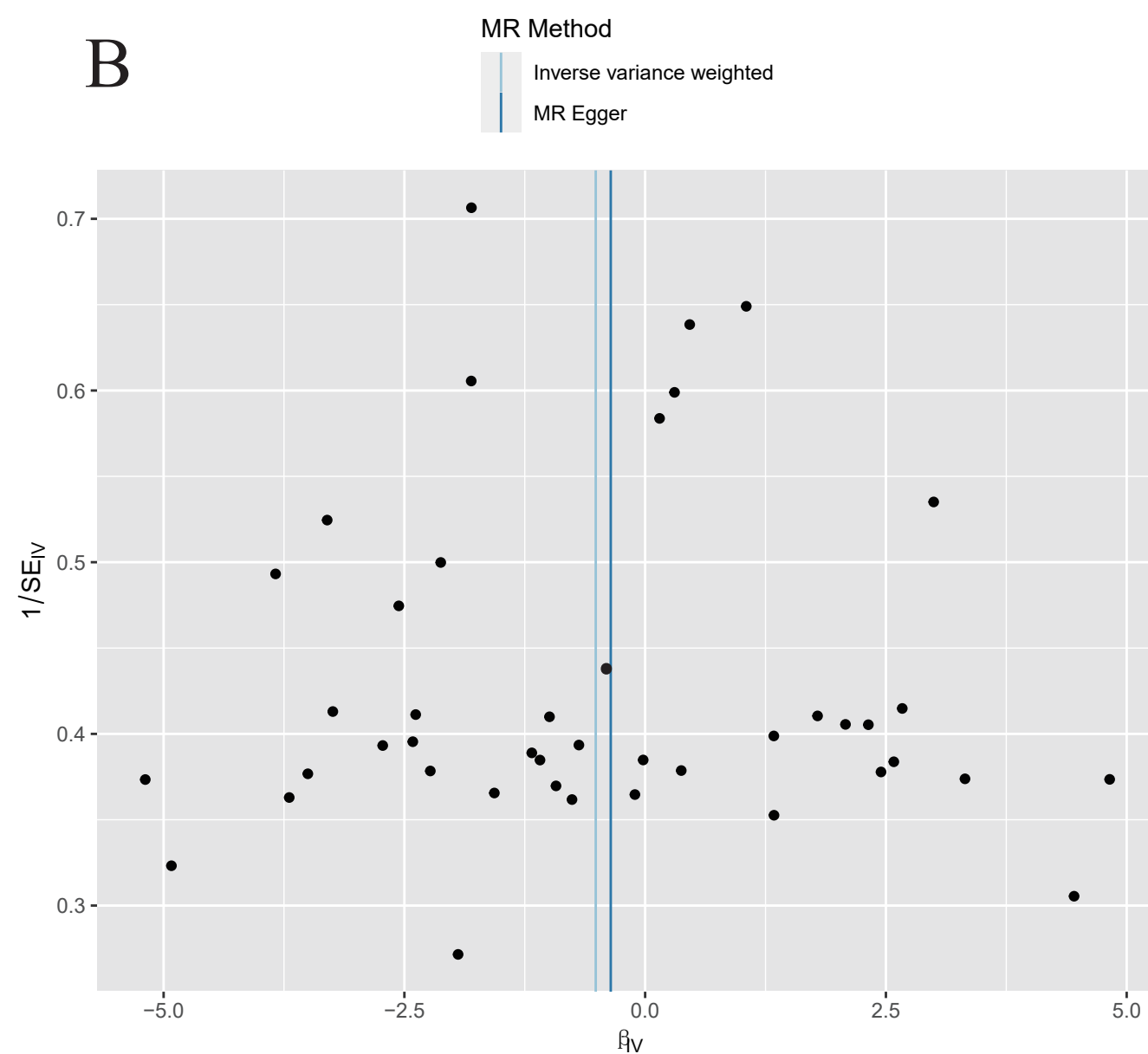

C

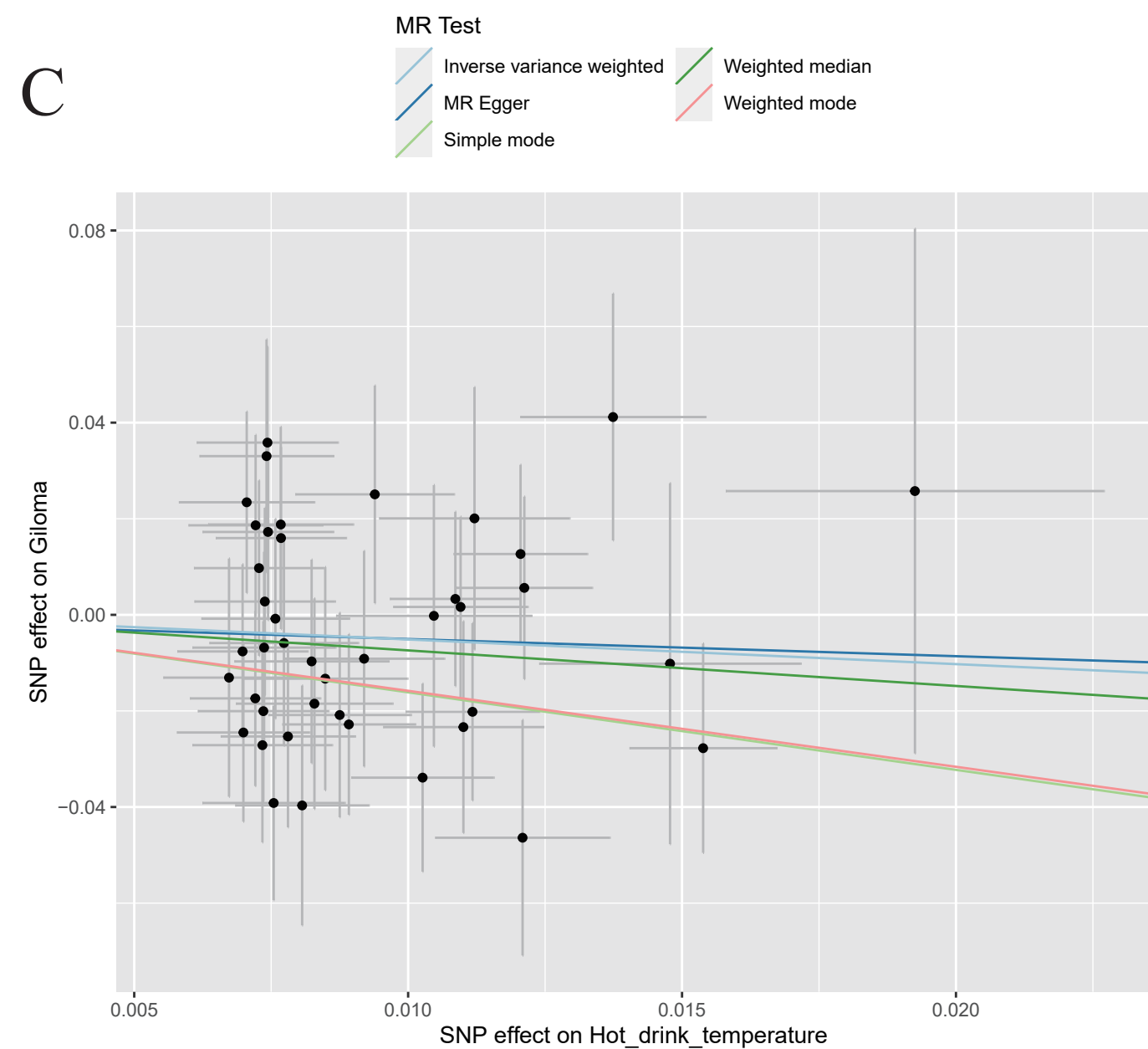

D

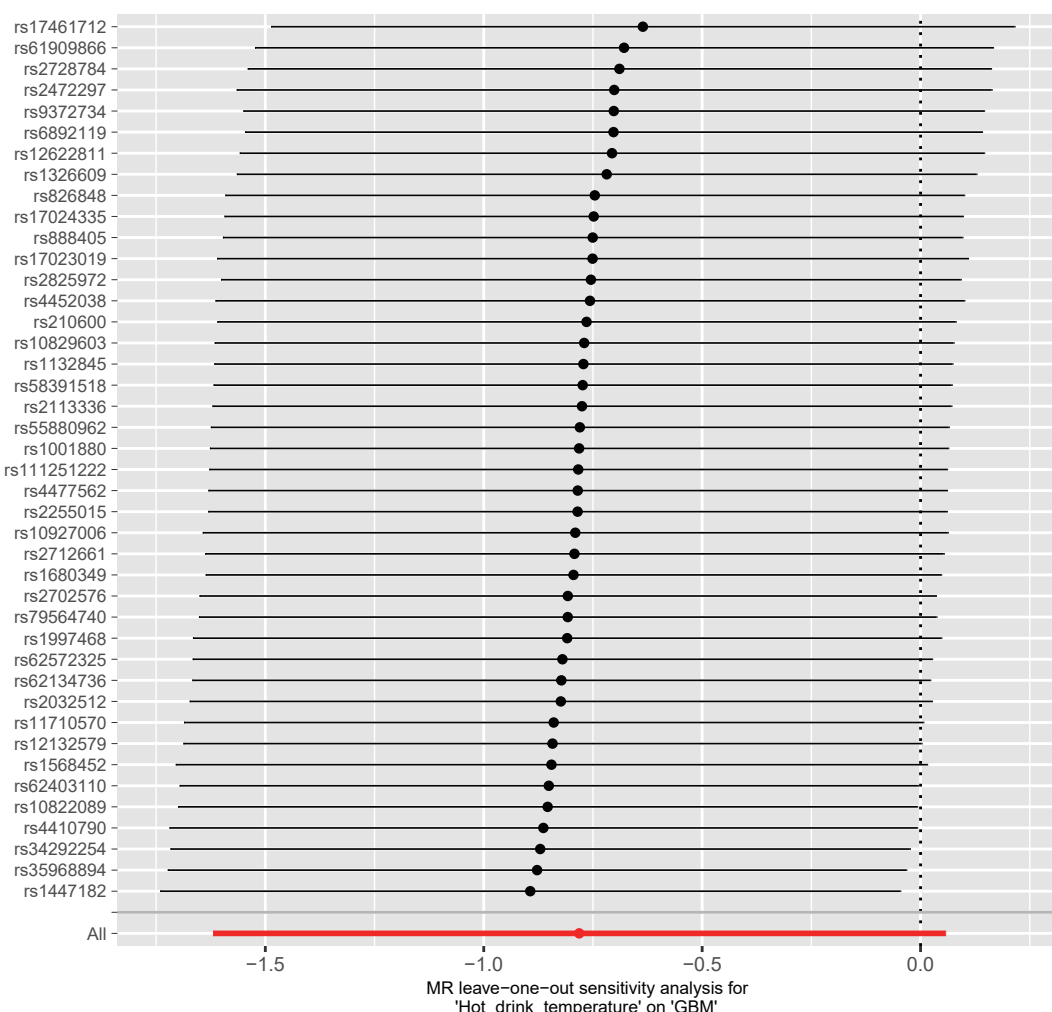

E

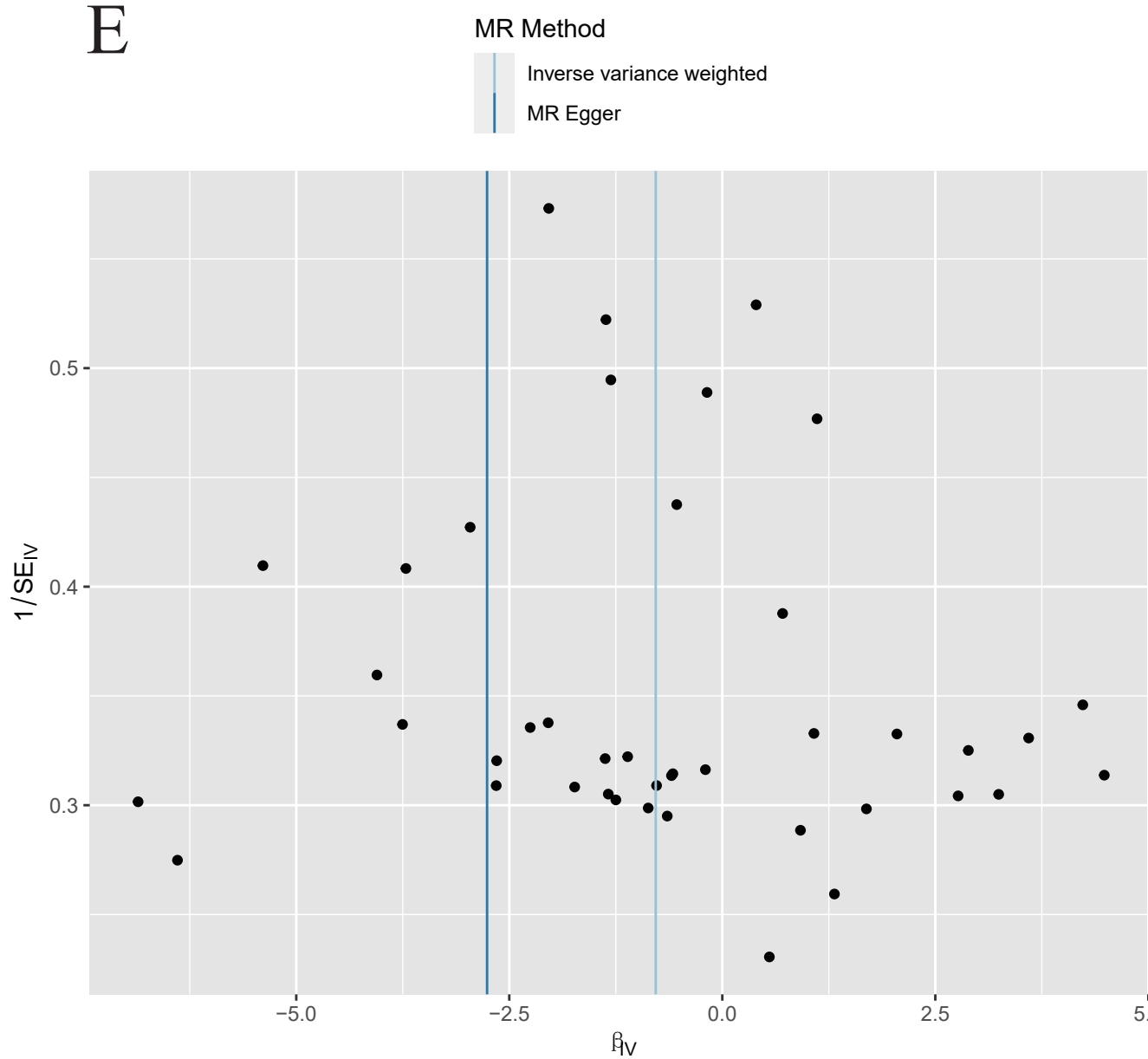

F

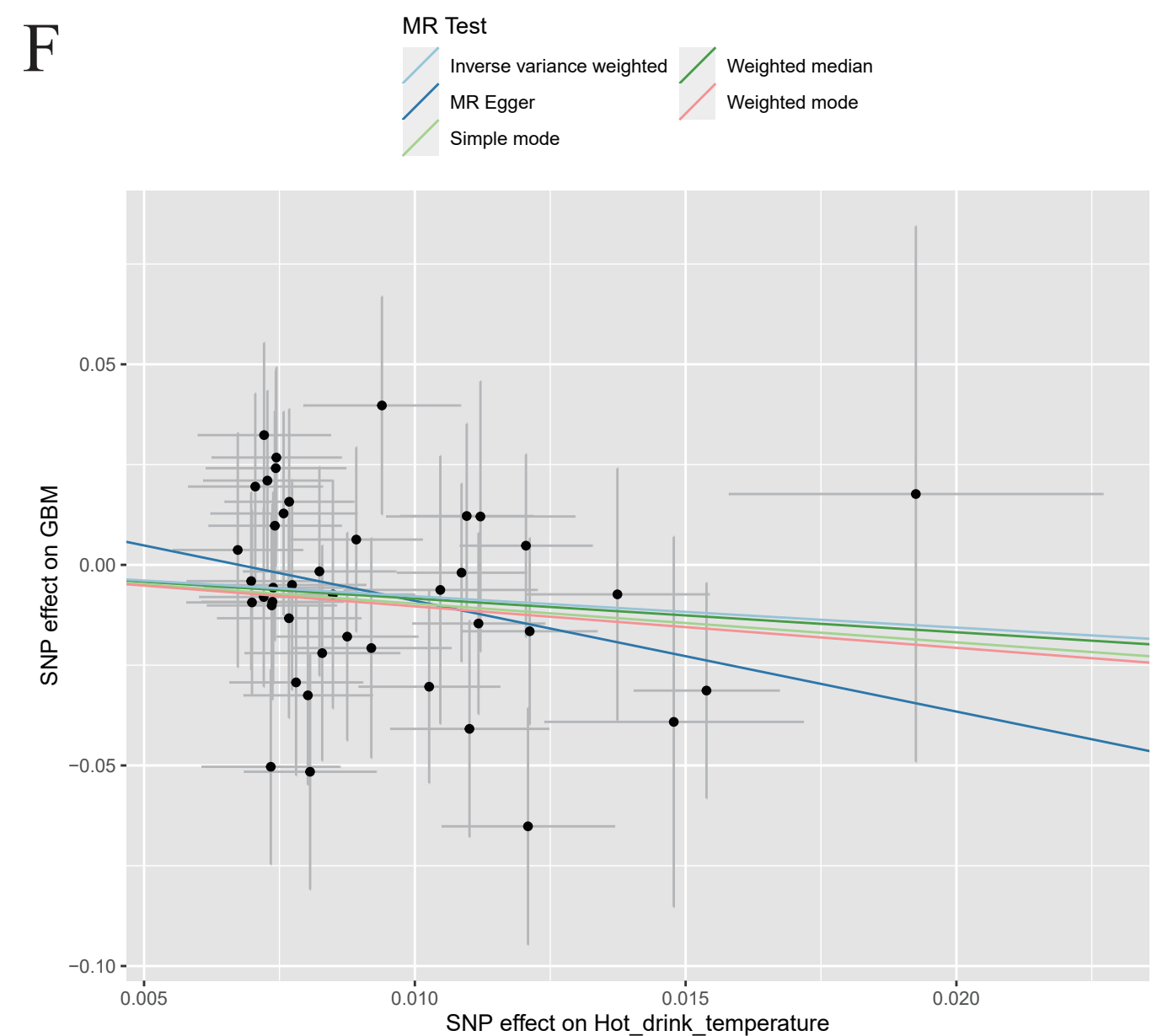

G

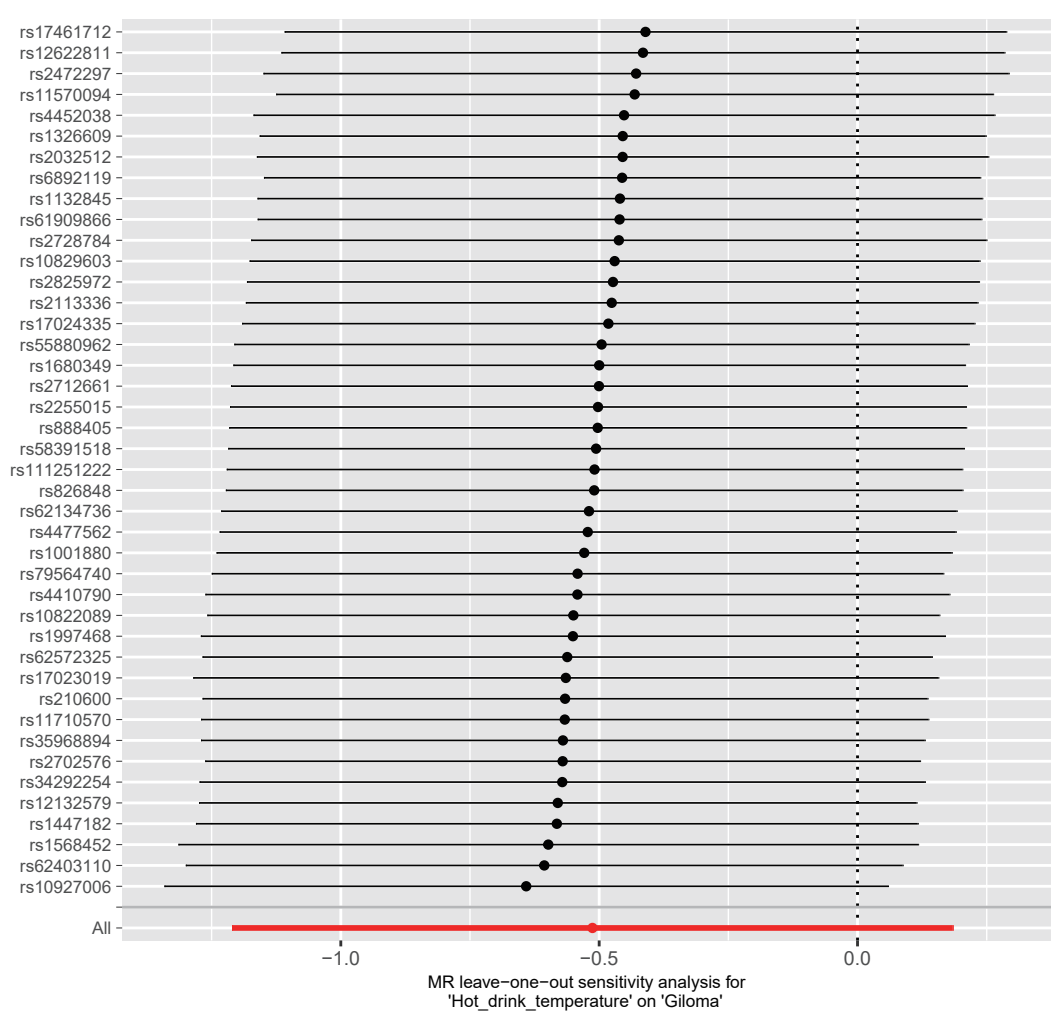

H

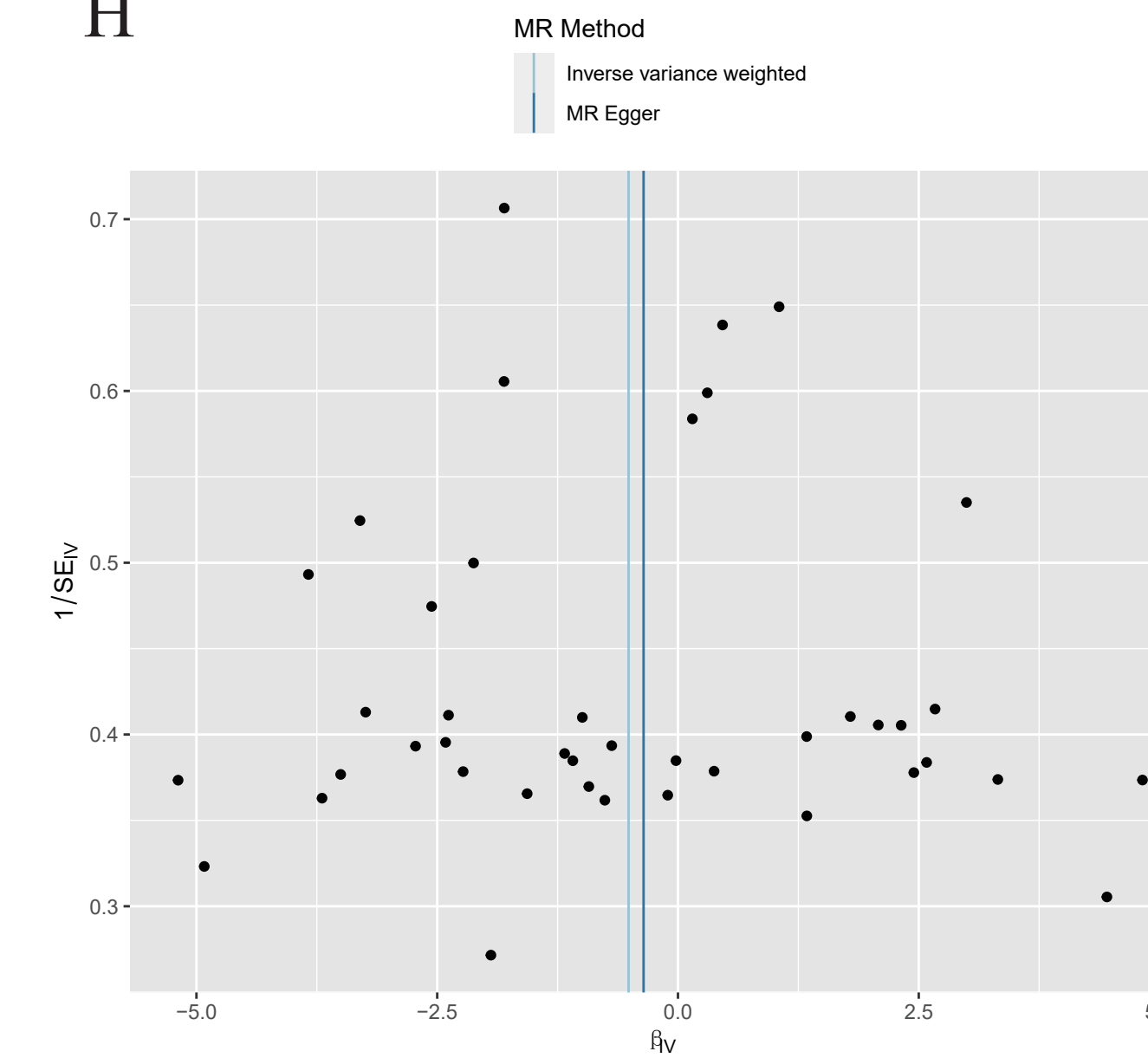

I

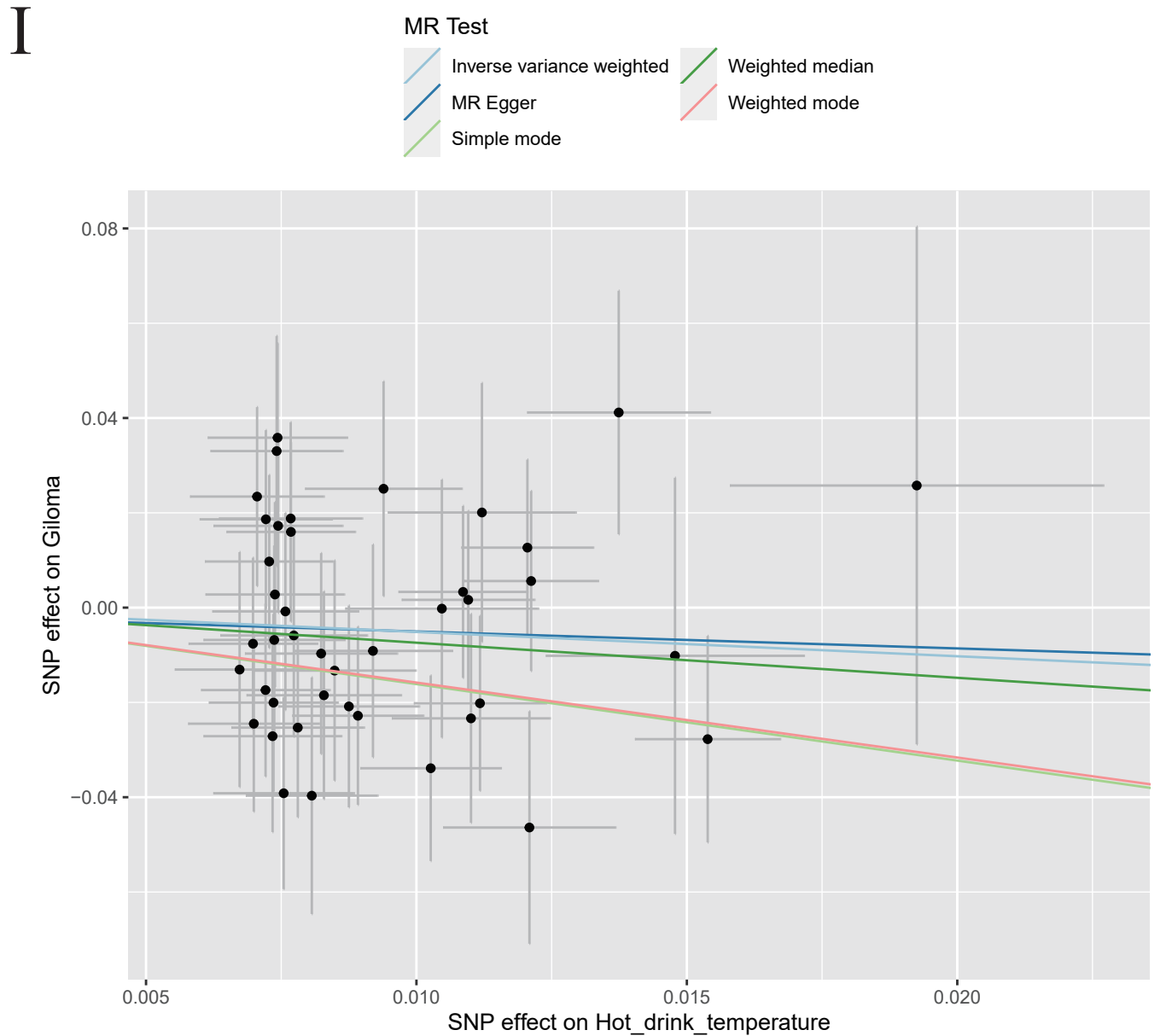

Supplement: Supplementary file 1 [file nutrients-17-00582-s001.zip › nutrients-3462880-supplementary/Sup_3.pdf]

A

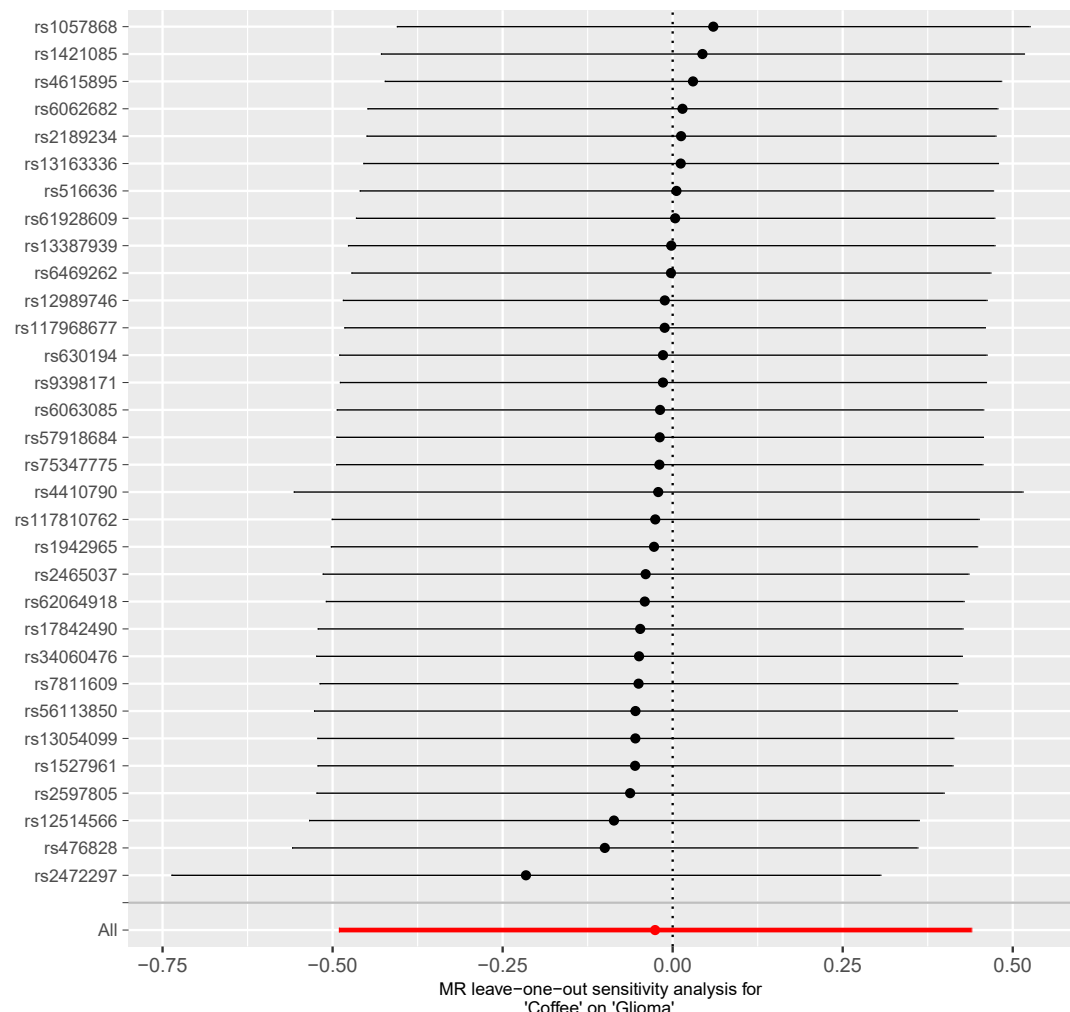

B

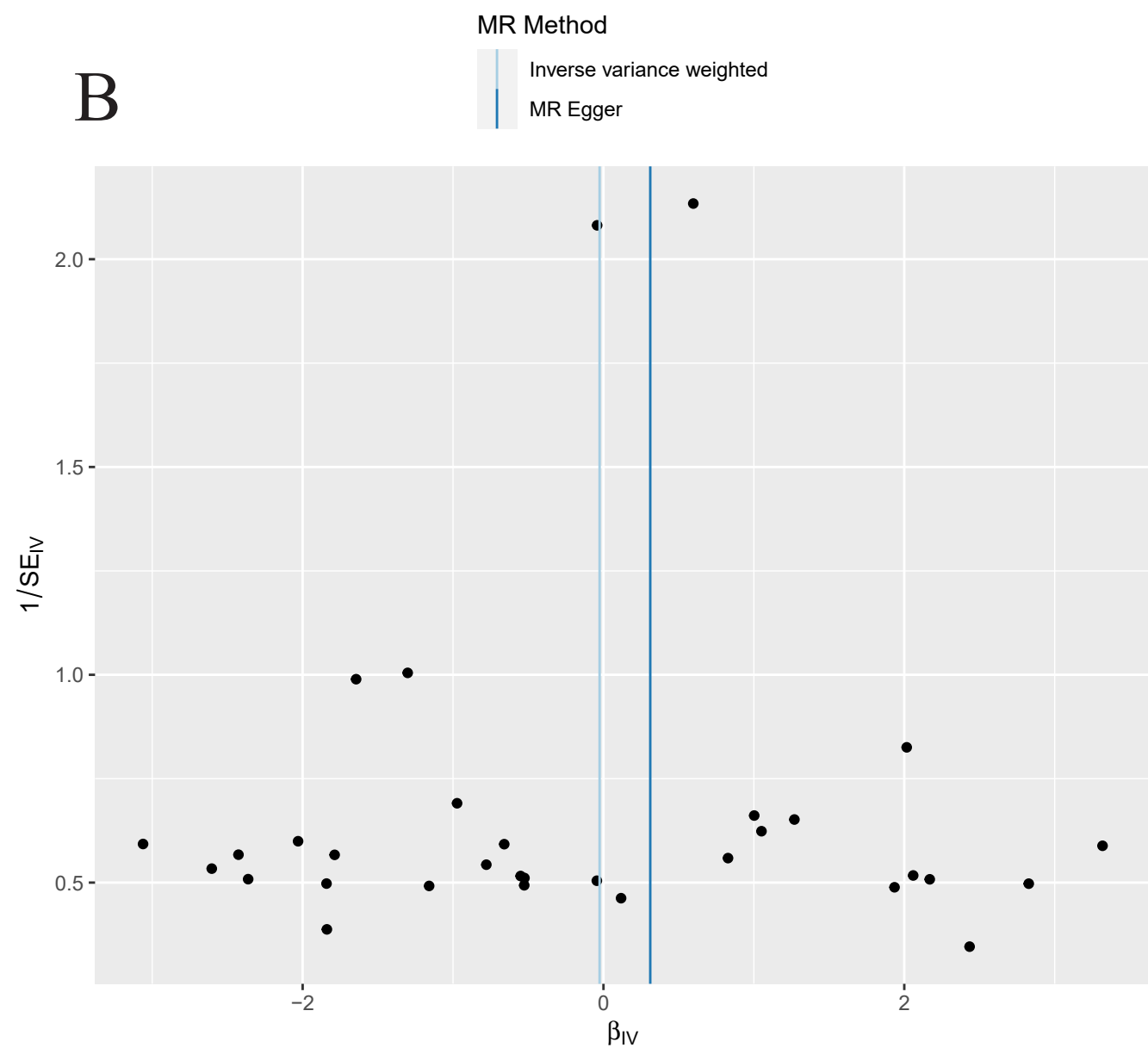

C

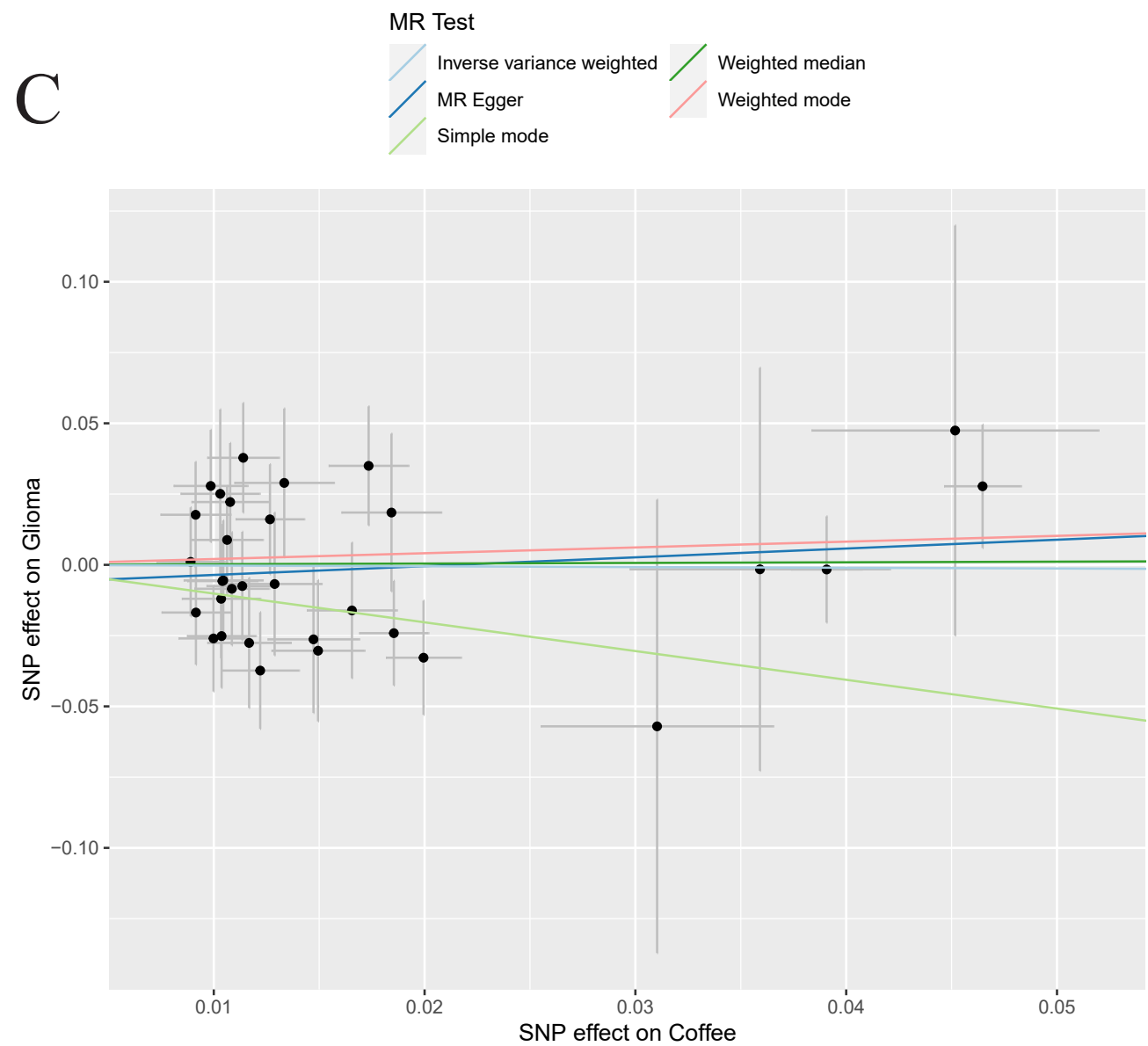

D

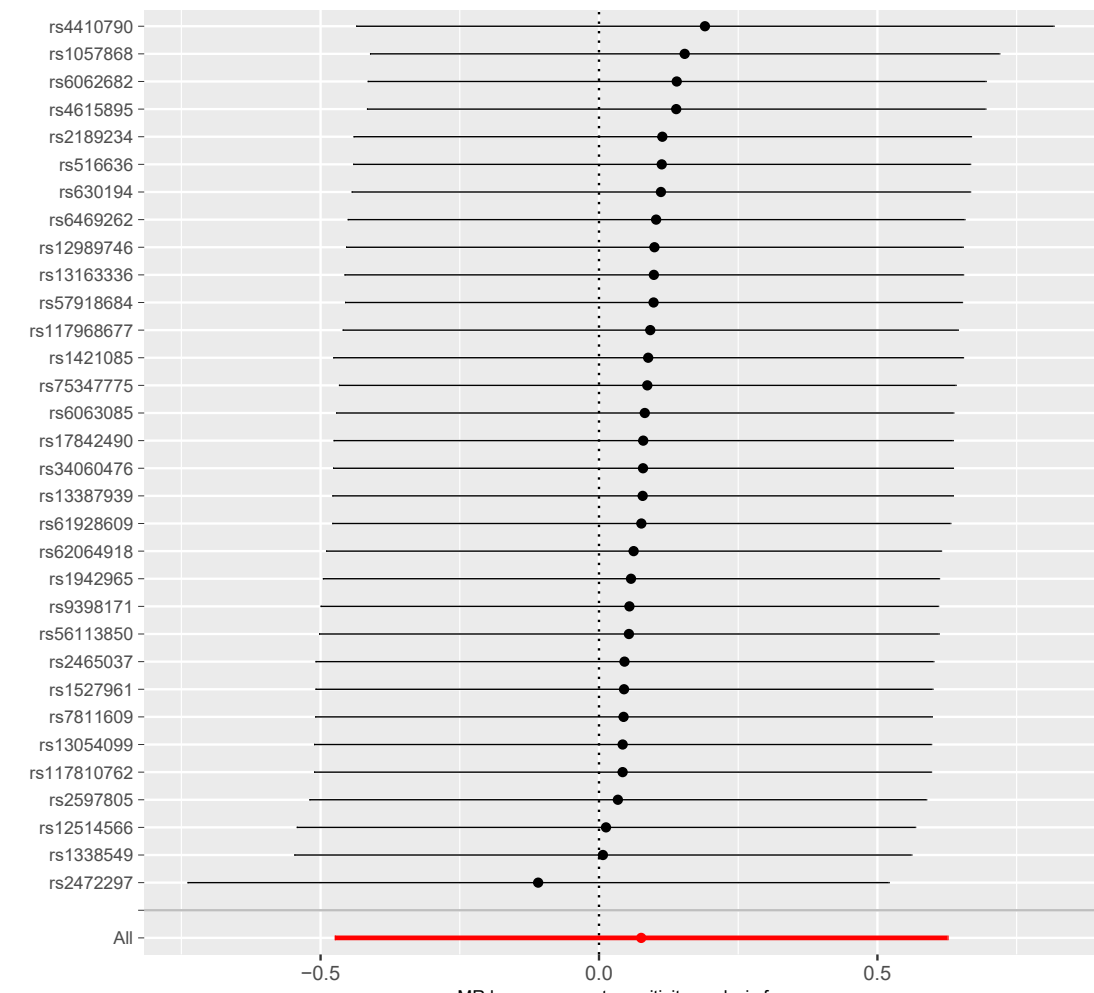

E

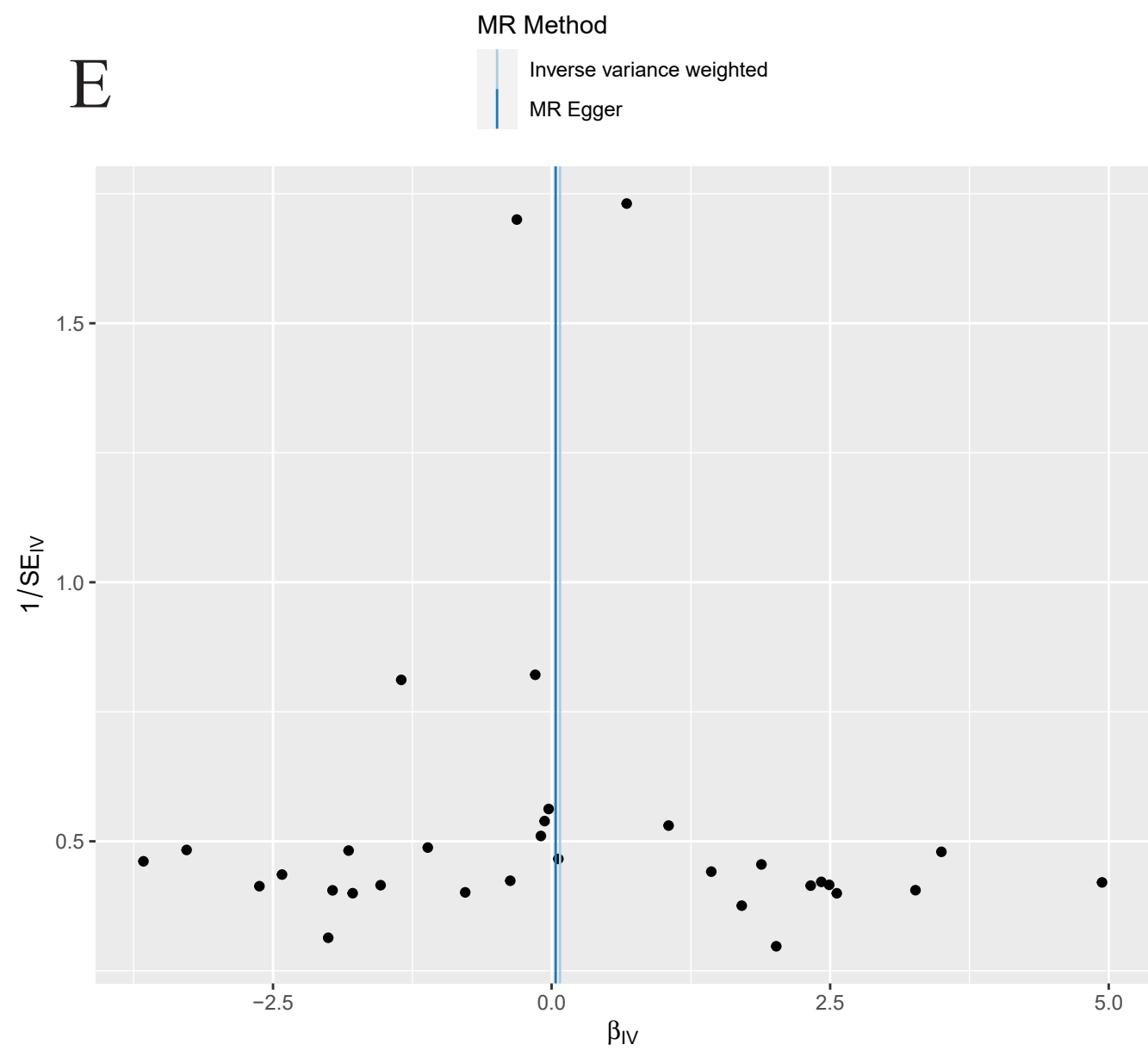

F

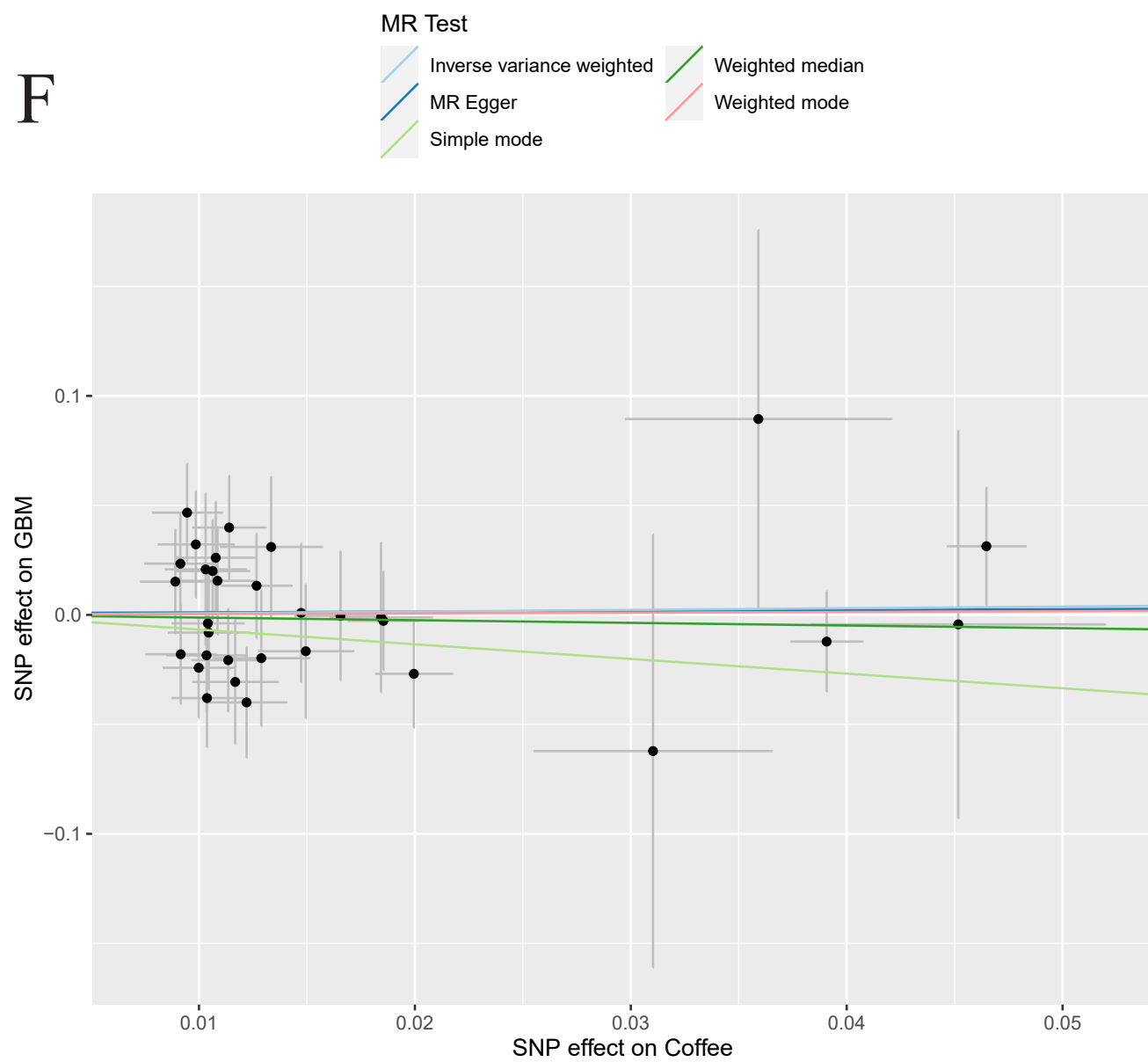

G

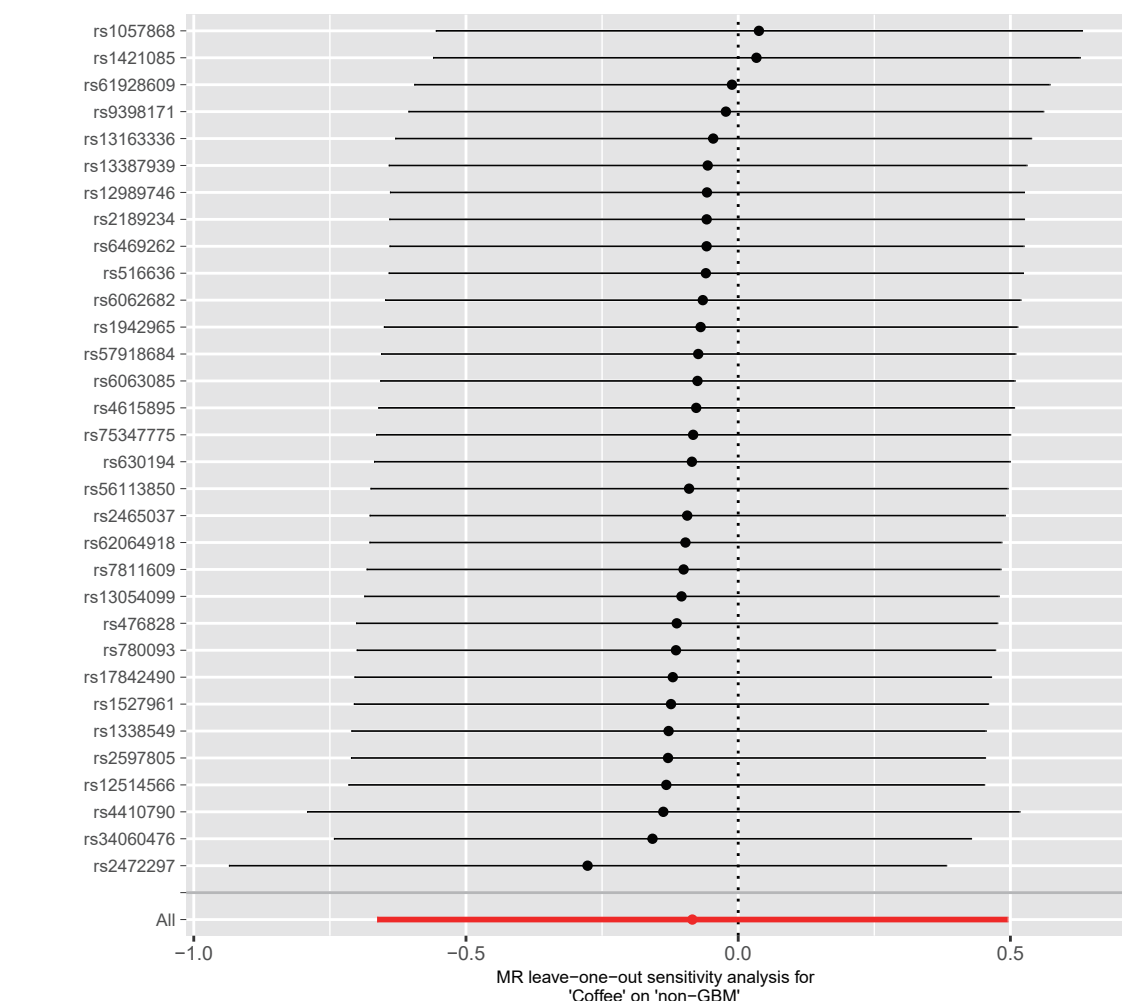

H

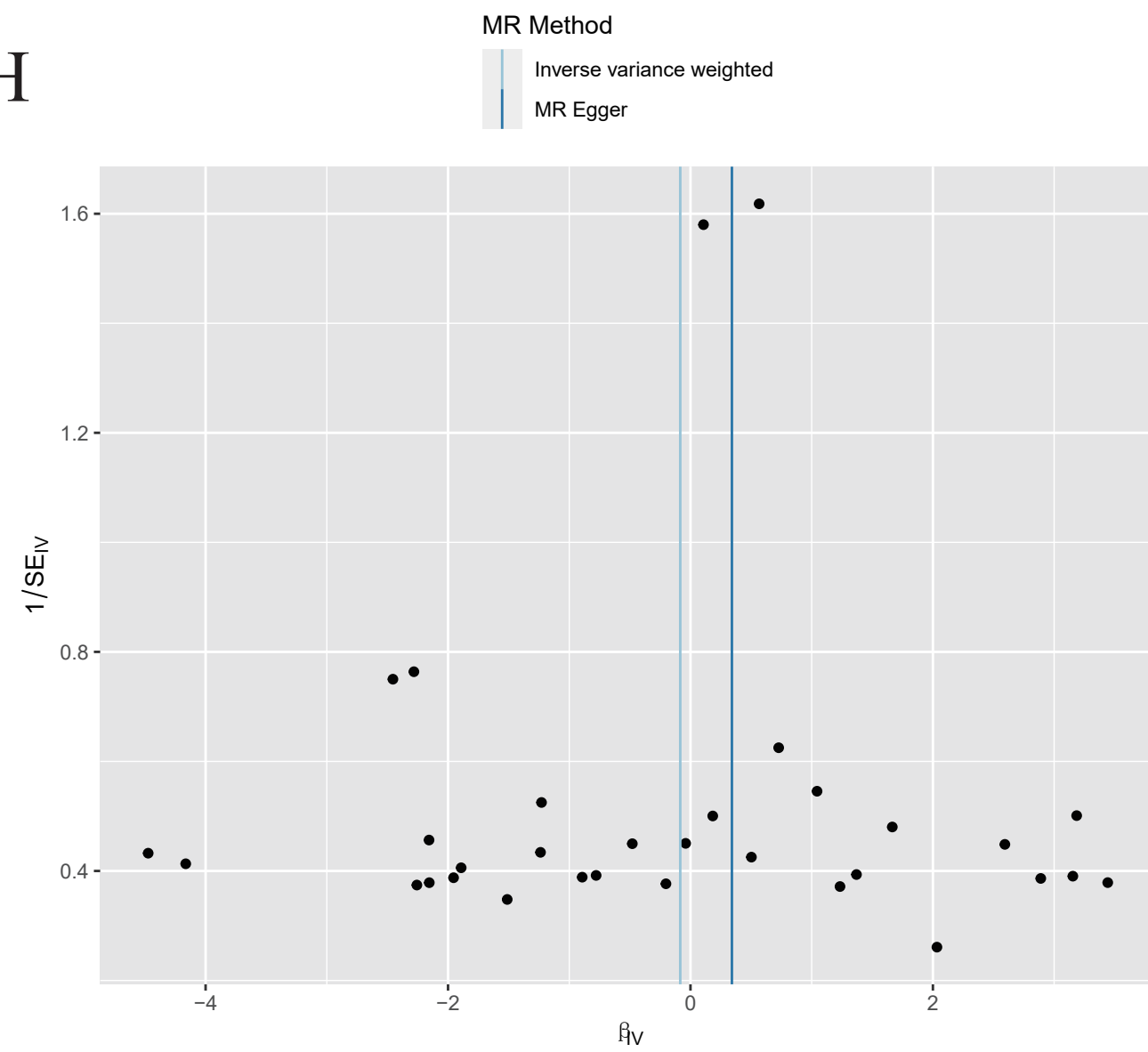

I

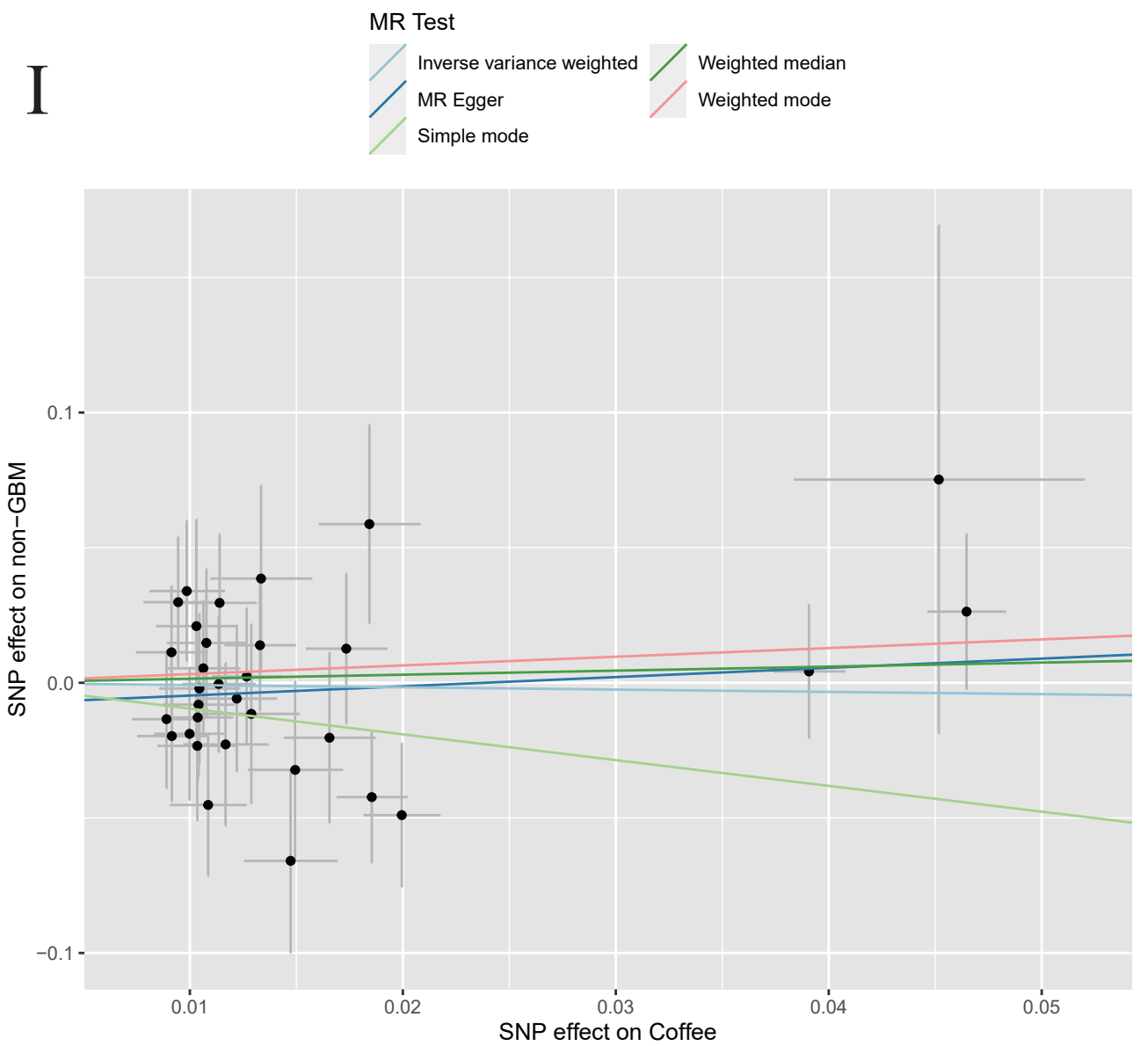

Supplement: Supplementary file 1 [file nutrients-17-00582-s001.zip › nutrients-3462880-supplementary/Sup_4.pdf]

A

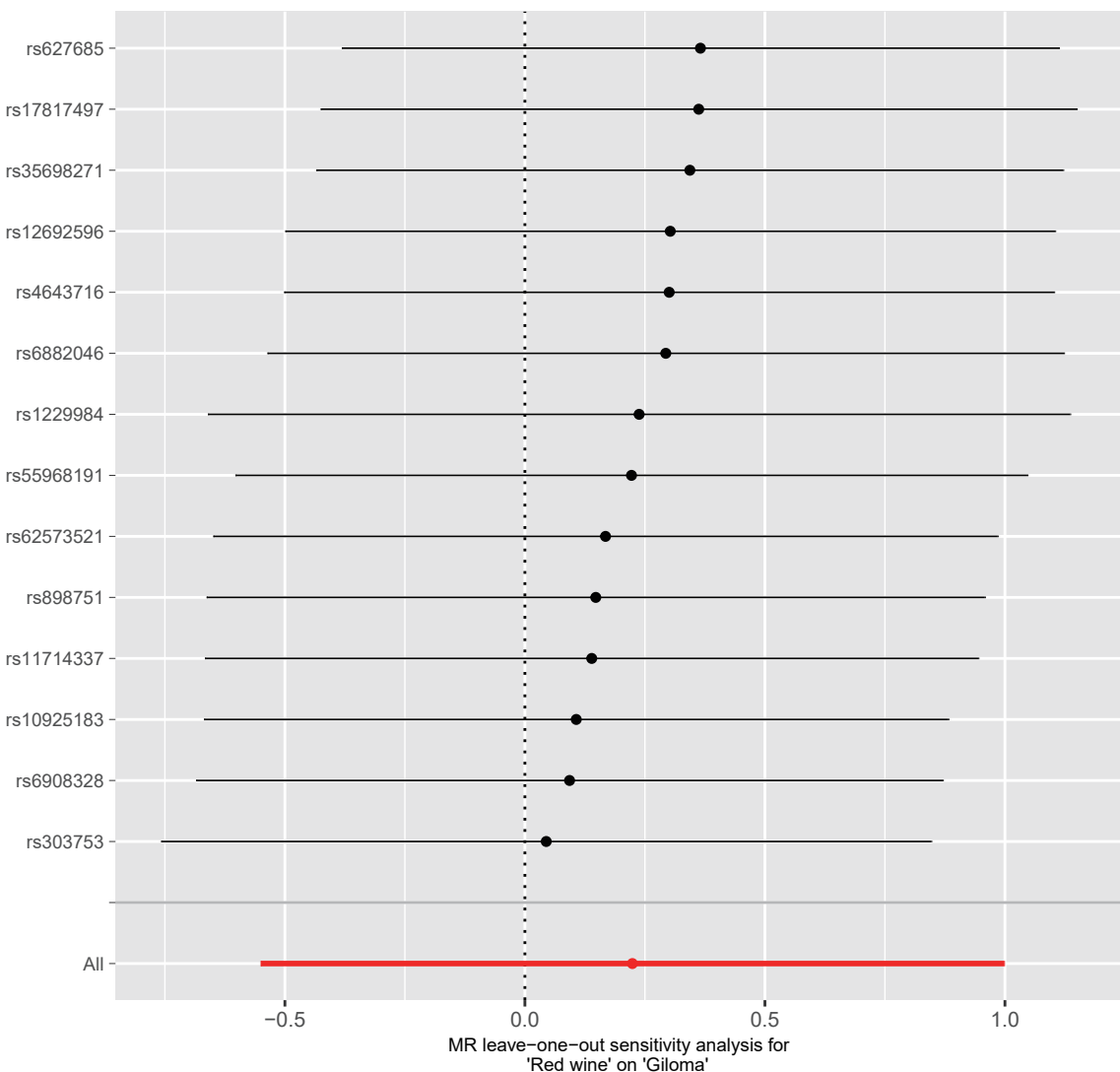

B

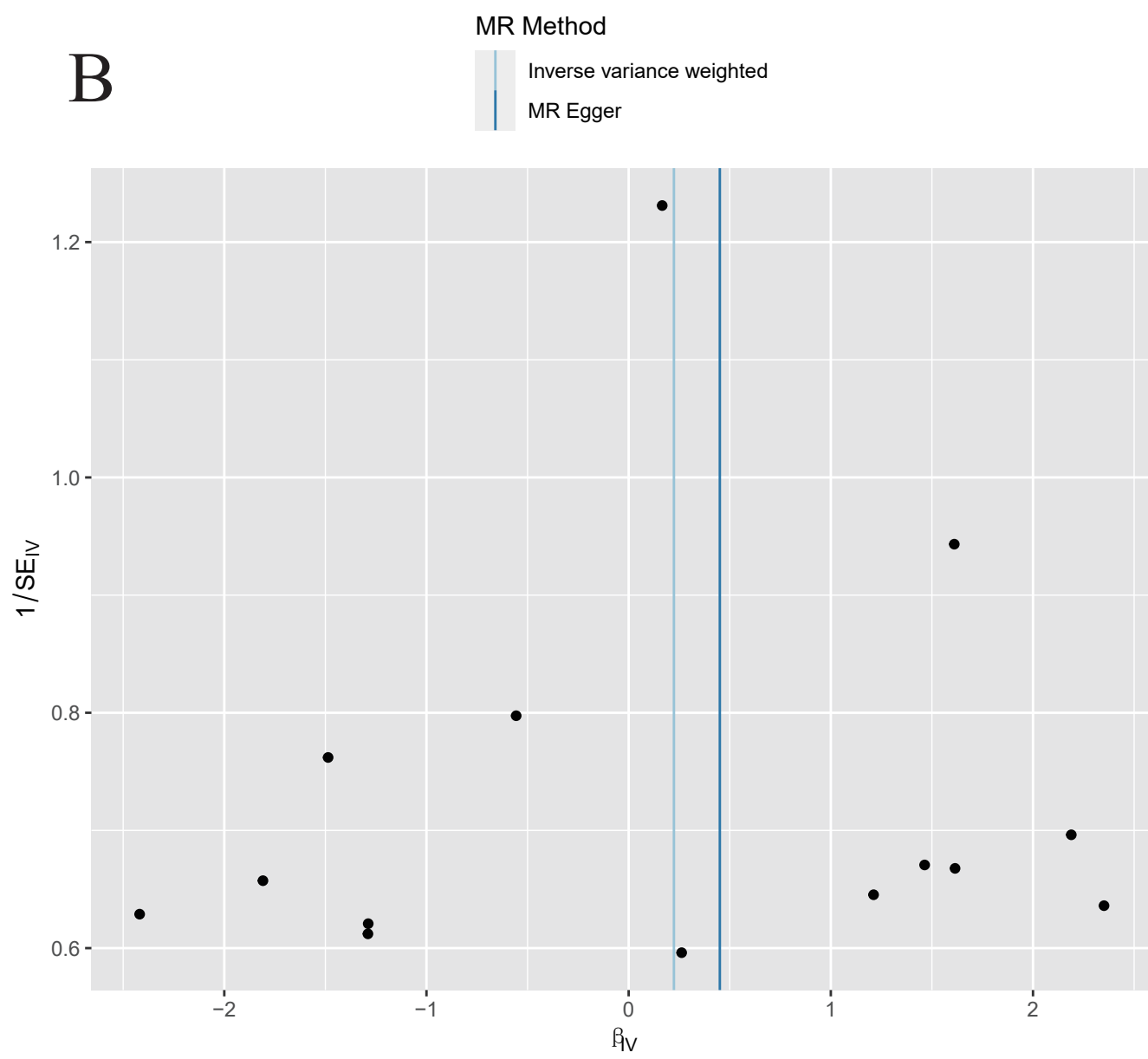

C

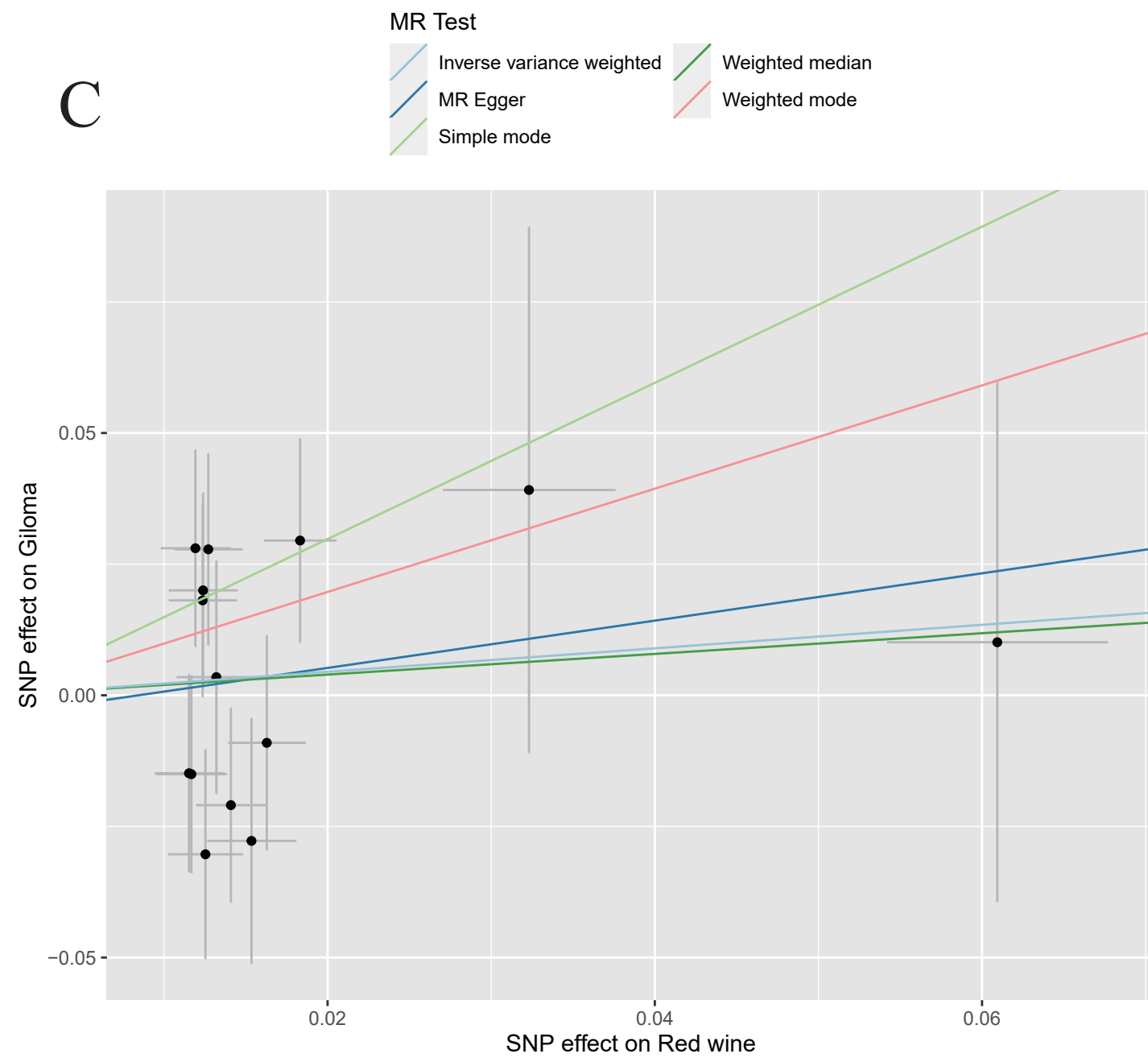

D

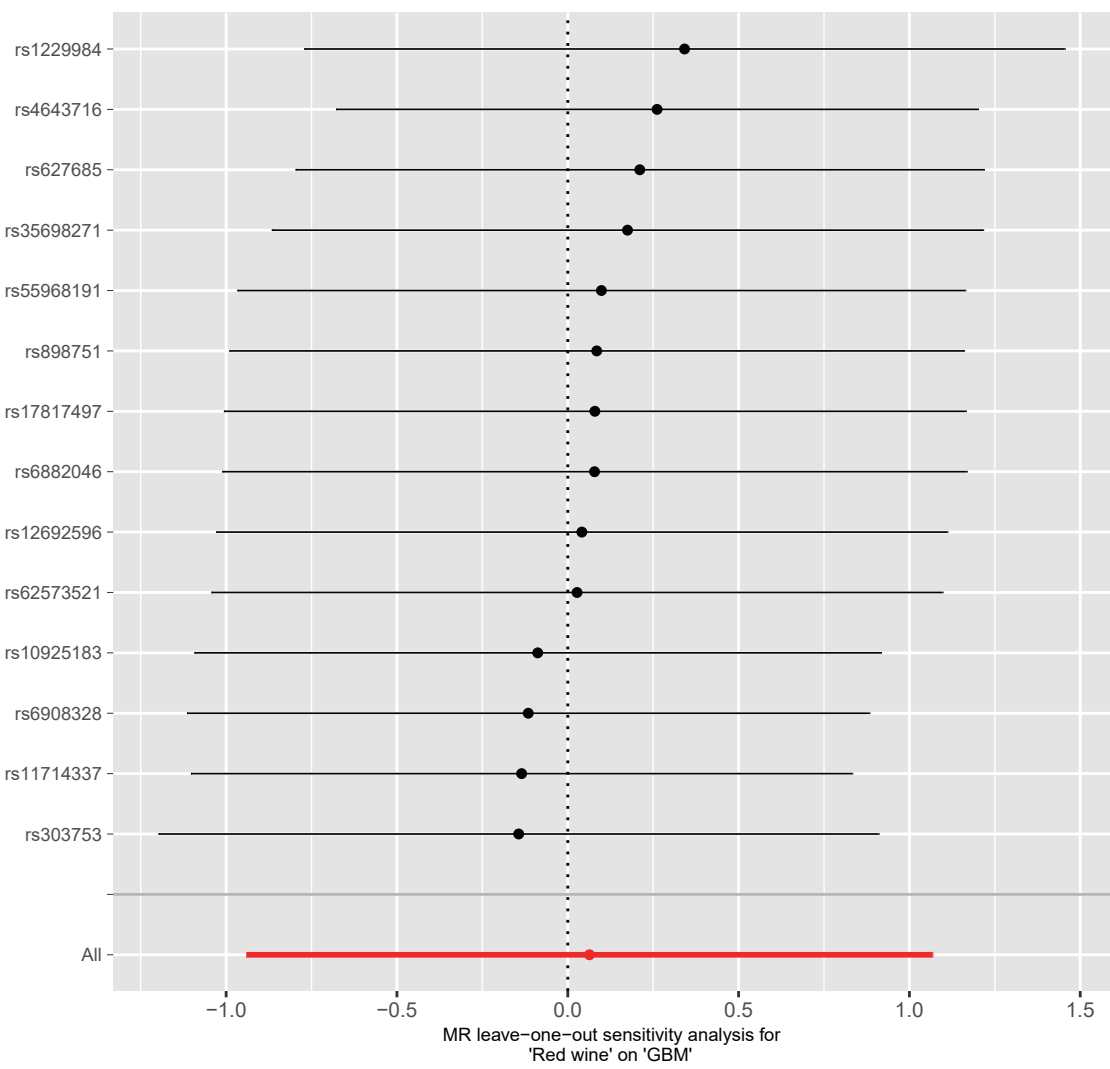

E

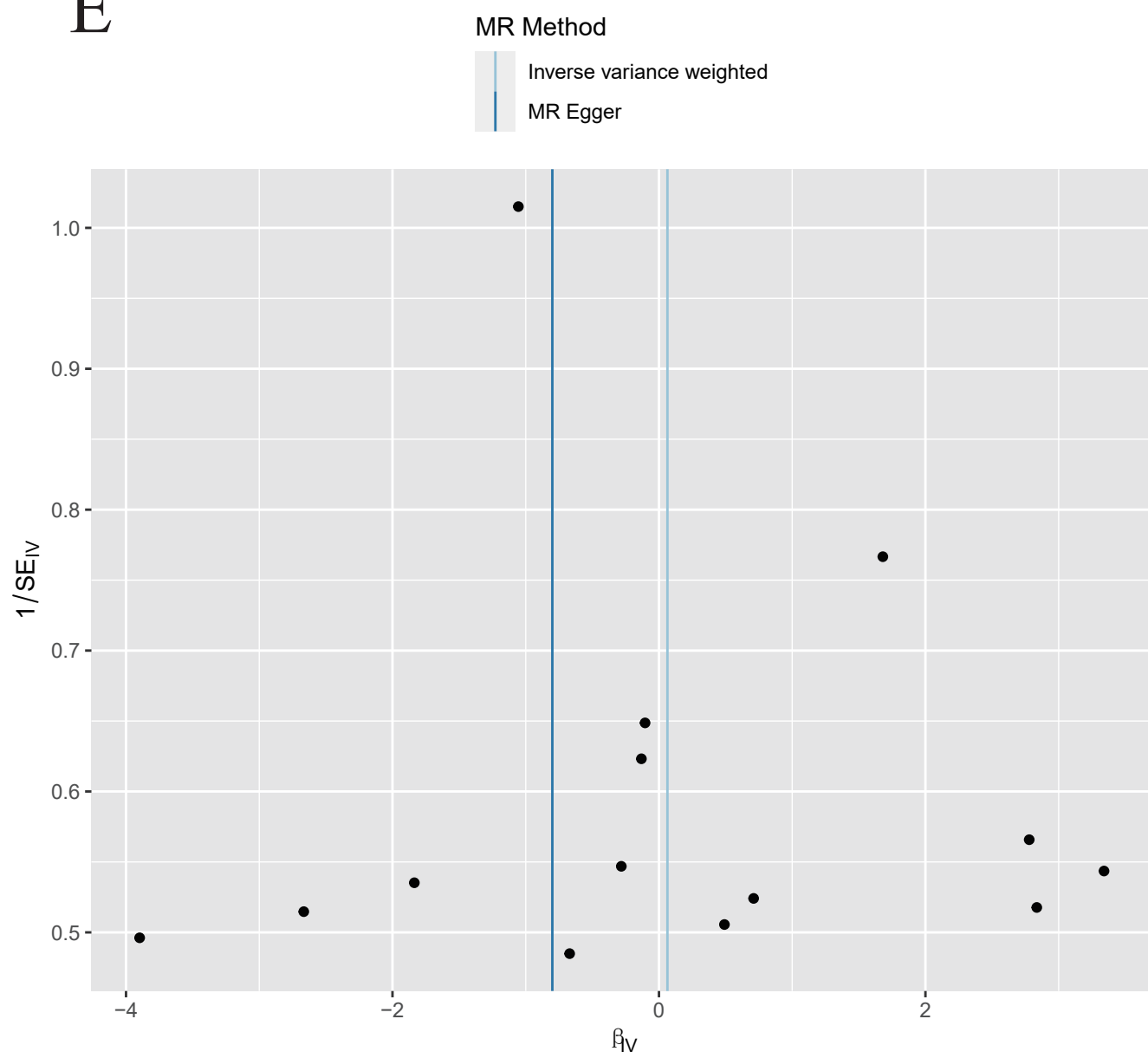

F

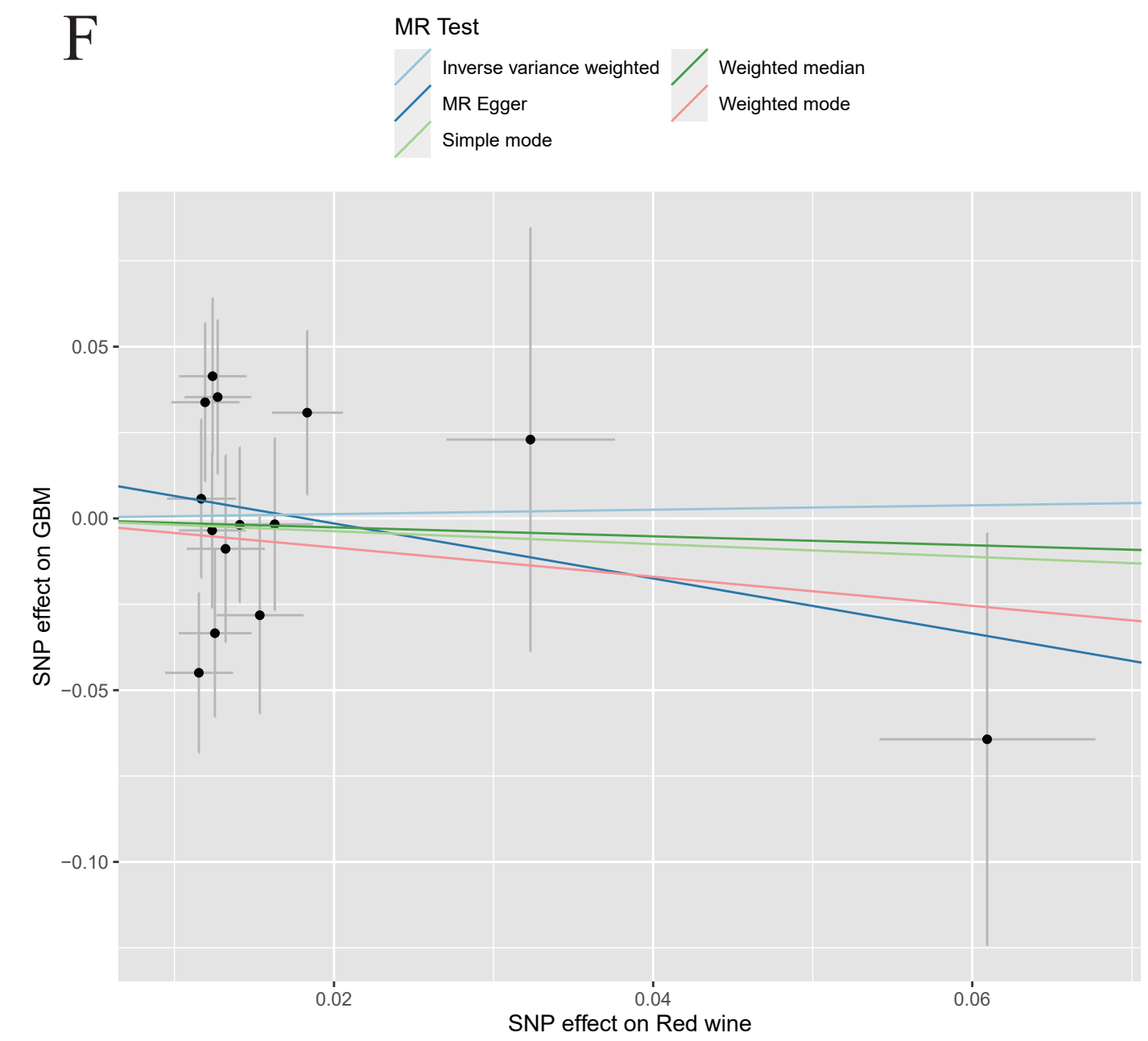

G

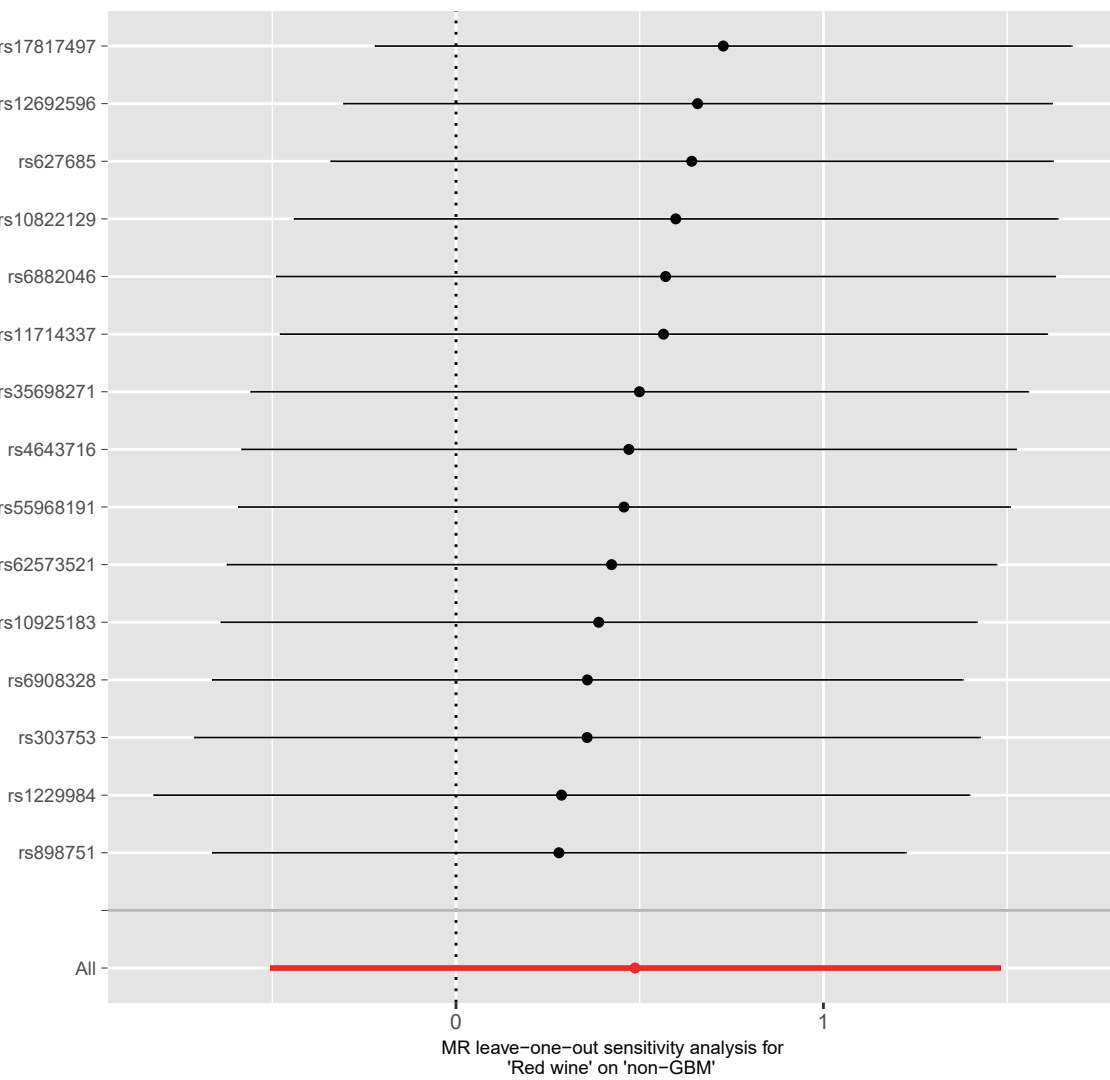

H

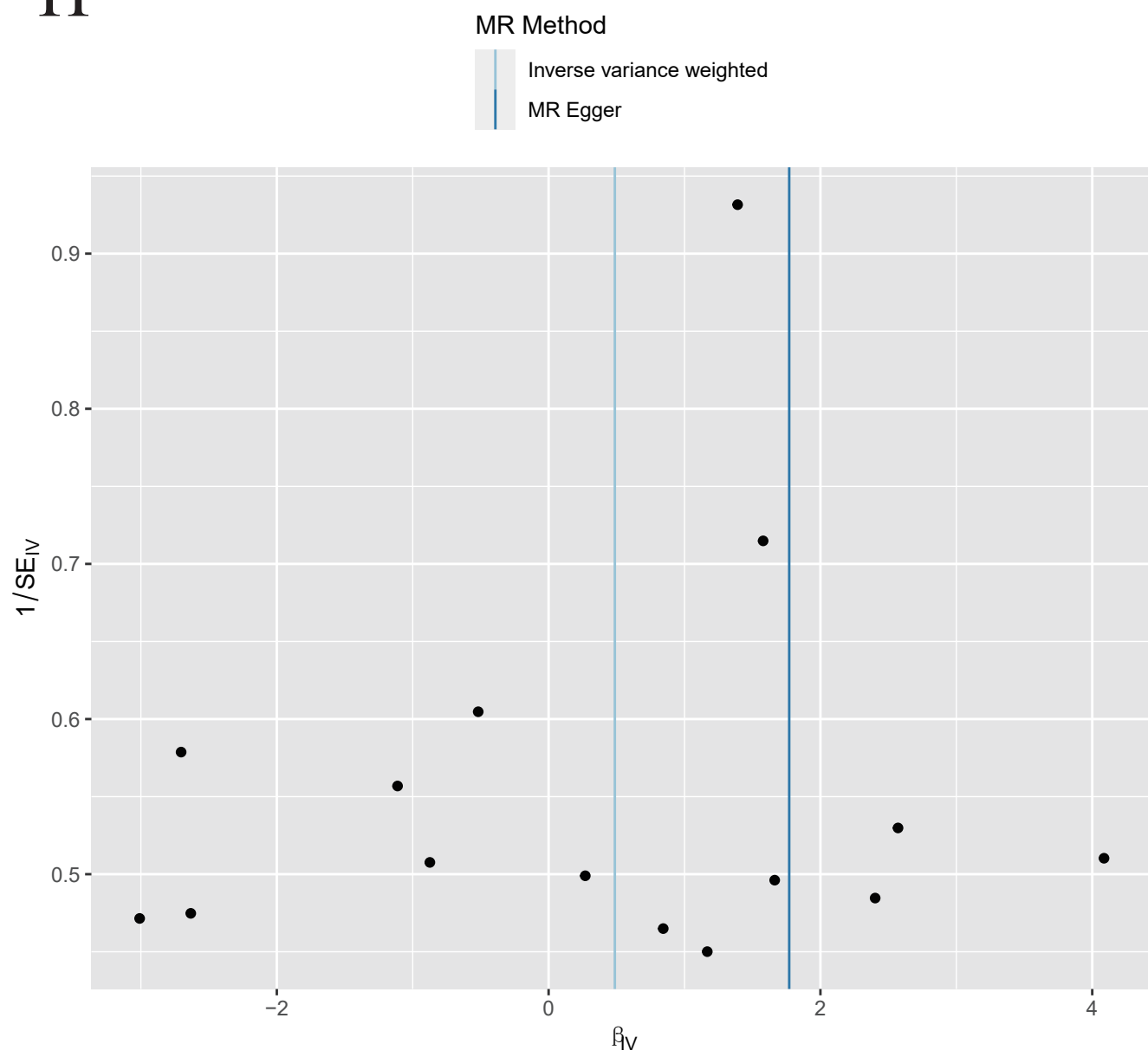

I

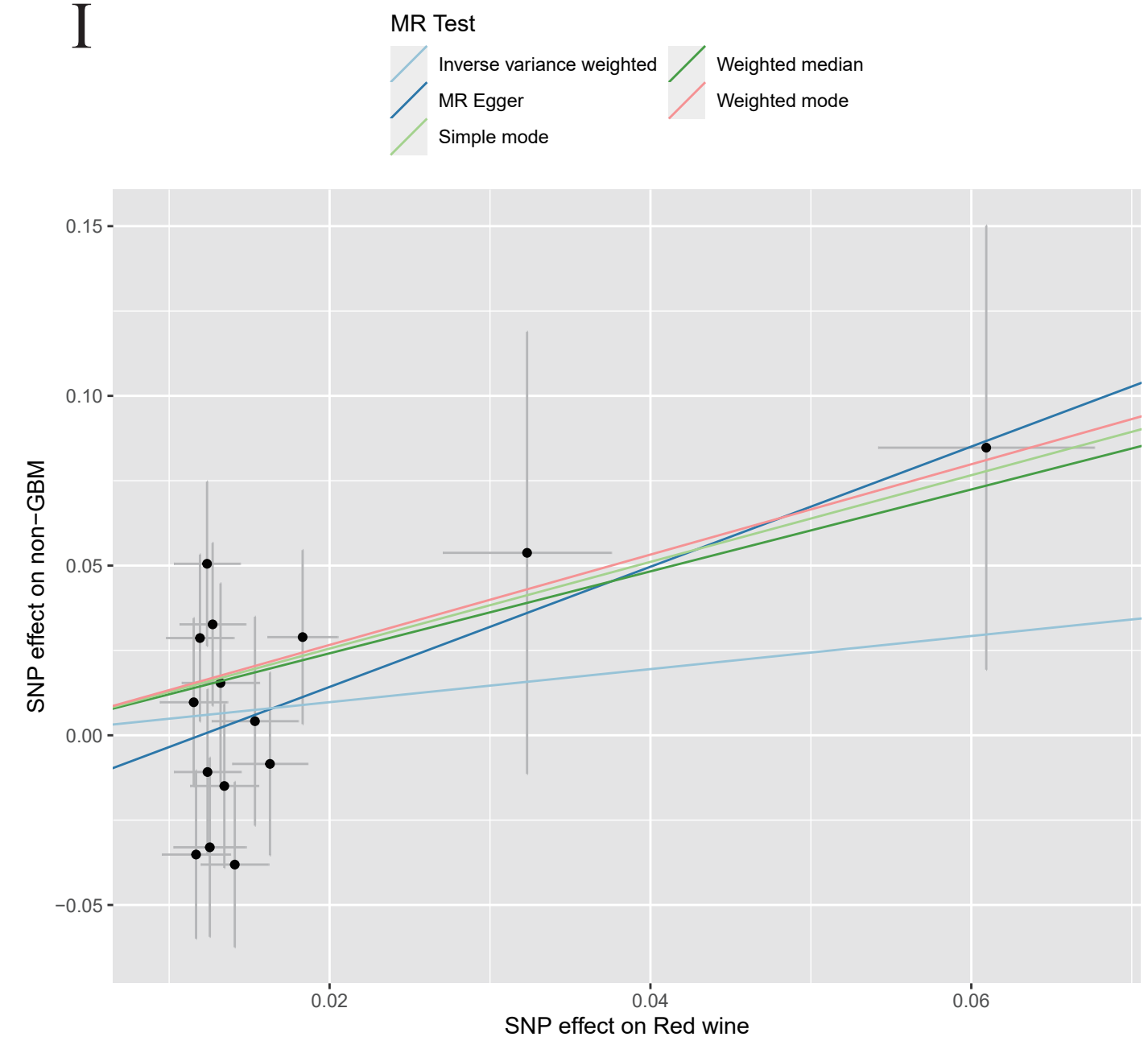

Supplement: Supplementary file 1 [file nutrients-17-00582-s001.zip › nutrients-3462880-supplementary/Sup_5.pdf]

A

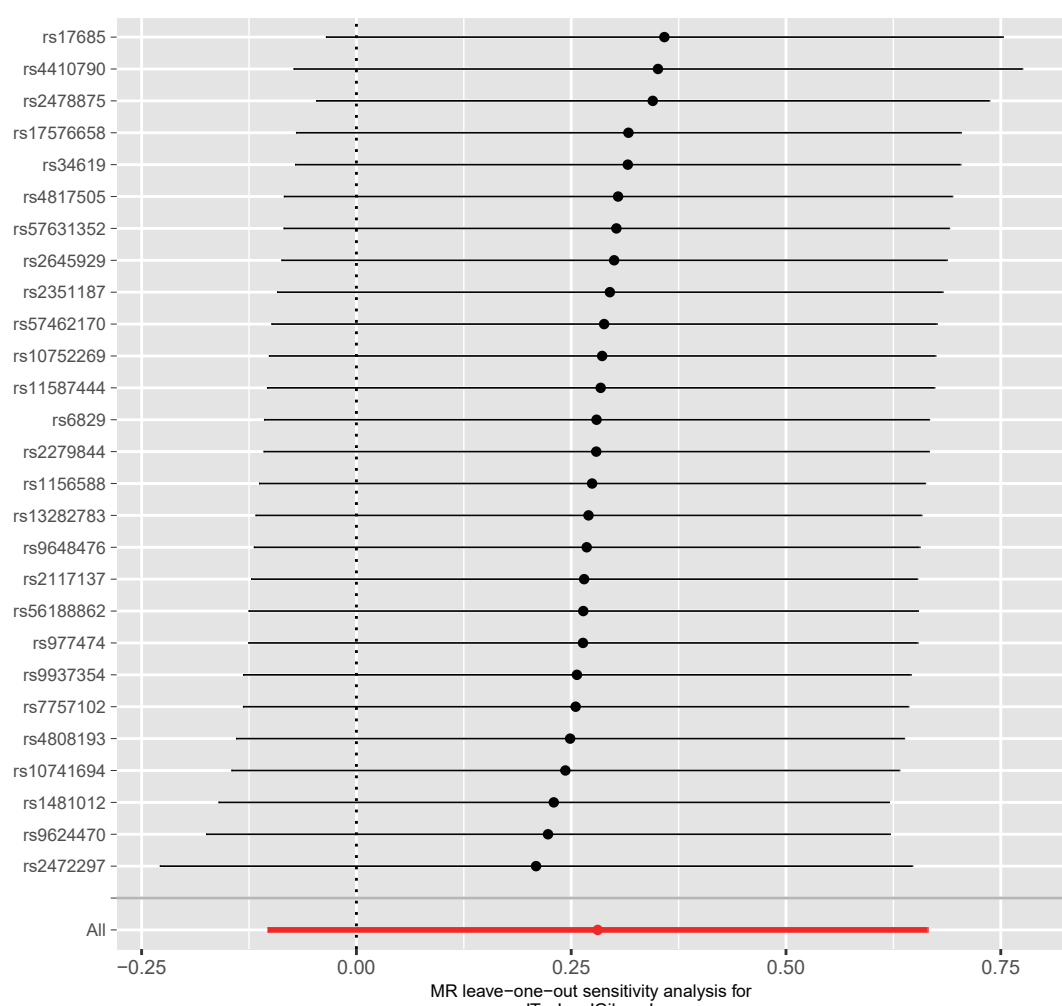

B

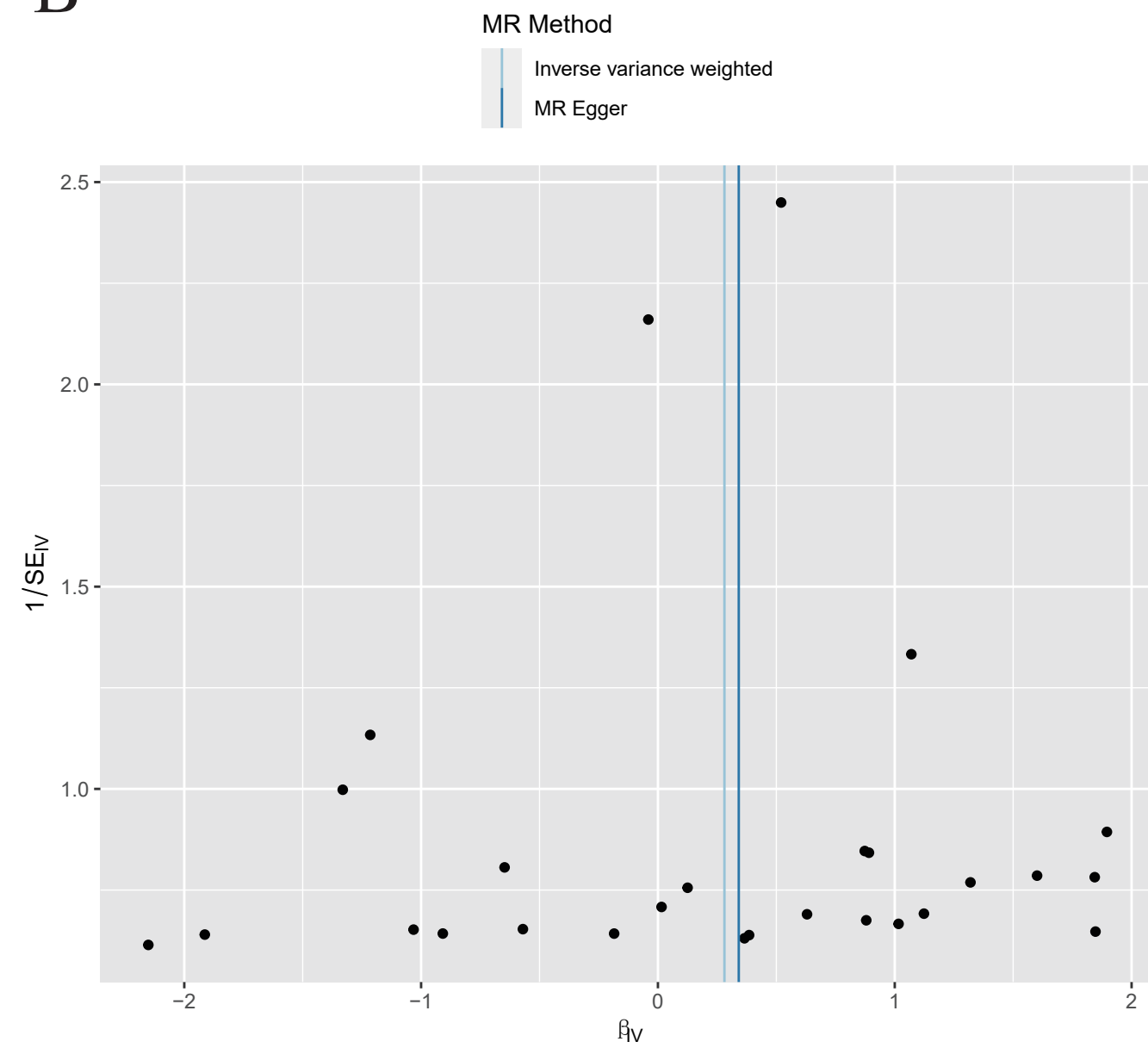

C

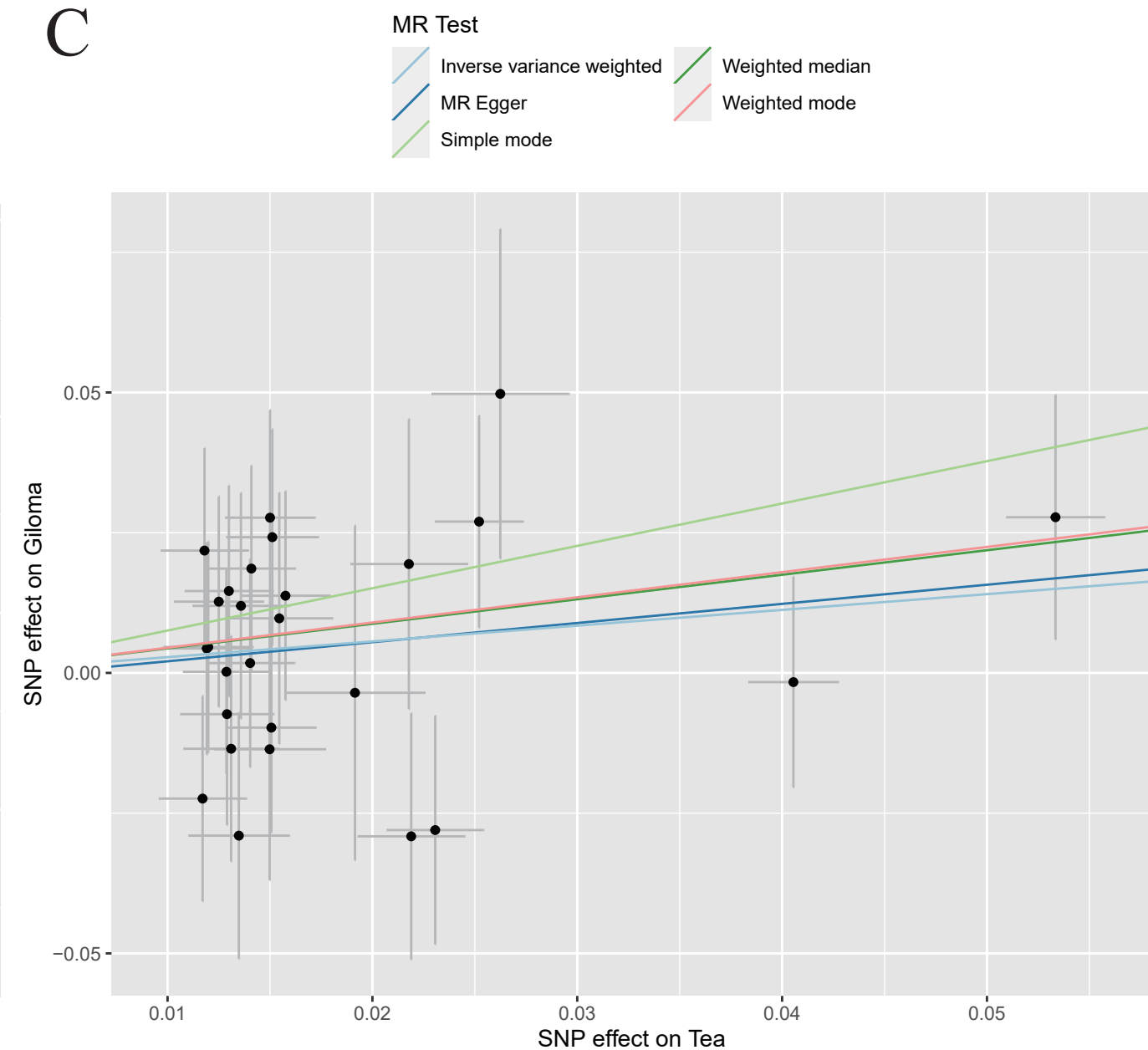

D

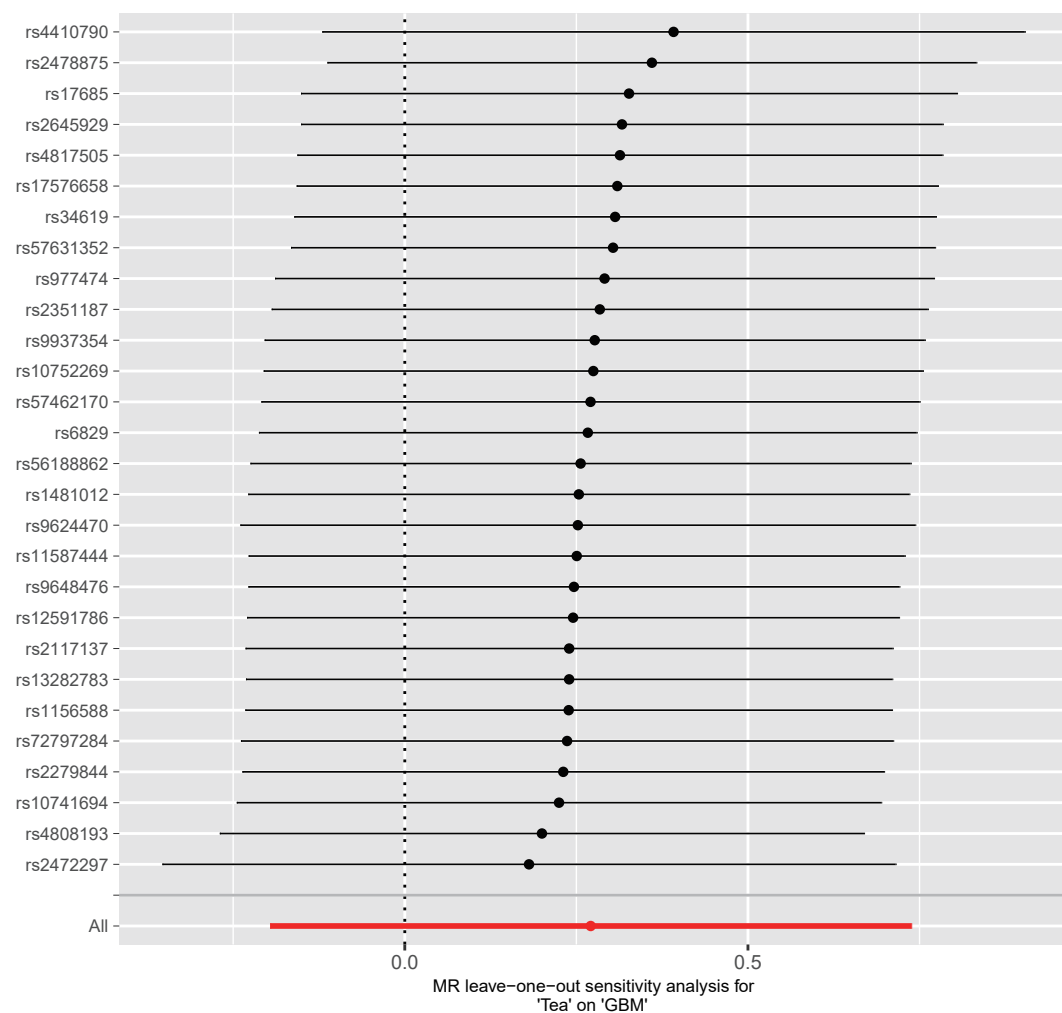

E

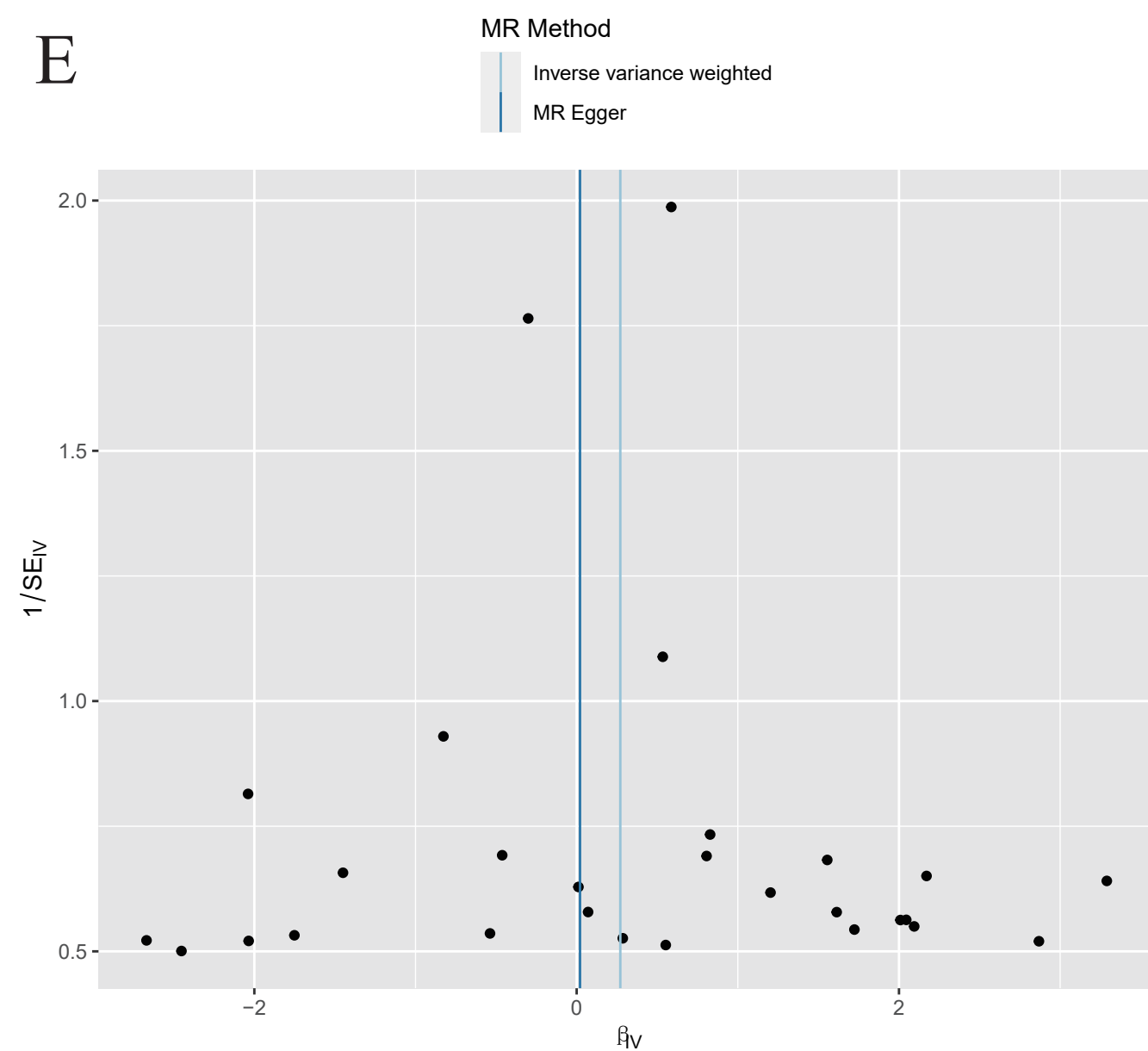

F

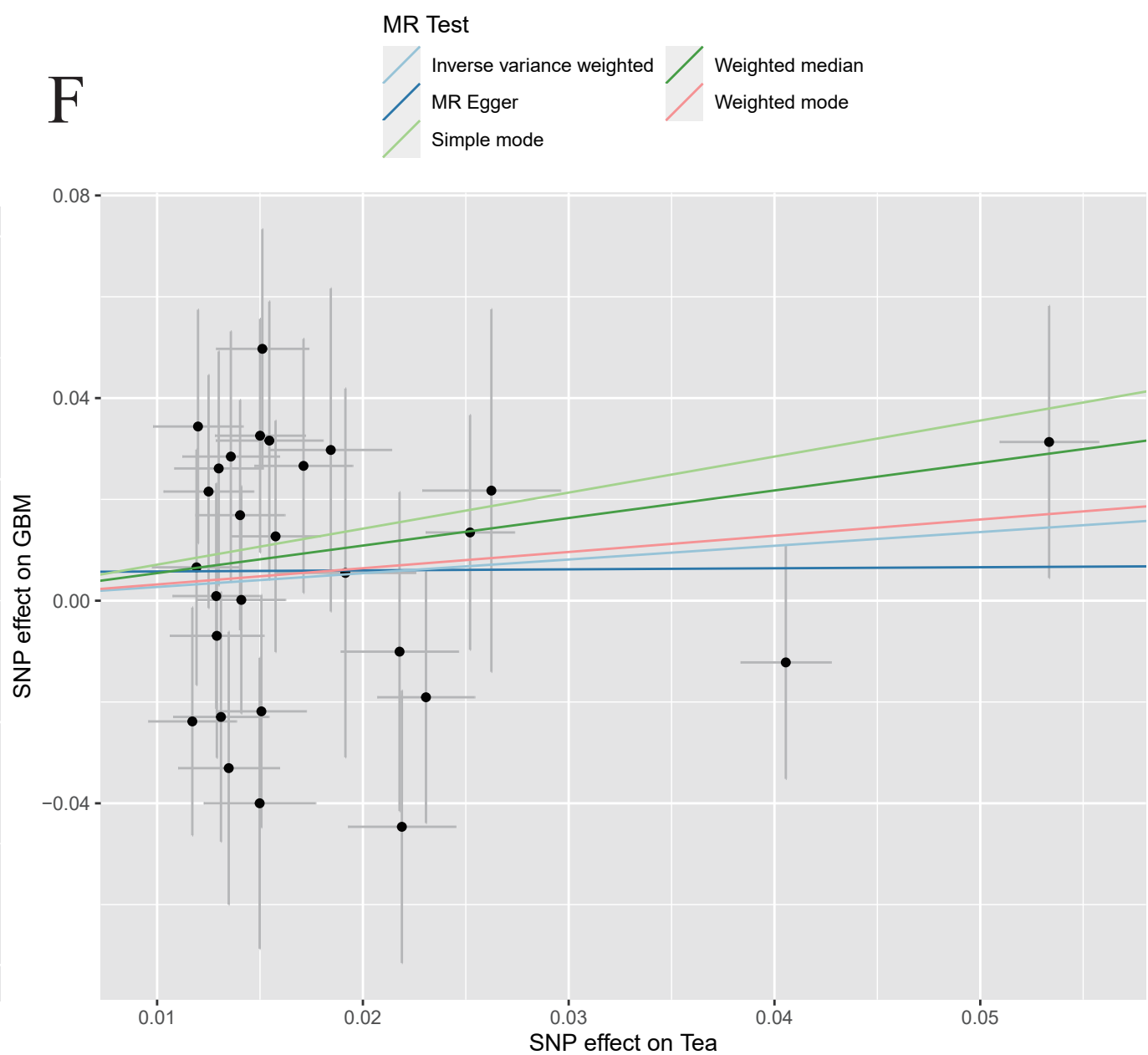

G

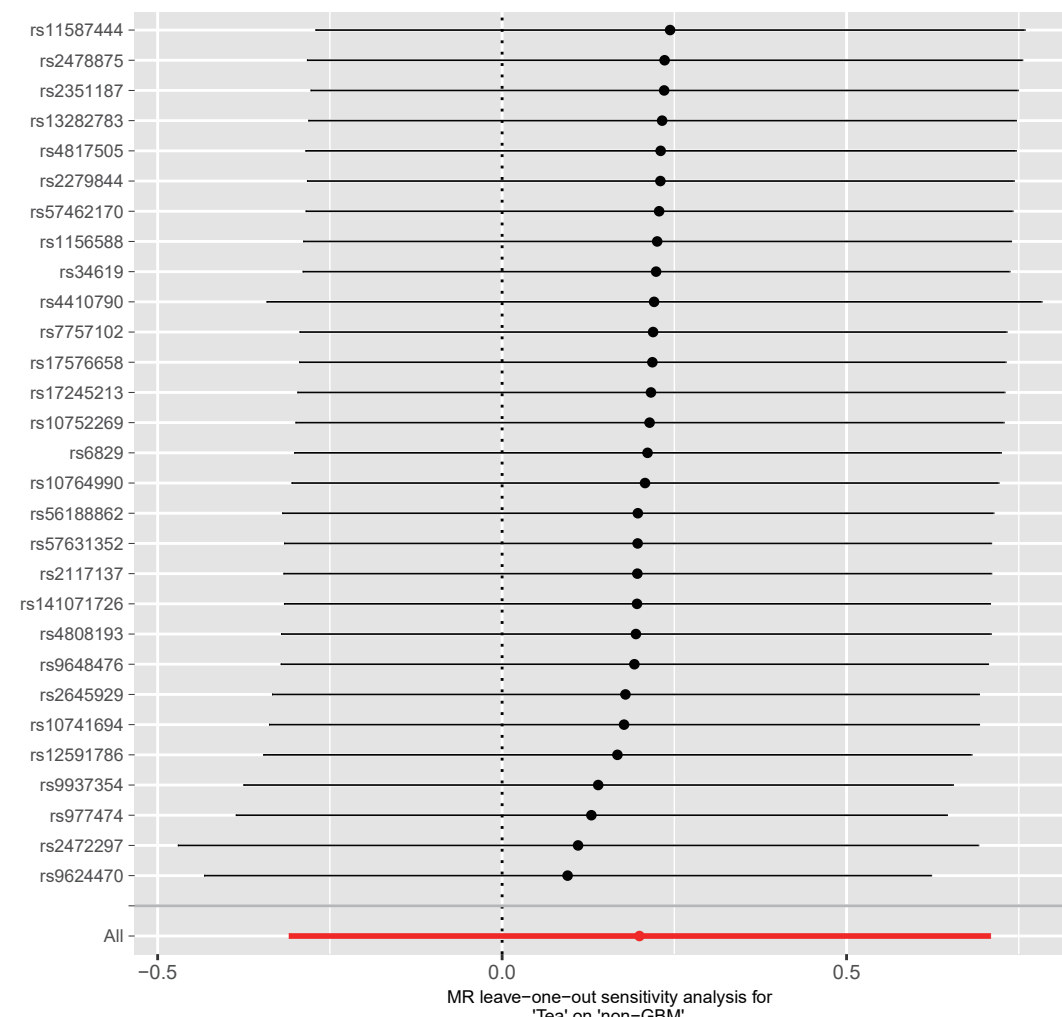

H

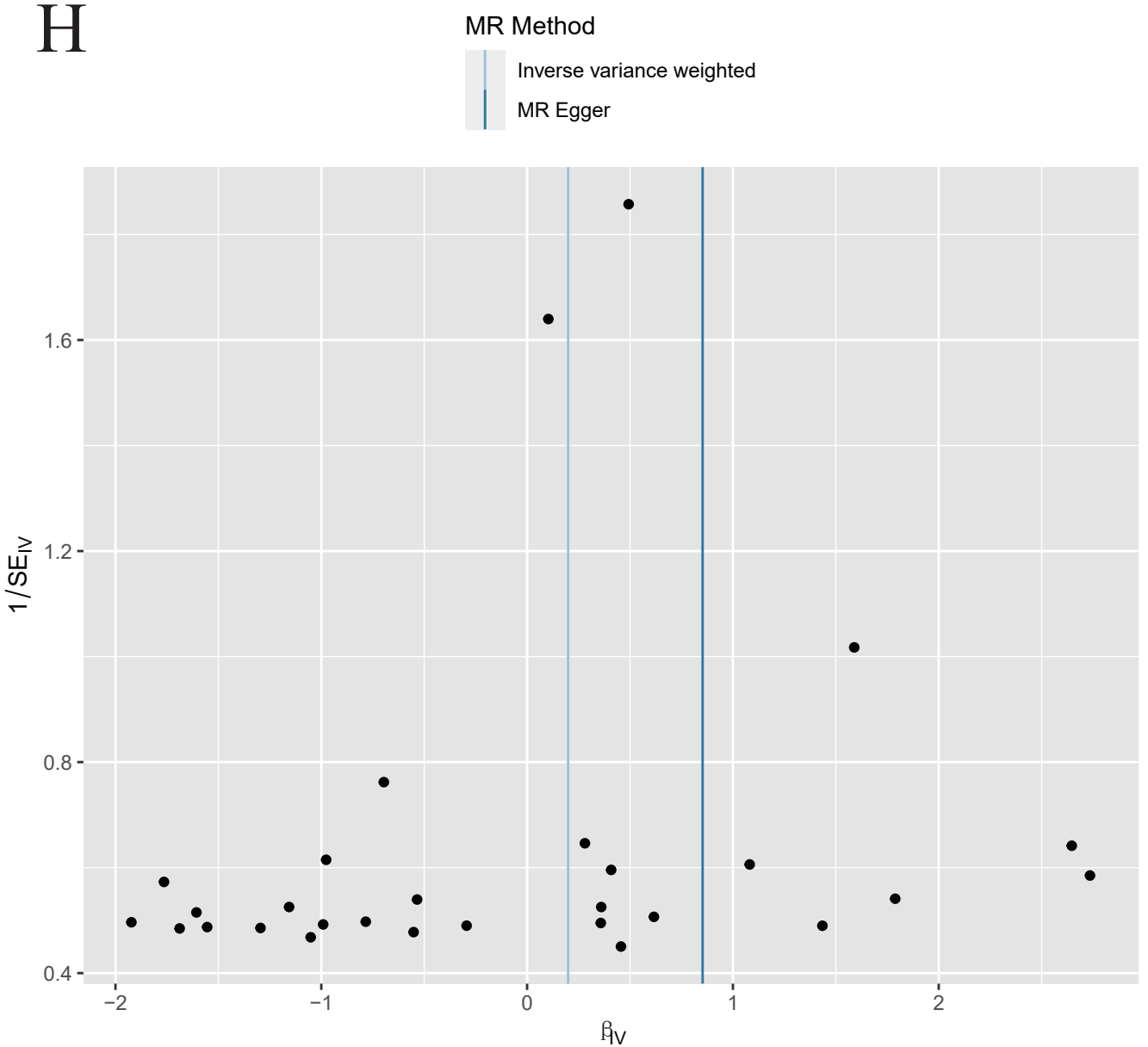

I

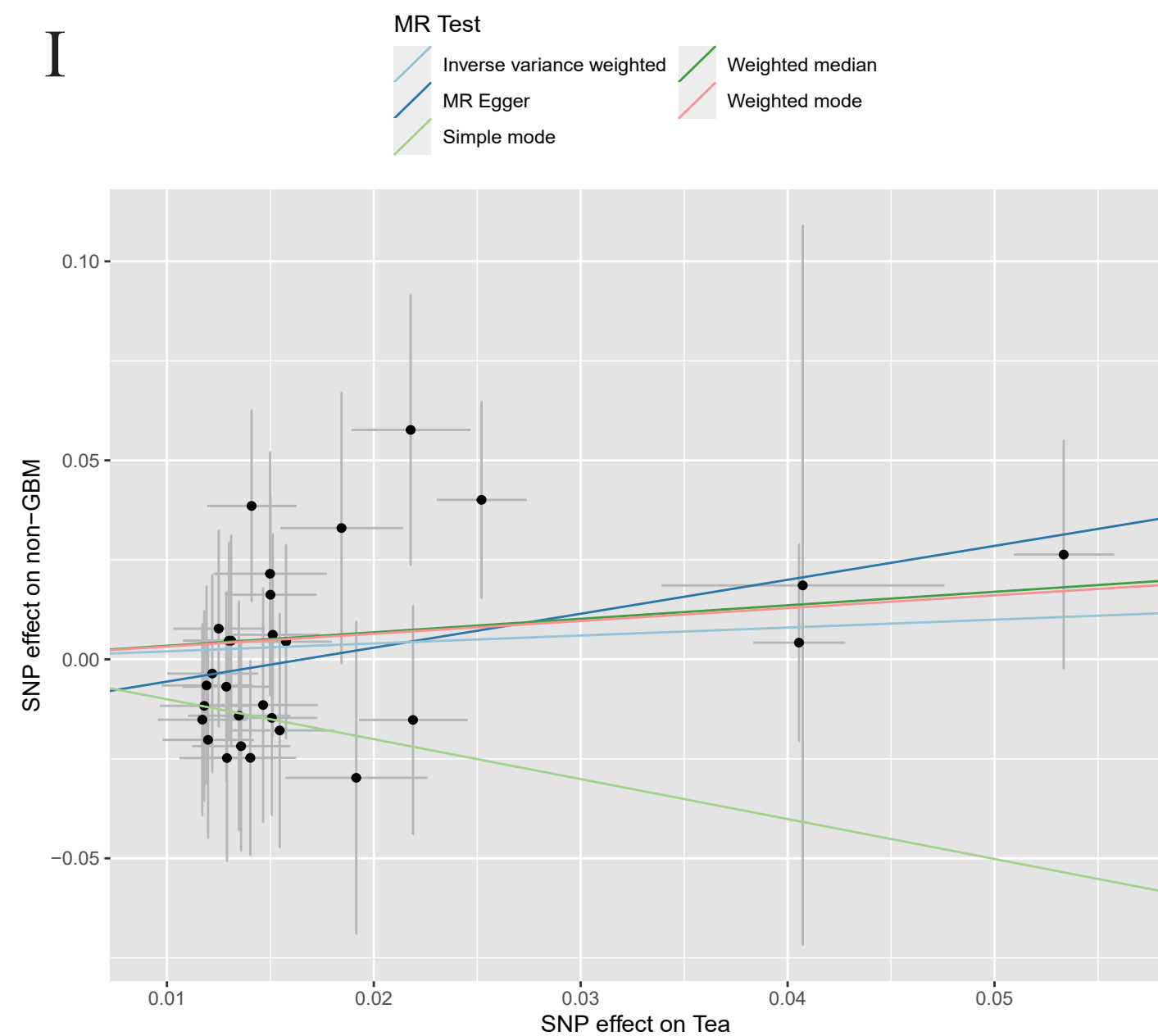

Supplement: Supplementary file 1 [file nutrients-17-00582-s001.zip › nutrients-3462880-supplementary/Sup_6.pdf]

A

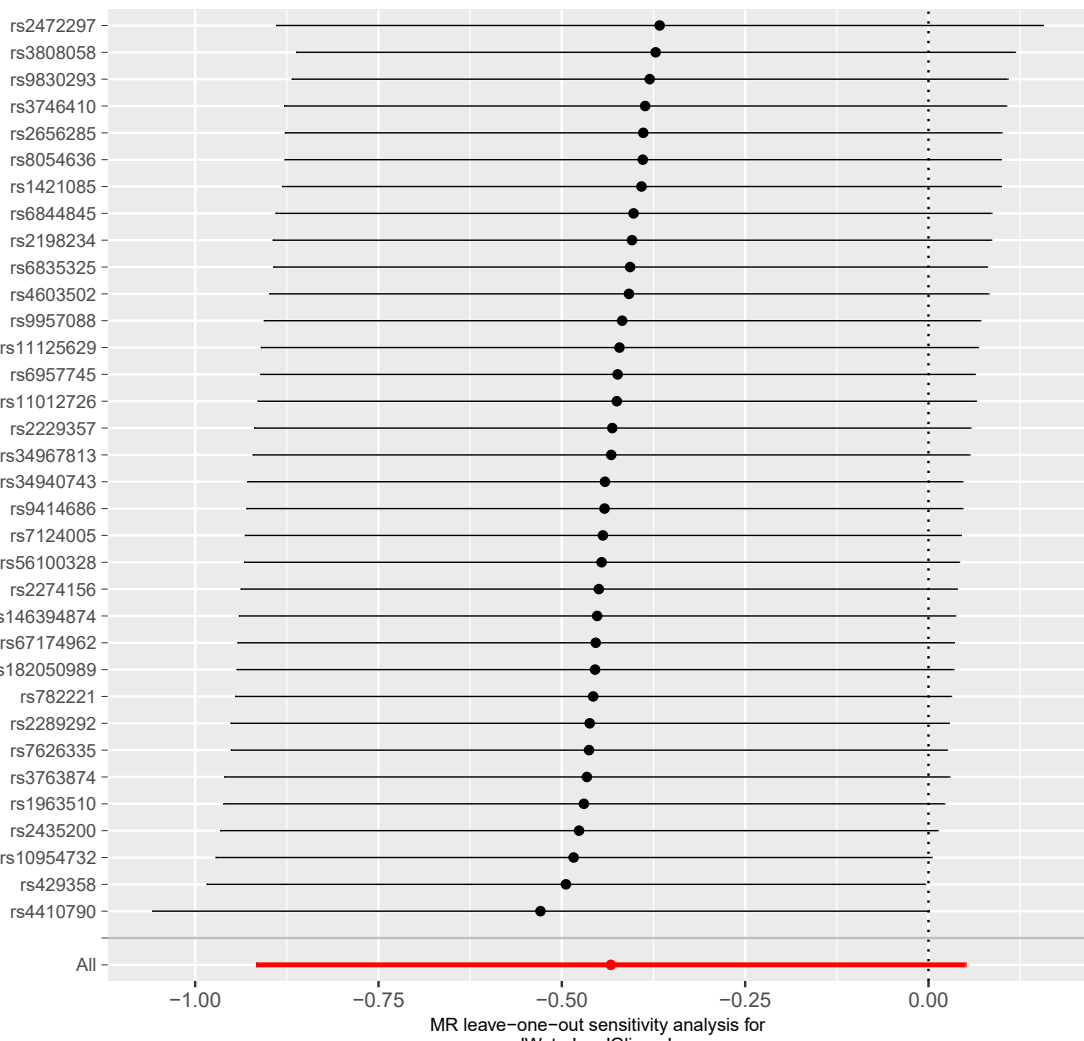

B

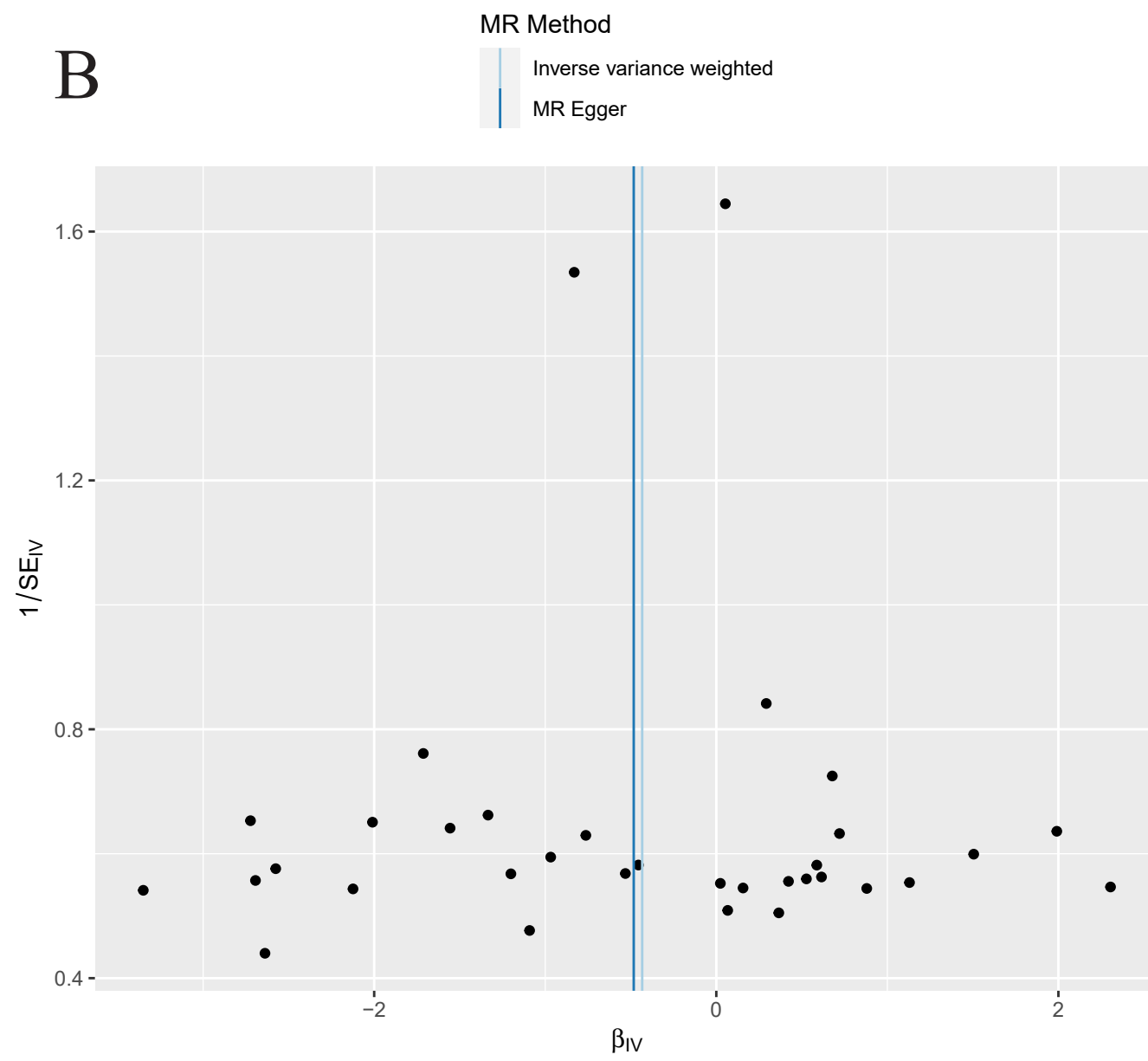

C

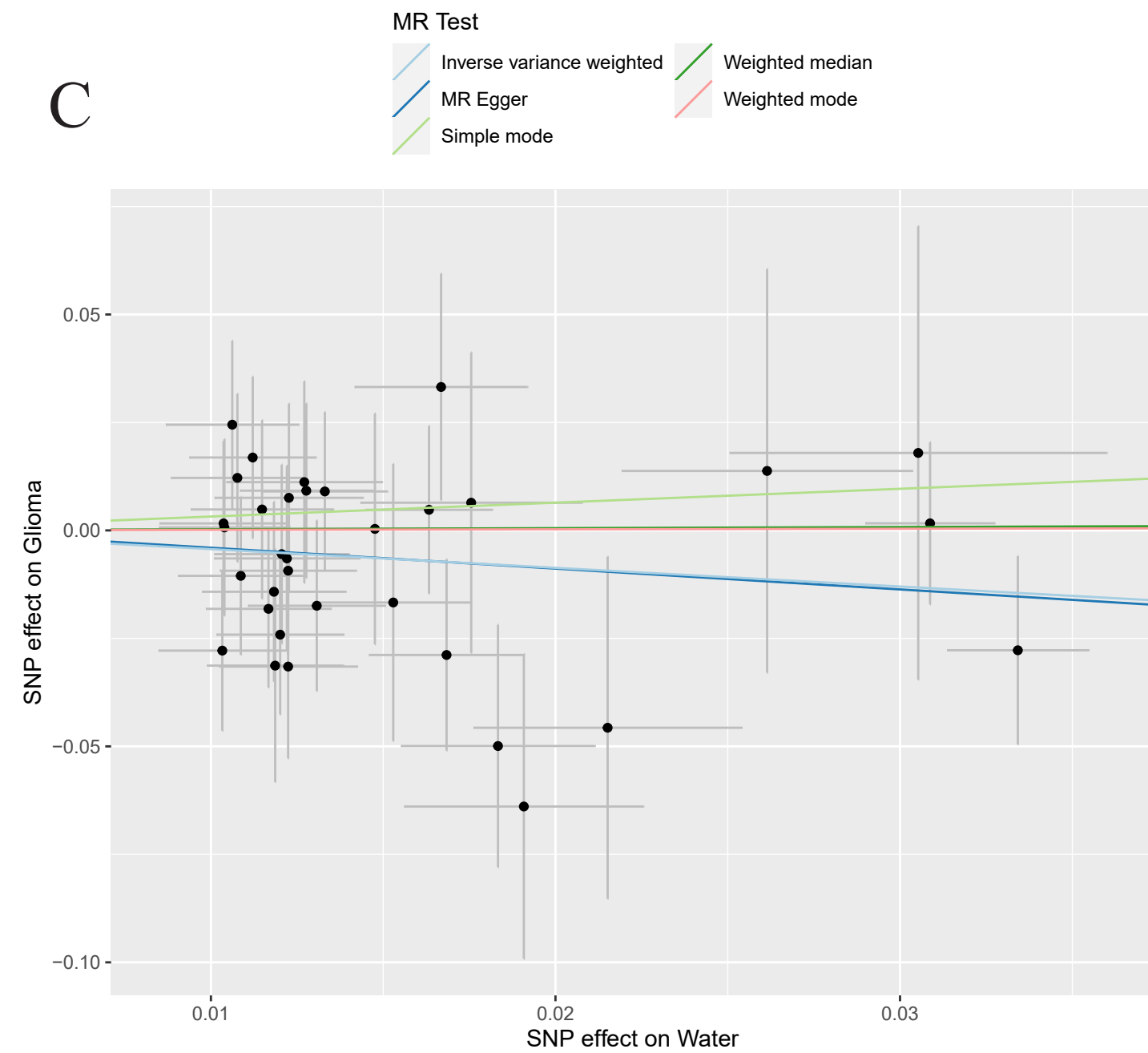

D

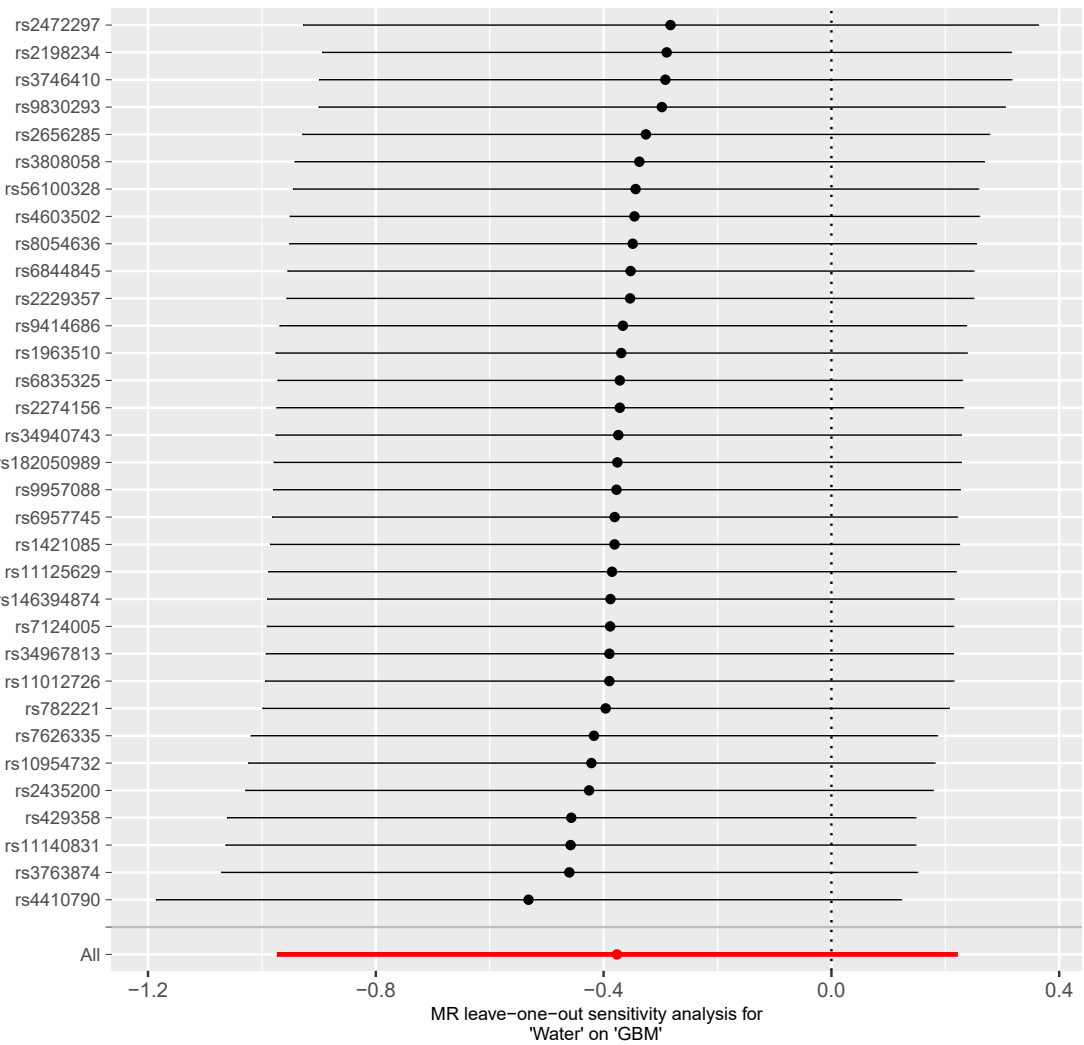

E

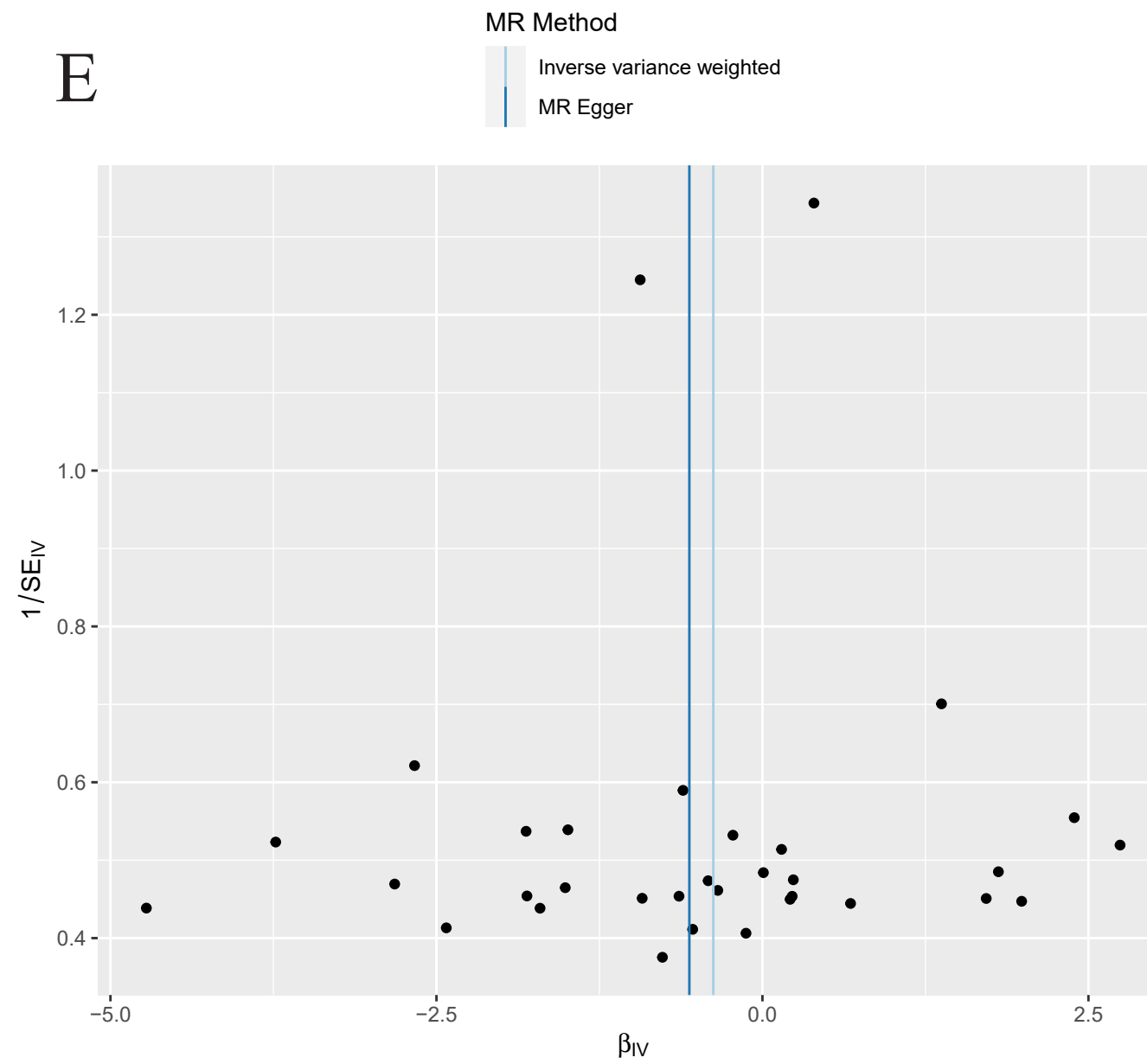

F

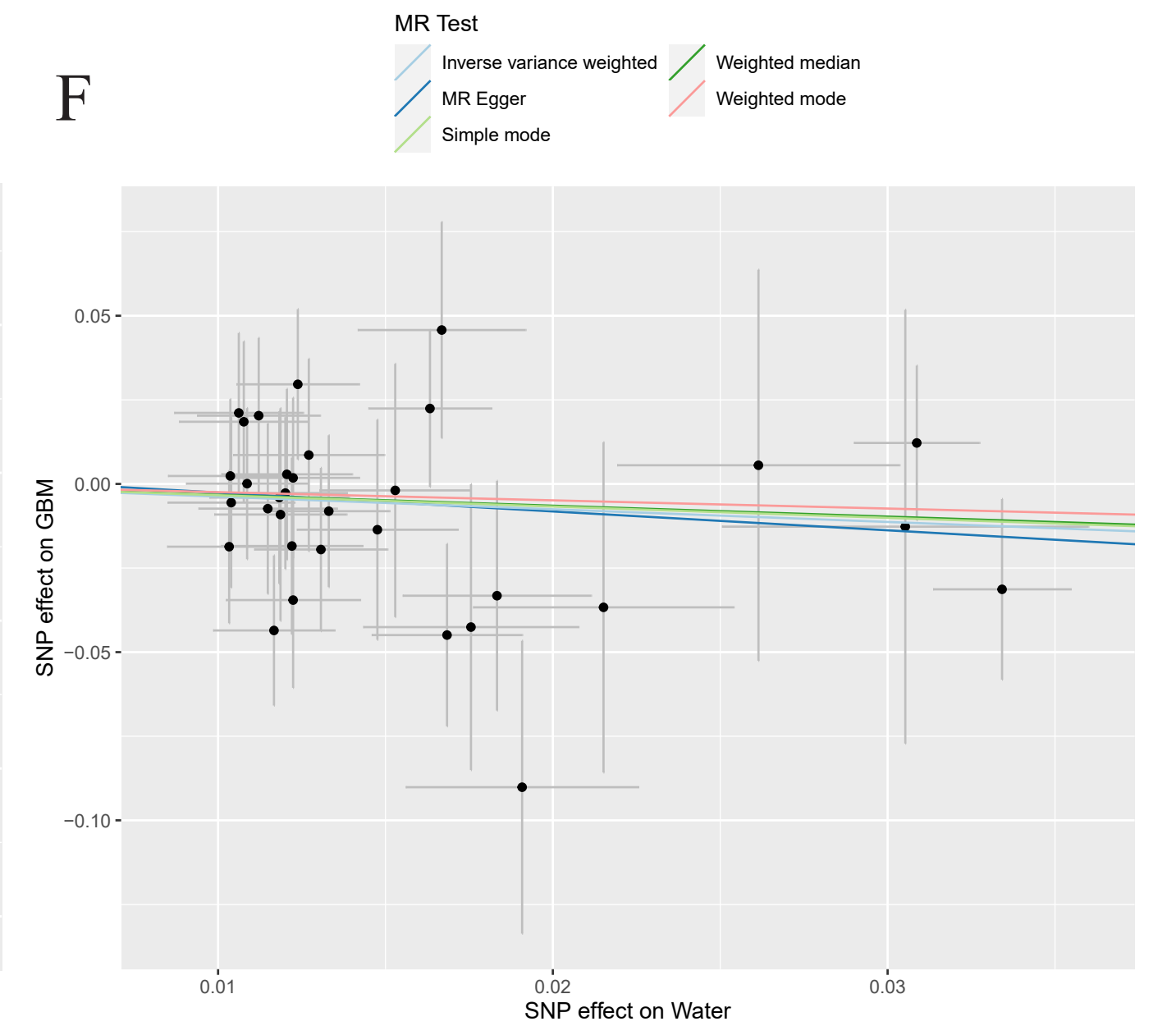

G

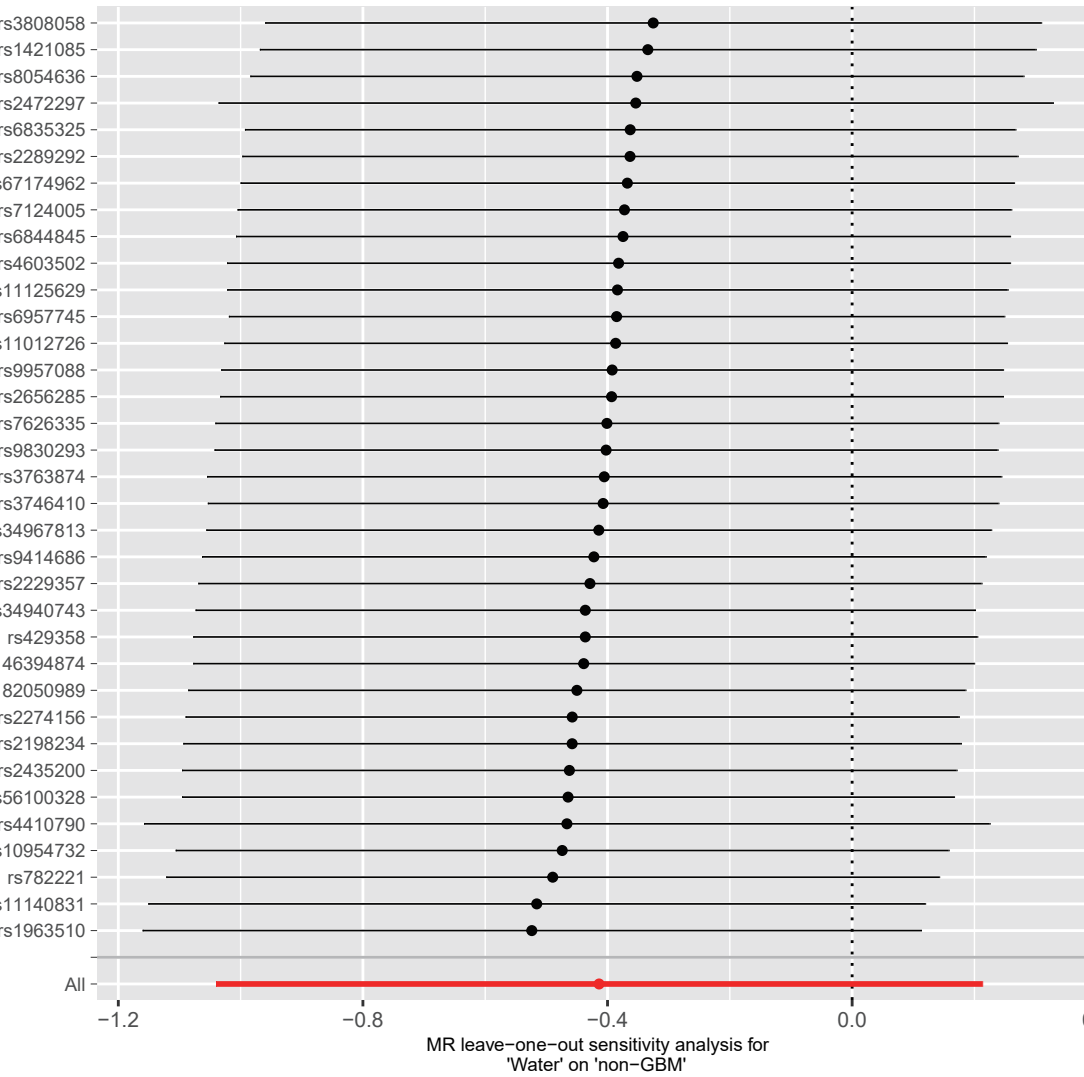

H

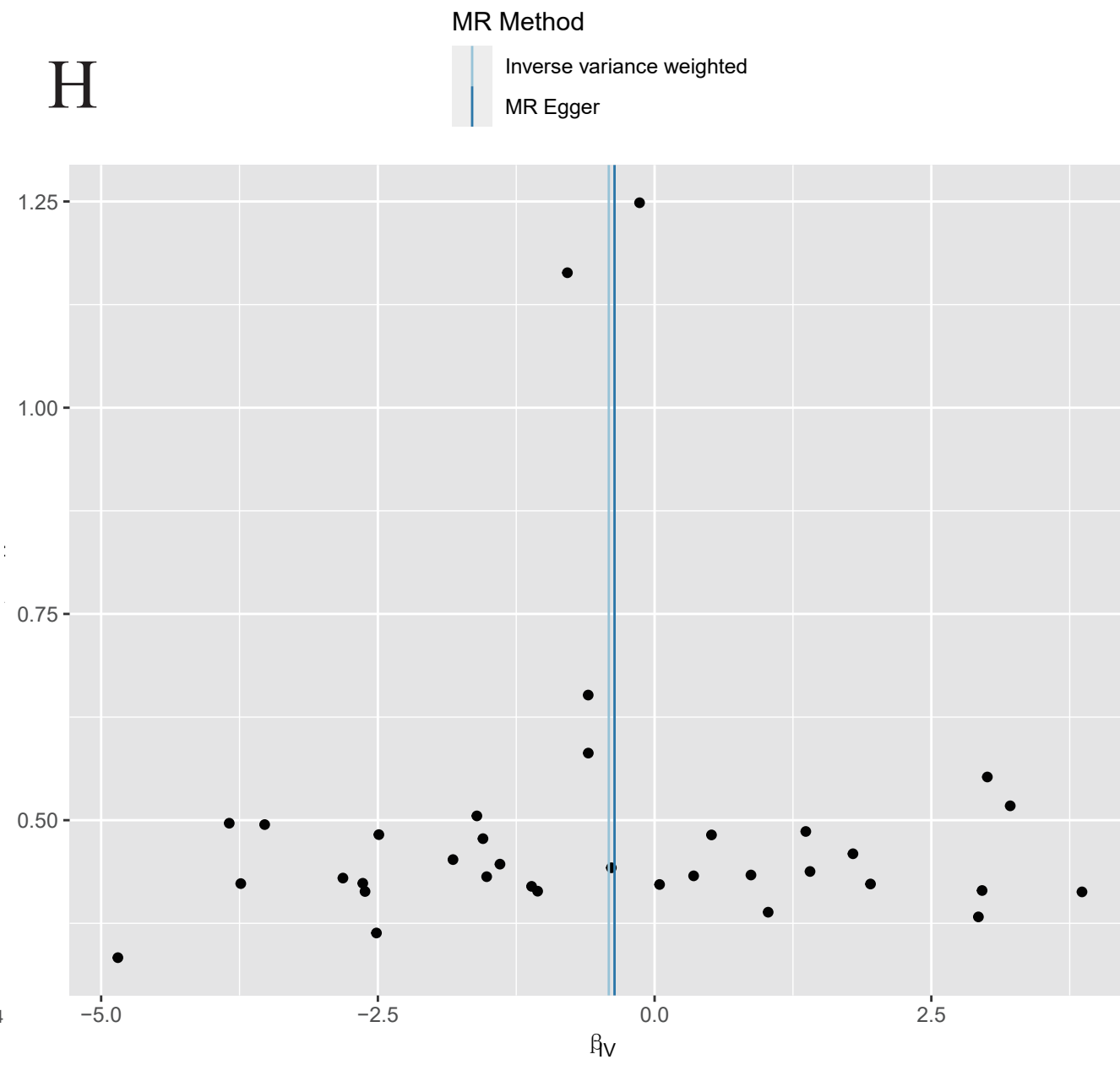

I

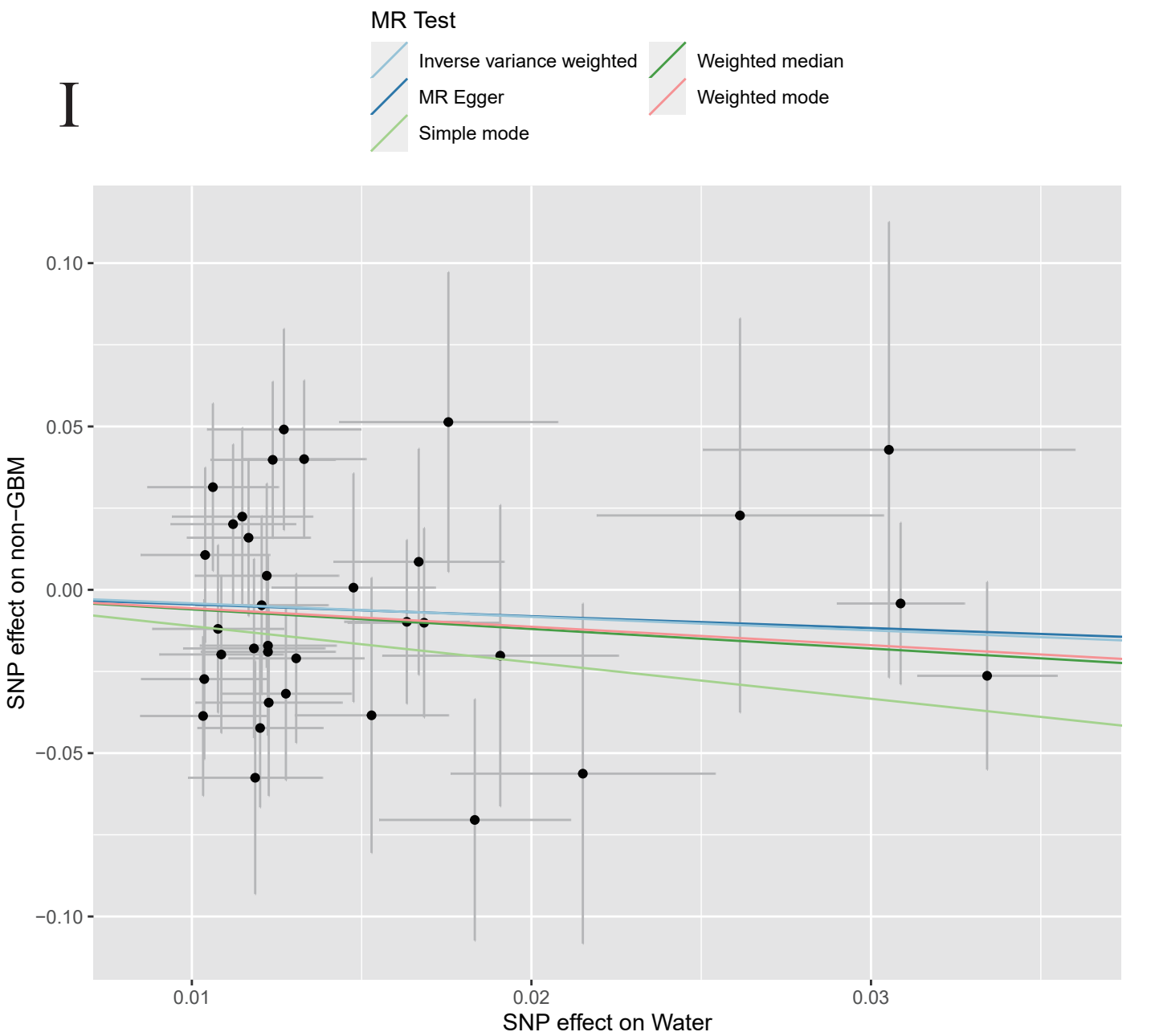

Supplement: Supplementary file 1 [file nutrients-17-00582-s001.zip › nutrients-3462880-supplementary/Sup_7.pdf]

A

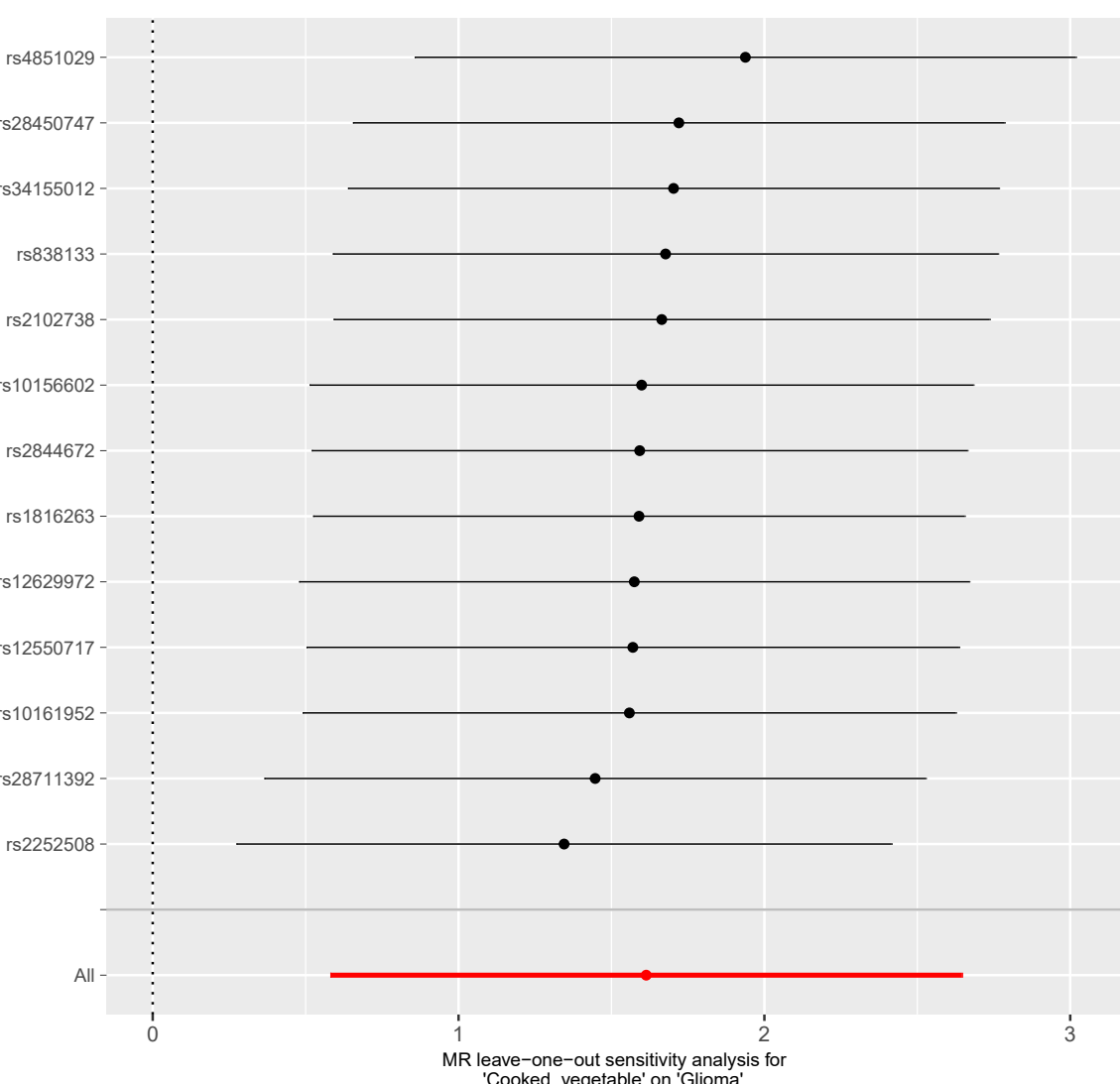

B

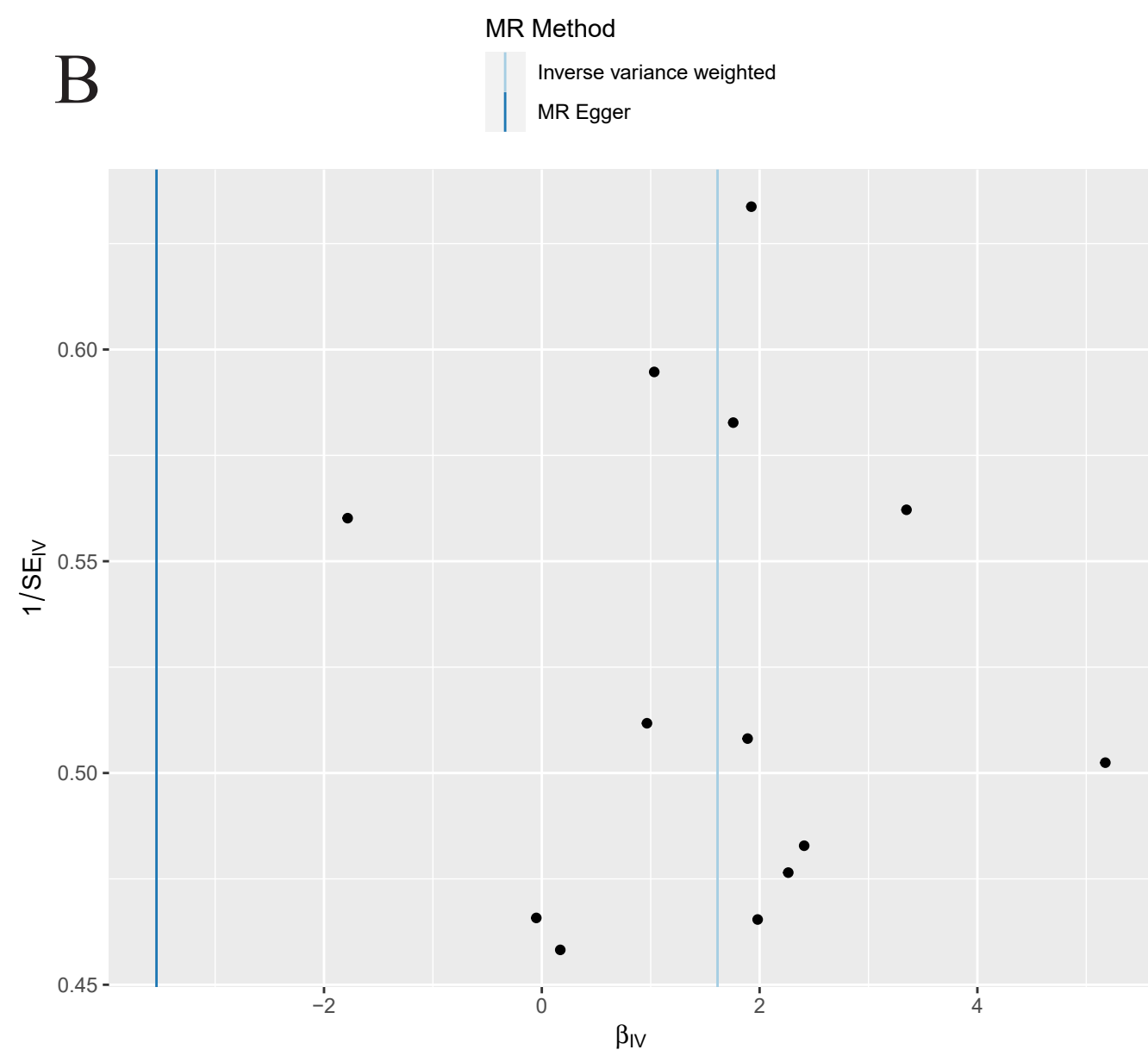

C

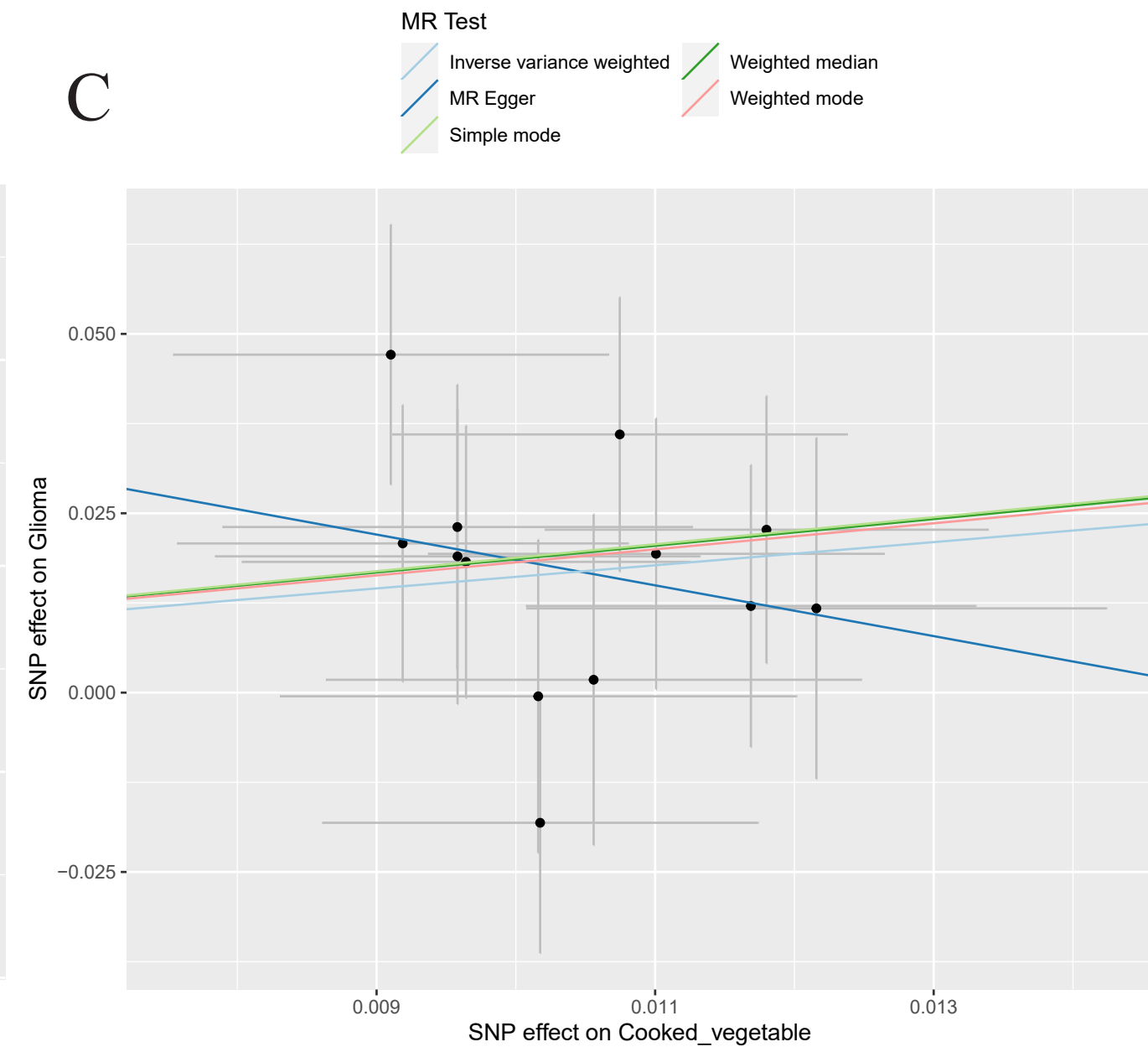

D

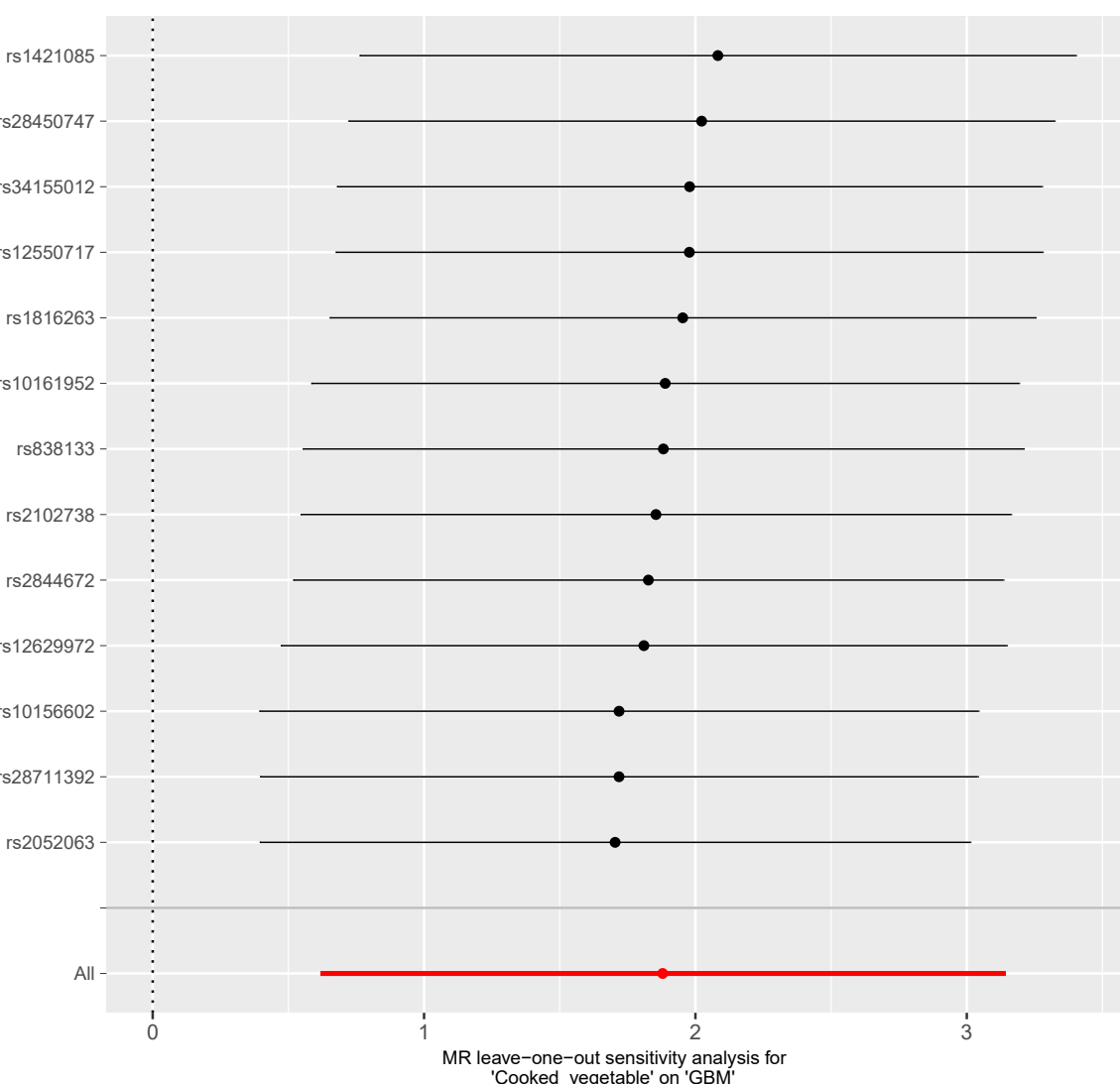

E

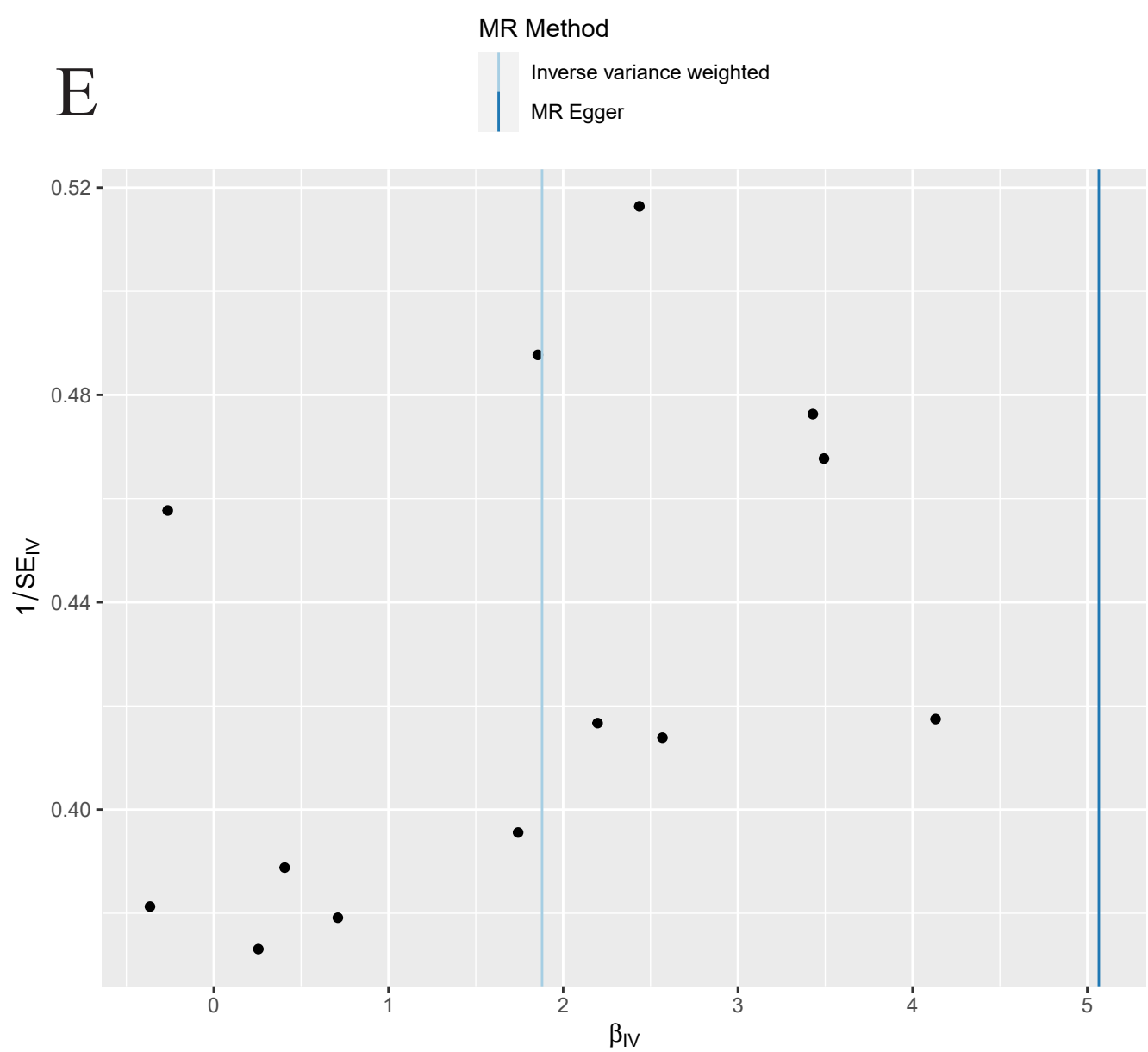

F

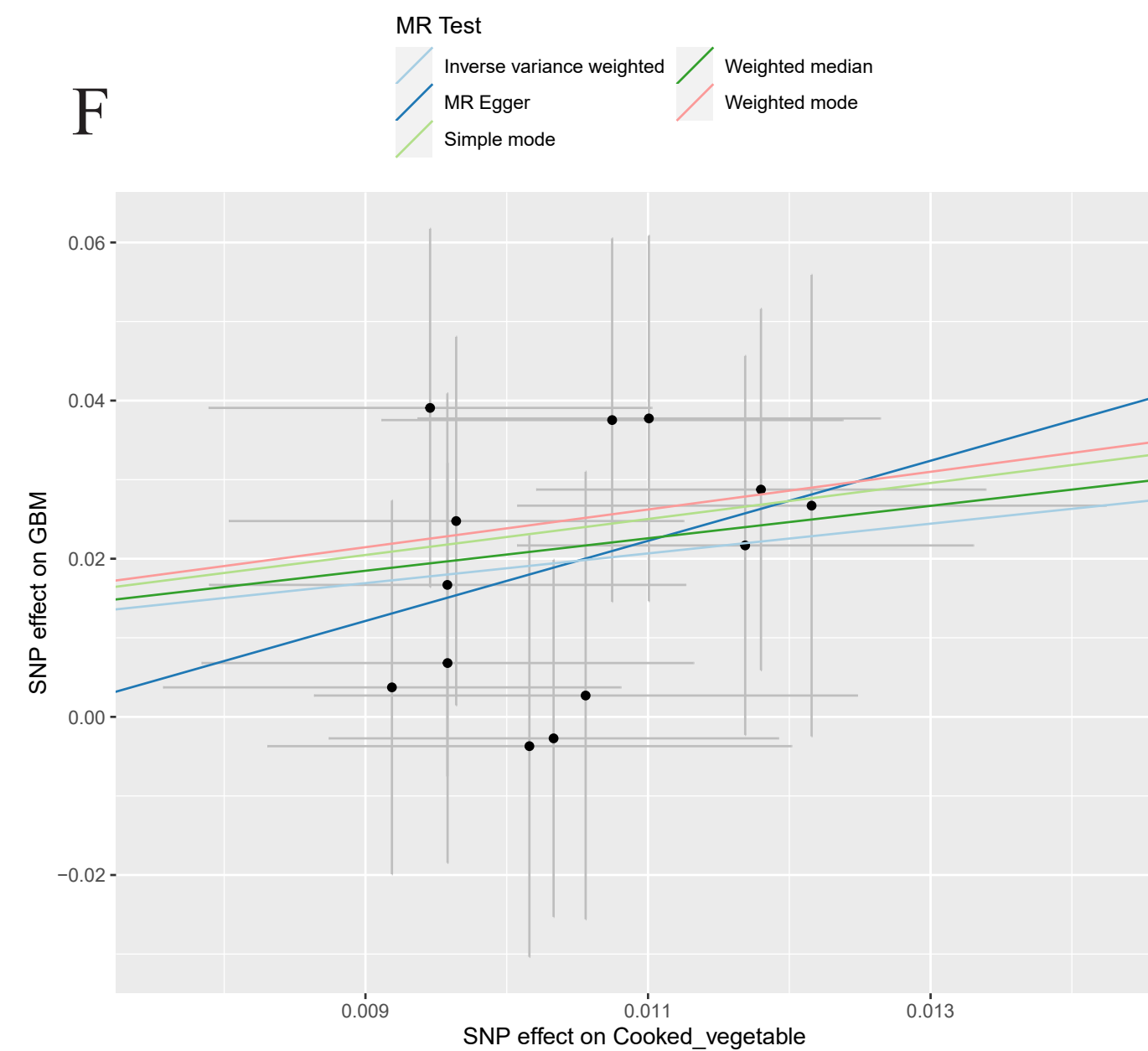

G

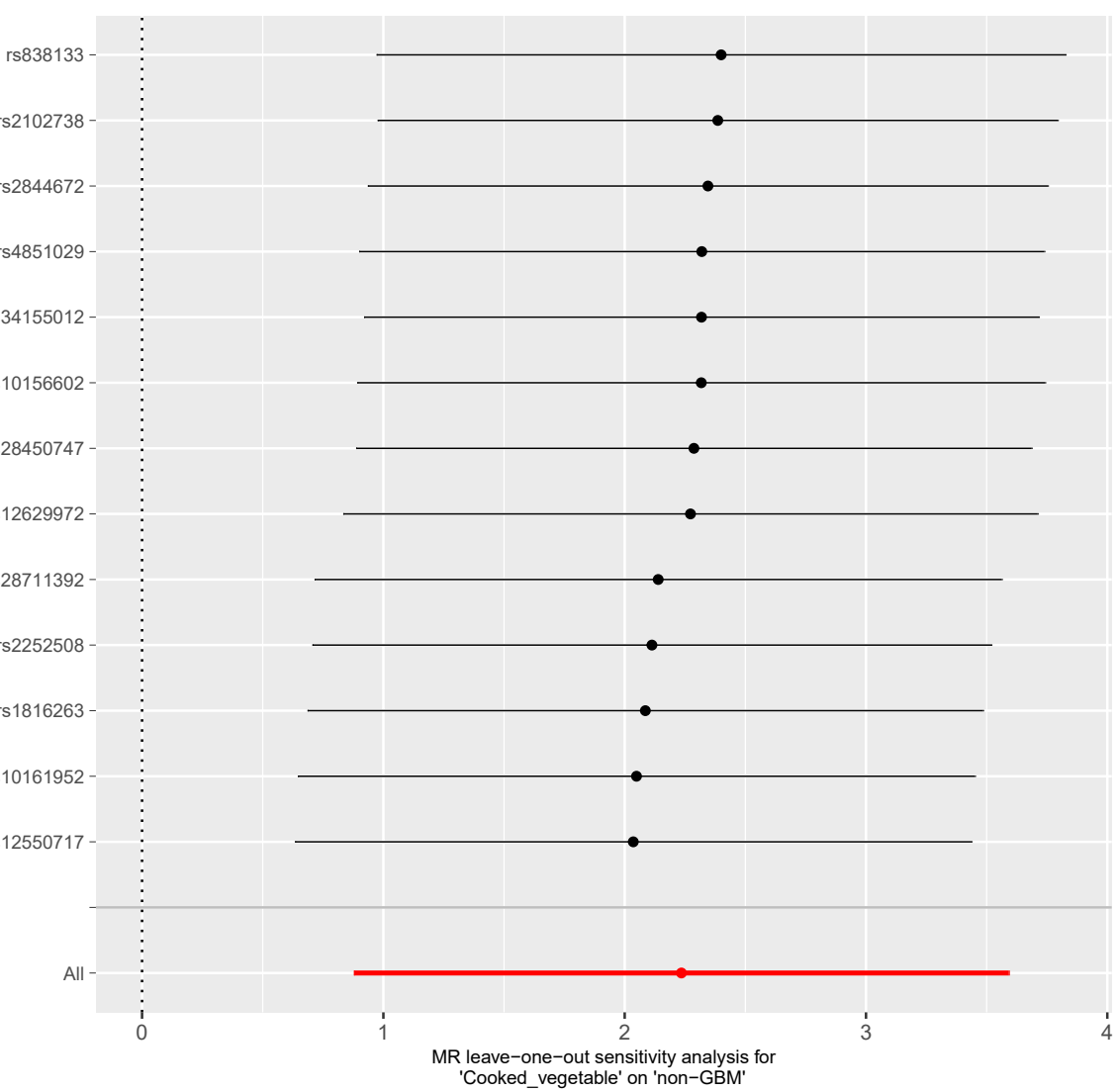

H

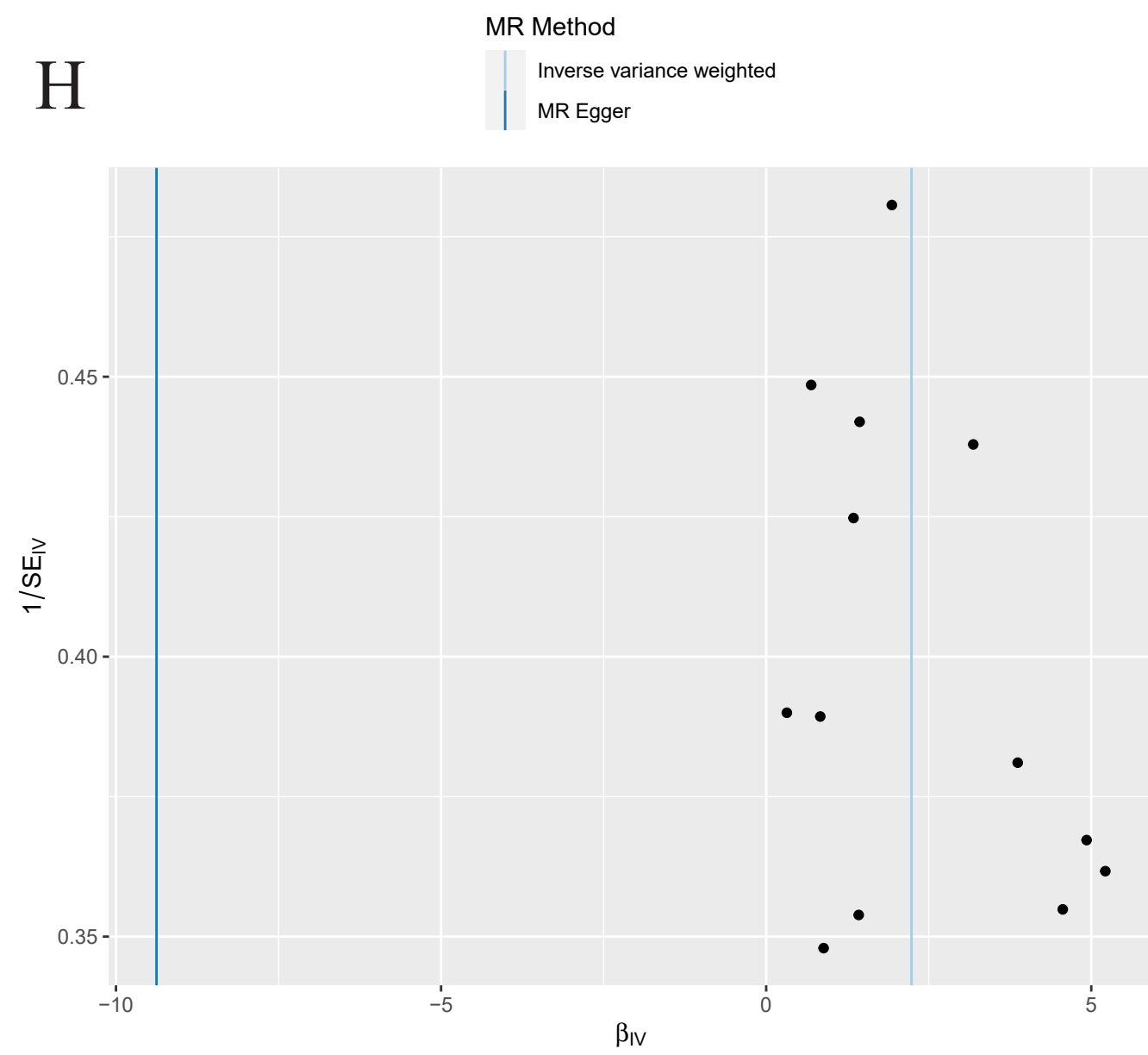

I

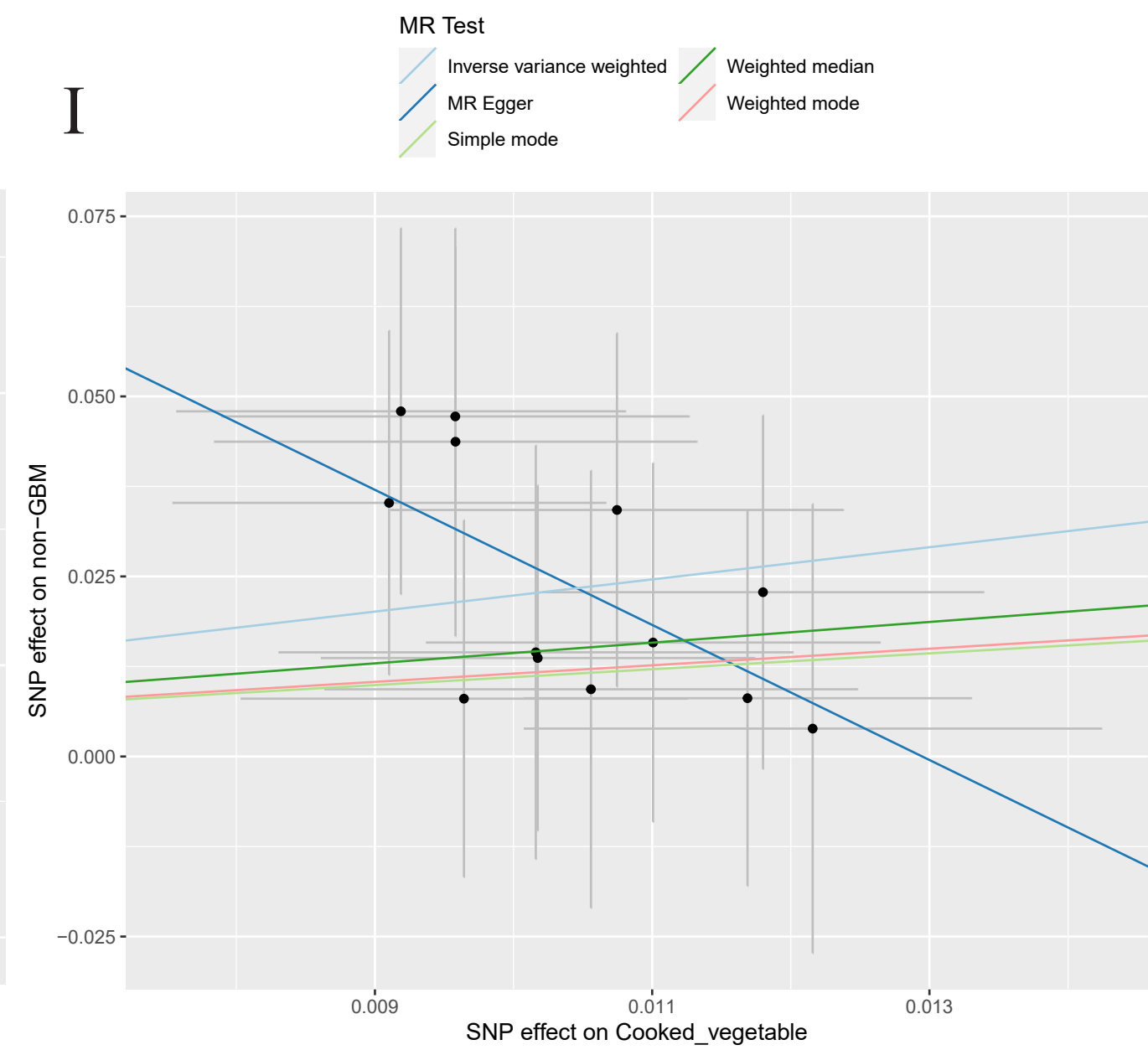

Supplement: Supplementary file 1 [file nutrients-17-00582-s001.zip › nutrients-3462880-supplementary/Sup_8.pdf]

A

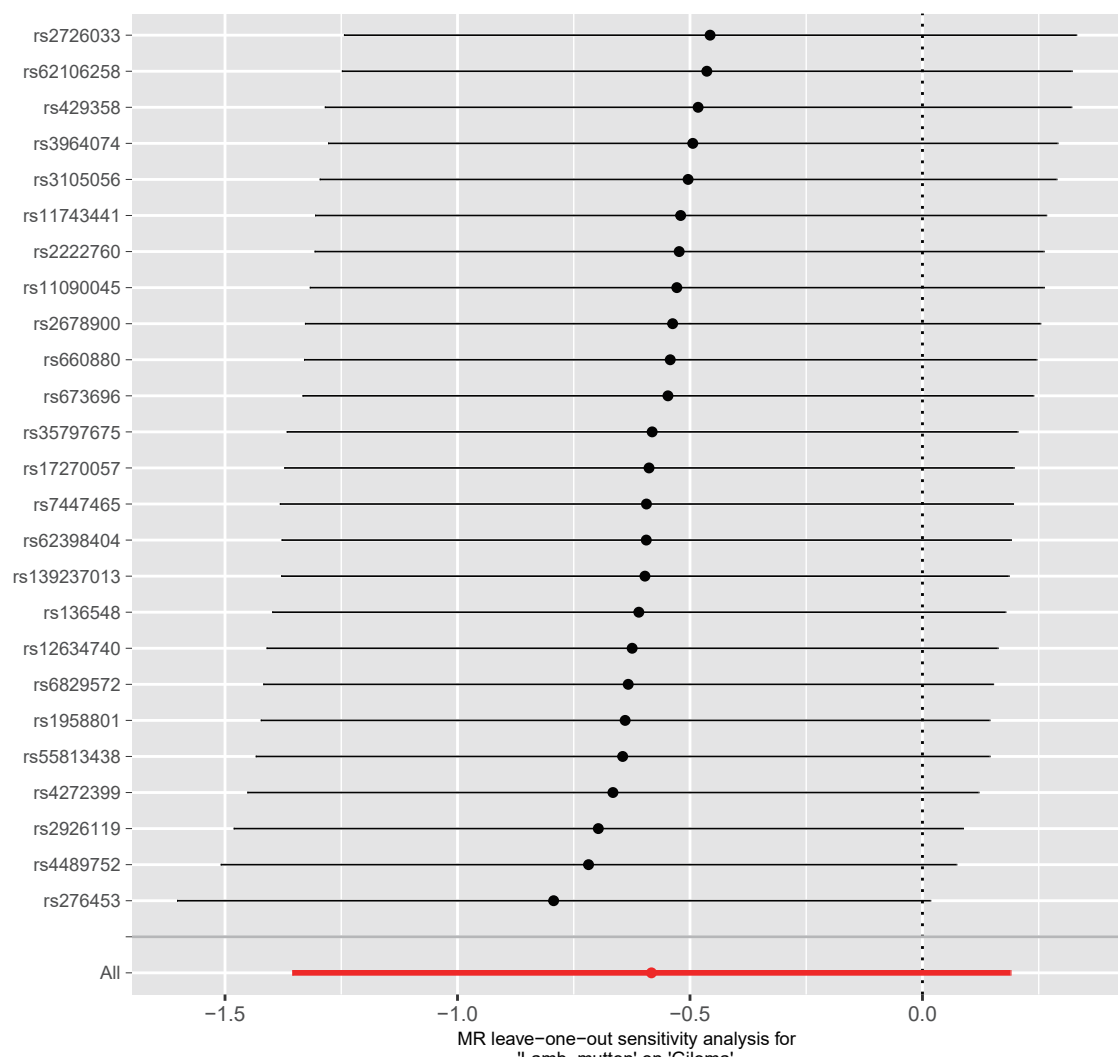

B

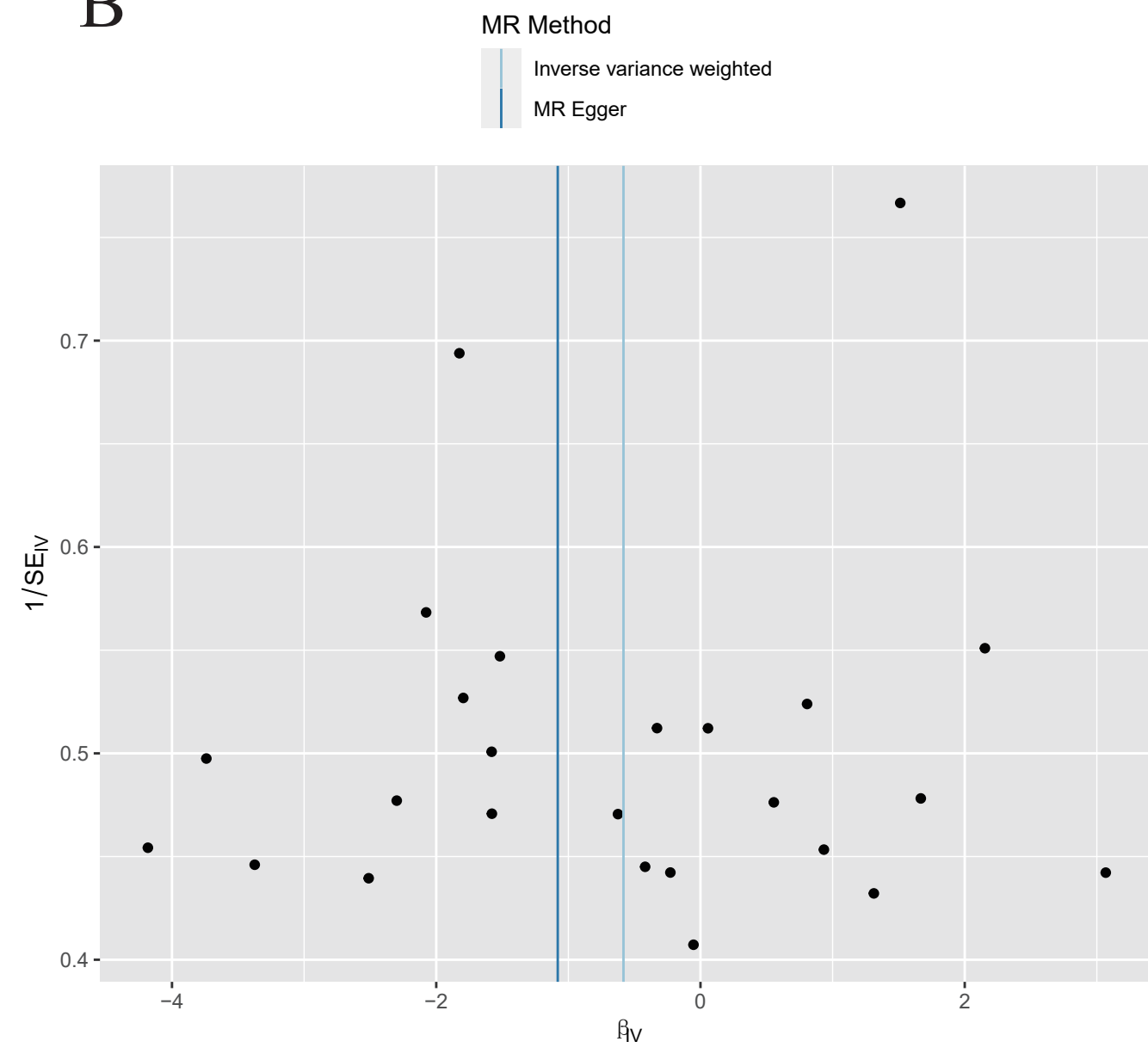

C

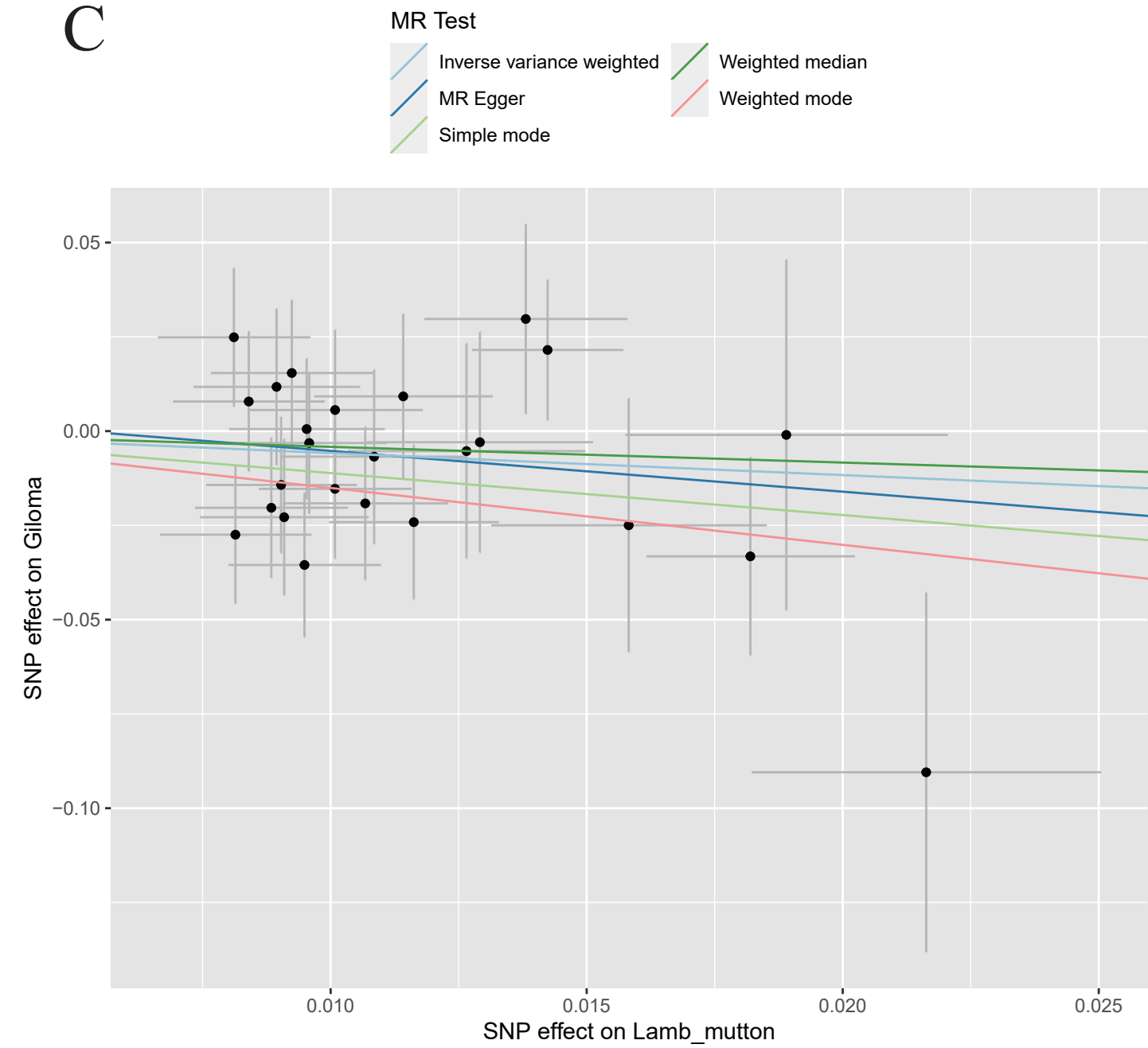

D

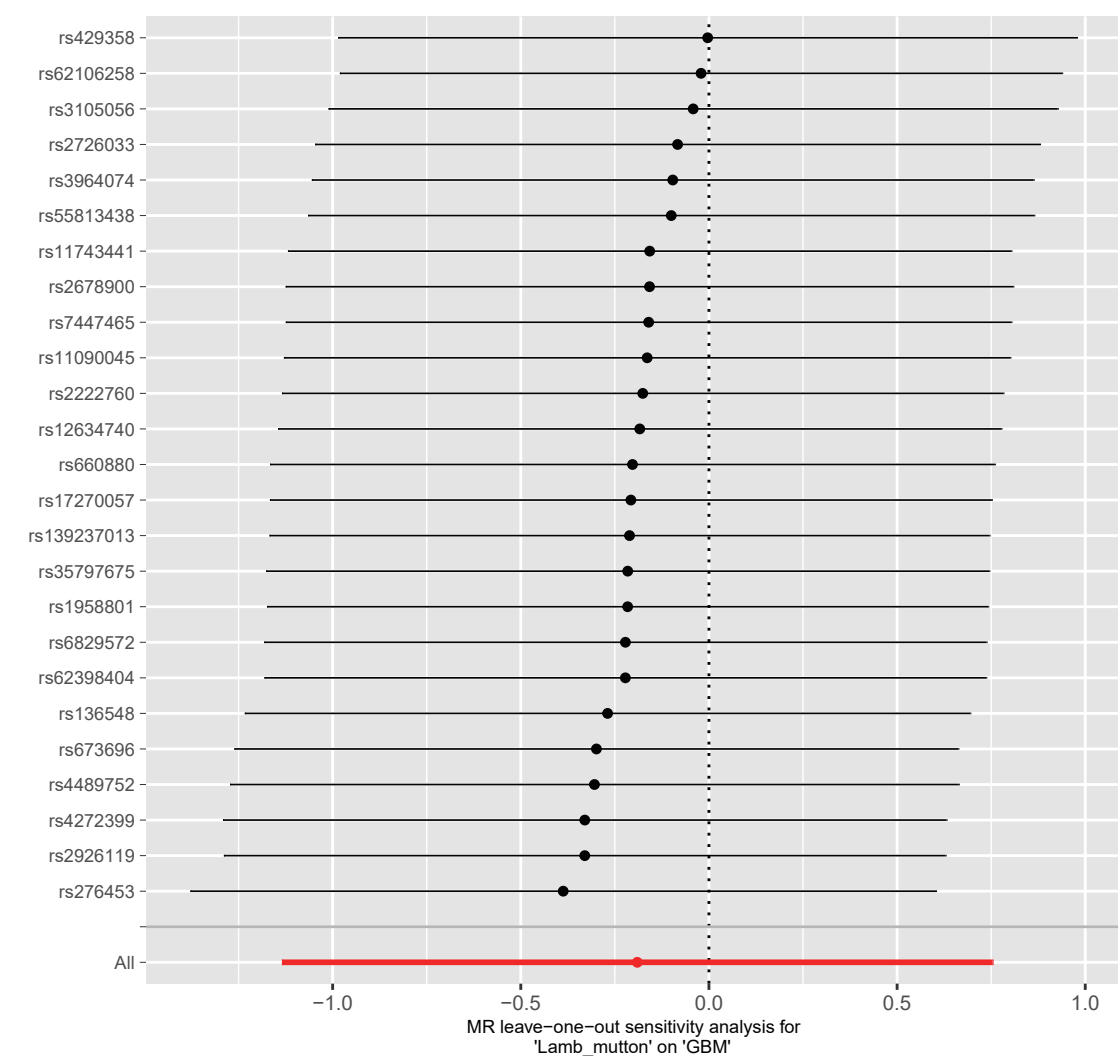

E

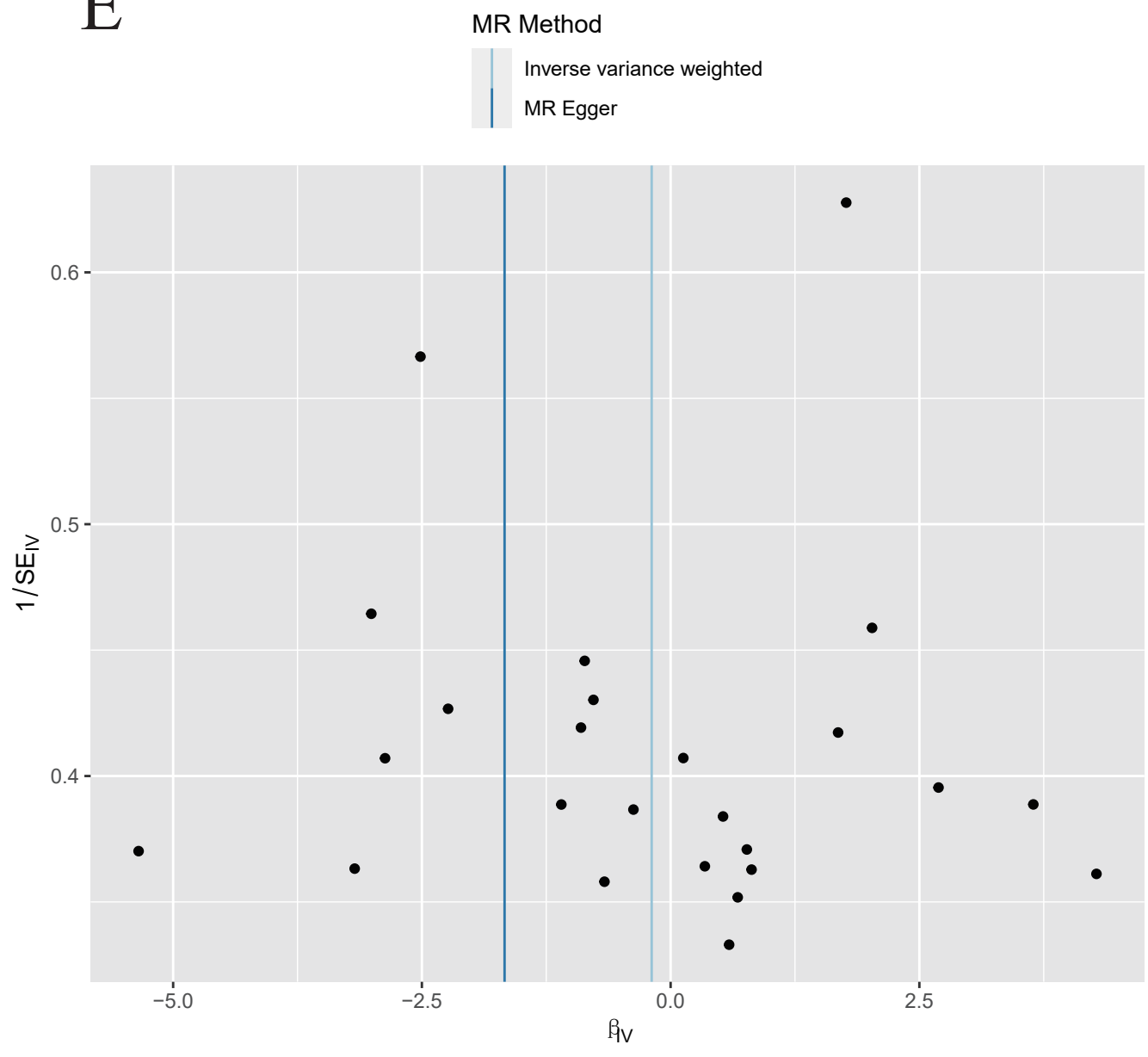

F

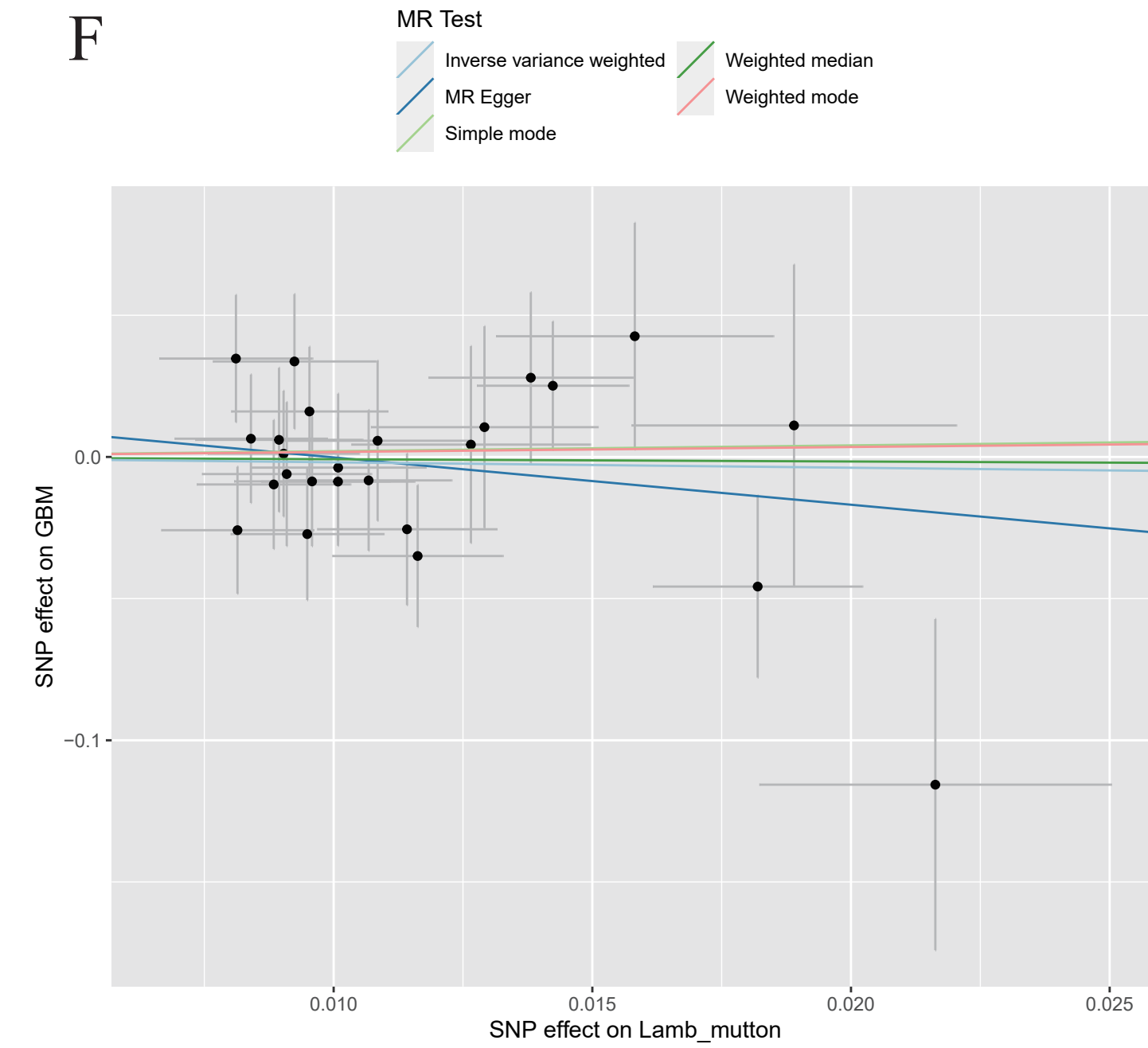

G

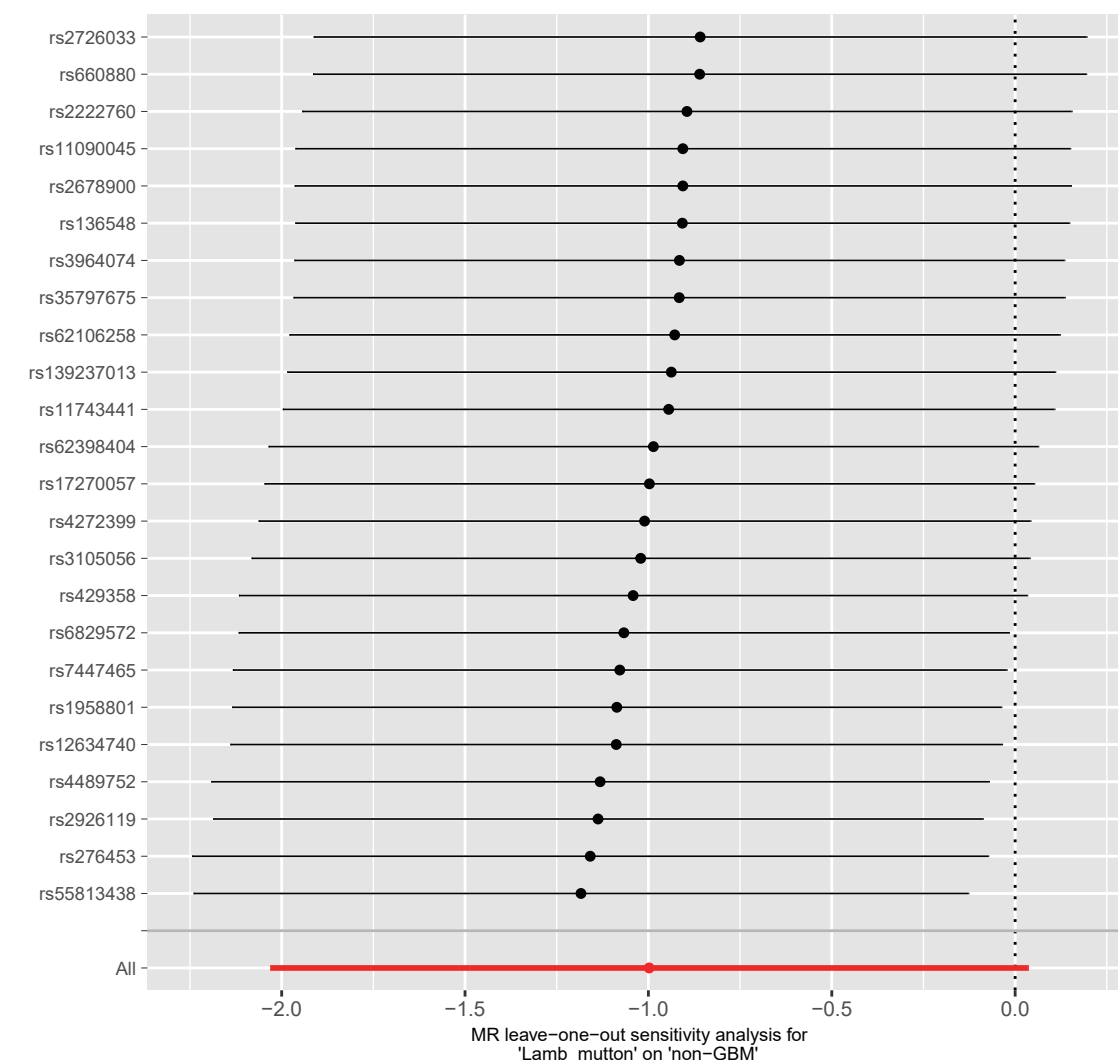

H

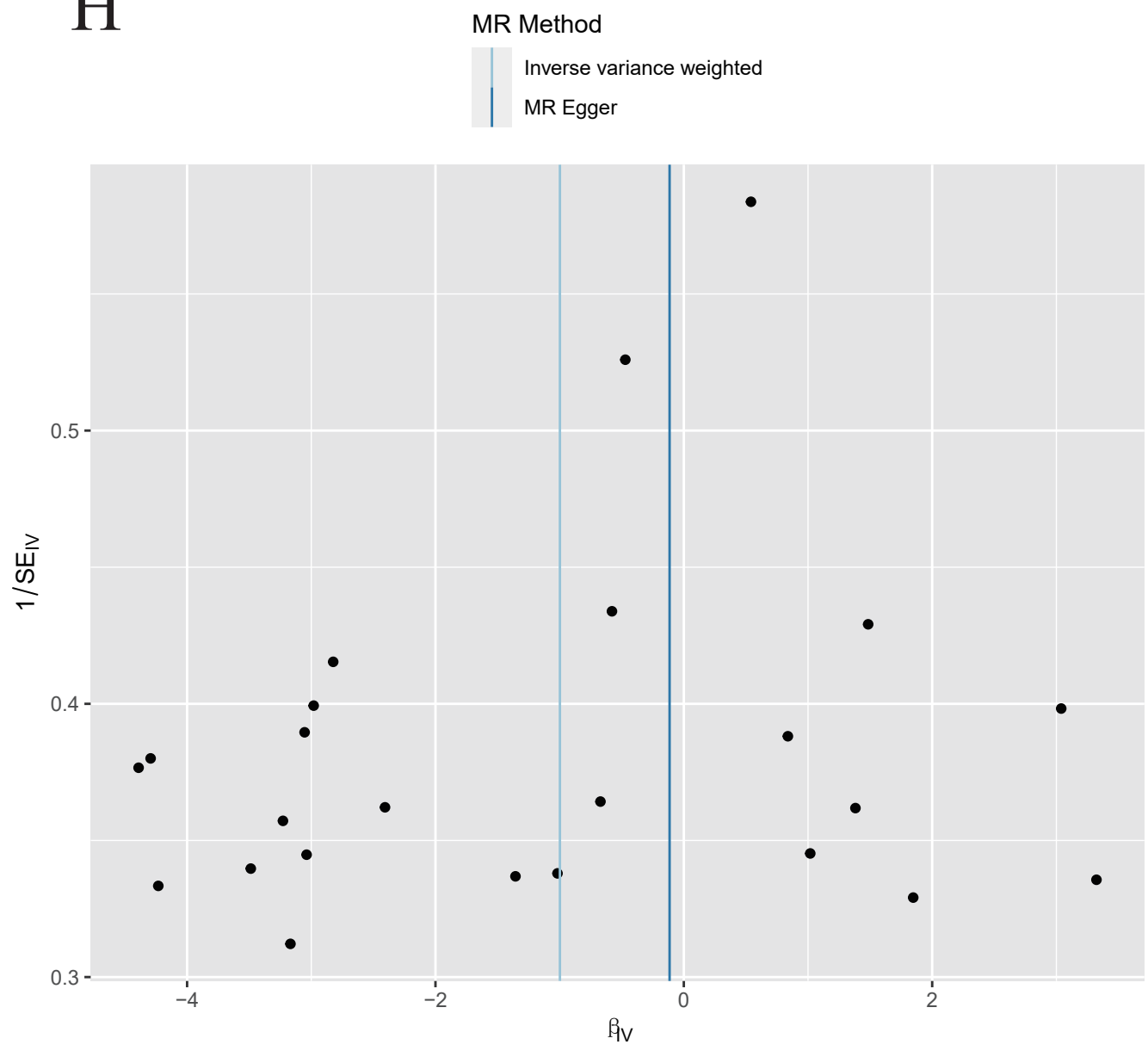

I

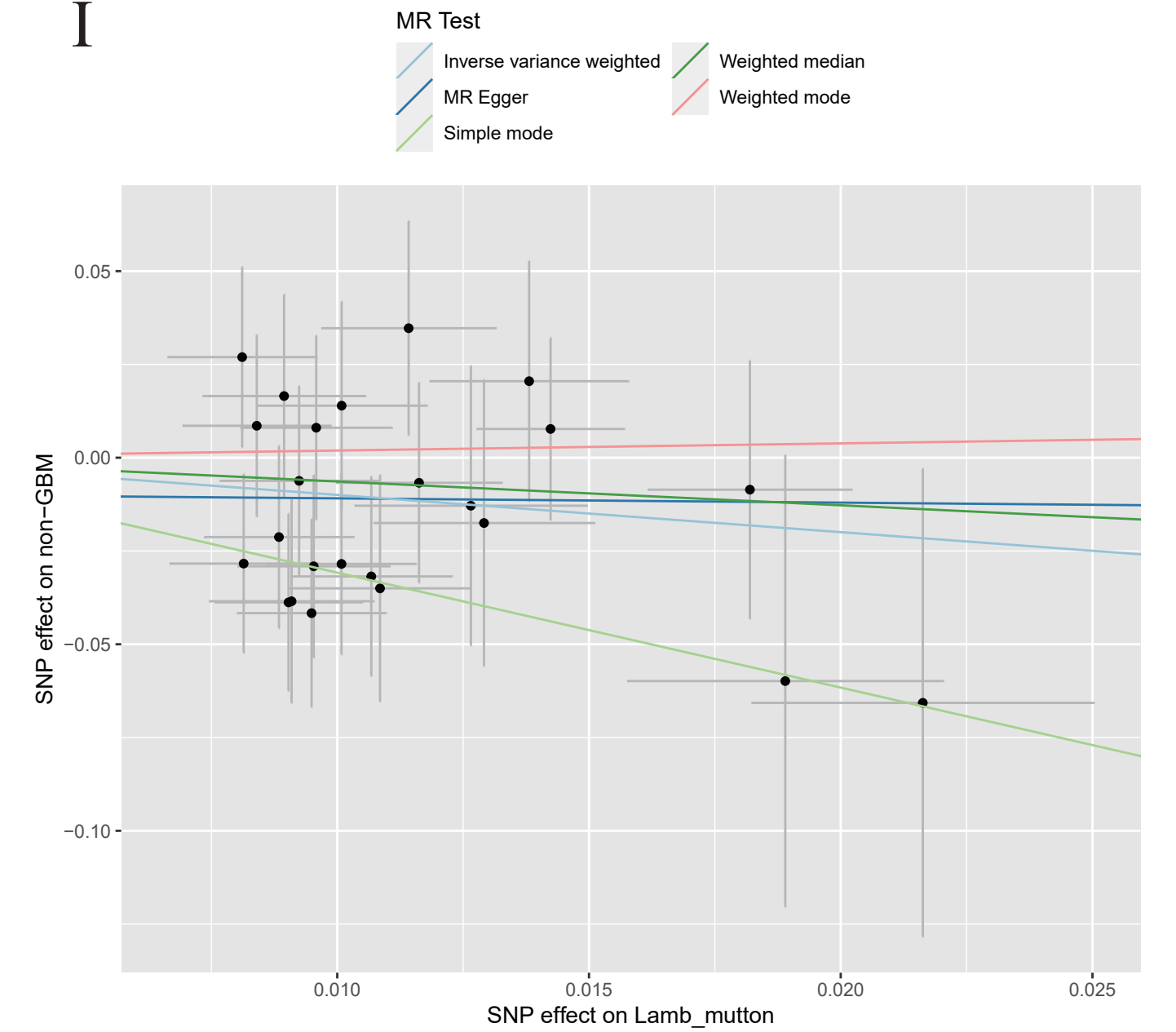

Supplement: Supplementary file 1 [file nutrients-17-00582-s001.zip › nutrients-3462880-supplementary/Sup_9.pdf]
